# Effects of elastic band resistance training on the physical and mental health of elderly individuals: A mixed methods systematic review

**DOI:** 10.1371/journal.pone.0303372

**Published:** 2024-05-13

**Authors:** Aiying Li, Yan Sun, Meng Li, Dongyang Wang, Xiaofeng Ma

**Affiliations:** 1 Sichuan Academy of Medical Sciences & Sichuan Provincial People’s Hospital, Chengdu, Sichuan, China; 2 Chengdu University of Traditional Chinese Medicine, Chengdu, Sichuan, China; 3 The Philippines Women’s University, Manila, Metro Manila, Philippines; 4 The Third People’s Hospital of Hanan Provinnce, Zhengzhou, Henan, China; 5 Binzhou Medical University, Binzhou, Shangdong, China; 6 Henan University of Chinese Medicine, Zhengzhou, Henan, China; University of Montenegro, MONTENEGRO

## Abstract

**Objectives:**

Elastic band resistance training in elderly individuals can improve physical fitness and promote mental health in addition to other benefits. This systematic review aimed to review, summarize, and evaluate quantitative, qualitative, and mixed methodological studies on the use of elastic band resistance training in elderly individuals, and to investigate the influence of elastic band resistance training on the physical and mental health of elderly individuals, as well as their preferences and obstacles in training.

**Methods:**

A convergent separation approach was used to synthesize and integrate the results, specifically the mixed systematic review approach recommended by the Joanna Briggs Institute. The extensive search strategy included electronic database searches in the Cochrane Library, PubMed, Embase, Web of Science, Google Scholar, MEDLINE, and CINAHL. The researchers rigorously screened the literature, extracted and analyzed the data, and evaluated the quality of the included studies using the Mixed Methods Appraisal Tool (MMAT).

**Results:**

Twenty-eight studies were included, of which 25 were quantitative studies, 2 were qualitative studies, and 1 was a mixed-methods study. A total of 1,697 subjects were investigated across all studies. Quantitative evidence supports the notion that elastic band resistance training can improve upper and lower limb flexibility, endurance, upper strength, physical balance, and cardiopulmonary function and enhance the mental health of elderly individuals. Participants in the qualitative study reported some preferences and obstacles with band resistance training, but most participants reported physical benefits.

**Conclusions:**

Despite the heterogeneity between studies, this review is the first systematic review to comprehensively evaluate the effectiveness of elastic band resistance training in older adults. It not only shows the influence of elastic band resistance training on the physical and mental health of the elderly, but also emphasizes the preference and obstacles of elderly individuals face.

## 1. Introduction

At present, the world is facing a state of accelerated population aging, with a significant increase in the number of elderly people [1–3]. It is estimated that between 2015 and 2050, the population of those over 60 years old will rise from 900 million to 2 billion [4]. With increasing age, the body’s physiological functions gradually decline, and the most obvious changes are the gradual decline in strength caused by muscle atrophy, the continuous increase in body fat content, and the loss of skeletal muscle volume [5–7], resulting in the occurrence of physical weakness, mobility decline, a decrease in quality of life, accidental falls, and other adverse events [8–10]. At the same time, due to the decline in physical fitness, loneliness, depression, and other psychological problems easily occur [11–13], which causes a burden of care and economic burden for patients, their families, and society [14–16].

Improving physical activity levels has become an important part of chronic disease management strategies in elderly individuals [17–19]. Regular physical exercise can improve physical function, enhance muscle mass and strength, improve quality of life, reduce depressive symptoms, relieve anxiety, and promote mental health [20–22]. In addition, physical exercise not only embodies a healthy lifestyle for the elderly but also represents an important measure for countries around the world to actively cope with the aging population [23–25].

Physical exercise includes aerobic and resistance exercise [26, 27]. Resistance training is more effective than aerobic exercise in enhancing muscle strength [28–30]. Resistance exercises, also known as strength training, include any activity that causes muscles to contract in order to increase muscle strength and functional ability [31]. One of the commonly used equipment for resistance training is elastic band, which is characterized by low cost, easy access, small size and safety [32].

To date, five reviews have evaluated the effects of elastic band resistance training in older adults. A review by Daryanti et al [31] examined the effects on frail elderly people. Martins et al [32] focused only on the effects of band resistance training on muscle strength parameters. Kim et al [33] mainly studied the influence of elastic band exercise on shoulder function in elderly individuals, but did not explore the influence of resistance training on mental health. Yeun [34] investigated the influence of resistance training with an elastic band on the flexibility and balance of elderly individuals in the community, and included an intervention of resistance training with an elastic band, plus other interventions. Lin et al [35] only included quantitative studies on the effects of elastic band exercise on the physiological function of elderly individuals and did not pay attention to the effects of resistance training on mental health.

The current literature does not fully synthesize what is known in this area and lacks a mixed-approach systematic review of the effects of band resistance training on physical and mental health in elderly individuals. This systematic review is different from previous ones in two aspects. First, it evaluates the influence of elastic band resistance training on the physical and mental health of elderly individuals, as well as their preferences and obstacles faced during training. Second, the results combine quantitative and qualitative evidence.The purpose of this study was to investigate the influence of elastic band resistance training on the physical and mental health of elderly individuals, as well as their preferences and obstacles in training.

## 2. Methods

The authors followed a convergent segregated approach recommended by the JBI methodology of mixed-methods systematic reviews (MMSR). In this approach, discrete quantitative and qualitative syntheses are conducted first; then, the evidence from both syntheses is combined [36, 37]. The protocol for this study was registered on the PROSPERO register of systematic reviews (CRD42023387711).

### 2.1. Selection criteria

#### 2.1.1. Inclusion criteria

The review included quantitative randomized controlled trials, quantitative non-randomized studies, quantitative descriptive analyses, qualitative studies, and mixed-methods studies that used elastic band resistance training as an intervention in older adults. The subjects of the study were elderly people ≥ 60 years old with normal communication and comprehension. The application environment of elastic band resistance training is unlimited, including hospitals, communities, homes, and nursing homes. The quantitative portion of the intervention for elastic band resistance training in older adults was also reviewed. Outcome measures were physical fitness (including upper and lower limb flexibility, upper and lower limb endurance, upper and lower limb strength, body balance, cardiopulmonary function) and mental health (including scales with mental health dimensions). The qualitative component of this review considered studies that investigated the experiences and perceptions of elastic band resistance training in older adults.

#### 2.1.2. Exclusion criteria

The exclusion criteria were (1) studies with patient populations less than 60 years of age, (2) studies in non‐English languages, (3) review articles and conference abstracts, and (4) exercise interventions and other interventions (such as nutrition interventions) combined.

### 2.2. Search strategy

The electronic databases of the Cochrane Library, PubMed, Embase, Web of Science, Google Scholar, MEDLINE, and CINAHL were searched to identify articles that met the inclusion criteria. The search period was from the establishment of the database to December 31, 2022. When searching the database, the retrieval method of combining subject words and free words was adopted. The following keywords were used in the database search: aged, aging, age-related, seniors, elderly, older, resistance band, elastic band, resistance rope, elastic rope, exercise, train, movement.

The first and second authors worked together to develop keywords and search strategies according to the requirements of each database. The first author independently reviewed the title and abstract of each study based on the inclusion and exclusion criteria. The first and second authors then read each full text independently and cross-checked it. Disagreements were resolved through discussion or through consultation with the third author. Meanwhile, the authors manually searched the references of the included studies to supplement unretrieved relevant literature.

### 2.3. Assessment of methodological quality

The first and second authors independently evaluated the quality of the included studies using the Mixed Methods Appraisal Tool (MMAT) [38]. After completing the evaluation, the results were cross-checked. If there was any disagreement, it was resolved through discussion. If there was still no decision, it was submitted to the third author for negotiation. There was 100% agreement between the first and second authors on the methodological quality entries of MMAT. The fourth author verified the methodological quality assessment.

### 2.4. Data extraction

The authors followed the JBI method of MMSR for data extraction [36, 37]. The extracted data included the author(s), year of publication, country, study methods, population, aim of the study, intervention delivery, outcome measures and key findings. The first author extracted the data independently, the second author checked the accuracy of the data extraction, and the third author randomly cross-checked the data to reduce the probability of data extraction errors. In case of disagreement, the full text was reread, and the results were discussed with the fourth author to resolve differences in the extracted data.

### 2.5. Data synthesis and integration

The authors followed the convergent segregated approach to synthesize and integrate the data based on the JBI methodology of MMSR [36, 37]. Although randomized controlled trials account for the majority of quantitative studies, meta-analyses cannot be conducted due to the heterogeneity of research interventions, results, and measurements. Therefore, quantitative results are reported in narrative form, and descriptive analysis is performed. The number of available qualitative studies was too few; therefore, a narrative synthesis was used to present the findings. Finally, we used a narrative synthesis approach to integrate the evidence from the quantitative and qualitative studies.

## 3. Results

**Fig 1:**
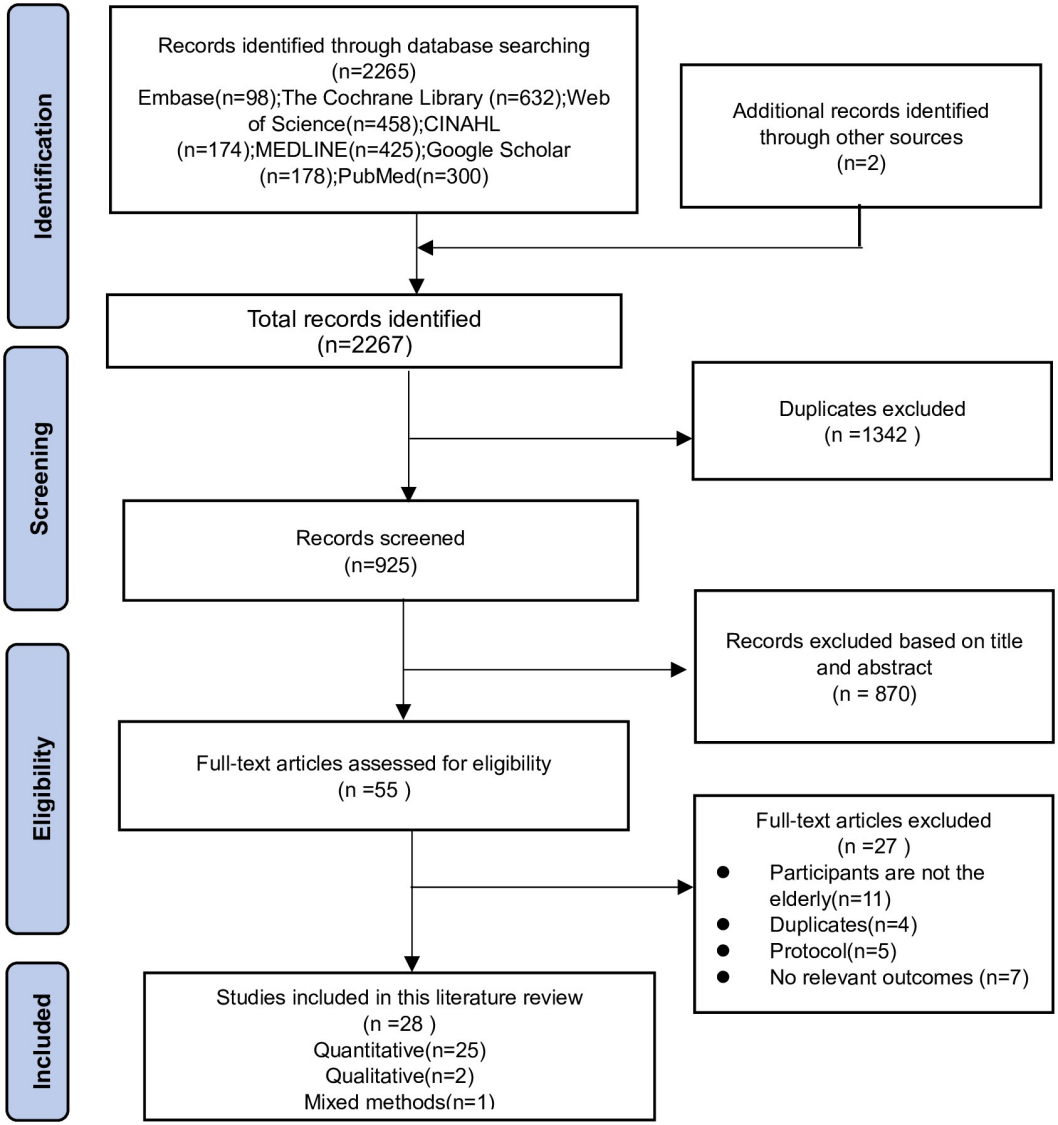
PRISMA flow diagram.

The PRISMA Flow Diagram presents the number of papers included throughout the selection process, alongside with the reasons for exclusion (Fig 1).

A total of 28 studies were considered to meet the inclusion and exclusion criteria in the review [39–66], of which 25 were quantitative studies [39–54, 58–66], 2 were qualitative studies [55, 56], and 1 was a mixed-methods study [57]. These studies were from nine countries: China (10), South Korea (6), Austria (2), the United States (3), Japan (1), Denmark (1), Spain (3), Serbia (1), and Iran (1).

### 3.1. Characteristics of the included studies

Table 1 presents the key characteristics of the included studies. Most were published between 2013 and 2022, and the majority were quantitative studies, including 7 quantitative non-randomized studies [42, 46, 48, 54, 58, 65, 66], 1 quantitative descriptive study [39], and 17 quantitative randomized controlled trials [40, 41, 43–45, 47, 49–53, 59–64]. The subjects were all 60 or older; six of the studies looked specifically at older women [41, 51, 52, 60, 64, 65], and one specifically at older men [44].

**Table 1:** Details of study characteristics.

Authors,country	Study design	Sample,setting	Aim of the study	Intervention	Data collection(measures)	Key findings
Park et al.(2015) [39], Korea	Quantitative Descriptive	46 elderly people (8 males, 38 females) aged over 65 in a rural area.	Examine the effects of a muscle strengthening exercise program using an elastic band on changes in the physical abilities and quality of life	A total of 16 80-minute sessions of resistance training were performed over an eight-week period.	Arm Curl,TCR test,2-Minute step in place test,One-leg standing-eyes open and closed,FRT,BST,TUG test,Tandem walk test、the Korean version of the World Health Organization Quality of Life (WHOQOL)-BREF questionnaire.	*After 8 weeks, muscle endurance, body balance and limb flexibility were improved (p<0.05). *The score in quality of life showed statistically significant improvements after training(p<0.05).
Chen et al. (2016) [40]a, China	RCT	Aged 65 years and over. EG:n = 59(stage I),n = 56(stage II) CG: n = 56(stage I),n = 51(stage II)	Test the feasibility and effects of 12 months Wheelchair-bound Senior Elastic Band (WSEB) group-exercises.	EG: The WSEB program was conducted three times per week and 40 min per session in two stages: volunteer-led for the first six months (stage I) followed by the DVD-guided modality for another six months (stage II). CG: Normal daily activities were maintained without any special intervention.	Lung capacity, body flexibility, range of joint motion, muscle strength and endurance,ADL measured by the Barthel Index; sleep quality measured by the Pittsburgh Sleep Quality Index.	After 6 months and 12 months of intervention, the results of all variables in the experimental group were better than those in the control group (p<0.05).
Lee et al.(2021) [41], China	RCT	EG: n = 15(70.13±4.41 years old) CG: n = 12(71.82±5.23years old)	Investigated the effects of elastic band resistance exercise on the physical capacity and body composition of elderly women with osteosarcopenic adiposity.	EG: Participants used elastic bands to perform resistance training for about 60 minutes three times a week for 12 weeks under the supervision of an advanced licensed physical therapist. CG: Attend health education seminar and get an elastic band so you may work out at home with brochures.	Body fat percentage,appendicular lean mass(ALM),lean muscle mass index(LMI),SMI (ALM/height), Bone density parameter(spine bone density, spine T value), FRT, HGS, one-leg standing test with eyes closed, 10-meter walking speed, TUG test, TCR test.	*After 12 weeks of intervention, bone mineral density and T-value of spine in the experimental group were significantly higher than baseline (p<0.05). *Compared with the control group, the 10-meter walking speed, the standing test and the number of sitting and standing from chair in 30 seconds were significantly improved (p<0.05). *Standing up walking test, functional stretching test, and 10-meter walking speed showed significant improvement compared with baseline after 3 months of intervention, and functional stretching test still showed significant improvement after 6 months of intervention (p<0.05).
Fahlman et al.(2011) [42], US	Quasi-experimental trial	Older adults living independently, with some functional limitations, currently do not exercise CG: n = 41(75.6±1.3 years old, BMI = 29.5±4.2) EG: n = 46(74.8±1.0years, BMI = 30.5±3.2)	Determine the effects of 16 weeks of strength training on measures of functional ability in elderly who are functionally limited.	EG: Perform 13 different strength training exercises three times a week for 16 weeks using Thera-Band resistive bands. CG: Normal activities.	Time required to go up and down 27.7 inches of steps, Arm Curl, TCR test, gait characteristics, and isometric knee and arm flexion and extension.	*The exercise group demonstrated significant improvements in upper-body strength as measured by biceps curl (p < 0.05) and lower-body strength as measured by chair sit-to-stand (p < 0.05). Gait velocity (p < 0.05) and step length (p < 0.05) both increased for the exercise group at week 9. *The exercise group demonstrated significant improvements in upper-body strength as measured by biceps curl and lower-body strength as measured by chair sit-to-stand(p<0.05).
Franzke et al. (2015) [43]a, Austrian	RCT	Cognitive Training(CT): n = 33(83.5±5.4years old) Resistance Training(RT): n = 35(82.8±5.7years old) RT+nutritional supplement(RTS): n = 29(82.5±7.5years old)	To investigate the effect of 6 months resistance training on chromosome damage in elderly Austrian patients.	Total 6 months. RT: Received resistance training twice a week, supervised by an exercise scientist who recorded attendance. The intensity and volume of exercise was gradually increased starting from the fifth week. RTS: The same training as the RT group was carried out. In addition, fluid supplements were taken every morning and directly after each training session. CT: Performed coordination or cognitive tasks twice a week with seated memory training and finger dexterity training.	The cytokinesis-block micronucleus (CBMN),TCR test,HGS,6MWT,vitamin B12 and folic acid in red blood cells.	*For CBMN, there was no significant difference between the three groups (p>0.05). *Participants in the RT and RTS groups showed significant improvements in the chair sit-to-stand test and the 6-minute walk test (p<0.05). *The CT group showed no improvement in functional parameters (p>0.05) and all three groups showed no significant improvement in grip strength test (p<0.05). *After six months, only the RTS group showed a significant change in vitamin B12(p<0.05).
Park(2016) [44], Korea	RCT	Thirty healthy elderly men (age ≥65 years) CG: n = 15 EG: n = 15	To examine the effect of resistance exercise on carotid intima-media thickness, luminal diameter, peak systolic flow velocity, end diastolic flow velocity, and wall shear rate in healthy elderly men.	EG: The 24-week exercise intervention consisted of 3 days of resistance exercise per week using an elastic band per week.	Body fat percent, skeletal muscle mass, systolic blood pressure,HGS, TCR test, SRT,, maximum walking speed, time up and go, 2-Minute step in place test, peak systolic flow velocity, end diastolic flow velocity, and wall shear rate	Body fat percent, skeletal muscle mass, systolic blood pressure, grip strength, arm curl, chair stand up, sit and reach, maximum walking speed, time up and go, and two-minute step test showed significant interaction. Peak systolic flow velocity, end diastolic flow velocity, and wall shear rate also showed significant interaction(p<0.05).
Cancela et al.(2017) [45], Spanish	RCT	A group:n = 13(89.83±5.29years old) B group:n = 12(84.92±3.40 years old) C group:n = 11(89.00±5.43years old)	Compare the effects of three chair-based exercise programs on people older than 80 years.	Participants in group A participated in an aerobic exercise program. Participants in Group B participated in a band resistance training program. Participants in Group C participated in a joint mobility program. The participants exercised 3 days per week during 3 months.	The Tinetti Gait Balance, the Barthel Index, HGS, TUG test (Sit to stand,Gait to go,Turning,Gait return,Stand to sit).	*Participants in Group A experienced significant improvements in the portion of time required for gait-to-target and gait-return during the get-up-and-walk trial (p<0.05). *Participants in group B experienced a stable significant improvement in grip strength and balance levels and a significant decrease in time in the get-up-and-walk trial (p<0.05). *Part of the time (Sit to stand, Turning and Stand to sit) increased significantly (p<0.05) for participants in group C in the Stand to walk trial. *After 12 weeks of training, the RE group showed significantly greater improvement in arm curl compared to the NW and CW groups (p<0.05); in terms of lower body strength, the RE group improved more than the CW group in the chair stand test (p<0.05), with no difference in improvement compared to NW (p>0.05). *Improvement in upper and lower body flexibility was observed in all exercise groups compared to the control group (p<0.05).
Takeshima et al.(2013) [46], Japan	Quantitative nonrandomized studies	Nordic walking(NW):n = 17(70±5years old,8male and 9female) Conventional walking(CW):n = 16(68±5years old,8male and 8 female) Resistance exercise(RE):n = 15(68±5years old, 5 males and 10 females) CG:n = 17(70±7years old, 7males and 10 females)	To compare the effects of Nordic walking with conventional walking and band-based resistance exercise on functional fitness, static balance and dynamic balance in older adults.	NW: The NW group performed supervised exercise sessions three times a week for 12 weeks, 50–70 min per day (warm-up 10–15 min, main exercise 30 min in the first eight weeks and 40 min in the final four weeks, cool down 10–15 min). CW: They performed supervised exercise sessions three times a week for 12 weeks for a total of 50–70 min each day. RE:The RE group performed elastic resistance band-based exercises for all major muscle groups.	Arm Curl,TCR test, 12-Minute walk,TUG test,FRT,BST,sit and reach.	*the NW group had greater improvements in arm curl compared to the CW group. For lower body strength, RES (21.1%) improved more than CW (9.5%) in the chair stand and improvements for RES and NW (12.6%) did not differ.(p<0.05). *Upper (back scratch) and lower (sit & reach) body flexibility improved in all exercise groups compared to the control group (p<0.05).
Chen et al.(2019) [47], China	RCT	EG:n = 33(76.97±5.19 years old,12males and 21females) CG:n = 33(75.27±5.98years old, 11males and 22 females)	To discuss the effects of elastic band exercise on the frailty states in pre-frail elderly people.	CG: No exercise was applied to the control group. EG:Elastic band exercise was applied to elastic band group, 45–60 min per time for 8 weeks by 3 days a week.	The Fried frailty phenotype, HGS walking speed, physical activity.	The elastic band group showed significant improvements in the frailty states, grip strength (female) and walking speed both after 4-week and 8-week intervention (p<0.001), and significant improvements in grip strength (male) and physical activity after 8-week intervention (p<0.05). Within-group analysis (pre-intervention vs. after 4-week, after 4-week vs after 8-week, pre-intervention vs after 8-week) showed significant improvements (p<0.001) in grip strength (female/male) and walking speed in the elastic band group over time, while no significant differences in the control group (p>0.05).
Ponce-Bravo et al.(2015) [48], Spain	Quantitative nonrandomized	EG:n = 22 CG:n = 32	Examines the impact of a resistance-band functional exercise program, compared with a recreational exercise program, on physical fitness and reaction times in persons older than 60 years.	Participants in each group completed a 4-week training program consisting of 5 weekly sessions of 50 min each. EG:Functional training with resistance elastic-bands. CG:Recreational training.	Arm Curl, TCR test, TUG test, HGS, simple reaction and choice tests, BMI, Waist-to-hip ratio(WH-r), Expected aerobic performance(EAP).	*Intragroup comparisons revealed increases in leg and arm strength and improved aerobic performance in both groups (p < 0.05). *The CG participants also showed a slight reduction in BWI (p < 0.05). *In EG, handgrip strength and gross motor abilities also improved (p < 0.01). *Compared to CG, the EG showed greater improvements in response to training in handgrip strength, arm strength, and gross motor abilities. On average, functional training led to an 11% improvement in cognitive performance over recreational training.
Oesen et al.(2015) [49]a, Austrian	RCT	One hundred and seventeen older adults. cognitive training group(CT):n = 40 resistance exercise training(RT):n = 41 RT in combination with nutrient supplementation(RTS): n = 36	To evaluate the effects of elastic band resistance training in combination with nutrient supplementation on muscular strength and the ability to perform mobility-related activities of daily living in older adults living in retirement care facilities.	A 6-month intervention period. RT:Participants received resistance training twice a week and were supervised by an exercise scientist who recorded attendance. The intensity and volume of exercise was gradually increased from the fifth week onwards. RTS: Participants performed the same training as the RT group. In addition, fluid supplements were taken every morning and directly after each training session. CT: Participants performed a coordination or cognitive task twice a week, performing seated memory training and finger dexterity training.	Isokinetic torque measurements of the knee extensors and flexors in concentric mode at 60 and 120°/s, HGS, TCR test, Arm Curl, 6MWT, FRT, physical activity.	A repeated-measures ANOVA analysis revealed significant improvements in physical function of lower (p = 0.002) and upper extremities (p = 0.006) for RT and/or RTS in comparison to CT. For isokinetic measurements, 6 MWT, and gait speed time effects (p<0.05) were detected without any group × time interaction effects. Dropouts showed lower performance in chair stand test (p = 0.012), 6 MWT (p = 0.003), and gait speed (p = 0.013) at baseline than that of the finishers of the study.
Kwak et al.(2016) [50], Korea	RCT	EG: n = 15(80.1±4.7years old, 10females and 5 males) CG: n = 15(77.4±5.5years old, 9femals and 6 males)	To analyze the effects of elastic-band resistance exercise on balance, gait function, flexibility and fall efficacy in the elderly people of rural community.	CG: Both experimental group and control group underwent typical physical therapy 60 minutes per time, 3 times a week for 8 weeks. EG: The experimental group conducted elastic band resistance exercise in 1 time 3 sets, 30 minutes per time, 3 times a week for 8 weeks.	FRT, Berg’s balance scale, Dynamic Gait Index, TUG test, SRT, ABC scale.	*Measurements before and after intervention were significantly different between the two groups except for FRT and Berg’s balance scale (p<0.05). *After intervention, there were differences between the experimental group and the control group (p<0.05).
Damush et al. (1999) [51],US	RCT	N = 62(68±5.58years old) EG: n = 33 CG: n = 29	The short-term effects of an accessible exercise intervention on the strength and health-related quality of life (HRQOL) among older adult women were evaluated.	EG: Participants did strength training twice a week for eight weeks, lasting about 45 minutes each time. CG: There was no exercise intervention of any kind during this period.	Health-Related Quality of Life (HRQOL), HGS, latissimus dorsi, pectoral and quadriceps muscle strength.	*Quadriceps muscle and pectoral muscle strength were significantly enhanced (P<0.01); There was no significant change in grip strength. *After 8 weeks of intervention, changes in mental and physical health function in the experimental group were not significantly different from those in the control group.
Stojanović et al.(2021) [52], Serbia	RCT	EG: n = 86(75.7±8.9 years old) CG: n = 82(74.5±8.2 years old)	To examine the effects of 12 weeks of chair-based, low-load resistance training with elastic band (EBT) on functional fitness and metabolic biomarkers in older women.	EG: periodized chair-based, low-load whole-body resistance exercises (2 sets, 12–15 repetitions, 40–60% of one repetition maximum-1RM) using an elastic band, twice weekly for 12 weeks. CG: exclusively institution’s activities including chess, dice, reading, crafts etc.	*TCR test, Arm Curl, 2-minutes step in place test, sit and reach, BST, TUG test, HGS. * Measurement of serum glucose (GLU), total cholesterol (TC), high-density lipoprotein (HDL), low-density lipoprotein (LDL) and triglyceride(TG) levels.	*There were significant changes for GLU, TC, HDL and LDL pre vs post in experimental group as well as blood glucose in control group (p < 0.05). *Significant changes occurred before and after the 30-second chair rise tests, 30-second arm curl Tests, and 2-minute step in place test in both groups, while significant changes occurred only in the experimental group in the sit and reach, BST, TUG test, and HGS (p<0.05).
Chen et al.(2015) [53]a, China	RCT	Aged 65 and over EG: n = 59 CG: n = 55	To test the effectiveness of six-month Wheelchair-bound Senior Elastic Band exercises on the functional fitness of older adults in nursing homes.	EG: A 40-min Wheelchair-bound Senior Elastic Band exercise program was implemented three times per week for six months. CG: The participants followed their daily activities, which did not include any type of elastic band exercise.	Barthel Index rating scale, lung capacity, BST,sit and reach, Dominant lateral shoulder flexion and abduction angles, HGS, TCR test, Arm Curl.	*Compared with baseline, after 6 months, all functional health indicators except ADL in the experimental group were significantly improved (p<0.05), while ADL, lunge capacity and lower limb flexibility in the control group were significantly improved (p<0.05). *After 3 and 6 months of intervention, all the test indicators of the experimental group were better than those of the control group (p<0.05).
Yang et al.(2015) [54], China	Quasi-experimental design	EG: n = 84 CG: n = 85	The transtheoretical model was applied to promote behavioural change and test the effects of a group senior elastic band exercise programme on the functional fitness of community older adults in the contemplation and preparation stages of behavioural change.	EG: Participants attended a 40-minute senior stretch band exercise program three times a week for six months. CG: Participants did not receive any intervention and maintained their daily activities.	Exercise status questionnaire, lung capacity, 2-minute step in place test, BST, sit and reach, HGS, TCR test.	*The results after 6 months of intervention in the experimental group were better than those before and after 3 months of intervention (p<0.05). At the start of the study, 97 participants in the experimental group were in the intent or readiness phase across the theoretical model, and 84 participants moved into the action phase six months after the intervention.
Chen et al.(2013) [55], China	Qualitative	n = 10(82.10±5.86years old)	Assess the feasibility of an elastic band exercise program for older adults in wheelchairs.	A feasibility appraisal survey was administered to 10 older adults in wheelchairs through individual interviews after 4 weeks of the WSEB program.	Participants were interviewed through a semi-structured questionnaire to evaluate the simplicity, safety, appropriateness and helpfulness of the training and to make recommendations.	*Participants felt more strength in their hands and leg muscles, increased flexibility and range of motion in their bodies, more energy, and willing to continue to participate in elastic band exercises. *Most participants tended to participate in 40 minutes of training 3 times a week.
Chen et al.(2013) [56], China	Qualitative	In phase I,11 professional experts In phase II, 20 participants 65 years or older	To develop a tailored elastic band exercise program for aged adults (persons 65 years and older), to evaluate the feasibility of a program, and to determine appropriate elastic band exercise frequencies and preferences of aged adults.	In phase I, 11 professional experts were consulted to develop the Senior Elastic Band (SEB) exercise program. They responded to detailed description and demonstrations of the program contained on either a hard copy or a DVD. In phase II, 20 participants 65 years or older were interviewed for their feedback on the SEB after participating in 1 month of instructor-led SEB group practice.	In phase I, The experts rated each exercise movement and made suggestions for modification. In phase II, participants were interviewed through a semi-structured questionnaire to evaluate the simplicity, safety, appropriateness and helpfulness of the training and to make recommendations.	Both the experts in phase I and the senior participants in phase II rated the SEB highly and commented that the program was feasible, safe, suitable, and helpful. The participants further suggested practicing SEB 3 times per week for 60 minutes per session in a group of 20 to 29 people.
Rathleff et al.(2017) [57],Denmark	Mixed methods studies	Elderly inpatient: n = 15 Ward staff: n = 4	To investigate feasibility and acceptability of an unsupervised progressive strength training intervention monitored by BandCizer for frail geriatric inpatients.	At hospitalization, the patients were prescribed two elastic band exercises to be performed unsupervised once daily. The patients were instructed in performing strength training: 3 sets of 10 repetitions (10–12 repetition maximum (RM)) with a separation of 2-min pauses and a time-under-tension of 8 s. The feasibility criterion for the unsupervised progressive exercises was that 33% of the recommended number of sets would be performed by at least 30% of patients.	*The following data were collected from the BandCizer: number of sets, number of repetitions, average time-under-tension, and total time-under-tension. *patients and staff were interviewed about their experiences with the intervention.	*The results of objective monitoring of training implementation indicate that the intervention in its current form is not feasible. *Generally positive attitude of patients and staff towards unsupervised training.
Chan et al.(2016) [58], China	Quasi-experimental trial	n = 20(72.00±5.12 years old, 2male 18female)	Assess the effectiveness of a newly developed Senior Elastic Band (SEB) exercise program on the health of older adults in community care stations.	The SEB intervention included three phases (warm-up, aerobic motion, and static stretching) and was conducted three times per week, 40 minutes per session for 1 month.	Blood pressure, lung capacity, 2-minute step in place test, BST, sit and reach, HGS, TCR test, Standing on one leg with eyes closed, SF-12, PSQI.	*Participants showed improved performance at the end of the 1-month study for the following indicators: lung capacity, cardiopulmonary fitness, upper and lower body flexibilities, upper limb muscle power, lower limb muscle endurance, and self-perceived physical health status (all p< 0.05).
Chen et al.(2020) [59], China	RCT	EG: n = 30(65.9±2.9years old, 11male19female) CG: n = 30(65.0±3.1years old, 18male 12female)	To investigate the effects of two resistance exercise approaches on glycated hemoglobin (HbA1c) level and function performance.	EG: A total of 12 weeks, three times per week, with the first two weeks of training using elastic bands under the guidance of clinical staff, followed by at-home training and communication of exercise through ongoing phone calls. CG: Except for the use of elastic bands, the training movements were the same as the experimental group.	Glycated hemoglobin, TCR test, TUG test, WOMAC scale.	*Training had no statistically significant effect on glycated hemoglobin (p>0.05). *The standing walking test score, 30-second sit-to-stand count, and overall WOMAC scale score were all higher than baseline; all gains were greater in the experimental group than in the control group (p<0.05).
Lee et al.(2015) [60], Korea	RCT	EG:n = 10(74±4.6years old) CG:n = 10(73±6.4years old)	To investigate the effect of an exercise intervention on the balance ability and muscle function of elderly women.	EG:The subjects participated in an elastic band exercise program lasting for 8 weeks, exercising for 40 minutes, four days a week with resting terms of 60 sec. CG:Did not receive any type of exercise intervention.	TCR test, 2-minute step in place test, Standing on one leg with the eyes open.	the number of repetitions of sit-to-stand in 30s, the number of knee raises in 2 minutes and the time standing on one leg with the eyes open significantly increased in the exercise group after the exercise intervention (p<0.05).
Yu et al.(2013) [61], Korea	RCT	EG: n = 12(65.5±4.5years old, 8male 4 female) CG: n = 12(65.0±3.4years old, 6male 6 female)	To investigate the effects of resistance exercise using Thera-band on balance of elderly adults.	The experimental group performed stretching and resistance exercises, and the control group performed stretching exercises only three times a week for five weeks.	Berg’s balance scale, TUG test, and Standing on one leg with eyes open and closed.	*The values of the Tetrax in the weight distribution index with eyes open and that with eyes closed and the stability test index with eyes open were significantly lower in the resistance exercise group than in the control group. *The pre-test values were was significantly higher than the post-test values. *There were no significant differences between groups in the values of the Berg Balance Scale, the Timed Up & Go Test, and the Tetrax stability test index with the eyes closed.
Jette et al.(1996) [62], US	RCT	EG: n = 42(71.0±4.3years old, 54.8%female) CG: n = 51(73.2±5.4years old, 70.6%female)	Describes a videotaped, home-based, strength training program, titled Strong-for-Life and report on its effectiveness in improving muscle strength, psychological well-being, and health status.	EG: Physical therapists instruct participants in an exercise program. The participants then followed a 30-minute video at home and exercised with elastic bands three times a week for 12–15 weeks. CG: Received the same training as the experimental group after the study.	Peak torque of right lower limb knee and right upper limb shoulder extensor and flexor, POMS, SF-36.	*Compared with the control group, the knee flexion and extension torque of the experimental group increased after intervention (p<0.05). *After intervention, the sense of anger and tension in the experimental group was significantly reduced (p<0.05), and the energy was also significantly increased (p<0.05). *Compared with the control group, the social function of the elderly in the intervention group increased significantly (p = 0.04).
Su et al.(2022) [63], China	RCT	A total of 61 participants (79.25±8.19years old, 49.2%male, 50.8%female) EG:n = 31 CG:n = 30	To study the effects of 3-month elastic band exercise program on daily living activities, hand muscle strength, balance and lower limb muscle strength in the elderly.	EG:Participants took part in 40 minutes of elastic band training three times a week for three months. CG:Participants participated in a regular exercise program provided by the nursing facility.	Blood pressure, BMI, Barthel Index rating scale, HGS, TCR test, Berg’s balance scale.	* In the experimental group, ADL, grip strength and balance improved significantly after 3 months of intervention (p<0.05). * After intervention, the experimental group performed better than the control group in ADL, grip strength, balance, and 30-second chair rise tests (p<0.05).
Kim et al.(2022) [64], Korea	RCT	EG: n = 15(81.6±4.78 years old, Body fat percentage:35.59±4.22%) CG: n = 15(79.6±5.19 years old, Body fat percentage:36.88±2.50%)	To investigate the effects of a 24-week resistance training program on the body of obese elderly women.	Participants in the experimental group trained for 60 minutes twice a week for 24 weeks. The program includes 10 minutes of warm-up, 40 minutes of resistance training with elastic bands and 10 minutes of static stretching.	Fat mass, fat free mass, body fat percentage, bone density, HGS, sit and reach, BST, Arm Curl, TCR test, TUG test, 2-minute step in place test.	* After intervention, there was a significant difference in body fat percentage between the experimental group and the control group (p<0.05). * After 24 weeks, grip strength and lower body strength were significantly increased in the experimental group (p <0.01). * After intervention, the experimental group outperformed the control group in grip strength, 30-second chair rise tests, and sitting and stretching tests (p<0.05).
Azamian et al.(2022) [65], Iran	Quasi-experimental trial	EG:n = 14(73.29±5.44 years old) CG:n = 14(74.79±3.87 years old)	To explore whether resistance training with elastic bands can increase serum adropin levels and improve cardiometabolic status in elderly women.	EG: A 55-minute stretch band resistance training program was performed three times a week for 12 weeks under the supervision of two qualified exercise scientists. CG:They were asked to maintain normal physical activity during the study.	Arm Curl, TCR test, BMI, waist-hip ratio(WHR), Body fat percentage, Serum levels of insulin, glucose, total cholesterol, HDL cholesterol, LDL, triglycerides, and hypersensitive C-reactive protein.	*After intervention, serum adropin level increased by 40.71% in the experimental group (p = 0.001), while no significant change was found in the control group. *After intervention, serum levels of tumor necrosis factor-α (TNF-α) and hypersensitive C-reactive protein (hsCRP) in the experimental group were significantly lower than those in the control group (p<0.05). *After intervention, insulin resistance index (HOMA-IR) was significantly improved in the experimental group compared with the control group (p&lt; 0.05). *After 12 weeks of exercise intervention, the body fat percentage of the experimental group was significantly lower than that of the control group (p = 0.023). *Significant improvement in 30-second chair rise tests and 30-second arm curl Tests in the experimental group compared to baseline and control groups (p> 0.05).
Sanchez-Lastra et al.(2022) [66],Spain	Quantitative nonrandomized studies	EG1:n = 20(87.6±6.4 years old) EG2:n = 29(81.4±7.7 years old) CG:n = 19(81.3±9.5years old)	To compare the effects of upper and lower body resistance exercise on cognition and physical function in hospitalized elderly.	EG1:The first phase began with a three-month resistance training program focused on the upper body. In the second phase, a three-month resistance training program focused on the lower body. EG2:The first phase began with a three-month resistance training program focused on the lower body. In the second phase, a three-month resistance training program focused on the upper body. CG:The same flexibility training program was performed in both sessions for the same amount of time as in the experimental group.	The spanish version of the mini-mental state examination, trail making test part A, fototest, TUG test, sit and reach, BST, HGS.	*After the first phase, cognitive function in both groups improved significantly, as did grip strength in experimental group 2 (p<0.05). *Lower limb and shoulder flexibility improved in all groups after phase 2. *Upper body movement was more effective for cognitive function, lower body movement had a better effect on body function parameters.

^a^This symbol means that multiple papers have been published in the same sample of participants.

Abbreviations:CG, control group; EG, experience group; RCT, randomized controlled trial; HGS, Hand grip strength test; PSQI, Pittsburgh Sleep Quality Index; SF-12, 12-Item Short-Form Health Survey; TUG test, Time Up and Go Test; ABC scale, Activities-specific Balance Confidence scale; SRT,sit and reach test;6MWT, Six minute walking test; FRT, Functional Reach Test; BST, Back Scratch Test; TCR test,Timed chair rise test;sit and reach, Chair Sit and Reach Test; Arm Curl, 30-s Arm Curl Test ; SF-36, the short form 36 health survey questionnaire; POMS, Profile of Mood States

All studies involved elastic band resistance training in the elderly population. The duration of training is between one and 12 months, two to five times a week, for 30 to 80 minutes each time. The main outcomes measured in the quantitative studies were body balance, upper and lower limb muscle strength, endurance and flexibility, and mental health status. The qualitative study used semis-structured face-to-face interviews to focus on the experience and suggestions of the elderly on elastic band resistance training.

### 3.2. Quality evaluation of the included studies

All included studies were scored according to the MMAT. The specific evaluation results are shown in Table 2.

**Table 2:** Quality assessment results according to mixed methods appraisal tool.

**Category of study design: Qualitative**	**Screening question responses**		**Methodological quality criteria responses**				
	**S1**	**S2**	**1.1**	**1.2**	**1.3**	**1.4**	**1.5**
Chen et al.(2013) [55]	Yes	Yes	Yes	Yes	Yes	Yes	Yes
Chen et al.(2013) [56]	Yes	Yes	Yes	Yes	Yes	Yes	Yes
**Category of study design:Quantitative randomized controlled trials**	**Screening question responses**		**Methodological quality criteria responses**				
	**S1**	**S2**	**2.1**	**2.2**	**2.3**	**2.4**	**2.5**
Chen et al.(2016) [40]a	Yes	Yes	Yes	Yes	Yes	No	Yes
Lee et al.(2021) [41]	Yes	Yes	Yes	Yes	Yes	Yes	No
Franzke et al.(2015) [43]a	Yes	Yes	Can’t tell	Yes	Yes	Yes	Yes
Park(2016) [44]	Yes	Yes	Can’t tell	Yes	Yes	Can’t tell	Yes
Cancela et al.(2017) [45]	Yes	Yes	Yes	Yes	Yes	Can’t tell	Yes
Chen et al.(2019) [47]	Yes	Yes	Yes	Yes	Yes	Yes	Yes
Oesen et al.(2015) [49]a	Yes	Yes	Can’t tell	Yes	Yes	Can’t tell	Yes
Kwak et al.(2016) [50]	Yes	Yes	Can’t tell	Yes	Yes	Can’t tell	Yes
Damush et al.(1999) [51]	Yes	Yes	Can’t tell	Yes	Yes	Can’t tell	Yes
Stojanović et al.(2021) [52]	Yes	Yes	Can’t tell	No	Yes	Yes	Yes
Chen et al.(2015) [53]a	Yes	Yes	Yes	Yes	Yes	No	Yes
Chen et al.(2020) [59]	Yes	Yes	Yes	Yes	Yes	Can’t tell	Yes
Lee et al.(2015) [60]	Yes	Yes	Can’t tell	Yes	Yes	Can’t tell	Yes
Yu et al.(2013) [61]	Yes	Yes	Can’t tell	Yes	Yes	Can’t tell	Yes
Jette et al.(1996) [62]	Yes	Yes	Can’t tell	Yes	Yes	Yes	Yes
Su et al.(2022) [63]	Yes	Yes	Yes	Yes	Yes	Can’t tell	Yes
Kim et al.(2022) [64]	Yes	Yes	Can’t tell	Yes	Yes	Can’t tell	Yes
**Category of study design: Quantitative nonrandomized studies**	**Screening question responses**		**Methodological quality criteria responses**				
	**S1**	**S2**	**3.1**	**3.2**	**3.3**	**3.4**	**3.5**
Fahlman et al.(2011) [42]	Yes	Yes	Yes	Yes	Yes	No	Yes
Takeshima et al.(2013) [46]	Yes	Yes	Yes	Yes	Yes	Yes	Yes
Ponce-Bravo et al.(2015) [48]	Yes	Yes	Yes	Yes	Yes	No	Yes
Yang et al.(2015) [54]	Yes	Yes	Yes	Yes	Yes	Yes	Yes
Chan et al.(2016) [58]	Yes	Yes	Yes	Yes	Yes	No	Yes
Azamian et al.(2022) [65]	Yes	Yes	Yes	Yes	Yes	Yes	Yes
Sanchez-Lastra et al.(2022) [66]	Yes	Yes	Yes	Yes	Yes	No	Yes
**Category of study design: Quantitative Descriptive**	**Screening question responses**		**Methodological quality criteria responses**				
	**S1**	**S2**	**4.1**	**4.2**	**4.3**	**4.4**	**4.5**
Park et al.(2015) [39]	Yes	Yes	No	Yes	Yes	Yes	Yes
**Category of study design: Mixed methods studies**	**Screening question responses**		**Methodological quality criteria responses**				
	**S1**	**S2**	**5.1**	**5.2**	**5.3**	**5.4**	5.5
Rathleff et al.(2017) [57]	Yes	Yes	Yes	Yes	Yes	Yes	Yes

### 3.3. Quantitative evidence

#### 3.3.1. Physical fitness

*3*.*3*.*1*.*1*. *Upper and lower limb flexibility*. Twelve of the 25 quantitative studies measured the effects of elastic band resistance training on upper and lower limb flexibility in older adults. Six were randomized controlled trials [40, 44, 50, 52, 53, 64], five were non-randomized controlled trials [46, 48, 54, 58, 66], and one was a quantitative descriptive study [39].

Nine studies used back scratch tests [39, 40, 46, 52–54, 58, 64, 66], and one used simple reaction and choice tests to assess upper limb flexibility [48]. Of the ten studies, statistically significant improvement in upper limb flexibility after intervention was noted in seven studies (p<0.05) [40, 46, 48, 52–54, 58], and a non-significant improvement was noted in three studies [39, 64, 66]. Of the studies measuring lower limb flexibility, eight used the chair sit and reach test [39, 40, 46, 52–54, 58, 64, 66], and two used the sit and reach test [44, 50]. All studies found statistically significant improvement in lower limb flexibility after intervention (p<0.05). The above evidence suggests that elastic band resistance training could improve upper and lower limb flexibility in elderly individuals.

*3*.*3*.*1*.*2*. *Upper and lower limb endurance*. A total of 18 of 25 quantitative studies evaluated the effect of elastic band resistance training on upper and lower limb endurance in older adults. Eleven studies were randomized controlled trials [40, 41, 43, 44, 49, 52, 53, 59, 60, 63, 64], six were non-randomized controlled trials [42, 46, 48, 54, 58, 65], and one was a quantitative descriptive study [39]. Sixteen of the studies assessed the effect of elastic band resistance training on upper and lower limb endurance in older adults using the 30-s arm curl test and the 30-s chair rise test [39–44, 46, 48, 49, 52, 53, 59, 60, 63–65], and two studies used the 60-s chair rise test [54, 58]. Of the 10 studies that used the arm curl test, only one did not observe a significant increase in arm curling after the intervention [64]. In 18 studies that assessed lower limb endurance, there was a significant rise in the number of times older adults moved from sitting to standing after the intervention (p<0.05). In two of the studies [48, 52], the control group also showed significant improvement in upper and lower limb endurance after the intervention. The above results indicate that elastic band resistance training helped to improve upper and lower limb endurance in elderly individuals.

*3*.*3*.*1*.*3*. *Upper limb strength*. Grip strength mainly measures the development level of upper limb muscle groups. Among the 25 quantitative studies included, 16 measured the effect of elastic band resistance training on hand grip strength in elderly individuals. Four were non-randomized controlled trials [48, 54, 58, 66], and the rest were randomized controlled trials [40, 41, 43–45, 47, 49, 51–53, 63, 64]. Hand grip strength was significantly enhanced after intervention in 12 studies (p<0.05) [40, 44, 45, 47, 48, 52–54, 58, 63, 64, 66], and no significant change was found in 4 studies [41, 43, 49, 51]. Overall, elastic band resistance training may have improved upper limb muscle strength in older adults.

*3*.*3*.*1*.*4*. *Body balance*. Seventeen of the 25 quantitative studies assessed the effect of elastic band resistance exercises on balance in older adults with varying assessment tools. Five of the studies used the functional reach test [39, 41, 46, 49, 50], which assesses balance function by measuring subjects’ ability to extend their elbows forward. Four studies showed a significant improvement after the intervention (p<0.05) [39, 41, 46, 50], but no significant change in the study of Oesen et al [49]. Three studies used Berg’s balance scale [50, 61, 63], two studies had significant changes after the intervention [50, 63], and one had no significant changes [61].

Five studies used the single-leg standing test with eyes open and/or closed, two studies showed no significant change in standing time after the intervention [41, 58], and three studies showed a significant increase (p<0.05) [39, 60, 61]. Twelve studies used the up-and-go test [39, 41, 44–46, 48, 50, 52, 59, 61, 64, 66], which is used to assess balance and functional exercises and to predict fall risk in older adults, and all observed a significant reduction in measurement time after intervention except in the work of Park [39], Yu [61], Kim [64], and Sansanz-Lastra et al [66]. Other studies using the Tinetti Gait and Balance test [45], the Activities-specific Balance Confidence scale [49], the Dynamic Gait Index [50], and the time required to measure a 27.7-inch step up and down [42] to assess participants’ balance improved significantly after the intervention (p<0.05), in which the control group in Fahlman et al [42] also improved their balance (p<0.05). In general, elastic band resistance training could improve the balance of elderly individuals.

*3*.*3*.*1*.*5*. *Cardiopulmonary function*. Eleven quantitative studies evaluated the effects of elastic band resistance training on cardiopulmonary function in older adults using three main assessment tools [39, 40, 43, 44, 49, 52–54, 58, 61, 64]. Of these, four studies measured vital capacity [40, 53, 54, 58], six used the 2-minute step in place test [39, 44, 52, 54, 58, 61, 64], and two used the 6-minute walking test [43, 49]. Except for Kim et al ’s study [64], the experimental group was superior to the control group after intervention, and the difference was statistically significant (p<0.05), indicating that elastic band training could improve cardiopulmonary function in elderly individuals.

#### 3.3.2. Mental health

Five of the 25 quantitative studies evaluated the effects of elastic band resistance training on mental health in older adults [39, 51, 58, 62, 66]. Some of these studies did not directly use dedicated scales to assess mental health but employed scales containing dimensions of mental health [39, 51, 58].

Park et al [39] used the South Korean version of the World Health Organization’s Quality of Life questionnaire, which was divided into four main areas: physical health, psychological relations, social relations, and the surrounding environment. After the intervention, scores for psychological relations, social relations, and the surrounding environment showed statistically significant improvement. Damush et al [51] used the health-related quality of life (HRQOL) scale containing both mental and physical health functioning dimensions and observed no significant difference in changes in mental and physical health functioning compared to the control group. Chan et al [58] used the 12-Item Short-Form Health Survey, which measures participants’ perceived health, both physical and mental. In terms of mental health, there was no significant difference. Jette et al [62] used the Mood State Scale specifically to assess and understand the emotional state of participants, which was divided into six dimensions: tension-anxiety, depression-dejection, vigor, fatigue, anger, and confusion. Compared with the control group, the elderly men in the experimental group experienced significantly less anger, less tension-anxiety, and significantly more vigor after the intervention. In addition, the study also used the Short Form 36 Health Survey Questionnaire, which revealed no improvement in mental health. Sanchez-Lastra et al [66] used the Spanish version of the Mini-Mental State Examination and Trail Making Test Part A to assess participants’ mental state. The results showed that elastic band training can improve the cognitive function of elderly individuals.

Of the five studies on mental health, three interventions showed positive mental health benefits, and two interventions did not change, suggesting that the beneficial effects of elastic band resistance training on mental health in older adults are uncertain.

### 3.4. Qualitative evidence

Two qualitative studies and the qualitative portion of one study with a mixed approach explored the benefits, preferences and obstacles of elastic band resistance training in older adults.Two qualitative studies were conducted by Chen et al [55, 56]. The first study evaluated the feasibility of using a band resistance exercise regimen for older adults in wheelchairs [55]. After four weeks of elastic band resistance training, elderly people in wheelchairs were interviewed using semi-structured questionnaires to discuss the simplicity, safety, suitability, and helpfulness of the training and put forward suggestions. The second study by Chen et al [56] evaluated the feasibility of a band exercise program for older adults and determined appropriate exercise frequency and preferences for older adults. The study was divided into two stages. In the first stage, experts evaluated the simplicity, safety, suitability, and helpfulness of the program, scored each exercise, and put forward suggestions for modification. In the second stage, after a one-month group training with the elastic band for elderly individuals, participants were interviewed individually to evaluate the simplicity, safety, suitability, and helpfulness of the training and put forward suggestions. A mixed-methods study by Rathleff et al [57] investigated the feasibility and acceptability of unsupervised elastic band training for frail elderly inpatients. On day 1, the patient received 30 minutes of practice instruction, after which an unsupervised stretch band exercise was performed once daily, and the performance of the exercise was checked on days 2 and 4. Semi-structured interviews were held with patients and ward staff the day before discharge and after intervention.

Participants in all three studies reported physical and mental benefits of band resistance training [55–57]. After the intervention, participants felt that their muscles were more powerful in both their hands and legs, that they had increased body flexibility and joint range of motion, and they were more energetic and willing to continue participating in the stretch band exercises.

Participants also reported some preferences and barriers to training. In the first study by Chen et al [55], most participants tended to be placed in groups with 15 to 20 other participants, who engaged three times per week in 40 minutes of training. In the second study, most participants preferred to exercise in groups of 20 to 29 people for 1 hour 3 times a week. In addition, more than half of the participants preferred female coaches to lead them in the exercises [56]. Patients in the study of Rathleff et al [57] reported no difficulty in understanding and performing these exercises,the training time was unlimited, and the elastic band was easy to use and store. The motivation for training was mainly because they thought the exercises would help them return to their previous level of functioning. In addition to the physical benefits, the staff found that the exercises encouraged patients to take greater responsibility for their lives, and through active communication with the patients, they could learn which daily activities the patients needed help with and which ones they could perform independently. They also found that patients needed a certain level of cognitive ability to carry out the exercises independently. Statements about the amount of training indicated that patients did not truly care about the number of repetitions of the training movements but were more interested in whether they had been training at all times. If the training process was disturbed, the training amount was greatly affected.

### 3.5. Integration of quantitative and qualitative evidence

The quantitative and qualitative evidence was integrated based on five questions used to integrate evidence in the JBI methodology of MMSR [36, 37]. The independent syntheses of the quantitative and qualitative studies partially supported each other.

Evidence from integrated quantitative studies showed that elastic band resistance training helped to improve mental health, upper and lower limb flexibility, endurance, upper strength, and enhanced physical balance and cardiopulmonary function in older people, which has also been partially empirically verified in qualitative studies. Qualitative studies indicate that band resistance training could improve mental health, hand and leg muscle strength, and physical flexibility in older adults. However, there is no evidence from qualitative studies that training improved physical balance and cardiorespiratory function in older adults. One reason may be that relevant questions were not included in semi-structured interviews for qualitative research. Therefore, these two aspects should be discussed in future qualitative research on resistance training with an elastic band.

In the integrated quantitative study, the frequency of intervention was approximately 2–5 times per week, 30 to 80 minutes each time, which is consistent with the exercise preference of the elderly determined in the qualitative study (it was 40 to 60 minutes three times per week).

The number of exercise groups, repetitions, and mean and total muscle activation times were monitored in the study by Rathleff et al [57]. The results suggest that the current form of intervention was not feasible because few patients reached the standard training dose. However, qualitative evidence implies that patients and staff had a positive attitude toward the campaign. Future studies should adjust and refine interventions to improve patient adherence to exercise.

## 4. Discussion

### 4.1. Effects on physical fitness and mental health

This mixed-methods systematic review pooled evidence from the quantitative and qualitative studies on the effects of elastic band resistance training on physical and mental health in older adults. The quantitative studies support the idea that training interventions in the elastic band group are effective in improving upper and lower limb flexibility, upper and lower limb endurance, upper strength, balance, and cardiopulmonary function in elderly individuals. The results are similar to studies of other types of resistance training such as with soft weights, ankle weights, and medicine balls, which indicate that resistance training improved balance and physical activity by increasing muscle mass, muscle strength, and bone density [67, 68]. Other studies used resistance training to achieve lipid reduction, and to improve blood sugar, blood pressure, blood lipids, lung capacity, muscle flexibility, and joint mobility function [69, 70]. Data from qualitative and quantitative studies are combined using established methods to increase the richness and robustness of the synthesis. In addition, by changing the thickness and length of the elastic band, the level of resistance training could be increased or decreased flexibly, expanding the range of application of elastic band resistance training, such as in frail elderly people, obese elderly people, and elderly people in wheelchairs [40, 41, 47].

Of the five quantitative studies, three showed positive effects on the mental health of older adults, and two showed no difference. In the qualitative studies, participants had a positive attitude toward resistance training with elastic bands and reported feeling more energetic in their daily activities after the intervention. Combined with the results of qualitative research, elastic band resistance training was an effective, safe and economical measure that was beneficial to the mental health of elderly individuals. The reason might be related to the improvement of physical function or social interaction of the elderly through exercise [50]. Active exercise not only played an important role in reducing the onset of disease and increasing the independence of older people but also enhanced their self-esteem and body image, encouraged them to adopt a healthier lifestyle, and was an effective way of coping with stress. In addition, from a physiological point of view, the slow and gradual increase in the intensity of resistance training with elastic bands led to decreased sympathetic nerve output and increased happiness [51]. However, due to the small number of studies on the improvement of mental health by resistance training with elastic bands, these results should be interpreted with caution.

### 4.2. Training preferences and obstacles

The quantitative part of Rathleff et al [57] found that few patients reached the standard training dose with unsupervised training, while the qualitative part found that patients did not truly care about the number of repetitions of training movements but were more interested in whether they were training all the time. Similar to previous findings, Liaghat et al [71] found that supervised training had potential benefits regarding quality of life, dropout, and training adherence. Dalager et al [72] found that supervision was not a significant predictor of compliance, but compared with the control group, the intervention effect of the supervision group was more significant. It is suggested that in the future resistance training, the support and supervision equipment of mobile technology should be applied, combined with remote sensing technology, remote control technology and information processing technology. In this way, individual elderly people can carry out personalized exercise guidance, functional training, evaluation and supervision, timely correction of irregular training movements, enhance training compliance, and improve exercise effects. In the first study by Chen et al [55], the majority of participants tended to take part in a group of 15 to 20 participants at a frequency of three times for 40 minutes per week. In the second study [56], it was found that most participants preferred the frequency of exercise for an hour three times a week in groups of 20 to 29 people. Some studies maintained that group intervention was an effective way to promote healthy behavior [73, 74]. The intervention process was conducive to peer support and communication, meeting the needs of interpersonal communication among elderly individuals, reducing loneliness, and promoting mental health. Group training can create cohesion, provide fun, and promote behavioral change, also known as a sense of belonging in a group [40]. In addition, more than half of the participants preferred a female trainer to lead their workouts. They believed that a coach must be patient with the learner, and it would be better if it was someone they knew well. One reason might be that a familiar coach made participants less nervous, made it easier to establish a trusting relationship, and helped them feel more confident about the task, thus allowing them to engage in the exercise environment. At the same time, group training at a time convenient for participants could also improve the effect of the intervention.

### 4.3. Included study quality

This review used MMAT to assess the quality of the included studies, which varied from study to study. All studies had clear research questions, and the collected data could also answer the research questions.

For quantitative studies, the problems of randomized controlled studies focused on the random allocation method and blind implementation of outcome index evaluators. Ten randomized controlled studies did not introduce how to implement the random allocation method in detail but only mentioned randomness [43, 44, 49–52, 60–62, 64]. Ten studies did not report on whether the outcome indicator evaluators were blinded [44, 45, 49–51, 59–61, 63, 64], and two studies mentioned that the outcome indicator evaluators were not blinded [40, 53]. In future studies, the random assignment method should be reported on in detail, and the outcome index evaluators should be blinded to improve study quality. In the inclusion of non-randomized controlled studies, 4 studies did not take into account confounding factors that might affect the interpretation of the results, which could be improved in future research [42, 48, 58, 66]. For qualitative research, all quality evaluation criteria are satisfied [55, 56].

### 4.4. Limitations

This review has some limitations. First, resistance training had many benefits for elderly individuals, not only in terms of mental health and physical fitness but also in terms of sleep quality, blood pressure, bone density, blood sugar in serum, total cholesterol, high-density lipoprotein, low-density lipoprotein, and other levels. However, integration failed due to the small number of relevant studies. Second, there were only two qualitative studies and one mixed-methods study, which limited the integration and analysis of the qualitative studies, thus requiring careful interpretation of the results from the qualitative evidence. Third, the number of quantitative studies assessing mental health was limited, the assessment tools were not targeted, and the results need to be interpreted with caution. Future studies should pay attention to the impact of elastic band resistance training on the mental health of elderly individuals. Fourth, due to the majority of quantitative studies in this systematic review, which also caused limitations for the integration of quantitative and qualitative evidence. It was suggested that qualitative research on elastic band resistance training should be strengthened in the future to supplement the views and suggestions of older people regarding this intervention.

## 5. Conclusion

Elastic band resistance training appears to be effective in improving mental health, upper and lower limb flexibility, endurance, upper strength, and enhancing balance and cardiopulmonary function in elderly individuals. Despite the limited number of qualitative studies, consistent with quantitative evidence, participants felt that elastic band resistance training can offer physical and mental benefits. The preferences and obstacles encountered in implementing elastic band resistance training were evaluated to provide a reference for future research.

## Supporting information

S1 File (ZIP)
