# Supplementary material for: Effects of elastic band resistance training on the physical and mental health of elderly individuals: A mixed methods systematic review
Source: PLoS One. 2024 May 13;19(5):e0303372. doi: 10.1371/journal.pone.0303372 (PMC11090353; doi:10.1371/journal.pone.0303372)
Supplement: S1 File — (ZIP) [file pone.0303372.s001.zip › Supporting Information/Included study 58.pdf]

Health Promotion Outcomes of a Newly Developed Elastic Band Exercise Program for Older Adults in the Community: A Pilot Test

Shu-Ya Chan[1] • Chang-Chih Kuo[2] • Kuei-Min Chen[3]* • Wei-Shyuan Tseng[4]
Hsin-Ting Huang[5] • Chun-Huw Li[6]

[1]MS, RN, Doctoral Student, College of Nursing, Kaohsiung Medical University • [2]PhD, OT, Assistant Professor, Department of Occupational Therapy, Kaohsiung Medical University • [3]PhD, RN, Professor, College of Nursing, Kaohsiung Medical University • [4]BS, Yoga Instructor, Center for Continuing Education and Social Resources, Kaohsiung Medical University • [5]BS, Research Assistant, College of Nursing, Kaohsiung Medical University • [6]MS, RN, Lecturer, Department of Nursing, Yuh-Ing Junior College of Health Care and Management.

ABSTRACT

Background: Studies indicate that the incidence of disease, the mortality rate, and medical costs are significantly higher in people aged 65 years and over who do not engage in physical activities than in their peers who do engage in these activities. Therefore, promoting appropriate physical activity among older adults in the community is essential to delaying the health implications of aging.

Purpose: This pilot test was developed to assess the effectiveness of a newly developed Senior Elastic Band (SEB) exercise program on the health of older adults in community care stations.

Methods: A quasiexperimental design was used. A convenience sample of 20 participants from a community care station was recruited. The SEB intervention included three phases (warm-up, aerobic motion, and static stretching) and was conducted three times per week, 40 minutes per session for 1 month. Twelve health indicators in three categories (functional fitness, self-perceived health status, and sleep quality) were examined before and immediately after 1 month of SEB exercises.

Results: Participants showed improved performance at the end of the 1-month study for the following indicators: lung capacity, cardiopulmonary fitness, upper and lower body flexibilities, upper limb muscle power, lower limb muscle endurance, and self-perceived physical health status (all *p*s < .05). No significant differences were identified for the other indicators.

Conclusions/Implications for Practice: The SEB exercise program shows preliminary and promising effects on improving the health of older adults in a community care station. Healthcare professionals who work with older adults living in the community may consider the SEB exercise program as a health promotion modality to recommend and implement with this population. However, we recommend further testing the long-term effects of this program on a larger population.

KEY WORDS:
community care station, elastic band, health promotion, older adults.

Introduction

Healthcare for older adults should emphasize not only health maintenance but also health promotion. Health promotion stimulates the maximum body potential for older adults and reduces the impacts of aging (Lin & Lee, 2012). Older adults in communities should maintain an active lifestyle and exercise regularly (Chen, Lin, Wang, Huang, & Li, 2012). However, studies have found that older adults in Taiwan have low levels of physical activity (Lin, 2007). An increase in the level of physical activity should be vigorously planned to achieve healthy aging. The establishment of community care stations for older adults is one of the welfare policies in Taiwan, with a goal of promoting the physical and mental health of older adults living in the community and decreasing the negative effects of aging (Ministry of Interior, Taiwan, Department

Accepted for publication: October 14, 2014
*Address correspondence to: Kuei-Min Chen, No.100, Shih-Chuan 1st Rd., Sanmin District, Kaohsiung City 80708, Taiwan, ROC.
Tel: +886 (7) 3136900; E-mail: kmc@kmu.edu.tw
The authors declare no conflicts of interest.

Cite this article as:
Chan, S. Y., Kuo, C. C., Chen, K. M., Tseng, W. S., Huang, H. T., & Li, C. H. (2015). Health promotion outcomes of a newly developed elastic band exercise program for older adults in the community: A pilot test. *The Journal of Nursing Research*, *24*(2), 137–144. doi:10.1097/jnr.0000000000000099

Copyright © 2016 Taiwan Nurses Association. Unauthorized reproduction of this article is prohibited.

of Social Affairs, Taiwan, ROC, 2013). At the end of 2012, there were 1714 community care stations in Taiwan (Ministry of Interior, Affairs, Taiwan, ROC, 2013). Various types of dynamic and sedentary activities are held for the older adults at these stations. Implementing and encouraging this group of older adults to exercise regularly is essential to achieving health promotion goals (Hung & Chen, 2007).

The benefits of doing elastic band exercises have been confirmed for both healthy and frail older adults (Dancewicz, Krebs, & McGibbon, 2003; Topp, Woolley, Hornyak, Khuder, & Kahaleh, 2002). Evidence-based studies indicate that elastic band exercises help participants enhance muscle power and endurance (Binder et al., 2005; Zion, De Meersman, Diamond, & Bloomfield, 2003), stability and balance (Topp, Mikesky, Dayhoff, & Holt, 1996; Topp, Mikesky, Wigglesworth, Holt, & Edwards, 1993), flexibility and joint mobility (Sugimoto & Blanpied, 2006), vital capacity (Kim et al., 2012), and sleep quality (Chen, Tseng, Huang, & Li, 2013). Furthermore, elastic band exercises help participants improve their daily living functions (Topp et al., 2002), knee and hip extensions (Dancewicz et al., 2003), ability to stand up from a sitting position (Chen et al., 2009), ability to walk backward (Topp et al., 1993), and ankle dorsiflexion mobility and strength (Topp et al., 1996) as well as reduce knee pain (Topp et al., 2002) and body fat (Binder et al., 2005).

Chen, Tseng, et al. (2013) designed a Senior Elastic Band (SEB) exercise program for older adults that was adjustable to the physical activity tolerance levels of participants. Moreover, this program was designed to improve the six common health problems of older adults that were identified in our previous studies: (a) reduction of cardiovascular capacity, (b) reduced body flexibility, (c) weakened upper-limb muscle power, (d) weakened lower-limb muscle endurance, (e) poor balance, and (f) poor quality of sleep (Chen et al., 2012; Chen, Lin, Wang, Li, & Huang, 2013). The validity of the SEB exercise program was verified by 11 experts in the fields of elderly exercise, gerontological nursing, physical therapy, exercise physiology, sports medicine, and sports injuries (Chen, Tseng, et al., 2013). However, no studies have been conducted to elicit the health promotion effects of this newly developed SEB exercise program, which may be increasingly adopted by older adults. Therefore, this study conducted a pilot test to assess the effectiveness of the SEB exercise program on improving the health (functional fitness, self-perceived health status, and sleep quality) of older adults in community care stations.

Methods

Design

A quasiexperimental design that used one group and pretest and posttest was adopted. Before and at the end of 1 month of SEB exercises, participant variables were remeasured to explore the changes in functional fitness, self-perceived health status, and sleep quality.

Setting and Participants

This study was conducted at a community care station in Kaohsiung City in southern Taiwan. A convenience sampling method was used to recruit participants who met the following inclusion criteria: (a) over 60 years old, (b) never performed elastic band exercises, (c) able to stand without assistance, (d) intact cognitive functions (≥8 points on the Short Portable Mental Status Questionnaire), and (e) either mildly dependent or independent in activities of daily living (≥91 points on the Barthel Index). Individuals with severe or acute cardiovascular, musculoskeletal, or lung diseases were excluded from participation.

Ethical Considerations

Before conducting the intervention, the study was approved by the institutional review board of a university and the person in charge of the community care station. All participants were informed of the study by the authors. Participants indicated their consent to the study by signing or giving a thumbprint on the written consent form. During the study period, participants maintained the right to withdraw from the study at any time. All data were collected anonymously and were coded to maintain confidentiality. All participants were informed that the collected data would be used only in academic research.

Intervention

The SEB exercise program included three phases: (a) warm-up, with seven movements conducted over 12 minutes, aimed to loosen joints in all parts of the body and to improve mental, physical, and spiritual health; (b) aerobic motion, with seven movements conducted over 10 minutes, aimed to increase cardiovascular capacity and improve balance; and (c) static stretching, with six movements conducted over 18 minutes, aimed to enhance muscle power and endurance and improve body flexibilities (Chen, Tseng, et al., 2013). The intervention was conducted three times per week, 40 minutes per session for 1 month (Chen, Tseng, et al., 2013). Two instructors who had taken and passed the SEB instructor training program implemented the intervention. To prevent injuries, each SEB exercise was monitored by the research assistant, who was a licensed registered nurse. Twenty older adults participated in and completed the 1-month intervention (retention rate = 100%). The average attendance rate was 92.50%. The main reasons for absence were visiting relatives and being busy with family affairs.

Data Collection

The outcome measurements included (a) functional fitness, (b) self-perceived health status, and (c) sleep quality. The same research assistant (licensed registered nurse) collected all research data. Functional fitness included (a) cardiovascular–respiratory functions (resting blood pressure, lung capacity, and cardiopulmonary fitness), (b) body flexibilities, (c) muscle power and endurance, and (d) balance.

Copyright © 2016 Taiwan Nurses Association. Unauthorized reproduction of this article is prohibited.

Cardiovascular–respiratory functions

The resting blood pressure of participants was measured using a digital sphygmomanometer (OMRON-HEM707) after a 10-minute rest, with millimeter of mercury used as the recording unit. Lung capacity was measured using the TruZone peak flow meter, with liter used as the recording unit. Participants were asked after inhaling fully to exhale forcefully and quickly into the peak flow meter (MacDonald, Dixon, Cohen, & Hazlitt, 2004). Cardiopulmonary fitness was measured using the 2-minute step test. Participants were required to lift their knees to a height midway between the patella (knee cap) and the iliac crest (top of the hip bone). The total number of times the right knee of the participants reached the required height in 2 minutes was recorded (Rikli & Jones, 2001). The test–retest reliability was .90, and the criterion validity of the step test was .74 (Hung & Chen, 2007).

Body flexibility

Upper body flexibility was measured using the back scratch test (Rikli & Jones, 2001). Participants placed the dominant hand back over their shoulder and the other hand behind their back to measure the distance between the middle fingers. If the fingers did not touch, the distance was recorded as (−). If the fingers overlapped, the distance was recorded as (+). If the fingers just touched, the distance was recorded as 0 (Rikli & Jones, 2001). Lower body flexibility was measured using the chair sit-and-reach test (Rikli & Jones, 2001). During the measurement, participants sat on the one-third edge of a chair, one leg was bent with the foot flat on the floor, and the other leg was extended as straight as possible in front of the hip with the heel placed on the floor and with the foot flexed at approximately 90°. Participants were asked to stretch out their arms, hands overlapping, and slowly bend forward at the hip joint reaching as far forward as possible toward or past the toes. The distance between the middle fingers and the right hallux (big toe) was measured. If the fingers did not touch the right hallux, the distance was recorded as (−). If the fingers overlapped the right hallux, the distance was recorded as (+). If the fingers only touched the right hallux, the distance was recorded as 0 (Rikli & Jones, 2001).

Muscle power and endurance

The upper limb muscle power of the dominant hand of participants was measured twice using a digital handgrip dynamometer (TKK 5101). The better result was recorded, and the unit used in the measurement was kilogram (Brooks, Woodruff, Wright, & Donatelli, 2005). To ensure consistency, all participants used the second section of their fingers to squeeze the dynamometer. The standardized method was used to improve reliability. Lower limb muscle endurance was measured using the chair sit-to-stand test. Participants crossed their arms in front of their chest and sat on a chair without armrests. The total number of times that participants could completely stand up and sit down within a 60-second period was used to measure lower limb muscle endurance (Rikli & Jones, 2001).

Balance

The test of standing on one foot (dominant foot) with eyes closed was used to measure the participants' balance. Participants stood in their bare feet behind a chair, put their hands beside their body, closed their eyes, and lifted one foot 2–5 cm above the ground. The duration in seconds that each participant stood on one foot with eyes closed was recorded (Manini, Cook, VanArnam, Marko, & Ploutz-Snyder, 2006).

The functional fitness measuring instruments were calibrated regularly by specialists. The test–retest reliabilities of the measuring tests were ≥.95.

Self-perceived health status

The Chinese version of the 12-Item Short-Form Health Survey (SF-12) was used to measure participants' self-perceived health status, including physical and mental aspects (Ware, Kosinski, & Keller, 1996). The SF-12 is the shorter version of the SF-36, with 12 self-assessed items taken by the participants. The survey employed the Likert scoring method. Each item had a different scale. All scores were transformed into a scale of 0–100 points. A score of 0 point indicated the worst condition, and a score of 100 points indicated the best condition (Ware et al., 1996). The SF-12 includes two subscales: Physical component summary (PCS) and mental component summary (MCS), which measure eight health aspects. The PCS subscale includes physical function, role limitations because of physical health problems, bodily pain, and general health. The MCS subscale includes vitality, social functioning, role limitations because of emotional problems, and emotional well-being. The physical and mental scores of the SF-36 and SF-12 summary are highly correlated (*r*s = .95 and .97, respectively; Ware et al., 1996). Hung and Chen (2007) applied the SF-12 to assess the health status of urban older adults and obtained Cronbach's alpha values for the PCS and MCS subscales of .80 and .72, respectively. In this study, the PCS and MCS subscales obtained Cronbach's alpha values of .80 and .68, respectively.

Sleep quality

The Chinese version of the 18-item Pittsburgh Sleep Quality Index (PSQI) was used to assess participants' sleep quality during the previous month (Buysse, Reynolds, Monk, Berman, & Kupfer, 1989). The PSQI contains seven components: self-rated sleep quality (one item), sleep latency (two items), sleep duration (one item), habitual sleep efficiency (two items), sleep disturbances (nine items), daytime dysfunction (two items), and use of sleep medications (one item; Buysse et al., 1989). Each component was scored between 0 and 3, with total possible scores ranging from 0 to 21. Lower scores indicate better sleep quality, and a PSQI global score greater than 5 points indicates poor sleep quality (Buysse et al., 1989). The PSQI global score > 5 points correctly identified 88.50% as poor sleepers, with a sensitivity of 89.60% and a specificity of 86.50% (Buysse et al., 1989). Chen et al. (2010) applied the PSQI to assess the sleep quality

Copyright © 2016 Taiwan Nurses Association. Unauthorized reproduction of this article is prohibited.

of 55 older adults in assisted living facilities and obtained a Cronbach's alpha value of .76. The PSQI of this study obtained a Cronbach's alpha value of .78.

Data Analysis

Data were summarized and analyzed using the SPSS for Windows 18.0. Descriptive statistics were used to describe the demographic data of participants in terms of mean, standard deviation (*SD*), range, number of participant, and percentage (%). A paired *t* test was used to analyze the significant differences between the pretest and posttest means ($p < .05$ was used as the criterion for a significant difference).

Results

Demographic Characteristics of Participants

All participants (*N* = 20) completed the 1-month intervention. There were 18 female and 2 male participants, aged 67–85 years (72.00 ± 5.12 years). Most participants were widowed (70.00%) and living with family (65.00%). More than half of the participants exercised regularly (70.00%). The exercise frequency per week was 4.55 times (*SD* = 3.19), and the exercise duration was 41.25 ± 34.94 minutes. A large number of participants (40.00%) did regimental exercises (e.g., Tai Chi, Qi Gong, and Xiang Gong) followed by healthy gymnastics (30.00%). Most participants (70.00%) had chronic diseases, including hypertension (50.00%), diabetes (20.00%), and heart disease (10.00%). The average number of chronic diseases was 0.95 ± 0.76. The participants had intact cognitive functions, with a mean Short Portable Mental Status Questionnaire score of 9.75 ± 0.55 points. All participants were independent in activities of daily living and were able to take care of themselves. The average scores of participants on the Barthel Index were 100.00 ± 0.00 points (Table 1).

TABLE 1.
Demographic Characteristics of Participants (N = 20)

Variable	*n*	%	*M*	*SD*
Age (years)			72.00	5.12
Gender				
Male	2	10		
Female	18	90		
Marital status				
Married	6	30		
Widowed	14	70		
Educational level				
Illiterate	6	30		
Elementary school	7	35		
High school and above	7	35		
Religious belief				
No	3	15		
Yes	17	85		
Living status				
Live alone	7	35		
Live with family	13	65		
Exercise habit				
No	6	30		
Yes	14	70		
Chronic diseases			0.95	0.76
No	6	30		
Yes	14	70		
Exercise frequency (times/week)			4.55	3.19
Exercise duration (minutes/time)			41.25	34.94
SPMSQ			9.75	0.55
Barthel Index			100.00	0.00

Note. SPMSQ = Short Portable Mental Status Questionnaire.

Pretest and Posttest Differences in Terms of Functional Fitness, Self-Perceived Health Status, and Sleep Quality

Cardiovascular–respiratory functions

After 1 month of SEB exercises, the lung capacity of participants increased significantly from 282.00 ± 63.46 to 306.00 ± 52.05 L ($t(19) = -2.57$, $p = .019$), and cardiopulmonary fitness increased significantly from 61.75 ± 16.70 to 76.15 ± 18.45 steps ($t(19) = -4.52$, $p < .001$). The systolic and diastolic blood pressure of participants decreased slightly but not significantly ($t(19) = 0.21$, $p = .840$ and $t(19) = 0.91$, $p = .372$, respectively; Table 2).

Body flexibility

Upper body flexibility increased significantly from −5.25 ± 9.59 to −3.43 ± 9.86 cm ($t(19) = -2.40$, $p = .027$). Lower body flexibility increased significantly from 8.10 ± 7.86 to 12.53 ± 7.07 cm ($t(19) = -4.45$, $p < .001$; Table 2).

Muscle power and endurance

Upper limb muscle power (handgrip strength) increased significantly from 17.43 ± 6.78 to 20.27 ± 6.18 kg ($t(19) = -4.73$, $p < .001$). Lower limb muscle endurance increased significantly from 24.60 ± 7.03 to 27.85 ± 5.78 times ([$t(19) = -2.62$, $p = .017$; Table 2).

Balance

Results of the balance test showed a nearly significant increase between pretest and posttest ($t(19) = -2.01$, $p = .059$; Table 2).

Self-perceived health status (12-item short-form health survey)

After 1 month of SEB exercises, the PCS scores increased significantly from 51.29 ± 2.41 to 53.47 ± 1.75 points ($t(19) = -4.01$, $p = .001$). However, there were no significant

Copyright © 2016 Taiwan Nurses Association. Unauthorized reproduction of this article is prohibited.

TABLE 2.
Differences Between Pretest and Posttest on the Functional Fitness, Self-Perceived Health Status, and Sleep Quality of the Participants (N = 20)

Variable	Pretest		Posttest				
	M	*SD*	*M*	*SD*	*t*	*df*	*p*
Functional fitness							
Systolic blood pressure (mmHg)	132.60	18.34	131.90	13.44	0.21	19	.840
Diastolic blood pressure (mmHg)	72.90	11.65	70.80	9.57	0.91	19	.372
Lung capacity (L)	282.00	63.46	306.00	52.05	−2.57	19	.019*
Cardiopulmonary fitness (times)	61.75	16.70	76.15	18.45	−4.52	19	<.001***
Upper body flexibility (cm)	−5.25	9.59	−3.43	9.86	−2.40	19	.027*
Lower body flexibility (cm)	8.10	7.86	12.53	7.07	−4.45	19	<.001***
Upper limb muscle power (kg)	17.43	6.78	20.27	6.18	−4.73	19	<.001***
Lower limb muscle endurance (times)	24.60	7.03	27.85	5.78	−2.62	19	.017*
Balance (second)	3.46	2.69	4.52	3.22	−2.01	19	.059
Self-perceived health status							
Physical component summary (PCS)	51.29	2.41	53.47	1.75	−4.01	19	.001**
Mental component summary (MCS)	59.36	3.39	57.65	1.75	1.99	19	.062
Sleep quality	3.85	1.84	4.30	2.83	−0.95	19	.353

$^{*}p < .05$. $^{**}p < .01$. $^{***}p < .001$.

differences between pretest and posttest scores in terms of mental health ($t(19) = 1.99$, $p = .062$; Table 2).

Sleep quality (Pittsburgh sleep quality index)

The pretest score of PSQI was 3.85 ± 1.84 points, and the posttest score was 4.30 ± 2.83 points, indicating no statistically significant difference ($t(19) = -0.95$, $p = .353$; Table 2).

Discussion

After the 4-week SEB intervention, the lung capacity of participants increased significantly, which was consistent with the research findings of Kim et al. (2012). Furthermore, results indicated a significant improvement in the cardiopulmonary fitness of the participants, which has been little documented in the literature to date and thus represents a significant new finding of this study. This result may be because of the fact that the warm-up, aerobic motion, and static stretching of the SEB exercises enabled participants to increase their amount of physical activity, which improved blood circulation and cardiovascular capacity (Chen, Tseng, et al., 2013). Although there was no significant pretest/posttest difference in the systolic and diastolic blood pressure of participants, both pretest and posttest measurements were in the normal range.

In terms of body flexibility, participants had significant improvements in upper and lower body flexibilities, which were consistent with the results of other studies (Rogers, Sherwood, Rogers, & Bohlken, 2002; Zion et al., 2003). The static stretching of the SEB exercises emphasized stretching the upper and lower limb muscles through the use of elastic movements. By using slow movements, the upper and lower limb muscles could reach the maximal level of extension to improve joint mobility, muscle elongation, and muscle power and endurance. Therefore, the SEB exercises significantly improved body flexibility, muscle power, and muscle endurance (Chen, Tseng, et al., 2013).

With regard to balance, the results of the balance test showed a nonsignificant increase, which was consistent with the research findings of Bullani et al. (2011). However, the results of other studies (Topp et al., 1993; U.S. Department of Health and Human Services, 2003) have resulted in significant increases. Possible explanations include (a) the use in this study of a convenience sample of only 20 participants and (b) the short-term (4-week) duration of the intervention, which may be too short to detect a significant improvement in balance.

The findings indicated that the participants experienced a significant improvement in their self-perceived physical health status, which was consistent with the research findings of Sillanpää, Häkkinen, Holviala, and Häkkinen (2012). The stretching training of the SEB exercises helped participants stretch and loosen up their whole body, which promoted blood circulation and joint mobility, enhanced muscle power and endurance, and improved body flexibility. The SEB exercises increased the physical flexibility of the participants and thus had a significant impact on their self-perceived physical health.

The mental health of the participants did not improve significantly, which was consistent with the results of other studies (Damush & Damush, 1999; Oliveros et al., 2011; Segura-Ortí, Kouidi, & Lisón, 2009). A possible reason for this result was that mental health promotion requires a long-term, regular intervention. Further studies are needed to follow up on the efficacy of long-term interventions. Finally, no significant difference was found in the sleep quality of the participants. The pretest and posttest scores of the

Copyright © 2016 Taiwan Nurses Association. Unauthorized reproduction of this article is prohibited.

participants were all less than 5, which indicates that this group of participants was not sleep disturbed. Participants may have had good sleep quality because of their living in the community. Most had roommates, had few or no diseases, exercised regularly, and interacted with others at the community care station.

This newly developed SEB exercise program differed from other types of elastic band exercises in three ways: (a) the exercises in the SEB were less strenuous to accommodate the reductions in body flexibility and strength experienced by many older adults, (b) most of the exercises in the program emphasized only bending the knees and elbows slightly to protect joins, and (c) the thickness of the elastic band was "medium" to permit the level of resistance training to be adjusted up or down to accommodate the muscle strength of individual participants (Chen, Tseng, et al., 2013). Although the SEB program is not as strenuous as other types of elastic band exercises, this pilot study showed the potential effectiveness of this program on improving the health of older adults in community care stations. This was the first study to show that a less strenuous elastic band exercise program may achieve health promotion effects that are similar to the relatively more strenuous elastic band exercise programs described in the literature. Moreover, based on the baseline data, this particular sample appeared to be very healthy in general. This study revealed another important message that even a healthy group of older adults has the potential for health promotion, not just health maintenance. This finding supports Lin and Lee (2012) and is an essential message for those healthcare professionals working with the elderly populations.

Finally, as suggested by Chen, Tseng, Ting, and Huang (2007), it is important that exercise programs for older adults be manageable while challenging participants, so that older adults have an opportunity to improve, earn a sense of overcoming a challenge, and promote self-efficacy. If the exercise programs are overly complicated to learn or perform, they are likely to discourage older learners, undermine self-esteem, diminish interest, and decrease participation (Chen, Chen, & Huang, 2006). As evidenced in this study, the retention rate was 100%, and the average attendance rate was 92.50%, which indicated that the participants enjoyed the program. Therefore, this newly developed SEB exercise program is considered feasible for older adults who live in the community.

Study Limitations and Further Research Recommendations

This study had limitations in terms of sample selection and the research design used (one-group pretest and posttest). Our convenience sample was composed of only healthy older adults. Individuals in the sample had less than one chronic illness on average and were totally independent in their activities of daily living. Thus, the profile of participants may differ significantly from the older population in general. Furthermore, many participants were regular exercisers. The average exercise frequency of participants of 4.55 times per week and 41.25 minutes per session was higher than the intervention protocol of three times per week and 40 minutes per session. Participants all had regular exercise habits before participation, which continued during the 1-month study period. Although the additional 1-month SEB exercise program resulted in significant improvements in 7 of the 12 outcome indicators between pretest and posttest measurements, the lack of a control group introduced the risk that confounding factors may have affected these results, implying weaker inferences. This study was defined as a pilot test of the SEB exercise program. Although preliminary and promising outcomes were detected, results of this study should be explained and inferred carefully with regard to other populations.

In future studies, adopting a randomized controlled design with a two-group construct (experimental group and control group) and testing the long-term effects of the SEB exercise program in a larger population are recommended. A long-term, regular intervention is expected to result in better treatment effectiveness. Repeated measurement may be used to avoid the effect of maturation on the study results. In addition, applications of the SEB exercise program with less active or functionally dependent elderly participants should be tested.

Conclusions and Implications for Practice

This study preliminarily confirmed the positive effects of SEB exercises in terms of improving the health of older adults in community care stations. Moreover, the participants reported experiencing no negative effects or discomfort while doing these exercises. Therefore, healthcare professionals who work with older adults in the community may consider recommending or implementing the SEB exercise program as a health promotion modality for their older adult clients. Future studies should work to assess and validate the long-term effects of the SEB exercise program in a larger population.

Acknowledgments

Sincere appreciation is directed by our group to the National Science Council, Taiwan, for funding this study (NSC97-2314-B-037-052-MY3); to Professor Frank Belcastro for his superlative manuscript editing; to the administrator, staff, and volunteers of the community care station for their support and assistance; and to the 20 wonderful older adults for their generous participation.

References

Binder, E. F., Yarasheski, K. E., Steger-May, K., Sinacore, D. R., Brown, M., Schechtman, K. B., & Holloszy, J. O. (2005). Effects of progressive resistance training on body composition in frail older adults: Results of a randomized, controlled trial. *The Journals of Gerontology: Series A, Biological Sciences and Medical Science, 60*(11), 1425–1431. doi:10.1093/gerona/60.11.1425

Brooks, C. P., Woodruff, L. D., Wright, L. L., & Donatelli, R. (2005). The immediate effects of manual massage on power-grip performance after maximal exercise in healthy adults. *The Journal of Alternative and Complementary Medicine, 11*(6), 1093–1101. doi:10.1089/acm.2005.11.1093

Copyright © 2016 Taiwan Nurses Association. Unauthorized reproduction of this article is prohibited.

Bullani, R., El-Housseini, Y., Giordano, F., Larcinese, A., Ciutto, L., Bertrand, P. C., … Teta, D. (2011). Effect of intradialytic resistance band exercise on physical function in patients on maintenance hemodialysis: A pilot study. *Journal of Renal Nutrition*, *21*(1), 61–65. doi:10.1053/j.jrn.2010.10.011

Buysse, D. J., Reynolds, C. F. 3rd., Monk, T. H., Berman, S. R., & Kupfer, D. J. (1989). The Pittsburgh Sleep Quality Index: A new instrument for psychiatric practice and research. *Psychiatry Research*, *28*(2), 193–213. doi:10.1016/0165-1781(89)90047-4

Chen, K. M., Chen, M. H., Lin, M. H., Fan, J. T., Lin, H. S., & Li, C. H. (2010). Effects of yoga on sleep quality and depression in elders in assisted living facilities. *Journal of Nursing Research*, *18*(1), 53–61. doi:10.1097/JNR.0b013e3181ce5189

Chen, K. M., Chen, W. T., & Huang, M. F. (2006). Development of the simplified Tai-Chi exercise program (STEP) for frail older adults. *Complementary Therapies in Medicine*, *14*(3), 200–206. doi:10.1016/j.ctim.2006.05.002

Chen, K. M., Lin, M. H., Wang, Y. C., Huang, H. T., & Li, C. H. (2012). A model-based survey of physical health in community-dwelling older adults. *The Journal of Nursing Research*, *20*(4), 239–248. doi:10.1097/jnr.0b013e3182736571

Chen, K. M., Lin, M. H., Wang, Y. C., Li, C. H., & Huang, H. T. (2013). Psychological and socioeconomic health of community-dwelling older adults. *International Journal of Psychology*, *48*(6), 1038–1049. doi:10.1080/00207594.2013.771814

Chen, K. M., Tseng, W. S., Huang, H. T., & Li, C. H. (2013). Development and feasibility of a senior elastic band exercise program for aged adults: A descriptive evaluation survey. *Journal of Manipulative and Physiological Therapeutics*, *36*(8), 505–512. doi:10.1016/j.jmpt.2013.08.002

Chen, K. M., Tseng, W. S., Ting, L. F., & Huang, G. F. (2007). Development and evaluation of a yoga exercise programme for older adults. *Journal of Advanced Nursing*, *57*(4), 432–441. doi:10.1111/j.1365-2648.2007.04115.x

Chen, T. A., Wu, Y. T., Lee, M. B., Liang, K. C., Lin, K. N., & Tsai, M. W. (2009). Effects of exercise on depression symptoms, physical function, and quality of life in community-dwelling elderly. *Formosan Journal of Physical Therapy*, *34*(3), 209–218. (Original work published in Chinese)

Damush, T. M., & Damush, J. G. Jr. (1999). The effects of strength training on strength and health-related quality of life in older adult women. *The Gerontologist*, *39*(6), 705–710. doi:10.1093/geront/39.6.705

Dancewicz, T. M., Krebs, D. E., & McGibbon, C. A. (2003). Lower-limb extensor power and lifting characteristics in disabled elders. *Journal of Rehabilitation Research and Development*, *40*(4), 337–347. doi:10.1682/JRRD.2003.07.0337

Hung, H. M., & Chen, K. M. (2007). Effects of the simplified Tai-Chi exercise program in promoting the health of the urban elderly. *Journal of Evidence-Based Nursing*, *3*(3), 225–235. doi:10.6225/JEBN.3.3.225 (Original work published in Chinese)

Kim, J. H., Park, H. K., Jeon, S. Y., Oh, D. W., Park, H. J., & Park, W. J. (2012). Initial effect of an elastic chest band during inspiratory exercise on chest function improvement in people with limited lib mobility: A randomized controlled pilot trial. *Physiotherapy Research International*, *17*(4), 208–213. doi:10.1002/pri.1520

Lin, L. C., & Lee, M. D. (2012). Introduction. In L. C. Lin (Ed.), *Gerontological nursing* (6th ed., pp. 1–16). Taipei, Taiwan, ROC: Farseeing. (Original work published in Chinese)

Lin, P. Y. (2007). *Factors related to physical activity among the elderly in Taiwan* (Unpublished master's thesis). Chang Gung University, Taoyuan City, Taiwan, ROC. (Original work published in Chinese)

MacDonald, S. W., Dixon, R. A., Cohen, A. L., & Hazlitt, J. E. (2004). Biological age and 12-year cognitive change in older adults: Findings from the Victoria longitudinal study. *Gerontology*, *50*(2), 64–81. doi:10.1159/000075557

Manini, T. M., Cook, S. B., VanArnam, T., Marko, M., & Ploutz-Snyder, L. (2006). Evaluating task modification as an objective measure of functional limitation: Repeatability and comparability. *The Journal of Gerontology: Series A, Biological Sciences and Medical Sciences*, *61*(7), 718–725. doi:10.1093/gerona/61.7.718

Ministry of Interior, Taiwan, Department of Social Affairs, ROC. (2013). *Annual report of social developments*. Retrieved from http://www.sfaa.gov.tw/ (Original work published in Chinese)

Oliveros, R. M. S., Avendaño, M., Bunout, D., Hirsch, S., De La Maza, M. P., Pedreros, C., & Müller, H. (2011). A pilot study on physical training of patients in hemodialysis. *Revista Médica De Chile*, *139*(8), 1046–1053.

Rikli, R. E., & Jones, C. J. (2001). *Senior fitness test manual*. Champaign, IL: Human Kinetics.

Rogers, M. E., Sherwood, H. S., Rogers, N. L., & Bohlken, R. M. (2002). Effects of dumbbell and elastic band training on physical function in older inner-city African-American women. *Women & Health*, *36*(4), 33–41. doi:10.1300/J013v36n04_03

Segura-Ortí, E., Kouidi, E., & Lisón, J. F. (2009). Effect of resistance exercise during hemodialysis on physical function and quality of life: Randomized controlled trial. *Clinical Nephrology*, *71*(5), 527–537. doi:10.5414/CNP71527

Sillanpää, E., Häkkinen, K., Holviala, J., & Häkkinen, A. (2012). Combined strength and endurance training improves health-related quality of life in healthy middle-aged and older adults. *International Journal of Sports Medicine*, *33*(12), 981–986. doi:10.1055/s-0032-1311589

Sugimoto, D., & Blanpied, P. (2006). Flexible foil exercise and shoulder internal and external rotation strength. *Journal of Athletic Training*, *41*(3), 280–285.

Topp, R., Mikesky, A., Dayhoff, N. E., & Holt, W. (1996). Effect of resistance training on strength, postural control, and gait velocity among older adults. *Clinical Nursing Research*, *5*(4), 407–427. doi:10.1177/105477389600500404

Topp, R., Mikesky, A., Wigglesworth, J., Holt, W. Jr., & Edwards, J. E. (1993). The effect of a 12-week dynamic resistance strength training program on gait velocity and balance of older adults. *The Gerontologist*, *33*(4), 501–506. doi:10.1093/geront/33.4.501

Topp, R., Woolley, S., Hornyak, J. 3rd., Khuder, S., & Kahaleh, B. (2002). The effect of dynamic versus isometric resistance training on pain and functioning among adults with osteoarthritis of the knee. *Archives of Physical Medicine and Rehabilitation*, *83*(9), 1187–1195. doi:10.1053/apmr.2002.33988

U.S. Department of Health and Human Services. (2003). *With chart book on trends in the health of Americans*. Hyattsville, MD: Author.

Ware, J. Jr., Kosinski, M., & Keller, S. D. (1996). A 12-item short-form health survey: Construction of scales and preliminary tests of reliability and validity. *Medical Care*, *34*(3), 220–233.

Zion, A. S., De Meersman, R., Diamond, B. E., & Bloomfield, D. M. (2003). A home-based resistance-training program using elastic bands for elderly patients with orthostatic hypotension. *Clinical Autonomic Research*, *13*(4), 286–292. doi:10.1007/s10286-003-0117-3

Copyright © 2016 Taiwan Nurses Association. Unauthorized reproduction of this article is prohibited.

新發展的彈力帶健身操方案對社區老人健康促進之成效：前驅性試驗

詹淑雅[1] 郭昶志[2] 陳桂敏[3]* 曾維璇[4] 黃欣婷[5] 李純華[6]

[1]高雄醫學大學護理學系研究所博士班研究生 [2]高雄醫學大學職能治療學系助理教授
[3]高雄醫學大學護理學系教授 [4]高雄醫學大學推廣教育暨社會資源中心瑜珈指導老師
[5]高雄醫學大學護理學系研究助理 [6]育英醫護管理專科學校護理科講師

背 景 研究顯示65歲以上的老人若無從事身體活動，其罹病率、死亡率及醫療支出將大大提升。為了延緩老化過程對健康所造成的影響，社區老人之健康促進極為重要。

目 的 前驅測試新發展之「銀髮彈力帶健身操」方案對社區關懷據點老人健康促進之成效。

方 法 本研究採單組、前後測之類實驗性設計，以方便取樣方式選取20位社區照顧關懷據點老人參與本研究。「銀髮彈力帶健身操」介入措施包括三個階段(舒筋暖身、動態有氧、靜態伸展)，每週進行3次，每次40分鐘，為期一個月。研究個案之健康測量指標含三大類(功能性體適能、自覺健康狀態及睡眠品質)，共12個變項。測量時間點為介入措施執行前及完成一個月之介入措施後再度測量。

結 果 在為期一個月的研究結束後，研究個案的肺活量、心肺適能、肩臂柔軟度、坐姿體前彎、上肢肌力、下肢肌耐力及自覺身體健康狀態等7個項目皆有顯著改善(所有p值 $<$ .05)，其餘變項皆無顯著差異。

結 論／實務應用 研究初步支持「銀髮彈力帶健身操」對社區關懷據點老人之健康促進有正面的效果。建議從事社區老人照護之健康照護專業人員可考量將「銀髮彈力帶健身操」納為社區老人健康促進方法之一，推廣並鼓勵社區老人規律練習。建議未來擴大測試此運動方案於大樣本之老人族群，並檢視其長時間介入之成效。

關鍵詞：社區照顧關懷據點、彈力帶、健康促進、老人。

接受刊載：103年10月14日
*通訊作者地址：陳桂敏 80708高雄市三民區十全一路100號
電話：（07）3136900 E-mail: kmc@kmu.edu.tw

Copyright © 2016 Taiwan Nurses Association. Unauthorized reproduction of this article is prohibited.
