# Supplementary material for: Effects of elastic band resistance training on the physical and mental health of elderly individuals: A mixed methods systematic review
Source: PLoS One. 2024 May 13;19(5):e0303372. doi: 10.1371/journal.pone.0303372 (PMC11090353; doi:10.1371/journal.pone.0303372)
Supplement: S1 File — (ZIP) [file pone.0303372.s001.zip › Supporting Information/Included study 62.pdf]

A Home-based Exercise Program for Nondisabled Older Adults

Alan M. Jette, PT, MPH, PhD,[*†] *Bette Ann Harris, PT, MS,*[*‡§||] *Lynn Sleeper, ScD*[*] *Margie E. Lachman, PhD*[¶] *Diane Heislein, MS, PT, OCS*[§] *Marie Giorgetti, MS, PT, NCS*[§] *and Claudia Levenson, MS, PT*[§**]

OBJECTIVES: This paper describes a videotaped, home-based, strength training program, titled **Strong-for-Life** and reports on its effectiveness in improving muscle strength, psychological well-being, and health status in a sample of older persons.

DESIGN AND SETTING: We enrolled 102 nondisabled, community-dwelling older people aged 66 to 87, identified from the Medicare beneficiary list, into a randomized, controlled trial.

MEASUREMENTS: Effectiveness was based on change in isokinetic upper and lower extremity muscle strength, psychologic well-being, and health status.

RESULTS: Results revealed several statistically significant short-term benefits after 12 to 15 weeks of exercise, especially for men. Younger older adults demonstrated a 10% improvement in knee extensor strength relative to control subjects. Older male exercisers achieved significant differences relative to controls in perceived anger, tension, and overall social functioning. Male exercisers, in general, achieved significant improvement in perceived vigor. Women did not report psychological benefits following participation in the program.

CONCLUSION: Study results reveal that the **Strong for Life** program, designed to be widely disseminated to the nondisabled older population, has many short-term positive benefits. **J Am Geriatr Soc 44:644–649, 1996.**

Several exercise studies throughout the past 15 years have demonstrated the many positive benefits of enhancing levels of physical activity in older persons.[1–11] Typically, however, these earlier exercise trials in older samples have been conducted with small numbers of highly selected individuals who have participated in supervised training programs conducted in laboratory or clinical facilities. Although they provide the literature with important results regarding the efficacy of exercise in older persons, they tell us little about the feasibility and effectiveness of enhancing physical activity levels among older persons under more representative circumstances.[5] Given that the vast majority of older adults engage in no regular physical exercise,[12] feasible and effective exercise intervention strategies are needed that can be widely disseminated to the older population in need of increased activity.

The purpose of this paper is to describe an in-home strength training strategy, called *Strong-for-Life*, which is designed specifically for older persons, and to report on its effectiveness in a community-dwelling sample of nondisabled persons 65 years of age and older. Specifically, we hypothesized that community-dwelling, nondisabled, older persons would perform the *Strong-for-Life* program regularly and that regular strength training of this nature would result in increased upper and lower extremity strength, enhanced psychological well-being, and measurable improvements in overall health status.

METHODS

Subjects and Recruitment

The target population for this field trial was nondisabled, community-dwelling persons aged 65 years and older. The sampling frame consisted of a random sample of Medicare beneficiaries aged 65 and older residing in communities of Boston and East Cambridge, Massachusetts. Exclusion criteria, based on history, were: significant coronary artery disease, angina, congestive heart failure, a myocardial infarction, cardiac surgery, or significant or new onset rhythm disturbance; neurological disorders with residual deficit; renal failure requiring dialysis; recent cancer with active chemotherapy or radiation treatment; uncontrolled hypertension, diabetes, or seizure disorders; recent fracture; legal blindness; major mobility limitations. All subjects received written clearance from their primary care physician, who documented no contraindications for strength training. In addition, subjects passed an exercise safety evaluation that

From the *New England Research Institutes, Watertown, †Boston University School of Public Health, ‡MGH Institute of Health Professions, §Mass General Hospital, ||Harvard Medical School, Boston, ¶Brandeis University, Waltham, and **Beth Israel Hospital, Boston Massachusetts.

This study was supported by Grant #AGO9715 from the National Institute on Aging and, in part, by the Roybal Center for Research in Applied Gerontology (#AG11669).

Address correspondence to Alan M. Jette, PhD, New England Research Institutes, 9 Galen St., Watertown, MA 02172.

© 1996 by the American Geriatrics Society
0002-8614/96/$3.50

consisted of an assessment of resting heart rate and blood pressure and a treadmill exercise tolerance test using a modified Bruce protocol, as well as physiologic responses to isokinetic exercise. Persons were excluded if resting heart rate was greater than 120 bpm, resting systolic/diastolic blood pressure was greater than 165/100 or less than 80/50, or if they failed the exercise tolerance tests. As the final criteria for participation in the study, subjects had to be English speaking and had to have access to a videotape player (VCR) or be willing and able to use one provided by the project.

In total, we attempted to reach 2554 names from the sampling frame. Of these, 711 (27.8%) could not be reached either by telephone, by mail, or through a proxy. Of the remaining 1843 potential participants, 1517 (82.3%) did not meet the study's eligibility requirements. Of the 326 eligible individuals, 102 (31.3%) enrolled into the study, with 52 randomized as controls and 50 assigned to the intervention group. Refusers, compared with those who enrolled in the program, were not different with respect to health or functional status but were older (mean age of 75 years among refusers vs mean age of 72 years among participants; $P = .0001$) and had less formal education ($P = .004$).

Subjects' medical history, typical activity patterns, psychological well-being, and health status were assessed at baseline by trained telephone interviewers. Each participant who appeared to be eligible received an exercise safety test and strength testing during a baseline clinic visit and between 12 to 15 weeks after the baseline testing and entry into the study. Subjects who met the study criteria, agreed to participate, and gave written informed consent were randomized after baseline testing to either receive the exercise program immediately (intervention group) or to be placed on a waiting list for the program (control group) at the completion of the study. All telephone interviewers and clinic assessment staff were blinded to the subjects' group status at the follow-up contact. Assessment of pre- and post-study habitual physical activity using the Physical Activity Scale for the Elderly (PASE)[13] confirmed that the controls, compared with the intervention group, did not alter their habitual physical activity levels during the study period.

Of the 102 randomized subjects, one control and eight experimental subjects dropped out of the study. Two subjects dropped out because of the exercise program (one developed shortness of breath after doing the exercises and another developed back symptoms from the program). An additional three subjects developed medical problems unrelated to the study, and the remaining four dropouts cited lack of interest.

Dropouts did not differ from those who completed the study with respect to age, gender, education, or health status.

An additional 10 randomized subjects (3 controls and 7 intervention subjects) completed all baseline and follow-up telephone assessments but did not complete the final strength testing portion of the protocol. They are included in those analyses that used health and psychologic status data provided through the telephone interview.

Outcome Measures

Strength

A Cybex II isokinetic dynamometer (Cybex Division of Lumex Inc., Bayshore, NY) was used to test peak torque in the right lower extremity knee extensors and flexors and in the right upper extremity shoulder extensors and flexors. Data showing its reliability and stability when used in older samples are available.[14] When a painful condition or recent injury or surgery prevented use of the right extremity for testing, the left extremity was tested. The detailed strength testing protocols are available from the authors.

Psychological Well-Being

A shortened form of the Profile of Mood States battery (POMS)[15] was administered by telephone at the pretest and posttests. In past work, the POMS has been found to be useful for assessing emotional states in older adults.[16] We developed a 33-item version using the items with the highest item-to-total correlations reported for each subscale in the test manual.[15] We selected the best five items for each scale, with the exception of the depression-dejection subscale, for which we selected the best eight items. The 33-item version used is similar to the new 30-item POMS-SF.[17] Coefficient alpha internal consistency reliabilities for the sample on the subscales are: Tension-Anxiety, 0.79; Depression-dejection, 0.86; Vigor, 0.88; Fatigue, 0.89; Anger, 0.73; and Confusion, 0.73. Items were rated on a five-point scale using the following question: "Over the past week, including today, would you say you have been feeling (insert item) not at all, a little, moderately, quite a bit, or extremely?"

Health Status

Eight dimensions of health status were assessed: physical functioning, role limitations due to physical or emotional health, social activity limitations attributable to health, current health perceptions, pain, psychologic distress/well-being, and energy/fatigue. These health dimensions have been identified as likely to be affected by exercise.[18,19] These dimensions were measured using scales from the Medical Outcomes Study SF-36.[20] Each standardized scale ranges from 0 to 100, where 0 = worst possible function and 100 = best possible function in that dimension. The reliability and validity of the SF-36 have been examined extensively and were summarized recently by Stewart and Ware.[21] It has been shown to be sensitive to change in exercise training studies involving nondisabled older persons.[19,9]

Exercise Program

The *Strong-for-Life* program consisted of a 30-minute videotaped program of 10 exercise routines using elastic bands (Therabands®) that provided individualized levels of resistance to the exercisers. The program consisted of a 5-minute warm-up, 20 minutes of strengthening exercises, and 5 minutes of cool-down exercises done to music and led by a trained instructor with older participants. The goal of the subjects was to perform the program 3 times each week during the 12 to 15-week study period.

The strengthening component of the program consisted of exercises performed in a progressive weight-bearing sequence from prone lying to standing. This developmental sequence used trunk and proximal extremity musculature and facilitated movement patterns that are commonly lacking in traditional weight training programs. The exercises consisted of 10 single joint, whole limb, and trunk movements that incorporated diagonal and rotational motions associated with proprioceptive neuromuscular facilitation patterns.[22,23] Subjects were instructed to perform up to 10 repetitions of each movement pattern. When this was accomplished without significant fatigue or loss of proper execution, resistance

was increased. Progressive resistance was provided through the use of Therabands, where bands of graded thickness provided progressive increments of resistance. Subjects advanced within the program at their own pace in consultation with a physical therapist who provided periodic telephone follow-up during the intervention period.

Each subject assigned to the "exercise group" underwent a 50-minute training session where he/she met with a physical therapist who discussed the benefits of exercise and then instructed the subject in the exercise program. The training therapist called each subject 24 hours after the initial training session and again after 1, 2, 4, 8, and 11 weeks to check on progress and to discuss and resolve problems encountered.

Adherence

Adherence to the intervention protocol was based on self-report. Subjects were instructed to complete exercise logs on which they listed the date, length, and a global rating of the intensity of the exercises. Colored markers were used to record in the exercise log the thickness of Theraband used. Subjects were given self-addressed, stamped envelopes and instructed to mail the completed logs back to the training therapist monthly.

Statistical Methods

Baseline strength, psychologic, and health status measures by treatment group were compared using Student's *t* test for normally distributed variables. The Wilcoxon rank-sum test was used for non-normally distributed continuous variables. Fishers' Exact test was used to examine associations between treatment group and categorical variables. The effect of the intervention was evaluated by comparing change scores of the intervention and control using analysis of covariance, with the baseline measurement, continuous age, and gender as covariates. For each dependent variable, mean change adjusted for baseline value, age, and gender was estimated for the two treatment groups. When there was a significant interaction between treatment group and gender, the baseline-adjusted means were presented by gender as well as by treatment group. When there was a significant interaction between treatment group and age, the model was re-estimated using dichotomous age ($\leq$ 72 years vs > 72 years), and adjusted means were presented by treatment and age group. Effect sizes were calculated using the square root of the Mean Squared Error from the corresponding regression model. Cohen suggests the following interpretation of effect size magnitudes: 0.2 - 0.4 = small effect; 0.5 - 0.7 = moderate effect; 0.8 + = large effect.[24]

RESULTS

Baseline characteristics by treatment group for the 93 subjects who completed the study are displayed in Table 1. Subjects ranged in age from 66 to 87 years and were predominantly women. Control subjects, on average, were significantly older than those in the exercise group, and there was a trend toward more women in the control group compared with the exercise group. Because of these differences, all analyses were conducted with subject age and gender as covariates in the models. Table 2 displays baseline strength, psychological well-being, and health status for subjects by study group.

On average, intervention subjects performed 30 sessions of *Strong-for-Life* during the study period, approximately two exercise sessions per week. Average adherence rates for this program (expressed as the number of exercise sessions reported as a percentage of exercise sessions prescribed for the study) were 58%, and they ranged from 0 to 102%, with a median of 71%.

Changes in muscle strength by study group during the 12 to 15 week-study period are presented in Table 3. Results illustrate a trend of overall increase in knee flexion and knee extension torque and maintenance of shoulder flexion torque after the intervention among the exercise group compared with controls. Analysis also revealed a significant Age × Group interaction for change in knee extension torque, with exercise group subjects aged 72 years or less experiencing a

Table 1. Baseline Characteristics of the Subjects

	Exercise Group (n = 42)	Control Group (n = 51)	*P* Value
Age, yrs. (range)	71.0 ± 4.3 (66–84)	73.2 ± 5.4 (66–87)	.04
	(n = 42)	(n = 51)	
Female sex (% of subjects)	54.8	70.6	.13
Education (% of subjects)	(n = 42)	(n = 50)	
Elementary school	2.4	0.0	.66
Some high school	33.3	38.0	
Beyond high school	64.3	62.0	
Annual income (% of subjects)	(n = 39)	(n = 48)	
<$10,000/yr.	15.4	10.4	.75
$10–20,000/yr.	25.6	31.3	
>$20,000/yr.	59.0	58.3	
Weight (x lbs.)	162.4	146.7	.01
Perceived health (% of subjects)			
Excellent	16.6	27.4	.31
Very Good	40.5	45.1	
Good	40.5	23.5	
Fair/Poor	2.4	4.0	

Table 2. Baseline Strength, Psychological Well-Being, and Health Status Characteristics of Subjects

	Exercise Group	Control Group	*P* Value
Knee strength (ft. lbs.)	(n = 35)	(n = 48)	
Extension torque	48.8 ± 18.5	45.6 ± 19.1	.44
Flexion torque	27.2 ± 11.4	24.8 ± 11.9	.36
Shoulder strength (ft. lbs.)	(n = 34)	(n = 47)	
Extension torque	31.7 ± 13.4	26.1 ± 11.2	.04
Flexion torque	17.4 ± 7.8	15.7 ± 7.4	.30
Psychologic Status	(n = 42)	(n = 51)	
Tension	6.9 ± 2.0	7.2 ± 2.6	.47
Vigor	15.2 ± 4.0	16.4 ± 5.0	.21
Depression	10.5 ± 2.7	10.6 ± 4.4	.86
Fatigue	9.0 ± 3.1	8.8 ± 3.6	.76
Anger	7.3 ± 2.0	7.2 ± 2.7	.78
Confusion	7.0 ± 2.2	6.9 ± 2.2	.76
Health Status			
Health perceptions	78.5 ± 13.1	79.4 ± 15.3	.77
Physical function	86.1 ± 12.9	86.6 ± 14.0	.88
Energy	66.8 ± 14.8	64.2 ± 20.8	.49
Role function (physical)	89.9 ± 22.8	91.2 ± 21.7	.78
Role function (emotional)	94.4 ± 22.0	90.8 ± 25.9	.48
Social function	93.2 ± 12.1	95.3 ± 12.7	.40
Pain	86.4 ± 18.1	83.9 ± 19.8	.52
Mental Health	80.9 ± 11.9	80.7 ± 12.6	.94

significant increase in knee extension torque of approximately 10% over baseline whereas no change was observed in controls ($P = .007$). In contrast, older subjects did not experience a significant change in knee extension torque regardless of treatment group.

Results of analyses of change in psychological well-being are presented in Table 4. These analyses revealed several significant interaction effects. There were two Age × Gender × Group interactions, consistent with the basic study hypotheses. The first revealed that **older men** in the control group experienced a significant increase in **anger** relative to the men who participated in the intervention. The second interaction revealed a borderline group difference ($P = .09$) among **older men: tension** decreased after the intervention but increased in men assigned to the control group. In addition, there was a significant Gender × Group interaction for **vigor:** men experienced significantly more vigor postintervention compared with men in the control group, who experienced significantly less vigor. This group difference was not observed in women. Two significant findings that were not hypothesized included a significant group difference in anger in younger men ($P = .01$) attributable to a significantly decreased anger in the control group and a significant group difference in confusion among older women ($P = .002$) caused by significantly increased confusion after the intervention.

Only one health status outcome was observed. There was an Age × Group interaction with social functioning, which revealed that older subjects in the intervention group had a significantly higher increase in **social functioning** ($P = .04$) relative to older control subjects whose social functioning significantly decreased. This effect was not observed in the younger age group.

DISCUSSION

The findings from this investigation demonstrate that a 12 to 15-week, home-based, strengthening program designed for nondisabled, community-dwelling, older persons, did achieve positive improvements in strength, psychological well-being, and social health status. Overall, lower extremity strength improved among participants, but degree of improvement was modest. The 10% improvement in knee extension strength for younger participants achieved in this study is somewhat less than levels achieved in more supervised training programs, but meaningful given the low level of supervision in the program.[7,25]

Several dimensions of psychological well-being improved, particularly for men and older subjects. Consistent with earlier work,[6] older male exercisers reported a significant difference in perceived anger following their participation in the program.

Male exercisers of all ages reported feeling more lively, active, and energetic following the intervention. In contrast, men in the control group showed a significant decrease in feelings of vigor and pep from the pretest to the posttest. Thus, the intervention served not only to enhance psychological well-being, but it also appeared to minimize declines that might otherwise prevail among older men. There was also a trend for older men in the exercise group to report decreased tension compared with the control group. Thus, the exercise program may have led to reduced feelings of nervousness, uneasiness, and shakiness for older men. Our findings are consistent with other studies that have demonstrated psychological benefits in middle-aged and older subjects who exercise.[6,26]

Women reported no psychological improvement following participation in the program, and older women appeared to report increased confusion after their participation in the program. In future work, it will be important to explore why women subjects did not respond to this program as well as men, identify possible negative implications of program termination, and find adaptive strategies for maintaining involvement and commitment to the exercise program even after the formal study is completed.

For older subjects, significant improvement in social functioning followed participation in the program, signifying increased social contact with family, friends, neighbors, and groups following participation in the program and a substantial drop in social function for the older members of the control group. This finding suggests that participating in the exercise program may have enabled more contact with friends and family by enhancing mobility, thereby curtailing a reduction in social function often found among the oldest old. There were, however, no measurable improvements in other dimensions of health status. This, in part, may be attributable to a ceiling effect for these variables inasmuch as all subjects were nondisabled.[19]

The study's recruitment procedures, which utilized the Medicare beneficiary list as the sole sampling frame, did achieve a more representative sample than has been enrolled in previous exercise studies with volunteer subjects. A com-

Table 3. Change in Muscle Strength*

	Exercise group		Control Group		
	Mean SE	ES	Mean SE	ES	*P* Value
Knee strength (ft. lbs)					
Extension torque	3.2 ± (1.2)	0.4	0.2 ± (1.1)	0.03	.074
≤72 yrs.	4.4 ± (1.4)	0.7	−1.0 ± (1.5)	−0.2	.007
>72 yrs.	−0.1 ± (2.4)	−0.02	1.1 ± (1.7)	0.2	.695
Flexion torque	5.8 ± (1.2)	0.8	3.3 ± (1.1)	0.5	.117
Shoulder strength (ft. lbs)					
Extension torque	3.0 ± (1.0)	0.5	1.0 ± (0.9)	0.2	.136
Flexion torque	−0.3 ± (0.7)	−0.1	−1.9 ± (0.6)	−0.5	.067

* Data are presented as means ± standard error and effect size. All change scores and analyses are adjusted for baseline measurement of the same variable, age, and gender.

Table 4. Change in Psychological Well-Being*

	Exercise Group			Control Group			
	Mean	SE	ES	Mean	SE	ES	*P* Value
Psychological Status:							
Tension	0.22	(0.46)	0.08	0.33	(0.45)	0.11	0.87
Male, ≤72	0.99	(0.84)	0.34	−0.96	(0.96)	−0.33	0.13
Male, >72	−1.31	(1.10)	−0.45	1.41	(1.18)	0.49	0.09
Female, ≤72	0.47	(0.66)	0.16	1.38	(0.63)	0.48	0.32
Female, >72	1.28	(1.44)	0.44	−0.24	(0.74)	−0.08	0.35
Vigor	0.43	(0.46)	0.15	−0.93	(0.46)	−0.32	0.04
Male	1.60	(0.70)	0.55	−2.18	(0.76)	−0.74	0.01
Female	−0.75	(0.62)	−0.26	0.32	(0.50)	0.11	0.18
Depression	−0.35	(0.35)	−0.16	0.32	(0.33)	0.14	0.18
Fatigue	−0.26	(0.50)	−0.12	−0.05	(0.47)	−0.02	0.76
Anger	0.15	(0.33)	0.07	−0.27	(0.32)	−0.13	0.36
Male, ≤72	0.90	(0.59)	0.44	−1.53	(0.67)	−0.76	0.01
Male, >72	−0.45	(0.76)	−0.22	1.97	(0.83)	0.98	0.03
Female, ≤72	0.13	(0.47)	0.06	−0.31	(0.44)	−0.15	0.49
Female, >72	0.44	(1.01)	0.22	0.04	(0.52)	0.02	0.73
Confusion	0.16	(0.30)	0.08	−0.07	(0.29)	−0.04	0.59
Male, ≤72	−0.28	(0.54)	−0.15	−0.59	(0.63)	−0.32	0.71
Male, >72	−0.29	(0.72)	−0.16	0.46	(0.76)	0.24	0.48
Female, ≤72	−0.19	(0.43)	−0.10	0.38	(0.41)	0.20	0.33
Female, >72	2.83	(0.93)	1.51	−0.52	(0.48)	−0.28	0.01

* Data presented are means ± standard error and effect size. All change scores and analyses are adjusted for baseline measurements of the same variable, age, and gender.

parison of the demographic data in Table 1 with the 1990 census data revealed that the resulting sample was very similar to the older population in Boston, although it was somewhat younger, better educated, and had higher annual incomes, on average, than the older population of the communities from which the sample was drawn.

Variability in adherence to the *Strong-for-Life* program explains some of the selectivity of benefits achieved from the program and points to areas where improvement in home-based programs is needed. The low level of professional supervision of the program is a likely contributor to the variability of adherence observed in this study. The current study included only one in-person training session with a physical therapist, with periodic telephone follow-up. Using a similar protocol with middle-aged subjects, aged 50 to 65,[5] achieved adherence rates of 75% or higher compared with a mean of 58% in this study. In this study, subjects were considerably older, with ages ranging from 66 to 87 years of age. Home-based exercise programs with subjects in their 70s and 80s may require more supervision, possibly involving more than one training visit, ideally in the subject's own home, as well as more frequent telephone follow-up of subjects, to maintain adherence and to maximize potential benefits.

We had hypothesized that improvement in muscle strength and psychological and health status outcomes would be associated positively with the frequency and dose of exercise received during the study. To examine this hypothesis, we created an exercise dosage variable that was the product of the number of exercise sessions participated in during the study period and the level of Theraband resistance used for each exercise session (as reflected by the 4 available colors of

Theraband used in this study). As hypothesized, the exercise dosage variable was correlated positively with improvement in knee extension ($r = .38$; $P = .03$).

The most likely explanation for some of the age group and gender differences observed in the study results is differential adherence to the program. Although limited sample size made it difficult to explore this hypothesis fully, subgroup analyses did reveal a nonsignificant ($P = .15$) trend of higher adherence to the program among subjects 72 years of age or younger compared with those older than 72 and for men compared with women ($P = .07$). Adherence rates, however, were unrelated to psychological benefits and improvement in social functioning. Older men may be more attracted to an in-home program they can do on their own, whereas women may find the social component of group exercises more appealing. The preponderance of psychological benefits among men and not women subjects is an area that deserves further investigation.

Another challenge encountered was subject reluctance to progress with the thickness of Theraband used in the exercise program. Although the design of the program called for gradual progression in resistance throughout the 12 to 15 weeks, progression to higher levels of resistance was difficult to achieve. Among the exercise participants, 9% did not change their level of Theraband resistance at all throughout the study period, while another 35% progressed by only one of the four possible levels of Theraband. Future studies may also benefit from tailoring the exercises more to individual needs, implementing specific behavioral strategies to progress in the program, and working more with participants to set realistic but challenging goals.

This investigation revealed several positive, short-term physiological, psychological, and social benefits that accrued to older persons in their 60s, 70s, and 80s as a result of participating in a home-based, strength training program. Physiological results are clearest among those less than age 73, whereas psychological and social benefits are seen predominantly among older participants, particularly men. These findings lend further support to the literature on the positive benefits of regular exercise in late life in a program designed to be widely disseminated among the older population in need of increased physical activity.

ACKNOWLEDGMENTS

The authors thank Ms. Shenkman and the field staff of NERI, Mr. Paul Mitchell, who performed the statistical programming, and Ms. Lisa King, who prepared the manuscript for publication. Copies of the final version of the *Strong-for-Life* videotape can be purchased from Baxley Media Group, 110 West Main, Urbana, IL 61801. (800) 421-6999. www.prairienet.org/business/bax/home.htm

REFERENCES

1. Agre J, Pierce, Raab D et al. Light resistance and stretching exercises in elderly women: Effect upon strength. Arch Phys Med Rehabil 1988;69:273–276.
2. Aniansson A, Ljungberg P, Rundgren A et al. The effect of a training programme for pensioners on condition, muscle strength and bone mineral density. Arch Gerontol Geriatr 1984;3:229–241.
3. Fiatarone M, Marks E, Ryan N et al. High intensity strength training in nonagerarians. JAMA 1990;263:3029–3034.
4. Foster V, Humme G, Bynness W et al. Endurance training for elderly women: Moderate vs. low intensity. J Gerontol 1989;44:M184–188.
5. King AC, Haskell W, Taylor B et al. Group vs. home-based exercise training in healthy older men and women. JAMA 1991;266:1535–1541.
6. Blumenthal JA, Emery CF, Madden DJ et al. Cardiovascular and behavioral effects of aerobic exercising training in healthy older men and women. J Gerontol 1989;44:M147–157.
7. Frontera WR, Meredith CN, O'Reilly KP et al. Strength conditioning in older men: Skeletal muscle hypertrophy and improved function. J Appl Physiol 1988;64:1038–1044.
8. Brown M, Holloszy JO. Effects of walking, jogging and cycling on strength, flexibility, speed and balance in 60- to 72-year-olds. Aging Clin Exp Res 1993;5:427–434.
9. Buchner DM, Cress ME, de Lateur BJ, Wagner EH. Variability in the effect of strength training on skeletal muscle strength in older adults. Facts Res Gerontol 1993;7:143–153.
10. Fiatarone MA, O'Neill EF, Ruan ND et al. Exercise training and nutritional supplementation for physical frailty in very elderly people. N Engl J Med 1994;330:1769–1775.
11. Nelson ME, Fiatarone MA, Morganti CM et al. Effects of high-intensity strength training on multiple risk factors for osteoporotic fractures. JAMA 1994;272:1909–1914.
12. McGinnis JM. The public health burden of a sedentary lifestyle. Med Sci Sports Exerc 1992;24:S196–200.
13. Washburn R, Smith K, Jette AM et al. The physical activity scale for the elderly (PASE): Development and evaluation. J Clin Epidemiol 1993;48:153–162.
14. Morris-Chatta R, Buchner DM, de Lateur BJ et al. Isokinetic testing of ankle strength in older adults: Assessment of inter-rater reliability and stability of strength over six months. Arch Phys Ther Rehabil 1994;in press.
15. McNair DM, Lorr M, Droppleman LF, eds. Profile of Mood States Manual. San Diego, CA: , 1981.
16. Kaye JM, Lawton MP, Gitlin LN et al. Older people's performance on the profile of mood states (POMS). Clin Gerontol 1988;7:35–56.
17. Curran SL, Andrykowski MA, Studts JL. Short form of the Profile of Mood States (POMS-SF): Psychometric information. Psychol Assessment 1995;7:80–83.
18. Stewart AL, King AC. Evaluating the efficacy of physical activity for influencing quality of life outcomes in older adults. Ann Behav Med 1991;13:108–116.
19. Stewart AL, King AC, Haskell WL. Endurance exercise and health-related quality of life in 50- 65-year-old adults. Gerontologist 1993;33:782–789.
20. Ware JE, Sherbourne CD. The MOS 36-item short-form health survey (SF-36): Conceptual framework and item selection. Med Care 1992;30:473–483.
21. Stewart AL, Ware JE Jr, eds. Measuring Functioning and Well-Being: The Medical Outcomes Study Approach. Durham & London: Duke University Press, 1992.
22. Knott M, Voss D. Proprioceptive Neuromuscular Facilitation—Patterns and Techniques, 2nd Ed. New York: Harper & Row, 1968.
23. Heislein D, Harris BA, Jette AM. A strength training program for postmenopausal women: A pilot study. Arch Phys Med & Rehabil 1994;75:196–204.
24. Cohen J. Statistical Power Analysis for the Behavioral Sciences. New York: Academic Press, 1977.
25. Wolfson L, Whipple R, Derby C et al. Short- and Long-Term Gains from Balance and Strength Training in Older Persons. Submitted for publication, 1995;1–22.
26. King AC, Taylor CB, Haskell WL. The effects of differing intensities and formats of twelve months of exercise training on psychological outcomes in older adults. Health Psychol 1993;12:292–300.
