# Supplementary material for: Effects of elastic band resistance training on the physical and mental health of elderly individuals: A mixed methods systematic review
Source: PLoS One. 2024 May 13;19(5):e0303372. doi: 10.1371/journal.pone.0303372 (PMC11090353; doi:10.1371/journal.pone.0303372)
Supplement: S1 File — (ZIP) [file pone.0303372.s001.zip › Supporting Information/Included study 63.pdf]

The Journal of Nursing Research ■ VOL. 30, NO. 5, OCTOBER 2022

DOI: https://doi.org/10.1097/jnr.0000000000000511

OPEN

Effectiveness of Elastic Band Exercises on the Functional Fitness of Older Adults in Long-Term Care Facilities

Yi-Lin SU[1] • Hsiao-Lien CHEN[2] • Shao-Li HAN[3] • Yueh-Kuei LIN[4] • Su-Yun LIN[5] • Chieh-Hsing LIU[6]*

ABSTRACT

Background: Population aging has caused a rise in the institutionalization, disability, and mortality rates of older adults worldwide. Older adults are able to engage in muscle training. Elastic band exercises can safely and effectively improve the upper and lower muscle strength and balance of older adults.

Purpose: This study was developed to examine the effects of a 3-month elastic band exercise program on the activities of daily living (ADLs), hand muscle strength, balance, and lower limb muscle strength of older adults living in institutional settings.

Methods: This was a randomized controlled trial. Sixty-one participants were randomly sampled from two long-term care facilities (LTCFs) in northern Taiwan (31 participants in the experimental group and 30 participants in the control group). Both groups underwent pretesting concurrently. The experimental group participated in 3 months of elastic band exercises, whereas the control group participated in the routine exercise program in their LTCFs. All of the participants were tested 1 and 3 months after the intervention.

Results: The average ADL, hand muscle strength, balance, and lower limb muscle strength scores of participants in the experimental group had improved significantly more than those of the control group at posttest (all *p*s < .05).

Conclusions/Implications for Practice: Elastic band exercises positively affect ADLs, hand muscle strength, balance, and lower limb muscle strength in older adults living in LTCFs. Moreover, the high benefit-to-cost ratio of these exercises helps lower the threshold of health promotion. We recommend including elastic band exercises in routine activities and designing different elastic band exercises for older adults at different proficiency levels. Furthermore, an elastic band exercise network should be established to improve the policy and implementation aspects of elastic band activities, raise awareness among community-dwelling and institutionalized older adults, and promote elastic band exercises to LTCFs nationwide.

Key Words:
elastic band exercise, functional fitness, older adult residents, long-term care facilities (LTCFs).

Introduction

The global population of people over the age of 65 years is growing faster than any other age group and exceeded the population under the age of 5 years for the first time in 2018. By 2050, one sixth (16%) of the world's population is expected be over 65 years old, which is significantly higher than that of one tenth (9%) reached in 2019. Moreover, the number of people over the age of 80 years is expected to more than double from 143 million in 2019 to 426 million by 2050 (Population Division, Department of Economic and Social Affairs, United Nations, 2019). Taiwan became an aging society in 1993 and an aged society in 2018. It is estimated that Taiwan will transition into a super-aged society by 2026. By 2065, roughly four of every 10 people in Taiwan will be over the age of 65 years and one in four older adults will be over 85 years old (National Development Council, 2018). Population aging is widely associated with increasing numbers of people admitted to institutions and significantly higher healthcare costs (de Meijer et al., 2012; Wübker et al., 2015).

Muscle strength is a key factor in maintaining balance. Muscle function begins to degrade at the age of 50 years and begins to rapidly deteriorate at the age of 65 years (Gallahue & Ozmun, 2012). Older adults who develop muscle weakness, lose their balance and fall, or become disabled rely on assistive devices such as wheelchairs and crutches (Karmarkar et al., 2011; Moncada & Mire, 2017). Aging leads to the degeneration of muscle mass and lower limb functions, which hinders older adults from performing

[1]MSN, RN, Doctoral Candidate, Department of Health Promotion and Health Education, National Taiwan Normal University, and Deputy Director, Department of Nursing, Sijhih Cathay General Hospital, Taiwan, ROC • [2]PhD, RN, Consultant, Department of Nursing, Cathay General Hospital, Taiwan, ROC • [3]PhD, MD, Attending Physician, Division of Rehabilitation Medicine, Sijhih Cathay General Hospital, Taiwan, ROC • [4]MSN, RN, Dean, Hang-An Senior Living, Taiwan, ROC • [5]BSN, RN, Director, Hang-An Senior Living, Taiwan, ROC • [6]PhD, Professor, Department of Health Promotion and Health Education, National Taiwan Normal University, Taiwan, ROC

Copyright © 2022 The Authors. Published by Wolters Kluwer Health, Inc.

This is an open access article distributed under the Creative Commons Attribution License 4.0 (CCBY), which permits unrestricted use, distribution, and reproduction in any medium, provided the original work is properly cited.

activities of daily living (ADLs) and impacts their quality of life. Furthermore, muscle degeneration increases the risk of falling, which can be fatal (Cheng et al., 2014). Research has identified falling as one of the major causes of disability in older adults (Boelens et al., 2013). Therefore, effectively strengthening the lower limb muscles in older adults and reducing the incidence of falls are presently the main focuses of disability prevention efforts for older adults.

Physical activity has been shown to benefit older adults with reduced mobility and reduce the risks of impairment, disability, and loss of independence (Denison et al., 2015; Tak et al., 2013). Physical activity can enhance muscle strength and gait balance, delaying the decline in ability to perform ADLs in long-term care facilities (LTCFs) and significantly improving the ability to respond to adversities such as falling, cardiovascular diseases, emotional disorders, quality of life, and depression (de Souto Barreto et al., 2016; Moncada & Mire, 2017). The benefits of resistance training include building skeletal muscle mass; enhancing muscle strength, flexibility, and dynamic balance; improving walking speed; and reducing age-related losses in muscle strength, muscle mass, and bone density. Resistance training concurrently improves physical functions, including sit-to-stand time, walking endurance and speed, balance, and fall avoidance, which improve quality of life and prolong independent living (Kuo et al., 2016; Mora & Valencia, 2018; Vafaeenasab et al., 2019).

Elastic bands, the primary tools used in low-intensity resistance training, are lightweight, compact when folded, and portable and may be used for training in a wide range of venues. Elastic bands are generally made from thin pieces of plastic and are available in a variety of specifications for different training needs. With elastic bands, resistance may be increased or decreased based on the user's muscle strength (Vafaeenasab et al., 2019). Studies have shown elastic band exercises to be low-impact and high-stability activities that improve cardiopulmonary and musculoskeletal function, muscle endurance, flexibility, lower body balance, and quality of life in older adults. Therefore, these exercises are safe and effective approaches to health enhancement (K. M. Chen, Tseng, Chang, et al., 2013; Park et al., 2015).

In recent years, many studies have examined the effects of elastic band exercises on healthy, subhealthy, and disabled older adults in various living arrangements. For example, a study conducted in Korea found that the use of elastic band exercises to tighten leg and hip joints significantly improved the gait stability and walking speed of older adult patients with stroke (Hwang et al., 2013). In Chou and Chen (2019), elastic band exercises were incorporated into a 4-week, lower-limb physical therapy regimen for older adult patients who had undergone total knee-joint replacement. The patients exercised with elastic bands 4 times per day, with a 2-hour break between each exercise session. The average 2- and 4-week postsurgery active/passive knee flexion and quadriceps strength were better in the experimental group than the control group. With regard to healthy, older adult patients, Vafaeenasab et al. (2019) evaluated 60 Iranian women aged 60–66 years who performed lower-limb elastic band exercises 3 times per week for 8 weeks, finding significantly improved static and dynamic balance, walking speed, and muscle strength at posttest. Kwon et al. (2019) implemented an 8-week elastic-band training intervention on 15 older women from community welfare centers for two 1-hour sessions per week, finding greatly improved skeletal muscle mass and significantly improved walking velocity, grip power, and body functions at posttest. With respect to LTCFs, K. M. Chen et al. (2015) devised a 6-month training program comprising 72 elastic band exercises for 114 wheelchair-bound residents. Participation in this program was found to significantly improve functional fitness and ability to perform ADLs. M. C. Chen et al. (2016) examined the effects of a 6-month elastic band exercise program on functional health and ability to perform ADLs in older adults experiencing cognitive dysfunction, finding significant and positive changes in ADL performance posttest. Elastic band exercises have also been negatively correlated with short-term morbidity and assisted-living needs in older adults. Thus, providing opportunities for exercise is critical for future self-care capacity and independence in older adults (Fahlman et al., 2011).

Because residents of LTCFs spend approximately 75% of their waking hours (i.e., more than 12 hours per day) engaged in sedentary activities, it is appropriate for this population to engage in activities that reduce the amount of sedentary time (Ikezoe et al., 2013; Keogh et al., 2015). On the basis of the known benefits of elastic band exercises, this study was designed to explore the effects of a 3-month elastic band exercise program on functional fitness in older adults living in LTCFs.

Methods

Study Design and Sampling

An experimental design was adopted in this study. Using a randomized sampling method, the participants were allocated into two groups based on the last digit of their national ID number, with those with an odd digit allocated to the experimental group and those with an even digit allocated to the control group. The participants were all recruited from two LTCFs in Northern Taiwan. The inclusion criteria were as follows: (a) older adults aged 65 years or older, (b) conscious with the ability to understand Mandarin or Taiwanese, (c) able to walk independently or with assistive devices, (d) determined by a physician trained in sports prescriptions to meet the criteria suggested in the American College of Sports Medicine Guidelines for Exercise Testing and Prescription (Pescatello et al., 2014), and (e) willing to participate in this study and sign the informed consent agreement. This study was approved by the institutional review board of Cathay General Hospital (CGH-P106042).

The data collection period was between April 20 and October 31, 2018. Both groups underwent pretesting in March. The experimental group participated in 3 months of elastic band exercises, whereas the control group

participated in the routine exercise program provided in their LTCFs. The minimum sample size was estimated using G*Power 3.1 statistical software. Referencing the research of K. M. Chen et al. (2015) on the effects of elastic band exercises on institutionalized older adults, *t* tests were used for analysis (effect size = .40, α = .05, power = .80). The software calculated that 48 participants would be necessary in total. After adding a 20% buffer to account for potential participant withdrawals, 31 participants were recruited for each group. One participant in the control group died from disease during the research period. Therefore, the experimental and control groups ultimately were composed of 31 and 30 participants, respectively, for a total sample size of 61 participants.

Intervention

Elastic band exercises

The elastic band exercises employed in this study were based on the exercises described by K. M. Chen, Tseng, Huang, et al. (2013) and were designed by a physiotherapist with 17 years of work experience. The participants participated in 40-minute sessions, 3 times per week for 3 months. The content of the program included five warm-up exercises (breathing exercise, neck stretches, chest stretches, back stretches, and leg stretches for 5 minutes), 10 aerobic exercises using light-resistance elastic bands (ankle dorsiflexion, knee flexion, hip abduction/adduction, side bends [lateral flexion], sitting flex [for trunk extension], elbow extension, biceps curl, shoulder external rotation, shoulder diagonal flexion [shoulder flexion], and lateral shoulder raises for 30 minutes), and three cooldown exercises (breathing and stretches for 5 minutes).

Instructors

The physiotherapist who designed the elastic band exercises served as the instructor for the program. The physiotherapist trained the exercise coordinators (nurses, nutritionists, social workers, and nurse aides). The training course lasted 60 minutes. During the course, the instructor highlighted the precautions, introduced the activities, and provided demonstrations. After the training course, the elastic band exercises were recorded on a video for use during the intervention.

Measurements

Measurement times

Using the instruments designed for this study, measurements were taken before the intervention, as well as 1 and 3 months after the intervention. The testers were a physiotherapist with 17 years of work experience and several instructors.

Measurement instruments

1. Anthropometric assessment

Measures of blood pressure (BP), pulse, body height, body weight, and body mass index (BMI, kg/m^2) were taken.

2. Activities of daily living

The Barthel index (BI) was used in this study to measure the dependency level of the participants' ADL functioning. The 10-item BI uses feeding, grooming, toileting, bathing, dressing, bowel control, and bladder control to assess self-care ability and also transfers, mobility, and climbing stairs to assess activity capacity. The total BI score range is 0–100, with 0–20 indicating totally dependent, 21–60 indicating heavily dependent, 61–90 indicating moderately dependent, 91–99 indicating mildly dependent, and 100 indicating total independence in functioning (K. M. Chen et al., 2015).

3. Grip power measurement

Grip power measurement was used in this study to measure hand muscle strength. A grip dynamometer (Baseline electronic hand dynamometer) was used for testing. In a sitting position, the participants were asked to relax their shoulders, let their arms naturally hang beside their bodies, flex their elbow to form a 90° angle with their upper arm, and straighten their wrist. They were then asked to grip the dynamometer and squeeze with maximum effort for 5 seconds. Participants completed one practice round before testing to ensure that they fully understood the test process. Two measurements were taken 15 seconds apart. The dynamometer was reset before each test. The average of the two readings was determined and presented as a force value in kilograms (Liao et al., 2014).

4. Berg Balance Scale

The Berg scale was used in this study to measure static balance. The participants were asked to perform 14 activities. The degree of execution for each activity was quantified to calculate balance. A score of 0–4 was allocated for each activity for a total possible scale score ranging between 0 and 56, with higher scores indicating better balance (Berg et al., 1995; Kornetti et al., 2004).

5. Thirty-second chair-stand test

The 30-second chair-stand test was used to measure muscle strength in the lower limbs. A chair with armrests (seat height of 40 cm) was used for testing. Participants were asked to sit in the center of the seat with their backs upright, feet naturally resting on the floor, arms crossed in front their chests, and eyes looking forward. When they heard the instruction "go," they were required to rise to a standing position at a comfortable speed, return to a sitting position once their knees were straight, and then repeat this action. The participants practiced this action several times. Once they correctly performed the action, they began the test. The number of times the participants were able to complete the action within the 30-second test period was recorded. The same chair was used for all tests, and similar environmental conditions were ensured (Millor et al., 2013).

Data Analysis

SAS 9.4 software (SAS Institute Inc., Cary, NC, USA) was used for data analysis, with descriptive statistics presented as mean, standard deviation, and percentages to illustrate the demographic profiles of the participants. Pearson χ^2 tests or independent *t* tests were conducted to validate intergroup differences in demographic data and intragroup differences in pretest and posttest data. A mixed-design, two-way analysis of variance was conducted to determine the interaction effects of the variables at different test times and between the two groups. A one-way analysis of variance was conducted to determine the main effects of the variables with significant interaction effects at different times in each group. Moreover, the differences between 1-month and 3-month posttest were analyzed. To further understand the group differences after 1 and 3 months, an analysis of covariance was conducted on the variables with significant interaction effects to determine the adjusted mean values of the pretest data. These values were adopted as the initial population variance. No data were missed in this study because the functional fitness of the participants was independently measured, and all tests were performed according to the plan. Different researchers were responsible for data collection, data transmission, and data analysis.

Results

Demographics of Participants

Sixty-one participants completed this study (experimental group, $n = 31$; control group, $n = 30$). The mean (*SD*) age of the participants was 79.25 (8.19) years; gender distribution was quite even, with 49.2% male and 50.8% female; and most used assistive devices (62.3%) and had chronic illnesses (65.7%). The top five chronic illnesses reported were as follows: hypertension (62.3%), diabetes (32.9%), heart disease (21.3%), dementia (21.3%), and stroke (14.8%). No significant difference in demographic data was identified between the two groups, and the data were normally distributed (all *p*s > .05).

Changes Between the Three Time Points in Each Group

Ability to perform ADLs, grip power, and balance improved significantly over time in the experimental group (all *p*s < .05), with no significant change at 1 month and a significant change at 3 months ($p < .05$). No significant change was identified in either group in terms of systolic BP ($F = 0.01$, $p = .990$), diastolic BP ($F = 0.04$, $p = .956$), BMI ($F = 0.02$, $p = .981$), or the 30-second chair-stand test ($F = 1.30$, $p = .278$; Tables 1 and 2). However, the systolic BP ($F = 5.00$, $p = .009$) of the control group deteriorated significantly (Table 3).

Group Comparisons at Different Time Points

Ability to perform ADLs, grip power, balance, and 30-second chair-stand test results were better in the experimental group than in the control group after the first month (all *p*s < .05; Table 4) and after 3 months (all *p*s < .05; Table 5). Thus, the experimental group results were all better than those of the control group. However, no significant differences in systolic BP, diastolic BP, or BMI were found between the groups.

Discussion

After performing the elastic-band exercise intervention for 3 months, the experimental group showed significant improvements in ADLs, hand muscle strength (grip power measurement), balance (Berg scale), and lower limb muscle strength (30-second chair-stand test). These findings are similar to

Table 1

Pretest and Posttest Changes in the Experimental Group (N = 31)

Variable	(1) Pretest		(2) Posttest 1		(3) Posttest 2		*F*	*p*	Post Hoc [a]
	M	*SD*	*M*	*SD*	*M*	*SD*			
Systolic BP (mmHg)	129.42	14.56	128.87	17.57	129.13	14.77	0.01	.990	–
Diastolic BP (mmHg)	73.61	11.43	72.77	12.54	73.00	10.19	0.04	.956	–
Body mass index (kg/m^2)	24.31	4.44	24.16	4.46	24.10	4.42	0.02	.981	–
ADL [b] (scores)	70.81	24.73	73.06	21.08	73.23	21.00	3.43	.037*	(1) < (2) [c]
Grip power measurement (kg)	12.45	7.94	12.58	7.16	13.90	7.96	3.22	.045*	(1) < (2)
Berg Balance Scale (scores)	13.81	3.79	13.87	4.74	14.06	4.80	4.54	.013*	(1) < (2)
30-second chair-stand test (numbers)	7.03	4.72	6.74	4.43	6.45	4.26	1.30	.278	–

Note. One-way repeated measures analyses of variance were performed to analyze the simple main effect of different time points in the experimental group. En dash means a post hoc analysis was not performed because of a nonsignificant *F* value. BP = blood pressure; ADL = activities of daily living.
[a]Bonferroni post hoc test (adjusting α levels for multiple tests). [b] Measured by the Barthel index. [c] Posttest 1 = 1 month; Posttest 2 = 3 months.
*$p < .05$.

Table 2
Pretest and Posttest 2 Significant Changes in the Experimental Group (N = 31)

Variable	Pretest		Posttest 2		95% CI	*F*	*p*
	M	*SD*	*M*	*SD*			
ADL (scores)	70.81	24.73	73.23	21.00	[−26.53, −1.21]	3.43	.037*
Grip power measurement (kg)	12.45	7.94	13.90	7.96	[−11.21, −0.27]		.045*
Berg Balance Scale (scores)	13.81	3.79	14.06	4.80	[−4.79, −0.63]	4.54	.013*

Note. ADL = activities of daily living.
$^{*}p < .05$.

those of M. C. Chen et al. (2016). The positive impact on ADLs of the exercise program may help delay aging-related physical deterioration, whereas the improvement in grip strength may significantly improve the ability to perform activities such as combing hair, dressing, and bathing. The findings of this study are similar to those of K. M. Chen, Tseng, Chang, et al. (2013). A previous study found grip strength in older adults to be positively correlated to physical function (Auyeung et al., 2014). Balance and lower limb strength have been associated with the safety of activities. Compared with the control group, the balance and lower limb strength of the experimental group was significantly improved after both the first and third months of the intervention, which also support the findings of previous studies (Idland et al., 2014; Vafaeenasab et al., 2019; Yu et al., 2013).

No significant change in BP was found in either group. Because BP is a dynamic parameter influenced by disease and drug control and nearly 70% of the participants in both groups had a history of chronic illness, this parameter is naturally difficult to control. However, improvements may be achieved using long-term training. The results of this study are similar to those of M. C. Chen et al. (2016). Elastic band exercises may be used to improve body composition, muscle mass, and physical function in older adults (Helbostad et al., 2004; Park et al., 2015). A slight but not significant postintervention improvement in BMI was found in the experimental group. This may be attributed to the metabolic functions of older adults. Moreover, exercise is not the only factor influencing BMI and body weight. Nutrition intake is also an important factor. Therefore, future research on improving older adults' body composition should take into account both physical activity and nutrition intake to improve the accuracy of results.

The elastic band exercise intervention has been shown to have positive effects on the functional fitness of older adults living in LTCFs. In this study, the elastic band exercise program had 3-month effects similar to those reported by K. M. Chen et al. (2015) and M. C. Chen et al. (2016) at 6 months after the intervention. However, the significant differences reported by these two studies in ADLs and hand muscle strength after 3 months differ from the results of this study. Reflecting the higher cost–benefit ratio of elastic band

Table 3
Pretest and Posttest Changes in the Control Group (N = 30)

Variable	(1) Pretest		(2) Posttest 1		(3) Posttest 2		*F*	*p*	Post Hoc [a]
	M	*SD*	*M*	*SD*	*M*	*SD*			
Systolic BP (mmHg)	136.60	20.95	126.03	15.70	123.07	15.04	5.00	.009**	(1) > (3) [b]
Diastolic BP (mmHg)	78.07	10.33	74.80	12.11	74.17	10.32	1.10	.339	–
Body mass index (kg/m^2)	23.25	3.76	28.96	17.58	29.01	18.00	1.53	.222	–
ADL [c] (scores)	61.17	23.22	56.83	26.24	55.33	26.52	0.43	.653	–
Grip power measurement (kg)	9.62	4.51	9.02	4.83	9.26	5.08	2.68	.075	–
Berg Balance Scale (scores)	11.33	3.82	10.67	4.87	11.00	4.73	0.16	.848	–
30-second chair-stand test (numbers)	5.80	4.54	4.40	4.53	3.87	4.70	1.31	.275	–

Note. One-way repeated measures analyses of variance were performed to analyze the simple main effect of different time points in the control group. En dash means a post hoc analysis was not performed because of a nonsignificant *F* value. BP = blood pressure; ADL = activities of daily living.
[a]Bonferroni post hoc test (adjusting α levels for multiple tests). [b] Activities of daily living, measured by the Barthel index. [c] Posttest 1 = 1 month; Posttest 2 = 3 months.
$^{**}p < .01$.

Table 4
Group Differences After 1 Month of the Intervention (N = 61)

Variable	Group	Adjusted *M*	SSE	*df*	MSE	*F*	*p*
Systolic BP (mmHg)	E	128.89	135.53	1	135.53	0.47	.494
	C	125.90					
Diastolic BP (mmHg)	E	72.74	67.89	1	67.89	0.43	.513
	C	74.85					
BMI (kg/m^2)	E	24.19	332.44	1	332.44	2.00	.163
	C	28.86					
ADL (scores)	E	73.23	3858.17	1	3858.17	6.91	.011*
	C	57.30					
Grip power measurement (kg)	E	12.42	165.39	1	165.39	4.47	.039*
	C	9.12					
Berg Balance Scale (scores)	E	14.06	167.30	1	167.30	7.21	.011*
	C	10.74					
30-second chair-stand test (numbers)	E	6.72	83.62	1	83.62	4.18	.046*
	C	4.46					

Note. SSE = sum of squares due to error; MSE = mean squared error; BP = blood pressure; BMI = body mass index; ADL = activities of daily living (measured by the Barthel index); E = experimental group; C = control group.
*$p < .05$.

exercises, related programs are conducive to lowering the threshold of health promotion. In addition, this study engaged a diverse study population and was not limited to wheelchair-bound older adults. This diversification should help promote elastic band exercises to older adults in the general population.

Conclusions/Implications for Practice

The benefits of elastic band exercises for older adults with health and physical limitations have been widely analyzed in recent years, with findings supporting these exercises as extremely beneficial. After 3 months of elastic band exercises, the results for ADLs, hand muscle strength, balance,

Table 5
Group Differences After 3 Months of the Intervention (N = 61)

Variable	Group	Adjusted *M*	SSE	*df*	MSE	*F*	*p*
Systolic BP (mmHg)	E	129.01	134.50	1	134.50	1.07	.921
	C	129.06					
Diastolic BP (mmHg)	E	72.98	16.84	1	16.84	0.16	.693
	C	74.04					
BMI (kg/m^2)	E	24.34	312.09	1	312.09	1.82	.183
	C	28.87					
ADL (scores)	E	73.06	4658.48	1	4658.48	7.98	.007**
	C	55.56					
Grip power measurement (kg)	E	13.85	304.85	1	304.85	6.96	.011*
	C	9.37					
Berg Balance Scale (scores)	E	13.79	113.81	1	113.81	4.94	.030*
	C	11.05					
30-second chair-stand test (numbers)	E	6.44	96.23	1	96.23	4.73	.034*
	C	3.92					

Note. Analysis of covariance was computed to test the group differences after 3 months of the study. SSE = sum of squares due to error; MSE = mean squared error; BP = blood pressure; BMI = body mass index; ADL = activities of daily living (measured by the Barthel index); E = experimental group; C = control group.
*$p < .05$. **$p < .01$.

and lower limb muscle strength were significantly improved in the experimental group. Elastic band exercises are accessible, affordable, and reliable. Therefore, we recommend that LTCFs incorporate elastic band exercises into their routine activities to enhance and maintain functional fitness in older adults, which helps delay the onset of disability. Elastic band exercises are particularly beneficial for improving muscle strength in the lower limbs and balance, reducing the likelihood of falls, which significantly threatens the lives of older adults. In addition, we recommend designing different elastic band exercises to meet the different levels and needs of older adults and to enhance the sense of achievement and participation.

In this study, we designed an elastic band exercise program for older adults. A physiotherapist was invited to train program coordinators and record an exercise video to promote the program. Moving forward, we should continue to develop the ideal elastic band program (with different intensity levels to meet the different physical statuses of older adults). Moreover, an elastic band exercise network should be created based on empirical research results to improve the policy and implementation aspects of elastic band activities, raise awareness among community-dwelling and institutionalized older adults, and promote elastic band exercises to LTCFs nationwide.

Acknowledgments

Sincere appreciation is directed by our group to the Cathay General Hospital, Taiwan, for funding this study (CGH-MR-B10607); to the directors and staff of the two participating long-term care institutions for their support and assistance; and to the 61 wonderful older adults for their generous participation.

Author Contributions

Study conception and design: HLC, YLS
Data collection: SLH, YKL, SYL
Data analysis and interpretation: YLS
Drafting of the article: YLS
Critical revision of the article: CHL

Received: November 10, 2020; Accepted: August 18, 2021
*Address correspondence to: Chieh-Hsing LIU, PhD, Department of Health Promotion and Health Education, National Taiwan Normal University, No. 162, Section 1, Heping E. Rd., Taipei City 106, Taiwan, ROC; E-mail: eline@cgh.org.tw
The authors declare no conflicts of interest.

Cite this article as:
Su, Y.-L., Chen, H.-L., Han, S.-L., Lin, Y.-K., Lin, S.-Y., & Liu, C.-H. (2022). Effectiveness of elastic band exercises on the functional fitness of older adults in long-term care facilities. *The Journal of Nursing Research, 30*(5), Article e235. https://doi.org/10.1097/jnr.0000000000000511

References

Auyeung, T. W., Lee, S. W. J., Leung, J., Kwok, T., & Woo, J. (2014). Age-associated decline of muscle mass, grip strength and gait speed: A 4-year longitudinal study of 3018 community-dwelling older Chinese. *Geriatrics & Gerontology International*, *14*, 76–84. https://doi.org/10.1111/ggi.12213

Berg, K., Wood-Dauphinee, S., & Williams, J. I. (1995). The Balance Scale: Reliability assessment with elderly residents and patients with an acute stroke. *Scandinavian Journal of Rehabilitation Medicine*, *27*(1), 27–36.

Boelens, C., Hekman, E. E. G., & Verkerke, G. J. (2013). Risk factors for falls of older citizens. *Technology and Health Care*, *21*(5), 521–533. https://doi.org/10.3233/THC-130748

Chen, K. M., Li, C. H., Chang, Y. H., Huang, H. T., & Cheng, Y. Y. (2015). An elastic band exercise program for older adults using wheelchairs in Taiwan nursing homes: A cluster randomized trial. *International Journal of Nursing Studies*, *52*(1), 30–38. https://doi.org/10.1016/j.ijnurstu.2014.06.005

Chen, K. M., Tseng, W. S., Chang, Y. H., Huang, H. T., & Li, C. H. (2013). Feasibility appraisal of an elastic band exercise program for older adults in wheelchairs. *Geriatric Nursing*, *34*(5), 373–376. https://doi.org/10.1016/j.gerinurse.2013.05.005

Chen, K. M., Tseng, W. S., Huang, H. T., & Li, C. H. (2013). Development and feasibility of a senior elastic band exercise program for aged adults: A descriptive evaluation survey. *Journal of Manipulative and Physiological Therapeutics*, *36*(8), 505–512. https://doi.org/10.1016/j.jmpt.2013.08.002

Chen, M. C., Chen, K. M., Chang, C. L., Chang, Y. H., Cheng, Y. Y., & Huang, H. T. (2016). Elastic band exercises improved activities of daily living and functional fitness of wheelchair-bound older adults with cognitive impairment: A cluster randomized controlled trial. *American Journal of Physical Medicine & Rehabilitation*, *95*(11), 789–799. https://doi.org/10.1097/PHM.0000000000000518

Cheng, I.-C., Wei, S.-H., Lieu, F.-K., & Tsai, P.-C. (2014). Biomechanical analysis of lower limb muscle power in elderly individuals. *Taiwan Journal of Physical Medicine and Rehabilitation*, *42*(3), 147–152. https://doi.org/10.6315/2014.42(3)02 (Original work published in Chinese)

Chou, L. N., & Chen, M. L. (2019). Effects of elastic band exercise on lower limb rehabilitation of elderly patients undergoing total knee arthroplasty. *Rehabilitation Nursing Journal*, *44*(1), 60–66. https://doi.org/10.1097/rnj.0000000000000109

de Meijer, C. A., Majer, I. M., Koopmanschap, M. A., & van Baal, P. H. (2012). Forecasting lifetime and aggregate long-term care spending: Accounting for changing disability patterns. *Medical Care*, *50*(8), 722–729. https://doi.org/10.1097/MLR.0b013e31824ebddc

de Souto Barreto, P., Morley, J. E., Chodzko-Zajko, W., Pitkala, K. H., Weening-Djiksterhuis, E., Rodriguez-Manas, L., Barbagallo, M., Rosendahl, E., Sinclair, A., Francesco Landi, F., Vellas, B., Rolland, Y., & Izquierdo, M. (2016). Recommendations on physical activity and exercise for older adults living in long-term care facilities: A taskforce report. *Journal of the American Medical Directors Association*, *17*(5), 381–392. https://doi.org/10.1016/j.jamda.2016.01.021

Denison, H. J., Cooper, C., AihieSayer, A., & Robinson, S. M. (2015). Prevention and optimal management of sarcopenia: A review of combined exercise and nutrition interventions to improve muscle outcomes in older people. *Clinical Interventions in Aging*, *10*, 859–869. https://doi.org/10.2147/CIA.S55842

Fahlman, M. M., McNevin, N., Boardley, D., Morgan, A., & Topp, R. (2011). Effects of resistance training on functional ability in

elderly individuals. *American Journal of Health Promotion*, *25*(4), 237–243. https://doi.org/10.4278/ajhp.081125-QUAN-292

Gallahue, D. L., & Ozmun, J. C. (2012). *Understanding motor development: Infant, children, adolescents, adults*. McGraw-Hill.

Helbostad, J. L., Sletvold, O., & Moe-Nilssen, R. (2004). Effects of home exercises and group training on functional abilities in home-dwelling older persons with mobility and balance problems. A randomized study. *Aging Clinical and Experimental Research*, *16*(2), 113–121. https://doi.org/10.1007/BF03324539

Hwang, Y. I., Yoo, W. G., & An, D. H. (2013). Effects of the elastic walking band on gait in stroke patients. *NeuroRehabilitation*, *32*(2), 317–322. https://doi.org/10.3233/NRE-130850

Idland, G., Sylliaas, H., Mengshoel, A. M., Pettersen, R., & Bergland, A. (2014). Progressive resistance training for community-dwelling women aged 90 or older; a single-subject experimental design. *Disability and Rehabilitation*, *36*(15), 1240–1248. https://doi.org/10.3109/09638288.2013.837969

Ikezoe, T., Asakawa, Y., Shima, H., Kishibuchi, K., & Ichihashi, N. (2013). Daytime physical activity patterns and physical fitness in institutionalized elderly women: An exploratory study. *Archives of Gerontology and Geriatrics*, *57*(2), 221–225. https://doi.org/10.1016/j.archger.2013.04.004

Karmarkar, A. M., Dicianno, B. E., Cooper, R., Collins, D. M., Matthews, J. T., Koontz, A., Teodorski, E. E., & Cooper, R. A. (2011). Demographic profile of older adults using wheeled mobility devices. *Journal of Aging Research*, *2011*, Article ID 560358. https://doi.org/10.4061/2011/560358

Keogh, J. W., Senior, H., Beller, E. M., & Henwood, T. (2015). Prevalence and risk factors for low habitual walking speed in nursing home residents: An observational study. *Archives of Physical Medicine and Rehabilitation*, *96*(11), 1993–1999. https://doi.org/10.1016/j.apmr.2015.06.021

Kornetti, D. L., Fritz, S. L., Chiu, Y. P., Light, K. E., & Velozo, C. A. (2004). Rating scale analysis of the Berg Balance Scale. *Archives of Physical Medicine and Rehabilitation*, *85*(7), 1128–1135. https://doi.org/10.1016/j.apmr.2003.11.019

Kuo, P.-L., Lin, C.-Y., Chang, L.-T., & Chu, P.-H. (2016). A practice model and its efficacy for resistance training in the elderly. *Journal of Gerontechnology and Service Management*, *4*(1), 117–128. https://doi.org/10.6283/JOCSG.2016.4.1.117 (Original work published in Chinese)

Kwon, I., Shin, C. H., & Kim, J. H. (2019). Associations between skeletal muscle mass, grip strength, and physical and cognitive functions in elderly women: Effect of exercise with resistive Theraband. *Journal of Exercise Nutrition & Biochemistry*, *23*(3), 50–55. https://doi.org/10.20463/jenb.2019.0023

Liao, W. C., Wang, C. H., Yu, S. Y., Chen, L. Y., & Wang, C. Y. (2014). Grip strength measurement in older adults in Taiwan: A comparison of three testing positions. *Australasian Journal on Ageing*, *33*(4), 278–282. https://doi.org/10.1111/ajag.12084

Millor, N., Lecumberri, P., Gómez, M., Martínez-Ramírez, A., & Izquierdo, M. (2013). An evaluation of the 30-s chair stand test in older adults: Frailty detection based on kinematic parameters from a single inertial unit. *Journal of Neuroengineering and Rehabilitation*, *10*(1), Article No. 86. https://doi.org/10.1186/1743-0003-10-86

Moncada, L. V. V., & Mire, L. G. (2017). Preventing falls in older persons. *American Family Physician*, *96*(4), 240–247.

Mora, J. C., & Valencia, W. M. (2018). Exercise and older adults. *Clinics in Geriatric Medicine*, *34*(1), 145–162. https://doi.org/10.1016/j.cger.2017.08.007

National Development Council. (2018). *Taiwan, ROC population estimate (2018–2065)*. https://pop-proj.ndc.gov.tw/chart.aspx?c=10&uid=66&pid=60 (Original work published in Chinese)

Park, S. Y., Kim, J. K., & Lee, S. A. (2015). The effects of a community-centered muscle strengthening exercise program using an elastic band on the physical abilities and quality of life of the rural elderly. *Journal of Physical Therapy Science*, *27*(7), 2061–2063. https://doi.org/10.1589/jpts.27.2061

Pescatello, L. S., Riebe, D., & Thompson, P. D. (Eds.). (2014). *ACSM's guidelines for exercise testing and prescription* (9th ed.). Lippincott Williams & Wilkins.

Population Division, Department of Economic and Social Affairs, United Nations. (2019). *World population ageing 2019: Highlights* (ST/ESA/SER.A/430). United Nations. https://digitallibrary.un.org/record/3846855/files/WorldPopulationAgeing2019-Highlights.pdf?ln=en

Tak, E., Kuiper, R., Chorus, A., & Hopman-Rock, M. (2013). Prevention of onset and progression of basic ADL disability by physical activity in community dwelling older adults: A meta-analysis. *Ageing Research Reviews*, *12*(1), 329–338. https://doi.org/10.1016/j.arr.2012.10.001

Vafaeenasab, M. R., Kuchakinejad Meybodi, N., Morowatisharifabad, M. A., Namayandeh, S. M., & Beigomi, A. (2019). The effect of lower limb resistance exercise with elastic band on balance, walking speed, and muscle strength in elderly women. *Elderly Health Journal*, *5*(1), 58–64. https://doi.org/10.18502/ehj.v5i1.1201

Wübker, A., Zwakhalen, S. M., Challis, D., Suhonen, R., Karlsson, S., Zabalegui, A., Soto, M., Saks, K., & Sauerland, D. (2015). Costs of care for people with dementia just before and after nursing home placement: Primary data from eight European countries. *The European Journal of Health Economics*, *16*(7), 689–707. https://doi.org/10.1007/s10198-014-0620-6

Yu, W., An, C., & Kang, H. (2013). Effects of resistance exercise using thera-band on balance of elderly adults: A randomized controlled trial. *Journal of Physical Therapy Science*, *25*(11), 1471–1473. https://doi.org/10.1589/jpts.25.1471
