# Supplementary material for: Effects of elastic band resistance training on the physical and mental health of elderly individuals: A mixed methods systematic review
Source: PLoS One. 2024 May 13;19(5):e0303372. doi: 10.1371/journal.pone.0303372 (PMC11090353; doi:10.1371/journal.pone.0303372)
Supplement: S1 File — (ZIP) [file pone.0303372.s001.zip › Supporting Information/Included study 61.pdf]

J. Phys. Ther. Sci.
25: 1471–1473, 2013

Effects of Resistance Exercise Using Thera-band on Balance of Elderly Adults: A Randomized Controlled Trial

Wonjong Yu, MSc, PT[1)], Changsik An, PhD, PT[2)*], Hyungkyu Kang, PhD, PT[3)]

[1)] *Department of Physical Therapy, Seonam University, Republic of Korea*
[2)] *Department of Physical Therapy, Eulji University: 553 Sanseong-daero, Sujeong-gu, Seongnam-si, Gyeonggi-do 461-713, Republic of Korea*
[3)] *Department of Physical Therapy, College of Natural Sciences, Kyungnam University, Republic of Korea*

Abstract. [Purpose] This study investigated the effects of resistance exercise using Thera-band on balance of elderly adults. [Methods] Subjects (age range, 60–70 years) were randomly assigned to an experimental (n=12) or control group (n=12). The experimental group performed stretching and resistance exercises, and the control group performed stretching exercises only. Before and after the 5-week intervention, the participants' static and dynamic balance were evaluated using the Berg Balance Scale, the Timed Up & Go Test, and the Tetrax Portable Multiple System (Tetrax Ltd., Ramat Gan, Israel) after 5 weeks. [Results] After the intervention, the values of the Tetrax in the weight distribution index with eyes open and that with eyes closed and the stability test index with eyes open were significantly lower in the resistance exercise group than in the control group, and the pre-test values were was significantly higher than the post-test values. However, there were no significant differences between groups in the values of the Berg Balance Scale, the Timed Up & Go Test, and the Tetrax stability test index with the eyes closed. [Conclusion] The findings of this study indicate that resistance exercise using the Thera-band is possible to improve the static and dynamic balance of elderly adults.
Key words: Stretching, Resistance exercise, Balance

(This article was submitted May 15, 2013, and was accepted Jun. 20, 2013)

INTRODUCTION

Functional ability and strength decreases with age because of a decrease in muscle fiber, degeneration of organs and tissues in the body, and a decrease in balance control ability due to degeneration of the visual and vestibular systems[1)]. Asymmetric posture and gait, and balance problems due to weakness in the lower extremities are responsible for a reduced ability to perform activities of daily living[2)]. Decreased balance causes disturbances while standing and during gait, through a decreases in the activities of daily living, and an increases the incidence of falls[3)].

One-third of the population aged ≥ 65 years experiences a fall at some time in their lives, the frequency of falls increases with age and 50% adults ≥ 80 years of age experience a fall each year[4)]. This increased risk of fallings in the elderly is attributed to the decreased balance and strength of the elderly. Therefore, lower-extremity resistance exercise is important for preventing falls in the elderly. Moreover, the relapse rate of falling was found to decrease sufficiently with home management[5)]. Therefore, it is important to devise a simple exercise program for improving the balance of the elderly adults. Stretching and resistance exercises have been shown to improve the balance ability in elderly adults[6)].

Resistance exercise using the Thera-band is simple and economical, and has safety advantages. It is generally used for rehabilitation purposes[7)], because training can be selected case by case through free control of the loading intensity. Several studies have reported that strengthening exercise using the Thera-band for the lower extremities improves balance ability. Therefore, strengthening exercise with a Thera-band in the body; thus, this is a suitable home-based exercise program for improving the balance in activities of daily living of elderly adults. We evaluated the effects of stretching and resistance exercises on the static and dynamic balance of elderly adults who had poor balance ability and did not perform exercises regularly.

SUBJECTS AND METHODS

The study subjects were 24 elderly adults (12 each in the experimental and control groups), 60–70 years of age, and who attended an exercise class for fall prevention in

*Corresponding author. Changsik An (e-mail: acsik@eulji.ac.kr)
©2013 The Society of Physical Therapy Science
This is an open-access article distributed under the terms of the Creative Commons Attribution Non-Commercial No Derivatives (by-nc-nd) License <http://creativecommons.org/licenses/by-nc-nd/3.0/>.

N country, in Korea. The general characteristics of the subjects are given in Table 1. The criteria for subject selection were as follows: age ≥ 60 years, no visual or vestibular disorders, no pain limiting exercise performance, ability to walk independently, no limitation of activities of daily living, and no regular exercise in the previous 6 months. The subjects included in this study were randomly divided into the experimental and control groups. They were given a complete explanation of the study and were asked to voluntarily sign the participation consent forms before beginning the exercises.

The experimental and control groups performed stretching exercises. Subjects in the experimental group also performed resistance exercises. The following stretching exercises were performed: knee flexion, hip flexion, hip abduction, and hip extension while standing; standing on tip toes; and knee extension while sitting. The resistance exercises performed using the Thera-band were as follows: knee flexion to extension while sitting, standing from sitting, knee extension to flexion while sitting, hip flexion to extension while standing, hip extension to flexion while standing, and hip adduction to abduction while standing. The exercise intensity in this study was gradually increased from mild to moderate. The exercises were performed 2 times for 1 set, 3 days per week for 5 weeks. A 1- to 2-s break was given after each motion, and a 2-min break was given after each set to minimize muscle fatigue.

The Berg Balance Scale (BBS) is a widely used clinical test used for determining an elderly adults' static and dynamic balance abilities, and it takes 15–20 min to complete. It is comprised a set of 14 simple balance-related tasks, ranging from standing from a sitting position to standing on 1 foot[8]. The Timed Up & Go Test (TUG) is a simple test used to evaluate an elderly adults' mobility, which requires both static and dynamic balance. It measures the time a subject takes to rise from a chair, walk 3 meters, turn around, walk back to the chair, and sit down[9]. The Tetrax Portable Multiple System (Tetrax Ltd., Ramat Gan, Israel) is a balance evaluation device that uses visual and vestibular feedback. The Tetrax system has 2 mobile force plates (12 × 30 cm), and postural disturbance is assessed by the change in weight on 4 points from which the stability test index and static balance index are computed. Measurement is performed in the standing position without speaking or moving for 32 s. The stability test index and weight distribution index are used for the analysis of static balance. Balance was measured on a stable surface with the eyes open and closed.

Statistical analyses were performed using SPSS version 18.0 software. The pre-intervention and post- intervention data were examined using the paired t-test within each group of subjects, and the independent *t*-test between the groups. The level of significance was chosen as 5% for all statistical analyses.

Table 1. General characteristics of the subjects

	Experimental (n=12)	Control (n=12)
Sex (male/female)	8/4	6/6
Age (years)	65.5 ± 4.5	65.0 ± 3.4
Height (cm)	160.5 ± 6.9	159.4 ± 7.6
Weight (kg)	64.3 ± 7.0	60.2 ± 5.7

Values are mean ± SD

RESULTS

Changes in the BBS and TUG scores of subjects are summarized in Table 2. The BBS and TUG were not significantly different between the experimental and control groups, and between each test. The weight distribution index with eyes open was significantly lower in the resistance exercise group than in the control group, and the pre-test value was significantly higher than the post-test value ($p<0.05$). The weight distribution index with eyes closed was significantly lower in the resistance exercise group than in the control group, and the pre-test value was significantly higher than the post-test value ($p<0.05$). The stability test index with eyes open was significantly lower in the resistance exercise group than in the control group, and the pre-test value was significantly higher than the post-test value ($p<0.05$). There were no significant differences in the stability test index with eyes closed between the groups, or between pre- and post-test.

DISCUSSION

This study evaluated the effects of stretching and resis-

Table 2. The comparison of dynamic and static balance

		Control (n=12)		Experimental (n=12)	
		Pre	Post	Pre	Post
TUG (s)		11.9 ± 1.2	11.7 ± 1.1	12.5 ± 1.1	12.4 ± 0.8
BBS (point)		50.1 ± 2.3	50.7 ± 1.9	50.3 ± 2.1	50.4 ± 1.1
EO	WDI	7.5 ± 3.3	8.8 ± 4.0	9.8 ± 5.8	4.3 ± 1.8[ab]
	ST	29.7 ± 7.5	26.9 ± 10.6	26.8 ± 8.5	19.2 ± 6.3[ab]
EC	WDI	9.2 ± 3.4	11.6 ± 3.6[a]	10.2 ± 2.9	7.9 ± 3.0[ab]
	ST	34.7± 12.2	32.1 ± 15.6	32.9 ± 10.0	36.8 ± 7.7

Values are mean ± SD, EO, eye open; EC, eye close; WDI, weight distribution index; ST, stability index

[a]Statistically significant difference between pre-test and post-test ($p<0.05$).

[b]Statistically significant difference between groups ($p<0.05$).

tance exercises on the static and dynamic balance of elderly adults who lacked exercise and had poor balance ability. Aging is a physical and psychological process that results in a decrease in balance ability[10, 11]. Generally, a continuous decrease in balance ability starts in adults in their forties because of muscle weakening, slow movement, increased fatigue causing gait problems, and functional defects in balance[12]. It can also be caused by decreased lower-body strength, coordination, flexibility, or proprioception[13]. Therefore, strengthening exercises are necessary for the recovery of balance ability. A previous study reported that resistance exercise using the Thera-band strengthened the lower limbs of elderly adults[14]. Therefore, this study evaluated the effects of easy and simple stretching and resistance exercises on the static and dynamic balance of elderly adults.

In an evaluation of dynamic balance using the TUG, it was reported that a time >14 s indicates a high risk of falling[9]. The TUG scores in this study were not significantly different between the groups, or between pre- and post-test. These results corroborate the findings of a previous study indicating that stretching does not improve balance ability of elderly adults[15]. However, these results do not agree with those of a previous study that showed that exercise using the Thera-band significantly improved the TUG score[16]. Our simple program consisting of stretching and resistance exercises was not sufficient to influence the TUG time, because of the program composition and the lack of time.

The BBS scores were not significant between the groups, or between pre- and post-test, indicating our simple tests. As a result, a single stretching and resistance exercise should be performed over the long term or be accompanied by other routines in order to have an effect on enhancing the dynamic balance performance of elderly subjects. The abnormally high results in the weight distribution index were related to orthopedic and neurological problems. A previous study reported that weight distribution indices of elderly adults were higher than those of young adults, because of poorer integration of visual and somatosensory information due to by aging-induced unstable balance ability[17].

The weight distribution index with eyes open was significantly lower in the resistance exercise group than in the control group, and the pre-test value was significantly higher than the post-test value. We conclude that an exercise program using elastic resistance improves the static balance of both limbs while maintaining a normal weight range and also improves suitable weight distribution. Our results support existing research claiming that regular training and increase of muscle strength enhance postural control by improving the sensory reactions in the nervous system[18]. We were considered that muscular training enhanced balance ability by improving the visual system and somatosensory senses.

The stability index is proportional to instability. The stability test index with eyes open was significantly lower in the resistance exercise group than in the control group, and the pre-test value was significantly higher than the post-test value. These results show that resistance exercise performed by elderly adults improved balance better than stretching. Alfieri et al.[19] reported that the center of gravity of subjects was maintained after trunk and lower-extremity strengthening exercises. Improved stability while performing resistance exercise in the standing position may cause overall strengthening.

Our study was limited by the relatively short period and the use of only 1 exercise among the numerous available methods. Thus, these findings cannot be generalized to all elderly adults. Therefore, additional research is necessary to determine the effects of balance improvement programs using various enjoyable exercises.

REFERENCES

1) Seidler RD, Bernard JA, Burutolu TB, et al.: Motor control and aging: links to age-related brain structural, functional, and biochemical effects. Neurosci Biobehav Rev, 2010, 34: 721–733. [Medline] [CrossRef]
2) O'Halloran AM, Finucane C, Savva GM, et al.: Sustained attention and frailty in the older adult population. J Gerontol B Psychol Sci Soc Sci, 2013 (in press). [Medline] [CrossRef]
3) Walker C, Brouwer BJ, Culham EG: Use of visual feedback in retraining balance following acute stroke. Phys Ther, 2000, 80: 886–895. [Medline]
4) Campbell AJ, Borrie MJ, Spears GF, et al.: Circumstances and consequences of falls experienced by a community population 70 years and over during a prospective study. Age Ageing, 1990, 19: 136–141. [Medline] [CrossRef]
5) Nikolaus T, Bach M: Preventing falls in community-dwelling frail older people using a home intervention team (HIT): results from the randomized Falls-HIT trial. J Am Geriatr Soc, 2003, 51: 300–305. [Medline] [CrossRef]
6) Bird ML, Hill K, Ball M, et al.: Effects of resistance- and flexibility-exercise interventions on balance and related measures in older adults. J Aging Phys Act, 2009, 17: 444–454. [Medline]
7) Cheema B, Abas H, Smith B, et al.: Randomized controlled trial of intradialytic resistance training to target muscle wasting in ESRD: the Progressive Exercise for Anabolism in Kidney Disease (PEAK) study. Am J Kidney Dis, 2007, 50: 574–584. [Medline] [CrossRef]
8) Berg KO, Maki BE, Williams JI, et al.: Clinical and laboratory measures of postural balance in an elderly population. Arch Phys Med Rehabil, 1992, 73: 1073–1080. [Medline]
9) Podsiadlo D, Richardson S: The timed "Up & Go": a test of basic functional mobility for frail elderly persons. J Am Geriatr Soc, 1991, 39: 142–148. [Medline]
10) Peterka RJ, Black FO: Age-related changes in human posture control: motor coordination tests. J Vestib Res, 1990, 1: 87–96. [Medline]
11) Wolfson L, Judge J, Whipple R, et al.: Strength is a major factor in balance, gait, and the occurrence of falls. J Gerontol A Biol Sci Med Sci, 1995, 50: 64–67. [Medline]
12) Balogun JA, Akindele KA, Nihinlola JO, et al.: Age-related changes in balance performance. Disabil Rehabil, 1994, 16: 58–62. [Medline] [CrossRef]
13) Kauffman TL: Geriatric Rehabilitation Manual. Philadelphia: Churchill Livingstone, 1999.
14) Gardner MM, Buchner DM, Robertson MC, et al.: Practical implementation of an exercise-based falls prevention programme. Age Ageing, 2001, 30: 77–83. [Medline] [CrossRef]
15) Alfieri FM, Riberto M, Abril-Carreres A, et al.: Effectiveness of an exercise program on postural control in frail older adults. Clin Interv Aging, 2012, 7: 593–598. [Medline] [CrossRef]
16) Beebe JA, Hines RW, McDaniel LT, et al.: An isokinetic training program for reducing falls in a community-dwelling older adult: a case report. J Geriatr Phys Ther, 2012 (in press).
17) Owen N, Leadbetter AG, Yardley L: Relationship between postural control and motion sickness in healthy subjects. Brain Res Bull, 1998, 47: 471–474. [Medline] [CrossRef]
18) Hu MH, Woollacott MH: Multisensory training of standing balance in older adults: I. Postural stability and one-leg stance balance. J Gerontol, 1994, 49: M52–M61. [Medline] [CrossRef]
19) Alfieri FM, Riberto M, Gatz LS, et al.: Comparison of multisensory and strength training for postural control in the elderly. Clin Interv Aging, 2012, 7: 119–125. [Medline] [CrossRef]
