# Supplementary material for: Effects of elastic band resistance training on the physical and mental health of elderly individuals: A mixed methods systematic review
Source: PLoS One. 2024 May 13;19(5):e0303372. doi: 10.1371/journal.pone.0303372 (PMC11090353; doi:10.1371/journal.pone.0303372)
Supplement: S1 File — (ZIP) [file pone.0303372.s001.zip › Supporting Information/Included study 49.pdf]

Experimental Gerontology 72 (2015) 99–108

Contents lists available at ScienceDirect

Experimental Gerontology

journal homepage: www.elsevier.com/locate/expgero

Effects of elastic band resistance training and nutritional supplementation on physical performance of institutionalised elderly — A randomized controlled trial

Stefan Oesen [a], Barbara Halper [a], Marlene Hofmann [a], Waltraud Jandrasits [a], Bernhard Franzke [a], Eva-Maria Strasser [c], Alexandra Graf [e], Harald Tschan [b], Norbert Bachl [b], Michael Quittan [c], Karl Heinz Wagner [a,d], Barbara Wessner [a,b,*]

[a] *Research Platform Active Ageing, University of Vienna, Vienna, Austria*
[b] *Centre for Sport Science and University Sports, University of Vienna, Auf der Schmelz 6, 1150 Vienna, Austria*
[c] *Karl Landsteiner Institute for Remobilization and Functional Health and Institute for Physical Medicine and Rehabilitation, Kaiser Franz Joseph Hospital, Social Medical Center South, Kundratstrasse 3, 1100 Vienna, Austria*
[d] *Faculty of Life Sciences, Department of Nutritional Science, University of Vienna, Althanstraße 14, 1090 Vienna, Austria*
[e] *Center for Medical Statistics, Informatics, and Intelligent Systems, Section for Medical Statistics, Medical University of Vienna, Spitalgasse 23, 1090 Vienna, Austria*

ARTICLE INFO

Article history:
Received 18 February 2015
Received in revised form 6 August 2015
Accepted 25 August 2015
Available online 2 September 2015

Section Editor: Christiaan Leeuwenburgh

Keywords:
Vienna Active Ageing Study (VAAS)
Low-intensity resistance exercise
Protein supplementation
Physical function
Seniors

ABSTRACT

Objectives: To evaluate the effects of elastic band resistance training in combination with nutrient supplementation on muscular strength and the ability to perform mobility-related activities of daily living in older adults living in retirement care facilities.
Design: Randomized controlled trial, with a 6-month intervention period.
Setting: A retirement care facility, Vienna, Austria.
Participants: One hundred and seventeen older adults (14 males (12%) and 103 females (88%)), aged 65 to 97 years (mean age: 82.8 ± 6.0), having a mini-mental state examination score ≥23 and no chronic diseases posing a medical contraindication to training therapy.
Intervention: Participants were randomly assigned, but stratified by sex, to one of three intervention groups: supervised resistance exercise training (RT), RT in combination with nutrient supplementation (RTS), or cognitive training group (CT). All interventions were performed two times a week for 6 months. RT was designed to train all major muscle groups using elastic bands. The nutrient supplement (rich in proteins, vitamin D, B2, B12) was distributed every morning as well as after each RT session.
Measurements: A battery of motor ability tests and functional test were performed prior to as well as following 3 months and finally after 6 months of intervention. These tests included isokinetic torque measurements of the knee extensors and flexors in concentric mode at 60 and 120°/s, isometric handgrip strength, senior arm-lifting test, chair stand test, maximum walking speed and a 6-minute walking test (6MWT).
Results: A repeated-measures ANOVA analysis revealed significant improvements in physical function of lower ($p = 0.002$) and upper extremities ($p = 0.006$) for RT and/or RTS in comparison to CT. For isokinetic measurements, 6MWT, and gait speed time effects ($p < 0.05$) were detected without any group × time interaction effects. Dropouts showed lower performance in chair stand test ($p = 0.012$), 6MWT ($p = 0.003$), and gait speed ($p = 0.013$) at baseline than that of the finishers of the study.
Conclusion: Six months of a low intensity resistance exercise using elastic bands and own body weight is safe and beneficial in improving functional performance of institutionalised older people. Multinutrient supplementation did not offer additional benefits to the effects of RT in improving muscular performance.
© 2015 Elsevier Inc. All rights reserved.

Abbreviations: ACSM, American College of Sports Medicine; AJLL, artificial joints of lower limbs; CT, cognitive training group; H, hamstrings; MMST, Mini Mental State Test; MNA, Mini Nutritional Assessment; PT, peak torque; RT, resistance training group; Q, quadriceps; RTS, resistance training plus nutritional supplement group; WA, walking aids; 6MWT, 6-minute walking test.

* Corresponding author.
E-mail addresses: stefan.oesen@univie.ac.at (S. Oesen), barbara.halper@univie.ac.at (B. Halper), marlene.hofmann@univie.ac.at (M. Hofmann), waltraudjandrasits@gmail.com (W. Jandrasits), bernhard.franzke@univie.ac.at (B. Franzke), eva-maria.strasser@wienkav.at (E.-M. Strasser), alexandra.graf@meduniwien.ac.at (A. Graf), harald.tschan@univie.ac.at (H. Tschan), norbert.bachl@univie.ac.at (N. Bachl), michael.quittan@wienkav.at (M. Quittan), karl-heinz.wagner@univie.ac.at (K.H. Wagner), barbara.wessner@univie.ac.at (B. Wessner).

http://dx.doi.org/10.1016/j.exger.2015.08.013
0531-5565/© 2015 Elsevier Inc. All rights reserved.

1. Introduction

Since the mid-twentieth century, the proportion of older persons in the total population has been rising continuously. Based on a recent projection of the United Nations globally the number of persons aged 60 years and over will more than double from 841 million people in 2013 to more than 2 billion in 2050, and by 2050 the share in older persons aged over 80 is expected to be more than three times than that of the present (United Nations, 2013). With increasing age progressive physiological changes occur that lead to a decrease in the function of various organ systems including the muscular system. Ageing-induced losses of skeletal muscle mass, strength, power and function typically occur from midlife onwards and are well documented (McGregor et al., 2014; von Haehling et al., 2010). The preservation of muscle strength and lean muscle mass are key components to perform activities of daily living and to maintain a satisfactory health-related quality of life (Geirsdottir et al., 2012).

Lifestyle choices such as regular physical exercise (both aerobic as well as strength exercises) and a balanced diet containing a sufficient amount of essential amino acids are generally regarded to be effective measures capable of preventing or counteracting age-related muscular decline (Pasiakos et al., 2015). Evidence suggests that intense resistance exercise interventions using free weights and exercise machines may reverse declines in muscle strength, lean muscle mass and functional capacity of older individuals (Romero-Arenas et al., 2013; Silva et al., 2014; Van Roie et al., 2013). The other side of the coin is that resistance exercise training applying high intensity modes and/or maximum strength assessments may pose a higher risk of injury or overuse especially to older people (Liu and Latham, 2010). Unlike traditional resistance exercise, elastic tubing provides adjustable resistance (the level of resistance varies according to rate and maximal stretch of the material such as greater stretch produces greater resistance) allowing a smooth progression (Martins et al., 2013). Not least because of this issue, elastic band exercise is frequently used for therapeutic training. It has been documented as a safe and effective strategy to enhance muscular performance finally improving the ability to perform functional tasks of older individuals (Colado and Triplett, 2008). Additional advantage of exercise training with elastic resistance bands includes high acceptance in older adults, its simplicity, versatility, portability, minimal space requirements and the comparatively low costs (Fahlman et al., 2011).

When resistance training is combined with protein supplementation, muscle protein synthesis is stimulated to an extent where the net protein balance (muscle protein synthesis minus muscle protein breakdown) becomes more positive than that with feeding alone (Phillips et al., 2005). However, there is evidence that the aged muscle is less responsive to the anabolic stimuli of amino acids and exercise, a state that is termed ‘anabolic resistance’ (Cuthbertson et al., 2005; Rennie, 2009). With respect to protein quality and quantity it has been hypothesized that protein supplements for the elderly should be rich in essential amino acids such as leucine in order to stimulate muscle protein synthesis in a way similar to that of younger adults (Yang et al., 2012). Besides proteins, vitamin D has been suggested to influence muscular performance, especially in subjects with low or deficient vitamin D levels (Halfon et al., 2015). Hence, a combination of resistance training with protein and vitamin D supplementation has been shown to be superior in preserving muscle mass during a weight loss programme in elderly than resistance training alone (Verreijen et al., 2015).

With these aspects in mind, the current study aimed to compare three different supervised training interventions (cognitive training, elastic band resistance training, and elastic band resistance training plus nutritional supplementation) on muscular strength and the ability to perform functional tests related to activities of daily living in older adults living in retirement homes.

2. Methods

2.1. Participants

Of 230 older men and women who were approached at 5 different retirement care facilities located in Vienna, 117 persons (103 females and 14 men, respectively) met the inclusion criteria and volunteered to participate in the study. Participants were required to be over the age of 65 years and physically cleared for medical stability for study participation. The exclusion criteria referred to recommendations of the American Heart Association (Williams et al., 2007) and included cognitive impairment (mini-mental state examination scores being <23) (Folstein et al., 1975), chronic diseases posing a contraindication to training therapy, serious cardiovascular diseases, diabetic retinopathy, and regular use of cortisone-containing drugs. In addition, subjects did not perform any regular resistance training. Before being included in the study, all subjects were comprehensively informed about the purpose, procedures, benefits and risks of and discomfort that might result from study participation. Written informed consent was obtained from all participants. The present study was conducted in accordance to the Austrian laws (including Doctors Act, CISA, Data Protection Act), the Declaration of Helsinki (as revised in Edinburgh 2000), and in analogous accordance with the ICH-GCP guidelines. The study has been approved by the ethics committee of the City of Vienna (EK-11-151-0811) and registered at ClinicalTrials.gov, NCT01775111.

2.2. Experimental design

To examine the effects of 6 months of supervised, progressive resistance exercise training with and without nutrient supplementation, participants of this controlled study were randomly assigned to one of three intervention groups. These intervention groups either performed resistance exercise training (RT), or the same resistance training protocol in combination with nutrient supplementation (RTS), or cognitive training (CT), the latter serving as the control group. Exercise intervention started immediately following baseline pre-testing which included anthropometric measurements, isokinetic torque measurements of knee extensors- and flexors in concentric mode, measurement of isometric handgrip strength of the dominant hand, and a battery of functional tests including upper body lift and reach test, chair stand test, maximum walking speed, and the 6 minute walking test (6MWT). In order to compare physiological adaptations, tests were repeated following 3 and 6 months of intervention, respectively. The tests were supervised by the same investigators. For each measurement time point the assessments were performed on two different days between 9:00 and 11:00 using identical equipment, the same subject positioning as well as standardized instructions. In addition, the order of testing remained the same on both testing days (day 1: Gait speed, chair stand test, 6MWT; day 2: Functional reach test, handgrip strength, isokinetic peak torque measurement (PT 60°/s Q., PT 60°/s H., PT 120°/s Q., PT 120°/s H.), arm-lifting test).

2.3. Resistance exercise intervention

The individualized exercise intervention using elastic band exercise as resistance (Thera-Band®, The Hygenic Corporation, Akron, OH, USA) was designed to train all major muscle groups (training volume and intensity were progressively increased) and was performed two times per week on non-consecutive days (separated by at least 48 h). Exercise training took place in small groups of not more than 10 participants, guided and closely supervised by appropriately trained and experienced sport scientists to ensure safety and compliance. Resistance exercise training was based on guidelines provided by the American College of Sports Medicine (ACSM) for older adults (Nelson et al., 2007). Each exercise session consisted of a general warm-up of 10 min, followed by a resistance training session (35–40 min) incorporating one to two

exercises (in a slow controlled manner) for each of the six muscle groups (legs, back, abdomen, chest, shoulder and arms), and was completed by a cool-down routine. Following an adaptation phase of 4 weeks using low external resistance (yellow Thera-Band®, 1 set of 15 repetitions per exercise with a higher resistance only if the subject was obviously unchallenged) exercise intensity was progressively increased by adapting the resistance of the elastic band (based on the Thera-Band® force-elongation table) (Page and Ellenbecker, 2011) from yellow to red and further to black. Additionally, the exercise volume was enhanced by increasing the number of sets from one to two. Rate of progression was based on individual improvements (band colour was changed if participant would have been able to perform two more repetitions in the second set and reported to be below seven on the OMNI Resistance for active muscle scale (0 extremely easy to 10 extremely hard)) (Lagally and Robertson, 2006). In the final training phase 56.9% of the participants had switched to the red elastic band while 43.1% even used the black one (specific information on the exercises are shown in Supplemental file 1).

2.4. Nutrient supplementation

In addition to resistance exercise training, subjects of RTS group received a nutrient supplement drink (FortiFit, NUTRICIA GmbH, Vienna, Austria) every morning after breakfast, as well as immediately after each training session. Each drink had a caloric value of 150 kcal and contained 20.7 g protein (3 g leucine, >10 g essential amino acids), 9.3 g carbohydrates, 3 g fat, vitamins (800 IU vitamin D, 2.9 mg vitamin B6, 3 μg vitamin B12) and minerals. A research dietician distributed the supplements and monitored adherence. Participants were instructed to maintain their regular food intake.

2.5. Cognitive training

The CT group served as sham-control to the resistance-exercise training groups mentioned above. Different from common control groups not receiving any treatment CT subjects participated in activities including cognitive tasks (memory training) and coordinative tasks (such as manual dexterity) twice weekly to provide a timely effort which was equal to those of the RT and RTS groups, respectively (Gatterer and Croy, 2004).

2.6. Anthropometric assessment

Body mass and body height were measured following standardized anthropometric procedures using a digital scale and a stadiometer during each testing session. Body mass was measured to the nearest 0.1 kg (SECA Model 877, Seca GmbH & Co. KG, Hamburg, Germany) and height was measured to the nearest 0.1 cm using a portable stadiometer (SECA Model 217, Seca GmbH & Co. KG, Hamburg, Germany). Body mass index was calculated as body mass relative to height in meters squared (kg/m^2).

2.7. Nutritional status

The Mini Nutritional Assessment (MNA) was used to assess nutritional status (Vellas et al., 1999). According to standard analyses subjects were classified as having a normal nutritional status (>24 points), as being at risk for malnutrition (17–23.5 points) or as being malnourished (<17 points).

2.8. Isokinetic dynamometry

Due to the relevance of the muscles of the lower extremities to activities of daily living, quadriceps and hamstring muscles were selected for muscle strength assessment. Isokinetic peak torque measurements of knee extensors and flexors were performed using a LIDO Multijoint II isokinetic loading dynamometer (Loredan Biomedical Inc., Sacramento, CA, USA). Participants were tested in a sitting position with their hip flexed at approximately 90° and subjects securely strapped to the seat of the chair using adjustable trunk and waist stabilisation belts. The anatomic axis of the knee rotation at the knee joint was aligned with the machine axis of rotation to insure similar movements for all participants. A thigh strap on the test leg was used to restrict any lateral movement at the knee, allowing only knee flexion and extension. Finally, a resistance pad was placed on the distal tibia, and the ankle of the leg being tested was securely strapped to the dynamometer lever arm. Limits for the knee extension/flexion movement (range of motion) was set between 30° and 80° of knee angle (with 0° being fully extended) with 80° of knee flexion as the starting position. Calibration and gravity correction procedures were performed before starting the test protocol. Following a general warm-up subjects were carefully familiarised with the equipment by performing submaximal trials of the extension–flexion movement with increasing intensity. Finally, two continuous maximal repetitions of knee extensors and knee flexors were performed concentrically at each angular velocity (60°/s and 120°/s) for peak torque recording. Between each of the recorded tests for a given angular velocity subjects rested for 2–3 min before performing the next maximum effort test. Participants were verbally encouraged to make a maximum effort throughout the whole range of motion. The highest extensor and flexor peak torques (Nm) of the two maximal trials at each angular velocity were chosen for analyses (Patterson and Spivey, 1992).

2.9. Handgrip strength

Handgrip strength of the right hand was measured to the nearest kilogram (kg) using a Jamar hand dynamometer (Sammsons Preston, Inc. Bolingbrook IL, USA). Subjects were seated with their elbow unsupported and bent at an angle of 90°. Prior data collection, the width of the dynamometer handle was adjusted to the individual hand size (with the middle phalanx resting on the inner handle) and participants performed two submaximal trials to get acquainted with the instrument and measurement procedure. Finally, participants were encouraged to perform a maximal contraction within approximately 4 to 5 s. After a rest of 60 s, participants were asked to perform a second trial. The highest score of maximum voluntary contraction was used for data analyses (Mijnarends et al., 2013).

2.10. Chair stand test

Chair rise performance is influenced by strength and power of the lower extremities. The mechanical power required to perform repetitive chair-rise movements has been shown to be an important indicator of mobility, fall risk, and functional debility in older individuals (Rikli and Jones, 2013). For this test the maximum number of completed cycles of unsupported chair rises (from a seated to a fully erected position (hip and knees straightened)) completed within 30 s was counted. A straight-back chair with a seat height of 46 cm was placed against a wall for support and safety purposes. Prior data collection and following a demonstration by the tester, participants performed a 2–3 repetition practice trial to familiarise with the technique. Participants were instructed to keep their arms crossed at the wrists and held them against the chest and place their feet flat on the floor approximately shoulder-width apart. For the test participants were encouraged to complete as many full stands as possible. The number of stands executed correctly within 30 s were counted by the tester and used for data analyses (incorrectly performed stands were not counted — the last stand of the end of the 30 s limit was counted only if the subject completed more than 50% of the upward motion).

2.11. Arm-lifting test

Functional performance for sustained upper extremity activity was assessed by performing a 30 s arm-lifting task of the dominant arm. Participants sitting on an armless straight-back chair were asked to repeatedly lift a dumbbell of 2 kg (females) and 4.5 kg (males) from waist level over a distance of 30 cm to shoulder level marked by the upper edge of a box. Following initial submaximal trials for familiarisation and a subsequent rest of 60 s, participants were encouraged to complete as many correct arm lifts as possible within a time limit of 30 s. The number of cycles correctly completed were counted and used for further analyses (King et al., 2000).

2.12. Gait speed

Physical mobility was assessed by measuring maximal walking time over a distance of 6 m on a flat surface on a hallway using an electronic photocell timing system (Brower Timing System, UT, USA). Participants were allowed to increase their speed within 2 m before the starting line and were advised not to decelerate before the finish line. Walking time was determined to the nearest 0.01 s while participants were allowed to use walking aids but must not run. Each participant had two consecutive trials separated by a rest period of 2 min. Gait speed was calculated by transforming walking time for 6 m to gait speed (m/s) (Mijnarends et al., 2013).

2.13. Six-minute walking test

As indicator of aerobic endurance the distance which could be covered within 6 min was recorded. Participants were instructed to walk alone as quickly as possible (without running) around a 30 m shuttle course as many times as possible within the time limit. Turning points were marked with cones. Participants were allowed to reduce the speed or rest if necessary and resume walking based on their performance abilities. Participants were informed about elapsed time after 3, 4, and 5 min. A measuring tape fixed parallel to the walking course allowed the tester who registered the number of shuttles to calculate the final distance, whereby the nearest meter was used for data analyses (Steffen et al., 2002).

2.14. Functional reach test

The functional reach test is a clinical assessment, testing the maximal safe standing forward reach (maintaining a base of support in the standing position) as a marker of postural stability in older adults. The test examines the distance a subject can extend the centre of gravity over the base of support, quantifying the boundaries of sway (Duncan et al., 1990). Before starting the test was described and demonstrated by the tester and participants could perform a trial to get familiarised with the movement execution. Participants were instructed to stand erect (feet shoulder length apart), next to a parallel wall without touching it, arms elevated to 90° of shoulder flexion, elbows extended and hands formed to fists. The assessor records the starting position at the third metacarpal head on a yardstick attached to the wall at acromion height. Then, without moving their feet off the ground, participants performed a hip flexion by moving their trunk forward and reaching as far as they could without taking a step. At this point, the tester again located the position of the third metacarpal head. Subsequently, the subject returned to the starting position and remained still for several seconds to clearly differentiate the end of the movement. Scores were determined by assessing the difference between the start and end position. Each participant performed the test three times with the best score (cm) used for further analyses.

2.15. Determination of physical activity

Physical activity was measured using accelerometers (GT1M, Actigraph, FL, USA) at baseline (pre-training) as well as following 6 months of intervention. Technical specifications characteristics of the Actigraph accelerometer and its validity have been published previously (Kelly et al., 2013). Participants were instructed to wear the device for 5 consecutive days including one weekend on the right hip for all waking hours, except during water-based activities. Data were considered valid if participants had activity counts for at least 10 h on 3 of these days, which is in line with minimum recommendations provided by Trost et al. (2005).

2.16. Statistical analyses

Data acquisition and data analyses were performed using commercial software with data files coded and anonymized. The number of participants chosen was based on power estimation with an alpha level set at 0.05 and a power of > 0.85. The power calculation software (G * Power3.1.0) (Faul et al., 2007) estimated the sample size to be 86 using isokinetic peak torque (concentric knee extension at an angular velocity of 60°/s.; range of motion 20°–80°) as primary endpoint. Previous studies in a similarly difficult collective show a high drop-out rate of about 35–40%. Therefore, a total of about 120 subjects were included in the study.

Repeated Measurement Analyses of Variance (ANOVA) was performed accounting for group (RT, RTS, and CT), time (pre-, mid-term, and post-intervention), and the interaction term between group and time defined as fixed factors and the test subject as random factor (Intention to treat analysis). Each of the endpoints demonstrating a significant result for time or the interaction term ("promising endpoints") in the primary model were further analysed with respect to the following influencing factors: age, gender, training attendance, mental state and residence. For the "promising endpoints" first univariate random effect mixed models were used (with subject as random factor) to study the influence of each of each of the influencing factors, on each of the promising endpoints. The primary model (accounting for group, time and interaction term) was then repeated for each of the promising endpoints, now additional accounting for the influence factors being significant in the univariate analyses (further on denoted by "final" model). Analyses were performed using Proc Mixed, SAS version 9.1 system and R release 2.15.1 (SAS Institute Cary NC, USA), respectively.

3. Results

3.1. Participant flow and baseline characteristics

Details of the participant flow are displayed in Fig. 1. Briefly, 117 elderly women and men who fulfilled the inclusion criteria were randomly assigned to RT (n = 41), RTS (n = 36), and CT (n = 40). A total of 22 participants (18.8%) withdrew their willingness to participate before or immediately following baseline tests. Twelve additional participants dropped-out during the intervention period of 6 months (CT (n = 5; 4.3%), RT (n = 3; 2.6%), RTS (n = 5; 4.3%)). None of the dropouts left the intervention study as a result of injuries or adverse responses to the study but because of other medical reasons not related to the intervention. A total of 82 (70.1%) participants (CT (n = 26; 65.0%), RT (n = 31; 75.6%), RTS (n = 5; 69.4%)) completed the study after 6 months.

Study participants did not differ in most of the baseline characteristics such as age, weight, height, body mass index, cognitive performance as measured by Mini Mental State (MMST), use of walking aids (WA) or the incidence of artificial joints of lower limbs (AJLL). Although the number of male participants was generally low (12.0%), the ratio between males and females did not differ between groups (p = 0.849). Interestingly, the Mini Nutritional Assessment (MNA) revealed significantly lower scores for the CT group as compared to both, the RT and RTS

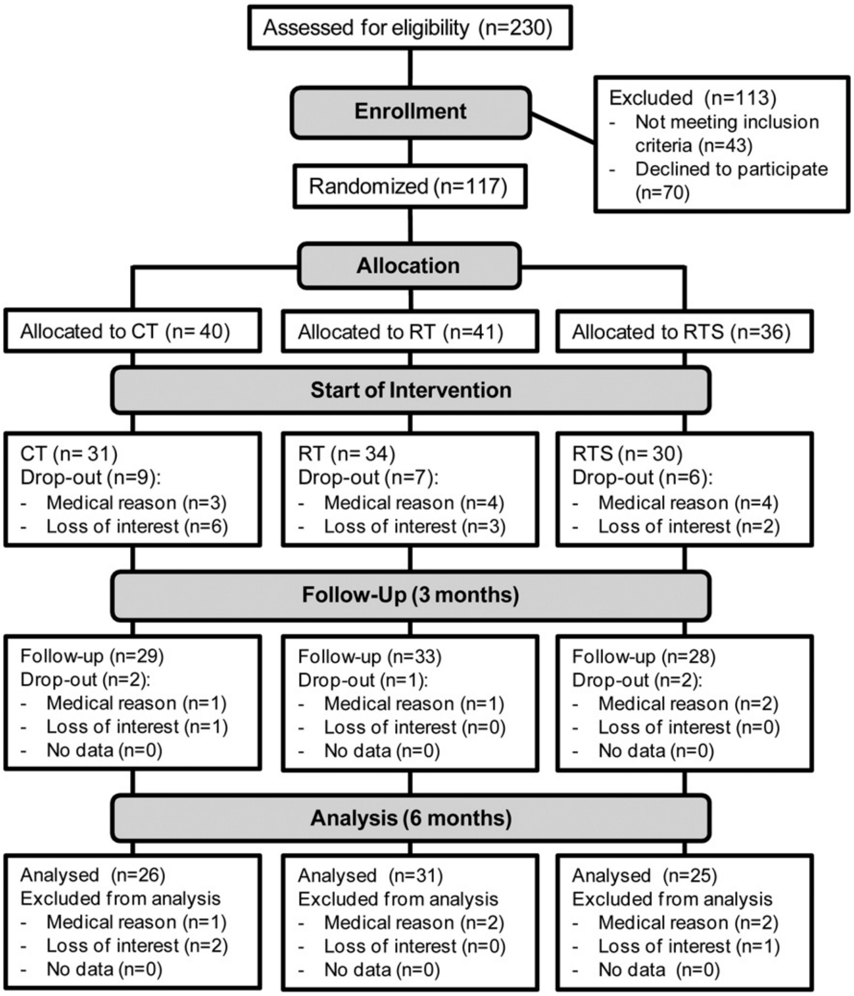

Fig. 1. Participant flow.

group (Table 1). None of the participants were classified as malnourished, 20.7% were at risk of malnutrition (32.3% of CT, 8.8% of RT, and 22.2% of RTS), whereas 79.3% showed a normal nutritional status (67.7% of CT, 91.2% of RT, and 77.8% of RTS; $p = 0.064$ for frequency distribution). When vitamin D deficiency was defined as having plasma values <25 nmol/l (Mosekilde, 2005), 20.0% of the study participants showed vitamin D deficiency (26.7% of CT, 14.3% of RT, and 20.0% of RTS; $p = 0.461$ for frequency distribution).

3.2. Muscle strength of the lower extremities (primary endpoint)

At baseline knee extensor and flexor peak torque did not differ between groups ($p > 0.05$). Peak torque significantly increased over time ($p < 0.05$) for all measurements. Post hoc analyses revealed an increase after 3 months for knee extensor peak torque at both velocities and for knee flexion at 120°/s ($p < 0.05$). After 6 months all measurements remained elevated with the exemption of knee extension at 60°/s which declined to baseline values. Interestingly, most of the elevations were reached at 3 months with only marginal changes between 3 and 6 months. No group ($p > 0.05$) or time × group interactions could be detected (Table 2).

3.3. Hand grip strength and functional performance (secondary endpoints)

Baseline measurements of handgrip strength ($p = 0.249$) and tests of functional performance such as chair stand test ($p = 0.815$), arm-lifting test ($p = 0.839$), gait speed ($p = 0.476$), 6MWT ($p = 0.911$), and functional reach test ($p = 0.142$) were similar among study groups.

With exception of handgrip strength and functional reach test, all measures of physical functioning including chair stand test, arm-lifting test, 6MWT, and gait speed significantly improved over time irrespective of group. While improvements for both, arm-lifting test ($p = 0.004$) and chair stand test ($p = 0.028$) could be detected at 3 months as well as after 6 months ($p < 0.001$), 6MWT ($p = 0.021$) and gait speed ($p = 0.004$) were increased only after 6 months.

Table 1
Baseline characteristics of participants.

	CT	RT	RTS	p-Value
Gender [females/males]	35/5	37/4	31/5	0.849
Age [years], n = 117	83.4 (±5.6)	83.0 (±5.5)	81.8 (±6.9)	0.498
Body mass [kg], n = 104	73.0 (±15.2)	72.5 (±9.7)	74.6 (±15.1)	0.802
Height [m], n = 104	1.59 (±0.08)	1.58 (±0.09)	1.58 (±0.07)	0.991
BMI [kg/m^2], n = 104	28.9 (±5.0)	28.9 (±3.7)	29.8 (±6.1)	0.713
MNA [−], n = 92	24.1 (±2.9)	26.3 (±1.8)**	25.9 (±2.2)**	<0.001
MMST [−], n = 117	27.8 (±1.7)	27.1 (±2.0)	27.6 (±1.9)	0.243
WA [number (%)]	11 (32.4%)	4 (11.4%)	6 (18.8%)	0.095
AJLL [number (%)]	3 (11.1%)	3 (9.1%)	1 (3.3%)	0.516

Values are shown as mean (± standard deviation). p-Values refer to differences between groups (Chi Square Test, one-factorial ANOVA).
CT (cognitive training group); RT (resistance training group); RTS (resistance training + supplement group); MNA (Mini Nutritional Assessment); MMST (Mini Mental State Test); WA (regular use of walking aids); AJLL (artificial joints of lower limbs).
** $p < 0.01$ versus CT.

Table 2
Isokinetic peak torque measurements.

Parameter	Group	Mean (± standard deviation)			Differences to baseline		p-values		
		Baseline	3 months	6 months	Δ (pre − 3 m)	Δ (pre − 6 m)	Time	Group	Time × group
PT 60°/s Q. rel. [Nm/kg]	CT	1.03 (±0.29)	1.09 (±0.34)*	0.97 (±0.38)	0.03 (±0.16)	−0.06 (±0.21)	**0.017**	0.476	0.230
	RT	0.98 (±0.32)	1.06 (±0.29)*	1.07 (±0.26)	0.07 (±0.18)	0.06 (±0.21)			
	RTS	1.07 (±0.42)	1.14 (±0.41)*	1.13 (±0.40)	0.06 (±0.16)	0.01 (±0.18)			
PT 60°/s H. rel. [Nm/kg]	CT	0.55 (±0.17)	0.61 (±0.15)	0.56 (±0.21)*	0.03 (±0.12)	0.00 (±0.12)	<**0.001**	0.552	0.061
	RT	0.51 (±0.16)	0.54 (±0.15)	0.60 (±0.14)*	0.02 (±0.10)	0.07 (±0.12)			
	RTS	0.59 (±0.22)	0.61 (±0.18)	0.66 (±0.21)*	0.02 (±0.13)	0.06 (±0.11)			
PT 120°/s Q. rel. [Nm/kg]	CT	0.82 (±0.27)	1.00 (±0.25)***	0.84 (±0.31)*	0.14 (±0.23)	0.01 (±0.19)	<**0.001**	0.794	0.683
	RT	0.82 (±0.26)	0.95 (±0.25)***	0.89 (±0.23)*	0.13 (±0.18)	0.05 (±0.17)			
	RTS	0.84 (±0.33)	0.98 (±0.37)***	0.95 (±0.34)*	0.15 (±0.20)	0.08 (±0.18)			
PT 120°/s H. rel. [Nm/kg]	CT	0.48 (±0.16)	0.63 (±0.15)***	0.56 (±0.23)***	0.12 (±0.16)	0.07 (±0.13)	<**0.001**	0.667	0.302
	RT	0.48 (±0.14)	0.55 (±0.14)***	0.55 (±0.15)***	0.07 (±0.10)	0.05 (±0.11)			
	RTS	0.52 (±0.19)	0.60 (±0.18)***	0.60 (±0.22)***	0.07 (±0.16)	0.06 (±0.11)			

Values are shown as mean (± standard deviation). p-Values refer to main effects of time and group as well as time×group interactions (repeated measurement ANOVA). Significant effects ($p < 0.05$) are shown in bold. CT (cognitive training group); RT (resistance training group); RTS (resistance training + supplement group); PT (peak torque);Q (Quadriceps, knee extension); H. (hamstrings, knee flexion); rel. (relative to body mass).
* $p < 0.05$ and *** $p < 0.001$ vs baseline for time effects.

An additional time × group effect could be detected for chair stand test ($p = 0.002$) and arm-lifting test ($p = 0.006$), indicating that changes over time were different between groups (Table 3). For the chair stand test a significant larger increase was found for RT (+17% at 3 and 6 months; $p < 0.001$) and RTS (+9% at 3 months and +15% at 6 months; $p = 0.007$) compared to CT. Interestingly, ameliorations in the arm-lift test were detected only in RTS (+19.1% at 3 months and +56% at 6 months; $p = 0.002$). No statistical differences could be observed between CT and RT ($p = 0.312$) as well as between RT and RTS ($p = 0.056$) (Fig. 2).

In order to determine whether a change in everyday physical activity could have influenced the primary and secondary outcomes, steps per day as marker of physical activity were determined by accelerometers. Valid data could be obtained from 61.5% of the participants at baseline but only from 36.7% after 6 months. Baseline measurements revealed an average of 4382 ± 2026 steps per day with no differences between intervention groups. Interestingly, after 6 months this measure decreased to 4197 ± 1696 steps per day ($p = 0.050$), while no group or time × group interaction effects could be detected.

3.4. Confounding factors

In general, a significant influence of age (with older participants having worse performance indices) was observed in several parameters, including peak torque measurements of knee extensors (120°/s) and flexors (60°/s and 120°/s) ($p < 0.001$), the 30-second arm lifting test ($p < 0.001$), and the 6MWT ($p = 0.016$), respectively. Besides exercise performance, older participants also had poorer scores in the mini mental state examination ($p = 0.019$).

Gender differences with higher values for male participants were observed in concentric isokinetic peak torque measurements of knee extensors (120°/s) and flexors (60°/s and 120°/s) ($p < 0.001$) and

Table 3
Handgrip strength and functional performance.

Parameter	Group	Mean (± standard deviation)			Differences to baseline		p-Value		
		Baseline	3 months	6 months	Δ (pre − 3 m)	Δ (pre − 6 m)	Time	Group	Time × group
Handgrip strength [kg]	CT	17.0 (±6.6)	17.3 (±8.2)	16.6 (±7.6)	0.8 (±2.6)	−0.3 (±2.4)	0.639	0.197	0.470
	RT	19.8 (±6.3)	20.2 (±5.6)	20.4 (±5.8)	−0.1 (±2.8)	0.4 (±2.7)			
	RTS	18.8 (±7.3)	19.2 (±7.0)	19.8 (±6.6)	0.2 (±2.5)	0.3 (±2.5)			
Arm-lifting test [repetitions]	CT	24 (±10)	23 (±9)**	23 (±10)***	2 (±9)	3 (±7)	<**0.001**	0.159	**0.006**
	RT	24 (±10)	28 (±10)**	30 (±12)***	2 (±4)	6 (±6)			
	RTS	22 (±11)	27 (±11)**	34 (±10)***	4 (±5)##	10 (±8)##			
Chair stand test [repetitions]	CT	11 (±5)	11 (±5)*	11 (±5)***	−1 (±2)	0 (±2)	<**0.001**	0.076	**0.002**
	RT	11 (±4)	14 (±4)*	15 (±4)***	2 (±3)###	2 (±3)###			
	RTS	12 (±5)	13 (±6)*	15 (±7)***	1 (±3)##	2 (±)##			
6MWT [m]	CT	369 (±111)	380 (±119)	389 (±113)*	−5 (±51)	9 (±59)	**0.006**	0.825	0.469
	RT	367 (±92)	394 (±89)	422 (±91)*	19 (±43)	23 (±45)			
	RTS	359 (±101)	393 (±158)	396 (±143)*	29 (±101)	29 (±98)			
Gait speed [m/s]	CT	1.48 (±0.57)	1.62 (±0.75)	1.62 (±0.67)**	0.05 (±0.36)	0.08 (±0.30)	**0.010**	0.800	0.296
	RT	1.49 (±0.39)	1.60 (±0.47)	1.71 (±0.52)**	0.11 (±0.31)	0.19 (±0.41)			
	RTS	1.50 (±0.70)	1.50 (±0.68)	1.62 (±0.83)**	−0.08 (±0.40)	0.00 (±0.46)			
Functional reach test [cm]	CT	27.3 (±5.3)	27.9 (±6.0)	27.5 (±6.5)	0.6 (±4.7)	0.8 (±5.9)	0.371	0.081	0.453
	RT	29.6 (±6.4)	29.9 (±7.9)	29.7 (±7.1)	0.3 (±6.1)	0.0 (±7.2)			
	RTS	30.4 (±7.1)	29.9 (±5.6)	32.6 (±6.8)	−0.5 (±6.3)	2.4 (±6.9)			
Physical activity [steps/d]	CT	4865 (±1576)	–	4713 (±1473)*	–	−152 (±1169)	**0.050**	0.818	0.380
	RT	4723 (±2271)	–	3988 (±1745)*	–	−735 (±1339)			
	RTS	4487 (±2236)	–	4194 (±1813)*	–	−293 (±862)			

Values are shown as mean (± standard deviation). p-Values refer to main effects of time and group as well as time×interaction effects (repeated measurement ANOVA). Significant values ($p < 0.05$) are shown in bold.
CT (cognitive training group); RT (resistance training group); RTS (resistance training + supplement group); 6MWT (Six-Minute Walking Test).
* $p < 0.05$, ** $p < 0.01$ and *** $p < 0.001$ vs baseline. ## $p < 0.01$ and ## $p < 0.001$ vs CT.

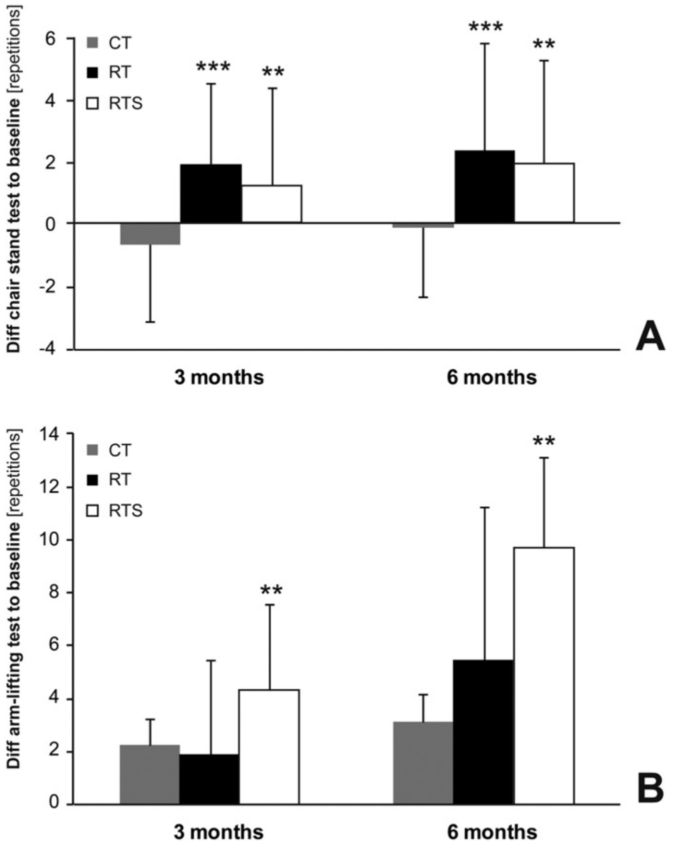

Fig. 2. Effects of resistance training ± nutritional supplement on functional performance. Values represent the difference in repetitions between baseline and 3 months or 6 months that could be performed within 30 s in the chair stand test (A) and the arm-lifting test (B); CT (control group); RT (resistance training group); RTS (resistance training + supplement group); **p < 0.01; ***p < 0.001 vs control group.

handgrip strength ($p < 0.001$). On average, men had larger walking speeds than women ($p = 0.003$) and reached higher distances in 6MWT ($p = 0.039$).

Attendance at the training sessions as calculated by the ratio between attended sessions and offered sessions in percent was 71% (±26.5%) with no significant differences between groups ($p > 0.05$). However, participants with higher attendance at the training sessions were more likely to have superior peak torque values of knee flexors at 120°/s ($p = 0.026$), and performed better in the 30 s chair stand test ($p = 0.030$) and the 6 MWT ($p = 0.019$), respectively.

MMST was affected by age ($p = 0.019$) but MMST did not influence any of the strength or functional performance parameters.

3.5. Baseline differences between finishers and drop-outs

As outlined in the participant flow (Fig. 1), in total nearly 30% of study participants quit the study directly after the baseline measurements (18.8%), between baseline and 3 months (4.3%) or between 3 and 6 months (6.8%). Interestingly, finishers of the intervention study had significantly higher performance indices already at the baseline tests in some of the functional parameters, namely chair stand test ($p = 0.012$), 6 MWT ($p = 0.005$) and gait speed ($p = 0.013$) compared to those subjects who dropped out from the intervention (Table 4).

4. Discussion

The main finding of the study was that six months of progressive elastic resistance training can be effective to enhance parameters of physical function such as number of repetitions in the chair stand test as well as the performance in the arm-lifting test even at old age.

Table 4
Baseline differences between finishers and drop-outs.

	Finishers	Drop-outs	p-Value
Participants [number (%)]	82 (70.1%)	35 (29.9%)	
Age [years]	82.4 (±6.2)	83.8 (±5.5)	0.221
MMST [−]	27.6 (±1.9)	27.3 (±2.0)	0.397
Chair stand test [repetitions]	12.0 (±4.7)	8.9 (±4.2)	**0.012**
6MWT [m]	379 (±91)	307 (±120)	**0.005**
Gait speed [m/s]	1.55 (±0.57)	1.19 (±0.44)	**0.013**
Functional reach test [cm]	28.9 (±6.4)	29.7 (±6.1)	0.694
Arm-lifting test [repetitions]	23.5 (±10.9)	23.8 (±4.7)	0.906
Handgrip strength [kg]	18.8 (±6.9)	17.2 (±5.5)	0.421

Values as mean (± standard deviation). p-Values refer to differences between the finisher and drop-outs (Chi Square-, independent t-test). Significant values ($p < 0.05$) are shown in bold. MMST: Mini Mental State Test; 6MWT: Six-Minute Walking Test.

However, this type of resistance training was not able to enhance isokinetic muscle torque of knee extensors and flexors, defined as primary outcome. This is different to many other resistance exercise studies with older adults using high-intensity programmes with free weights or weight machines for several weeks. There is evidence that training loads need to be high and of sufficient long duration if strength and muscle mass are to increase (Raymond et al., 2013; Silva et al., 2014; Steib et al., 2010). Intensities of 60–85% of the individual maximum voluntary strength have been recommended to increase muscle strength by increasing muscle mass and even higher intensities are required to improve the rate of force development in the elderly (Mayer et al., 2011). The study participants were instructed to choose the resistance (and colour) of the elastic band in a way that they should be able to perform two sets of exercises with a maximum of 15 repetitions per set and with a resting period of 30 to 60 s. Several equations have been developed to predict the one repetition maximum strength based on submaximal tests. Even if the accuracy of these equations is linked to specific exercises or populations, 15 repetitions as used in current study correspond to approximately 65% of the one repetition maximum (American College of Sports Medicine, 2009; Baechle and Earle, 2008). Therefore, the intensity chosen in our study can be defined as moderate (Steib et al., 2010). Although the participants were carefully supervised and encouraged, it cannot be excluded that some participants worked at a lower intensity potentially explaining the lack in strength gains. It might be of advantage in future studies to monitor elastic band exercises using stretch-sensors which have been shown to measure total and single repetition time-under-tension reliably (Skovdal Rathleff et al., 2013).

While higher training intensities seem to be superior to low or moderate intensities for improving maximal strength, low and moderate intensity programmes are frequently used especially for elderly subjects with functional limitations and with the presence of several comorbidities such as cardiovascular diseases (Liu and Latham, 2010; Pollock et al., 2000; Sousa et al., 2014). This is also the case for our study population which consisted of people who cannot be considered as thoroughly healthy as we included subjects with hypertension, hyperlipidemia, diabetes, osteoporosis, non-acute cardiac disesases and functional impairments (use of walking aids, dynapenia and sarcopenia) (Hofmann et al., 2015). One interesting outcome of our study has been that functional performance as assessed by chair stand and arm-lifting tests could be enhanced without improvements in muscle mass or strength. This is in line with the evidence demonstrating that high-intensity resistance training results in higher strength gains compared to low-intensity exercise while high intensities are not necessarily required to improve physical function (Raymond et al., 2013).

Specificity of the tests is an important issue when evaluating the effects of a training programme. We have used isokinetic dynamometry as the primary outcome as this method is considered to be the gold standard for assessing muscular strength (Gleeson and Mercer, 1996). Similar to other studies (Ostchega et al., 2004; Skelton et al., 1994) we observed good correlations of peak torques at both velocities with

handgrip strength ($r = 0.65–0.70$, $p < 0.001$), gait speed (0.53–0.54, $p < 0.001$), 6MWT (0.50–0.54, $p < 0.001$), and arm-lifting test ($r = 0.45–0.54$, $p < 0.001$). The correlations to chair stand test were somewhat lower (0.37–0.39, $p < 0.001$). In this study, two different angular velocities (60°/s, 120°/s) were used which can be considered as low to moderate also reflecting the training velocity of the elastic band resistance exercise training. We abstained from using higher velocities for assessment as institutionalised elderly might have been unable to reach the target velocity as shown previously (Lanza et al., 2003). Training with elastic bands includes concentric as well as eccentric components whereby the latter seem to be especially suitable for elderly because of the 'high-force–low-cost' manner of eccentric exercises (LaStayo et al., 2003; Skovdal Rathleff et al., 2013). Unfortunately eccentric muscle strength was not assessed in the current study making it impossible to judge the improvements in eccentric strength.

It has to be mentioned that the study population consisted of much more females (88%) than males (12%). While the proportion of males for community-dwelling men in Vienna is 34% at an age of 85 years (Statistik Austria, 2015), the proportion of males in the retirement homes is 19% in the age group of 80 to 89 years old (Bader et al., 2013) making our study population representative in terms of gender distribution of institutionalized elderly in Austria. However, with this small number of males it was not possible to conduct separate analyses for males and females. It is known that ageing affects muscle protein metabolism differently in men and women as the basal rate of muscle protein synthesis is approximately 30% higher in women than that in men, potentially explaining the slower loss of muscle mass in ageing females. On the other hand, exercise training doubles the basal muscle protein synthesis in men, whereas it is increased only by ~40% in females (Smith et al., 2008). However, it is interesting that trainability assessed by strength gains seem to be similar in older men and women (Lemmer et al., 2000; Peterson et al., 2010) making it unlikely (but not impossible) that larger or different improvements could have been observed in males.

One frequent limitation in many training intervention studies is the lack of a social activity for the control group that is of equal length and frequency to the exercising group. Therefore, we decided to control for social interactions by implementing a cognitive training for the control group. Interestingly, improvements over time were detected for many physical performance parameters such as isokinetic measurements, gait speed, and aerobic capacity as assessed by 6MWT regardless of groups indicating that also participants receiving only cognitive training improved in these parameters. In order to prove whether participants were changing their physical activity behaviour, we assessed steps per day at the beginning and at the end of the study as a measure for overall physical activity. Our data revealed that steps per day were generally low (only 10% met the recommendation of 7000 steps per day; Tudor-Locke et al., 2011) and decreased rather than increased in all groups. Therefore, it is unlikely that an increase in physical activity could have accounted for the observed improvements in performance of all groups. Future in-depth analyses will be conducted to assess whether time spent in different intensity zones or sedentary changed during the study period.

It has been shown that aerobic and/or strength training are able to improve cognitive function in patients with dementia (Bossers et al., 2015) and healthy older people (Nouchi et al., 2014). In addition, it is well known that difficulties with performance of functional activities may result from physical impairments but also from a cognitive decline (Fong et al., 2015). The question remains whether cognitive training interventions on the other hand could exert positive effects on physical performance. Usually, cognitive interventions have been developed to enhance memory and speed of processing, with the goal of maximizing current function and reducing the risk of cognitive decline in elderly populations (Acevedo and Loewenstein, 2007). However, a recent study has indicated that cognitive training might improve balance and gait in older adults known to have a history of falls (Smith-Ray et al., 2014) and longitudinal data from a large intervention trial have revealed that cognitive training ameliorates everyday functional outcomes in older adults demonstrating some transfer effects (Willis et al., 2006). Therefore, we cannot comprehensively exclude that cognitive training itself, although thought of as a control intervention, contributed to the ameliorations in gait speed and 6MWT.

Interestingly, protein supplementation was shown to be ineffective in this setting as we could not detect any differences between the trained only (RT) and the additionally supplemented group (RTS). We have assessed nutritional status by MNA and detected slightly lower scores in the cognitive group as compared to the other two intervention groups, whereby these scores did not change over time. In total, about 80% of the participants had a normal nutritional status. In previous publications we have reported that the nutritional supplementation resulted in an increased uptake of vitamin D and folic acid but not in protein being between 0.8 and 1.0 g/kg/day in the RTS group (Franzke et al., 2015a). Furthermore, plasma levels of vitamin B12 and folic acid in erythrocytes were enhanced due to supplementation (Franzke et al., 2015b). Therefore, we can confirm that the supplement was regularly consumed by the participants. There is an ongoing discussion about the optimal intake of proteins, especially in an elderly population as current recommendations for older adults range from 0.8 g/kg/day to 1.5 g/kg/day (Volkert and Sieber, 2011; Wolfe et al., 2008). It has been shown that both, protein quality and timing are important for efficiently enhancing muscle protein synthesis. In this study, we have used a protein supplement (20.7 g per serving) that contains whey protein enriched in leucine. Whey protein stimulates postprandial muscle protein accretion more effectively than do casein and casein hydrolysate due to its faster digestion and absorption kinetics and higher leucine content (Pennings et al., 2011). Increasing the proportion of leucine in a mixture of essential amino acids can reverse an attenuated response of muscle protein synthesis in the elderly (Katsanos et al., 2006). With respect to timing, the ingestion of dietary protein directly after resistance exercise increases post-exercise protein synthesis rates in young (Tipton et al., 2001) and old adults (Bukhari et al., 2015). In addition there is some evidence that providing enough protein to maximally stimulate muscle protein synthesis at each meal (breakfast, lunch, and dinner) induced the highest muscle protein synthesis in comparison to a skewed pattern with lower protein intakes for breakfast and lunch (Mamerow et al., 2014). Therefore, we assume that the pattern chosen in the current study with supplementation at breakfast and directly after the training sessions should be sufficient in order to additionally enhance muscle protein synthesis rate. However, the amount of protein could have been slightly too small as a recent dose–response study in elderly has shown that as little as 10 g of whey protein is able to prevent the significant decline in intramuscular branched chain amino acid levels, but only larger doses of whey (30 and 40 g) increase leucine level in skeletal muscle post-resistance exercise (D'Souza et al., 2014). However, even if a higher muscle protein synthesis can be achieved with protein supplementation post-exercise, this does not necessarily translate into improved muscle strength (Finger et al., 2015).

One of the most important secondary findings of our study is that those participants who quit the study before the final measurements were in a worse physical condition at baseline as measured by chair stand test, 6MWT and gait speed than those finalising the study after 6 months. We did not observe any side effects associated with the resistance exercise training or the nutritional supplementation. Reasons for dropouts mostly have been medical issues not related to the intervention but making a further participation difficult. This might be different to many other studies in older adults, pre-selecting a rather healthy and fit population of older adults but clinically relevant as a certain level of physical fitness seems to be needed before starting a group training programme.

In conclusion, six months of a moderate/low intensity resistance exercise using elastic bands and the own body weight has been shown to be beneficial in improving functional performance of institutionalised

older people with a mean age close to or even above the average life expectancy. Multinutrient supplementation did not offer additional benefits to the effects of RT in improving muscular performance.

Funding

This study was conducted with internal financial support from the University of Vienna, which enabled the establishment of the Research Platform "Active Ageing" as a means to foster interdisciplinary and innovative research.

Appendix A. Supplementary data

Supplementary data to this article can be found online at http://dx.doi.org/10.1016/j.exger.2015.08.013.

References

Acevedo, A., Loewenstein, D.A., 2007. Nonpharmacological cognitive interventions in aging and dementia. J. Geriatr. Psychiatry Neurol. 20, 239–249.
American College of Sports Medicine, 2009. American College of Sports Medicine position stand. Progression models in resistance training for healthy adults. Med. Sci. Sports Exerc. 41, 687–708.
Bader, E., Graumann, G., Hacker, M., Heissenberger, C., Honeder, M., Koblinger, N., Krb, W., Nutz, W., Redolfi, T., Scheidl, A., Schlöss, R., Stieb, H., Warmuth, H., 2013. Geschäftsbericht der Häuser zum Leben. http://www.kwp.at/pics/web/Dokumente/KWP_gesamt_web_low.pdf (2015-06-30).
Baechle, T.R., Earle, R.W., 2008. Essentials of strength training and conditioning. Human Kinetics, Champaign, IL.
Bossers, W.J., van der Woude, L.H., Boersma, F., Hortobagyi, T., Scherder, E.J., van Heuvelen, M.J., 2015. A 9-week aerobic and strength training program improves cognitive and motor function in patients with dementia: a randomized, controlled trial. Am. J. Geriatr. Psychiatr. http://dx.doi.org/10.1016/j.jagp.2014.12.191.
Bukhari, S.S., Phillips, B.E., Wilkinson, D.J., Limb, M.C., Rankin, D., Mitchell, W.K., Kobayashi, H., Greenhaff, P.L., Smith, K., Atherton, P.J., 2015. Intake of low-dose leucine-rich essential amino acids stimulates muscle anabolism equivalently to bolus whey protein in older women at rest and after exercise. Am. J. Physiol. Endocrinol. Metab. 308, E1056–E1065.
Colado, J.C., Triplett, N.T., 2008. Effects of a short-term resistance program using elastic bands versus weight machines for sedentary middle-aged women. J. Strength Cond. Res. 22, 1441–1448.
Cuthbertson, D., Smith, K., Babraj, J., Leese, G., Waddell, T., Atherton, P., Wackerhage, H., Taylor, P.M., Rennie, M.J., 2005. Anabolic signaling deficits underlie amino acid resistance of wasting, aging muscle. FASEB J. 19, 422–424.
D'Souza, R.F., Marworth, J.F., Figueiredo, V.C., Della Gatta, P.A., Petersen, A.C., Mitchell, C.J., Cameron-Smith, D., 2014. Dose-dependent increases in p70S6K phosphorylation and intramuscular branched-chain amino acids in older men following resistance exercise and protein intake. Phys. Rep. 2.
Duncan, P.W., Weiner, D.K., Chandler, J., Studenski, S., 1990. Functional reach: a new clinical measure of balance. J. Gerontol. 45, M192–M197.
Fahlman, M.M., McNevin, N., Boardley, D., Morgan, A., Topp, R., 2011. Effects of resistance training on functional ability in elderly individuals. Am. J. Health Promot. 25, 237–243.
Faul, F., Erdfelder, E., Lang, A.G., Buchner, A., 2007. G*Power 3: a flexible statistical power analysis program for the social, behavioral, and biomedical sciences. Behav. Res. Methods 39, 175–191.
Finger, D., Goltz, F.R., Umpierre, D., Meyer, E., Rosa, L.H., Schneider, C.D., 2015. Effects of protein supplementation in older adults undergoing resistance training: a systematic review and meta-analysis. Sports Med. 45, 245–255.
Folstein, M.F., Folstein, S.E., McHugh, P.R., 1975. "Mini-mental state". A practical method for grading the cognitive state of patients for the clinician. J. Psychiatr. Res. 12, 189–198.
Fong, T.G., Gleason, L.J., Wong, B., Habtemariam, D., Jones, R.N., Schmitt, E.M., de Rooij, S.E., Saczynski, J.S., Gross, A.L., Bean, J.F., Brown, C.J., Fick, D.M., Gruber-Baldini, A.L., O'Connor, M., Tabloski, P.A., Marcantonio, E.R., Inouye, S.K., 2015. Cognitive and Physical Demands of Activities of Daily Living In Older. Validation of Expert Panel Ratings. PM R, Adults http://dx.doi.org/10.1016/j.pmrj.2015.01.018.
Franzke, B., Halper, B., Hofmann, M., Oesen, S., Jandrasits, W., Baierl, A., Tosevska, A., Strasser, E.M., Wessner, B., Wagner, K.H., Vienna Active Ageing Study Group, 2015a. The impact of six months strength training, nutritional supplementation or cognitive training on DNA damage in institutionalised elderly. Mutagenesis 30, 147–153.
Franzke, B., Halper, B., Hofmann, M., Oesen, S., Pierson, B., Cremer, A., Bacher, E., Fuchs, B., Baierl, A., Tosevska, A., Strasser, E.M., Wessner, B., Wagner, K.H., Vienna Active Ageing Study, G, 2015b. The effect of six months of elastic band resistance training, nutritional supplementation or cognitive training on chromosomal damage in institutionalized elderly. Exp. Gerontol. 65, 16–22.
Gatterer, G., Croy, A., 2004. Geistig fit ins Alter 1: Neue Gedächtnisübungen für ältere Menschen. Springer, Vienna.
Geirsdottir, O.G., Arnarson, A., Briem, K., Ramel, A., Tomasson, K., Jonsson, P.V., Thorsdottir, I., 2012. Physical function predicts improvement in quality of life in elderly Icelanders after 12 weeks of resistance exercise. J. Nutr. Health Aging 16, 62–66.
Gleeson, N.P., Mercer, T.H., 1996. The utility of isokinetic dynamometry in the assessment of human muscle function. Sports Med. 21, 18–34.
Halfon, M., Phan, O., Teta, D., 2015. Vitamin D: a review on its effects on muscle strength, the risk of fall, and frailty. Biomed. Res. Int. 2015, 953241.
Hofmann, M., Halper, B., Oesen, S., Franzke, B., Stuparits, P., Tschan, H., Bachl, N., Strasser, E.M., Quittan, M., Ploder, M., Wagner, K.H., Wessner, B., 2015. Serum concentrations of insulin-like growth factor-1, members of the TGF-beta superfamily and follistatin do not reflect different stages of dynapenia and sarcopenia in elderly women. Exp. Gerontol. 64, 35–45.
Katsanos, C.S., Kobayashi, H., Sheffield-Moore, M., Aarsland, A., Wolfe, R.R., 2006. A high proportion of leucine is required for optimal stimulation of the rate of muscle protein synthesis by essential amino acids in the elderly. Am. J. Physiol. Endocrinol. Metab. 291, E381–E387.
Kelly, L.A., McMillan, D.G., Anderson, A., Fippinger, M., Fillerup, G., Rider, J., 2013. Validity of actigraphs uniaxial and triaxial accelerometers for assessment of physical activity in adults in laboratory conditions. BMC Med. Phys. 13, 5.
King, A.C., Pruitt, L.A., Phillips, W., Oka, R., Rodenburg, A., Haskell, W.L., 2000. Comparative effects of two physical activity programs on measured and perceived physical functioning and other health-related quality of life outcomes in older adults. J. Gerontol. A Biol. Sci. Med. Sci. 55, M74–M83.
Lagally, K.M., Robertson, R.J., 2006. Construct validity of the OMNI resistance exercise scale. J. Strength Cond. Res. 20, 252–256.
Lanza, I.R., Towse, T.F., Caldwell, G.E., Wigmore, D.M., Kent-Braun, J.A., 2003. Effects of age on human muscle torque, velocity, and power in two muscle groups. J. Appl. Physiol. 95, 2361–2369.
LaStayo, P.C., Ewy, G.A., Pierotti, D.D., Johns, R.K., Lindstedt, S., 2003. The positive effects of negative work: increased muscle strength and decreased fall risk in a frail elderly population. J. Gerontol. A Biol. Sci. Med. Sci. 58, M419–M424.
Lemmer, J.T., Hurlbut, D.E., Martel, G.F., Tracy, B.L., Ivey, F.M., Metter, E.J., Fozard, J.L., Fleg, J.L., Hurley, B.F., 2000. Age and gender responses to strength training and detraining. Med. Sci. Sports Exerc. 32, 1505–1512.
Liu, C.J., Latham, N., 2010. Adverse events reported in progressive resistance strength training trials in older adults: 2 sides of a coin. Arch. Phys. Med. Rehabil. 91, 1471–1473.
Mamerow, M.M., Mettler, J.A., English, K.L., Casperson, S.L., Arentson-Lantz, E., Sheffield-Moore, M., Layman, D.K., Paddon-Jones, D., 2014. Dietary protein distribution positively influences 24-h muscle protein synthesis in healthy adults. J. Nutr. 144, 876–880.
Martins, W.R., de Oliveira, R.J., Carvalho, R.S., de Oliveira Damasceno, V., da Silva, V.Z., Silva, M.S., 2013. Elastic resistance training to increase muscle strength in elderly: a systematic review with meta-analysis. Arch. Gerontol. Geriatr. 57, 8–15.
Mayer, F., Scharhag-Rosenberger, F., Carlsohn, A., Cassel, M., Muller, S., Scharhag, J., 2011. The intensity and effects of strength training in the elderly. Dtsch. Arztebl. Int. 108, 359–364.
McGregor, R.A., Cameron-Smith, D., Poppitt, S.D., 2014. It is not just muscle mass: a review of muscle quality, composition and metabolism during ageing as determinants of muscle function and mobility in later life. Longev Healthspan 3, 9.
Mijnarends, D.M., Meijers, J.M., Halfens, R.J., ter Borg, S., Luiking, Y.C., Verlaan, S., Schoberer, D., Cruz Jentoft, A.J., van Loon, L.J., Schols, J.M., 2013. Validity and reliability of tools to measure muscle mass, strength, and physical performance in community-dwelling older people: a systematic review. J. Am. Med. Dir. Assoc. 14, 170–178.
Mosekilde, L., 2005. Vitamin D and the elderly. Clin. Endocrinol. 62, 265–281.
Nelson, M.E., Rejeski, W.J., Blair, S.N., Duncan, P.W., Judge, J.O., King, A.C., Macera, C.A., Castaneda-Sceppa, C., 2007. Physical activity and public health in older adults: recommendation from the American College of Sports Medicine and the American Heart Association. Med. Sci. Sports Exerc. 39, 1435–1445.
Nouchi, R., Taki, Y., Takeuchi, H., Sekiguchi, A., Hashizume, H., Nozawa, T., Nouchi, H., Kawashima, R., 2014. Four weeks of combination exercise training improved executive functions, episodic memory, and processing speed in healthy elderly people: evidence from a randomized controlled trial. Age (Dordr.) 36, 787–799.
Ostchega, Y., Dillon, C.F., Lindle, R., Carroll, M., Hurley, B.F., 2004. Isokinetic leg muscle strength in older americans and its relationship to a standardized walk test: data from the national health and nutrition examination survey 1999–2000. J. Am. Geriatr. Soc. 52, 977–982.
Page, P., Ellenbecker, T.S., 2011. Strength Band Training. Human Kinetics, Leeds.
Pasiakos, S.M., McLellan, T.M., Lieberman, H.R., 2015. The effects of protein supplements on muscle mass, strength, and aerobic and anaerobic power in healthy adults: a systematic review. Sports Med. 45, 111–131.
Patterson, L.A., Spivey, W.E., 1992. Validity and reliability of the LIDO active isokinetic system. J. Orthop. Sports Phys. Ther. 15, 32–36.
Pennings, B., Boirie, Y., Senden, J.M., Gijsen, A.P., Kuipers, H., van Loon, L.J., 2011. Whey protein stimulates postprandial muscle protein accretion more effectively than do casein and casein hydrolysate in older men. Am. J. Clin. Nutr. 93, 997–1005.
Peterson, M.D., Rhea, M.R., Sen, A., Gordon, P.M., 2010. Resistance exercise for muscular strength in older adults: a meta-analysis. Ageing Res. Rev. 9, 226–237.
Phillips, S.M., Hartman, J.W., Wilkinson, S.B., 2005. Dietary protein to support anabolism with resistance exercise in young men. J. Am. Coll. Nutr. 24, 134S–139S.
Pollock, M.L., Franklin, B.A., Balady, G.J., Chaitman, B.L., Fleg, J.L., Fletcher, B., Limacher, M., Pina, I.L., Stein, R.A., Williams, M., Bazzarre, T., 2000. AHA Science Advisory. Resistance exercise in individuals with and without cardiovascular disease: benefits, rationale, safety, and prescription: An advisory from the Committee on Exercise, Rehabilitation, and Prevention, Council on Clinical Cardiology, American Heart Association; Position paper endorsed by the American College of Sports Medicine. Circulation 101, 828–833.
Raymond, M.J., Bramley-Tzerefos, R.E., Jeffs, K.J., Winter, A., Holland, A.E., 2013. Systematic review of high-intensity progressive resistance strength training of the lower limb

compared with other intensities of strength training in older adults. Arch. Phys. Med. Rehabil. 94, 1458–1472.
Rennie, M.J., 2009. Anabolic resistance: the effects of aging, sexual dimorphism, and immobilization on human muscle protein turnover. Appl. Physiol. Nutr. Metab. 34, 377–381.
Rikli, R.E., Jones, C.J., 2013. Development and validation of criterion-referenced clinically relevant fitness standards for maintaining physical independence in later years. Gerontologist 53, 255–267.
Romero-Arenas, S., Blazevich, A.J., Martinez-Pascual, M., Perez-Gomez, J., Luque, A.J., Lopez-Roman, F.J., Alcaraz, P.E., 2013. Effects of high-resistance circuit training in an elderly population. Exp. Gerontol. 48, 334–340.
Silva, N.L., Oliveira, R.B., Fleck, S.J., Leon, A.C., Farinatti, P., 2014. Influence of strength training variables on strength gains in adults over 55 years-old: a meta-analysis of dose–response relationships. J. Sci. Med. Sport 17, 337–344.
Skelton, D.A., Greig, C.A., Davies, J.M., Young, A., 1994. Strength, power and related functional ability of healthy people aged 65–89 years. Age Ageing 23, 371–377.
Skovdal Rathleff, M., Thorborg, K., Bandholm, T., 2013. Concentric and eccentric time-under-tension during strengthening exercises: validity and reliability of stretch-sensor recordings from an elastic exercise-band. PLoS One 8, e68172.
Smith, G.I., Atherton, P., Villareal, D.T., Frimel, T.N., Rankin, D., Rennie, M.J., Mittendorfer, B., 2008. Differences in muscle protein synthesis and anabolic signaling in the postabsorptive state and in response to food in 65–80 year old men and women. PLoS One 3, e1875.
Smith-Ray, R.L., Makowski-Woidan, B., Hughes, S.L., 2014. A randomized trial to measure the impact of a community-based cognitive training intervention on balance and gait in cognitively intact Black older adults. Health Educ. Behav. 41, 62S–69S.
Sousa, N., Mendes, R., Monteiro, G., Abrantes, C., 2014. Progressive resistance strength training and the related injuries in older adults: the susceptibility of the shoulder. Aging Clin. Exp. Res. 26, 235–240.
Statistik Austria, 2015. Bevölkerungsstand. http://www.statistik.at/web_de/statistiken/menschen_und_gesellschaft/bevoelkerung/bevoelkerungsstruktur/bevoelkerung_nach_alter_geschlecht/index.html (2015-06-30).
Steffen, T.M., Hacker, T.A., Mollinger, L., 2002. Age- and gender-related test performance in community-dwelling elderly people: Six-Minute Walk Test, Berg Balance Scale, Timed Up & Go Test, and gait speeds. Phys. Ther. 82, 128–137.
Steib, S., Schoene, D., Pfeifer, K., 2010. Dose–response relationship of resistance training in older adults: a meta-analysis. Med. Sci. Sports Exerc. 42, 902–914.
Tipton, K.D., Rasmussen, B.B., Miller, S.L., Wolf, S.E., Owens-Stovall, S.K., Petrini, B.E., Wolfe, R.R., 2001. Timing of amino acid-carbohydrate ingestion alters anabolic response of muscle to resistance exercise. Am. J. Physiol. Endocrinol. Metab. 281, E197–E206.
Trost, S.G., McIver, K.L., Pate, R.R., 2005. Conducting accelerometer-based activity assessments in field-based research. Med. Sci. Sports Exerc. 37, S531–S543.
Tudor-Locke, C., Craig, C.L., Brown, W.J., Clemes, S.A., De Cocker, K., Giles-Corti, B., Hatano, Y., Inoue, S., Matsudo, S.M., Mutrie, N., Oppert, J.M., Rowe, D.A., Schmidt, M.D., Schofield, G.M., Spence, J.C., Teixeira, P.J., Tully, M.A., Blair, S.N., 2011. How many steps/day are enough? For adults. Int. J. Behav. Nutr. Phys. Act. 8, 79.
United Nations, Department of Economic and Social Affairs, Population Division, 2013. World Population Ageing 2013. http://www.un.org/en/development/desa/population/publications/pdf/ageing/WorldPopulationAgeing2013.pdf (2015-02-12).
Van Roie, E., Delecluse, C., Coudyzer, W., Boonen, S., Bautmans, I., 2013. Strength training at high versus low external resistance in older adults: effects on muscle volume, muscle strength, and force-velocity characteristics. Exp. Gerontol. 48, 1351–1361.
Vellas, B., Guigoz, Y., Garry, P.J., Nourhashemi, F., Bennahum, D., Lauque, S., Albarede, J.L., 1999. The Mini Nutritional Assessment (MNA) and its use in grading the nutritional state of elderly patients. Nutrition 15, 116–122.
Verreijen, A.M., Verlaan, S., Engberink, M.F., Swinkels, S., de Vogel-van den Bosch, J., Weijs, P.J., 2015. A high whey protein-, leucine-, and vitamin D-enriched supplement preserves muscle mass during intentional weight loss in obese older adults: a double-blind randomized controlled trial. Am. J. Clin. Nutr. 101, 279–286.
Volkert, D., Sieber, C.C., 2011. Protein requirements in the elderly. Int. J. Vitam. Nutr. Res. 81, 109–119.
von Haehling, S., Morley, J.E., Anker, S.D., 2010. An overview of sarcopenia: facts and numbers on prevalence and clinical impact. J. Cachex. Sarcopenia Muscle 1, 129–133.
Williams, M.A., Haskell, W.L., Ades, P.A., Amsterdam, E.A., Bittner, V., Franklin, B.A., Gulanick, M., Laing, S.T., Stewart, K.J., American Heart Association Council on Clinical, C., American Heart Association Council on Nutrition, P.A., Metabolism, 2007. Resistance exercise in individuals with and without cardiovascular disease: 2007 update: a scientific statement from the American Heart Association Council on Clinical Cardiology and Council on Nutrition, Physical Activity, and Metabolism. Circulation 116, 572–584.
Willis, S.L., Tennstedt, S.L., Marsiske, M., Ball, K., Elias, J., Koepke, K.M., Morris, J.N., Rebok, G.W., Unverzagt, F.W., Stoddard, A.M., Wright, E., Group, A.S., 2006. Long-term effects of cognitive training on everyday functional outcomes in older adults. JAMA 296, 2805–2814.
Wolfe, R.R., Miller, S.L., Miller, K.B., 2008. Optimal protein intake in the elderly. Clin. Nutr. 27, 675–684.
Yang, Y., Breen, L., Burd, N.A., Hector, A.J., Churchward-Venne, T.A., Josse, A.R., Tarnopolsky, M.A., Phillips, S.M., 2012. Resistance exercise enhances myofibrillar protein synthesis with graded intakes of whey protein in older men. Br. J. Nutr. 108, 1780–1788.
