# Supplementary material for: Effects of elastic band resistance training on the physical and mental health of elderly individuals: A mixed methods systematic review
Source: PLoS One. 2024 May 13;19(5):e0303372. doi: 10.1371/journal.pone.0303372 (PMC11090353; doi:10.1371/journal.pone.0303372)
Supplement: S1 File — (ZIP) [file pone.0303372.s001.zip › Supporting Information/Included study 48.pdf]

©Journal of Sports Science and Medicine (2015) **14**, 716-722
http://www.jssm.org

Research article

Influence of Two Different Exercise Programs on Physical Fitness and Cognitive Performance in Active Older Adults: Functional Resistance-Band Exercises vs. Recreational Oriented Exercises

Hernán Ponce-Bravo [1,2], Christian Ponce [1], Belén Feriche [1] and Paulino Padial [1]✉
[1] Department of Physical Education and Sport, Faculty of Sport Sciences, University of Granada, Granada, Spain
[2] Faculty of Health Sciences, School of Physical Culture, National University of Chimborazo, Ecuador

Abstract
This study examines the impact of a resistance-band functional exercise program, compared with a recreational exercise program, on physical fitness and reaction times in persons older than 60 years. Fifty-four community-dwelling volunteers (71.76 ± 6.02 years) were assigned to a specific exercise program: Functional activity program (focused on resistance-band multi-joint activities; experimental group, EG), or recreational physical activity program (with gross motor activities of ludic content; control group, CG). Before and after the intervention, we determined cognitive capacity in terms of simple reaction time (S-RT), choice reaction time (C-RT) and fitness. In both groups physical performance improved, though this improvement was more marked in the EG for grip strength, arm strength and gross motor abilities ($p < 0.05$). Reaction times were better only in EG (S-RT = 10.70%, C-RT = 14.34%; $p < 0.05$) after the corresponding physical training intervention. The training period showed no effect on the moderate relationship between both RT and gross motor abilities in the CG, whereas the EG displayed an enhanced relationship between S-RT and grip-strength as well as the C-RT with arm strength and aerobic capacity ($r \sim 0.457$; $p < 0.05$). Our findings indicate that a functional exercise program using a resistance band improves fitness and cognitive performance in healthy older adults.

Key words: Aging, reaction time, physical activity program, cognitive capacity.

Introduction

According to estimates by the World Health Organization, elderly persons make up the most rapidly growing section of the population worldwide. It is forecast that by 2050, around 2000 million people –or one in four– will be older than 60 years of age.

The physical and cognitive decline that occurs during aging translates to an inability to carry out daily living tasks with consequent impacts on social relationships and quality of life. This has prompted the design of programs for this population sector targeted at improving functional health and promoting the independence of the elderly in their environment. In this context, routine physical exercise plays a major role in the life quality and expectancy of older adults (Blain et al., 2000; Katula et al., 2008; Poon and Fung, 2008; Vogel et al. 2009).

A lack of agility and dynamic balance (gross motor abilities) is a significant risk factor for loss of independence and increases the risk of falls. Balance also affects daily living activities such as standing, bending, climbing stairs, walking or responding to external stimuli (Sturnieks et al., 2008). The reason for this is the progressive deterioration in neurophysical skills that occurs with age, impairing sensory-motor functions and producing deficiencies in perception, muscle and cognitive function, and thus affecting balance and the risk of falling (Sturnieks et al., 2008). Regularly practicing some form of physical exercise reduces the risk of falls (Howe et al., 2007), essentially because of improved muscular strength, agility, dynamic balance and coordination (Blain et al., 2000; Karinkanta et al., 2009; Orr et al., 2008) and also helps preserve cognitive skills (Angevaren et al., 2008; Blain et al., 2000; Brisswalter et al., 2002; Colcombe and Kramer, 2003; Liu-Ambrose and Donaldson., 2009; Williamson et al. 2009). Indeed, different exercise programs seem to achieve proportional changes in physical condition and cognition in older adults (Berryman et al, 2014). However, the physiological pathway of these results displays a large variety of mechanisms liked with their cognitive impact, which nowadays should be clarified (Berryman et al., 2014; Voelcker-Rehage et al., 2010).

The available data indicates a direct link between improved cognitive performance and training programs designed to improve cardiovascular fitness (Colcombe & Kramer, 2003), strength and balance (Araya, 2011). However, there is no general consensus regarding the details of the most appropriate fitness training program (including the best intervention type, length of exercise program, session duration, etc.) or of its effects on indicators of cognitive function. Some of these indicators are simple reaction time (S-RT) (Dustman et al. 1984), choice reaction time (C-RT) (Van Boxtel et al., 1997), or visual-spatial tasks (Shay and Roth, 1992), which are considered as key markers of the functional independence of the elderly (Colcombe and Kramer, 2003). Training programs for older adults have been mostly based on exercises designed to improve cardiovascular fitness and muscular strength (de Vreede et al., 2005; Kalapotarakos et al., 2006; Katula, et al. 2008; Liu-Ambrose and Donaldson, 2009; Van Boxtel et al., 1997). However, it has not always been possible to correlate such improvements with a capacity to better carry out daily living tasks. Some authors propose the inclusion of functional exercises (multi-joint motor tasks that involve several body parts) in interventions planned for older adults (de Bruin and Murer, 2007; de Vreede et al. 2005) though their effects on cog-

Received: 10 June 2015 / Accepted: 29 July 2015 / Published (online): 24 November 2015

nitive performance and general fitness have not yet been established.

This study was designed to determine the impact of a program of functional exercises using elastic bands versus a recreational activity program on fitness and cognitive performance in active elderly participants older than 60 years of age.

Methods

Participants

Fifty-four participants were recruited from the community among older adults attending regular physical activity classes organized by the town hall (age 70.57 ± 5.46 years; weight 72.96 ± 10.54 kg; height 1.56 ± 0.09 m; 6 men, 48 women). Exclusion criteria were: A diagnosis of a progressive somatic or psychiatric disease, or any illness preventing participation in physical activities. The study protocol adhered to the tenets of the Declaration of Helsinki and received institutional review board approval. Written informed consent was obtained from each participant.

The participants enrolled were assigned to two groups matched in terms of their baseline reaction times and physical fitness, as well as the compatibility training schedule : an experimental group (EG; n = 22) and a control group (CG; n = 32). Participants in each group completed a 4-week training program consisting of 5 weekly sessions of 50 min each. Before and after the training intervention, fitness and cognitive performance were assessed in each participant.

Study design

In a longitudinal-experimental study, pre- and post-intervention data were compared in an experimental (functional training with resistance elastic-bands; EG) and control (recreational training; CG) group. The effects of both training programs on physical fitness and cognitive performance (simple and choice reaction times) were determined by intra- and intergroup comparisons of means. Sampling was performed via an intentional non-probabilistic convenience procedure.

Both types of training included a multidimensional activity program (endurance, strength, balance, gross motor, and flexibility training), which is considered optimal for health and functional benefits in older people (Cress et al., 2005). Differences between training groups came from the nature of the exercises (multi-joint vs analytical) and the focus towards which they were directed (physical condition improvement or ludic orientation): 1) Resistance-band functional training sessions combined several objectives simultaneously by means of exercise that incorporated multi-joint motor tasks that involved several body parts (functional exercises; de Bruin and Murer, 2007; de Vreede et al., 2005). These exercises were executed with low-resistance elastic-bands in order to highlight the strength content in each session (Cress et al., 2005); 2) Recreational training also combined several objectives simultaneously but used more analytical low-load exercises applied in a ludic form.

The variables used as indicators of physical fitness were: leg strength (LS) using the Chair-stand test; right/left arm strength (R/L AS) using the Arm curl test; and gross motor abilities (AG; 8-Foot up-and-go). For all tests, we followed the procedures and recommendations described for the Senior Fitness Test (SFT) battery (Rikli and Jones, 2001). The aerobic endurance test was reduced to 2 min, and performance subsequently estimated in the 6 min-test according to the procedure of Butland et al, (1982). Finally, expected aerobic performance (EAP) was calculated as the difference between aerobic performance and the expected minimum distance (Jones and Rikli, 2002).

Additionally, handgrip strength was determined in a maximum dynamometry test (dynamometer T.K.K. 5401 Grip-D, Tokyo Japan). From a standing position with arms extended on both sides and hands facing the thighs, participants were instructed to exert as much pressure as possible for 3-5 s on the grip adapted to hand-breadth. After 3 attempts with each hand, lower values were eliminated. Performance was calculated as the sum of the kg generated using both hands (HGS).

Cognitive performance was assessed by recording reaction times (RT) in the simple reaction (S-RT) and choice (C-RT) tests using the system Whole Body Reaction Measuring Equipment (FT-3130, TKK Takei & Company, LTD, Tokyo, Japan). This system comprises a table with three buttons and three light bulbs (red, yellow and blue). To determine S-RT, the participants sit at the table with hands on each side of the buttons. The tests consist of pressing any button with either hand as quickly as possible after a bulb lights up. When participant is ready, the evaluator presses a button and a random time between 1 and 3 s is set automatically by the system before the light turns on. The test result is recorded in milliseconds. To determine C-RT, participants respond to the three possible colored bulbs by pressing a different button (left, middle, right) for each color. Three attempts were allowed per test and the lowest value recorded as the result.

Testing procedure

Before and after the training interventions, all measurements were taken and tests performed on a single day. Each testing session commenced with the measurements: height (Holtain, Dyffed, UK), weight (Tanita TBF-300a, IL, USA) and waist/hip measurements. Body mass index (BMI) was determined using the Quetelet procedure as the ratio between weight (kg) and height squared (m^2). The waist-hip ratio was recorded using the corresponding measurements in cm. Anthropometric measurements were followed by the RT tests and then by the physical fitness tests in the order: strength tests (legs, arms and handgrip), gross motor and aerobic performance test.

Intervention programs

The intensity and difficulty of the experimental and control programs were increased as the participants adapted to each level of exercise. Sessions were always supervised by a sports expert.

In the experimental group, each training session commenced with 5 min of introduction, organization and warm-up (functional and stretching exercises). In the main part of the session (40 min), participants performed the functional exercises with elastic bands: aerobic (8 min), gross motor activities, action/reaction speed (7 min) and floor exercises (25 min). Each session finished with 5 min of active relaxation exercises. Training loads were adjusted according to the recommendations of Chodzko-Zajko et al. (2009). In weeks 1 and 4, loads were 8 repetitions of each exercise performed at an intensity of 5-6 on a scale of 0-10. This intensity was recorded and prescribed according to the expert's perception of each training session. In weeks 2 and 3, the load was increased to 12 repetitions and intensity to 7-8.

Training sessions for participants in the control group commenced with a warm up (10 min) of games and mixed exercises. The main part of each session (30 min) consisted of traditional aerobic exercises (20 min) and recreational (10 min) activities. All sessions ended with 10 min of relaxation exercises. Training loads were adjusted as in EG. In weeks 1 and 4, the load was 6 repetitions per exercise at an intensity of 3-4 on a scale of 0-10. In weeks 2 and 3, this volume was increased to 8 repetitions at an intensity of 5-6 (Chodzko-Zajko et al., 2009).

Statistical analysis

Data are provided as the mean and standard deviation (SD). The Shapiro-Wilk test was used to determine the distribution of data. To assess the effect of treatment on the measures of physical fitness, body composition and cognitive capacity in each group, we used a comparison of means test for paired data (pre vs. post intervention), or the Wilcoxon test for variables not normally distributed. The Student t-test for independent samples or Mann-Whitney U test were used to compare the difference produced in each variable (post-intervention value minus pre-intervention value) between CG and EG. The homogeneity of groups was determined using the Levene test. For non-homogeneous comparisons, the Welch test was employed. Correlations between physical condition variables and reaction times (S-TR and C-RT) were quantified through Pearson's product-moment correlation coefficient (r). Qualitative interpretations of the r coefficients as defined by Hopkins (2002) (0–0.09 trivial; 0.1–0.29 small; 0.3–0.49 moderate; 0.5–0.69 large; 0.7–0.89 very large; 0.9–0.99 nearly perfect; 1 perfect) were provided for all significant correlations. Statistical tests were performed using the software SPSS version 20.0 (SPSS, Chicago, IL, USA) and Microsoft Excel 2007. The confidence interval was set at 95%.

Results

Before the physical activity program, the experimental and control groups were homogenous in terms of body composition and indicators of fitness and cognitive performance ($p > 0.05$).

The pre-post comparisons of the variables recorded in each group may be seen in Table 1. Intragroup comparisons revealed increases in leg and arm strength and improved aerobic performance in both groups ($p < 0.05$). The CG participants also showed a slight reduction in BWI ($p < 0.05$). Additionally, in EG, handgrip strength and gross motor abilities also improved ($p < 0.01$) and significant enhancements were detected in reaction times in response to the training program (S-RT: -1.06 ± 14.99% vs. 10.70 ± 15.38%; C-RT: 3.63 ± 15.48% vs. 14.34 ± 15.58% for CG and EG respectively; $p < 0.01$) (Table 1).

The comparison of the two training programs for the variables examined is detailed in Table 2. Compared to CG, the EG showed greater improvements in response to training in handgrip strength (16.31%; $p < 0.01$), arm strength (R-AS: 14.37%; L-AS: 16.17%; $p < 0.05$), and gross motor abilities (15.96%; $p < 0.001$). On average, functional training led to an 11% improvement in cognitive performance over recreational training (S-RT: 11.76%; C-RT: 10.71%; $p < 0.05$) (Table 2).

Tables 3 and 4 show the relationship between the S-RT or C-RT and the fitness variables before and after the training period. In pre- intervention, Pearson´s product-moment correlation coefficients showed a wake-moderate relationship between S-RT and C-RT and gross motor abilities in the control group, there were however no relationships of significance detected for experimental group. In post- intervention, while no changes in correlations were observed in the control group with respect to

Table 1. Response to the training program recorded in the experimental and control groups. Data expressed as mean (SD).

		CG			EG		
		PRE	POST	*P*-value	PRE	POST	*P*-value
	**BMI (kg·m^{-2})**	30.31 (4.65)	30.12 (4.56)	.300	30.10 (4.61)	30.0 (4.71)	.254
	**WH-r (%)**	.91 (.09)	.89 (.06)	.116	.91 (.06)	.90 .05)	.129
	**S-RT (ms)**	.51 (.08)	.52 (.08)	.874	.47 (.09)	.41 (.08)	**.006**
	**C-RT (ms)**	.58 (.11)	.55 (.11)	.119	.56 (.14)	.48 (.12)	**.001**
握力	**HGS (kg)**	39.91 (11.94)	41.59 (13.86)	.207	44.42 (12.41)	52.25 (14.68)	**<.001**
腿力量---椅子站立	**LS (rep)**	11.75 (2.13)	14.31 (2.15)	**<.001**	12.00 (2.56)	14.86 (2.77)	**<.001**
手臂力量-----手臂卷曲（次数）	**R-AS(rep)**	15.39 (2.64)	18.48 (2.84)	**<.001**	14.68 (2.34)	19.55 (3.20)	**<.001**
	**L-AS (rep)**	16.13 (2.75)	19.13 (3.00)	**<.001**	15.09 (2.37)	20.27 (3.33)	**<.001**
粗运动能力-----AUG	**AG (s)**	6.13 (1.20)	6.27 (.97)	.210	5.91 (.98)	5.11 (.72)	**.001**
	**EAP (m)**	-9.10 (62.26)	30.54 (51.82)	**.001**	5.66 (65.26)	29.36 (73.92)	**.046**

CG = control group; EG = experimental group; PRE = pre-intervention; POST = post-intervention; BMI = body mass index; WH-r = waist-hip ratio; S-RT = simple reaction time; C-RT = choice reaction time; HGS = handgrip strength; LS = leg strength; R-AS = right arm strength; L-AS = left arm strength; AG = gross motor abilities; EAP = expected aerobic performance; *P*-value = statistical significance at 95% CI (values in bold indicate a significant difference).

Table 3. Correlation between reaction time (Simple and Choice) and physical performance variables in the control group.

	PRE				POST			
	S-RT		C-RT		*S-RT*		C-RT	
	r	*P*-value	*r*	*P*-value	*r*	*P*-value	*r*	*P*-value
**HGS (kg)**	-.21	.239	-.20	.273	-.27	.128	-.27	.138
**LS (rep)**	-.23	.204	-.08	.679	-.16	.368	-.29	.106
**R-AS (rep)**	-.24	.181	-.13	.469	-.24	.200	-.21	.260
**L-AS (rep)**	-.32	.075	-.18	.316	-.27	.149	-.21	.265
**AG (s)**	.57	**.001**	.36	**.041**	.54	**.001**	.44	**.013**
**EAP (m)**	-.21	.245	-.08	.646	-.23	.280	-.24	.255

r = Pearson's linear correlation coefficient; HGS = handgrip strength; LS = leg strength; R-AS = right arm strength; L-AS = left arm strength; AG = gross motor abilities; EAP = expected aerobic performance; *P*-value = statistical significance at the 95% CI (values in bold indicate a significant difference).

Table 4. Correlation between reaction time (Simple and Choice) and physical performance variables in the experimental group.

	PRE				POST			
	S-RT		C-RT		*S-RT*		C-RT	
	r	*P*-value	*r*	*P*-value	*r*	*P*-value	*r*	*P*-value
**HGS (kg)**	-.03	.905	-.32	.144	-.53	**.012**	-.29	.182
**LS (rep)**	.04	.868	-.07	.744	-.04	.865	-.02	.923
**R-AS (rep)**	.19	.378	-.24	.275	-.37	.085	-.43	**.047**
**L-AS (rep)**	.33	.135	-.09	.693	-.41	.058	-.46	**.033**
**AG (s)**	-.20	.359	-.12	.600	.32	.146	.37	.094
**EAP (m)**	-.08	.747	-.25	.256	-.41	.058	-.50	**.021**

r = Pearson's linear correlation coefficient; HGS = handgrip strength; LS = leg strength; R-AS = right arm strength; L-AS = left arm strength; AG = gross motor abilities; EAP = expected aerobic performance; *P*-value = statistical significance at the 95% CI (values in bold indicate a significant difference).

pre-intervention results, the experimental group displayed a moderate correlation between S-RT and handgrip strength and between C-RT and arms strength and the expected aerobic performance ($p < 0.05$).

Table 2. Comparison of training programs outcome. Data expressed as mean (SD).

	Diff CG	Diff EG	*p*-value
**BMI (kg·m^{-2})**	-.19 (.49)	-.10 (.42)	.485
**WH-r (%)**	-.02 (.08)	-.01 (.03)	.515
**S-RT (ms)**	.00 (.08)	-.06 (.09)	**.036**
**C-RT (ms)**	-.03 (.09)	-.09 (.10)	**.030**
**HGS (kg)**	1.68 (7.03)	7.84 (8.50)	**.005**
**LS (rep)**	2.56 (1.78)	2.86 (2.10)	.572
**R-AS (rep)**	2.56 (3.79)	4.86 (3.33)	**.025**
**L-AS (rep)**	1.78 (5.47)	5.18 (3.25)	**.005**
**AG (s)**	014 (.77)	-.79 (.89)	**<.001**
**EAP (m)**	39.64 (48.27)	23.70 (52.33)	.288

CG = control group; EG = experimental group; Diff CG = post-pre training difference recorded in CG; Diff EG = post-pre training difference recorded in EG; BMI = body mass index; WH-r = waist-hip ratio; S-RT = simple reaction time; C-RT = choice reaction time; HGS = handgrip strength; LS = leg strength; R-AS = right arm strength; L-AS = left arm strength; AG = gross motor abilities; EAP = expected aerobic performance; *P*-value = statistical significance at the 95% CI (values in bold indicate a significant difference).

Discussion

This study sought to compare the effects of two physical exercise programs (functional with elastic bands vs. recreational) on the cognitive performance of adults older than 60 years measured in terms of reaction times (S-RT and C-RT). Our main finding was that 20 sessions of either training mode, despite considerable content and workload differences, showed beneficial effects on overall leg/arm strength and aerobic capacity ($p < 0.05$), though no appreciable impacts were produced on body composition. However, a functional exercise program using an elastic exercise band led to additional improvements over those produced in the control group in arm strength (Δ 15.27%; $p < 0.05$), handgrip strength (~ 16.32%; $p < 0.001$), gross motor abilities (~ 15.95%; $p < 0.01$), and cognitive performance (S-RT: 10.70%, C-RT: 14.34%; $p < 0.01$). Additionally, only the EG improved the relationship between reaction times and fitness variables, confirming that better cognitive processes can be achieved as physical condition improves. However, the design used in this study did not allow us to determine if the mechanism responsible for this result is due to a minimum level of change in physical performance, or if it is inherent in the training method employed in this study (combination of functional exercises and elastic bands).

The available literature is replete with reports of training programs for individuals older than 60 years targeted at improving both physical (Blain et al., 2000; Karinkanta et al., 2009; Orr et al., 2008) and cognitive skills (Angevaren et al., 2008; Berryman et al., 2014; Brisswalter et al., 2002; Colcombe and Kramer 2003; Forte et al., 2013; Liu-Ambrose and Donaldson, 2009; Williamson et al., 2009). Baseline physical fitness and reaction time data for our study population is consistent with those reported for the same age group in similar studies (Jones and Rikli, 2002; Van Boxtel et al., 1997). In the present study, the control program was designed to represent the more traditional interventions with a high recreational component and low workload. In contrast, the experimental program consisted of combined objective

sessions incorporating motor tasks simultaneously involving several body parts (functional exercises) (de Bruin and Murer, 2007; de Vreede et al., 2005). These components address in a single session a large number of components implicated in cognitive decline (Voelcker-Rehage et al., 2010). The use of elastic bands increases strength gains as reflected by the improved effects of the experimental intervention over the control intervention on handgrip strength (16.31%; $p < 0.01$) and arm strength (R-AS = 14.37%; L-AS = 16.17%; $p < 0.05$). The shorter simple reaction time recorded in the EG and lack of change in this variable observed in the control group intervention concurs with the recommendation by Colcombe & Kramer (2003) that cardiovascular and resistance training should be combined in order to benefit cognitive skills (Tables 1 and 2).

Our findings indicate that 20 sessions of recreational physical training produced fitness but not cognitive benefits (Table 1). In contrast, a similar program involving 20 sessions of resistance-band functional training induced a greater impact on fitness and a mean 11% improvement in reaction times (simple and choice) (Tables 1 and 2). After 3 months (2 sessions/week) of multicomponent (neuromuscular coordination, balance, agility, and cognitive executive control) or progressive resistance training for strength gains, Forte et al (2013) observed that the beneficial effects of a resistance program on cognitive function were mediated by gains in muscular strength. However, the benefits of multicomponent training displayed a direct cognitive stimulation by a direct influence on neuromuscular coordination and perceptual motor adaptations. Reports in the current literature have described both beneficial (Kalapotharakos et al., 2006) and inappreciable (Paas et al., 1994; Powell, 1983) effects of physical activity programs on reaction times. Reaction time decreases with age (Sturnieks, et al., 2008; Van Boxtel et al., 1997) independently of gender (Silverman, 2006; Wellmon, 2012) and moderately dependently on changes in aerobic capacity (Colcombe and Kraemer, 2003; Van Boxtel et al., 1997). The participants of our study, with a mean age of 70, showed no impairment in cognitive function and their aerobic performance was just at the lower expected limit. In contrast with other reports (Barella et al., 2010; Kalapotharacos et al., 2006), a beneficial effect was detected on aerobic capacity in response to both training interventions ($p < 0.05$), although only the experimental intervention led to improved reaction times ($p < 0.01$). Contrary with the findings of Van Boxtel et al. (1997), a positive change in correlation was detected between the C-RT and aerobic performance in EG (Table 4).

Other authors have reported cognitive function improvements ranging from 13.4% to 9.6% in response to a 12-week recreational activity program of 2 and 3 sessions per week respectively in participants with a mean age of 79 years ($p < 0.001$) (Gálvez, 2012; Pereira, 2011). Despite the similarity with our control intervention, the different findings of these studies may be attributed to the mild cognitive impairment described for the participants of the studies by Gálvez and Pereira (scores of ~19 out of 30 in the Minimental test; MSSE; Folstein et al., 1975), and the 9-year difference in age with our study participants. These differences along with the different training regimens (20 vs. 24 vs. 36 sessions), could in part explain the different physical performance gains recorded in the different studies and our control group. Studies that have linked improved cognitive function to gains in cardiovascular capacity seem to suggest greater dedifferentiation in neuron activation pathways in younger adults (Colcombe and Kramer, 2003). Thus, recreational type activity sessions do not seem to offer a stimulus that is able to improve both the physical and cognitive capacities of older persons with no cognitive impairment.

Other studies have shown a direct relationship between improved cognitive performance and cardiovascular fitness, strength or balance training (Araya, 2011; Colcombe and Kramer, 2003). Araya et al. (2012), in response to a 12-week physical training program (3 sessions per week) completed by 33 women with a mean age of 72 years and with mild cognitive decline (MSSE = 24 out of 30), noted the improved fitness of the participants along with a cognitive capacity improvement of 7.4% ($p < 0.05$). In agreement with these findings, we observed improvements in the fitness (strength and gross motor abilities; $p < 0.05$) and cognitive skills (11% in RT, $p < 0.05$) of participants in the functional training group compared to the recreational intervention group (Table 2). Hence, in only 20 sessions, it seems that a functional resistance-band exercise program is able to improve both physical fitness and cognitive function in elderly participants with no cognitive deficiency. Such improvements are likely to play a role in preventing cognitive decline and maintaining independence.

Finally, despite the apparent contribution of physical activity programs in ameliorating cognitive skills (Colcombe and Kramer, 2003; Forte et al, 2013; Voelcker-Rehage et al., 2010), only a moderate correlation was observed between handgrip-strength and the S-RT and between arm-strength and endurance and the C-RT ($p < 0.05$) in EG. No such correlation was found in the CG despite the improvements in leg and arm strength and aerobic performance. Considering the greater improvements in strength and coordination following resistance band exercises when compared to recreational exercises, our results concur with other studies which reveal that the combination of physical and cognitive training maximizes cognitive benefits in the elderly (Foster et al., 2013; Oswal et al, 2006). Other studies have also described the correlation between C-RT and aerobic capacity (Rikli and Edwards, 1991). However, in our study the combination of functional exercises with an elastic band in the EG did not allow us to determine if the mechanism responsible for these findings results from a minimum change in physical performance, or if this benefit is inherent to the experimental training method used. Indeed, the mechanisms that cause the cortical changes linked to cognitive performance seem to differ depending on the intervention (Berryman et al., 2014; Voelcker-Rehage et al., 2010). Our results indicate a need for gains greater than 20-23% in handgrip, arm and leg strength for the neurophysiological mechanisms that regulate sensory-motor function to produce a beneficial impact on percep-

tion and reaction time, improving the gross motor abilities (such as agility and balance), and thus reducing the risk of falls (Sturnieks et al., 2008).

Conclusion

In summary, our findings indicate that 20 sessions of functional resistance or recreational training lead to general upper and lower body strength and aerobic capacity gains in adults older than 60 years. However, the improved cognitive function observed, as assessed through reaction times, seems more linked to the workload and resistance component of the training program. Thus, programs involving functional exercises with an elastic band improve both simple and choice reaction times and lead to greater gains in gross motor abilities, handgrip and arm strength over the improvement achieved by a more recreational type program. In turn, these latter gains correlate to improved C-RT. Collectively our findings indicate that exercise sessions of more recreational type contents do not seem to constitute a stimulus that is able to improve both physical and cognitive performance in healthy active older adults. We therefore recommend the incorporation of functional elastic-band exercises in physical activity programs designed for this population group.

Acknowledgements
The authors thank the elderly of the village of Maracena (Granada, Spain) and the Maracena Town Hall for their enthusiasm and willingness to participate in this study. The study was financed by Research group SEJ-438 of the Junta de Andalucía.

References

Angevaren, M., Aufdemkampe, G., Verhaar, H.J., Aleman, A. and Vanhees, L. (2008) Physical activity and enhanced fitness to improve cognitive function in older people without known cognitive impairment. *Cochrane Database of Systematic Reviews* **3** (CD005381).

Araya, S. (2011) *Effect of physical activity on fitness and cognitive ability of adult-more women in the community of Iquique, Chile*. Doctoral Thesis, University of Granada, Spain. (In Spanish: English abstract). Available from URL: http://hdl.handle.net/10481/21006.

Araya, S., Padial, P., Feriche, B., Gálvez, A., Pereira, J. and Mariscal-Arcas, M. (2012) Effect of a physical activity program on the anthropometric and physical fitness of women over 60 years. *Nutrición Hospitalaria* **27**, 1472-1479.

Barella, L.A., Etnier, J.L. and Chang, Y.K. (2010) The immediate and delayed effects of an acute bout of exercise on cognitive performance of healthy older adults. *Journal of Aging and Physical Activity* **18**, 87-98.

Berryman, N., Bherer, L., Nadeau,S., Lauziere, S., Lehr, L., Bobeuf,F., Lussier, M., Kergoat, M.J., Minh Vu , T.T. and Bosquet, L. (2014) Multiple roads lead to Rome: combined high-intensity aerobic and strength training vs. gross motor activities leads to equivalent improvement in executive functions in a cohort of healthy older adults. *AGE* **36**, 9710.

Blain, H., Vuillemin, A., Blain, A. and Jeandel, C. (2000) The preventive effects of physical activity in the elderly. *Presse Médicale* **29**, 1240-1248.

Brisswalter, J., Collardeau, M. and René, A. (2002) Effects of acute physical exercise characteristics on cognitive performance. *Sports Medicine* **32**, 555-566.

Butland, R.J., Pang, J., Gross, E.R., Woodcock, A.A. and Geddes, D.M. (1982) Two-, six-, and 12-minute walking tests in respiratory disease. *British Medical Journal* **284,** 1607-1608.

Chodzko-Zajko, W., Proctor, D., Fiatarone S. M., Minson, C., Nigg, C., Salem, G. and Skinner, J. (2009) Exercise and Physical Activity for Older Adults. *Medicine & Science in Sports & Exercise* **41**, 1510-1530.

Colcombe, S. and Kramer, A.F. (2003) Fitness effects on the cognitive function of older adults: a meta-analytic study. *Psychological Science* **14**, 125-130.

Cress, M.E., Buchner, D.M., Prohaska, T., Rimmer, J., Brown, M., Macera, C., DiPietro, L. and Chodzko-Zajko,W. (2005) Best Practices for Physical Activity Programs and Behavior Counseling in Older Adult Populations. *Journal of Aging and Physical Activity* **13**, 6-74

de Bruin, E.D. and Murer, K. (2007) Effect of additional functional exercises on balance in elderly people. *Clinical Rehabilitation* **21**, 112-121.

de Vreede, P.L., Samson, M.M., van Meeteren, N.L., Duursma, S.A. and Vehaar, H.J. (2005) Functional-Task Exercise versus resistance strength exercise to improve daily function in older women: A randomized, controlled trial. *American Geriatrics Society* **53**, 2-10.

Dustman, R.E., Ruhling, R.O., Russell, E.M., Shearer, D.E., Bonekat, W. and Shigeoka, J.W. (1984) Aerobic exercise training and improved neurophysiological function of older adults. *Neurobiology of Aging* **5,** 35-42.

Folstein, M.F., Folstein, S.E. and Mchugh, P.R. (1975) Minimental state. A practical method for grading the cognitive state of patients for the clinician. *Journal of Physchiatric Research* **12,** 189-198.

Forte, R., Boreham, C., Costa, J., De Vito, G., Brennan, L., Gibnet, E.R. and Pesce, C. (2013). Enhancing cognitive functioning in the elderly: multicomponent vs resistance training. *Clinical Interventions in Aging* **8**, 19-27.

Gálvez, A. (2012) *Effect of physical activity on cognition performance in patients over 60 years residents into a geriatric center*. Doctoral Thesis, University of Granada, Spain. (In Spanish: English abstract). Available from URL: http://hdl.handle.net/10481/21742

Hopkins, W.G. (2002) A scale of magnitudes for effect statistics. A new view of statistics. Available from URL: http://sportsci.org/ resource/stats/effectmag.html

Howe, T.E., Rochester, L., Jackson, A., Banks, P.M. and Blair, V.A. (2007) Exercise for improving balance in older people. *Cochrane Database of Systematic Reviews* **4**, CD004963.

Jones, C.J. and Rikli, R.E. (2002) Measuring functional fitness of older adults. *The Journal on Active Aging* **1**, 24-30.

Kalapotarakos, V.I., Michalopoulos, M., Strimpakos, N., Diamantopoulos, K. and Tokmakidis, S.P. (2006). Functional and Neuromotor Performance in Older Adults: Effect of 12 Weeks of Aerobic Exercise. *American Journal of Physical Medicine and Rehabilitation* **85,** 61-67.

Karinkanta, S., Heinonen, A., Sievänen, H., Uusi-Rasi, K., Fogelholm, M. and Kannus, P. (2009) Maintenance of exercise-induced benefits in physical functioning and bone among elderly women. *Osteoporosis International* **20**, 665-674.

Katula, J.A., Rejeski, W.J. and Marsh, A.P. (2008) Enhancing quality of life in older adults: A comparison of muscular strength and power training. *Health and Quality of Life Outcomes* **6**, 45-53.

Liu-Ambrose, T. and Donaldsonm, M.G. (2009) Exercise and cognition in older adults: is there a role for resistance training programmes? *British Journal of Sports Medicine* **43**, 25-27.

Orr, R., Raymond, J. and Fiatarone Singh, M. (2008) Efficacy of progressive resistance training on balance performance in older adults: a systematic review of randomized controlled trials. *Sports Medicine* **38**, 317-343.

Oswald, W., Gunzelmann, T., Rupprecht, T. and Hagen, B. (2006) Differential effects of single versus combined cognitive and physical training with older adults: the SimA study in a 5-year perspective. *European Journal of Ageing* **3**, 179-192.

Paas, F.G., Adam, J.J., Jansen, G.M., Vrenken, J.G. and Bovens, A.M. (1994) Effect of a 10-month endurance-training program on performance of speeded perceptual-motor tasks. *Perceptual and Motor Skills* **78**, 1267-1273.

Pereira, J. (2011) *Physical activity and cognition in human aging.* Doctoral Thesis. University of Granada, Spain. (In Spanish: English abstract). Available from URL: http://hdl.handle.net/ 10481/21024.

Poon, C.Y. and Fung, H.H. (2008) Physical activity and psychological well-being among Hong Kong Chinese older adults: exploring the moderating role of self-construal. *The International Journal*

of Aging and Human Development **66**, 1-19.

Powell, R.R. (1983) Reaction time changes following aerobic conditioning. *Journal of Human Movement Studies* **9**, 145-150.

Rikli, R.F. and Edwards, D.J. (1991) Effects of three years exercise program on motor function and cognitive processing speed in older women. *Research Quarterly for Exercise and Sport* **62**, 61-67.

Rikli, R. and Jones, C. (2001) *Senior Fitness Test Manual.* Human Kinetics, Champaign, Illinois.

Shay, K. and Roth, D. (1992) Association between aerobic fitness and visuospatial performance in healthy older adults. *Psychology and Aging* **7**, 15-24.

Silverman, I.W. (2006) Sex differences in simple visual reaction time: A historical meta-analysis. *Sex Roles* **54**, 57-68.

Sturnieks, D.L., George, R. and Lord, S.R. (2008) Balance disorders in the elderly. *Neurophysiologie Clinique* **38**, 467-478.

Van Boxtel, M.P., Paas, F.G., Houx, P.J., Adam, J.J., Teeken, J.C. and Jolles, J. (1997) Aerobic capacity and cognitive performance in a cross-sectional aging study. *Medicine & Sciencie in Sports and Exercise* **29**, 1357-1365.

Voelcker-Rehage, C., Godde, B. and Staudinger, U.M. (2010) Physical and motor fitness are both related to cognition in old age. *European Journal of Neuroscience* **31**, 167-176.

Vogel, T., Brechat, P., Leprêtre, P.M., Kaltenbach, G., Berthel, M. and Lonsdorfer, J. (2009) Health benefits of physical activity in older patients: a review. *International Journal of Clinical Practice* **63**, 303-320.

Wellmon, R. (2012) Does the attentional demand of walking differ for older men and women living independently in the community? *Journal of Geriatric Physical Therapy* **35**, 55-61.

Williamson, J.D., Espeland, M., Kritchevsky, S.B., Newman, A.B., King, A.C., Pahor, M. and Miller, M.E. (2009) Changes in cognitive function in a randomized trial of physical activity: results of the lifestyle interventions and independence for elders pilot study. *Journal of Gerontology: Medical Sciences* **64**, 688-694.

Key points

- Better cognitive processes can be achieved as physical condition improves
- Exercise sessions of a more recreational type do not seem to constitute a stimulus able to improve both physical and cognitive performance in healthy active older adults
- The improvement of cognitive function, as assessed through reaction times, seems more linked to the workload and strength component of the training program.

AUTHOR BIOGRAPHY

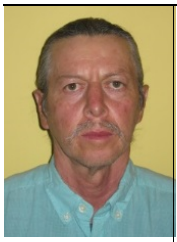

Hernán PONCE-BRAVO

Employment

Lecturer in Sports Training, Swimming Training. Department of Physical Education and Sport Training, National University of Chimborazo- Ecuador. PhD student. Department of Physical Education and Sport, University of Granada. Spain.

Degree

MSc

Research interests

Physical activity and health.

E-mail: herpobraunach@hotmail.com

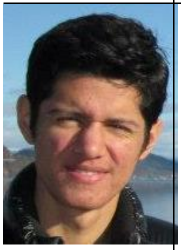

Christian PONCE

Employment

PhD student. Department of Physical Education and Sport, Faculty of Sport Sciences, University of Granada, Granada, Spain.

Degree

MSc

Research interests

Physical Activity and health.

E-mail: christian.natacion@gmail.com

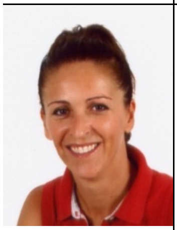

Belén FERICHE Employment

Lecturer in Sport Training. Department of Physical Education and Sport, University of Granada, Spain.

Degree

PhD

Research interests

Sport training, training assessment, altitude training.

E-mail: mbelen@ugr.es

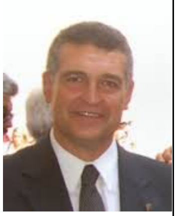

Paulino PADIAL

Employment

Lecturer in Sport Training. Department of Physical Education and Sport, University of Granada, Spain.

Degree

PhD

Research interests

Strength training, sport performance and training in combat sports.

E-mail: ppadial@ugr.es

✉ **Paulino Padial**

Department of Physical Education and Sport, Faculty of Sport Sciences, University of Granada, Granada, Spain
