# Supplementary material for: Effects of elastic band resistance training on the physical and mental health of elderly individuals: A mixed methods systematic review
Source: PLoS One. 2024 May 13;19(5):e0303372. doi: 10.1371/journal.pone.0303372 (PMC11090353; doi:10.1371/journal.pone.0303372)
Supplement: S1 File — (ZIP) [file pone.0303372.s001.zip › Supporting Information/Included study 60.pdf]

J. Phys. Ther. Sci.
27: 989–992, 2015

Original Article

Effect of exercise performance by elderly women on balance ability and muscle function

Hyo-Cheol Lee, PhD[1], Mi Lim Lee, PhD[2], Seon-Rye Kim, PhD[3]*

[1] Department of Emergency Medical Services, College of Health Science, Honam University, Republic of Korea
[2] Department of Dental Hygiene, College of Health Science, Honam University, Republic of Korea
[3] Department of Pharmacy, College of Pharmacy, Chungnam National University: 99 Daehakro, Yuseonggu, Daejeon 305-764, Republic of Korea

Abstract. [Purpose] The aim of this study was to investigate the effect of an exercise intervention on the balance ability and muscle function of elderly women. [Subjects] The subjects were randomly divided into a control group (n=10) and an exercise group (n=10). [Methods] The subjects participated in an elastic band exercise program lasting for 8 weeks, exercising for 40 minutes, four days a week with resting terms of 60 sec. Subjects used a band corresponding to 60% of the strength of the color band with which repetitive exercise was possible up to twelve times. The subjects performed elastic band exercises, with variations to the number of band exercises according to the improvement of their physical fitness levels. When fifteen repetitive elastic band exercises could be performed with no damage of the body, we changed the band for one which was one level up from the former one and subjects used the same band for the upper body and lower body. [Results] Leg muscular strength measured as sit-stand repetitions in 30 s significantly increased in the exercise group after the intervention compared to before the intervention. Leg muscular endurance measured as the number of knee ups in 2 minutes significantly increased in the exercise group after the intervention compared to before the intervention. Balance measured by one-leg standing time with the eyes open significantly improved in the exercise group after the intervention compared to before the intervention. [Conclusion] Balance ability and muscle function significantly improved in the exercise group and showing that the intervention is effective at improving balance, muscle strength, and muscle endurance of elderly women.
Key words: Balance ability, Muscle function, Exercise therapy

(This article was submitted Sep. 17, 2014, and was accepted Nov. 28, 2014)

INTRODUCTION

Since 1970, the proportion of the elderly population in Korea aged 65 and over has been on the increase, rising from 3.1%, and account for 12.2% of the population in 2013. It is anticipated that this proportion will account for 24.3% by 2030, and increase to 37.4% of the population in 2050. Importantly, it is expected that the proportion of the super elderly population over 85 will increase to 2.5% in 2030, and 7.7% in 2050, from 0.9% in 2013[1].

In Korea, compared with major advanced countries, the aging rate and dependency ratio of old age were low in 2010, but they will eventually reach high levels, comparable to Japan.

While it is expected that period of transition of elderly population ratio from 7% to 14% will take 18 years, this is a shorter time than the 115 years for France, 75 years for the United States, 75 years for the United Kingdom, and 24 years for Japan[1].

Sarcopenia occurs with the progress of aging[2]. Although sarcopenia seems not to be directly related to medical expenses, it reduces independent living and causes declines in fitness and activity, and increases the risk of injury from falls, because it is directly related to muscular strength. It is also related to physical disability and it is associated with chronic degenerative diseases such as diabetes, obesity, hyperlipidemia, hypertension. Finally, sarcopenia is a factor affecting the mortality rate[3]. Preventing sarcopenia is important for maintaining vibrant aging through independent living. Various kinds of intervention should be tried, even though they are not directly reflected in medical expenses, because preventing sarcopenia is an objective for preventing chronic degenerative and adult diseases[1]. The elderly population has a higher risk of injury during exercise than younger age groups. Although resistance exercise is recommended for the prevention and treatment of sarcopenia, the potential for injury should always be considered[5].

Flexibility is an important function which is related to the daily life of the elderly population being a factor of health-related and active fitness. Although women tend to be fatter and have less muscular strength, muscular endurance, and cardiovascular endurance than men, women are generally

*Corresponding author. Seon-Rye Kim (E-mail: sjsanj@hanmail.net)

©2015 The Society of Physical Therapy Science. Published by IPEC Inc. This is an open-access article distributed under the terms of the Creative Commons Attribution Non-Commercial No Derivatives (by-nc-nd) License <http://creativecommons.org/licenses/by-nc-nd/3.0/>.

more flexible. Because activities of daily living vary with health-related fitness[6], a fitness improvement exercise program is needed. Also, loss of equilibrium sense due to decline in lower leg proprioception, slowdown in information delivery between the central and peripheral nervous systems due to myoatrophy, and weakness of muscular strength lead to injuries from falls[5].

Previous studies of elderly women have reported that exercise with Thera-bands improves fitness and body composition[7], and that aerobic exercise is an effective fitness improvement method for the elderly population[8]. Shin et al. proved that there was improved effectiveness of leg muscular endurance, equilibrium, and gait function[9], and Hwang reported significant results, showing that elastic band exercise improves dynamic balance, dexterity, muscular strength, and muscular endurance[10].

Accordingly, exercises with elastic bands are considered important exercises because they can improve gait function and to reinforce muscle function.

In this study, we investigated the effect of elastic band exercise on an elderly population who had problems with mobility and functional disorder, the purpose of this study was to examine the effect of elastic band exercise on the subjects gait function and muscular strength.

SUBJECTS AND METHODS

This study conducted a band exercise program for elderly women volunteers aged 60 and over, who were randomly divided into a control group (n=10) and an exercise group (n=10) (Table 1).

Before the program, we explained the purpose and contents of the exercises to the subjects in detail and they participated in the exercise after signing a consent form. The inclusion criteria were: independent gait, absence of surgical symptoms, and voluntary consent to participation in the study. All experiments were reviewed and approved by a committee of Honam University.

Subjects participated in the elastic band exercise program for 8 weeks, exercising for 40 minutes, four days a week with resting terms of 60sec. Subjects used a band corresponding to 60% of the strength of the color band with which repetitive exercise was possible up to twelve times. This study used elastic bands of 6 inch width, which were developed by the American Physical Therapy Association[11], whose extension percentage is designated by color. This study conducted elastic band exercises, using various band exercises according to the improvement of the physical fitness levels of the subjects.

When the elastic band exercise could be repeated 15 times without exceeding physiological limits, we changed the band for one which had an extension percentage one level from the former one, and we used the same band for the upper body and lower body exercises.

During the first six weeks, the exercises were conducted using band strengths corresponding to 50–60% of HRmax and a rate of perceived exertion (RPE) of 11–12. During the last two weeks, the exercises were conducted using bands corresponding to 60–70% of HRmax and an PRE of 12–13.

The exercise program was began with warm up and ended with cooling down program designed for the weak elderly population, which were modified and supplemented with static stretching exercise. The subjects stood still and slowly and smoothly stretched in one posture for 30–60 seconds, the stretches were conducted in a range that did not cause pain.

Table 1. Characteristics of the subjects

Group	Age (years)	Height (cm)	Weight (kg)
Exercise	74±4.6	150.4±6.5	50.4±8.4
Control	73±6.4	148.7±6.2	51.7±5.4

The main exercise consisted of a revised elastic band core exercise designed for elderly women[12]. The initial exercise level was an RPE 11–13 in consideration of the fitness characteristics of the subjects, and before exercise we measured the stable heart rate and calculated individual target heart rates using Karvonen's formula. Heart rate was monitored by checking the exercise heart rate frequently in order to prevent the heart rate exceeding 50–70% of the maximal heart rate.

The exercises were implemented using a range of motion (ROM) within which the subjects didn't feel pain. The standard of main exercise with the band was 3 sets, without demanding immoderate postures causing injury. A rest of a minute and half was instituted between each set, and two assistants other than the leader of the exercises helped the elderly subjects. During the 8-weeks, intervention physical activities other than the program were restricted, but restriction of diet was not implemented. Warm up consisted of stretching and breathing for 3–5 minutes.

The main exercise was elastic band exercise for 30 minutes. Details of the exercise program were: pair-gymnastics, upper-extremity and elbow flexion/extension, upright row, side band, leg press, knee extension, ankle plantar-flexion, rhythmical gymnastics, movement learning, and repeated practice. Cool down consisted of stretching and breathing for 3–5 minutes.

Sit to stand is a method of measuring leg muscular strength. The subjects sat on a chair with their arms crossed over their chests. After the start signal, the subjects stood from the sitting position and sat down again repeatedly for 30 seconds and the number of times they could stand from the sitting position was measured[13].

Knees-up for 2 minutes is a method of evaluating leg muscular endurance. Subjects bend and raise their knees until the knee and the iliac crest are in line

The Subjects raised their knees alternately, except when they didn't raise the knee to the iliac crest, which was not counted. Subjects were asked to rate the degree of difficulty to prevent excessive effort during measurement and measurement was ended when subjects were unable to raise their knees. The total number of knee raises the iliac crest were recorded for each individual[14].

Standing on one leg with the eyes open was used to evaluate balance. The subjects selected the most comfortable leg, after experimentally standing on one leg with the eyes open with both legs. The times for which subjects could stand with

their hands on their waists with one leg raised and the sole of that foot placed on the inside of the knee was measured. If the hands fell from the waist or the leg or the body moved, measurement was stopped. Measurement was implemented twice. The smallest unit of record was 0.1 seconds and the highest record was 60 seconds[15].

All data was analyzed using SPSS/PC. Ver. 18.0 for Windows program t-test which uses the preliminary value as the covariate was used to test the significance of the differences between the measured values after the exercise program. Statistical significance in all analyses was accepted for values of $p<0.05$.

Table 2. The changes in balance ability and muscle function

Group		Pre	Post
Sit-to-stand	Exercise	7.1±3.5	14.4±5.8*
	Control	8.3±2.1	19.6±6.3
Knee-up	Exercise	40.9±6.7	59.3±9.8*
	Control	45.5±6.9	48.9±7.9
One leg with eyes open (sec)	Exercise	2.9±1.7	4.7±2.1*
	Control	1.6±4.8	1.8±8.9

*Values are mean±SD. *Significantly different between pre-and post-intervention

RESULTS

Leg muscular strength of the quadriceps femoris muscle measured as the number of repetitions of sit-to-stand in 30s significantly increased in the exercise group after the exercise intervention compared with leg muscular strength before the exercise intervention, as determined by the paired sample t-test ($p<0.05$).

Leg muscular endurance measured as the number of knee raises in 2 minutes significantly increased in the exercise group after the exercise intervention compared with leg muscular endurance before the exercise intervention, as determined by the paired sample t-test ($p<0.05$).

Balance measured as the time standing on one leg with the eyes open significantly increased in the exercise group after the exercise intervention compared with balance before the exercise intervention, as determined by the paired sample t-test ($p<0.05$) (Table 2).

DISCUSSION

Elastic band exercise is extensively used at home, parks, and welfare centers because it is easy and safe to use by anyone without restriction of space. Especially, elastic band exercise has received attention because it reduces the risk of injury in the elderly population which has a relatively high risk of injury and it is effective at maximizing body function and improving of health-related-fitness[16].

The purpose of this study was to examine the effects of elastic band exercise on leg muscular strength and balance, and the usefulness of elastic band exercise for the effectiveness and durability of factors related to fitness reinforcement and prevention of disease.

The results of this study confirmed that elastic band exercise is a good exercise for improving muscular strength and fitness and their effective maintenance.

The aim of this study was the improvement of muscular strength. However, elastic band exercise positively influences whole body function factors such as cardiovascular endurance, flexibility of the upper body and waist, dexterity, dynamic balance. It suggests that elastic band exercise leads to improvement of whole body function (fitness) as shown by the effects of aerobic exercise to elderly people. Through the study of elastic band exercise applied with further variations, we should conduct a study that shows elastic band exercise can be a method that improves aerobic exercise ability.

It was reported that continuous activity for a long time keeps and improves changes in body composition[17]. Because the exercise program of this study involved physical activity moving the whole body with a band for 40 minutes or more, continuous movement would have led to a reduction of percentage of body fat, reducing fat free percentage, and maintenance of fat free percentage.

Basic physical fitness is necessary for the free activity of the elderly population, and regular exercise is effective at improving physical fitness. Lee suggested regular exercise and physical activity as a method for the improvement and enhancement of fitness related to the health of the elderly population, improving their resistance to disease, and preventing disease.[18] Chang et al. reported that leg muscular strength and gait ability is improved and knee pain was relieved after 8 weeks of moderate intensity lower body exercise using an elastic band performed by elderly women with arthritis[19].

Stretching and resistance exercises have been shown to improve the balance ability of elderly adults[20].

Resistance exercises using the Thera-band are simple and economical, and have safety advantages. They are generally used for rehabilitation purposes[21] because training can be selected case by case with control over the loading intensity.

Several studies have reported that strengthening exercise using the Thera-band for the lower extremities improves balance ability[22]. Therefore, strengthening exercise with a Thera-band is a suitable home-based exercise program for improving the balance needed for activities of daily living of elderly adults.

This study used sit-to-stand, knees up, and balancing on one leg with the eyes open to evaluate fitness. Sit-to-stand and knees up are items used to evaluate leg muscular strength and muscular endurance, which are abilities closely connected with self reliance of physical activity such as whole body movement, and change of direction. Enhancing leg muscular strength through regular exercise is very important for the maintenance and improvement of movement ability because leg muscular strength declines during the process of aging.

Elastic band exercise over an 8-week exercise period improved and maintained fitness, unlike in a study of young adults which found only just maintenance of strength after an 8 week exercise period, and we expect that further study will be carried out.

REFERENCES

1) National Statistical Office, 2006.
2) Rosenberg IH: Epidemiologic and methodologic problems in determining nutritional status of older person. Am J Clin Nutr, 1989, 50: 1121–1123.
3) Nair KS: Aging muscle. Am J Clin Nutr, 2005, 81: 953–963. [Medline]
4) National Statistical Office, 2013.
5) Perrin PP, Gauchard GC, Perrot C, et al.: Effects of physical and sporting activities on balance control in elderly people. Br J Sports Med, 1999, 33: 121–126. [Medline] [CrossRef]
6) Yuh BK: Effects of swimming on physical fitness in adult women. Kor J Sports Sci, 2000, 9: 603–623.
7) Kim CS, Park IH, Kim MW, et al.: Effects of exercise using Thera band on body compositions, blood pressure and physical fitness in the elderly women. J Muscle Jt Health, 2007, 14: 158–168.
8) Park IR: Effects of 12 weeks aerobic exercise on health-related physical fitness and bone density in elderly. J Sport Leis Stud, 2004, 22: 459–469.
9) Shin SM, Ahn NY, Kim KJ: Effect of resistance training with elastic band on the improvement of balance and gait in the elderly women. Kor J Growth Dev, 2006, 14: 45–56.
10) Hwang BY: Effect of resistance excercise using elastic band on functional fitness and body composition in elderly women. Unpublished master's thesis, Kookmin University, 2006.
11) American Physical Therapy Association, 2008.
12) Kim SH: Effects of 12 weeks core exercise to range of motion and isokinetic muscle functions of elderly women. J Kor Phys Edu Asso Girl Wom, 2012, 26: 145–156.
13) Choi SW, Lee JS, Ku HJ, et al.: Effects of resistive and balance training on walking patterns in the falls experienced elderly women. Kor J Phys Edu, 2005, 44: 287–295.
14) Kim HS, Tanaka K: The assessment of functional age using "Activities of Daily Living" performance test—A study of Korea women—. J Aging Phys Act, 1995, 3: 39–53.
15) Park WY: Effects of 16 week physical training on equilibrium sensory and motor control function of old adults. Kor Sport Res, 2004, 15: 1948–1954.
16) Phillip P, Todd S: The Scientific and Clinical Application of Elastic Resistance. Human Kinetics Publishers, 2003.
17) Kobayashi K, Hiiragi Y, Maruyama H: Development of the "10-second Open-Close Stepping Test" (OCS-10) and fundamental study of its measurement values through a comparison of healthy young people and community-dwelling elderly. J Phys Ther Sci, 2012, 24: 747–749. [CrossRef]
18) Lee MS: Item selection and assessment of daily living-related functional fitness for healthy aging in Korean elderly. Korean J Phys Educ, 2003, 42: 541–550.
19) Chang TF, Liou TH, Chen CH, et al.: Effects of elastic-band exercise on lower-extremity function among female patients with osteoarthritis of the knee. Disabil Rehabil, 2012, 34: 1727–1735. [Medline] [CrossRef]
20) Bird ML, Hill K, Ball M, et al.: Effects of resistance- and flexibility-exercise interventions on balance and related measures in older adults. J Aging Phys Act, 2009, 17: 444–454. [Medline]
21) Cheema B, Abas H, Smith B, et al.: Randomized controlled trial of intradialytic resistance training to target muscle wasting in ESRD: the progressive exercise for anabolism in kidney disease (PEAK) study. Am J Kidney Dis, 2007, 50: 574–584. [Medline] [CrossRef]
22) Yu W, An C, Kang H: Effects of resistance exercise using Thera-band on balance of elderly adults: a randomized controlled trial. J Phys Ther Sci, 2013, 25: 1471–1473. [Medline] [CrossRef]
