# Supplementary material for: Effects of elastic band resistance training on the physical and mental health of elderly individuals: A mixed methods systematic review
Source: PLoS One. 2024 May 13;19(5):e0303372. doi: 10.1371/journal.pone.0303372 (PMC11090353; doi:10.1371/journal.pone.0303372)
Supplement: S1 File — (ZIP) [file pone.0303372.s001.zip › Supporting Information/Included study 39.pdf]

J. Phys. Ther. Sci.
27: 2061–2063, 2015

Original Article

The effects of a community-centered muscle strengthening exercise program using an elastic band on the physical abilities and quality of life of the rural elderly

So-Yeon Park, OT, PhD[1)], Jin-Kyung Kim, OT, PhD[1)*], Seong-A Lee, OT, PhD[2)]

[1)] *Department of Occupational Therapy, College of Health Science, Hanseo University: 46 Hanseo 1-ro, Hami-Myun, Seosan-si, Chungcheongnam-do, Republic of Korea*
[2)] *Department of Occupational Therapy, College of Medical Science, Soonchunhyang University, Republic of Korea*

Abstract. [Purpose] The purpose of this study was to examine the effects of a muscle strengthening exercise program using an elastic band on changes in the physical abilities and quality of life of the rural elderly. [Subjects] The subjects of this study were 46 elderly people (8 males, 38 females) aged 65 or older, who lived in a rural area and managed their daily lives independently. [Methods] The study's exercise program was conducted 16 times for 80 minutes each session over an eight-week period. This program consisted of several exercises to strengthen muscular endurance and improve balance ability based on exercises using Thera-bands. The physical abilities of the subjects were divided into muscular endurance, upper-extremity flexibility, balance, and low-extremity agility. Each ability was measured to compare the effects of the exercise program. In addition, the Korean version of the World Health Organization Quality of Life (WHOQOL)-BREF questionnaire was used to examine changes in the subjects' quality of life. [Results] The subjects showed improvements in muscular endurance, balance, and low-extremity agility. They also exhibited an overall statistically significant improvement in quality of life scores after the exercise program. In terms of the main items, changes were observed in the areas of psychological relations, social relations, and environment. [Conclusion] The community-centered muscle strengthening exercise program using the elastic band was found to improve muscular endurance, balance, agility, and quality of life of rural elderly subjects.
Key words: Elderly, Physical ability, Quality of life

(This article was submitted Feb. 19, 2015, and was accepted Mar. 17, 2015)

INTRODUCTION

Population aging has increased public interest in maintaining a healthy and high-quality life[1)]. Methods to prevent the functional disorders of the elderly, lengthen the span of their healthy lives, and reduce their social and economic burdens are needed[2, 3)]. In recent years, customized exercise methods for the elderly, which can maintain and improve appropriate muscular strength, muscular endurance, and cardiopulmonary functions, have drawn attention as opposed to passive methods, such as medication[4, 5)]. However, the rural elderly show low rates of participation in sports activities due to poor access to sports centers or other amenities that provide exercise programs, and heavy economic burdens. In particular, the rural elderly often experience physical pain in the back and knees due to farming for long periods. Therefore, opportunities are urgently required to encourage them to improve their physical abilities and quality of life[6–8)]. The purpose of this study was to examine the effects of regular exercise on changes in the physical functions and quality of life of the rural elderly by designing an exercise program for them that is appropriate for their physical condition and can be easily performed at home.

SUBJECTS AND METHODS

The subjects of this study were 46 elderly people (8 males, 38 females) aged 65 or older, who resided in a rural area. On average, the subjects were 73.41±8.77 years of age and had engaged in farming for 36.08±15.73 years. Selection criteria were: subjects aged 65 or older who managed their daily lives independently, who were capable of verbal communication, and had the cognitive ability to listen to and follow explanations about the exercises. Before participation, the procedures, risks, and benefits were explained to all the participants, who gave their informed consent. The participants' rights were protected according to the guidelines of the University of Hanseo.

The exercise program was conducted 16 times in 80 minute sessions over an eight-week period from June 25

*Corresponding author. Jin-Kyung Kim (E-mail: k6j4k@hanseo.ac.kr)

©2015 The Society of Physical Therapy Science. Published by IPEC Inc.
This is an open-access article distributed under the terms of the Creative Commons Attribution Non-Commercial No Derivatives (by-nc-nd) License <http://creativecommons.org/licenses/by-nc-nd/3.0/>.

Table 1. A comparison of the physical abilities of the subjects before and after the exercises (n=46)

Items		Exercise program	
		Pre-training	Post-training
Strengthen muscular endurance	Arm curl-dominant side (times)	46.3±22.1	64.8±26.3*
	Sitting and standing up from a chair (times)	39.7±18.4	49.7±23.4*
	2-Minute step in place test (times)	94.9±37.0	130.0±41.2*
Balance	One-leg standing- eyes open (sec)	19.6±21.1	27.3±24.2*
	One-leg standing- eyes closed (sec)	5.3±6.8	8.4±10.5*
	Functional reach- dominant side (mm)	302.8±92.4	372.9±73.8*
Upper-extremity flexibility	Back scratch (mm)	147.7±116.8	132.8±110.9
Lower-extremity agility	Timed up and go (sec)	9.8±2.8	9.2±3.5
	Foot stepping (times)	21.4±7.3	24.7±8.4*
	Tandem walk (sec)	9.9±4.6	9.0±4.6

[a]Mean±SD, *p<0.05

to August 20. The exercise program consisted of exercises to improve upper and lower-extremity muscular endurance and balance based on exercises using Thera-bands. The program was conducted in a local community hall and included a warm-up of ten minutes, main exercises lasting 65 minutes, and a cool-down of five minutes. Among the senior fitness test items for the elderly developed by Rikli and Jones, arm curls, 30-second standing up from a sitting position on a chair, and two-minute step in place test were used to evaluate the physical abilities of the participants[9]. In addition, the distance between the two hands of each subject was measured during his/her back scratching to measure flexibility. One-leg standing with the eyes open and closed, and functional reach were also measured to assess balance. In addition, the timed up and go test, tandem walk test, and foot stepping were performed to examine lower-extremity agility[10]. The Korean version of the World Health Organization Quality of Life (WHOQOL)-BREF questionnaire was used to assess the effects on quality of life of the changes in the physical abilities of the elderly[11]. The evaluation items were divided into four main areas of physical health, psychological relations, social relations, and environment, which were further segmented into 26 lower-level items. Quality of life scores were measured and recorded based on the subjects' subjective perceptions of their quality of life in the 26 lower-level items.

The experimental results were statistically analyzed using SPSS 22.0 KO (IBM, IL, USA). Frequency analysis and descriptive statistics were employed to identify the general characteristics of the subjects. Changes in the physical abilities and the quality of life after completion of the exercise program were analyzed using the paired t-test with a statistical significance level of $\alpha= 0.05$.

RESULTS

After the intervention the physical abilities of the subjects were compared with their pre-intervention values. It was found that muscular endurance showed improvements in arm curls, sitting and standing up from a chair, and two-minute step in place test, and balance ability (one-leg standing, functional reach) and agility (foot stepping) also improved (p<0.05) (Table 1). The overall quality of life score increased by 7.31 points after the intervention and more specifically the scores of psychological and social relations, and environment exhibited statistically significant improvements after the exercises (p<0.05) (Table 2).

Table 2. A comparison of the quality of life of the subjects before and after the exercises (n=46)

Domain	Exercise program	
	Pre-training	Post-training
Physical health	20.4±5.4[a]	21.4±5.9
Psychological	17.8±4.5	19.8±3.9*
Social relationships	10.3±1.6	11.2±1.7*
Environment	23.6±4.7	26.9±3.9*
Total	72.0±13.4	79.4±12.8*

[a]Mean±SD, *p<0.05

DISCUSSION

This study conducted an exercise program for the rural elderly two times each week over an eight-week period. The resistance exercises using an elastic band effectively improved the physical abilities and the quality of life of the rural elderly. After the exercises, the subjects showed improvements in their physical abilities: left and right upper-extremity arm curls showed improvements, and the score for sitting and standing up from a chair also increased. In addition, lower-extremity balance was enhanced, as shown by improvements in the two-minute step in place test, one-leg standing time with eyes open, and functional reach. Foot stepping also improved. The above results agreed with the outcome of the study by So et al.[12], in which dumbbell exercises were performed by the elderly for 12 weeks, twice each week. They reported statistically significant improvements in the two-minute step in place test, sitting and standing up from a chair, and 244 cm walking back and forth after the exercises. The present study found there were statistically significant improvements in arm curls, sitting and standing up from a chair, two-minute step in place test, one-leg stand-

ing time, functional reach, and foot stepping. Therefore, the subjects may have improved their upper-extremity muscular endurance, general endurance, balance, and agility. The results were also similar to the findings of the study by Yu et al.[13], in which resistance exercises were performed using an elastic band for 12 weeks, three times each week, leading to improvements in muscular endurance (sitting and standing up from a chair) and balance (functional reach). However, the results were contrary to those of the study by Kazumasa et al.[14], in which an easy and simple resistance exercise program was implemented for the elderly of a local community. The eight-week exercise program conducted in the present study was found to be effective at enhancing the physical functions of the elderly.

The assessment of changes in the quality of life using the WHOQOL-BREF questionnaire before and after the exercises found there was an overall improvement in QOL after the intrvention. In particular, the quality of life scores in the areas of psychological and social relations, and environment improved after the intervention. However, although the quality of life score in the area of physical health was improved after the exercises, this result was not statistically significant. The exercise program employed in this study was conducted in a community hall to increase the social participation and performance of the elderly. Therefore, the participation of the elderly in physical activities such as flexibility exercises performed to their favorite music and muscle strengthening exercises may have influenced their psychological and social relations and quality of life. The results of the present study may support the conclusions of existing research that the participation of the elderly in exercise programs is effective at changing their psychological and social relations.

The present study had the limitation that it targeted elderly people in a rural area, and thus the results cannot be generalized to all age groups or elderly people. Despite this, this study confirmed that an exercise program was effective at changing the physical functions and the quality of life of the rural elderly. Most of the elderly in rural areas are facing threats to their health, such as musculoskeletal diseases, due to their lifestyle of farming for long periods of time. Moreover, they have fewer opportunities to participate in exercise programs than do the elderly in urban areas. In this regard, based on the findings of the present study, more diverse studies should be carried out in the future to help the rural elderly improve their physical functions and social participation.

REFERENCES

1) Okubo Y, Osuka Y, Jung S, et al.: Walking can be more effective than balance training in fall prevention among community-dwelling older adults. Geriatr Gerontol Int, 2015. [Medline] [CrossRef]
2) Campbell AJ, Borrie MJ, Spears GF: Risk factors for falls in a community-based prospective study of people 70 years and older. J Gerontol, 1989, 44: M112–M117. [Medline] [CrossRef]
3) Pa J, Goodson W, Bloch A, et al.: Effect of exercise and cognitive activity on self-reported sleep quality in community-dwelling older adults with cognitive complaints: a randomized controlled trial. J Am Geriatr Soc, 2014, 62: 2319–2326. [Medline] [CrossRef]
4) Watanabe Y, Tanimoto M, Oba N, et al.: Effect of resistance training using bodyweight in the elderly: Comparison of resistance exercise movement between slow and normal speed movement. Geriatr Gerontol Int, 2015, ▪▪▪: 17. [Medline]
5) Rejeski WJ, Mihalko SL: Physical activity and quality of life in older adults. J Gerontol A Biol Sci Med Sci, 2001, 56: 23–35. [Medline] [CrossRef]
6) Chodzko-Zajko WJ, Proctor DN, Fiatarone Singh MA, et al. American College of Sports Medicine: American College of Sports Medicine position stand. Exercise and physical activity for older adults. Med Sci Sports Exerc, 2009, 41: 1510–1530. [Medline] [CrossRef]
7) Unsworth J: Falls in older people: the role of assessment in prevention and care. Br J Community Nurs, 2003, 8: 256–262. [Medline] [CrossRef]
8) Whitehurst MA, Johnson BL, Parker CM, et al.: The benefits of a functional exercise circuit for older adults. J Strength Cond Res, 2005, 19: 647–651. [Medline]
9) Rikli RE, Jones CJ: Development and validation of criterion-referenced clinically relevant fitness standards for maintaining physical independence in later years. Gerontologist, 2013, 53: 255–267. [Medline] [CrossRef]
10) Rikli RE, Jones CJ: Senior fitness test manual. Human Kinetics Publishers Inc.
11) Min SK, Lee CI, Kim KI, et al.: Development of Korean version of WHO quality of life scale abbreviated version (WHO-QOL-BREF). J Korean Neuropsychiatr Assoc, 2000, 39: 571–579.
12) So WY, Song MS, Cho BL, et al.: The effects of treatment dumbbell exercise on body composition, fitness, and blood lipid profiles in sarcopenic elderly. J Korea Gerontological Soc, 2009, 29: 837–850.
13) Yu W, An C, Kang H: Effects of resistance using thera-band on balance of elderly adults: a randomized controlled trial. J Phys Ther Sci, 2013, 25: 1471–1473. [Medline] [CrossRef]
14) Nakagawa K, Inomata N, Nakazawa R, et al.: The effect of a health promotion program consisting of easy and simple exercises for community living elderly adults. J Phys Ther Sci, 2007, 19: 235–242. [CrossRef]
