# Supplementary material for: Effects of elastic band resistance training on the physical and mental health of elderly individuals: A mixed methods systematic review
Source: PLoS One. 2024 May 13;19(5):e0303372. doi: 10.1371/journal.pone.0303372 (PMC11090353; doi:10.1371/journal.pone.0303372)
Supplement: S1 File — (ZIP) [file pone.0303372.s001.zip › Supporting Information/Included study 45.pdf]

REJUVENATION RESEARCH
Volume 20, Number 5, 2017
© Mary Ann Liebert, Inc.
DOI: 10.1089/rej.2017.1924

Effects of Three Different Chair-Based Exercise Programs on People Older Than 80 Years

José M. Cancela Carral,[1,2] Estrella Pallin,[2] Ander Orbegozo,[2] and Carlos Ayán Pérez[1,2]

Downloaded by Seoul National Univ. Medical College from online.liebertpub.com at 11/18/17. For personal use only.

Abstract

This study aimed at comparing the effects of three chair-based exercise programs on people older than 80 years. Thirty-six participants (87.91 ± 4.70 years) were randomly allocated to an aerobic, muscular resistance, or joint mobility exercise program. The participants exercised 3 days per week during 3 months. A hand-held dynamometer, the Tinetti Gait Balance, the Barthel Index, and the Timed Up and Go Test (TUG) (assessed by means of the Wiva® science sensor) were used to evaluate the effects of the programs on the participants' strength, balance, functional independence, and functional mobility, respectively. After the intervention, it was observed that only the elastic-band program resulted in significant improvements in strength and balance. These results imply that when choosing a low-cost exercise program for very old people, the use of elastic bands stands as a far better option than pedaling on a pedal exerciser or performing mobility exercises.

Keywords: frail, elderly, exercise, balance, independence

Aging involves a decline in the physical condition of elderly people and eventually, it has an impact on their health, functional independence, and physiological function.[1] This is especially true among those older than the age of 80, often referred to as a "very old population,"[2] since they suffer significant losses in their levels of strength,[3] as well as a significant increase in their risk of falling down.[4] In this respect, following a healthy lifestyle that includes physical activities has been advised as a palliative measure for this age group.[5] Indeed, it has been noticed that very old people not only can take part safely in physical training programs but also can experiment with positive changes in some aspects that determine their functional independence, such as muscle strength, or balance.[6,7]

In any case, when it comes to promoting physical activity habits among very old people, it is necessary to take into account some special considerations regarding their old age.[2] It has been put forward that one of the essential factors to act on this population is adherence to treatment.[8] For example, it has been noticed that the economic status and the level of functional ability are two factors that clearly determine the level of attendance of very old people to physical exercise programs.[9] So, there are some things that not every one can afford, such as paying a coach, a gym membership, or the fare to the gym. Indeed, financial costs have been previously identified as a barrier for exercising in elderly populations.[8] Similarly, a higher number of comorbidities and chronic diseases has been shown to correlate to a lower adherence of subjects to an exercise program.[10] This finding implies that worse health negatively affects the way daily activities are carried out, which, in turn, affects functional performance and ability. That is, the poorer the health is, the less ability a person has to be independent, and the less active he or she is. Therefore, functional ability has been identified as an impediment to adherence with physical exercise programs in the elderly.[11] Consequently, it seems relevant to design and discuss the effects of performing exercise programs that consider both factors as a way of increasing the adherence of this population to the practice of physical activity.

On the one hand, these physical exercise programs should be low cost. On the other hand, they should include easy-to-perform tasks that do not demand a high level of functional ability from the performer. Regarding the first aspect, it should be possible to carry out the training programs with low-cost materials that are easy to transport and store, thus preventing the need to attend sport centers or pay membership fees to join them, since this type of materials would allow them to undertake those programs in the comfort of their homes.

To this effect, pedal exercisers or Thera-Bands® are an interesting alternative as they fulfil the previous requirements.[12]

[1]HealthyFit Research Group, University of Vigo, Galicia Sur Health Research Institute (IIS Galicia Sur), Sergas-UVIGO, Pontevedra, Spain.
[2]Faculty of Education and Sport Sciences, Sport Sciences, University of Vigo, Pontevedra, Spain.

Downloaded by Seoul National Univ. Medical College from online.liebertpub.com at 11/18/17. For personal use only.

In relation to the proposal of exercises that can be done by most very old people (even though they have certain health constraints in their functional ability), the practice of chair-based exercise (CBE) can initially be considered a suitable solution.[13] This modality of training is primarily a seated exercise program that is specifically designed for frail old people and that can be used as a starting point to progress to standing programs.[14] Actually, scientific literature on the benefits of CBE on very old people is limited; we have noted that more studies are needed on this particular aspect.[15] For instance, the vast majority of randomized studies carried out about the effects of CBE in very old people has been focused on analyzing the impact of a single intervention[11,16] but, to the authors' knowledge, no study so far has aimed at comparing the effects of different CBE training protocols on very old people. Under these circumstances, the aim of this study was to analyze the effects of three low-cost CBE programs on the strength, balance, functional independence, and functional mobility of people older than 80 years.

Materials and Methods

Participants

Participants in this study were recruited through an invitation letter sent to three day care centers for the elderly located in a city in the northwest of the Iberian Peninsula (Galicia). The inclusion criteria to take part in the study were: (1) to be considered ''very old people'' (to be 80 or more years of age)[2] and (2) to be able to follow simple instructions. The presence of unstable cardiovascular, metabolic, or neuromuscular conditions, as well as any other chronic disease that could limit training (*i.e.*, hemiparetic disease, congestive heart failure, uncontrolled sever hypertension ...) or testing were considered exclusion factors. Before the study was performed, informed consent was requested from the participants or their relatives or caregivers, in those cases who were unable to themselves provide consent. The study was approved by the local ethics committee and registered as a clinical test (EudraCT: 2016-005059-25).

Intervention

Participants were randomly allocated to three groups by using IBM SPSS Statistics v. 22 for Mac's random sampling function. With this in mind, we used the function known as ''random sample of cases,'' and we assigned 33% of cases to group A. Next, we selected at random 50% of the remaining cases assigned to group B. All the other cases were assigned to group C.

Participants in group A took part in an aerobic exercise program, whereas participants in groups B and C carried out a muscle resistance and a joint mobility program, respectively. All three groups exercised three times a week in 45-minute sessions for 3 months. The training programs took place at the day care centers, and each group trained separately. All the exercises included in the programs were performed in chairs with armrests. The initial and final part of the sessions were the same for the three groups: 10 minutes doing breathing exercises as a part of the warm-up and 5 minutes doing passive muscle stretches to conclude the session.

In the main part, the participants in group A pedaled continuously for half an hour by using a variable resistance pedal exerciser (five levels) MRP290 model. During the first 5 minutes, the participants were allowed to pedal forward and backward at their discretion, carrying out the movement on a continuous basis. The trainer encouraged the participants to keep a steady pace as long as possible. The trainer was also told that if he noted signs of fatigue (excessive sweating, shortness of breath, discomfort when pedaling), he should make the participants slow down their pedaling pace until those signs disappeared. Resistance was set at level 1 for the first 2 weeks. During the next 5 weeks, it was set at level 2 and it went up to level 3 during the last 5 weeks. Participants carried a Polar Heart Rate S610I to record their heart rate (HR) achieved throughout each session. When all the sessions were finished, participants were asked to report the degree of dyspnea that they had felt, which was contrasted with the Spanish version of the modified Borg scale.[17]

Also in the main part, the participants in group B performed muscular-resistance exercises by using a yellow Thera-Band with level 3 elongation. The exercises were performed continuously, with no pauses between them, following the organization and dosage schedule shown in Table 1.

As for the participants in group C, their main part consisted in performing a joint mobility routine (two sets of three repetitions). They carried out mobilization exercises of shoulders (*i.e.*, lifting shoulders), neck (*i.e.*, neck rotation), elbow (*i.e.*, arm flexion; touching the opposite shoulder by crossing the arm over the breast), hands (*i.e.*, flexion and extension; opening and closing hands), hip (*i.e.*, lifting legs alternatively), knee (*i.e.*, flexion and extension; dragging feet), and ankle (*i.e.*, flexion and extension; inversion and eversion). Each exercise was done three times, with no repetitions and with no break between repetitions or between series. Once routine was over, lasting about 20 minutes approximately, they did exercises by using a foam ball during 20 minutes (*i.e.*, lifting the ball over head, throwing the ball at the wall, bouncing the ball between legs, *etc.*) based on basic exercises, rotating neck, clapping hands at chest height, opening and closing hands, raising legs, *etc.* All sessions were monitored by two specialists in physical exercise with previous experience of prescribing exercises to old people.

Assessments

Before the participants' initial assessment, their medical records were requested to gather information about their anthropometric characteristics and the state of their cognitive functioning, which was assessed with the Spanish version of the Mini Mental State Examination (MMSE).[18]

Adherence was defined as the number of sessions attended divided by the number of sessions offered. The number of sessions performed by the participants in each of the three groups was registered through an attendance checklist that was filled in by the person in charge.

To measure the effects of the interventions on strength, a SAEHAN Digital Hand Dynamometer was used. Measurement was performed with both the dominant and the nondominant hand while participants remained in a sitting position with their elbows flexed at 110°. Participants were asked to squeeze the dynamometer handles as hard as possible for 3 to 5 seconds, twice, with a 30-second rest interval between trials. Only the highest value in the series was recorded for analysis.

Downloaded by Seoul National Univ. Medical College from online.liebertpub.com at 11/18/17. For personal use only.

Table 1. Muscular Resistance Training Program (Group B)

		Weeks				
		1	*2*	*4*	*7*	*9*
		3 Sets of 6 repetitions 20" break	*3 Sets of 7 repetitions 20" break*	*3 Sets of 8 repetitions 20" break*	*3 Sets of 10 repetitions 20" break*	*3 Sets of 12 repetitions 20" break*
Upper limbs (sitting position)						
Shoulder	One-sided flexion	Stepping the rubber strip, upper limb flexion, extending elbow. Alternate both arms.				
	Unilateral flexion	Stepping the rubber strip, upper limb extension, slightly flexed elbow. Alternate both arms.				
	Unilateral abduction	Stepping the rubber strip with homolateral foot, adduction of oblique upper member in oblique axis				
	Bilateral abduction	Stepping the rubber strip with contraletral foot, abduction of upper member in oblique axis.				
Elbow	Bilateral flexo-extension	Stepping the rubber strip, elbow flexion with both arms at a time.				
Lower limbs (sitting position)						
Hip (rubber strip knotted around the medial third of the thigh)	Unilateral flexion	Leg elevation with flexed knee. Alternate both legs.				
	Bilateral abduction	Separate knees, opening and closing legs.				
	Unilateral abduction	Separate one leg, opening and closing. Alternate both legs.				
Knee (rubber strip knotted around the medial third of the leg)	Unilateral extension	Knee extension. Alternate both legs.				
Ankle (rubber strip gripped with both hands, around the foot sole)	Unilateral plantar flexion	Foot plantar flexion, pressing with both hands to act on rubber strip. Alternate both feet.				

The Spanish validated version of the Tinetti Gait and Balance test[19] was used to assess the effects of the interventions on the participants' stability. This adaptation of Tinetti includes a 16-item test to assess and score balance and gait individually. Balance is evaluated while sitting, arising, standing (immediate and prolonged), and turning. In addition, the maintenance of balance is tested against attempts at disruption (nudge) and without a horizon reference (eyes closed). The maximum score of the balance subscale is 16. Gait is evaluated while the patient walks across the room. Assessments include initiation of gait, step characteristics (height, length, symmetry, and continuity), path deviation, trunk stability, and walking stance. The maximum score of the gait subscale is 12. The Tinetti total score ranges from 0 to 28, with higher values indicating better stability.

The Spanish validated version of the Barthel Index (BI) was used to evaluate the impact of the program on the participants' functional independence.[20] This questionnaire includes 10 Likert-type questions about the patient's autonomy when performing daily activities. The score goes from 0 to 100, with higher values indicating higher levels of dependence.

The effect of the programs on the participants' functional mobility was assessed by means of the TUG.[21] To perform this test, the patient needs to rise from a chair, walk 3 m, turn around, walk back to the chair, and sit down. In this study, TUG performance was analyzed through Wiva® science sensors; wireless inertial sensing devices were set between vertebrae L4 and L5. Wiva includes an accelerometer, a magnetometer, and a gyroscope, which allow practitioners to record information about the angular velocities reached during TUG. In addition, Wiva provides data about the partial times obtained in the main phases of TUG (*Sit to stand*, *Gait to go*, *Turning*, *Gait return*, *Stand to sit*), as well as the total time needed to complete the task. All this information was recorded and sent to a PC via Bluetooth with Biomech Study 2011 v. 1.1. The assessments took place 1 week before the intervention started and 1 week after it finished, and they were carried out by the same professionals who monitored the intervention.

Statistical analysis

A descriptive analysis was performed by using central tendency measures to determine the characteristics of the sample assigned to each group of intervention. A Shapiro-Wilk test was applied to verify normal distribution. Once normality of distribution was checked, parametric tests were carried out in the second part of the analysis. A factorial ANOVA test was used to check the homogeneity of the sample. To evaluate the existence of differences between the three programs (aerobic, muscular resistance, joint mobility) and two moments (baseline, post-test), an analysis of variance was used (ANOVA 3×2). To identify the existence of clinically meaningful change as a result of the programs performed, the smallest real difference (SRD) and the standard error of measurement (SEM) were calculated. Measurement errors were quantified through the computation of SEM ($SEM = \text{standard deviation of all test-retest scores} \times \sqrt{1 - ICC}$). The SRD ($SRD = 1.96 \times SEM \times \sqrt{2}$) was used to determine whether the change score of an individual patient was real at the 95% confidence level.[22] In

Downloaded by Seoul National Univ. Medical College from online.liebertpub.com at 11/18/17. For personal use only.

Table 2. Participants' Baseline Characteristics

	Group A (n = *13*)		*Group B* (n = *12*)		*Group C* (n = *11*)	
	Mean ± SD	*Min–Max*	*Mean ± SD*	*Min–Max*	*Mean ± SD*	*Min–Max*
Age (years)	89.83 ± 5.29	82.00–96.00	84.92 ± 3.40	80.00–90.00	89.00 ± 5.43	82.00–96.00
Gender (% women)	84.61	—	91.66	—	63.63	—
Weight (kg)	63.77 ± 12.97	49.90–85.50	72.78 ± 12.29	46.10–91.00	62.89 ± 9.69	47.80–76.50
Height (cm)	153.78 ± 4.84	144.00.159.00	153.17 ± 5.92	140.00–163.00	155.50 ± 7.87	141.00–164.00
MMSE (pts)	19.08 ± 6.29	7.00–28.00	21.30 ± 4.74	14.00–28.00	18.75 ± 8.62	8.00–29.00
BMI (kg/m^2)	26.79 ± 3.98	21.23–33.99	30.95 ± 4.83	23.52–41.54	25.98 ± 5.44	19.22–33.25

BMI, body mass index; MMSE, Mini Mental State Examination; pts, points.

accordance with Dvir,[23] to indicate a clinically meaningful change at the group level—SRDg—the cut-off has to be smaller and has to be equal to 1.96 SEM. The SPSS statistics program v. 22 for Mac was used to perform all statistical analyses, with *p*-values lower than 0.05 being considered statistically significant.

Results

Out of the 50 people who began the research, 37 attended more than 80% of the sessions and provided useful data to be analyzed. The percentage of adherence was 80% (29 sessions), 86% (31 sessions), and 80% (30 sessions); it was 82%, 85%, and 83% for groups A, B, and C, respectively. One person in group C was excluded because he did not complete the final assessments, so the sample was finally composed of 36 people. Participants' characteristics, relative to the group they were assigned to, may be observed in Table 2. The average results achieved in MMSE showed the presence of cognitive impairment in each of the three groups under analysis. In group A, a mean HR of 83.01 and ±85.56 bpm was recorded in the first and last sessions, respectively (Fig. 1). Throughout the program, the degree of dyspnea was considered severe (6.26 ± 0.86 in the first sessions and 5.97 ± 0.53 in the last sessions).

Once the intervention ended (Table 3), the participants in group A experienced a significant improvement ($p < 0.05$) in the partial time needed to complete TUG phases *Gait to go* and *Gait return*. The participants in group B achieved statistically significant improvements in their muscular strength and in their balance level, as well as a significant reduction of partial time in the TUG phases *Sit to stand*, *Gait to go*, and *Gait return*. The participants in group C achieved a significant increase in the partial times of TUG phases *Sit to stand, Turning*, and *Stand to sit.* The analysis of the Moment × Program showed that the muscular resistance training program (group A) was significantly more effective than the aerobic training one (group B) when reducing TUG total time ($F_{2.34} = 4.196$; Sig = 0.001). Table 4 shows the effects of the intervention on biomechanical parameters analyzed through Wiva with respect to the performance in TUG. There were also significant improvements in peak flexion and extension angle (*Sit to stand* phase) and peak flexion angle (*Stand to sit* phase) in the participants of group A. Meanwhile, significant improvements were noticed among the participants of group B with regard to these same variables and six more. In group C, there was a significant worsening in the following variables: angular velocity, peak flexion, peak extension angle (*Sit to stand* phase), and average angular velocity (*Turning* and *Stand to sit* phases). The results of the Moment × Program analysis showed that the muscular resistance program carried out by the participants in group A led to more remarkable improvements than the aerobic one performed by the participants in group B, with regard to the variables of peak angular velocity and peak flexion angle (*Sit to stand* phase). These differences in the benefits derived from both programs turned out to be statistically significant in average angular velocity and peak angular velocity (*Turning* and *Stand to sit* phases). The statistical analyses showed that none of the differences found between the initial values and the final ones could be considered a clinically meaningful change.

Discussion

This study explored the effect of three seated exercise programs on strength, balance, functional independence, and functional mobility in a sample composed of people older than 80 years. The obtained results showed that, when choosing an inexpensive physical exercise protocol with low functional requirements for people older than 80 years with cognitive impairment, the performance of muscular resistance seated exercises by using Thera-Band is a more efficient option than programs based on pedal exercisers or routines of joint mobility. However, it must be noted that, according to the statistical analysis done, no clinically meaningful changes were found once the three training programs were finished.

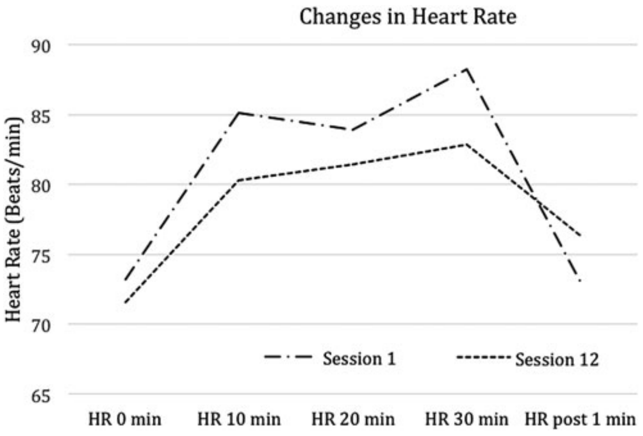

FIG. 1. Mean heart rate registered during the first and last sessions in group A.

Downloaded by Seoul National Univ. Medical College from online.liebertpub.com at 11/18/17. For personal use only.

TABLE 3. EFFECTS OF THE THREE EXERCISE PROGRAMS CARRIED OUT

	Baseline			Post-test			ANOVA (2×3) moment×program	
	Group A (n=13)	Group B (n=12)	Group C (n=11)	Group A (n=13)	Group B (n=12)	Group C (n=11)		
	Mean±SD	*Mean±SD*	*Mean±SD*	*Mean±SD*	*Mean±SD*	*Mean±SD*	*F*	*Sig*
Tinetti test	22.33±5.79	22.33±4.94	18.56±6.46	21.58±7.68	23.33±5.79[a]	18.67±7.00	$F_{2,34}=3.011$	0.024
Tinetti test gait	9.25±2.67	8.92±2.39	7.56±3.45	9.17±3.51	9.17±1.75	7.33±3.50	$F_{2,34}=0.977$	0.084
Tinetti test balance	13.08±3.23	13.42±2.64	11.00±3.04	12.42±4.29	14.17±2.41[a]	11.33±3.64	$F_{2,34}=2.127$	0.038
Barthel index	53.33±19.81	52.42±17.77	56.67±16.01	61.58±19.48	64.17±2.16	57.22±16.22	$F_{2,34}=0.340$	0.033
Hand-grip strength right (kg)	13.00±6.18	11.83±6.22	13.38±5.80	13.25±3.89	12.58±4.12[a]	13.56±6.52	$F_{2,34}=2.336$	0.049
Hand-grip strength left (kg)	13.75±4.77	11.33±6.14	13.50±6.14	13.83±4.15	13.00±5.36[a]	13.67±7.58	$F_{2,34}=2.604$	0.033
Timed Up and Go Test								
Sit to stand (s)	3.19±2.17	3.88±1.95	5.03±3.99	3.01±1.84	3.45±3.77[a]	5.43±2.92[a]	$F_{2,34}=3.627$	0.003
Gait go (s)	9.52±6.84	8.63±2.78	7.66±3.47	8.81±5.33[a]	6.66±2.87[a]	7.86±2.77	$F_{2,34}=2.021$	0.039
Turning (s)	5.55±4.78	4.13±1.19	4.97±2.08	5.20±4.49	3.96±2.54	5.31±1.42[a]	$F_{2,34}=2.220$	0.034
Gait return (s)	7.16±7.35	7.13±2.69	7.51±3.52	4.69±2.51[a]	5.05±2.89[a]	7.43±2.93	$F_{2,34}=2.595$	0.010
Stand to sit (s)	4.90±2.96	5.33±2.09	5.12±1.56	4.87±11.83	5.06±2.06	7.18±8.13[a]	$F_{2,34}=1.863$	0.049
TUG total (s)	30.32±21.37	29.10±5.40	30.28±11.92	26.58±20.28	24.18±8.36	33.21±13.58	$F_{2,34}=4.196$	0.001

[a]Significant differences: baseline–post-test ($p<0.05$).
TUG, Timed Up and Go Test.

These findings can serve as a guide for those health professionals in need of information to decide which protocols should be prescribed and what effects such programs might have on very old populations with low economic resources and limited functional ability. Furthermore, since all three programs proved to be easy to perform, adherence was high, and no injury was sustained, it could be advisable to organize a multi-modal exercise program containing the three protocols (aerobic, strength, and range of motion). This modality of training has been shown to have beneficial effects in the elderly.[24] Thus, a CBE multi-component program could be an interesting exercise training strategy for very old people.

Aerobic exercise intervention

In this research, we presented an aerobic training program based on the use of pedal exercisers. There are not many studies on the effects of these types of programs on very old people; that is why the results of this study can be added to the scientific evidence regarding its usefulness to this population. The obtained findings imply that, for people older than 80 years, pedaling for half an hour continuously on a pedal exerciser did not translate into significant improvements in balance, functional independence, and functional mobility, which would limit the presumed potential benefits of this protocol.

In line with the present findings, Bouaziz et al.[25] carried out a revision on the effects of pedaling in adults older than 70 years and they found that this type of exercise was not considered efficient to improve balance in this population. Opposed to this idea, in a very recent study, Torres et al.[26] found significant changes in static balance in a group of old people (average age 75.6±6.2 years) after them taking part in a pedaling program by using a pedal exerciser. This new result may be due both to the characteristics of the sample (much younger and free from cognitive impairment) and to the protocol, since the intensity of the training increased daily.

The lack of significant effects of the aerobic intervention on the functional mobility is somehow an unexpected finding, since according to the revision carried out by Bouzaiz et al.[25] pedaling involved significant improvements in TUG total time. In this respect, it should be mentioned that the data provided by Wiva showed that the aerobic protocol involved improvements in moving speed, hence the significant reduction observed in the *Gait to go* and *Gait return* phases of TUG, which led to an improvement of almost 4 seconds in TUG total time after the intervention. However, this change was not statistically significant, which might be a consequence of the great variability seen in the results while carrying out the test. In any case, it should not be ruled out that cognitive impairment may have been an influential factor that limited the possibility of significant improvements having been attested. In this regard, Varela et al.,[27] in a sample of old people with cognitive impairment who participated in a recumbent bike pedaling program for 3 months, noticed a trend toward improvement in TUG total time, but that could not reach statistically significant levels. However, Cancela et al.[28] found significant improvements in TUG total score after implementing a similar training program for people older than 80 years with cognitive impairment, although the study period was much longer (15 months). Therefore, perhaps the duration of training might

Downloaded by Seoul National Univ. Medical College from online.liebertpub.com at 11/18/17. For personal use only.

Table 4. Analysis of the Dynamic Balance Parameters (Timed Up and Go Test)

	Baseline			*Post-test*			*ANOVA (2×3) moment×program*	
	Group A (n=*13)*	*Group B* (n=*12)*	*Group C* (n=*11)*	*Group A* (n=*13)*	*Group B* (n=*12)*	*Group C* (n=*11)*		
	Mean±SD	*Mean±SD*	*Mean±SD*	*Mean±SD*	*Mean±SD*	*Mean±SD*	*F*	*Sig*
TUG: sit to stand								
Average angular velocity (°/s)	10.12±3.58	12.01±4.95	11.63±5.49	10.43±3.65	11.21±4.81	9.54±3.10[a]	$F_{2.34}$=2.346	0.046
Peak angular velocity (°/s)	44.35±16.92	46.33±22.98	49.83±161.46	40.05±15.01	40.76±17.53[a]	50.00±12.20	$F_{2.34}$=1.458	0.122
Peak flexion angle (°)	−11.14±6.71	−15.82±6.76	−11.08±21.29	−9.62±7.35[a]	−10.57±7.47[a]	−14.09±6.88[a]	$F_{2.34}$=3.163	0.018
Peak extension angle (°)	4.78±6.73	4.47±8.74	7.58±9.99	7.18±10.40[a]	2.06±4.40[a]	2.50±3.40[a]	$F_{2.34}$=3.371	0.021
AP range (m/s^2)	7.23±1.25	6.50±1.95	6.58±2.22	6.17±1.69	7.18±2.79	6.71±1.59	$F_{2.34}$=0.986	0.419
PD range (m/s^2)	3.85±1.06	3.43±1.21	2.59±0.83	4.17±0.98	4.79±1.27[a]	2.83±1.48	$F_{2.34}$=1.728	0.115
ML range (m/s^2)	2.52±0.89	2.63±1.37	2.80±0.89	2.50±0.80	2.24±0.93	2.56±0.98	$F_{2.34}$=0.121	0.975
TUG: turning								
Average angular velocity (°/s)	75.65±58.70	60.76±15.48	56.49±34.26	79.89±19.98	79.04±22.04[a]	38.30±10.79[a]	$F_{2.34}$=4.403	0.006
Peak angular velocity (°/s)	103.07±59.36	81.24±32.75	90.79±70.85	101.45±25.43	113.46±23.48[a]	85.23±27.51	$F_{2.34}$=4.433	0.003
TUG: stand to sit								
Average angular velocity (°/s)	76.59±30.23	64.12±13.55	55.04±10.55	80.36±43.24	72.91±14.68[a]	39.81±34.75[a]	$F_{2.34}$=4.375	0.009
Peak angular velocity (°/s)	119.52±65.39	82.99±23.75	78.58±13.52	117.83±48.73	107.93±22.30[a]	80.66±165.47	$F_{2.34}$=2.331	0.036
Peak flexion angle (°)	35.76±32.09	43.76±36.77	43.69±39.81	47.59±39.12[a]	54.33±26.70[a]	38.00±33.49	$F_{2.34}$=2.396	0.039
Peak extension angle (°)	−55.98±39.25	−48.99±29.80	−54.49±33.85	−30.47±36.57	−42.92±30.14	−43.08±36.60	$F_{2.34}$=1.107	0.358
Turning peak angular velocity (°/s)	108.07±53.61	86.24±25.83	83.26±76.78	107.83±48.73	87.93±22.30	80.66±165.47	$F_{2.34}$=0.536	0.710

[a]Significant differences: baseline–post-test ($p<0.05$).
AP, anterior-posterior; ML, medial-lateral; PD, proximal-distal.

Downloaded by Seoul National Univ. Medical College from online.liebertpub.com at 11/18/17. For personal use only.

be considered as another constraining factor in this respect. Finally, it could be hypothesized that the lack of significant effects of the aerobic intervention on TUG total time might be due to the level of dependence and the old age of the sample, since both factors have a direct influence on test performance.[29] In fact, the level of dependence to carry out daily activities did not experience significant changes throughout the intervention, a finding that can be explained on the basis of the intensity with which the program was performed. Thus, it could be expected that, after an aerobic exercise program, the cardiorespiratory capacity would improve and adults would become less tired when dealing with their daily activities. Nevertheless, in the light of the data registered by the HR monitor, pedaling intensity was less than 60% maximum HR,[30] showing that perhaps the training program was not intense enough to cause changes in the level of tiredness. Similarly, Cancela et al.[28] did not find a significant effect on functional independence after a 15-month pedaling program, which revealed the existence of a certain difficulty for this type of populations to improve this parameter through aerobic pedaling interventions.

Muscular resistance exercise intervention

Exercises with elastic bands have shown that they are useful in the elderly, even though there are not many studies regarding the effects of this type of programs when they are performed sitting down. The training protocol suggested in this study had significant effects on muscle strength and balance, a finding previously reported in other pieces of research. For instance, Lee et al.[31] found significant improvements in the levels of muscle force of a group of elderly women (average age 74.2 ± 4.6) after them participating in an 8-week elastic band exercise program. Similarly, a recent study by Kwak et al.[32] found significant improvements in static balance in a group of 15 healthy independent senior citizens (average age 80.1 ± 4.7 years) who were free from cognitive impairment and who took part in an 8-week elastic band exercise program.

In a way similar to what could be noted after the aerobic program, the training with elastic bands did not mean an important improvement in the functional mobility. The data provided by Wiva showed that muscular resistance training led to significant reductions in partial time in three of the five phases in which TUG was divided. It also meant significant improvements in angular velocity during the exercise, which involved a reduction in TUG total time of almost 5 seconds. Despite this, the changes found in this variable were not statistically significant, which might be due to the protocol that they were following. Kwak et al.[32] found statistically significant improvements in TUG total time, although the training protocol combined lying down, sitting, and standing exercises. Furthermore, during the final weeks, participants replaced the yellow elastic band with a green one, which presented more resistance; therefore, it may be assumed that the effect of the program was more intense.

In the present study, it was not possible to change the level of resistance of the elastic band because the participants' strength levels did not advise it. Gill et al.,[33] on their part, observed that in a home-based exercise intervention carried out on a group of frail people older than 80 years, most participants did not manage to go beyond the initial Thera-Band, which confirmed the difficulty of increasing exercise intensity when applying this kind of programs to older populations. Similar to what happened to the participants of group A in this study, the lack of significant improvements in TUG might perhaps be related to the fact that the sample consisted of people with cognitive impairment. Other researchers who developed interventions based on Thera-Band exercise performance by elderly people (79–80 years old) with cognitive impairment found that the program, despite causing significant changes in variables such as gait speed, did not produce significant improvements in TUG total time.[34,35] The age of the sample and its level of dependence may have limited the scope for improvement in this test, as set out earlier. Indeed, as previously observed in the participants in group A, the muscular resistance protocol did not result in significant changes in the functional independence of the participants included in group B. Given that participants in groups A and B achieved higher BI scores at the end of the intervention, unlike what happened to the participants in group C, the effect found would seem to be one of maintenance function in a population that tends to become increasingly frail and dependent.[36]

The fact that the muscular resistance did not have a significant impact on the functional independence agrees with other authors' comments. Así, Liu et al.,[5] and Liu and Latham[37] reported that resistance strength training had a positive influence, although not significant, on the capacity to deal with daily activities in an effective way. It should be noted that Chen et al.[38] found that a strength exercise program with Thera-Band resulted in significant improvements in the functional independence of a group of 80-year-olds, who performed the exercises while sitting on their wheel chairs. The program lasted for 6 months and it was not until the end that significant changes were found in the way that the participants faced their daily activities, showing a higher level of independence. Therefore, it could be argued that the efficiency of the protocol suggested in this study could have been limited by program duration.

Study limitations

Despite the original approach taken in this study, which compares three different protocols of low-cost training programs of seated exercises, the characteristics of the sample (consisting only of adults in a day care center), its reduced size, and the fact that researchers were not blind may limit the solidity and potential generalization of the results presented here. Moreover, it should be taken into consideration that the sample in this research had cognitive impairment, constraints in their level of functional independence, and very high TUG scores. Finally, it should be noted that the proposed protocols were carried out in groups and under supervision. Therefore, further research is needed to test their feasibility when performed at home.

Conclusion

In a sample of people older than 80 years, there were no significant changes in functional mobility and functional independence after three different types of physical exercise programs were performed in sitting position, using pedal exercisers, performing muscular resistance exercises with elastic bands, and participating in joint mobility routines for

3 months. The positive effects on strength and balance caused by the Thera-Band training protocol make this option the most advisable of the three proposals.

Acknowledgments

The authors thank all the participants and their families for their willingness to take part in this study. They also thank the geriatric medical staff for their support with data collection and scientific activities.

Author Disclosure Statement

No competing financial interests exist.

References

1. Mendonca GV, Pezarat P, Vaz JR, Silva L, Almeida ID, Heffernan KS. Impact of exercise training on physiological measures of physical fitness in the elderly. Curr Aging Sci 2016;9:240–259.
2. Schwartz JB. Primary prevention: Do the very elderly require a different approach? Trends Cardiovasc Med 2015;25:228–239.
3. Venturelli M, Schena F, Richardson RS. The role of exercise capacity in the health and longevity of centenarians. Maturitas 2012;73:115–120.
4. Álvarez F, del Pozo J, del Pozo B, Alfonso RM, Rogers ME, Zhang Y. Effects of supervised whole body vibration exercise on fall risk factors, functional dependence and health-related quality of life in nursing home residents aged 80+. Maturitas 2014;79:456–463.
5. Liu D, Tan J, Guo Y, et al. The contributing risk factors, prevention and treatment of functional dependence among the oldest-old and elderly subjects. Chin J Inter Med 2014;53:772–777.
6. Ansai JH, Aurichio TR, Gonçalves R, Rebelatto JR. Effects of two physical exercise protocols on physical performance related to falls in the oldest old: A randomized controlled trial. Geriatr Gerontol Int 2016;16:492–499.
7. Serra JA, Bustamante N, Hierro M, et al. Short-term, light-to-moderate-intensity exercise training improves leg muscle strength in the oldest old: A randomized controlled trial. J Am Geriatr Soc 2011;59:594–602.
8. van Roie E, Bautmans I, Coudyzer W, Boen F, Delecluse C. Low-and high-resistance exercise: Long-term adherence and motivation among older adults. Gerontology 2015;61:551–560.
9. Picorelli AMA, Pereira LSM, Pereira DS, Felício D, Sherrington C. Adherence to exercise programs for older people is influenced by program characteristics and personal factors: A systematic review. J Physiother 2014;60:151–156.
10. van Heuvelen MJ, Hochstenbach JB, Brouwer WH, de Greef MH, Scherder E. Psychological and physical activity training for older persons: Who does not attend? Gerontology 2006;52:366–375.
11. Miranda A, Picorelli A, Pereira DS, Felício DC, Dos Anjos DM, Pereira DAG. Adherence of older women with strength training and aerobic exercise. Clin Interv Aging 2014;9:323–331.
12. Zak M, Swine C, Grodzicki T. Combined effects of functionally-oriented exercise regimens and nutritional supplementation on both the institutionalised and free-living frail elderly (double-blind, randomised clinical trial). BMC Public Health 2009;9:39–44.
13. Anthony K, Robinson K, Logan P, Gordon AL, Harwood RH, Masud T. Chair-based exercises for frail older people: A systematic review. Biomed Res Int 2013;2013:309506.
14. Robinson KR, Leighton P, Logan P, Gordon AL, Anthony K, Harwood RH, Gladman J, Masud T. Developing the principles of chair based exercise for older people: A modified Delphi study. BMC Geriatr 2014;14:65–74.
15. Robinson KR, Masud T, Hawley-Hague H. Instructors' perceptions of mostly seated exercise classes: Exploring the concept of chair based exercise. Biomed Res Int 2016; 2016:3241873.
16. Durutürk N, Manolya A, Karataş M. Effects of chair-based exercises for older people on physical fitness, physical activity, sleep problems and quality of life: A randomized controlled trial. Turkiye Klinikleri J Health Sci 2016;1: 47–54.
17. López TI, Torres I, Martín SA, Ortiz RA, Rodríguez AE, Valenza MC. Cognitive impairment, nutritional status and clinical profile in chronic obstructive pulmonary disease. Nutr Hosp 2013;30:1152–1159.
18. Lobo A, Saz P, Marcos G, et al. Revalidación y normalización del mini-examen cognoscitivo (primera versión en castellano del mini-mental status examination) en la población general geriátrica. Med Clin 1999;112:767–774.
19. Rodríguez C, Lugo LH. Validity and reliability of Tinetti scale for Colombian people. Rev Colomb Reumatol 2012; 19:218–233.
20. Cabañero MJ, Cabrero J, Richart M, Muñoz CL. The Spanish versions of the Barthel index (BI) and the Katz index (KI) of activities of daily living (ADL): A structured review. Arch Gerontol Geriatr 2009;49:e77–e84.
21. Podsiadlo D, Richardson S. The timed ''Up & Go'': A test of basic functional mobility for frail elderly persons. J Am Geriatr Soc 1991;39:142–148.
22. Chen HM, Chen CC, Hsueh IP, Huang SL, Hsieh CL. Test-retest reproducibility and smallest real difference of 5 hand function tests in patients with stroke. Neurorehabil Neural Repair 2009;23:435–440.
23. Dvir Z. Difference, significant difference and clinically meaningful difference: The meaning of change in rehabilitation. J Exerc Rehabil 2015;11:67–73.
24. de Labra C, Guimaraes-Pinheiro C, Maseda A, Lorenzo T, Millán-Calenti JC. Effects of physical exercise interventions in frail older adults: A systematic review of randomized controlled trials. BMC Geriatr 2015;15:154–169.
25. Bouaziz W, Schmitt E, Kaltenbach G, Geny B, Vogel T. Health benefits of cycle ergometer training for older adults over 70: A review. Eur Rev Aging Phys Act 2015;12:8–21.
26. Torres I, Valenza MC, Cabrera I, López I, Benítez Á, Conde A. Effects of an exercise intervention in frail older patients with chronic obstructive pulmonary disease hospitalized due to an exacerbation: A randomized controlled trial. COPD 2016;14:37–42.
27. Varela S, Ayán C, Cancela JM, Martín V. Effects of two different intensities of aerobic exercise on elderly people with mild cognitive impairment: A randomized pilot study. Clin Rehabil 2012;26:442–450.
28. Cancela JM, Ayán C, Varela S, Seijo M. Effects of a long-term aerobic exercise intervention on institutionalized patients with dementia. J Sci Med Sport 2016;19:293–298.
29. Ayán C, Cancela, JM, Gutiérrez A, Prieto I. Influence of the cognitive impairment level on the performance of the Timed ''Up & Go'' Test (TUG) in elderly institutionalized people. Arch Gerontol Geriatr 2013;56:44–49.

Downloaded by Seoul National Univ. Medical College from online.liebertpub.com at 11/18/17. For personal use only.

30. Tanaka H, Monahan KD, Seals DR. Age-predicted maximal heart revisited. J Am Coll Cardiol 2001;37:153–156.
31. Lee HC, Lee ML, Kim SR. Effect of exercise performance by elderly women on balance ability and muscle function. J Phys Ther Sci 2015;27:989–992.
32. Kwak CJ, Kim YL, Lee SM. Effects of elastic-band resistance exercise on balance, mobility and gait function, flexibility and fall efficacy in elderly people. J Phys Ther Sci 2016;28:3189–3196.
33. Gill TM, Baker DI, Gottschalk M, et al. A prehabilitation program for physically frail community-living older persons. Arch Phys Med Rehabil 2003;84:394–404.
34. Hageman PA, Thomas VS. Gait performance in dementia: The effects of a 6-week resistance training program in an adult day-care setting. Int J Geriatr Psychiatry 2002;17:329–334.
35. Thomas VS, Hageman PA. Can neuromuscular strength and function in people with dementia be rehabilitated using resistance-exercise training? Results from a preliminary intervention study. J Gerontol A Biol Sci Med Sci 2003;58:M746–M751.
36. Crocker T, Young J, Forster A, Brown L, Ozer S, Greenwood DC. The effect of physical rehabilitation on activities of daily living in older residents of long-term care facilities: Systematic review with meta-analysis. Age Ageing 2013;42:682–688.
37. Liu CJ, Latham NK. Progressive resistance strength training for improving physical function in older adults. Cochrane Database Syst Rev 2009;8:CD002759.
38. Chen MC, Chen KM, Chang CL, Chang YH, Cheng YY, Huang HT. Elastic band exercises improved activities of daily living and functional fitness of wheelchair-bound older adults with cognitive impairment: A cluster randomized controlled trial. Am J Phys Med Rehabil 2016;95:789–799.

Address correspondence to:
José M. Cancela Carral
Departament Specials Didactics
Faculty of Education and Sport Sciences, Sport Sciences
University of Vigo
Campus A Xunqueira s/n
36005 Pontevedra
Spain

E-mail: chemacc@uvigo.es

Received: January 16, 2017
Accepted: May 8, 2017

Downloaded by Seoul National Univ. Medical College from online.liebertpub.com at 11/18/17. For personal use only.
