# Supplementary material for: Effects of elastic band resistance training on the physical and mental health of elderly individuals: A mixed methods systematic review
Source: PLoS One. 2024 May 13;19(5):e0303372. doi: 10.1371/journal.pone.0303372 (PMC11090353; doi:10.1371/journal.pone.0303372)
Supplement: S1 File — (ZIP) [file pone.0303372.s001.zip › Supporting Information/Included study 51.pdf]

Copyright 1999 by
The Gerontological Society of America

The Gerontologist
Vol. 39, No. 6, 705–710

The short-term effects of an accessible exercise intervention on the strength and health-related quality of life (HRQOL) among older adult women were evaluated. We conducted an 8-week resistance training intervention utilizing elastic bands in 62 community-dwelling women with a mean age of 68 years. Participants were randomly assigned to either an exercise or a control group. Pre- and postintervention assessments included strength tests and HRQOL. Results revealed significant increases in three major muscles compared to the control group. However, there were no significant changes on either mental or physical health functioning. The elastic bands provide older adult women with an inexpensive, practical exercise program that effectively increases strength within 8 weeks but may have little effect on self-reported HRQOL.
Key Words: Exercise, Mental functioning, Physical functioning

Downloaded from https://academic.oup.com/gerontologist/article/39/6/705/593459 by University of Electronic Science & Tech user on 23 July 2022

The Effects of Strength Training on Strength and Health-Related Quality of Life in Older Adult Women

Teresa M. Damush, PhD,[1] and Joseph G. Damush, Jr., MA[2]

Resistance training interventions have successfully produced strength improvements in older adults (Fiatarone et al., 1994; Mihalko & McAuley, 1996; Morganti et al., 1995; Pyka, Lindenberger, Charette, & Marcus, 1994). Indeed, strength training appears to offer aging adults many physiological benefits (e.g., increased muscle mass [Pratley et al., 1994] and bone density [Heath, 1988; Layne & Nelson, 1999]). In addition, increased strength has been shown to help individuals maintain their functioning and mobility as they age (Simonsick et al., 1993), thereby suggesting that strength training may positively affect multiple domains of health-related quality of life (HRQOL; e.g., pain, physical and social functioning, and psychological distress). However, older adults, especially older adult women, may not have access to traditional resistance training equipment (e.g., weight machines) to reap potential health benefits.

The majority of resistance training interventions have involved the use of weight machines at a facility or clinical laboratory (McCartney, Hicks, Martin, & Webber, 1996; Morganti et al., 1995). Although these interventions produced strength improvements, these gains will diminish if the intervention is not maintained. Alternative, more accessible, resistance programs may be more feasible for older women to implement and maintain long term. Few intervention trials have used alternative, more accessible forms of strength training such as hand and ankle weights (Ettinger et al., 1997; Mihalko & McAuley, 1996) and elastic bands (Jette et al., 1996). These interventions were advantageous in that external validity was high and participants were able to transfer the intervention training to their home environment.

In addition to a lack of alternative resistance programs, there is a paucity of research on the effects of resistance training on HRQOL in older adults. Recent randomized controlled trials of aerobic exercise interventions in aging adults have demonstrated improvements in physical health (Stewart, King, & Haskell, 1993), social activity and perceived energy (Emery & Blumenthal, 1990), and anxiety (King, Taylor, & Haskell, 1993). Only two recent studies examined the effects of resistance training on HRQOL among older adults. First, Mihalko and McAuley (1996) reported a positive trend on subjective well-being and activities of daily living in a small elderly nursing home sample after strength training. However, the differences were nonsignificant. A second study reported that strength training enhanced self-reported psychological well-being and health status in nondisabled, community-dwelling older adult men; however, this effect was not found in older adult women (Jette et al., 1996). A greater understanding of the effects of a program of strength training on multiple domains of HRQOL among older adults is thus indicated.

Fitness Wholesale and the Hygenics Corporation, Akron, Ohio provided in-kind support for this study. Partial support was provided by the University of California, Riverside Graduate Dean's Dissertation Research Grant. Part of this article was presented at the 1997 annual meeting of the Society of Behavioral Medicine, San Francisco, California. The authors would like to thank the communities of The Colony at California Oaks, Murrieta, CA, and The Club in Sun City, CA, for the use of their facilities and the women who participated. We would also like to thank Dr. Robin DiMatteo for guidance on the project, Tony Perkins for statistical support, and Drs. Dan Clark, Faryle Nothwehr, Anita Stewart, and Morris Weinberger for comments on the article.
[1]Address correspondence to Dr. Teresa M. Damush, Regenstrief Institute for Health Care, Indiana University Center for Aging Research, and School of Medicine, Indiana University, 1001 W. 10th St., RG6, Indianapolis, IN 46202-2859. Email: damush_t@regenstrief.iupui.edu
[2]Five Seasons Sports Club, Indianapolis, IN.

The purpose of this study was to evaluate prospectively the impact of an accessible, strength training program on overall improvements in health, functioning, and well-being as well as strength among older adult women. The design included an 8-week randomized trial of a resistance training intervention using elastic bands. More specifically, the average positive change in strength and HRQOL among the exercise group at postintervention was expected to be greater than the average positive change among the control group. Exercise participation was expected to result in increased strength and improved mental and physical functioning.

Methods

Overview of Study Design

This randomized trial evaluated the effects of strength training on strength and HRQOL. During the pre- and postintervention assessments, we assessed all participants on all measures listed following provision of informed consent. We distributed a self-administered battery of HRQOL measures. We conducted strength assessments during individual appointments in the strength facilities of both senior communities. Missing data resulted from participant scheduling difficulties and illness. The exercise participants completed an 8-week strength training class intervention, and the waiting list group attended the intervention but did not exercise.

Participants

We recruited women, aged 55 years and older from two retirement residential (single-home dwellings) communities in Southern California through a media-based promotion. We recruited women interested in participation from "no obligation" seminars where we pre-sented a general description of the study and an exercise demonstration. The demographic characteristics of the final sample at postintervention (*N* = 62) are presented in Table 1. The majority of the women were Caucasian, married, high school graduates, and nonsmokers.

Assignment to Groups.—Seventy-one women interested in participating in the intervention returned a completed written medical clearance form from their primary care physician, which indicated no contraindications for exercising. They were randomly assigned to either the exercise or waiting list control group.

Experimental Group.—The experimental group of 40 women proceeded through an 8-week, twice-weekly strength training intervention program designed to tone and strengthen three major muscle groups utilizing elastic exercise bands (VanGalen, 1995). Of the 40 participants in the exercise group, 7 (17%) women did not complete the study. The reasons for dropout were illness in six cases and moving for one participant. The final size of the exercise group was 33 older adult women.

Table 1. Sample Characteristics (*N* = 62)

Characteristic	Statistic
Age in years	
Mean (*SD*)	68 (5.58)
Range	59–84
Education	
Completed high school	93%
Attended some college	57%
Have a college degree	5%
Ethnicity	
Caucasian	95%
Hispanic/Latino	5%
Household income	
<$20,000	13%
$20,000–$39,999	66%
≥$40,000	21%
Married	75%
Current cigarette smokers	8%
Number of chronic conditions	
Range	0–6
Mean (*SD*)	1.89 (1.6)

Notes: The response choice for education included: grade school, some high school, high school graduate, some college, college graduate, and graduate or professional degree. The response set for annual household income included eleven categories of income by increments of $10,000.

Waiting List Control Group.—Thirty-one women were assigned to the waiting list control group. Of the 31 women, 2 (6%) dropped out of the study. One reported moving and the other did not state a reason. Thus, the final sample of the control group was 29 older adult women. This group of women attended all of the exercise classes with the intervention group but did not exercise. The purpose of this design, in addition to comparing physical status, was to control for the social interaction and scheduled commitment from attending the class. Each of the control group members was randomly paired with an exercising partner. The waiting list group member kept a record of the intensity and amount of repetitions that the exerciser completed for each exercise. This information provided feedback to the exerciser and was not used as data. The waiting list control group did not receive any type of exercise intervention for the eight weeks and was asked to refrain from using elastic bands. The control group did have the opportunity to exercise in a stretch band class or individually after the initial intervention was completed.

Measures

The questionnaire described below was used for both baseline and postintervention assessments. It included demographic information including age, annual household income, education, and marital and retirement status, and a battery of HRQOL measures. The health and functioning scales used in the questionnaire were measures identified in the psychological and health literature as HRQOL concepts (Stewart & King, 1991; Stewart et al., 1993; Stewart & Ware, 1992).

Downloaded from https://academic.oup.com/gerontologist/article/39/6/705/593459 by University of Electronic Science & Tech user on 23 July 2022

Health-Related Quality of Life (HRQOL).—We created two summary HRQOL scores, Mental and Physical Health Functioning, for a more conservative analyses. The two summary scores comprised ten HRQOL measures from the RAND Medical Outcomes Study (MOS; Stewart & Ware, 1992) and included: anxiety (e.g., feelings of nervousness), depression (e.g., feelings of low spirits), energy-fatigue (e.g., feelings of pep), sleep problems (e.g., sleep disturbance), general health perceptions (e.g., overall health), pain (e.g, interference with life), physical functioning (e.g., activity functioning), positive affect (e.g., feelings of happiness), role functioning (e.g., role limitations), and social activity limitations (e.g., limits due to health). The ten measures were reliable at baseline assessment. The alpha internal-consistency coefficients of baseline measures ranged from .73 to .92. All scores were transformed to a 0–100 score range. Higher scores indicated better health. These scores represent the percentage of total possible score achieved. The two HRQOL indices were created from the average of the ten scales' transformed scores.

The Mental Health Functioning index encompassed anxiety, depression, energy-fatigue, positive affect, and sleep problems. The Physical Health Functioning index consisted of current health perceptions, physical functioning, pain, role functioning, and social activity limits. Similar indices using the RAND MOS measures have been previously created in a sample of exercising older adults (Stewart et al., 1993).

Chronic Conditions.—Participants self-reported the total number of chronic conditions or illnesses as diagnosed by a physician using a checklist of 19 common medical conditions (i.e., arthritis, cancer, diabetes, hypertension). The mean, standard deviation, and range are listed in Table 1.

Strength Measures

We conducted strength assessments of the exercise and waiting list control groups during the pre- and post-test assessments.

Strength.—A three-repetition maximum (3-RM) method was used to assess maximum strength by measuring the maximum amount of resistance that was moved in three consecutive repetitions with correct form through the full range of motion for each exercise (Pratley et al., 1994). We used a 3-RM to prevent injury in this older adult sample. To avoid injury and to familiarize the participants with the apparatus, we began each strength test with a warm-up set with low weights on each of the machines. At the beginning of testing, initial weights were set at levels close to anticipated maximal weight based upon participants' current activity level in order to decrease fatigue. Weights were then adjusted in increments that ranged from 0.5–10 lbs.

In order to determine the resistance progression during the strength test for this inexperienced, elderly sample, we assessed subjective exertion. The exercise physiologist assessed the subjective, perceived effort using both the participant's perception of her ability to complete three additional repetitions using correct form and the Borg perceived exertion scale (Noble, 1982). The participant had reached her 3-RM when she perceived that she could not complete another three repetitions with full range of motion and perceived the effort to be at least "Somewhat Hard or Strong Exertion," level 4 on the Borg scale. We tested three major muscle groups: latissimus dorsi (assessed on latissimus pull down exercise), pectorals (assessed on seated chest press), and quadriceps (assessed on seated leg extension).

We assessed strength in both the left and right hands with a Jamar hand dynamometer. This apparatus measures maximal grip effort in pounds per force (Kallman, Plato, & Tobin, 1990). After adjusting the dynamometer for hand comfort, seated participants were instructed to grip the device with their maximal effort. Grip strength in each hand was assessed three times and the highest value for each hand was retained for analyses.

Procedures

Eight-Week Training Protocol.—The training sessions were held twice per week, and each session lasted approximately 45 min. Each class began with a 5-min walk as a warm-up, 30 min of elastic band exercises, and a 5-min cooldown of upper and lower body stretching. The exercisers sat in folding chairs and were led by an American College of Sports Medicine (ACSM) and American Senior Fitness Association certified and degreed instructor. The control group member sat facing the exerciser while monitoring the exercise.

The first class of the intervention was spent performing the exercises and establishing the initial intensity level of elastic resistance using the manufacturer's guidelines (VanGalen, 1995). Each exerciser started with the lightest band and changed bands when the routine no longer felt difficult by the end of the exercise. To aid participants with their subjective evaluations of perceived exertion, we instructed the exercisers to evaluate the exertion using the Borg scale (Noble, 1982) as a reference after completing one exercise set. If exertion was less than level 4, "Somewhat Hard or Strong Exertion," we encouraged the participant to increase the resistance. The elastic band progression suggested by the manufacturer was provided to the participants.

The intervention consisted of one set each of seven different resistance exercises using elastic stretch bands and involved major muscle groups of the upper and lower extremities. These exercises included: seated rear lat pulldown, seated single leg press, seated chest press, seated single toe press, standing tricep press, standing bicep curl, and seated leg extension. The participants were instructed to perform as many repetitions as they could in one set until they reached "Somewhat Hard or Strong Exertion," level 4 on the Borg Perceived Exertion Scale. Participants rested in between the seven exercises for approximately 90 s.

As an incentive to complete the study, waiting list group participants were required to earn their free

Downloaded from https://academic.oup.com/gerontologist/article/39/6/705/593459 by University of Electronic Science & Tech user on 23 July 2022

exercise by attending at least 75% or 12 of the 16 scheduled exercise classes during the 8 weeks, and the exercise group earned their elastic bands by attending 75% of the classes. The average class attendance for each group was 14 classes. The entire final sample completed at least 12 of the 16 scheduled classes.

Methods of Analysis

To assess baseline demographic differences between groups, we used *t* tests to compare age, annual household income, educational level, and total number of chronic conditions. For dichotomous variables, marital and retirement status, we computed chi-square analyses.

To determine baseline strength differences between groups and the effects of the resistance training on strength changes, we computed repeated measures (group by time) analyses of variance for each of the three major muscle groups and both grips. Group status was the between-group factor, and time or repeated measures was the within-subject factor.

In addition, analysis of variance was computed to compare baseline HRQOL between groups. Given that HRQOL can be affected by age, chronic conditions, and smoking status independent of strength (Parker, Thorslund, Lundberg, & Kareholt, 1996), we used analysis of covariance to control for these variables. In addition, baseline mental and physical functioning indices were added as covariates to evaluate the effect of strength training participation on HRQOL changes between baseline and postintervention.

An alpha level of $p < .05$ was used for all statistical tests.

Results

Participant Characteristics

At baseline, self-reported physical activity participation indicated that over 50% of participants engaged in exercise or a physical activity during their leisure time at least 4 to 5 times per week. Baseline self-reported physical activity frequency did not significantly differ between the exercise and control groups, $t(60) = -.12$, $p \leq .90$.

Analysis of demographic information assessed at baseline revealed that participants in the exercise and control groups did not differ significantly from each other in age ($t(59) = .29$, $p \leq .77$), annual household income ($t(53) = -.35$), $p \leq .72$.), educational level ($t(56) = .54$, $p \leq .59$), total number of chronic conditions ($t(60) = -.76$), $p \leq .45$.), marital status ($\chi^2(1, N = 62) = .34$, $p \leq .56$) or retirement status ($\chi^2(1, N = 62) = .94$, $p \leq .63$).

Strength Changes

To determine the effects of the strength training intervention on strength, we made several comparisons between the exercise and control groups. The exercise group's average strength increased from baseline to postintervention for all three major muscle groups. On average, the exercise group showed a 19.7% increase in latissimus dorsi strength, a 27.7% increase in quadricep strength, and a 16.5% increase in pectoral strength after participation in the 8-week strength training intervention. On average, the waiting list control group strength scores indicated a 1% decrease in latissimus dorsi strength, a 3% increase in quadricep strength, and a 1.7% decrease in pectoral strength. Table 2 presents the pre- and postintervention strength scores for the exercise and control groups.

To evaluate baseline strength differences between groups and the effects of the resistance training on strength changes, we computed repeated measures (Group × Time) analyses of variance for the major muscle groups and both grips. Group status was the between-group factor, and time or repeated measures was the within-subject factor. To protect the probability of Type I error in these five analyses, Boneferroni adjustment indicated that effects had to be significant at the .01 level. At baseline, the groups did not differ significantly on any of the strength scores, latissimus dorsi ($F(1,56) = .18$, $p \leq .67$), pectorals ($F(1,57) = .52$, $p < .47$), and quadriceps ($F(1,57) = 1.48$, $p \leq .23$), left grip ($F(1,39) = .01$, $p \leq .92$), or right grip ($F(1,45) = .58$, $p \leq .45$). As predicted, the exercise group significantly increased their strength from baseline to postintervention (Group × Time interaction) in the major muscle groups, latissimus dorsi ($F(1,56) = 28.9$, $p \leq .0001$), pectorals ($F(1,57) = 9.39$, $p \leq .01$), and quadriceps ($F(1,57) = 20.56$, $p \leq .0001$), compared to the waiting list control group. Furthermore, there were no significant changes in left ($F(1,39) = 5.11$, $p \leq .05$) or right hand grip strength ($F(1,45) = .19$, $p \leq .67$) compared to the control group.

Health-Related Quality of Life Changes

Table 3 presents the means and standard deviations of the 10 HRQOL measures at baseline and postintervention for the exercise and control groups, respectively. Analysis of variance revealed no significant differences between groups at baseline for all the HRQOL scales. To evaluate the effect of strength training participation on HRQOL changes between baseline

Table 2. Strength Scores Before and After Intervention for the Exercise and Control Group

	Exercise Group (n = 33)		Social Waiting List Group (n = 29)	
Variable	Baseline	8 weeks	Baseline	8 weeks
Latissimus dorsi	52 (9.7)	60 (10.3)	54 (10.2)	53 (10.4)
Pectorals	42 (8.7)	48 (11.2)	41 (7.7)	40 (11.8)
Quadriceps	46 (13.0)	58 (17.6)	51 (14.6)	52 (17.4)
Left grip	53 (10.2)	59 (10.3)	55 (8.4)	55 (10.5)
Right grip	60 (10.1)	64 (12.2)	57 (13.8)	64 (14.1)

Notes: Latissimus dorsi, pectorals, and quadriceps are major muscle groups and strength is measured in pounds. Grip strength is measured in pounds per force. Strength scores presented represent 3-RM values. Values are mean (*SD*).

Downloaded from https://academic.oup.com/gerontologist/article/39/6/705/593459 by University of Electronic Science & Tech user on 23 July 2022

Table 3. The Mean and Standard Deviation of Health-Related Quality of Life Measures

Construct	Exercise Group (n = 33) Baseline	Exercise Group (n = 33) 8 Weeks	Social Waiting List Group (n = 29) Baseline	Social Waiting List Group (n = 29) 8 Weeks
Mental Health				
Anxiety	78.6 (16.8)	81.4 (14.9)	84.9 (13.3)	83.2 (15.6)
Depression	79.5 (20.2)	81.6 (17.9)	84.3 (18.5)	83.2 (17.2)
Energy-fatigue	60.3 (21.3)	61.8 (19.6)	56.4 (25.2)	62.4 (21.3)
Positive affect	67.6 (18.5)	69.1 (16.3)	68.8 (19.0)	71.2 (18.2)
Sleep problems	73.9 (14.4)	76.1 (13.9)	76.9 (16.2)	79.2 (15.6)
Physical Health				
Current health perceptions	71.1 (21.1)	74.2 (19.6)	66.0 (23.0)	75.3 (19.9)
Pain	74.2 (25.6)	79.0 (20.4)	82.5 (16.8)	85.6 (18.7)
Physical functioning	80.3 (19.5)	81.8 (18.8)	78.8 (24.5)	80.7 (24.2)
Role functioning	72.7 (31.8)	75.9 (33.4)	75.5 (38.2)	78.3 (36.7)
Social functioning	73.2 (15.4)	73.4 (14.9)	73.4 (17.9)	76.3 (12.5)

Notes: All measures are part of the RAND Medical Outcomes Study Core Measures (Stewart & Ware, 1992). A higher score indicates better health. Scores range from 0–100. Missing data were replaced with group means. Each of the HRQOL scales were nonsignificant between groups at baseline. Values are mean (*SD*).

and postintervention compared to the control group, we conducted several analyses of covariance. For conservative analyses, changes in the two HRQOL indices, Mental and Physical Health Functioning, were the dependent variables. Baseline mental and physical functioning indices, as well as age, total chronic conditions, and smoking status were employed as covariates.

Results indicated that after adjusting for covariates, the exercise group's change in mental health functioning ($F(1,61) = .31$, $p \geq .10$, effect size = .10) and physical health functioning ($F(1,61) = .01$, $p \geq .10$, effect size = .02) did not differ significantly from the control group after 8 weeks of strength training.

Discussion

This study examined the effects of an elastic band resistance strength training program on the strength and HRQOL of older adult women. Consistent with other strength intervention studies using weight machines (McCartney et al., 1996; Pyka et al., 1994), participants who completed the 8-week strength training intervention significantly increased their strength in the three major muscle groups (15% latissimus dorsi, 13% pectorals, and 25% quadriceps) compared to the social control participants. Other studies produced larger strength changes (50%–95% increase). However, their duration was much longer (1–2 years; McCartney et al., 1996; Pyka et al., 1994) or used nursing home residents who had limited initial strength (Fiatarone et al., 1994). The only other elastic band study showed a 10% increase in knee-extension strength in older adults after a 12–15 week home-based intervention and no significant increase in shoulder strength (Jette et al., 1996).

These strength results demonstrate that modest strength gains in healthy older adult women can be obtained with inexpensive equipment in a relatively short time period. The use of resistance other than weight machines in an intervention may provide participants with the opportunity to continue and maintain the exercise regimen postintervention.

We had hypothesized that participating in a strength training intervention would produce improvements in mental health functioning. Although we did not find significant differences between groups, the mean differences were in the predicted direction for anxiety and depression. Other short-term strength training interventions reported similar results. Jette and colleagues (1996) reported no significant decreases in nervousness and tension in older adult women after a 12–15 week home-based (low supervision) elastic band intervention. Mihalko and McAuley (1996) also found less reported negative affect and greater positive affect after an 8-week strength training intervention, but the differences were also not significant from the control group members who attended a separate fluid movement class. It is possible that changes in mental health functioning develop over a longer duration of resistance training or that the social factor associated with the class was enough to stimulate improvements in mental health functioning independent of changes in strength. The length of this duration is unclear as well as the mechanisms involved in these changes.

Contrary to our hypothesis, the elastic band strength training intervention did not significantly affect physical health functioning (self-reported) although the mean differences were in the predicted direction. It is likely that a ceiling effect was present in these variables. Our participants were nondisabled, community dwellers. Jette and colleagues (1996) also did not find any significant differences in self-reported physical functioning between the intervention and control groups except for social functioning. Unlike the present study, the control group in Jette and colleagues (1996) was a waiting list group and did not have any group or social contact with other members.

The intervention in our study included a unique aspect in the design, a social waiting list control group. The class and partner structure provided instructions as well as a social supportive environment to exercise. The mere social atmosphere of the intervention may have had an effect on the HRQOL outcomes. We partially controlled this potential confound of so-

Downloaded from https://academic.oup.com/gerontologist/article/39/6/705/593459 by University of Electronic Science & Tech user on 23 July 2022

cial support by exposing the control group to the same instructor and class conditions except for resistance training. However, the social group improved on all of the physical health domains and some of the mental health domains. The control group improvements on HRQOL despite a lack of strength training and strength changes suggests that attending a scheduled, peer group activity outside of the home and assisting a partner may positively affect functioning among older adult women by possibly providing a sense of belonging, something meaningful to do, or a social network. There are no other studies to our knowledge that have exposed the control group simultaneously to the social aspect of a group exercise intervention. Some studies provide a separate and different class to control group members (Mihalko & McAuley, 1996). However, the instructor or class conditions may differ. In addition, having to walk from home or the parking lot to the class twice a week may have had an effect on physical functioning among the control group.

Several limitations of this study need to be acknowledged. First, the number of participants was small even with extensive recruitment procedures. The consequence of obtaining a small sample size was low power. Second, the overall attrition rate was 13% (9 participants). Although the participants in both of the groups did not significantly differ on any of the measured baseline demographic or HRQOL variables, there may have been some selection factors operating. That is, women interested in exercise and group activity may have been more likely to participate in this study. Third, the study lacked an additional no contact control group to account for the social exposure provided to both the exercise and waiting list control groups.

Future studies of stretch band interventions should be tested in populations with specific health conditions to determine the generalizability of the effectiveness of these bands on HRQOL and, obviously, in programs of long duration (i.e., greater than 8 weeks). One of the goals of health care is to improve HRQOL of patients. For older adult patients, it may be sufficient to organize social activities outside of the home to improve HRQOL. However, maintenance of strength may provide the independence necessary to participate in organized external social activities.

References

Emery, C., & Blumenthal, J. (1990). Perceived change among participants in an exercise program for older adults. *The Gerontologist, 30,* 516–521.

Ettinger, W., Jr., Burns, R., Messier, S., Applegate, W., Rejeski, W., Morgan, T., Shumaker, S., Berry, M., O'Toole, M., Monu, J., & Craven, T. (1997). A randomized trial comparing aerobic exercise and resistance exercise with a health education program in older adults with knee osteoarthritis. *Journal of the American Medical Association, 277,* 25–31.

Fiatarone, M., O'Neil, E., Ryan, N., Clements, K., Solares, G., Nelson, M., Roberts, S., Kehayias, J., Lipsitz, L., & Evans, W. (1994). Exercise training and nutritional supplementation for physical frailty in very elderly people. *New England Journal of Medicine, 330,* 1769–1775.

Heath, G. W. (1988). Exercise programming for the older adult. In S. Blair, P. Painter, R. Pate, L. Smith, & C. Taylor (Eds.), *Resource manual for guidelines for exercise testing and prescription: American College of Sports Medicine* (pp. 315–320). Philadelphia: Lea & Febiger.

Jette, A., Harris, B., Sleeper, L., Lachman, M., Heislein, D., Giorgetti, M., & Levenson, C. (1996). A home-based exercise program for nondisabled older adults. *Journal of the American Geriatrics Society, 44,* 644–649.

Kallman, D., Plato, C., & Tobin, J. (1990). The role of muscle loss in the age-related decline of grip strength: Cross-sectional and longitudinal perspectives. *Journal of Gerontology, 45,* M82–M88.

King, A., Taylor, C., & Haskell, W. (1993). Effects of differing intensities and formats of 12 months of exercise training on psychological outcomes in older adults. *Health Psychology, 12,* 292–300.

Layne, J. E., & Nelson, M. E. (1999). The effects of progressive resistance training on bone density: A review. *Medicine and Science in Sports and Exercise, 31,* 25–30.

McCartney, N., Hicks, A., Martin, J., & Webber, C. (1996). A longitudinal trial of weight training in the elderly: Continued improvements in year 2. *Journal of Gerontology, 51A,* B425–B33.

Mihalko, S., & McAuley, E. (1996). Strength training effects on subjective well-being and physical function in the elderly. *Journal of Aging and Physical Activity, 4,* 56–68.

Morganti, C., Nelson, M., Fiatarone, M., Dallal, G., Economos, C., Crawford, B., & Evans, W. (1995). Strength improvements with 1 year of progressive resistance training in older women. *Medicine and Science in Sports and Exercise, 27,* 906–912.

Noble, B. (1982). Clinical applications of perceived exertion. *Medicine and Science in Sports and Exercise, 14,* 406–411.

Parker, M. G., Thorslund, M., Lundberg, O., & Kareholt, I. (1996). Predictors of physical function among the oldest old. *Journal of Aging and Health, 8,* 444–460.

Pratley, P., Nicklas, B., Rubin, M., Miller, J., Smith, A., Smith, M., Hurley, B., & Goldberg, A. (1994). Strength training increases resting metabolic rate and norepinephrine levels in healthy 50- to 65-yr-old men. *Journal of Applied Physiology, 76,* 133–137.

Pyka, G., Lindenberger, E., Charette, S., & Marcus, R. (1994). Muscle strength and fiber adaptations to a year-long resistance training program in elderly men and women. *Journal of Gerontology, 49,* M22–M27.

Simonsick, E., Lafferty, M., Phillips, C. , Mendes de Leon, C., Kasl, S., Seeman, T., Fillenbaum, G., Hebert, P., & Lemke, J. (1993). Risk due to inactivity in physically capable older adults. *American Journal of Public Health, 83,* 1443–1450.

Stewart, A., & King, A. (1991). Evaluating the efficacy of physical activity for influencing quality-of-life outcomes in older adults. *Annals of Behavioral Medicine, 13,* 108–116.

Stewart, A., King, A., & Haskell, W. (1993). Endurance exercise and health-related quality of life in 60–65 year old adults. *The Gerontologist, 33,* 782–789.

Stewart, A., & Ware, J. (Eds.). (1992). *Measuring functioning and well-being.* Durham, NC: Duke University Press.

VanGalen, P. A. (1995). *Exercising with Dynaband resistive exerciser.* Akron, OH: The Hygenic Corporation.

Received September 18, 1998
Accepted August 24, 1999

Downloaded from https://academic.oup.com/gerontologist/article/39/6/705/593459 by University of Electronic Science & Tech user on 23 July 2022
