# Supplementary material for: Effects of elastic band resistance training on the physical and mental health of elderly individuals: A mixed methods systematic review
Source: PLoS One. 2024 May 13;19(5):e0303372. doi: 10.1371/journal.pone.0303372 (PMC11090353; doi:10.1371/journal.pone.0303372)
Supplement: S1 File — (ZIP) [file pone.0303372.s001.zip › Supporting Information/Included study 44.pdf]

J. Phys. Ther. Sci. 28: 2793–2797, 2016

The Journal of Physical Therapy Science

Original Article

Effects of 24-week resistance exercise training on carotid peak systolic and end diastolic flow velocity in healthy older adults

Jinkee Park[1)]

[1)] *Institute of Taekwondo for Health and Culture, Dong-A University: 840 Hadan 2-Dong, Saha-Gu, Busan 604-714, Republic of Korea*

Abstract. [Purpose] The aim of this study was to examine the effect of resistance exercise on carotid intima-media thickness, luminal diameter, peak systolic flow velocity, end diastolic flow velocity, and wall shear rate in healthy elderly men. [Subjects and Methods] Thirty healthy elderly men (age ≥65 years) were randomly divided into a control (n=15) and resistance exercise (n=15) groups. The 24-week exercise intervention consisted of 3 days of resistance exercise per week using an elastic band per week. Body composition, physical function, blood pressure, and carotid variables were measured at baseline and after 24 weeks. [Results] Body fat percent, skeletal muscle mass, systolic blood pressure, grip strength, arm curl, chair stand up, sit and reach, maximum walking speed, time up and go, and two-minute step test showed significant interaction. Peak systolic flow velocity, end diastolic flow velocity, and wall shear rate also showed significant interaction. [Conclusion] A 24-week resistance exercise program, using elastic bands, effectively improves carotid flow velocity and wall shear rate in healthy elderly men.

Key words: Carotid flow velocity, Elderly men, Resistance exercise

(This article was submitted Apr. 14, 2016, and was accepted Jun. 16, 2016)

INTRODUCTION

Negative change within the carotid artery is reportedly an independent risk factor for cardiovascular disease (CVD) and stroke[1)]. Several studies report an association of CVD risk with increased of carotid intima-media thickness (CIMT)[2–4)] and luminal diameter (CLD)[5, 6)]. Recently, a followed-up study reported that low carotid artery flow velocity (i.e. peak-systolic velocity [PSV] and end-diastolic velocity [EDV]) is associated with CVD risk in hypertensive patients[7)]. Another study focusing on risk factor evolution and CVD development demonstrated that low PSV and EDV were independently associated with future CVD[8)]. A cross-sectional study comparing patients with ischemic stroke patients and subjects without stroke found an association between lower carotid flow velocity (FV) and increased stroke risk[9)]. Several cross-sectional studies reported that improved of oxygen uptake and walking speed is associated with preventing to the negative change of carotid arteries (in CIMT or CLD) in elderly[3, 10)].

Resistance exercise is effectively improves of physical performance such as muscle strength, agility and dynamic balance, flexibility, walking ability, and aerobic endurance in the elderly[11–13)]. Additionally, resistance exercise can improve body composition, including decreasing body fat mass or increasing of lean body mass in elderly populations[11, 12)]. In contrast, studies on the effect of resistance exercise on arterial function demonstrated that resistance exercise improves vascular endothelial function and increases blood flow velocity in the brachial artery in healthy overweight women and young adults[14, 15)]. Also, resistance exercise is reported to decrease the CIMT and increase the CLD in the carotid artery in patients with chronic heart failure patients[16)]. However, the effects of resistance exercise on carotid artery FV parameters are not yet known in healthy elderly men. Therefore, we examined the effect of a 24 weeks resistance exercise program on carotid artery flow velocity in this population.

Correspondence author. Jinkee Park (E-mail: park7166@dau.ac.kr)

©2016 The Society of Physical Therapy Science. Published by IPEC Inc.
This is an open-access article distributed under the terms of the Creative Commons Attribution Non-Commercial No Derivatives (by-nc-nd) License <http://creativecommons.org/licenses/by-nc-nd/4.0/>.

Table 1. The anthropometric characteristics, body composition and blood pressure of subjects

Variables	Control group (n=15)	Exercise group (n=15)
Age (years)	70.9 ± 3.9	73.1 ± 3.0
Height (cm)	164.9 ± 7.5	164.8 ± 5.4
Body mass (kg)	64.5 ± 8.0	66.0 ± 6.1
Body mass index (kg/m^2)	23.8 ± 2.9	24.3 ± 2.1
Percent body fat mass (%)	23.7 ± 7.1	25.6 ± 6.2
Skeletal muscle mass (kg)	27.3 ± 3.0	27.4 ± 3.7
Systolic blood pressure (mmHg)	127.7 ± 6.7	129.1 ± 7.4
Diastolic blood pressure (mmHg)	74.4 ± 8.0	75.1 ± 8.2

Values are means ± SD

SUBJECTS AND METHODS

In this study initially enrolled 42 healthy elderly men (age ≥65 years) without hypertension, hyperlipidemia, diabetes, history of CVD, or other diagnosed illness. Twelve subjects were excluded for smoking (n=7), and regular participation in physical activity within the past 6 months (n=5). The remaining 30 elderly men were randomly divided into a control group (n=15) and a resistance exercise group (n=15). Table 1 shows the participants' physical characteristics. In accordance with the ethical standards of the Declaration of Helsinki, informed consent was obtained from all participants after they were provided with a detailed description of the study.

After measuring the subjects' height and body mass, their body fat mass (FM) percent and skeletal muscle mass (SM) were measured by bioelectrical impedance analysis, using a body composition analyzer (InBody 370, Biospace, Seoul, South Korea). Body mass index (BMI) was calculated as weight (kg) divided by height squared (m^2). For the 2 days prior to measurement of body composition, subjects were asked to not engage in physical activities other than the activities they usually performed and to refrain from drinking or eating excessively. Systolic blood pressure (SBP) and diastolic blood pressure (DBP) were measured using a mercury sphygmomanometer (HICO, Tokyo, Japan) after 10 minutes of rest.

Upper and lower body muscle strength, agility/dynamic balance, flexibility, walking ability, and aerobic endurance were measured after a 10-minute warm up. Muscle strength was measured by grip strength using a grip strength dynamometer (TKK-5401, Japan) for the upper body and chair stand up for the lower body. Chair stand up were scored based on the number of repetitions completed within 30 seconds. Agility and dynamic balance was measured by the time up and go (TUG, 2.44 m). Participants were informed that this was a timed test and that the objective was to walk as quickly as possible (without running) around a cone and back to the chair. Flexibility was measured by the sit and reach test. Participants were instructed to take a deep breath and then breathe out while leaning forward as far as possible, without bending the knees. Participants walked as quickly as possible without running the 14 m distance. In addition, participants started walking two meters before the start line so that walking speed did not include the acceleration time. And maximum walking speed was defined as the ratio between distance and time. Two-minute steps was measured as an alternative to the aerobic endurance test. Participants' score was the number of full steps completed in 2 minutes, raising each knee to a point midway between the patella and iliac crest.

Resistance exercises used in this study conformed to the guidelines of physical activity recommended for the elderly[17]. The resistance exercise was performed with green elastic exercises bands (Thera-Band, Ohio, USA) for 30–50 minutes per session, 3 days per week, with 10–15 repetitions per set (weeks 1–12: 10–12 repetitions per set; weeks 13–24: 13–15 repetitions per set), 3–5 sets (weeks 1–12: 3 sets, weeks 13–24: 5 sets, 1 minute rest between sets) for 14 items (elbow flexion, lateral raise, shoulder flexion and extension, biceps curl, chest press and flies, abdominal crunch, trunk extension, side bend, mini squat, leg press, calf raise, and ankle inversion)[17, 18]. Participants performed a 10-minute warm and cool down before and after the exercise program, respectively.

Carotid artery variables were measured using B-mode ultrasound and a 10 MHz probe (LOGIQ 3, GE Healthcare, Wauwatosa, WI, USA). The left carotid was then measured by ultrasound. CIMT and systolic CDL were measured from the far wall of the distal common carotid 1 cm proximal to the carotid bifurcation. The CIMT was defined as the distance from the lumen-intima interface to the intima-adventitia interface, and systolic CLD was defined as the distance between the near and far wall intima-media interfaces[19]. For Doppler spectral analysis, peak systolic flow velocity (PSV) and end diastolic flow velocity (EDV) were measured by continuous-wave Doppler examination in the common carotid 1–3 cm proximal to the bifurcation[8]. Wall shear rate (WSR) was calculated using to the following formula[20]:

Table 2. Changed of body composition, blood pressure, physical function, and carotid artery variables in baseline and after 24 weeks

Variables	Control group (n=15)		Change	Exercise group (n=15)		Change
	Baseline	24 week		Baseline	24 week	
Body fat mass percent (%)	23.7 ± 7.1	23.8 ± 6.6	0.1	25.6 ± 4.3	25.0 ± 3.6	−1.2*†
Skeletal muscle mass (kg)	27.3 ± 3.0	27.0 ± 3.0	−0.3	27.4 ± 3.7	28.2 ± 3.7	0.8**††
Systolic blood pressure (mmHg)	127.7 ± 6.7	127.9 ± 6.3	0.2	129.1 ± 7.4	128.3 ± 6.1	−0.8*†
Diastolic blood pressure (mmHg)	74.4 ± 8.0	74.5 ± 6.9	0.1	75.1 ± 8.2	74.9 ± 7.4	−0.4
Left grip strength (kg)	33.3 ± 5.5	32.9 ± 5.4	−0.4	31.0 ± 6.6	33.0 ± 5.5	2.04**††
Right grip strength (kg)	33.2 ± 5.2	32.9 ± 5.0	−0.3	31.2 ± 7.1	34.3 ± 5.8	3.1**†††
Chair stand up (repetitions/30 sec)	16.3 ± 3.6	16.1 ± 3.0	−0.2	16.3 ± 3.4	18.2 ± 3.1	1.9**††
Sit and reach (cm)	10.0 ± 6.1	9.9 ± 6.0	−0.1	8.9 ± 7.8	9.8 ± 7.5	0.9**†
Maximum walking speed (m/sec)	1.67 ± 0.3	1.64 ± 0.3	−0.03	1.58 ± 0.2	1.67 ± 0.3	0.09**††
Time up and go (sec)	5.84 ± 1.0	5.86 ± 1.0	0.02	5.87 ± 1.1	5.65 ± 0.8	−0.22*†
Two-minute step test (repetitions)	101.4 ± 13.1	101.3 ± 12.1	−0.1	99.3 ± 15.6	103.1 ± 12.9	3.9**††
Carotid intima media thickness (mm)	0.71 ± 0.11	0.71 ± 0.10	0.0	0.71 ± 0.08	0.71 ± 0.07	0.0
Systolic carotid luminal diameter (cm)	0.62 ± 0.1	0.62 ± 0.1	0.0	0.63 ± 0.1	0.64 ± 0.1	0.01
Peak systolic flow velocity (cm/sec)	63.4 ± 9.9	62.6 ± 11.1	−0.8	62.2 ± 13.9	71.4 ± 13.0	9.2**†††
End diastolic flow velocity (cm/sec)	20.4 ± 3.8	20.5 ± 3.8	0.1	19.2 ± 3.7	21.0 ± 4.0	1.8**†
Wall shear rate (s^{-1})	409.6 ± 65.3	403.7 ± 74.6	−5.9	403.1 ± 108.1	453.9 ± 116.7	50.8*†

Values are means ± SD.
Significantly different within group: *p<0.05, **p<0.01; Significant group × time interaction: †p<0.05, ††p<0.01, †††p<0.001

$$WSR_{peak}=4\times V_{peak}/CLD$$

Where WSR is wall shear rate (s^{-1}), Vpeak is the peak systolic flow velocity and CLD is systolic luminal diameter.

Statistical Package for the Social Sciences (SPSS) ver. 17.0 (SPSS Inc., Chicago, IL, USA) was used for statistical analysis, and results reported as mean and standard deviation. The Student's t-test was used to assess differences in baseline variables. Two-way factorial analysis of variance was used to compare parameters at baseline and after the 24 weeks of the study participation. When significant group × time interactions were identified, paired t-tests were performed to detect differences between time points. Statistical significance was set at p<0.05.

RESULTS

Table 2 shows changes of body composition, blood pressure, physical function, and carotid variables in baseline and after 24 weeks. FM percent (p<0.05), SM (p<0.01), and SBP (p<0.05) showed significant interactions. Grip strength on the left (p<0.01) and right (p<0.001), chair stand up (p<0.01), sit and reach (p<0.05), maximal walking speed (p<0.01), TUG (p<0.01), and two-minute step (p<0.01) had significant interactions. PSV (p<0.001), EDV (p<0.05), and WSR (p<0.05) showed significant interactions. However, in the DBP, CIMT, and CLD, interactions were no identified.

In the paired t-test results of the exercise group, FM percent, SBP, and TUG were significantly decreased (p<0.05, each), and SM, left and right grip strength, chair stand up, sit and reach, maximal walking speed, and two-minutes step were significantly increased (p<0.01, each), also PSV (p<0.01), EDV (p<0.01), and WSR (p<0.05) were significantly increased after 24 weeks. Body composition, blood pressure, and physical function variables showed no change in the control group. But in exercise group, DBP, CIMT, and CLD were no change, also in control group, all variables were no change.

DISCUSSION

This study examined the effects of a 24-week resistance exercise program on carotid parameters in elderly men. The primary findings of this study are that the resistance exercise program increased PFV, EDV, and WSR in this study population.

Decreased of carotid artery FV is associated with early progress of atherosclerosis, and stoke in elderly patients[9]. In addition, two recently cross-sectional studies reported that low PSV and EDV were independently associated with future DVD[7, 8]. The previous studies suggest that increase of PSV and EDV is associated with a potential prevention for CVD.

Studies on the effects of exercise training on arterial parameters demonstrated that resistance exercise increases blood flow in the femoral artery and increases flow mediated dilation of the brachial artery in adults[14, 21, 22]. Resistance exercise is also reported to increase the flow velocity of the brachial and femoral artery in adults and older subjects[15, 22]. Changes in brachial and femoral parameters due to resistance exercise are considered to significantly improve of peripheral artery function[14, 15]. However, effects of long-term resistance exercise on carotid artery FV have not been confirmed. Several studies reported

that carotid FV is increased during dynamic exercise or after acute high intensity exercise in young adults[23, 24]. A study of obese adults reported that resistance exercise alone could improve brachial artery endothelial function[14]. In the present study, resistance exercise was shown to increase both PSV and EDV. This study demonstrated that long-term resistance exercise improves to age-related carotid FV in health elderly men. Aging is reported as a major risk factor for decreased of carotid artery flow[25]. In the elderly, improvement of carotid FV by exercise is considered as a potential prevention on CVD.

Low WSR is associated with increased CIMT, and is directly associated with changes in carotid FV, viscosity, and CLD[26]. In the current study, WSR was used as a surrogate marker of wall shear stress, and was calculated by systolic CLD and PSV[26]. We found that exercise resulted in increased PSV, but there was no change in CLD. Therefore, increased of WSR is considered an effect of increased carotid FV. Two intervention studies in overweight or obese adults demonstrated that short- or long-term resistance training is not effective in improving CIMT and CLD[14, 21]. In this study, resistance exercise after 24 weeks was not changed in CIMT and CLD. This result suggests that resistance exercise does not effectively improve CIMT and CLD in health elderly.

Two intervention studies demonstrated that resistance exercise using weightlifting machine is effectively improves of body composition and physical function in the elderly[11, 12]. Other intervention study demonstrated that resistance exercise using elastic band is effectively improves physical fitness such as muscle function and balance ability in elderly women[13]. And resistance exercise using elastic band are simple and economical, and have safety advantages[13]. In this study, resistance exercise was to use elastic bands, and health-related indicators such as blood pressure as well as body composition and physical fitness variables were examined. This study showed that resistance exercise using elastic bands for 24 weeks is effectively improves of blood pressure as well as body composition and physical fitness variables in elderly.

This study did not examine the blood biochemical factors associated with cardiovascular disease. This is a limitation of the study. And the subjects was limited to elderly men. Therefore, effects of resistance exercise on the carotid artery may be limited to elderly men.

In conclusion, 24-week resistance exercise program, using elastic bands, effectively improves carotid flow velocity and wall shear rate in healthy elderly men.

ACKNOWLEDGEMENT

The study was supported by the Dong-A University Research fund.

REFERENCES

1) Dijk JM, van der Graaf Y, Bots ML, et al.: Carotid intima-media thickness and the risk of new vascular events in patients with manifest atherosclerotic disease: the SMART study. Eur Heart J, 2006, 27: 1971–1978. [Medline] [CrossRef]
2) Takato T, Yamada N, Ashida T: Effects of aging and sex on progression of carotid intima-media thickness: a retrospective 6-year follow-up study. Geriatr Gerontol Int, 2008, 8: 172–179. [Medline] [CrossRef]
3) Hamer M, Kivimaki M, Lahiri A, et al.: Walking speed and subclinical atherosclerosis in healthy older adults: the Whitehall II study. Heart, 2010, 96: 380–384. [Medline] [CrossRef]
4) Park JK, Park H, Kim KB: The relationship between distribution of body fat mass and carotid artery intima-media thickness in Korean older adults. J Phys Ther Sci, 2015, 27: 3141–3146. [Medline] [CrossRef]
5) Eigenbrodt ML, Sukhija R, Rose KM, et al.: Common carotid artery wall thickness and external diameter as predictors of prevalent and incident cardiac events in a large population study. Cardiovasc Ultrasound, 2007, 5: 11. [Medline] [CrossRef]
6) Miyamoto M, Kotani K, Okada K, et al.: The correlation of common carotid arterial diameter with atherosclerosis and diabetic retinopathy in patients with type 2 diabetes mellitus. Acta Diabetol, 2012, 49: 63–68. [Medline] [CrossRef]
7) Bellinazzi VR, Cipolli JA, Pimenta MV, et al.: Carotid flow velocity/diameter ratio is a predictor of cardiovascular events in hypertensive patients. J Hypertens, 2015, 33: 2054–2060. [Medline] [CrossRef]
8) Chuang SY, Bai CH, Cheng HM, et al.: Common carotid artery end-diastolic velocity is independently associated with future cardiovascular events. Eur J Prev Cardiol, 2016, 23: 116–124. [Medline] [CrossRef]
9) Bai CH, Chen JR, Chiu HC, et al.: Lower blood flow velocity, higher resistance index, and larger diameter of extracranial carotid arteries are associated with ischemic stroke independently of carotid atherosclerosis and cardiovascular risk factors. J Clin Ultrasound, 2007, 35: 322–330. [Medline] [CrossRef]
10) Gando Y, Yamamoto K, Kawano H, et al.: Attenuated age-related carotid arterial remodeling in adults with a high level of cardiorespiratory fitness. J Atheroscler Thromb, 2011, 18: 248–254. [Medline] [CrossRef]
11) Binder EF, Yarasheski KE, Steger-May K, et al.: Effects of progressive resistance training on body composition in frail older adults: results of a randomized, controlled trial. J Gerontol A Biol Sci Med Sci, 2005, 60: 1425–1431. [Medline] [CrossRef]
12) Fatouros IG, Kambas A, Katrabasas I, et al.: Strength training and detraining effects on muscular strength, anaerobic power, and mobility of inactive older men are intensity dependent. Br J Sports Med, 2005, 39: 776–780. [Medline] [CrossRef]
13) Lee HC, Lee ML, Kim SR: Effect of exercise performance by elderly women on balance ability and muscle function. J Phys Ther Sci, 2015, 27: 989–992. [Medline] [CrossRef]
14) Olson TP, Dengel DR, Leon AS, et al.: Moderate resistance training and vascular health in overweight women. Med Sci Sports Exerc, 2006, 38: 1558–1564.

[Medline] [CrossRef]
15) Okamoto T, Masuhara M, Ikuta K: Effect of low-intensity resistance training on arterial function. Eur J Appl Physiol, 2011, 111: 743–748. [Medline] [CrossRef]
16) Maiorana AJ, Naylor LH, Exterkate A, et al.: The impact of exercise training on conduit artery wall thickness and remodeling in chronic heart failure patients. Hypertension, 2011, 57: 56–62. [Medline] [CrossRef]
17) Nelson ME, Rejeski WJ, Blair SN, et al.: Physical activity and public health in older adults: recommendation from the American College of Sports Medicine and the American Heart Association. Med Sci Sports Exerc, 2007, 39: 1435–1445. [Medline] [CrossRef]
18) Zion AS, De Meersman R, Diamond BE, et al.: A home-based resistance-training program using elastic bands for elderly patients with orthostatic hypotension. Clin Auton Res, 2003, 13: 286–292. [Medline] [CrossRef]
19) Schott LL, Kao AH, Cunningham A, et al.: Do carotid artery diameters manifest early evidence of atherosclerosis in women with rheumatoid arthritis? J Womens Health (Larchmt), 2009, 18: 21–29. [Medline] [CrossRef]
20) Jiang Y, Kohara K, Hiwada K: Association between risk factors for atherosclerosis and mechanical forces in carotid artery. Stroke, 2000, 31: 2319–2324. [Medline] [CrossRef]
21) Schjerve IE, Tyldum GA, Tjønna AE, et al.: Both aerobic endurance and strength training programmes improve cardiovascular health in obese adults. Clin Sci (Lond), 2008, 115: 283–293. [Medline] [CrossRef]
22) Anton MM, Cortez-Cooper MY, DeVan AE, et al.: Resistance training increases basal limb blood flow and vascular conductance in aging humans. J Appl Physiol 1985, 2006, 101: 1351–1355. [Medline] [CrossRef]
23) Sato K, Ogoh S, Hirasawa A, et al.: The distribution of blood flow in the carotid and vertebral arteries during dynamic exercise in humans. J Physiol, 2011, 589: 2847–2856. [Medline] [CrossRef]
24) Babcock MC, Lefferts WK, Hughes WE, et al.: Acute effect of high-intensity cycling exercise on carotid artery hemodynamic pulsatility. Eur J Appl Physiol, 2015, 115: 1037–1045. [Medline] [CrossRef]
25) Scheel P, Ruge C, Schöning M: Flow velocity and flow volume measurements in the extracranial carotid and vertebral arteries in healthy adults: reference data and the effects of age. Ultrasound Med Biol, 2000, 26: 1261–1266. [Medline] [CrossRef]
26) Malík J, Kudlička J, Tuka V, et al.: Common carotid wall shear stress and carotid atherosclerosis in end-stage renal disease patients. Physiol Res, 2012, 61: 355–361. [Medline]
