# Supplementary material for: Effects of elastic band resistance training on the physical and mental health of elderly individuals: A mixed methods systematic review
Source: PLoS One. 2024 May 13;19(5):e0303372. doi: 10.1371/journal.pone.0303372 (PMC11090353; doi:10.1371/journal.pone.0303372)
Supplement: S1 File — (ZIP) [file pone.0303372.s001.zip › Supporting Information/Included study 52.pdf]

©Journal of Sports Science and Medicine (2021) **20**, 133-141
http://www.jssm.org DOI: https://doi.org/10.52082/jssm.2021.133

Research article

Effects of Chair-Based, Low–Load Elastic Band Resistance Training on Functional Fitness and Metabolic Biomarkers in Older Women

Marko D.M Stojanović [1,2]✉, Mladen Mikić [1,2], Zoran Milošević [1], Jovan Vuković [1], Tatjana Jezdimirović [1] and Vlatko Vučetić [3]

[1] University of Novi Sad, Faculty of Sport and Physical Education, Novi Sad, Serbia; [2] Advanced Rehab & Conditioning Lab, Faculty of Sport and Physical Education, University of Novi Sad, Serbia; [3] Faculty of Kinesiology, University of Zagreb, Croatia

Abstract

Strength training can improve myriad health parameters in elderly cohorts. Although potentially more appropriate for the elderly, low-load resistance training protocols have been less investigated. We aimed to examine the effects of 12 weeks of chair-based, low-load resistance training with elastic band (EBT) on functional fitness and metabolic biomarkers in older women. One hundred sixty-eight women were allocated randomly to an elastic band resistance training (EBT, n = 86, 75.7 ± 8.9 years, 71.3 ± 12.2 kg) or a control group (CON, n = 82, 74.5 ± 8.2years, 70.6 ± 12.0 kg). RT protocol consisted of periodized chair-based, low-load whole-body resistance exercises (2 sets, 12-15 repetitions, 40-60% of one repetition maximum-1RM) using an elastic band, twice weekly for 12 weeks. The resistance training program was generally designed to maintain internal load over time, provided with increasing intensity using various elastic bands (Thera-Band). Functional fitness (30-s Chair Stand,30-s Arm Curl, 2-min Step Test, Chair Sit-and-Reach, Back Scratch, 8-Foot Up-and-Go, Handgrip Strength) and metabolic markers (Fasting blood glucose, triglycerides, total cholesterol, high (HDL) and low (LDL) density lipoprotein) were measured before and after the training period. To detect pre/post intervention changes and between group- differences 2x2 repeated measures ANOVA was applied. Significant improvements over time for all fitness variables for EBT comparing to CON were obtained ($F = 12.78$, $p < 0.05$ for 30-s Chair Stand; $F = 14.04$, $p < 0.05$ for 30-s Arm Curl; $F = 5.18$, $p < 0.05$ for 2-min Step Test; $F = 10.90$, $p < 0.05$ for Chair Sit-and-Reach; $F = 16.57$, $p < 0.05$ for Back Scratch; $F = 11.79$, $p < 0.05$ for 8-foot Up-and-Go; and $F = 29.25$, $p < 0.05$ for Handgrip Strength). In addition, significant improvements over time for all but one (triglycerides) biomarkers for EBT comparing to CON were obtained ($F = 7.30$, $p < 0.05$ for blood sugar levels; $F = 13.36$, $p < 0.05$ for total cholesterol; $F = 8.61$, $p < 0.05$ for HDL; and $F = 11.53$, $p < 0.05$ for LDL). Furthermore, the participants' adherence to training sessions of over 90% was reported. In conclusion, 12 weeks of EBT is safe and beneficial for improving health-related fitness and metabolic biomarkers in older women and seems to be viable model to ensure a high training adherence rate.

Key words: Aging, lipoproteins, low-intensity strength training, physical function.

Introduction

Aging is an irreversible process accompanied by muscle volume and strength loss, and their preservation is considered an essential prerequisite for health, functional independence and wellbeing of elderly people (Manini and Clark, 2012). Major biochemical changes with increasing age are strongly related to cardiovascular disease, the leading health issue in elderly subjects (Zaslavsky and Gus, 2002). Between them, negative alterations in serum blood glucose (GLU) and lipid fractions were proved to impact cardiovascular disease risk substantially, especially in women (Tan et al., 2010).

Resistance training has been proved to increase strength and consequently counteract age-related dysfunctions in functional capacity and biomarkers profile (Aagaard et al., 2010). Linear relationship between strength training load and an increase in muscle size and strength in older women has been purported (Ribeiro et al., 2018). However, resistance training modes applying high loads (above 65% of 1 repetition maximum) both increase a risk of injury or overuse during training (Liu and Latham, 2010) and resulted in reduced physical function 48 hours after training (Orssatto et al., 2018) in elderly, hindering its potential use in this specific population. Previous studies indicated that strength-naïve women tend to respond similarly to different training stimuli (Colado and Triplett, 2008), with low-load elastic band training regularly found to provide superior adherence to usual resistance training program with weight machines. In addition, elastic band training likely provides more benefits compared to free-weight training, such as hindered injury risk, enhanced functional strength, and ability to change muscle emphasis during exercises (Melchiorri and Rainoldi, 2011). Thus, the effectiveness of alternative, low-load elastic band resistance training methods warrant further investigation.

Resistance training with elastic bands has lately become popular and proposed as a reasonable alternative to traditional free weights or resistance machines strength training (Colado and Triplett, 2008; Gene-Morales et al., 2020; Flandez et al.,2017). Elastic band resistance training has been growingly used since it is more versatile and accessible for various age-group individuals, allows functional movement patterns and can easily be conducted in different clinical conditions (Martins et al., 2015). Elastic bands are portable, less expensive, allow one to exercise in safe manner easily adjusting resistance (Colado et al., 2020). The use of elastic bands provides a variable load throughout a range of motion with the most resistance experienced at or near full muscular extension, thus better mimicking length-tension relationship of most muscles in the body (Wilson and Kritz, 2014). In addition, its use as a

Received: 05 October 2020 / Accepted: 08 January 2021 / Published (online): 01 March 2021

resistive modality has been reported to be similarly effective in muscle activation of prime-movers and superior in muscle activation of synergists compared to weight machines, for both single-joint and multiple-joint exercise (Jakobsen et al., 2012; 2014; Calatayud et al., 2015; Bergquist et al., 2018). The use of elastic bands seems very advantageous in elderly populations since even strength training -naïve persons can easily implement this effective training method proved to induce significant beneficial short-term effects (Martins et al., 2013). Resistance training with elastic bands has also shown high acceptance in elderly (Fahlman et al., 2011). In addition, chair-based exercise was proved to be an accessible training method for sedentary elderly subjects with beneficial effects on various fitness and function parameters (Robinson et al., 2014). Generally, it incorporates organized, load-increasing chair-supported exercises that minimize balance problem, accounting individual limitations and enabling participants to work out without the usual safety-related constraints (Furtado et al., 2016). While aerobic-oriented chair-based programs have been proved effective for cognition, balance and cardiorespiratory fitness in elderly adults (DeSure et al., 2013), effectiveness of chair-based resistance training exercise programs has been studied less extensively. Rieping et al. (2019) showed that 14-week chair-based resistance training improved functional autonomy in elders but with largely unaffected physical fitness (except 30s chair stand test). Contrary to this finding, Liao et al. (2018) reported significant increases in physical capacity of older women following 12 weeks of chair based elastic resistance routine.

Taking all the aforementioned into account, we aimed to investigate the effects of chair-based, low load resistance training with elastic bands (EBT) on functional fitness and metabolic biomarkers in older women. It was hypothesized that EBT would improve functional fitness and metabolic biomarkers in older women.

Methods

Study design

This study was a single blind randomized controlled trial conducted in a geriatric centers in Vojvodina, Serbia. A total of 6 institutions (Zrenjanin, Sombor, Subotica, B.Palanka, Ruma, Novi Sad) were chosen based on their acclaimed interest in cooperating projects related to exercise and health for elderly. An introductory lecture was organized in each center, with benefits of strength training for health in general, study objectives and measurements procedures presented to potential study-participants. After the lecture, female residents volunteered for the study and proceed to medical checking for suitability to practice resistance training. The medical check-ups have been conducted for two consecutive days for every institution. Among 238 subjects expressing interest, 40 were excluded because one of the following exclusion criteria: 1. Orthopaedic disorder, 2. Neurologic disorder, 3. Rheumatologic disorder 4. Cardiovascular disorder and 5. Participation in organized physical activity for past 2 years. Another 18 subjects lost their interest after detailed explanation about study design. Altogether, 180 subjects signed the consent form and were randomly allocated to an experimental EBT group (n = 90) or an age-matched control group (n = 90). In the week following medical checking, subjects underwent testing, with familiarisation session at least 48 hours before pre-test. Testing took place in the early mornings (7.30 a.m.), and started with blood sampling, followed by height and body mass measurements (Tanita model BF-350), according to previously published protocols (Colado and Triplett, 2008). Thereafter, 45 min rest was allowed (breakfast, medications), followed by functional fitness test battery for older adults (Rikli and Jones, 2013, chapter 4) with addition of handgrip strength test. Following 12-week training period, subject underwent second testing. Experimental group followed structured resistance training program, while for control group, exclusively institution's activities including chess, dice, reading, crafts etc. were considered. The women and institution supervisors were all told to maintain their behavior and diet during study. Same technicians, equipment and testing procedures were arranged for each subject for all pre- and post-training measurements. Testing procedures for all subjects were done at a standardized room conditions (temperature around 22 Cº and humidity around 50%) during the same week for both pre and post training measurements. The examiners (15 in total, 3 teams of 5) were blinded to group allocation, appropriately trained and qualified. Overall, 168 women finished the study with results included in further analyses. Data were collected between February 1, 2019 and Jun 1, 2019. This study was approved by the Ethics Committee of the University of Novi Sad (Ref. No. 44-01-03/2018-6).

Instruments

Participants were strongly instructed to avoid exercise and alcohol/caffein-containing drinks 24 hours and 3 days prior to testing, respectively. The blood was collected and stored in serum separator tubes by a physician after overnight fasting, from the superficial veins of the upper limb. After storage, serum separation was provided with blood samples centrifuged for 10 min at 3000 rpm. Serum levels of glucose (GLU), total cholesterol (TC), high-density lipoprotein (HDL-C), low-density lipoprotein (LDL-C) and triglycerides (TG) were measured in an accredited laboratory at the city clinic in Novi Sad. The biochemical analyses were conducted using an automatic biochemistry analyzer system (MINDRAY BS 800-Bio-Medical Electronics Co.; Shenzhen, China) according to protocol. Friedewald equation was used for the estimation of Low-density lipoprotein (LDL)-cholesterol level (Friedewald et al., 1972).

Senior Fitness Test battery (SFTB) with addition of handgrip strength test was used. SFTB is a 6 item field-based test battery designed and validated to assess functional fitness attributes (strength, flexibility, dynamic balance and cardiorespiratory fitness) for seniors (Rikli and Jones, 2013). The following tests were conducted: Handgrip Strength Test (kg-left+right), 30-s Chair Stand (repetitions), 30-s Arm Curl (repetitions), Chair Sit-and-Reach (centimeters), Back Scratch (centimeters), 2.4m Up-and-Go (seconds), and 2-minute Step Test (number of knee rises in 2 min period), respectively. Handgrip Strength Test was included as general biomarker of health (Bohannon,

2019) and conducted as suggested by Fess (1992), while Senior Fitness Test was carried out as suggested by authors (Rikli and Jones, 2013- chapter 4). Participants performed 5- to 10-minute warm-up routine before commencing the test. All SFTB procedures, apart from the 2-minute step test, were executed twice, with the better result recorded. For isometric handgrip test the highest sum (left+right) out of 3 trials, separated by 1-minute recovery period was used for statistical analysis. The reproducibility of SFTB and handgrip strength test was assessed in a test-retest protocol (pre-experimental study) in a sample of 25 randomly selected participants from Novi Sad. We found intraclass correlation coefficients between 0.84-0.93 for all items, indicating very high reproducibility for selected functional fitness measurements.

Training protocol

The experimental group received 12 weeks of elastic band resistance training, organized in groups with up to 10 patients and monitored by 2 qualified instructors. Training program consisted of 12 chair-based exercises for knee/hip/shoulder/elbow/trunk extension and flexion, hip abduction and adduction (Table 1).

Table 1. Resistance training exercises

Exercise	Sets	Reps	Cadence	Rest	RPE-O
**Chair EB knee extension**	2	12-15	3s+3s	1 min	4-5
**Chair EB knee flexion**	2	12-15	3s+3s	1 min	4-5
**Chair EB bent row**	2	12-15	3s+3s	1 min	4-5
**Chair EB chest press**	2	12-15	3s+3s	1 min	4-5
**Chair EB unilateral hip flexion**	2	12-15	3s+3s	1 min	4-5
**Chair spine twist extension arm (oblique's)**	2	12-15	3s+3s	1 min	4-5
**Chair upright row**	2	12-15	3s+3s	1 min	4-5
**Chair EB trunk flexion**	2	12-15	3s+3s	1 min	4-5
**Chair EB Biceps arm curl**	2	12-15	3s+3s	1 min	4-5
**Chair EB overhead triceps extension**	2	12-15	3s+3s	1 min	4-5
**Chair EB hip abduction**	2	12-15	3s+3s	1 min	4-5
**Standing EB hip extension**	2	12-15	3s+3s	1 min	4-5

RPE-O = Rating of perceived exertion, OMNI-scale.

Light intensity resistance bands (Thera-Bands, The Hygenic Corporation, Akron, OH, USA) with a relaxed length of 1.5 m were used as exercise equipment in all exercises. Every participant was provided with EB which they used exclusively. All exercises were performed with continuously tensioned band during eccentric portion of the movement and until the predetermined range of motion reached for concentric phase, using appropriate grip width. Training sessions consisted of approximately 10 min of warm-up, followed by 40 min of EBT and 5-10 min of cooling down. The warm-up consisted of exercise -movement rehearsal, ensuring maximal range-of-motion of involved muscle groups.

The cool-down included several stretching exercises (one for each muscle group involved), with 2*20-30s of static stretching. Structured program with standardized volume (12-15 repetitions * 2 sets), intra-set rest periods (1 min), frequency (twice per week) and movement cadence (3 s for both shortening and lengthening phase without rest between phases and repetitions) was applied. Training intensity ranged from 4 to 5 on OMNI Resistance for active muscle scale, which has been previously validated for older adults (Colado et al., 2018) and corresponds to 40% to 50% of 1RM (Lagally and Robertson, 2006). The concept was to keep internal load throughout the study constant with the aim of quantifying low load training- effects on measured outcomes. Consequently, as the participants increased the level of strength during study, we changed the external load of training sessions accordingly (Colado et al., 2014; 2018; 2020). During the course of the study, when ratings dropped below 4 conducting predetermined number of repetitions (12-15), we modified the exercise intensity either by decreasing the elastic band grip-width or by changing elastic band colour. The exercise sequencing was altered on a weekly basis with the goal to preserve participants' motivation. Instructor kept a record of training session attendance.

Statistical analysis

Descriptive data are expressed as means ± standard deviation (*SD*). Since Kolmogorov Smirnov test showed all parameters follow normal distribution, parametric statistics were applied. Possible baseline between-group differences were calculated with two-sample Student's *t* test. To detect pre/post intervention changes and respective between group- differences repeated measures ANOVA using 2 groups x 2 time design was applied. When an F-ratio was significant, the Tukey post hoc test was employed to identify significant differences. Cohen's d as the measure of the effect size of the mean difference was calculated by subtracting the means and dividing the result by the pooled standard deviation. A Cohen's d of 0.00–0.19, 0.20–0.49, 0.50 - 0.79 and ≥ 0.80 was considered as trivial, small, moderate, and large, respectively (Cohen, 1988). Percentage of change ([post value/pre value] - 1) was calculated and presented for each variable. Significant differences for all analyses were assumed a when 2-tailed $p \leq 0.05$. The IBM SPSS Statistics version 22 (IBM Corp. Released 2013. IBM SPSS Statistics for Windows, Version 22.0. Armonk, NY: IBM Corp.) was used to analyze all data.

Results

The subjects had excellent adherence to the program, participating in 22 ± 1 of the scheduled 24 training sessions, which is 92% of the total sessions. During study period, no side effects were reported. Baseline difference between the experimental and control group were nonsignificant for demographic parameters (Table 2), while significant baseline differences were established for Glucose, HDL and Back Scratch (Tables 3 and 5) ($p < 0.05$).

There were significant changes for glucose, total cholesterol, HDL and LDL pre vs post in experimental group as well as blood glucose in control group ($p < 0.05$). Significant time*group interactions were obtained for blood sugar levels (F = 7.30; $p < 0.05$), total cholesterol (F = 13.36; $p < 0.05$), HDL (F = 8.61; $p < 0.05$) and LDL (F = 11.53; $p < 0.05$) (Table 3).

The effect sizes were of trivial to small magnitude for glucose (d: EBT. = 0.186; CON = 0.062), total

Table 2. Physical characteristics of subjects. Values are means ± SD.

Characteristics	Experimental (n = 86)	Control (n = 82)	F (p)	TOTAL
Age (years)	75.7 ± 8.9	74.5 ± 8.2	2.21 (.139)	75.1 ± 8.5
Height (m)	1.61 ± 0.06	1.59 ± 0.06	.167 (.648)	160.1 ± 6.4
Body mass (kg)	71.3 ± 12.2	69.9 ± 11.8	.16 (.899)	70.6 ± 12.0

There were no significant differences between groups. F(p)- F values with respective significance level.

Table 3. Biochemical variables in experimental and control group. Values are means ± SD.

Variables	Experimental (n = 86)			Control (n = 82)		
	Pre	Post	Percent change	Pre	Post	Percent change
Glucose (mmol/L)	5.96 ± 1.76	5.64 ± 1.68*†	-5	6.30 ± 2.24	6.44 ± 2.28*	2
Total cholesterol (mmol/L)	6.01 ± 1.29	5.56 ± 1.22*†	-7	6.09 ± 1.42	6.07 ± 1.27	1
HDL (mmol/L)	1.31 ± 0.34	1.37 ± 0.38*†	4	1.50 ± 0.37	1.47 ± 0.40	-2
LDL (mmol/L)	3.90 ± 1.22	3.52 ± 1.04*†	-10	3.84 ± 1.22	3.84 ± 1.15	0
Triglycerides (mmol/L)	1.66 ± 0.74	1.65 ± 0.66	-1	1.68 ± 0.62	1.71 ± 0.65	1

Significant baseline differences between groups for Glucose and HDL were established ($p < 0.05$). HDL – high-density lipoprotein; LDL – low-density lipoprotein. * Indicates significant difference pre- versus post at $p < 0.05$; † significant difference experimental vs. control at $p < 0.05$.

Table 4. Effects sizes, relative effect size and relative effect size-magnitude for all variables.

Variables	Effect size - EBT	Effect size -CON	Relative effect size	Magnitude
Glucose (mmol/L)	0.186	0.062	0.248	Small
Total cholesterol (mmol/L)	0.358	-0.015	0.373	Small
HDL (mmol/L)	0.166	-0.078	0.244	Small
LDL (mmol/L)	0.335	0.000	0.335	Small
Triglycerides (mmol/L)	0.014	0.047	0.061	Trivial
30-s Chair Stand (reps)	0.323	0.154	0.169	Trivial
30-s Arm Curl (reps)	0.378	0.146	0.232	Small
2-min Step Test	0.258	0.089	0.169	Trivial
Chair Sit-and-Reach (cm)	0.315	0.044	0.271	Small
Back Scratch (cm)	-0.291	-0.014	0.277	Small
8-Foot Up-and-Go (s)	-0.227	0.032	0.259	Small
Handgrip Strength (kg)	0.288	0.022	0.266	Small

Table 5. Results of physical fitness in experimental and control group. Values are means ± SD.

Variables	Experimental (n = 86)			Control (n = 82)		
	Pre	Post	Percent change	Pre	Post	Percent change
30-s Chair Stand (reps)	12.2 ± 6.3	14.2 ± 6.1*†	16	11.7 ± 5.2	12.5 ± 5.2*	7
30-s Arm Curl (reps)	17.3 ± 7.1	20.0 ± 7.2*†	39	15.2 ± 5.0	16.0 ± 5.9*	5
2-min Step Test	79.1 ± 33.6	87.8 ±33.8*†	11	81.2 ± 30.0	83.9 ± 30.4*	3
Chair Sit-and-Reach (cm)	0.51 ± 9.4	3.2 ± 7.6 *†	540	0.7 ± 9.1	1.1 ± 9.1	57
Back Scratch (cm)	11.6 ± 12.8	8.1 ± 11.2*†	30	3.0 ± 14.2	2.8 ± 13.8	6
8-Foot Up-and-Go (s)	9.58 ± 6.09	8.34 ± 5.47*†	9	9.03 ± 5.5	9.4 ± 5.1	4
Handgrip Strength (kg)	32.6 ± 15.2	37.1 ± 16.0 *†	14	38.1 ± 17.7	38.5 ± 17.9	1

Significant baseline differences between groups for Back Scratch was established ($p < 0.05$) * Indicates significant difference pre- versus post at $p < 0.05$; † significant difference experimental vs. control at $p < 0.05$.

cholesterol (d: EBT = 0.358; CON = -0.015), HDL (d: EBT. = 0.166; CON = -0.078), LDL (d: EBT = 0.335; CON = 0) and triglycerides (d: EBT. = 0.014; CON = 0.047). In addition, relative effect sizes were also trivial to small magnitude for glucose (d = 0.248), total cholesterol (d = 0.373), HDL (d = 0.244), LDL (d = 0.355) and triglycerides (d = 0.061) (Table 4).

Both groups showed significant change pre vs post in 30-s Chair Stand, 30-s Arm Curl and 2 min Step Test while Chair Sit and Reach, Back Scratch, 8-foot Up and Go and Handgrip Strength Test showed significant change pre vs post in experimental group only. In addition, time*group interactions were obtained for 30-s Chair Stand ($F = 12.78$; $p < 0.05$) , 30-s Arm Curl ($F = 14.04$; $p < 0.05$), 2-min Step Test ($F = 5.18$; $p < 0.05$), Chair Sit-and-Reach ($F = 10.90$; $p < 0.05$) Back Scratch ($F = 16.57$; $p < 0.05$), 8-foot Up-and-Go ($F = 11.79$; $p < 0.05$) and Handgrip Strength ($F = 29.25$; $p < 0.05$) (Table 5).

The effect sizes were of trivial to small magnitude for 30-s Chair Stand (d: EBT. = 0.323; CON = 0.154), 30-s Arm Curl (d: EBT. = 0.378; CON = 0.146), 2-min Step Test (d: EBT. = 0.258; CON = 0.089), Chair Sit-and-Reach (d: EBT = 0.315; CON = 0.044) Back Scratch (d: EBT = 0.291; CON = 0.014), 8-foot Up-and-Go (d: EBT = 0.227; CON = 0.032) and Handgrip Strength (d: EBT = -0.288; CON = 0.022) (Table 3). In addition, relative effect sizes were also trivial-to-small magnitude for 30-s Chair Stand (d = 0.169), 30-s Arm Curl (d = 0.232), 2-min Step Test (d = 0.169), Chair Sit-and-Reach (d = 0.271), Back Scratch (d = 0.277), 8-foot Up-and-Go (d = 0.259) and Handgrip Strength (d = 0.266) (Table 4).

Discussion

The objective of this study was to investigate the effects of chair-based, low load resistance training with elastic bands (EBT) on functional fitness and metabolic biomarkers in older women. Our findings show that EBT can lead to a significant increase in physical fitness (small effect sizes) as well as metabolic biomarkers improvements (trivial to

small effect sizes) including blood sugar, total cholesterol, HDL and LDL in older women. In addition, we observed high adherence rate (92%) which is frequently stated as an important issue considering resistance training interventions in elderly population (Picorelli et al., 2014). Thus, EBT can be regarded as a worthy health-improvements training method in older women. It is widely accepted that strength training in elderly women can substantially improve various health-related biomarkers, physical fitness and overall health (Liu and Latham, 2009; Gargallo et al., 2018; Fritz et al., 2018). In addition, it has previously been observed that chair-based resistance training using elastic band leads to increased functional autonomy and steroid hormone responses, while simultaneously decreases physical frailty and functional disabilities in older women (Furtado et al., 2020; Rieping et al., 2019). No studies to date, however, have discussed the physical fitness and metabolic biomarkers responses following EBT, clearly justifying rationale of this study.

Training protocol effects on metabolic biomarkers

Regular aerobic training has been promoted for decades as a potent tool for lipid-profile improvements (Mann et al., 2014). However, resistance training has also been proved potent to alter level of lipoproteins (Ibáñez et al., 2010), with a number of studies in recent years investigating resistance training effect on metabolic biomarkers in aged women. Conceição et al. (2013) reported that trice-a-week progressive resistance training conducted for 16 weeks and with 10 exercises done with 3 sets of 8-10 repetitions, significantly improves fasting blood glucose and metabolic syndrome Z-score (score including five variables: triglycerides, HDL, blood glucose, waist circumference and mean arterial pressure) in postmenopausal women. Further, no significant changes for HDL and triglycerides were reported, with authors speculated that initial normal range levels of lipoproteins in study subjects are likely responsible for these results. Similarly, total cholesterol and low-density lipoprotein showed a decline while very-low-density lipoprotein, high-density lipoprotein, and triglycerides showed a nonsignificant ($P > 0.05$) increase following a year of elastic band resistance training in 65+ women (Gómez-Tomás et al., 2018). Exercises were conducted trice per week (Monday-Wednesday-Friday), in 3 sets with 10 repetitions and intensity progression from 3-7. The outcomes of the Fahlman et al. (2002) study demonstrate that high-loads strength training performed trice per week for 10 weeks produce an increase in HDL cholesterol (47.1 ± 3.3 mg/dl vs 57.4 ± 2.0mg/dl) and a decrease in triglycerides (113.5 ± 12.9 mg/dl vs 84.6 ± 12.9 mg/dl) and LDL levels (107.3 ± 11.2mg/dl vs 89.0 ± 11.2mg/dl) in elderly women cohort (73 ± 3 years, n = 15). Recently, Ihalainen et al. (2019) reported that long-term high-intensity (70-90% of 1RM) resistance training, conducted once, twice or thrice a week resulted in significant HDL-cholesterol increase in elderly women and men. Furthermore, low density lipoprotein (LDL) declined ($\Delta = -0.38 \pm 0.44$ mmol/L^{-1}, $P = 0.003$) in the group that had 3 resistance training sessions a week. Noteworthy, study showed that poor baseline level of metabolic syndrome is a determinant of significant training - induced improvements, regardless of the frequency. The influence of 8 weeks of traditional (TD) and pyramidal (PR) resistance training on muscle volume/muscle strength ratio and metabolic biomarkers was examined on 25 elderly women by Ribeiro et al. (2016). TD comprised of 3 constant-weight sets with 8–12 repetitions maximum (RM), while RT comprised of 3 sets of incremental weight with 12/10/8 RM, respectively. Each program was conducted 3 times per week. Both groups significantly improved ($P < 0.05$) glucose (TD = −4.5% vs. PR = −1.9%), triglycerides (TD = −18.0% vs. PR = −11.7%), high density lipoprotein (TD = +10.6 vs. PR = +7.8%), and low density lipoprotein (TD = −23.3% vs. PR = −21.0%), without significant group differences reported. Collectively, these data indicate that regular resistance training can significantly improve metabolic biomarkers in elderly women, which is in accordance with our study results. However, larger magnitude of change in most mentioned studies than we reported should be noted, with strength training program design and/or subjects characteristics likely responsible. Metabolic changes induced by resistance training are considered to be dependent on exercise program design, with load (volume and intensity) being regularly underlined (Garber et al., 2011). Except Ihalainen et al. (2019), all aforementioned studies used resistance training with medium or high intensity resistance training and with 3 times per week frequency. Indeed, Nunes et al. (2016) indicate superiority of high vs low-volume strength training (six sets/exercise vs three sets/exercise) in reducing the total cholesterol and LDL levels in postmenopausal women. In addition, it was reported that low intensity strength training (80% of 10 repetition maximum (10 RM)) failed to significantly modify blood lipid profile in apparently healthy, inactive postmenopausal women (Elliott et al., 2002). Finally, subjects characteristics may also influence the lipid-profile training-effects, as resistance training showed greater muscular adaptations in novice lifters, as in our study, when compared with an experienced one (Ribeiro et al., 2015). Aforementioned may explain why we found that low load resistance training significantly improved blood lipids and glucose level in elderly women. For all we know, our study is the first one showing that conducting 2 times per week chair based, low load (intensity- around 50% of 1RM) elastic band resistance training, has the potential to significantly improve metabolic biomarkers in older women. We consider this an important finding and substantial addition to the existing understanding of resistance training effects on health for this specific population.

The fact worth noting, beyond the scope of this paper is that mechanisms of the resistance training effect on metabolic biomarkers remains unclear. It can be speculated that lipid-lowering capacity of resistance training could lie in the increased ability of the skeletal muscle to utilize lipids vs. glycogen, consequently altering lipoprotein levels (Mann et al., 2014). Resistance training may have positive effect on lipoprotein fractions in older women via an increase in lipoprotein lipase which likely mediate LDL-C plasma removal and lipid oxidation (Mann et al., 2014). Finally, muscle-strengthening exercise was shown to increase density of glucose transporter GLUT-4 but also

quantity and kinetic properties of glycogen synthase (Phillips and Winett, 2010), consequently reducing blood glucose level.

Training protocol effects on functional fitness

Resistance training is the most widely recognized strategy to increase muscle strength and improve functional fitness in elderly cohorts (Csapo and Alegre, 2016; Brady and Straight, 2014). However, studies exploring the effect of EBT are scarce, with a heterogenous study design (exercise load and study duration), and consequently insufficient to draw firm conclusions. Our study results revealed a significant effect of EBT on upper body strength, lower body strength and general strength (handgrip strength), flexibility, agility and aerobic endurance in elderly women. Our study may be the first to show significant magnitude (small effect size) of elastic band resistance training effects on all functional fitness parameters included in SFBT in this specific population. Of these functional fitness attributes, flexibility is likely to be the least respected considering its health-related potential for elderly cohorts, though associated with disability and distress in elderly people (Shultz, 1992). Consequently, flexibility has been sparsely selected as measured outcome in studies exploring resistance training effects on functional fitness parameters (Barbosa et al., 2002; Fatouros et al., 2006; Carneiro et al., 2015), with all studies being unison regarding strength training -induced improvements in flexibility in elderly subjects. In addition, previous study suggested that resistance training effects on flexibility was both intensity-dependent (80% of 1RM superior to 60% of 1RM which is superior to 40% of 1RM) and frequency- dependent (3 times per week induces greater increases- 13,8% than 2 times per week-3%), with intensity above 60% of 1RM as a likely threshold for flexibility improvements. Contrary to this, we showed that low intensity resistance training (between 40-50% of 1RM) with low frequency (2 times per week) was potent enough to produce significant improvements in flexibility, with chair sit and reach improved by 540% and back scratch by 30% (Cohen's d = 0.315 and -0.291, respectively). These are larger increases than presented earlier (Fatouros et al., 2006; Carneiro et al., 2015), although it must be noted that different flexibility tests were used than in our study. The exact mechanisms for resistance training induced effects on flexibility are yet to be elucidated, but we can speculate that the decrease in musculotendinous and musculoarticular stiffness (Ochala et al., 2007) could at least be partially responsible. Clearly, mechanism studies about the topic are needed.

Systematic review by Martins et al. (2013) stated that duration of the studies using elastic band resistance vary from short-term 6 to long-term 24 weeks (average around 14), conducted 1 to 5 times per week (average = 3.2), 1-3 sets for 2-11 exercises done with 10-12 repetitions. In addition, the training intensity was variable and indirectly controlled choosing a predetermined number of repetitions related to a certain rating of subjects perceived exertion. Overall, they found elastic band resistance training as an effective method for improving muscle strength in elderly. In agreement with this, Liao et al. (2018) recently announced significant muscle mass, muscle quality and functional fitness improvements following 12 weeks elastic band resistance exercise with moderate load (volume-3 times/week with 3* 10 repetition ; intensity- 13 on the 0-20 RPE scale) in older women with sarcopenia. Functional fitness was assessed with 10ft up and go and 30-s chair stand tests and reported results indicate comparable increases to our study results (18% and 12%, respectively). Similarly, Oesen et al. (2015) showed that moderate load elastic band resistance training, conducted twice per week during a three months period, was able to enhance functional fitness, assessed by chair stand (11 ± 4 vs 14 ± 4 for pre vs post) and arm lifting test (24 ± 10 vs 28 ± 10 for pre vs post) but not handgrip strength, suggesting that functional fitness can be improved without concomitant increase in strength parameters in elderly (Raymond et al., 2013). Interestingly, this model of resistance training was not able to improve aerobic endurance (6 min walking test), which is contrary to our and several other study findings (Ades et al., 1996; Wieser and Haber, 2007). A significant association ($r = -0.632$, $p<0.001$) between the initial aerobic endurance and resistance training-induced change in aerobic endurance in both young and elderly subjects has been reported, suggesting that subject's initial aerobic endurance level is related to resistance training-induced increases in aerobic endurance (Ozaki et al., 2013). Although not reported, we can speculate that participants in Oesen et al. (2015) study in comparison to ours were aerobically fit, or at least with average aerobic endurance for elderly subjects (around 30 ml/kg/min) and consequently non-responders to resistance training-induced adaptation to aerobic endurance. The resistance training impact on cardiorespiratory fitness in elderly subjects was hypothesized to be attributable to increases in the capillary to fiber ratio and mitochondrial content and function (Ozaki et al., 2013). In addition, low load resistance training was found to produce robust increase in mitochondrial protein synthesis rate in resistance training experienced by elderly subjects (Burd et al., 2012). Recently, Bårdstu et al. (2020) reported significant improvements in stair climb (18%, $p = 0.03$) and maximal gait speed (8%, $p = 0.01$), but no improvements in 30-s chair stand, 8 foot up and go test and handgrip strength after 4 months of low volume (twice a week) and moderate intensity strength training including elastic bands in elderly subjects (80-90 years, 60% women). The lack of significant functional fitness changes with resistance training in comparison to our study could be due to lower exercise number (5 vs 10-12, respectively) and substantially lower attendance rate (51%), which seems insufficient training stimulus for adaptation to occur (Borde et al., 2015). Finally, Rieping et al. (2019) recently revealed that 14 week of EBT including 8-10 multiple joint exercises, executed with 2-3 sets and 15-20 repetitions significantly improves just 30-s chair stand test (6.67 ± 2.55 reps vs 8.47 ± 3.44 reps for pre vs post) out of four tests from SFTB (Rikli and Jones, 2013). It should be noted however, that all functional fitness parameters show improvement tendency, some with magnitude greater than in our study (8-Foot up-and-go test improved 25%), but did not reach statistical significance largely because of various methodological issues (big

variability of data and small sample size in particular). Collectively, evidences from our and other studies indicate elastic band training to be effective method for improving functional fitness in elderly women but also that there are magnitude effects- differences between studies. Intensity-related differences between studies could be at least partially responsible for heterogeneous results, as exercise intensity was not quantitatively described (ex: %1RM). In addition, differences in training volume and duration, and differences in the population profile (health and training history status) likely contribute to equivocal study results.

A few study limitations are noteworthy. First, the control group improved several functional fitness parameters which are likely to be attributed to the habitual physical activity- increment during intervention, though all were advised to preserve their daily habits (Walker et al., 2017). The need to objectively quantify the subject habitual physical activity level is therefore underlined. Second, only female patients were included, precluding generalizability of obtained data to elderly population. Thirdly, dietary intake was not controlled during the study period. Notwithstanding these limitations, the effectiveness of EBT on functional fitness and metabolic biomarkers in elderly women is clearly supported with present study findings.

Conclusion

The present resistance training protocol produced significant benefits on functional fitness and metabolic biomarkers in elderly women with very high adherence rate. In addition, EBT might be advised as an efficient strategy for the prevention and attenuation of age-associated metabolic markers and functional fitness alterations.

Acknowledgements

The article is dedicated to Z. Djindjic (1952–2003). The authors certify that there is no conflict of interest with any financial organization regarding the material discussed in the manuscript. Supported by Provincial Secretariat for Higher Education and Scientific Research (142-402-7/2017-1). The authors have no conflict of interest to declare. The datasets generated during and/or analyzed during the current study are not publicly available, but are available from the corresponding author who was an organizer of the study.

References

Aagaard, P., Suetta, C., Caserotti, P., Magnusson, S. P. and Kjær, M. (2010) Role of the nervous system in sarcopenia and muscle atrophy with aging: strength training as a countermeasure. *Scandinavian Journal of Medicine & Science in Sports* **20**, 49-64.

Ades, P. A., Ballor, D. L., Ashikaga, T., Utton, J. L. and Nair, K. S. (1996) Weight training improves walking endurance in healthy elderly persons. *Annals of Internal Medicine* **124**, 568-572.

Barbosa, A.R., Santarém, J.M., Filho, W.J. and Marucci, M. de F.N. (2002) Effects of resistance training on the sit-and-reach test in elderly women. *Journal of Strength and Conditioning Research* **16(1)**, 14-18.

Bårdstu H.B., Andersen V. and Fimland M.S. (2020) Effectiveness of a resistance training program on physical function, muscle strength, and body compositionin community-dwelling older adults receiving home care: a cluster- randomized controlled trial. *European Review Aging and Physical Acivityt* **17(1)**, 1-11.

Bergquist, R., Iversen, V. M., Mork, P. J. and Fimland, M. S. (2018) Muscle Activity in Upper-Body Single-Joint Resistance Exercises with Elastic Resistance Bands vs. Free Weights. *Journal of human kinetics* **61**, 5-13.

Bohannon, R. W. (2019) Grip strength: an indispensable biomarker for older adults. *Clinical Interventions in Aging* **14**, 1681-1691.

Borde, R., Hortobágyi, T. and Granacher, U. (2015) Dose-response relationships of resistance training in healthy old adults: a systematic review and meta-analysis. *Sports Medicine* **45**, 1693-1720.

Brady, A. O. and Straight, C. R. (2014) Muscle capacity and physical function in older women: What are the impacts of resistance training? *Journal of Sport and Health Science* **3**, 179-188.

Burd, N. A., Andrews, R. J., West, D. W., Little, J. P., Cochran, A. J. and Hector, A. J. (2012) Muscle time under tension during resistance exercise stimulates differential muscle protein sub-fractional synthetic responses in men. *Journal of Physiology* **590**, 351-362.

Calatayud, J., Borreani, S., Colado, J. C., Martin, F., Tella, V. and Andersen, L. L. (2015) Bench press and push-up at comparable levels of muscle activity results in similar strength gains. *Journal of Strength and Conditioning Research* **29(1)**, 246-253.

Carneiro, N.H., Ribeiro, A.S., Nascimento, M.A., Gobbo, L.A., Schoenfeld, B.J. and Achour, Júnior A., Gobbi, S. (2015) Effects of different resistance training frequencies on flexibility in older women. *Clinical Interventions in Aging* **10**, 531-538.

Cohen J. (1988) *Statistical power analysis for the behavioral sciences.* 2nd edition. Lawrence Earlbaum Associates, Hillsdale, New Jarsey

Colado J.C., Mena R., Calatayud J., Flández J. and Page P. (2020) Effects of strength training with variable elastic resistance across the lifespan: a systematic review. *Cultura, Ciencia y Deporte* **15**, 147-164.

Colado, J. C. and Triplett, N. T. (2008) Effects of a short-term resistance program using elastic bands versus weight machines for sedentary middle-aged women. *The Journal of Strength & Conditioning Research* **22**, 1441-1448.

Colado, J. C., Garcia-Masso, X., Triplett, N. T., Calatayud, J., Flandez, J., Behm, D. and Rogers, M. E. (2014) Construct and Concurrent Validation of a New Resistance Intensity Scale for Exercise with Thera-Band® Elastic Bands. *Journal of Sports Science and Medicine* **13**, 758-766.

Colado, J.C., Pedrosa, F. M., Juesas, A., Gargallo, P., Carrasco, J.J., Flandez, J., Chupel, M.U., Teixeira, A.M. and Naclerio, F.(2018) Concurrent validation of the OMNI-Resistance Exercise Scale of perceived exertion with elastic bands in the elderly. *Experimental Gerontology* **103**, 1-16.

Colado, J. C., Furtado, E.G., Teixeira, A.M., Flandez, J. and Naclerio, F. (2020) Concurrent and construct validation of a new scale for rating perceived exertion during elastic resistance training in the elderly. *Journal of Sports Science and Medicine* **19**, 175-186

Conceição, M. S., Bonganha, V., Vechin, F. C., de Barros Berton, R. P., Lixandrão, M. E., Nogueira, F. R. D., and Libardi, C. A. (2013) Sixteen weeks of resistance training can decrease the risk of metabolic syndrome in healthy postmenopausal women. *Clinical Interventions in Aging* **8**, 1221-1228.

Csapo, R. and Alegre, L. M. (2016) Effects of resistance training with moderate vs heavy loads on muscle mass and strength in the elderly: A meta-analysis. *Scandinavian Journal of Medicine & Science in Sports* **26**, 995-1006.

DeSure, A. R., Peterson, K., Gianan, F. V. and Pang, L. (2013) An exercise program to prevent falls in institutionalized elderly with cognitive deficits: a crossover pilot study. *Hawai'i Journal of Medicine & Public Health* **72**, 391-395.

Elliott, K. J., Sale, C. and Cable, N. T. (2002) Effects of resistance training and detraining on muscle strength and blood lipid profiles in postmenopausal women. *British Journal of Sports Medicine* **36**, 340-344.

Fahlman, M. M., Boardley, D., Lambert, C. P. and Flynn, M. G. (2002) Effects of endurance training and resistance training on plasma lipoprotein profiles in elderly women. *The Journals of Gerontology Series A: Biological Sciences and Medical Sciences* **57**, B54-B60.

Fahlman, M. M., McNevin, N., Boardley, D., Morgan, A. and Topp, R. (2011) Effects of resistance training on functional ability in elderly individuals. *American Journal of Health Promotion* **25**, 237-243.

Fatouros, I.G., A. Kambas, I. Katrabasas, D. Leontsini, A. Chatzinikolaou, A.Z. and Jamurtas, I. (2006) Resistance training and detraining effects on flexibility performance in the elderly are

intensity dependent. *Journal of Strength and Conditioning Research* **20(3)**, 634-642.

Fess, E.E.M.C. (1992) Grip strenght. In: *Clinical assessment recommendations.* Ed: Casanova J.S, 2 th edition. Chicago: American society of hand therapists. 41-45.

Flandez, J., Belando, N., Gargallo, P., Fernández-Garrido, J., Vargas-Foitzick, R.A., Devis-Devis, J. and Colado, J.C. (2017) Metabolic and functional profile of premenopausal women with metabolic syndrome after training with elastics as compared to free weights. *Biological Research for Nursing* **19(2)**, 190-197.

Friedewald, W.T., Levy, R.I. and Fredrickson, D.S. (1972) Estimation of the concentration of low-density lipoprotein cholesterol in plasma, without use of the preparative ultracentrifuge. *Clinical Chemistry* **18**, 499-502.

Fritz, N.B., Juesas, Á., Gargallo, P., Calatayud, J., Fernández-Garrido, J., Rogers, M.E. and Colado, J.C. (2018) Positive Effects of a Short-Term Intense Elastic Resistance Training Program on Body Composition and Physical Functioning in Overweight Older Women. *Biological Research for Nursing* **20(3)**, 321-334.

Furtado, G. E., Carvalho, H. M., Loureiro, M., Patrício, M., Uba-Chupel, M., Colado, J. C. and Teixeira, A. M. (2020) Chair-based exercise programs in institutionalized older women: Salivary steroid hormones, disabilities and frailty changes. *Experimental Gerontology* **130**, 1-11.

Furtado, G. E., Uba-Chupel, M., Carvalho, H. M., Souza, N. R., Ferreira, J. P. and Teixeira, A. M. (2016) Effects of a chair-yoga exercises on stress hormone levels, daily life activities, falls and physical fitness in institutionalized older adults. *Complementary Therapies in Clinical Practice* **24**, 123-129.

Garber, C. E., Blissmer, B., Deschenes, M. R., Franklin, B. A., Lamonte, M. J., Lee, I. M. and Swain, D. P. (2011) American College of Sports Medicine position stand. Quantity and quality of exercise for developing and maintaining cardiorespiratory, musculoskeletal, and neuromotor fitness in apparently healthy adults: guidance for prescribing exercise. *Medicine and Science in Sports and Exercise* **43**, 1334-1359.

Gargallo, P, Colado, J.C., Juesas, A., Hernando-Espinilla, A., Estañ-Capell, N., Monzó-Beltran, L., García-Pérez, P., Cauli, O. and Sáez, G.T. (2018) The effect of moderate- versus high-intensity resistance training on systemic redox state and DNA damage in healthy older women. *Biological Research for Nursing* **20(2)**, 205-217.

Gene-Morales, J., Gené-Sampedro, A., Salvador, R. and Colado, J.C. (2020) Adding the Load Just Above Sticking Point Using Elastic Bands Optimizes Squat Performance, Perceived Effort Rate, and Cardiovascular Responses. *Journal of Sports Science and Medicine* **19**, 735-744.

Gómez-Tomás, C., Chulvi-Medrano, I., Carrasco, J. J. and Alakhdar, Y. (2018) Effect of a 1-year elastic band resistance exercise program on cardiovascular risk profile in postmenopausal women. *Menopause* **25**, 1004-1010.

Ibáñez, J., Izquierdo, M., Martínez-Labari, C., Ortega, F., Grijalba, A., Forga, L. and Gorostiaga, E. M. (2010) Resistance training improves cardiovascular risk factors in obese women despite a significative decrease in serum adiponectin levels. *Obesity* **18**, 535-541.

Ihalainen, J. K., Inglis, A., Mäkinen, T., Newton, R. U., Kainulainen, H., Kyröläinen, H. and Walker, S. (2019) Strength training improves metabolic health markers in older individual regardless of training frequency. *Frontiers in Physiology* **10**, 32.

Jakobsen, M. D., Sundstrup, E., Andersen, C. H., Bandholm, T., Thorborg, K., Zebis, M. K. and Andersen, L. L. (2012) Muscle activity during knee-extension strengthening exercise performed with elastic tubing and isotonic resistance. *International Journal of Sports Physical Therapy* **7(6)**, 606-616.

Jakobsen, M. D., Sundstrup, E., Andersen, C. H., Persson, R., Zebis, M.K. and Andersen, L. L. (2014) Effectiveness of hamstring knee rehabilitation exercise performed in training machine vs. elastic resistance: Electromyography evaluation study. *American Journal of Physical Medicine & Rehabilitation* **93**, 320-327.

Lagally, K. M. and Robertson, R. J (2006) Construct validity of the OMNI resistance exercise scale. *Journal of Strength and Conditioning Research* **20**, 252-256.

Liao, C. D., Tsauo, J. Y., Huang, S. W., Ku, J. W., Hsiao, D. J. and Liou, T. H. (2018) Effects of elastic band exercise on lean mass and physical capacity in older women with sarcopenic obesity: A randomized controlled trial. *Scientific Reports* **8**, 1-13.

Liu, C. J. and Latham, N. (2010) Adverse events reported in progressive resistance strength training trials in older adults: 2 sides of a coin. *Archives of Physical Medicine and Rehabilitation* **91**, 1471-1473.

Liu, C. J. and Latham, N. K. (2009) Progressive resistance strength training for improving physical function in older adults. *Cochrane Database of Systematic Reviews* **3**, CD002759.

Manini, T. M. and Clark, B. C. (2012) Dynapenia and aging: an update. *Journals of Gerontology Series A: Biomedical Sciences and Medical Sciences* **67**, 28-40.

Mann, S., Beedie, C. and Jimenez, A. (2014) Differential effects of aerobic exercise, resistance training and combined exercise modalities on cholesterol and the lipid profile: review, synthesis and recommendations. *Sports Medicine* **44**, 211-221

Martins, W. R., de Oliveira, R. J., Carvalho, R. S., de Oliveira Damasceno, V., da Silva, V. Z. M. and Silva, M. S. (2013) Elastic resistance training to increase muscle strength in elderly: a systematic review with meta-analysis. *Archives of Gerontology and Geriatrics* **57**, 8-15.

Martins, W.R., Safons, M.P., Bottaro, M., Blasczyk, J.C., Diniz, L.R., Fonseca, R.M.C., Bonini-Rocha, A.C. and De Oliveira, R.J. (2015) Effects of short term elastic resistance training on muscle mass and strength in untrained older adults: A randomized clinical trial. *BMC Geriatrics* **15**, 99.

Melchiorri, G. and Rainoldi, A. (2011) Muscle fatigue induced by two different resistances: Elastic tubing versus weight machines. *Journal of Electromyography and Kinesiology* **21**, 954-959.

Nunes, P. R. P., Barcelos, L. C., Oliveira, A. A., Júnior, R. F., Martins, F. M., Orsatti, C. L. and Orsatti, F. L. (2016) Effect of resistance training on muscular strength and indicators of abdominal adiposity, metabolic risk, and inflammation in postmenopausal women: controlled and randomized clinical trial of efficacy of training volume. *Age* **38**, 40.

Ochala J., Lambertz D., Van Hoecke J. and Pousson M. (2007) Changes in muscle and joint elasticity following long-term strength training in old age. *European Journal of Applied Physiology* **100(5)**, 491-498.

Oesen, S., Halper, B., Hofmann, M., Jandrasits, W., Franzke, B., Strasser, E. M. and Wagner, K. H. (2015) Effects of elastic band resistance training and nutritional supplementation on physical performance of institutionalised elderly - A randomized controlled trial. *Experimental Gerontology* **72**, 99-108.

Orssatto, L. D. R., Moura, B. D., Bezerra, E. D. S., Andersen, L. L., Oliveira, S. D. and Diefenthaeler, F. (2018) Influence of strength training intensity on subsequent recovery in elderly. *Experimental Gerontology* **106**, 232-239.

Ozaki, H., Loenneke, J. P., Thiebaud, R. S. and Abe, T. (2013) Resistance training induced increase in VO 2 max in young and older subjects. *European Review of Aging and Physical Activity* **10**, 107-116.

Phillips, S. M. and Winett, R. A. (2010) Uncomplicated resistance training and health-related outcomes: evidence for a public health mandate. *Current sports medicine reports* **9**, 208-213.

Picorelli, A. M. A., Pereira, L. S. M., Pereira, D. S., Felício, D. and Sherrington, C. (2014) Adherence to exercise programs for older people is influenced by program characteristics and personal factors: a systematic review. *Journal of Physiotherapy* **60**, 151-156.

Raymond, M. J., Bramley-Tzerefos, R. E., Jeffs, K. J., Winter, A. and Holland, A. E. (2013) Systematic review of high-intensity progressive resistance strength training of the lower limb compared with other intensities of strength training in older adults. *Archives of Physical Medicine and Rehabilitation* **94**, 1458-1472.

Ribeiro, A. S., Aguiar, A. F., Schoenfeld, B. J., Nunes, J. P., Cavalcante, E. F., Cadore, E. L. and Cyrino, E. S. (2018) Effects of different resistance training systems on muscular strength and hypertrophy in resistance-trained older women. *The Journal of Strength & Conditioning Research* **32**, 545-553

Ribeiro, A. S., Schoenfeld, B. J., Souza, M. F., Tomeleri, C. M., Venturini, D., Barbosa, D. S. and Cyrino, E. S. (2016) Traditional and pyramidal resistance training systems improve muscle quality and metabolic biomarkers in older women: A randomized crossover study. *Experimental Gerontology* **79**, 8-15.

Ribeiro, A. S., Tomeleri, C. M., Souza, M. F., Pina, F. L. C., Schoenfeld, B. J., Nascimento, M. A. and Cyrino, E. S. (2015) Effect of resistance training on C-reactive protein, blood glucose and lipid

profile in older women with differing levels of RT experience. *Age* **37**, 109.

Rieping, T., Furtado, G. E., Letieri, R. V., Chupel, M. U., Colado, J. C., Hogervorst, E. and Ferreira, J. P. (2019) Effects of different chair-based exercises on salivary biomarkers and functional autonomy in institutionalized older women. *Research Quarterly for Exercise and Sport* **90**, 36-45.

Rikli, R. E. and Jones, C. J. (2013) Development and validation of criterion-referenced clinically relevant fitness standards for maintaining physical independence in later years. *The Gerontologist* **53(2)**, 255-267.

Robinson, K. R., Leighton, P., Logan, P., Gordon, A. L., Anthony, K., Harwood, R. H. and Masud, T. (2014) Developing the principles of chair based exercise for older people: a modified Delphi study. *BMC Geriatrics* **14**, 65.

Shultz, A.B. (1992) Mobility impairment in the elderly: Challenges for biomechanics research. *Journal of Biomechanics* **25,** 519–528.

Tan, Y. Y., Gast, G. C. M. and van der Schouw, Y. T. (2010) Gender differences in risk factors for coronary heart disease. *Maturitas* **65**, 149-160

Walker, S., Haff, G. G., Häkkinen, K. and Newton, R. U. (2017) Moderate-load muscular endurance strength training did not improve peak power or functional capacity in older men and women. *Frontiers in Physiology* **8**, 743.

Wieser, M. and Haber, P. (2007) The effects of systematic resistance training in the elderly. *International Journal of Sports Medicine* **28**, 59-65.

Wilson, J. and Kritz, M. (2014) Practical Guidelines and Considerations for the Use of Elastic Bands in Strength and Conditioning. *Strength and Conditioning Journal* **36(5)**, 1-9.

Zaslavsky, C. and Gus, I. (2002) The elderly. heart disease and comorbidities. *Arquivos Brasileiros de Cardiologia* **79**, 635-639.

Key points

- EBT lead to a significant improvement in glucose, total cholesterol, HDL and LDL in older women.
- EBT lead to a significant increase in cardiorespiratory fitness, handgrip strength, lower- body strength and flexibility, upper-body strength and flexibility and agility in older women.
- EBT seems to be viable model to ensure high training adherence rate.

✉ **Marko D.M. Stojanovic, PhD**
Faculty of Sport and Physical education, Lovcenska 16, Novi Sad, 21000, Serbia.

AUTHOR BIOGRAPHY

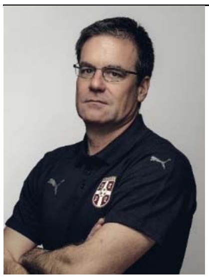

Marko DM. STOJANOVIC
Employment
Faculty of Sport and Physical Education, University of Novi Sad, Serbia.
Degree
PhD
Research interests
Physiological testing of the high-performance athlete, Strength training and conditioning, Growth, maturation and sport performance.
E-mail: marko.ns.stojanovic@gmail.com

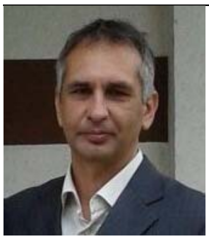

Mladen J . MIKIC
Employment
Faculty of Sport and Physical Education, University of Novi Sad, Serbia.
Degree
PhD
Research interests
Physiological testing of the high-performance athlete, Team sports performance analysis.
E-mail: mmmikac@gmail.com

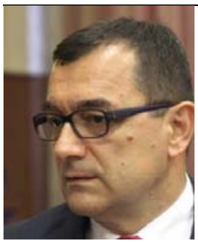

Zoran MILOSEVIC
Employment
Faculty of Sport and Physical Education, University of Novi Sad, Serbia.
Degree
PhD
Research interests
Motor Control, Kinesiology.
E-mail: zoranaisns29@gmail.com

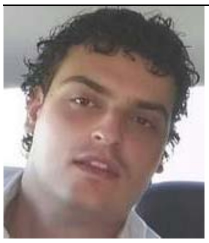

Jovan VUKOVIC
Employment
Faculty of Sport and Physical Education, University of Novi Sad, Serbia.
Degree
MSc
Research interests
Human performance, growth &maturation, testing& prescription.
E-mail: jovanvukovic89@gmail

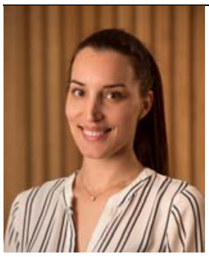

Tatjana JEZDIMIROVIC
Employment
RH Consulting, Serbia.
Degree
PhD
Research interests
Applied physiology, Clinical exercise physiology, Exercise and NCD in different ages.
E-mail: tatjanaj.ns@gmail.com

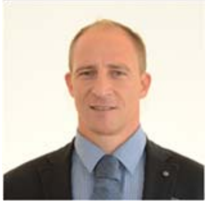

Vlatko VUCETIC
Employment
Faculty of Kinesiology, University of Zagreb, Croatia.
Degree
PhD
Research interests
Human performance, testing& prescription, strength & conditioning.
E-mail: vlatko.vucetic@kif.unizg.hr
