# Supplementary material for: Effects of elastic band resistance training on the physical and mental health of elderly individuals: A mixed methods systematic review
Source: PLoS One. 2024 May 13;19(5):e0303372. doi: 10.1371/journal.pone.0303372 (PMC11090353; doi:10.1371/journal.pone.0303372)
Supplement: S1 File — (ZIP) [file pone.0303372.s001.zip › Supporting Information/Included study 46.pdf]

©Journal of Sports Science and Medicine (2013) **12**, 422-430
http://www.jssm.org

Research article

Effects of Nordic Walking compared to Conventional Walking and Band-Based Resistance Exercise on Fitness in Older Adults

Nobuo Takeshima [1]✉, Mohammod M. Islam [2], Michael E. Rogers [3], Nicole L. Rogers [3], Naoko Sengoku [4], Daisuke Koizumi [1], Yukiko Kitabayashi [4], Aiko Imai [5] and Aiko Naruse [4]
[1] National Institute of Fitness and Sports in Kanoya, Kanoya, Japan; [2] Department of Rehabilitation, Yonaha General Hospital, Kuwana, Mie, Japan; [3] Wichita State University, Wichita, KS, USA; [4] Active Aging Association, Nagoya, Japan; [5] Nagoya City University, Nagoya, Japan

Abstract

The purpose of this study was to compare the effects of Nordic walking with conventional walking and band-based resistance exercise on functional fitness, static balance and dynamic balance in older adults. Volunteers (n = 65) were divided into four groups: Nordic walking (NW), conventional walking (CW), resistance (RES), and control. Each group performed activity 50-70 min·day^{-1} (warm-up 10-15 min, main exercise 30-40, and cool down 10-15 min), 3 days·week^{-1} (NW and CW) or 2 day·week^{-1} (RES) for 12 wks. Upper-body strength improved ($p < 0.05$) in the RES (22.3%) and the NW (11.6%) groups compared to the CW and control groups. Cardio-respiratory fitness improved more in the NW (10.9%) and CW (10.6%) groups compared to the RES and control groups. Upper- and lower-body flexibility also improved in all exercise groups compared to the control group. There were no improvements in balance measures in any group. While all modes of exercise improved various components of fitness, Nordic walking provided the best well-rounded benefits by improving upper-body strength, cardiovascular endurance, and flexibility. Therefore, Nordic walking is recommended as an effective and efficient mode of concurrent exercise to improve overall functional fitness in older adults.

Key words: Walking, resistance exercise, concurrent exercise, aging, functional fitness.

Introduction

Exercise plays important roles in maintaining and increasing levels of physical activity and function in older adults. Previous reports have shown the beneficial effects of exercise on cardio-respiratory fitness and muscular strength in older adults (American College of Sports Medicine, 2009; 2011; Takeshima et al., 2007; U.S. Surgeon General, 1996).Emphasis has also been placed on well-rounded exercise programs [WREP] that typically include four components (i.e., cardiovascular, flexibility, strength, and balance) of fitness training (ACSM, 1998).

Walking is popular among older individuals in Japan as well as in Western countries but it has been shown that walking alone will only improve cardiorespiratory fitness and has no effect on strength and several other parameters of fitness and function (Takeshima et al., 2007). On the other hand, resistance training will improve muscle strength but has no effect on cardiorespiratory fitness (Takeshima et al., 2007). Several studies have examined the effect of a combination of aerobic, resistance and flexibility exercises in healthy older adults (Takeshima et al., 2007) and older outpatients (Yamauchi et al., 2005). Supervised 12-wk training programs result in significant improvements in endurance, functional strength, flexibility, agility, serum lipids and blood pressure. These programs involved either concurrent or combined exercises. Concurrent training involves performing the activities in the same session (e.g., circuit training) while combined training involves performing a single type of exercise one day and a different type of exercise on another day. Traditionally, in order for older adults to improve each component of fitness, multiple modes or types of exercise have been performed. However, it is possible that a single type of exercise could provide improvements to each of these components.

Nordic walking is an activity involving specially designed poles used to push against the ground with each stride for the purpose of activating the upper body while walking. According to earlier studies, Nordic walking increases gait speed and cardiovascular metabolism (Church et al., 2002; Porcari et al., 1997). A more recent study found that when Nordic walking is performed at a slower pace than conventional walking without poles, it results in a higher heart rate and higher oxygen consumption (Schiffer et al., 2006). Nordic walking has also been used for rehabilitation in individuals with intermittent claudication (Oakley et al., 2008), Parkinson's disease (Van Eijikern et al., 2008), depression (Sujika et al., 2009), athletic injuries (Knobloch, et al., 2007) and chronic low back pain (Hartvisgsen et al., 2010). Moreover, in obese women, it elevated exercise intensity and adherence to the training program without increasing the perception of effort, leading to enhanced aerobic capacity (Figard-Fabre, et al., 2011). The results of studies conducted in the Scandinavian countries confirm that Nordic walking is more effective than walking without sticks in younger adults (Tschentscher et al., 2013). However, no one has yet performed a similar comparative study in older adults. Given the whole-body nature of the activity, Nordic walking using poles may be as, or more, beneficial to individual components of fitness compared to more common types of exercise such as conventional walking and resistance training for older adults.

The purpose of this study was to compare the effects of Nordic walking with conventional walking and

Received: 05 March 2013 / Accepted: 08 April 2013 / Available (online): 30 May 2013 / Published: 01 September 2013

elastic resistance band-based exercise on functional fitness, and static and dynamic balance in older adults. To our knowledge, no study has compared Nordic walking with conventional walking and /or resistance exercise in older adults.

Methods

Participants

A total of 65 community-dwelling seniors residing in Nagoya city volunteered to participate in the study. Participants were: (1) sedentary individuals for whom exercise was not contraindicated for any health reason; and (2) aged 60 years or older. They were assigned to one of four groups: a Nordic walking (NW) group, a conventional walking (CW) group or an elastic resistance band-based (RES) group, or control group. However, the group assignments were not completely randomized, as some of the participants were committed to attend exercise classes on assigned days (NW - Monday, Wednesday and Friday; CW - Tuesdays, Thursday and Saturday) and therefore some had to be transferred between the NW and CW groups. The RES group participated in university-based classes 2 days a week. The NW group had 17 members (average age: 70 ± 5 yr; 8 men and 9 women), the CW group 16 members (68 ± 5 yr; 8 men and 8 women), and the RE group 15 members (68 ± 5 yr; 5 men and 10 women), and the control group 17 members (70 ± 7 yr, 7 men and 10 women).

Preliminary screening was performed using a questionnaire covering medical history and physical activity. After the questionnaire was checked by a physician, seven participants underwent a physical examination and graded treadmill stress testing with EKG at a local hospital. These individuals were permitted by their physician to perform all of the exercises. The ethics committee of the Graduate School of Natural Sciences at Nagoya City University approved the study. All participants received written and oral instructions for the study and gave written informed consent prior to participation.

Interventions

Nordic walking: Participants met at a park near the university and were instructed to walk with poles continuously around the park as much possible during exercise classes. The NW group performed supervised exercise sessions three times a week for 12 weeks, 50-70 min per day (warm-up 10-15 min, main exercise 30 min in the first eight weeks and 40 min in the final four weeks, cool down 10-15 min). The participants walked on an asphalt course approximately 1400 meters in length. To ensure compliance with the exercise protocol, all sessions were supervised by research assistants who had been formally trained in Nordic walking technique. Participants were instructed to perform moderate- to high-intensity walking for 30 min in the second stage (week 5 to 8) and 40 min in the final stage (week 9 to 12) of the study. Exercise intensity was monitored based on heart rate (HR) and subjective ratings of perceived exertion (RPE) (Borg, 1982). During the first stage (week 1 to 4), participants mainly practiced walking with poles with the aim of improving their technique. HR was measured by an automatic monitor [PL600] (Cateye Co., Osaka, Japan) during exercise. All participants were instructed to achieve an exercise intensity corresponding to a HR of 100-120 bpm. All participants also wore an accelerometer [Kenz Lifecorder] (Suzuken Co. Nagoya, Japan) at waist-level to monitor the number of steps taken during each class.

Conventional walking: The CW group used the same course as the Nordic walking class. They performed supervised exercise sessions three times a week for 12 weeks for a total of 50-70 min each day (warm-up 10-15 min, main exercise 30 min in the first and second stages and 40 min in the final stage and cool down 10-15 min). Participants wore an accelerometer at waist-level to monitor the number and intensity of steps taken during exercise. When doing brisk walking, they were instructed to achieve an intensity corresponding to a HR of 100-120 bpm. Target intensities were the same for both the Nordic walking and conventional walking classes.

Resistance exercise: The RE group performed supervised exercises at a university fitness center, 50-60 min per day (warm-up 10-15 min, main exercise 30 min, cool down 10-15min), two times in a week for 12 weeks. The RE group performed elastic resistance band-based (Thera-Band®, Hygenic Corporation, Akron, OH, USA) exercises for all major muscle groups. Participants were instructed to progressively increase resistance every two to four weeks by advancing to the next color of band (in order from lower to higher resistance: yellow, red, green, blue, black, silver and gold). Specifically, they were instructed to change bands when able to perform 20 repetitions of a given motion. Exertion was rated using the 6-20 point Borg RPE scale (Borg, 1982). Participants were instructed to start resistance exercises at an intensity level of 11 to 13 and then to progressively increase to a level of 15 to 17. They were instructed to not hold their breath during exercises in order to prevent exercise-induced blood pressure elevations. A research assistant with experience leading group-based exercise served as the instructor and additional assistants supervised the performance of all exercises.

Nordic walking and conventional walking were performed three days per week in this study as this is the minimum frequency recommended by the American College of Sports Medicine (2011) for aerobic activity. Likewise, resistance training was performed two times per week as this is the minimum frequency recommended by the American College of Sports (2011) for this type of activity.

Measurement of functional fitness

A battery of field tests, specifically developed for older adults, was used to assess the components of functional fitness. These tests require very little equipment and are designed to be conducted in community settings. Using a standardized protocol, each test was conducted individually with the exception of the 12-minute walk that was conducted in small groups of 4-6 people. In this case, participants were instructed to set their own pace and to not walk with others. Participants completed all tests in no particular order (although the 12-min walk was always

performed last) in a single session with at least five minutes of rest between each test. These tests have been shown to have content and construct validity as well as good test-retest reliability (Duncan et al., 1990; Miotto et al., 1999; Rikli and Jones, 1999; Rogers et al., 2003). Following measurement of height and weight, body mass index (BMI) was computed as body weight (kg) divided by height (m) squared.

Upper-body strength was assessed using the 30-s Arm Curl Test [Arm Curl] (Rikli and Jones, 1999). On a signal, participants were instructed to flex and extend the elbow of the dominant hand, lifting a weight (men: 8-pound dumbbell, women: 5-pound dumbbell) through the complete range of motion, as many times as possible in 30 sec. After demonstration by the tester, a practice trial of one or two repetitions was given, followed by one 30-s test trial. The score was the number of repetitions completed within 30 seconds.

Lower-body strength was assessed using the 30-second Chair Stand Test [Chair Stand] (Rikli and Jones, 1999). On a signal, participants rose to a full standing position from a chair and then returned to a fully seated position, and continued to complete as many full stands as possible in 30 sec. After demonstration by the tester, a practice trial of one to three repetitions was given, followed by one 30-sec test trial. The score was the total number of stands executed correctly within 30 sec.

Balance and agility were assessed using the 8-foot Up and Go Test [up and go] (Rikli and Jones, 1999) and Functional Reach Test [functional reach] (Duncan et al., 1990). To perform up and go, participants were fully seated in a chair, hands on thighs and feet flat on the floor. On a signal, participants stood from the chair, walked as quickly as possible around a cone which was placed 8 feet (2.44 m) ahead of the chair, and returned to a fully seated position on the chair. Participants were told that this was a timed test and that the objective was to walk as quickly as possible (without running) around the cone and back to the chair. After demonstration by the tester, participants were given one practice trial and two test trials. The best performance time of the test trials was recorded in units of 0.1 sec. To perform the functional reach, the functional reach scale (graduated in centimeters) was hung from a wall at a height suitable for the participant. After demonstration by the tester, participants were given one practice trial and two test trials. The participant stood by the wall with feet together, hands clasped and both arms raised in front horizontally and held at the 0 centimeter level of the scale. On a signal, the participant leaned forward, moving the hands forward along the scale as far possible while keeping the heels in contact with the ground. Performance was assessed as the maximal distance the participant could reach forward beyond arms' length and without taking a step.

Upper-body flexibility was assessed using the Back Scratch Test [back scratch] (Rikli and Jones, 1999). Participants placed the preferred hand behind the same side shoulder with the forearm pronated and fingers extended. They placed the other hand behind the back, forearm supinated, reaching up in an attempt to touch or overlap the extended middle fingers of both hands. After demonstration by the tester, participants were asked to determine the preferred hand, and were given two practice trials, followed by two test trials. The score was the number of centimeters the middle fingers were short of touching (minus score) or overlapped each other (plus score). The best score of test trials was used to evaluate performance.

Lower-body flexibility was assessed using the Chair Sit and Reach Test [sit and reach] (Rikli and Jones, 1999). Participants sat on the edge of a chair with one leg bent and the other leg extended straight in front with the heel on the floor. Without bending the knee, participants slowly reached forward, sliding the hands down the extended leg in an attempt to touch the toes. After demonstration by the tester, participants were asked to determine the preferred leg and were given two practice trials on that leg, followed by two test trials. The score was the number of centimeters short of reaching the toes (minus score) or reached beyond the toes (plus score). The best score of two test trials was used to evaluate performance.

Cardio-respiratory fitness was assessed by performing the 12-minute Walk Test [12-min walk] which assessed the maximum distance walked in 12 minutes around a 60-meter rectangular course marked into 5-meter segments (Takeshima et al., 1992; Yamauchi et al., 2005). The score was the total number of meters walked in 12 minutes. To familiarize all participants with the 6-20 point Borg RPE scale (Borg, 1982), participants were instructed on its use and practiced using it during the pre-training 12-min walk trial.

After 12-weeks, all measurements were repeated in each participant. All tests were conducted by the same tester during pre and post testing.

Static and dynamic balance

To measure static and dynamic balance, a Balance Master Platform System [Balance Master 8.0.2] (NeuroCom International, USA) was used [22]. Static balance measures are often taken while standing on different surfaces (firm surface or form pad) with the eyes open or closed. In this study, the Clinical Test of Sensory Interaction for Balance was conducted to test static balance and measure the influence of sensory input on balance. For this test, postural sway was evaluated while the participant stood quietly with eyes open (EO) on a firm surface, eyes closed (EC) on a firm surface, EO on a foam surface and EC on a foam surface. An index of composite mean for the four conditions (SVcomp) was used as a static balance parameter. The force platform was marked to maintain consistency in foot placement. For each stance, the participant stood with their eyes at the horizon and their arms at the sides in a neutral position. An anthropometric kit was used to measure standing height, foot length, and foot width. This information was used later to express the results relative to the height and base of support of each participant. Trials were ten seconds in length and a trial was considered unsuccessful if the participant took a step or was unable to balance for the required time period without aid from a spotter.

Dynamic balance is the ability to anticipate changes and coordinate muscle activity in response to

perturbations of stability (Rogers et al., 2003). Dynamic balance is also used during forward, sideways, and backward learning. In this study, dynamic balance was evaluated by performing the limits of stability (LOS) test on the same balance platform. The LOS is an assessment tool utilized to quantify the maximum distance an individual can intentionally displace their center of gravity, that is, lean in a specified direction without losing balance, stepping, or reaching for assistance (Newton, et al., 2001). Each participant stood on the platform viewing a monitor located to the front on which a cursor representing his or her center of pressure (COP) was viewed. The screen also showed nine boxes, one representing the centered COP position, and eight others representing targets spaced at 45-degree intervals in an oval shape representing 100% of the distance from the center position to the theoretical LOS. Each participant's age and height were used to calculate the theoretical LOS. Each participant was instructed to stand as still as possible while maintaining the COP cursor within the highlighted center target. When a black circle appeared in a designated outer LOS target, the participant was instructed to move as quickly and accurately as possible so as to move the COP cursor to the designated target on a straight line, hold that position until termination of the trial (10 seconds), and then return the cursor to the center target (Rogers et al., 2003).

The endpoint excursion (EPE) is the distance the COP is displaced toward the target during the participant's primary movement. This movement segment ends when the COP movement first ceases progression toward the target (Rogers et al., 2003). Endpoint excursion is expressed as a percentage of the total distance to the target. Hence, a subject whose initial movement ends precisely at the target has an endpoint excursion of 100%. When initial attempts are substantially short of the target, most people initiate additional movements after the endpoint excursion is recorded. To represent this additional movement and COP excursion, an additional measurement, the maximum excursion (MXE), is used. The MXE is the maximum distance the COP is displaced toward the target over the entire duration of the trial (Rogers et al., 2003). MXE is also expressed as a percentage of the distance to the target. Since subjects make additional movement after the primary movement attempt described above, the MXE is usually somewhat larger than the EPE. In this study, indexes of composite mean of endpoint excursion (EPEcomp), and maximum excursion (MXEcomp) components, in the eight directions of LOS were used for analysis.

Before the present study was conducted, the test-retest reliability of balance (n= 13) was confirmed in a separate, but similar, population. Intra-class correlation coefficients [ICCs] in the Clinical Test of Sensory Interaction revealed a high level of reproducibility (ICC range= 0.88-0.90). The ICC values for Limits of Stability Test were 0.90-0.96.

Data analysis

Data analysis was completed using the statistical software program SPSS for MAC (V.19.0, SPSS Inc., Chicago, IL). Absolute values were used for statistical analysis. However, when discussing differences between interventions on functional fitness measures, relative change was used to provide a clearer translation for the purpose of comparisons between measures with different units. Data were screened for outliers, and assumptions of normality and homoscedasticity. To reduce the potential influence of outliers on the statistical analysis, box-and-whiskers plots were used to identify outliers, which were subsequently eliminated prior to analysis. Each parameter was examined for normality using the Kolomogorov-Smirnov test. Assumptions of homogeneity of variance and sphericity were evaluated. Baseline group mean comparisons were performed using two-tailed independent t-tests. Paired-sample t-tests and chi-square test were used to initially compare individual interventions. Comparisons between groups over time were evaluated using a two-way multivariate ANOVA with repeated measures. (ANOVA, Wilk's criterion). Group (NW, CW RE, and control) served as the between-subject factor, while Time (pre- and post-test) served as the within-subject factor. Univariate analyses were conducted when significant multivariate main effects were observed with follow-up Bonferroni post-hoc testing to determine group differences among parameters. Percent changes from pre to post were calculated from the differences in scores. Effect size [ES] was also calculated for each test. Cohen's definition of small, medium and large ESs (ES = 0.2, 0.5, and 0.8, respectively) was used (Cohen, 1988). A probability value of less than 0.05 was considered statistically significant.

Results

Pre- training data

No significant differences were noted at baseline for age, height, weight, BMI, or the sex ratio between men and women among the three groups (Table 1). No significant baseline difference was noted in any measured functional fitness and balance parameters among the groups (Table 1).

Table 1. **General characteristics of participants at baseline. Values are means (±SD).**

	Nordic walking (NW)	Conventional walking (CW)	Band-based resistance (REC)
**Number**	17	16	15
**Sex (M/F)**	8/9	8/8	5/10
**Age (yr)**	70.1 (5.3)	68.0 (4.9)	68.0 (5.4)
**Height (m)**	1.59 (.08)	1.59 (.08)	1.56 (.07)
**Body mass (kg)**	58.1 (12.9)	60.8 (9.5)	57.7 (6.9)
**BMI**	23.0 (3.9)	24.2 (3.3)	23.6 (2.0)

BMI, body mass index (mass/height^2); No test parameter was significantly different between the groups.

Table 2. Training data for Nordic walking and conventional walking. Values are means (±SD).

	Group	First stage (1-4 wk)	Second stage (5-8 wk)	Final stage (9-12 wk)
**Walk distance (m)**	**NW**	--------	2766 (200)	3829 (351
	**CW**	--------	2777 (52)	4033 (344)
**Duration (min)**	**NW**	--------	30 (1)	39 (1)
	**CW**	--------	30 (3)	40 (3)
**Mean walk velocity (m·min^{-1})**	**NW**	--------	93 (8)	97 (7)
	**CW**	--------	94 (10)	100 (9)
**RPE**	**NW**	--------	12.6 (.6)	12.8 (.5)
	**CW**	--------	13.1 (1.5)	14.1 (1.0) *
**HR (bpm)**	**NW**	--------	117 (10)	117 (10)
	**CW**	--------	117 (15)	123 (11)
**%HRmax (%)**	**NW**	--------	59.3 (12.1)	58.9 (14.5)
	**CW**	--------	52.1 (21.8)	60.8 (14.6)

NW, Nordic walking group; CW, conventional walking group; RPE, ratings of perceived exertion; HR, heart rate; %HRmax, percent of max heart rate (220-age); ----, no record; * $p < 0.05$ between groups.

Training data

All participants continued the Nordic walking, conventional walking and band-based resistance exercise with no incidence of injury during the study. Mean attendance rates for the NW, CW and RES groups were 80.2%, 78.2%, and 90.2%, respectively. Exercise intensity was not monitored in the first stage (1-4 weeks) when participants primarily practiced performing the exercises methodically and correctly in the NW and CW groups. During the final two stages (5-12 weeks) both groups trained at a low to moderate intensity as indicated by intensity monitoring parameters (Table 2). The mean walking velocity of participants during the second stage (week 5-8) was 93 m·min^{-1} in the NW group and 94 m·min^{-1} in CW group. Higher exercise intensity was noted in the final stage, but no significant difference was found in walk distance, duration, mean velocity, or heart rate (HR) between the two groups (Table 2). RPE, however, was significantly lower in the NW than CW group (Table 2). While intensity was not recorded for the RES group, they were instructed to gradually increase the intensity from 11-13 to 15-17 on the RPE scale. All participants in the RES group did increase the intensity of exercise as denoted by the color of the band used from stage to stage.

Post-training functional fitness data

After 12 wk of training, there were significant interactions (group × time) for arm curl, chair stand, 12-min walk, functional reach, up & go, back scratch, and sit & reach (Table 3). Based on post hoc testing, the NW and CW groups (10.9% and 10.6%, respectively) had similar improvements in the 12-min walk and both exhibited greater improvements than the RES group (3.2%). The RES group (23.3%) showed significantly greater improvements in arm curl than the NW and CW groups (11.6% and 5.1%, respectively) but the NW group had greater improvements in arm curl compared to the CW group. For lower body strength, RES (21.1%) improved more than CW (9.5%) in the chair stand and improvements for RES and NW (12.6%) did not differ.

Upper (back scratch) and lower (sit & reach) body flexibility improved in all exercise groups compared to the control group. In addition, the NW group (75.3%) improved relative to the CW group (61.3%).

On measures of agility and balance, few significant differences were found between groups. Values obtained using the balance platform (SVcomp, EPEcomp, and MXEcomp) did not differ between exercise groups or from the control group. Functional reach and up & go did improve in the RES group relative to the control group, but there were no changes in the NW or CW groups.

Discussion

The key finding of the current study is that all groups showed improvements in overall functional fitness over 12 weeks but Nordic Walking appears to provide the greatest benefit to overall fitness as indicated by cardio-respiratory fitness, strength and flexibility outcomes. The improvement in upper-body strength was significantly greater in the RES group than NW and CW groups. A significantly different, upper-body strength ($F = 4.472$, $p = 0.045$) to improve more in the NW group (12.9%) than AE (5.6%) group. Conversely, although cardio-respiratory fitness improved significantly in the two walking groups compared to the RES group, the increase did not differ between the NW and AE groups (10.9% and 10.7%, respectively) indicating that similar improvement is achieved with Nordic walking and conventional walking, yet Nordic walking was performed at a lower intensity.

Given the specific nature of adaptations to exercise and the need for maintaining muscle mass, muscular strength, and flexibility throughout life, a well-rounded training program consisting of resistance, aerobic and flexibility exercises is highly recommended (ACSM, 1998). Nordic walking can improve muscular strength and flexibility as well as aerobic endurance capacity. A combination of aerobic exercise and resistance training might have the same effect as Nordic walking. However, Nordic walking takes less time than performing the same amount of conventional walking along with additional resistance training sessions so the use of poles during walking is beneficial.

Kocur et al. (2009) described the effects of Nordic walking on functional fitness in cardiac patients. Similar to the current study, they found improvements in muscle strength for Nordic walking but not conventional walking. These studies demonstrate that greater strength benefits can be derived from Nordic walking compared to conventional walking. However, when comparing strength

Table 3. Effects of exercise mode on functional fitness in older adults.

	Pre Mean (±SD)	Post Mean (±SD)	Change (%)	Within-group difference in change scores from baseline Mean (95%CI)	ES	P< (group×time)	Group Differences
**Arm curl (times/30sec)**							
NW (n=17)	24.9 (4.7)	27.8 (4.4)	11.6	2.94 (1.7 to 4.2)	.62		CW, RES, CON
CW (n=16)	25.3 (3.5)	26.6 (3.2)	5.1	1.25 (0.2 to 2.3)	.37	<.001	NW, RES
RES (n=15)	25.7 (5.2)	31.7 (4.1)	23.3	6.07 (3.9 to 8.2)	1.15		NW, CW, CON
CON (n=17)	24.7 (4.3)	25.2 (3.5)	2.0	.59 (-.9 to 2.1)	.18		NW, RES
**Chair stand (times/30sec)**							
NW (n=17)	24.7 (6.0)	27.8 (5.4)	12.6	3.06 (1.7 to 4.4)	.52		
CW (n=16)	24.3 (4.0)	26.6 (3.9)	9.5	2.38 (1.1 to 3.7)	.58	<.05	RES
RES (n=15)	24.2 (5.4)	29.3 (4.7)	21.1	5.13 (2.8 to 7.5)	.95		CW, CON
CON (n=17)	25.7 (6.0)	27.3 (5.1)	6.2	1.65 (.1 to 3.2)	.26		RES
**12-min walk (m)**							
NW (n=17)	1142 (77)	1266 (89)	10.9	123.47 (92.5 to 154.4)	1.61		RES, CON
CW (n=16)	1187 (97)	1313 (112)	10.6	125.63 (97.4 to 153.9)	1.30	<.001	RES, CON
RES (n=15)	1143 (108)	1180 (133)	3.2	37.47 (4.7 to 70.2)	.37		NW, CW
CON (n=17)	1099 (86))	1128 (77)	2.6	28.65 (7.6 to 49.8)	.32		NW, CW
**Functional reach (cm)**							
NW (n=17)	28.7 (4.9)	30.5 (2.8)	6.3	1.76 (-.5 to 4.1)	.37		
CW (n=16)	29.0 (3.9)	31.8 (5.5)	9.7	2.75 (-.1 to 5.6)	.72	<.05	
RES (n=15)	32.0 (6.3)	35.4 (5.4)	10.6	3.40 (.1 to 6.7)	.54		CON
CON (n=17)	32.2 (3.5)	31.3 (3.9)	-2.7	-.94 (-2.5 to .6)	.32		RES
**Up & go (sec)**							
NW (n=17)	4.3 (.3)	4.1 (.4)	4.7	-.21 (-.4 to .0)	.67		
CW (n=16)	4.5 (.6)	4.2 (.5)	6.7	-.24 (-.5 to .0)	.50	<.05	
RES (n=15)	4.5 (.5)	4.2 (.5)	6.7	-.33 (-.6 to -.1)	.60		CON
CON (n=17)	4.5 (.5)	4.5 (.4)	.0	.00 (-.2 to .2)	.04		RES
**Back scratch (cm)**							
NW (n=17)	-9.2 (16.1)	-5.1 (13.6)	44.5	4.09 (1.1 to 7.0)	.26		CON
CW (n=16)	-8.0 (14.2)	-6.0 (13.7)	25.0	1.97 (0.4 to 3.5)	.14	<.05	CON
RES (n=15)	-3.2 (9.5)	-.1 (9.4)	93.8	3.01 (0.2 to 5.8)	.33		CON
CON (n=17)	-5.7 (14.3)	-7.1 (14.8)	-24.6	-1.32 (-2.5 to -0.2)	.10		NW, CW, RES
**Sit & reach (cm)**							
NW (n=17)	6.9 (13.2)	12.1 (11.5)	75.3	5.18 (2.5 to 7.8)	.39		CW, CON
CW (n=16)	3.1 (18.3)	5.0 (17.3)	61.3	1.91 (0.5 to 3.3)	.10	<.01	NW, CON
RES (n=15)	8.2 (12.2)	11.5 (12.4)	40.2	3.32 (0.7 to 5.9)	.27		CON
CON (n=17)	10.5 (11.7)	9.2 (11.8)	-12.3	-1.29 (-3.7 to 1.1)	.15		NW, CW, RES
**SVcomp (deg/sec)**							
NW (n=17)	1.02 (.25)	.87 (.17)	14.7	-0.15 (-0.2 to -0.1)	.60		
CW (n=16)	1.01 (.27)	.91 (.23)	9.9	-0.09 (-0.2 to 0.0)	.37	N.S.	
RES (n=15)	1.03 (.23)	.98 (.24)	4.9	-0.05 (-0.2 to 0.1)	.22		
CON (n=11)	.79 (.16)	.71 (.10)	-10.1	-0.08 (-0.2 to 0.0)	.50		
**EPEcomp (%)**							
NW (n=17)	77. 6 (12.2)	78.6 (10.6)	1.3	1.00 (-3.9 to 5.9)	.08		
CW (n=16)	75.3 (12.2)	73.6 (10.0)	2.3	-1.62 (-5.0 to 1.8)	.14	N.S.	
RES (n=15)	69.9 (14.7)	75.1 (10.0)	7.4	5.27 (0.3 to 10.3)	.35		
CON (n=11)	78.6 (10.4)	81.6 (10.6)	3.8	2.91 (-1.9 to 7.7)	.29		
**MXEcomp (%)**							
NW (n=17)	91.7 (9.8)	92.4 (7.2)	0.8	0.71 (-3.4 to 4.8)	.07		
CW (n=16)	90.7 (6.3)	90.8 (7.7)	0.1	0.06 (-2.6 to 2.7)	.02	N.S.	
RES (n=15)	85.3 (10.3)	88.9 (8.6)	4.2	3.40 (0.4 to 6.4)	.35		
CON (n=11)	92.2 (4.9)	97.1 (5.0)	5.3	4.91 (2.6 to 7.2)	.18		

NW, Nordic walking group; CW, conventional walking group; RES, band-based resistance exercise; CON, control group; SVcomp, composite mean of four conditions for the Clinical Test of Sensory Interaction; EPEcomp, composite mean of endpoint excursion for limits of stability (LOS); MXEcomp, composite mean of maximum excursion for limits of stability (LOS); 95%CI, 95% confidence intervals); ES, effect size; N.S., not significant.

outcomes of Nordic walking to resistance training, the improvements in arm curl and chair stand observed for Nordic walking in the current study were smaller than those observed with resistance training alone. Therefore, although Nordic walking provides benefits to muscular strength, if one is to maximize improvements in muscular strength then resistance training should be performed.

A number of studies have showed the effect of exercise on parameters of dynamic balance in older adults (Islam et al., 2004). Functional reach and up & go improved in the RES group, similar to what we previously found with a similar resistance program (Takeshima et al., 2007). Neither static balance (sway velocity) nor dynamic balance (end point excursion (EPE) and maximum end point excursion (MXE)) changed as a result of any exer-

cise program in the current study. Judge et al. (1993) reported that a vigorous program of lower-body strengthening, walking, and postural control exercise improved balance in older subjects. As the initial level of dynamic balance was relatively high in all groups (EPEcomp: 70%-78%, MXEcomp: 85-92%), the potential for improving dynamic balance in this study's participants was minimal and could account for the lack of improvement.

The current exercise course was on a flat asphalt-covered surface. Schiffer et al. (2011) have suggested that the impulse generated by the poles is the same on different surfaces. Given that there are differences in oxygen uptake between walking on grass and concrete, the main regulator for the propulsion must be the musculature of the lower extremities and the work of the upper extremities seems to be an additional effort for Nordic walkers. It is possible that performing Nordic walking on uneven and/or soft surfaces may induce improvements in balance and, therefore, further study is needed to determine the effects of Nordic walking on different surfaces.

The results of this study support Nordic walking as a safe, feasible, and beneficial form of exercise training that can improve multiple components of fitness in older adults. Conventional walking has a myriad of documented benefits associated with it, and is often used as a primary and secondary preventative measure (American College of Sports Medicine, 1998). Like conventional walking, Nordic walking can be performed by individuals or in group settings, as in the current study, which may provide benefits to social well-being. In addition, although often performed outdoors, Nordic walking can also be performed indoors with the use of rubber pole tips, making it an activity that can be performed year-round, even during inclement weather. Nordic walking appears to be as effective at improving cardiorespiratory fitness as conventional walking and provides additional benefits in upper-body strength and lower-body flexibility over conventional walking. Thus, Nordic walking may prove to be more effective than conventional walking in the improvement and maintenance of overall health and function. Further research is needed to compare these forms of exercise over longer periods of time and in different segments of the older adult population. Means by which strength gains can be enhanced with Nordic walking, possibly with the use of heavier poles, also needs to be addressed in future studies.

Conclusion

The results of this study indicate that both Nordic walking and conventional walking are beneficial for older adults. However, Nordic walking provides additional benefits in muscular strength compared to conventional walking, making it suitable for improving aerobic capacity and muscular strength as well as other components of functional fitness in a short period of time.

Acknowledgements

The authors acknowledge the participants for their voluntary involvement in this study. This study was supported by The Descente and Ishimoto Memorial Foundation for the Promotion of Sports Science (2010).

References

American College of Sports Medicine. (1998) The recommended quantity and quality of exercise for developing and maintaining cardiorespiratory and muscular fitness, and flexibility in healthy adults. *Medicine and Science in Sports and Exercise* **30,** 975-991.

American College of Sports Medicine. (2009) Exercise and physical activity for older adults- Position stand. *Medicine and Science in Sports and Exercise* **41,** 1510-1530.

American College of Sports Medicine. (2011) Quantity and quality of exercise for developing and maintaining cardiorespiratory, musculoskeletal, and neuromotor fitness in apparently healthy adults: Guidance for prescribing exercise. *Medicine and Science in Sports and Exercise* **43,** 1334-1359.

Borg, G. (1982) Psychophysical bases of perceived exertion. *Medicine and Science in Sports and Exercise* **14,** 377-381.

Church, S.C, Earnest, C.P and Morss, G.M. (2002) Field testing of physiological responses associated with Nordic Walking. *Research Quarterly for Exercise and Sport* **73,** 296-300.

Cohen J. (1988) *Statistical Power Analysis for the Behavioral Sciences* 2nd edition. Hillsdale, New Jersey: Lawrence Erlbaum

Duncan, P.W., Weiner, D. K., Chandler, J. and Studenski, S. (1990) Functional reach: a new clinical measure of balance. *The Journals of Gerontology. Series A, Biological Sciences and Medical Sciences* **45,** M192-M197.

Figard-Fabre, H., Fabre, N., Leonardi, A. and Schena, F. (2011) Efficacy of Nordic walking in obesity management. *International Journal of Sports Medicine* **32,** 407-417.

Hartvisgsen, J., Morso, L., Bendix, T. and Manniche, C. (2010) Supervised and non-supervised Nordic walking in the treatment of chronic low back pain: a single blind randomized clinical trial. *BMC Musculoskeletal Disorders [electronic resource]* 11. Avaliable form URL: http://www.biomedcentral.com/1471-2474/11/30

Islam, M.M., Nasu, E., Rogers, M.E., Koizumi, D., Rogers, N.L. and Takeshima, N. (2004) Effects of combined sensory and muscular training on balance in older adults. *Preventive Medicine* **39,** 1148-1155.

Judge, J. O., Lindsey, C., Underwood, M. and Winsemius, D. (1993) Balance improvements in older women: effects of exercise training. Physical Therapy **73,** 254-265.

Knobloch, K., Schreibmueller, L., Jagozinski, M., Zeichen, J. and Kretek, C. (2007) Rapid rehabilitation programme following sacral stress fracture in a long-distance running female athlete. *Archives of Orthopaedic and Trauma Surgery* **127,** 809-813.

Kocur, P., Deskur-Smielecka, E., Wilk, M., Dylewicz, P. (2009) Effects of Nordic Walking training on exercise capacity and fitness in men participating in early, short-term inpatient cardiac rehabilitation after an acute coronary syndrome- a controlled trial. *Clinical Rehabilitation* **23,** 995-1004.

Miotto, J.M., Chodzko-Zajko, W.J., Reich, J.L. and Supler, M.M. (1999) Reliability and validity of the Fullerton Functional Fitness Test: an independent replication study. *Journal of Aging and Physical Activity* **7,** 339-353.

Newton R. (2001) Validity of the multi-directional reach test: a practical measure for limits of stability in older adults. *The Journals of Gerontology. Series A, Biological Sciences and Medical Sciences* **56,** M248-M252.

Oakley, C., Zwierska, I., Tew, G., Beard, J. D. and Saxton, J. M. (2008) Nordic poles immediately improve walking distance in patients with intermittent claudication. *European Journal of Vascular and Endovascular Surgery* **36,** 689-694.

Porcari, J. P., Hendrikson, T. L., Walter, P. R., Terry, L. and Walsko, G. (1997) The physiological responses to walking with and without Power Poles on treadmill exercise. *Research Quarterly for Exercise and Sport* **68,** 161-166.

Rikli, R.E. and Jones, C.J. (1999) Development and validation of a functional fitness test for community-residing older adults. *Journal of Aging and Physical Activity* **7,** 129-161.

Rogers, M.E, Rogers, N.L., Takeshima, N. and Islam, M.M. (2003) Methods to evaluate and improve the physical parameters associate with fall risk in older adults. *Preventive Medicine* **36,** 255-264.

Schiffer, T., Knicker, A. and Struder, H. K. (2009) Energy cost and pole forces during Nordic walking under different surface condi-

tions. *Medicine and Science in Sports and Exercise* **41,** 663-668.
Schiffer, T., Knicker, A., Hoffman, U., Harwig, B., Hollmann, W. and Struder, H.K. (2006) Physiological responses to Nordic walking, walking and jogging. *European Journal of Applied Physiology* **98,** 56-61.
Schiffer, T., Kniker, A., Montanarella, M. and Struder, H. K. (2011) Mechanical and physiological effect of varying pole weights during Nordic walking compared to walking. *European Journal of Applied Physiology* **111,** 1121-1126.
Sujika, K., Pechter, U., Kaida, R., Tahepold, H., Maaroos, J. and Maaroos, H.I. (2009) Physical Activity of depressed patients and their motivation to exercise: Nordic walking in family practice. *International Journal of Rehabilitation Research* **32,** 132-138.
Takeshima, N., Rogers, M.E., Islam, M.M., Yamauchi, T., Watanabe, E. and Okada, A. (2004) Effect of concurrent aerobic and resistance circuit exercise training on fitness in older adults. *European Journal of Applied Physiology* **93,**173-182.
Takeshima, N., Rogers, N.L., Rogers, M.E., Islam, M.M., Koizumi, D. and Lee, S. (2007) Functional fitness gain varies in older adults depending on exercise mode. *Medicine and Science in Sports and Exercise* **39,** 2036-2043.
Takeshima, N., Tanaka, K., Kobayashi, F., Sumi, K., Watanabe, T. and Kato, T. (1992) Validity of the maximal aerobic capacity estimated from submaximal cycling exercise and field performance tests in the elderly. *Japanese Journal of Physical Fitness and Sports Medicine* **41,** 295-303 (In Japanese).
Tschentscher, M., Niederseer, D., and Niebauer, J. (2013). Health benefits of Nordic walking: a systematic review. *American Journal of Preventive Medicine* **44,** 76-84.
U.S. Department of Health and Human Services. (1996) *Physical Activity and Health: A Report of the Surgeon General.* Atlanta, GA: Center for Disease Control and Prevention. National Center for Chronic Disease Prevention and Health Promotion, S/N 017-023-00196-5.
Van Eijikern, F.J., Reijmers, R.S., Kleinveld, M.J., Minten, A., Bruggen, J.P. and Bloem, B.R. (2008) Nordic walking improves mobility in Parkinson's disease. *Movement Disorders* **23,** 2239-2243.
Yamauchi, T., Islam, M.M., Koizumi, D., Rogers, M.M., Rogers, N.L. and Takeshima, N. (2005) Effect of home-based well-rounded exercise in older adults. *Journal of Sports Science and Medicine* **4,** 563-571.

Key points

- Nordic walking, conventional walking, and resistance training are beneficial for older adults.
- Nordic walking and conventional walking both improve cardio-respiratory fitness while resistance training does not.
- Nordic walking provides additional benefits in upper-body muscular strength compared to conventional walking.
- Nordic walking is an effective and efficient mode of exercise to improve overall fitness in older adults.

AUTHORS BIOGRAPHY

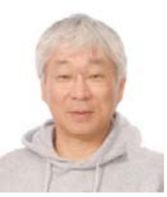

Nobuo TAKESHIMA
Employment
Laboratory of Exercise Gerontology, National Institute of Fitness and Sports in Kanoya, Kanoya, Kagoshima, Japan
Degree
PhD
Research interests
Well-rounded exercise program for older adults
E-mail: takeshima@nifs-k.ac.jp

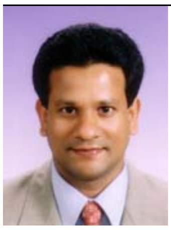

Mohammod M. ISLAM
Employment
Department of Rehabilitation, Yonaha General Hospital, Kuwana, Mie, Japan
Degree
PhD
Research interests
Fall prevention in older adults
E-mail: parumon@hotmail.com

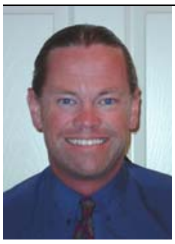

Michael E. ROGERS
Employment
Department of Human Performance Studies, Center for Physical Activity and Aging, Wichita State University, Wichita, USA
Degree
PhD
Research interests
Strength and balance training programs for older adults
E-mail: michael.rogers@wichita.edu

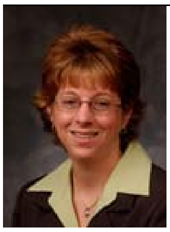

Nicole L. ROGERS
Employment
Department of Public Health Sciences, Wichita State University, Wichita, USA
Degree
PhD
Research interests
Health promotion for older adults
E-mail: nicole.rogers@wichita.edu

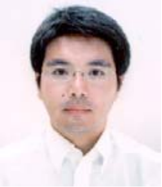

Daisuke KOIZUMI
Employment
Laboratory of Exercise Gerontology, National Institute of Fitness and Sports in Kanoya, Kanoya, Kagoshima, Japan
Degree
PhD
Research interests
Physical activity assessment for older adults
E-mail: dice.k.koizumi@gmail.com

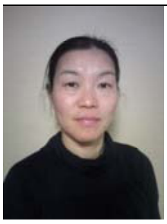

Naoko SENGOKU
Employment
Active Aging Association, Nagoya, JAPAN.
Degree
MSc
Research interests
Effect of Nordic walking
E-mail: naokosen@gmail.com

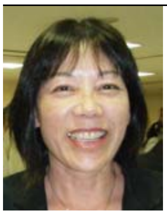

Yukiko KITABAYASHI
Employment
Active Aging Association, Nagoya, JAPAN.
Degree
MSc
Research interests
Health promotion of older adults
E-mail: kitabayashi.yukiko@isen.ac.jp

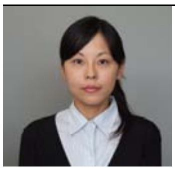

Aiko IMAI
Employment
Nagoya City University, Nagoya, JAPAN.
Degree
MSc
Research interests
Exercise effect on functional fitness of older adults
E-mail: imai-a@seijoh-u.ac.jp

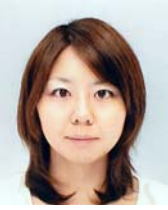

Aiko NARUSE
Employment
Active Aging Association, Nagoya, JAPAN.
Degree
MSc
Research interests
Effect of aquatic exercise for older adults
E-mail: aikohiko@gmail.com

✉ **Nobuo Takeshima**
National Institute of Fitness and Sports in Kanoya, Kanoya Japan
