# Supplementary material for: Effects of elastic band resistance training on the physical and mental health of elderly individuals: A mixed methods systematic review
Source: PLoS One. 2024 May 13;19(5):e0303372. doi: 10.1371/journal.pone.0303372 (PMC11090353; doi:10.1371/journal.pone.0303372)
Supplement: S1 File — (ZIP) [file pone.0303372.s001.zip › Supporting Information/Included study 47.pdf]

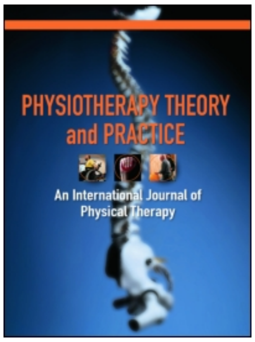

Taylor & Francis
Taylor & Francis Group

Physiotherapy Theory and Practice
An International Journal of Physical Therapy

ISSN: 0959-3985 (Print) 1532-5040 (Online) Journal homepage: https://www.tandfonline.com/loi/iptp20

Effects of elastic band exercise on the frailty states in pre-frail elderly people

Rujie Chen, Qingwen Wu, Dongyan Wang, Zhou Li, Howe Liu, Guangtian Liu, Ying Cui & Linlin Song

To cite this article: Rujie Chen, Qingwen Wu, Dongyan Wang, Zhou Li, Howe Liu, Guangtian Liu, Ying Cui & Linlin Song (2019): Effects of elastic band exercise on the frailty states in pre-frail elderly people, Physiotherapy Theory and Practice, DOI: 10.1080/09593985.2018.1548673

To link to this article: https://doi.org/10.1080/09593985.2018.1548673

Published online: 11 Feb 2019.

Submit your article to this journal

View Crossmark data

Full Terms & Conditions of access and use can be found at
https://www.tandfonline.com/action/journalInformation?journalCode=iptp20

PHYSIOTHERAPY THEORY AND PRACTICE
https://doi.org/10.1080/09593985.2018.1548673

Taylor & Francis
Taylor & Francis Group

Check for updates

Effects of elastic band exercise on the frailty states in pre-frail elderly people

Rujie Chen, MS[a], Qingwen Wu, MD[a], Dongyan Wang, MS[a], Zhou Li, BS[b], Howe Liu, PT, PhD, MD[c], Guangtian Liu, MS[a], Ying Cui, MS[a], and Linlin Song, MS[a]

[a]Rehabilitation Department, North China University of Science and Technology, Tangshan, Hebei, China; [b]School of Public Health, Southern Medical University, Guangzhou, Guangdong, China; [c]Physical Therapy Department, University of North Texas Health Science Center, Fort Worth, TX, USA

ABSTRACT

Objective: To discuss the effects of elastic band exercise on the frailty states in pre-frail elderly people.

Methods: This study was a randomized controlled trial. Trial registration number is ChiCTR-IOC-17012579. Seventy pre-frail elderly people were randomly divided into elastic band group (n = 35) and control group (n = 35). Elastic band exercise was applied to elastic band group, 45–60 min per time for 8 weeks by 3 days a week; no exercise was applied to the control group. The frailty states, grip strength (female/male), walking speed, and physical activity were measured by the Fried frailty phenotype at pre-intervention, 4, and 8 weeks after intervention to assess the effects of exercise.

Results: The elastic band group showed significant improvements in the frailty states, grip strength (female) and walking speed both after 4-week and 8-week intervention ($P < 0.001$), and significant improvements in grip strength (male) and physical activity after 8-week intervention ($P < 0.05$). Within-group analysis (pre-intervention vs. after 4-week, after 4-week vs after 8-week, pre-intervention vs after 8-week) showed significant improvements ($P < 0.001$) in grip strength (female/male) and walking speed in the elastic band group over time, while no significant differences in the control group ($P > 0.05$).

Conclusion: Elastic band exercise can improve frailty states in pre-frail elderly people, make them broke away from pre-frailty and restore them to non-frailty through improving the grip strength, walking speed and physical activity, and the effects after 8 weeks are better than those after 4 weeks.

ARTICLE HISTORY
Received 15 May 2018
Revised 1 September 2018
Accepted 28 September 2018

KEYWORDS
Elastic band exercise; pre-frail; elderly people; frailty states

Introduction

Frailty is a clinical state with increased vulnerability when exposed to an undesirable stimulation, resulting from age-related declines in physiologic reserve, and increases risks of adverse outcomes such as dependency, cognitive decline, disability, falls, hospitalization, institutionalization, and death (Langlois et al. 2013; Sánchez-García et al. 2017; Vaughan et al. 2015). Frailty reflects the problems of multiple physiological systems, which is more likely to be affected by multifaceted factors, so always in an ever-changing process (Jang 2016). Fried et al. (2001) divided the elderly into three stages by the frailty phenotype in 2001, respectively, were non-frailty, pre-frailty, and frailty. The phenotype consists of the following five physical symptoms: unintentional weight loss, self-reported exhaustion, low physical activity, slow walking speed, and weak grip strength. The presence of more than three criteria considers frailty, with 1–2 representing pre-frailty and the absence of criteria indicating non-frailty. Pre-frailty is a middle stage, in which elderly people change from non-frailty to frailty, belonging to the early stage of frailty. Similar to frailty, the prevalence of pre-frailty is higher in women than men (Choi et al. 2015; Sacha et al. 2017). Frailty may be reversible and preventable, pre-frail elderly people are more likely to return back to non-frail state than those who are frail (Gill et al. 2006), and preventing the level of frailty can lessen the burden from society and economy. In the whole stage of frailty, the early stage of frailty (pre-frailty) may be the optimal time to take intervention to prevent the frailty states of elderly people (McAdams-Demarco et al. 2013), so to identify people who may be pre-frail and provide early intervention is critical for prevention and treatment of frailty. However, there are few studies aimed at pre-frail elderly people and there is little evidence around what exercise intervention might work to prevent and restore the frailty states in pre-frail elderly people. Besides, most previous interventions largely focused on a single aspect (balance, muscle strength, quality of life, and so on) in

CONTACT Qingwen Wu, MD wxywqw@163.com Rehabilitation Department, North China University of Science and Technology, 21 bohai avenue, Caofeidian District, Tangshan, Hebei, China

Color versions of one or more of the figures in the article can be found online at www.tandfonline.com/iptp.

© 2019 Taylor & Francis Group, LLC

frail elders, the studies which using frailty phenotype as a measurement to evaluate the frailty states in pre-frail elders after intervention are lacked.

Exercise intervention is a necessary and key way for the prevention and treatment of frailty (Chen et al. 2014; Frost et al. 2017; Landi et al. 2010; Wang et al. 2017), there are many exercise forms for frail elderly people, usually including resistance, aerobic (Tousignant et al. 2013), balance, and multicomponent training (resistance, aerobic, balance, flexibility, and so on) (Cesari et al. 2015; Tarazona-Santabalbina et al. 2016), resistance exercise seems to be a critical element (Sacha et al. 2017). Elastic band exercise belongs to a home-based elastic resistance exercise (Oh et al. 2017), which alternates multiple muscle groups during exercise and gives the muscles sufficient time to recover, then avoids the risk of muscle injuries after nerve fatigue and stimulates the mobilization of muscle, consequently improving training effects (Si 2010). Elastic band exercise is a common exercise in the elderly, which can not only induce improvements in muscle strength, gait function, flexibility, fall efficacy, balance, and quality of life, but also reduce the risk of injuries (Kwak et al. 2016; Lin et al. 2015; Oh et al. 2017; Park et al. 2015). The safety and efficacy of using the elastic band in elderly people also have been proven (Lin et al. 2015). Therefore, we carried out this study to evaluate whether the elastic band exercise can prevent the frailty states, and improve grip strength (female/male), walking speed, and physical activity in pre-frail elderly people.

Methods

Participants

Participants were recruited from the community center through giving out leaflets, posting leaflets on the bulletin board or word of mouth. The sample size was calculated using the formula for a comparison of two samples mean values, 0.05 was set as the significant level, $u_\alpha = 1.96$; $\beta = 0.1$, $u_\beta = 1.282$; the calculation was based on walking speed: $\delta = 1.7$, $\sigma = 2.0$. Then these parameters generated a sample size of 31 participants for each group. Given the missing rate, the sample size increased by 10% to 70 cases. Seventy pre-frail community-dwelling elderly people in wenzhou city participated in this study from October 2017 to March 2018, they were randomly divided into elastic band group and control group, 35 people in each group. The allocation was implemented by a research assistant, who selected 70 random numbers from the table of random number. These numbers were put into a sealed box. Then participants who chose even numbers were assigned to the elastic band group and odd numbers were assigned to the control group. All participants signed informed consent and this study was approved by the Ethics Committee of the principle investigator's institution (2017241). In this study, two participants of elastic band group dropped out due to personal reasons and low attendance rate, two participants of the control group were lost for change of contact information. Finally, there were 66 people completed the study, 33 people in each group (Flow Diagram 1).

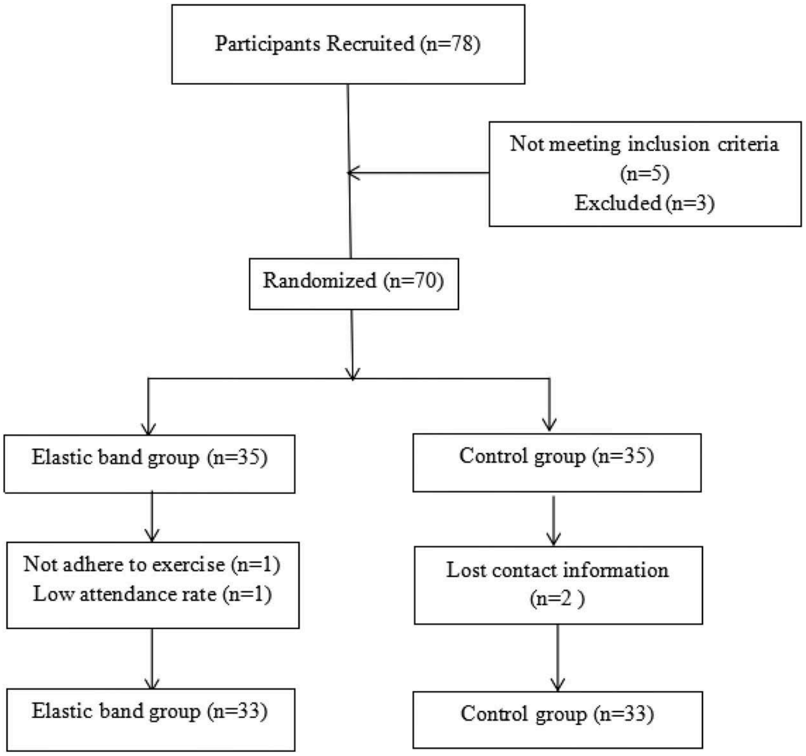

Diagram 1. Allocation and flow of participants.

Inclusion criteria

The inclusion criteria were aimed at pre-frail elderly people (who met 1–2 criteria based on the Fried frailty phenotype: unintentional weight loss, self-reported exhaustion, low physical activity, slow walking speed, weak grip strength), age between 65 and 85 years old, having no understanding/hearing/visual impairment and vestibular/cerebellar dysfunction, could follow general commands and communicate normally, could walk independently without assistance, having no other organized exercise training except elastic band exercise during intervention and voluntary participation in the study.

As the prevalence of frailty increases with age, older people (age 85 and over) are more likely to be frail than people who less than 85 (Fried et al. 2001), and our participants were aimed at pre-frail people. Besides, there are some comorbidities which may not be suitable for exercise in these older people (age 85 and over), so considering the safety of participants, we chose older people (65–85 years old) as participants.

There were five criteria presenting in the frailty phenotype (Fried et al. 2001): (1) unintentional weight loss, weight loss (not due to dieting or exercise) more than 4.5 kg in last 12 months; (2) self-reported exhaustion, you felt that everything you did was an effort or you could not get going, either of these two feelings appeared more than 3 days in last 7 days; (3) low physical activity, answering "yes" to either of the following two questions was considered as a positive result: "Having limits to some activities in daily life due to physical reasons, including moving the table, sweeping the floor, playing tai-chi, and simple gymnastics, climbing a few flights of stairs and so on" and "In the last month, the things (work or daily activities) you wanted to do only could be done in half due to physical reasons, or the type of work or daily activities was limited"; (4) slow walking speed, walk 4.5 m, men (≤ 173 cm) or women (≤ 159 cm) need more than 7 s, men (> 173 cm) or women (> 159 cm) need more than 6 s; and (5) weak grip strength, measured dominant hand three times and recorded mean value, then stratified by gender and BMI (i.e. men: BMI ≤ 24, grip strength ≤ 29 kg; BMI 24.1–28, grip strength ≤ 30 kg; BMI > 28, grip strength ≤ 32 kg and women: BMI ≤ 23, grip strength ≤ 17 kg; BMI 23.1–26, grip strength ≤ 17.3 kg; BMI 26.1–29, grip strength ≤ 18 kg; BMI > 29, grip strength ≤ 21 kg).

Score: 1 criterion represented 1 point; the total score was 5 points. The higher the score, the more severe the frailty states, those with 3 or more points were considered frailty, with 0 points were considered non-frailty and those with 1–2 points were hypothesized to be pre-frail.

Exclusion criteria

Participants were excluded if they had severe skeletal muscle diseases and serious diseases (e.g. heart, lung, liver, and kidney), with a previous history of mental illness or antipsychotic medication, unwilling to participate in the study or planning to be hospitalized in the next half year or participate in other organized exercise training, having regular exercise (more than 3 days/week) or good exercise levels, and having low attendance rate (<85%). Participants who died, appeared serious diseases or injury, dropped out in the process of study and could not continue due to adverse events such as cardiovascular and motor system during the study were also excluded.

Intervention

The control group maintained normal daily activity and did not receive any special intervention, irregular exercise (less than 2 days a week) or sedentary. The elastic band group was treated with elastic band exercise and intervention was conducted for 8 weeks, 3 days a week, 45–60 min per time, including warm-up activities before exercise and relaxed activities after exercise. We chose the yellow elastic band (JOINFIT natural latex elastic band) for exercise.

The duration of the interventions in frail people ranged from 1 to 18 months, and the common duration was 3 months (Theou et al. 2011), but after feedback from participants and considering the actual situation, pre-frail elderly people are more likely to return back to non-frail state than those who are frail (Gill et al. 2006), we selected 8 weeks (2 months) as intervention time.

All participants of the elastic band group were divided into three exercise groups, 11–12 participants in each group. Three exercise groups were led by a same intervener who was familiar with each exercise movement in different time. The intervener should receive twice elastic band training and pass the assessment. A physiotherapist or a trained community center worker could be the intervener who should have a strong sense of responsibility, high enthusiasm for this research and good communication skills. The intervention was delivered at the community room. For the safety consideration, we asked participants about their physical condition and measured their heart rate and blood pressure before each exercise session.

Eight exercise movements: (Figures 1–8) (1) Tied both sides of band to both hands and stretched the arms, left hand kept still and right hand tried to pull the band while withstanding the resistance of the band. (2) Passed the band behind the back and tied both sides of band to both hands, bended the elbows, then tried to straighten

the elbows forward. (3) Right foot stood on the side of the band and tied the other side to the right hand, then tried to abduct the right upper limb. (4) Tied the band to the left ankle and tied the other side to the right ankle, then tried the right hip joint flexion upward. (5) Sat on the chair, bended the knee joint 90 degrees, tied the band to the left ankle and tied the other side to the right ankle, then tried to extend the right knee. (6) Tied the band to the left ankle and tied the other side to the right ankle, then tried the right hip joint extension backwards. (7) Stood on the left/right leg for as long as possible and take turns, 2 times for each leg, 4–5 min. (8) On tiptoe, 20 repeats.

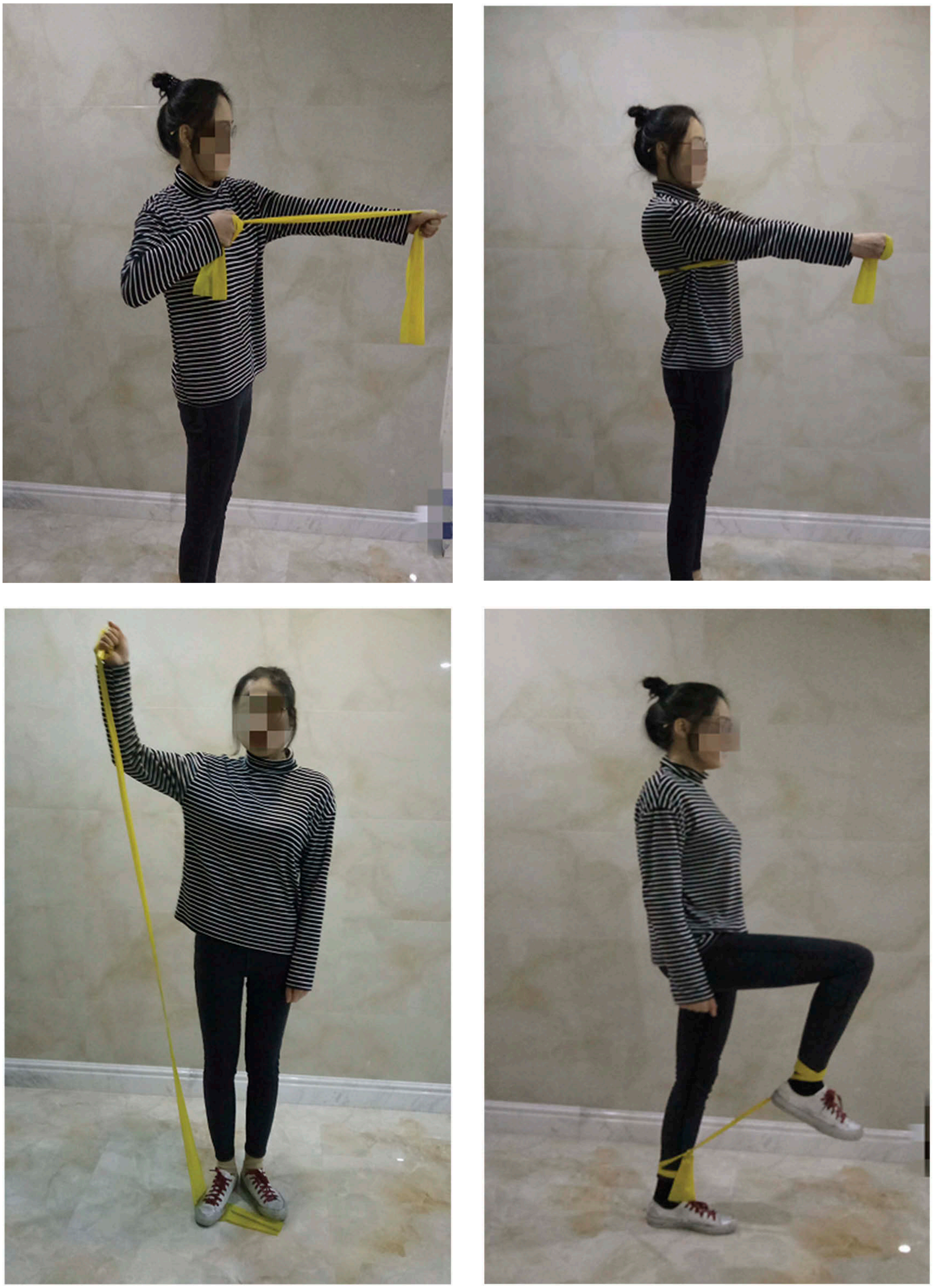

Figures 1–8. Steps in details for the eight exercise movement.

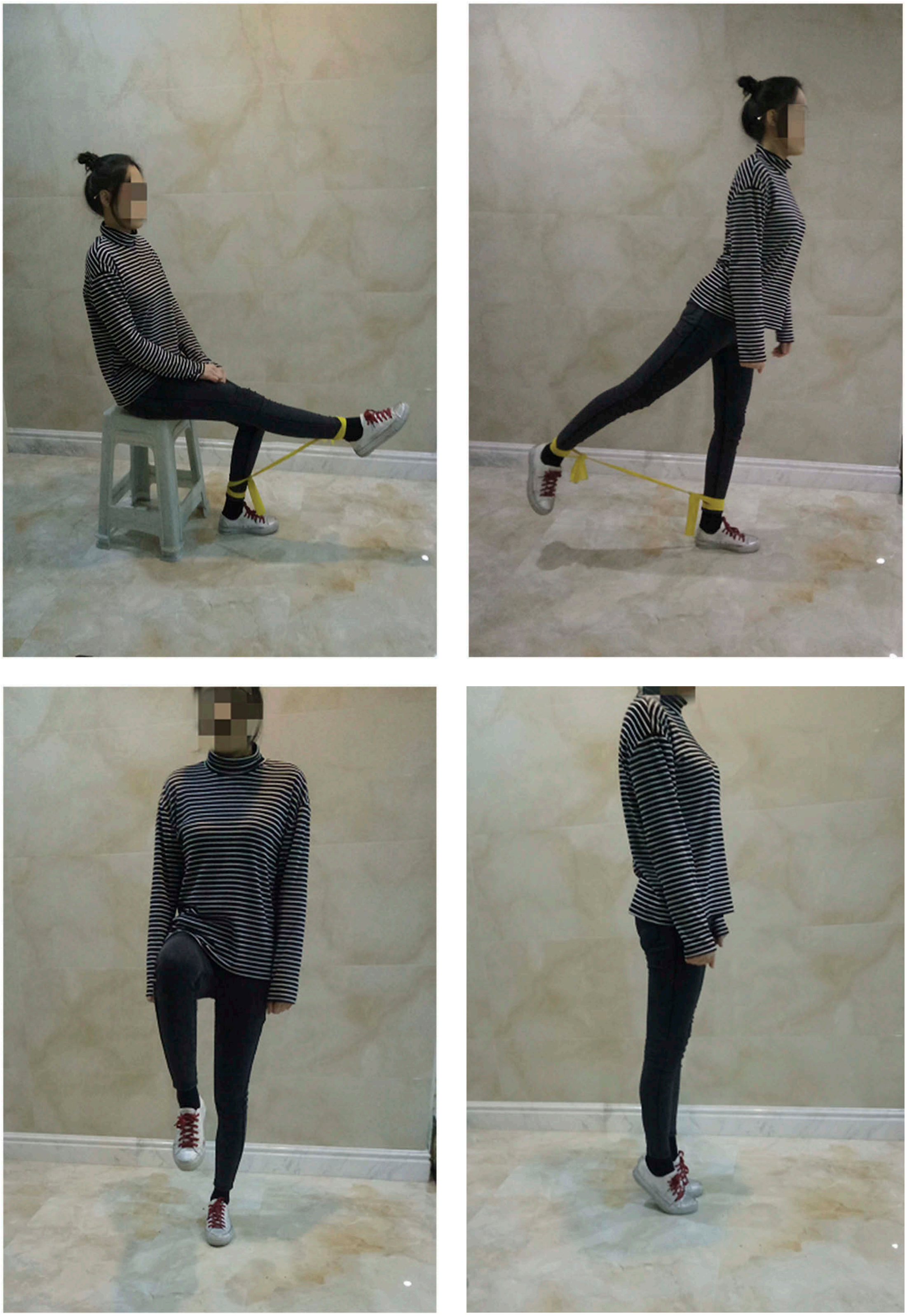

Figures 1–8. (Continued).

Each movement was conducted 2 sets, 10–15 repeats per set; no elastic band for movement 7 and 8; both left and right were performed alternately; 1–2 min rest between each set; allowed to seize the chair or desk to maintain balance for safety.

The other outcomes (i.e. Berg balance scale, fear of falling, timed up and go, functional reach, sit-to-stand, and quality of life) were also measured and will be reported in subsequent manuscripts.

Measurements

The measurements of the frailty states, grip strength (female/male), walking speed, and physical activity were tested by using the Fried frailty phenotype at pre-intervention, 4 and 8 weeks after the intervention to assess the exercise effects of the two groups by a blinded assessor.

Data analysis

All data was recorded in Excel, established the database, and analyzed by SPSS version 17.0. Data is presented as number, percentages, means, and standard deviations. Comparison of enumeration data used Chi-squared for trend test; an independent sample t-test was used to calculate the between-group differences at pre-intervention, 4, and 8 weeks after intervention. Repeated measurement analysis of variance was used to determine within-group differences, and statistical significance was set at 0.05.

Results

General characteristics

There showed no statistically significant differences in gender, age, height, weight, BMI between two groups ($P > 0.05$) (Table 1). In addition, one participant reported unintentional weight loss and no one developed exhaustion before intervention in our trial. So we excluded weight loss and exhaustion as outcome measures.

Table 1. General characteristics of participants (n = 33).

	Elastic band	Control	t/χ^2	P
Gender (n)				
Male	12	11	0.067	0.796
Female	21	22		
Age (year)	76.97 ± 5.19	75.27 ± 5.98	1.231	0.223
Height (cm)	155.11 ± 8.04	154.58 ± 5.68	0.309	0.758
Weight (kg)	54.72 ± 7.13	54.11 ± 7.88	0.334	0.739
BMI (kg/m^2)	22.79 ± 2.85	22.64 ± 3.11	0.201	0.841

Value are mean ± SD.

Frailty states

Before intervention, the difference of frailty states was not statistically significant between two groups (χ^2 = 1.032, P = 0.310). After 4 weeks, there were 17 participants (51.5%) returned to non-frailty from pre-frailty in elastic band group while only 2 participants (6.1%) in control group, which showed significant difference between two groups (χ^2 = 19.541, P = 0.000). After 8 weeks, there were 27 participants (81.8%) returned to non-frailty in elastic band group while only 3 participants (9.1%) in control group, 1 people (3.0%) even entered to frailty in control group, which showed significant difference between two groups (χ^2 = 33.763, P = 0.000). (Table 2).

Grip strength

Before intervention, the between-group differences of grip strength (female/male) were not statistically significant (female: d = 0.65, 95% CI −0.98 to 2.28, P = 0.428; male: d = −0.87, 95% CI −3.28 to 1.55, P = 0.464). There showed significant differences of grip strength in female (d = 3.62, 95% CI 2.0 to 5.24, P = 0.000) at 4-week after intervention, and in both female and male at 8-week after intervention between two groups (female: d = 6.03, 95% CI 4.35 to 7.72, P = 0.000; male: d = 3.97, 95% CI 1.05 to 6.88, P = 0.010) (Table 3). The within-group comparison of grip strength (pre-intervention vs. after 4-week, after 4-week vs after

Table 2. The comparison of the frailty states (number, %, n = 33).

Groups	Interval	Non frailty (0 point)	Pre-frailty I (1 points)	Pre-frailty II (2 points)	Frailty (≥ 3 points)
Elastic band	Pre	0	23 (69.7)	10 (30.3)	0
	4 weeks*	17 (51.5)	15 (45.5)	1 (3.0)	0
	8 weeks*	27 (81.8)	6 (18.2)	0	0
Control	Pre	0	19 (57.6)	14 (42.4)	0
	4 weeks	2 (6.1)	21 (63.6)	10 (30.3)	0
	8 weeks	3 (9.1)	17 (51.5)	12 (36.4)	1 (3.0)

*Statistically significant between-group difference, respectively, at 4-week and 8-week after intervention (P < 0.001).

Table 3. The comparison of grip strength.

	Groups	n	Pre	4 weeks	8 weeks
GS(f)	Elastic band	21	16.73 ± 2.42	19.92 ± 2.84#	21.76 ± 2.84#*
	Control	22	16.08 ± 2.84	16.30 ± 2.42	15.73 ± 2.61
	t		0.801	4.510	7.251
	P		0.428	0.000	0.000
GS(m)	Elastic band	12	25.90 ± 3.06	28.87 ± 3.23#	30.76 ± 4.11#*
	Control	11	26.77 ± 2.44	26.95 ± 2.34	26.79 ± 2.26
	t		−0.745	1.622	2.830
	P		0.464	0.120	0.010

Value are mean± SD, GS: grip strength (kg), (f): female, (m): male.

# Statistically significant difference compared with pre-intervention (GS(f): pre vs 4 weeks: t = −7.056, P = 0.000; pre vs 8 weeks: t = −8.823, P = 0.000; GS(m): pre vs 4 weeks: t = −7.630, P = 0.000; pre vs 8 weeks: t = −10.430, P = 0.000).

* Statistically significant difference compared with 4 weeks (GS(f): t = −5.571, P = 0.000; GS(m): t = −5.672, P = 0.000).

8-week, pre-intervention vs after 8-week) showed statistically significant improvements of grip strength in both female and male in elastic band group over time (female: pre vs. 4 weeks: t = −7.056, P = 0.000; 4 weeks vs 8 weeks: t = −5.571, P = 0.000; pre vs 8 weeks: t = −8.823, P = 0.000; male: pre vs 4 weeks: t = −7.630, P = 0.000; 4 weeks vs 8 weeks: t = −5.672, P = 0.000; pre vs 8 weeks: t = −10.430, P = 0.000); while no change in control group ($P > 0.05$) (Table 3).

Walking speed

Before intervention, the difference of walking time between the two groups was not statistically significant (d = −0.38, 95% CI −0.84 to 0.07, P = 0.097). After 4 weeks and 8 weeks, walking time in elastic band group were both shortened and differences were statistically significant between two groups (d = −1.17, 95% CI −1.53 to −0.81, P = 0.000; d = −1.65, 95% CI −2.05 to −1.26, P = 0.000) (Table 4). The within-group comparison of walking time (pre-intervention vs after 4-week, after 4-week vs after 8-week, pre-intervention vs after 8-week) showed statistically significant reduction of walking time in elastic band group over time (pre vs 4 weeks: t = 6.146, P = 0.000; 4 weeks vs 8 weeks: t = 4.673, P = 0.000; pre vs. 8 weeks: t = 8.783, P = 0.000) and no change in the control group ($P > 0.05$), those results demonstrated that with the increase of the intervention time, walking time was shortened and walking speed was obviously improved (Table 4).

Physical activity

Before intervention and after 4 weeks, there showed no significant differences in physical activity between the two groups (χ^2 = 0.062, P = 0.804; χ^2 = 0.655, P = 0.418). After 8 weeks, there were only two participants (6.1%) had low physical activity in elastic band group while 11 participants (33.3%) in control group, which showed significant difference between two groups (χ^2 = 7.642, P = 0.006) (Table 5).

Table 4. The comparison of walking time (n = 33).

Groups	n	Pre	4 weeks	8 weeks
Elastic band	33	5.59 ± 0.91	4.73 ± 0.51[#]	4.32 ± 0.57[#*]
Control	33	5.98 ± 0.95	5.90 ± 0.91	5.98 ± 0.97
t		−1.685	−6.483	−8.472
P		0.097	0.000	0.000

Value are mean ± SD, walking time (s)
[#]Statistically significant difference compared with pre-intervention (pre vs 4 weeks: t = 6.146, P = 0.000; pre vs 8 weeks: t = 8.783, P = 0.000)
*Statistically significant difference compared with 4 weeks (4 weeks vs 8 weeks: t = 4.673, P = 0.000)

Discussion

Currently, the frailty has become a hot spot of gerontology among health problems in elderly people. Frailty remains an evolving concept that can change over time in clinical practice and epidemiological investigations (Morley et al. 2013; Nourhashémi et al. 2001; Sánchez-García et al. 2017). Frailty is to dynamically develop and the degree is diverse in different stages, the process of frailty can be changed (Gill et al. 2006). Effective interventions in pre-frailty may delay or postpone the development of frailty (Apóstolo et al. 2018; Theou et al. 2011; Xi et al. 2015), otherwise as the frailty grows worse, intervention will be more difficult.

Elastic band exercise has no need for external load, exercise intensity increases with the length of the elastic band, the exerciser can achieve muscle contraction through adjusting the direction and intensity of load, consequently enhancing muscle strength and improving motor function (Carlos et al. 2016; Kwak et al. 2016). Due to inexpensive, safety, easy-to-use and various forms, elastic band is widely used for physical training of athletes and teenagers, clinical rehabilitation, and functional exercise of older adults (Chang et al. 2012; Chen et al. 2015; Lee et al. 2015; Yu et al. 2013), and it is also suitable for rural areas with poor training conditions or levels (Park et al. 2015).

The tension force of elastic band increases as the elongation percentage, the tension values of the different-colored elastic band have statistically differences (lowest at tan and highest at gold), excepting tan/yellow pair (Carlos et al. 2016), so yellow elastic band is suitable for older people.

Our randomized controlled trial found that pre-frail elderly people who carried out 8 weeks of elastic band exercise experienced an increase in grip strength and walking speed, the effects after 8 weeks were better than

Table 5. The comparison of physical activity (number, %, n = 33).

Groups	Interval	Low	Nomal
Elastic band	Pre	13 (39.4)	20 (60.6)
	4 weeks	8 (24.2)	25 (75.8)
	8 weeks*	2 (6.1)	31 (93.9)
Control	Pre	14 (42.4)	19 (57.6)
	4 weeks	11 (33.3)	22 (66.7)
	8 weeks	11 (33.3)	22 (66.7)

*Statistically significant between-group difference at 8-week after intervention (χ^2 = 7.642, P = 0.006).

those after 4 weeks. These findings are consistent with Rogers et al. (2002) who found an increase in grip strength after 4 weeks of elastic band exercise and Kwak et al. (2016) who found an increase in walking ability after 8 weeks. Furthermore, the results of grip strength in females were more positive than those in males, this phenomenon is consistent with Theou et al. (2011) who reported that females have been shown to have more room for exercise improvements compared to males.

In this study, participants who had low physical activity at pre-intervention reduced from 13 to 2 in the elastic band group after 8 weeks, in contrast to 11 participants in the control group, which represented a significant improvement in physical activity after 8 weeks of elastic band exercise. While no improvement in physical activity after 4 weeks compared to 8 weeks, which indicates that 4-weeks intervention is not enough to improve the physical activity in pre-frail elderly people, elastic band exercise may improve muscle strength and walking ability first, then enhance the level of physical activity. Low grip strength was the most common indicator in participants at pre-intervention in our trial, low physical activity, and slow walking speed were the second and third, these findings are consistent with Xue et al. (2008) who also found that people with unintentional weight loss or exhaustion were more likely to be frail.

The frailty states showed an improving trend in this study, which may be correlated with the increases in grip strength, walking speed, and physical activity. Seventeen participants restored to non-frailty after 4 weeks and the number added up to 27 after 8 weeks, while only 3 participants were non-frail in control group after 8 weeks. These findings indicate that elastic band exercise is helpful to prevent the evolution of frailty states in pre-frail elderly people and the effect after 8 weeks is more effective than it after 4 weeks; elderly people in the control group can return to non-frailty through active self-regulate, but the effect is inferior to elastic band exercise. Besides, 1 participant in the control group even entered to frailty after 8 weeks, which demonstrates that frailty states of elderly people may aggravate without regular exercise intervention in time.

The findings of the study are positive. One of the possible explanations is that our study used team training, which was good for communication and supervision. There were no adverse events reported during the study. And the exercise adherence was high, only one participant in elastic band group dropped out due to low attendance (<85%), suggesting that team training may provide motivation and elderly people find elastic band exercise to be acceptable. Furthermore, before the intervention, each exercise movement was explained and demonstrated to participants until they could follow instructions to complete each movement correctly, which may promote the effects of exercise. Finally, the intervention was carried out in a community room under the supervision rather than at home, which also resulted in high compliance.

This study has several limitations which need to be considered. First, the inclusion criteria did not include those older people who had some visual or hearing impairment or a serious disease, which reduces the generalizability of the results. Second, lack of participant blinding and small sample size in the study is another limitation of the study. Third, the study period is short, and without a follow-up observation after the cessation of study, the long-term effect of elastic band exercise remains to be confirmed. Additional studies with larger sample sizes and longer study time should be performed to further investigate this intervention.

This study provides evidence that elastic band exercise can improve frailty states in pre-frail elderly people, make them broke away from pre-frailty and restore them to non-frailty through improving grip strength, walking speed and physical activity, the effects after 8 weeks are better than those after 4 weeks. And this study may provide a theoretical reference for finding an exercise way to prevent and restore the frailty states in pre-frail elderly people.

Acknowledgments

The authors would like to acknowledge the participation of all participants in this study.

Disclosure of interest

The authors report no conflict of interest.

References

Apóstolo J, Cooke R, Bobrowicz-Campos E, Santana S, Marcucci M, Cano A, Vollenbroek-Hutten M, Germini F, D'Avanzo B, Gwyther H, et al. 2018. Effectiveness of interventions to prevent pre-frailty and frailty progression in older adults: A systematic review. JBI Database System Rev Implement Rep. 16:140–232.

Carlos UM, Makoto NM, Carvalho SRA, Toshio M, Hidenori A. 2016. Thera-band® elastic band tension: reference values for physical activity. J Phys Ther Sci. 28:1266–71.

Cesari M, Vellas B, Hsu FC, Newman AB, Doss H, King AC, Manini TM, Church T, Gill TM, Miller ME; LIFE Study Group. 2015. A physical activity intervention to treat the frailty syndrome in older persons-results from the life-p study. J Gerontol. 70:216–22.

Chang TF, Liou TH, Chen CH, Huang YC, Chang KH. 2012. Effects of elastic-band exercise on lower-extremity function among female patients with osteoarthritis of the knee. Disabil Rehabil. 34:1727–35.

Chen KM, Li CH, Chang YH, Huang HT, Cheng YY. 2015. An elastic band exercise program for older adults using wheelchairs in taiwan nursing homes: A cluster randomized trial. Int J Nurs Stud. 52:30–38.

Chen X, Mao G, Leng SX. 2014. Frailty syndrome: an overview. Clin Interv Aging. 9:433–41.

Choi J, Ahn A, Kim S, Chang WW. 2015. Global prevalence of physical frailty by fried's criteria in community-dwelling elderly with national population-based surveys. J Am Med Dir Assoc. 16:548–50.

Fried LP, Tangen CM, Walston J, Newman AB, Hirsch C, Gottdiener J, Seeman T, Tracy R, Kop WJ, Burke G; Cardiovascular Health Study Collaborative Research Group. 2001. Frailty in older adults: evidence for a phenotype. J Gerontol 56:M146–156.

Frost R, Belk C, Jovicic A, Ricciardi F, Kharicha K, Gardner B, Iliffe S, Goodman C, Manthorpe J, Drennan VM, et al. 2017. Health promotion interventions for community-dwelling older people with mild or pre-frailty: A systematic review and meta-analysis. BMC Geriatr. 17:157.

Gill TM, Gahbauer EA, Allore HG, Han L. 2006. Transitions between frailty states among community-living older persons. Arch Intern Med. 166:418–23.

Jang HC. 2016. Sarcopenia, frailty, and diabetes in older adults. Diabetes Metab J. 40:182–89.

Kwak CJ, You LK, Lee SM. 2016. Effects of elastic-band resistance exercise on balance, mobility and gait function, flexibility and fall efficacy in elderly people. J Phys Ther Sci. 28:3189–96.

Landi F, Abbatecola AM, Provinciali M, Corsonello A, Bustacchini S, Manigrasso L, Cherubini A, Bernabei R, Lattanzio F. 2010. Moving against frailty: does physical activity matter? Biogerontology. 11:537–45.

Langlois F, Vu TT, Chassé K, Dupuis G, Kergoat MJ, Bherer L. 2013. Benefits of physical exercise training on cognition and quality of life in frail older adults. J Gerontol. 68:400–04.

Lee HC, Lee ML, Kim SR. 2015. Effect of exercise performance by elderly women on balance ability and musclefunction. J Phys Ther Sci. 27:989–92.

Lin SF, Sung HC, Li TL, Hsieh TC, Lan HC, Perng SJ, Smith GD. 2015. The effects of tai-chi in conjunction with thera-band resistance exercise on functional fitness and muscle strength among community-based older people. J Clin Nurs. 24:1357–66.

McAdams-Demarco MA, Suresh S, Law A, Salter ML, Gimenez LF, Jaar BG, Walston JD, Segev DL. 2013. Frailty and falls among adult patients undergoing chronic hemodialysis: A prospective cohort study. BMC Nephrol. 14:224.

Morley JE, Vellas B, van Kan GA, Anker SD, Bauer JM, Bernabei R, Cesari M, Chumlea WC, Doehner W, Evans J, et al. 2013. Frailty consensus: A call to action. J Am Med Dir Assoc. 14:392–97.

Nourhashémi F, Andrieu S, Gilletteguyonnet S, Vellas B, Albarède JL, Grandjean H. 2001. Instrumental activities of daily living as a potential marker of frailty a study of 7364 community-dwelling elderly women (the epidos study). J Gerontol A Biol Sci Med Sci. 56:M448–453.

Oh SL, Kim HJ, Woo S, Cho BL, Song M, Park YH, Lim JY, Song W. 2017. Effects of an integrated health education and elastic band resistance training program on physical function and muscle strength in community-dwelling elderly women: healthy aging and happy aging II study. Geriatr Gerontol Int. 17:825–33.

Park SY, Kim JK, Lee S. 2015. The effects of a community-centered muscle strengthening exercise program using an elastic band on the physical abilities and quality of life of the rural elderly. J Phys Ther Sci. 27:2061–63.

Rogers ME, Sherwood HS, Rogers NL, Bohlken RM. 2002. Effects of dumbbell and elastic band training on physical function in older inner-city African-American women. Women Health. 36:33–41.

Sacha J, Sacha M, Soboń J, Borysiuk Z, Feusette P. 2017. Is it time to begin a public campaign concerning frailty and pre-frailty? A review article. Front Physiol. 8:484.

Sánchez-García S, García-Peña C, Salvà A, Sánchez-Arenas R, Granados-García V, Cuadros-Moreno J, Velázquez-Olmedo LB, Cárdenas-Bahena Á. 2017. Frailty in community-dwelling older adults: association with adverse outcomes. Clin Interv Aging. 12:1003–11.

Si WC. 2010. Application of elastic band in the three joints link strength training with practice in China national aerials team. Doctoral dissertation, Beijing Sports University.

Tarazona-Santabalbina FJ, Gómez-Cabrera MC, Pérez-Ros P, Martínez-Arnau FM, Cabo H, Tsaparas K, Salavador-Pascual A, Rodriguez-Manas L, Vina J. 2016. A multicomponent exercise intervention that reverses frailty and improves cognition, emotion, and social networking in the community-dwelling frail elderly: A randomized clinical trial. J Am Med Dir Assoc. 17:426–33.

Theou O, Stathokostas L, Roland KP, Jakobi JM, Patterson C, Vandervoort AA, Jones GR. 2011. The effectiveness of exercise interventions for the management of frailty: A systematic review. J Aging Res. 2011:569194.

Tousignant M, Corriveau H, Roy PM, Desrosiers J, Dubuc N, Hébert R. 2013. Efficacy of supervised tai chi exercises versus conventional physical therapy exercises in fall prevention for frail older adults: a randomized controlled trial. Disabil Rehabil. 35:1429–35.

Vaughan L, Corbin AL, Goveas JS. 2015. Depression and frailty in later life: A systematic review. Clin Interv Aging. 10:1947–58.

Wang YN, Xu JL, Song HL, Guo YJ. 2017. Advance of exercise therapy for frailty (review). Zhongguo Kangfu Lilun Yu Shijian. 23:558–62.

Xi X, Guo GF, Sun J. 2015. Frailty assessment tool for elderly people and its the application study progress. Chin J Gerontol. 35:5993–96.

Xue QL, Bandeen-Roche K, Varadhan R, Zhou J, Fried LP. 2008. Initial manifestations of frailty criteria and the development of frailty phenotype in the women's health and aging study II. J Gerontol A Biol Sci Med Sci. 63:984–90.

Yu W, An C, Kang H. 2013. Effects of resistance exercise using thera-band on balance of elderly adults: A randomized controlled trial. J Phys Ther Sci. 25:1471–73.
