# Supplementary material for: Effects of elastic band resistance training on the physical and mental health of elderly individuals: A mixed methods systematic review
Source: PLoS One. 2024 May 13;19(5):e0303372. doi: 10.1371/journal.pone.0303372 (PMC11090353; doi:10.1371/journal.pone.0303372)
Supplement: S1 File — (ZIP) [file pone.0303372.s001.zip › Supporting Information/Included study 53.pdf]

International Journal of Nursing Studies 52 (2015) 30–38

Contents lists available at ScienceDirect

International Journal of Nursing Studies

journal homepage: www.elsevier.com/ijns

An elastic band exercise program for older adults using wheelchairs in Taiwan nursing homes: A cluster randomized trial

Kuei-Min Chen [a,*], Chun-Huw Li [b,1], Ya-Hui Chang [a,2], Hsin-Ting Huang [a,2], Yin-Yin Cheng [a,2]

[a] College of Nursing, Kaohsiung Medical University, 100 Shih-Chuan 1st Rd., Sanming District, Kaohsiung 80708, Taiwan
[b] Department of Nursing, Yuhing Junior College of Health Care and Management, No. 15, Lane 420, Dachang 2nd Rd., Sanmin District, Kaohsiung 80776, Taiwan

ARTICLE INFO

Article history:
Received 14 January 2014
Received in revised form 28 May 2014
Accepted 12 June 2014

Keywords:
Cluster randomized trial
Elastic band
Functional fitness
Nursing home
Older adults
Wheelchair exercise

ABSTRACT

Background: The number of older adults using wheelchairs in nursing homes is over 50% of that population, and many of them use wheelchairs due to muscle weakness in the lower extremities. Muscles of older adults are trainable, and progressive resistance exercises using elastic bands can increase muscle strength in older adults.
Objectives: To test the effectiveness of six-month Wheelchair-bound Senior Elastic Band exercises on the functional fitness of older adults in nursing homes.
Design: Cluster randomized trial.
Settings: Ten nursing homes, southern Taiwan.
Participants: 127 participants were recruited, and 114 of them completed the study. Inclusion criteria were: (1) aged 65 and over, (2) using wheelchairs for mobility, (3) living in the facility for at least three months, (4) cognitively intact, and (5) heavily or moderate dependency in their activities of daily living. The mean age of the participants was 79.15 (7.03) years, and 98.20% of them had chronic illnesses.
Methods: Participants were randomly assigned to the experimental (five nursing homes, $n = 59$) or the control (five nursing homes, $n = 55$) group based on the nursing homes where they stayed. A 40-min Wheelchair-bound Senior Elastic Band exercise program was implemented three times per week for six months for the experimental group participants. The functional fitness (activities of daily living, lung capacity, body flexibilities, muscle power and endurance) of the participants was examined at baseline, after three months, and at the end of the six months study. The mixed-design, two-way analysis of variance was used to detect the interaction effects, and one-way repeated measures analysis of variance and analysis of covariance were performed to analyze the within-group and between-group differences.

* Corresponding author. Tel.: +886 7 3136900.
E-mail addresses: kmc@kmu.edu.tw, kueimin@yahoo.com (K. M. Chen), numa@ms.yuhing.edu.tw (C. H. Li), hui7742@yahoo.com.tw (Y. H. Chang), qwety789@livemail.tw (H. T. Huang), kuyin3641@yahoo.com.tw (Y. Y. Cheng).
[1] Tel.: +886 917665157.
[2] Tel.: +886 7 3136900.

http://dx.doi.org/10.1016/j.ijnurstu.2014.06.005
0020-7489/© 2014 Elsevier Ltd. All rights reserved.

Results: At the end of the six-month study, the Wheelchair-bound Senior Elastic Band group had better performances in all of the functional fitness indicators than the control group (all $p < 0.05$).

Conclusions: The Wheelchair-bound Senior Elastic Band exercises significantly improved the functional fitness of the older adults in wheelchairs. It is suggested that the program be incorporated as a part of daily activities for nursing home older adults in wheelchairs.

© 2014 Elsevier Ltd. All rights reserved.

What is already known about the topic?

- Many of the older adults using wheelchairs in nursing homes were due to muscle weakness in the lower extremities caused by previous falls, strokes, or inactivity.
- Muscles of the older adults are trainable, and progressive resistance exercises using elastic bands can increase muscle strength in older adults.
- The Wheelchair-bound Senior Elastic Band (WSEB) exercise program has been evaluated by experts and pilot-tested with older adults in wheelchairs with positive qualitative feedback.

What this paper adds

- The experimental group had better functional fitness than the control group after six months of the WSEB exercises.
- Nursing home directors could recruit volunteers to teach the WSEB exercise program and lead the older adults to practice the WSEB exercises in groups regularly in the facilities.

1. Introduction

About 5% of the older adults in the US (Karmarkar et al., 2011) and 2.7% in Taiwan (Yang et al., 2011) live in institutional settings. The number of older adults using wheelchairs in nursing homes is over 50% of that population (Kaye et al., 2002), and many of them use wheelchairs due to muscle weakness in the lower extremities caused by previous falls, strokes, or inactivity (Karmarkar et al., 2011). Wheelchair use is one of the barriers to physical activity (Rimmer, 2005), which might lead to further disability and mortality in older adults (Hirvensalo et al., 2000). Shore (2008) has reported that 12.3% of older adults in wheelchairs experienced worsening health. Muscle strength is essential in completing the activities of daily living (Topp et al., 1994), and the muscles of older adults are as trainable as those of younger adults. Progressive resistance exercises, including those using elastic bands, can increase muscle strength and size in older adults (Brown et al., 1990).

Elastic band exercise is documented as a safe and effective strategy to enhance the neuromuscular system, improve the muscle strength and power, and increase the ability to perform functional tasks of older adults (Galvao and Taaffe, 2005). The inherent properties of the elastic band could accommodate the length-tension characteristics of joint and muscle actions (Patterson et al., 2001). By changing the thickness and length of the elastic belt, resistance training can be flexibly adjusted to meet the needs of populations with different levels of body functioning (Damush and Damush, 1999). Evidence-based research supports that elastic band exercises are beneficial for both healthy and frail older adults (Dancewicz et al., 2003; Topp et al., 2002). The resistance exercise program using elastic bands improved maximal voluntary thigh muscle strength (Binder et al., 2005), knee extension and hip extension strength (Dancewicz et al., 2003), and sit-to-stand performance of community-dwelling older adults (Chen et al., 2009). Further, elastic band resistance training improved functional ability (Topp et al., 2005), increased flexibility and range of joint motion (Sugimoto and Blanpied, 2006; Swank et al., 2003), and enhanced gait and balance of older adults (Topp et al., 1993, 1996).

Our research group developed an elastic band exercise program for older adults in wheelchairs, called the Wheelchair-bound Senior Elastic Band (WSEB) exercise program (Chen et al., 2013). The WSEB is different from traditional elastic band exercises in the following ways. First, the WSEB program accommodates the reduced muscle strength and body flexibility experienced by many older adults and is less strenuous. Second, the thickness of the elastic belt is chosen as medium to allow the level of resistance training to be flexibly increased or decreased to accommodate the muscle strength of older adults. Third, the WSEB program accommodates the special characteristics of the wheelchair, and some exercises are executed using the wheelchair's handrails.

The WSEB program has been critically reviewed by a panel of 12 experts and further pilot-tested in a group of 10 older adults in wheelchairs for four weeks (Chen et al., 2013). Positive feedback was reported by the participants after four weeks of pilot-testing, which included having more muscle strength in their hands and legs, increased body flexibility and range of joint motion, and feelings of being more energetic (Chen et al., 2013). However, this positive feedback was qualitative and no objective formal testing was conducted to verify these findings. Therefore, this study aimed to test the effectiveness of six months of WSEB exercises on the functional fitness of nursing home older adults in wheelchairs using objective measurements.

2. Materials and methods

2.1. Design

A cluster randomized trial was used. Ten nursing homes were randomly assigned to either the experimental or control group using a black box drawing method (Polit and Beck, 2012), and the participants were randomly assigned

to the two groups based on the nursing home where they lived. A cluster randomization, instead of individual randomization, was adopted to reduce possible contamination among participants if one nursing home had both experimental and control groups participants. The functional fitness of the participants was examined at three points in time: baseline, after three months, and at the end of the six months study.

2.2. Setting & participants

The study was conducted in 10 nursing homes in southern Taiwan. Inclusion criteria were: (1) older adults aged 65 years and over, (2) using wheelchairs for mobility, (3) living in the facility for at least three months, (4) cognitively intact (a score of eight or higher on the Short Portable Mental Status Questionnaire) (Pfeiffer, 1975), and (5) heavily or moderate dependency in their activities of daily living (a score of 21–90 on the Barthel index). Exclusion criteria were: (1) having severe or acute cardiovascular, musculoskeletal, or pulmonary illnesses, or (2) suffering from a spinal cord injury with no rehabilitation potential. Based on the statistical software Sample Power 2.0, the required sample size was 56 participants for each group (power = 0.8; alpha = 0.05; R-square of covariate in medium level 0.13; effect size in medium level 0.25).

Following approval by the Institutional Review Board of the university hospital and the agency administrators, a convenience sample of 127 participants was recruited and randomly assigned to two groups based on the nursing homes where they lived: five nursing homes in the experimental group ($n = 64$) and five nursing homes in the control group ($n = 63$). A total of 115 participants completed the study (retention rate: 90.55%): nine participants withdrew by the end of the three-month interval (experimental group $n = 3$; control group $n = 6$) and three more participants withdrew by the end of the six-month interval (experimental group $n = 1$; control group $n = 2$). The reasons for withdrawal included: deceased (experimental group $n = 3$; control group $n = 3$) and discharged from nursing homes (experimental group $n = 1$; control group $n = 5$). To ensure the consistency of the intervention received by the participants, one experimental group participant with an attendance rate of less than 50% was excluded from the data analysis ($N = 114$; experimental group $n = 59$; control group $n = 55$) (Fig. 1). The characteristics of the participants who withdrew from the study were similar to those participants who remained in the study.

2.3. Intervention

The WSEB program has two levels: the basic and the advanced. The basic WSEB program has three phases (warm-up, aerobic motion, and harmonic stretching), and each phase has four elastic band exercises. Two relatively challenging exercises were added in each phase of the basic WSEB program to form the advanced WSEB program. Thus, the advanced WSEB program also has three phases but each phase has six elastic band exercises (Chen et al., 2013). Both the basic and the advanced WSEB program have a 5-min break right after the aerobic motion phase, and take 40 min to complete. The basic level was taught and practiced for the first three months followed by the advanced level for another three months (Chen et al., 2013). The experimental group participants received the WSEB exercises led by two certified instructors who completed a nine-hour training program, three times per week, 40 min per practice for six months. To make this exercise program as a part of regular activities in the facilities, the instructors were staff or volunteers from the facilities. To ensure intervention consistency across the intervention groups and inter-rater reliability among the instructors, a pre-recorded CD made by the principal investigator verbally guided the intervention process while the instructors demonstrated and led the elastic band exercises. Any signs or symptoms of discomfort that occurred during exercises were required to be recorded by the instructors; no special concerns occurred. The attendance rate of the participants was 94.51%. The control group participants were instructed to follow their usual daily activities, which excluded any types of elastic band exercises.

2.4. Data collection

Data were collected from March to September of 2012. Functional fitness included activities of daily living (ADL), lung capacity, body flexibilities, and muscle power and endurance.

2.4.1. Activities of daily living (ADL)

The Barthel index (BI) was used to measure the dependency level of the participants' ADL functioning. It includes seven items (feeding, grooming, toileting, bathing, dressing, bowel control, and bladder control) to assess self-care ability, and three items (transfers, mobility, and climbing stairs) to detect activity capacity. The BI scores range from 0 to 100: 0–20 indicates totally dependent; 21–60 means heavily dependent; 61–90 conveys moderate dependent; 91–99 means mild dependent; 100 indicates total independence in functioning (Liu, 2007). A Cronbach's alpha of 0.88 was obtained from this sample.

2.4.2. Lung capacity

It was measured by the TruZone^{TM} Peak Flow Meter (Trudell Medical International, Ontario, Canada: range 60–800 L) and recorded in litres. Participants were asked to take a deep breath and blow air into the meter as fast as they could.

2.4.3. Body flexibilities

It included upper body flexibility, lower body flexibility, and range of joint motion. Upper body flexibility was measured by the back scratch test (Rikli and Jones, 2001), in which the participants stood and placed their preferred hand over the same shoulder, palm down and fingers extended, reaching down the middle of the back as far as possible. At the same time, the participants placed the other arm around the back of the waist with the palm up, reaching up the middle of the back as far as possible in an attempt to touch or overlap the extended middle fingers of both hands. The distance between the extended middle

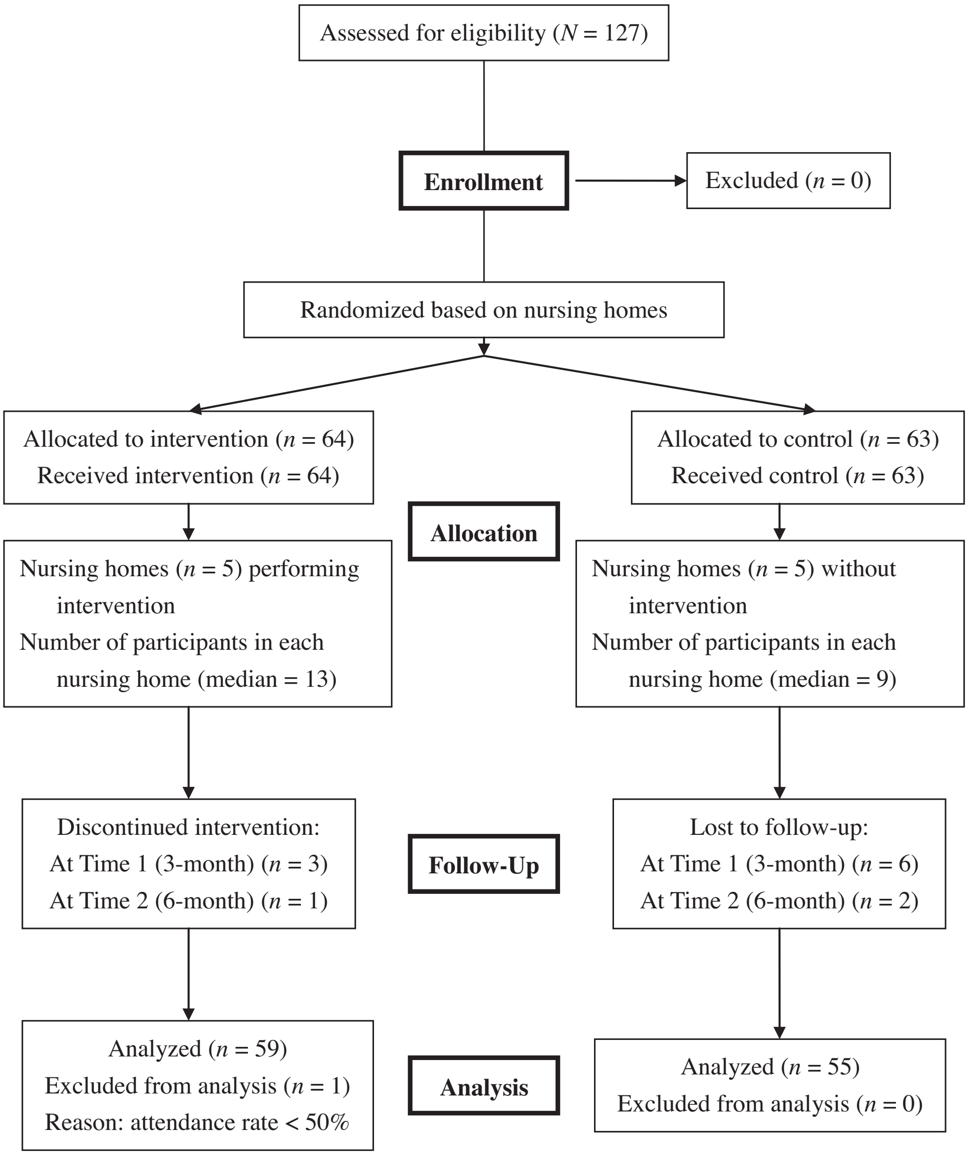

Fig. 1. Flow of the participants.

fingers of both hands was measured in centimeters: if the middle fingers just barely touched, a zero score was recorded; the distance of overlapping was recorded as a plus (+) score; the distance between the tips of the middle fingers was recorded as a minus (−) score (Rikli and Jones, 2001). Lower body flexibility was measured by the chair sit-and-reach test (Rikli and Jones, 2001). The participants were asked to sit on the edge of a chair. One leg was bent with the foot flat on the floor, and the other leg was extended as straight as possible in front of the hip with the heel placed on the floor and with the foot flexed at approximately 90 degrees. Participants were asked to stretch out the arms, hands overlapping, and slowly bend forward at the hip joint reaching as far forward as possible toward or past the toes. The distance from the tips of the middle fingers to the top of the toes was measured in centimeters: the midpoint at the top of the toes represented the zero point; if the reach was short of this point, the distance was recorded as a minus (−) score; if the reach was past the midpoint of the toes, the distance was recorded as a plus (+) score (Rikli and Jones, 2001). Range of joint motion included flexion and abduction of the shoulder joint on the dominant side. Goniometry was used to measure the degree of flexion and abduction.

2.4.4. Muscle power and endurance

Muscle power and endurance included hand-grip strength, upper limbs muscle endurance, and lower limbs muscle endurance. Hand-grip strength was measured using a digital handgrip dynamometer (Model TKK-5401 GRIP-D: Takei Scientific Instruments Co., Ltd, Japan: range 5–100 kg) with the dominant hand, measuring twice, and the best performance was recorded in kilograms. Upper limbs muscle endurance was measured by the arm curl

test (Rikli and Jones, 2001). Participants sat on a chair with the back straight and feet flat on the floor and with the dominant side of the body close to the edge of the seat. A dumbbell weight of eight pounds for men or five pounds for women was held down at the side, perpendicular to the floor, in the dominant hand with a handshake grip. From the down position, the weight was curled up with the palm gradually rotating to a facing-up position during flexion. The weight was then returned to the fully extended down position with the handshake grip. The score was recorded as the total number of arm curls executed in 30 s (Rikli and Jones, 2001). The chair-stand test was used to evaluate lower limbs muscle endurance; participants were asked to stand up and sit down, with arms folded across their chest, using an armless chair. The number of chair stands executed in 30 s was recorded.

All of the measurement devices were frequently used in clinical settings with high expert content validity and were used in previous studies with good psychometric properties (Chen et al., 2008). Two sequential trial tests were employed for each measurement device and their intraclass correlations were all above 0.80. Further, the physical measurement devices were maintained by the project manager and calibrated for their functions before each data collection to minimize errors.

2.5. Data analysis

The Statistical Products and Services Solutions (SPSS) Version 19.0 was used to analyze the data. Descriptive statistics such as mean, standard deviation, range, and frequency distribution were used to describe the demographic profiles of the participants. The Pearson χ^2 test or the independent t-test was used to test group differences in the demographic profiles and the pre-test data. The mixed-design, two-way analysis of variance (ANOVA) was used to detect the variables in which time and group had interaction effects. For those variables with significant interaction effects, one-way repeated measures ANOVAs were performed to analyze the simple main effect of different time points in each group. To further understand the group differences after three months and at the end of the six months study, analysis of covariance (ANCOVA) was computed on those variables with significant interaction effects using the pre-test data as the covariate to offset the group differences at the beginning of the study. No missing data occurred in this study since the physical measurements of participants' functional fitness were measured individually, and as per protocol analysis was conducted. Data entry and data analysis were not blinded since different coding system was assigned to difference group participants (e.g., E for the experimental group and C for the control group). However, the person who managed the data did not collect the data on the field.

3. Results

3.1. Participants' demographic profiles

A total of 114 participants completed the study (experimental group $n = 59$, control group $n = 55$). The mean age (SD) of the participants was 79.15 (7.03) years: young-old (65–74 years old; 26.30%), middle-old (75–84 years old; 52.60%), and old-old (85–99 years old; 21.10%). These three age subgroups were classified based on Tanner et al. (2010). The gender of the participants was quite evenly represented: 50.90% male and 49.10% female. The majority of participants was widowed (57.90%), had religious beliefs (93.90%), and had a six-year elementary school education (40.40%) or no education (34.20%). The cognitive function of the participants was intact [Short Portable Mental Status Questionnaire mean score = 9.07 (1.16)]. Almost all of the participants (98.20%) had chronic illnesses with an average number (SD) of 2.11 (1.03). The top five chronic illnesses reported were: (1) hypertension (64.90%), (2) stroke (43.00%), (3) diabetes (30.70%), (4) heart disease (27.20%), and (5) chronic obstructive pulmonary disease (COPD) (10.50%). The demographic profiles of the participants in the two groups had no significant differences (all $p > 0.05$), and were similar to the nursing home older adults in general.

3.2. Baseline comparisons between the two groups

Data were normally distributed, and results of the independent t tests indicated that participants in the experimental group had better mean (SD) upper body flexibility [−25.23 (15.16) cm] than the control group [−32.26 (13.38) cm] at baseline ($t = 2.62$, $p = 0.010$). The mean (SD) upper limbs muscle endurance of the experimental group participants [7.44 (4.95) times] was also better than the control group [5.35 (4.85) times] ($t = 2.28$, $p = 0.024$). No significant differences were found in the remaining variables (all $p > 0.05$) at baseline.

3.3. Interaction effects between time points and groups

Results of a mix-design, two-way ANOVA indicated that there were significant interaction effects between three time points and two different groups in all of the variables (all $p < 0.05$).

3.4. Changes among three time points in each group

Results indicated that all of the functional fitness indicators improved significantly in the experimental group (all $p < 0.05$), except the ADL ($F = 1.00$, $p = 0.371$) (Table 1). On the other hand, the ADL ($F = 5.51$, $p = 0.014$), lung capacity ($F = 4.01$, $p = 0.032$), and lower body flexibility ($F = 16.49$, $p < 0.001$) of the participants in the control group had deteriorated significantly (Table 2).

3.5. Group comparisons at different time points

Results indicated that the experimental group had better ADL, lung capacity, lower body flexibility, shoulder flexion and abduction, hand grip strength, and upper and lower limbs muscle endurance than the control group after the three months study (all $p < 0.05$). Although the upper body flexibility had no significant differences between the two groups at three months ($F = 3.11$, $p = 0.081$), the adjusted mean score of the experimental

Table 1
Changes among pre-test and post-tests in the experimental group ($n = 59$).

Variables	Pre-test		Post-test I		Post-test II		*F* (*p*)	Post hoc[a]
	M	*SD*	*M*	*SD*	*M*	*SD*		
ADL[b]	57.54	24.31	58.39	25.40	56.95	26.65	1.00 (0.371)	–
Lung capacity (L)	147.80	82.27	166.53	87.04	178.81	88.37	13.51 (<0.001)***	Pre < I; Pre < II
Upper body flexibility (cm)	−25.23	15.16	−22.83	14.96	−21.08	14.42	26.01 (<0.001)***	Pre < I < II[c]
Lower body flexibility (cm)	−17.91	12.94	−15.40	13.15	−13.32	13.14	16.16 (<0.001)***	Pre < I < II
Shoulder flexion (°)	140.47	20.61	143.02	17.53	146.46	16.95	8.95 (0.002)**	Pre < II; I < II
Shoulder abduction (°)	140.00	22.13	143.90	22.41	148.39	20.58	14.96 (<0.001)***	Pre < I < II
Hand grip strength (kg)	13.56	5.84	14.27	6.53	14.64	6.43	6.12 (0.004)**	Pre < II
Upper limbs muscle endurance (times/30 s)	7.44	4.95	9.10	5.00	9.73	5.05	19.70 (<0.001)***	Pre < I < II
Lower limbs muscle endurance (times/30 s)	1.53	3.04	2.64	4.11	2.58	4.18	10.14 (<0.001)***	Pre < I; Pre < II

Note: One-way repeated measures ANOVAs were performed to analyze the simple main effect of different time points in the experimental group.
–: post hoc analysis was not performed due to non-significant *F* value.
** $p < 0.01$.
*** $p < 0.001$.
[a] Bonferroni post hoc test (adjusting α levels of multiple tests).
[b] Activities of daily living (measured by the Barthel index).
[c] Pre = pre-test; I = post-test I; II = post-test II.

group is better than the control group (Table 3). By the end of the six months study, significant differences occurred in all of the variables between the two groups (all $p < 0.05$) (Table 4); the experimental group results were all better than the control group.

4. Discussions

Results indicated that all of the functional fitness indicators improved significantly in the experimental group, except the ADL. However, the ADL, lung capacity, and lower body flexibility of the control group participants deteriorated significantly. As reported by Shore (2008), 12.3% of older adults in wheelchairs experienced worsening health. Without proper activities or exercises, wheelchair users might experience further disability and mortality (Hirvensalo et al., 2000). There were two possible explanations as to why the ADL of the experimental group participants had no significant improvement. First, the ADL deterioration that occurred in the control group participants might possibly happen to the experimental group participants and compensate for the significant improvement of the ADL in the experimental group. Second, the ADL is a dynamic and complex function and may require longer training in order to see improvement.

Further, the experimental group participants had better ADL, lung capacity, lower body flexibility, shoulder flexion and abduction, hand grip strength, and upper and lower limbs muscle endurance than the control group after three months of the study and maintained throughout the six months of the study. The improvement of shoulder joint range of motion was similar to Sugimoto and Blanpied (2006) and Swank et al. (2003) studies. The enhancement of lower limbs muscle endurance was congruent with the studies of Binder et al. (2005), Chen et al. (2009), and

Table 2
Changes among pre-test and post-tests in the control group ($n = 55$).

Variables	Pre-test		Post-test I		Post-test II		*F* (*p*)	Post hoc[a]
	M	*SD*	*M*	*SD*	*M*	*SD*		
ADL[b]	51.45	23.11	48.73	22.88	46.36	23.54	5.51 (0.014)*	Pre > II
Lung capacity (L)	156.18	89.53	157.36	82.12	147.36	80.63	4.01 (0.032)*	I > II
Upper body flexibility (cm)	−32.26	13.38	−30.82	12.13	−31.13	12.09	2.36 (0.127)	–
Lower body flexibility (cm)	−13.93	13.31	−15.36	13.41	−16.71	13.56	16.49 (<0.001)***	Pre > I > II[c]
Shoulder flexion (°)	139.09	23.15	137.11	21.47	136.09	19.43	3.06 (0.059)	–
Shoulder abduction (°)	138.76	25.43	138.15	21.78	137.00	20.91	1.06 (0.328)	–
Hand grip strength (kg)	11.99	5.94	11.70	5.82	11.57	5.96	1.12 (0.314)	–
Upper limbs muscle endurance (times/30 s)	5.35	4.85	5.18	4.65	4.82	4.32	2.42 (0.113)	–
Lower limbs muscle endurance (times/30 s)	0.89	2.37	0.91	2.37	0.87	2.23	0.05 (0.857)	–

Note: One-way repeated measures ANOVAs were performed to analyze the simple main effect of different time points in the control group.
–: post hoc analysis was not performed due to non-significant *F* value.
* $p < 0.05$
*** $p < 0.001$.
[a] Bonferroni post hoc test (adjusting α levels of multiple tests).
[b] Activities of daily living (measured by the Barthel index).
[c] Pre = pre-test; I = post-test I; II = post-test II.

Table 3
Group differences after three months of the study ($N = 114$).

Variables	Adjusted *M*		*SS*	*df*	*MS*	*F* (*p*)
ADL[a]	E	55.56	401.69	1	401.69	6.44 (0.013)*
	C	51.77				
Lung capacity (L)	E	170.15	7902.50	1	7902.50	6.28 (0.014)*
	C	153.47				
Upper body flexibility (cm)	E	−25.81	87.65	1	87.65	3.11 (0.081)
	C	−27.62				
Lower body flexibility (cm)	E	−13.59	383.84	1	383.84	16.20 (<0.001)***
	C	−17.30				
Shoulder flexion (°)	E	142.51	669.48	1	669.48	6.45 (0.012)*
	C	137.66				
Shoulder abduction (°)	E	143.40	635.02	1	635.02	6.44 (0.013)*
	C	138.68				
Hand grip strength (kg)	E	13.53	30.16	1	30.16	5.17 (0.025)*
	C	12.49				
Upper limbs muscle	E	8.25	126.86	1	126.86	19.61 (<0.001)***
endurance (times/30 s)	C	6.09				
Lower limbs muscle	E	2.33	32.60	1	32.60	9.57 (0.002)**
endurance (times/30 s)	C	1.25				

Note: Analysis of covariance (ANCOVA) was computed to test the group differences after three months of the study.
E = experimental group; C = control group.
* $p < 0.05$.
** $p < 0.01$.
*** $p < 0.001$.
[a] Activities of daily living (measured by the Barthel index).

Dancewicz et al. (2003). This study confirmed that muscles of older adults are as trainable as those of younger adults (Brown et al., 1990), and elastic band exercises are feasible and helpful for frail older adults (Dancewicz et al., 2003; Topp et al., 2002). Even for older adults in wheelchairs, there is the potential to promote their health. This particular group of participants was rather old in age, less educated, and had multiple morbidities. Nearly 74% of the participants were over the age of 75 years, 74.6% of them had less than six-year elementary school education, and 98.2% had at least one chronic illness. Even with the multiple chronic illnesses of hypertension, stroke, diabetes, heart disease, and COPD, this group of older adults were still capable of learning, following, and doing the WSEB exercises. The participants enjoyed the WSEB exercise program as evidenced by the high attendance rate of 94.51%. The findings of the objective measurements in this study confirmed the qualitative positive feedback reported by the participants in the pilot-testing (Chen et al., 2013).

Table 4
Group differences at the end of six-month of the study ($N = 114$).

Variables	Adjusted *M*		*SS*	*df*	*MS*	*F* (*p*)
ADL[a]	E	54.19	665.17	1	665.17	4.74 (0.032)*
	C	49.32				
Lung capacity (L)	E	182.27	42356.83	1	42356.83	23.48 (<0.001)***
	C	143.65				
Upper body flexibility (cm)	E	−23.98	436.57	1	436.57	15.67 (<0.001)***
	C	−28.01				
Lower body flexibility (cm)	E	−11.60	1349.46	1	1349.46	34.37 (<0.001)***
	C	−18.56				
Shoulder flexion (°)	E	145.98	2505.08	1	2505.08	27.45 (<0.001)***
	C	136.60				
Shoulder abduction (°)	E	147.95	3118.62	1	3118.62	26.27 (<0.001)***
	C	137.48				
Hand grip strength (kg)	E	13.91	67.26	1	67.26	9.92 (0.002)**
	C	12.36				
Upper limbs muscle	E	8.94	293.17	1	293.17	37.76 (<0.001)***
endurance (times/30 s)	C	5.66				
Lower limbs muscle	E	2.27	31.72	1	31.72	8.27 (0.005)**
endurance (times/30 s)	C	1.20				

Note: Analysis of covariance (ANCOVA) was computed to test the group differences at the end of the six months study.
E = experimental group; C = control group.
* $p < 0.05$.
** $p < 0.01$.
*** $p < 0.001$.
[a] Activities of daily living (measured by the Barthel index).

Finally, although the upper body flexibility had no significant differences between the two groups at the end of three months of the study, the adjusted mean score of the experimental group is better than the control group, and by the end of six months of the study, a significant difference was observed. The upper body flexibility was measured by the back scratch test, which was an important functional ability for participants to complete daily activities, such as combing hair, changing clothes, scrubbing the back while showering or bathing. The WSEB exercise program specifically incorporated several exercises to train this functional ability, such as pulling the arms, raising hand, and demonstrating an action similar to whipping out a sword (Chen et al., 2013). Therefore, the upper body flexibility of the experimental group participants was better than the control group participants after a longer training using the WSEB exercises.

4.1. Study limitations

First, convenience sampling, rather than a probability sampling, was used. Although the method of random assignment by study sites into experimental or control group was applied, without random sampling, it was possible that some older adults who met the sample selection criteria but represented significant differences from the sample studied were not recruited since they were not in the study sites. Moreover, a cluster randomization for the group assignment might undermine the individual differences, and the clustering effect might cause the baseline differences between two groups, reduce its statistical power, and diminish the reliability of the results. A truly randomized control trial with participants individually randomized as to intervention or control group might provide more robust data. Second, although the sample size of the two groups in the initial recruitment (experimental $n = 64$, control $n = 63$) were nearly equal, possible unequal cluster size might exist and could weaken the reliability of the results. The clustering should be considered in the sample power calculation in future studies. Third, the interventions were carried out at the lobbies of the nursing homes. The lobbies of some nursing homes were located right next to the entrance, which may have distracted the participants and interfered with the intervention. A wide and ventilated setting is recommended for the WSEB group exercises. Finally, this study was conducted only for six months. The lasting effects or the plateau of the WSEB program were unknown. A longer intervention period of one to two years would be needed to see the trend of functional fitness changes after WSEB exercises.

5. Conclusions

This study concludes that regular practice of WSEB exercises significantly improved the functional fitness of the nursing home older adults in wheelchairs. The WSEB exercise program could be incorporated as a part of daily activities for nursing home older adults in wheelchairs. Nursing home directors could recruit volunteers to be trained as certified instructors of the WSEB exercises, and lead the nursing home older adults using wheelchairs to practice the WSEB exercises in groups regularly in the facilities. It is essential that more exercise-based activities with evidence-based outcomes be incorporated into long-term care facilities to enhance the physical fitness of older adults and to document their progression of health promotion.

Conflict of interest: No conflict of interest has been declared by the authors.

Funding: This study was funded by the National Science Council, Taiwan (NSC99-2628-B-037-066-MY3). The funding source supported the study financially and had no involvement in the study design, data collection, analysis and interpretation of data, writing of the report, and decision to submit the paper for publication.

Contributors: All authors meet the criteria for authorship, have approved the final article and that all those entitled to authorship are listed as authors.

Ethical Approval: This study was approved by the Institutional Review Board of Fooyin University Hospital (FYH-IRB-098-12-02).

Acknowledgements

Sincere appreciation is directed by our group to the National Science Council, Taiwan for funding this study (NSC99-2628-B-037-066-MY3), to Professor Frank Belcastro for his superlative manuscript editing, to the directors and staff of 10 nursing homes for their support and assistance, and to the 127 wonderful older adults for their generous participation.

References

Binder, E.F., Yarasheski, K.E., Steger-May, K., Sinacore, D.R., Brown, M., Schechtman, K.B., Holloszy, J.O., 2005. Effects of progressive resistance training on body composition in frail older adults: results of a randomized, controlled trial. J. Gerontol.: Med. Sci. 60A, 1425–1431.

Brown, A.B., McCartney, N., Sale, D.S., 1990. Positive adaptations to weight-lifting training in the elderly. J. Appl. Physiol. 69, 1725–1733.

Chen, K.M., Chen, M.S., Hong, S.M., Chao, H.C., Lin, H.S., Li, C.H., 2008. Physical fitness of older adults in senior activity centers after 24-week silver yoga exercises. J. Clin. Nurs. 17, 2634–2646.

Chen, K.M., Tseng, W.S., Chang, Y.H., Huang, H.T., Li, C.H., 2013. Feasibility appraisal of an elastic band exercise program for older adults in wheelchairs. Geriatr. Nur. (Lond.) 34, 373–376.

Chen, T.A., Wu, Y.T., Lee, M.B., Liang, K.C., Lin, K.N., Tsai, M.W., 2009. Effects of exercise on depression symptoms, physical function, and quality of life in community-dwelling elderly. Formos. J. Phys. Ther. 34, 209–218.

Damush, T.M., Damush, J.G., 1999. The effects of strength training on strength and health-related quality of life in older adult women. The Gerontologist 39, 705–710.

Dancewicz, T.M., Krebs, D.E., McGibbon, C.A., 2003. Lower-limb extensor power and lifting characteristics in disabled elders. J. Rehabil. Res. Dev. 40, 337–348.

Galvao, D.A., Taaffe, D.R., 2005. Resistance exercise dosage in older adults: single versus multiset effects on physical performance and body composition. J. Am. Geriatr. Soc. 53, 2090–2097.

Hirvensalo, M., Rantanen, T., Heikkinen, E., 2000. Mobility difficulties and physical activity as predictors of mortality and loss of independence in the community-living older population. J. Am. Geriatr. Soc. 48, 493–498.

Karmarkar, A.M., Dicianno, B.E., Cooper, R., Collins, D.M., Matthews, J.T., Koontz, A., Teodorski, E.E., Cooper, R.A., 2011. Demographic profile of older adults using wheeled mobility devices. J. Aging Res. 2011, 1–11, http://dx.doi.org/10.4061/2011/560358.

Kaye, S.H., Kang, T., LaPlante, M.P., 2002. Wheelchair use in the United States. Disability Statistics Center, San Francisco.

Liu, W.M., 2007. Assessment for older adults. In: Kao, S.F. (Ed.), Gerontological nursing. 2nd ed. Yeong Dah, Taipei, Taiwan, pp. 8-1–8-21.

Patterson, R.M., Jansen, C.W.S., Hogan, H.A., Nassif, M.D., 2001. Material properties of thera-band tubing. Phys. Ther. 81, 1437–1445.

Polit, D.F., Beck, C.T., 2012. Nursing research: generating and assessing evidence for nursing practice, 9th ed. Lippincott Williams & Wilkins, Philadelphia, USA.

Pfeiffer, E., 1975. A short portable mental status questionnaire for the assessment of organic brain deficit in elderly patients. J. Am. Geriatr. Soc. 23, 433–441.

Rikli, R.E., Jones, C.J., 2001. Senior fitness test manual. Human Kinetics, Champaign, IL.

Rimmer, J.H., 2005. Exercise and physical activity in persons aging with a physical disability. Phys. Med. Rehabil. Clin. N. Am. 16, 41–56.

Shore, S.L., 2008. Use of an economical wheelchair in India and Peru: impact on health and function. Med. Sci. Monit. 14 (12), PH71–PH79.

Sugimoto, D., Blanpied, P., 2006. Flexible foil exercise and shoulder internal and external rotation strength. J. Athl. Train. 41, 280–285.

Swank, A.M., Funk, D.C., Durham, M.P., Roberts, S., 2003. Adding weights to stretching exercise increases passive range of motion for healthy elderly. J. Strength Cond. Res. 17, 374–378.

Tanner, D.A., Kloseck, M., Crilly, R.G., Chesworth, B., Gilliland, J., 2010. Hip fracture types in men and women change differently with age. BMC Geriatr. 10, 12–14, http://dx.doi.org/10.1186/1471-2318-10-12.

Topp, R., Boardley, D., Morgan, A.L., Fahlman, M., McNevin, N., 2005. Exercise and functional tasks among adults who are functionally limited. West. J. Nurs. Res. 27, 252–270.

Topp, R., Mikesky, A., Bawel, K., 1994. Developing a strength training program for older adults: planning, programming, and potential outcomes. Rehabil. Nurs. 19, 266–297.

Topp, R., Mikesky, A., Dayhoff, N.E., Holt, W., 1996. Effect of resistance training on strength, postural control, and gait velocity among older adults. Clin. Nurs. Res. 5, 407–427.

Topp, R., Mikesky, A., Wigglesworth, J., Holt, W., Edwards, J.E., 1993. The effect of a 12-week dynamic resistance strength training program on gait velocity and balance of older adults. The Gerontologist 33, 501–506.

Topp, R., Woolley, S., Hornyak, J., Khuder, S., Kahaleh, B., 2002. The effect of dynamic versus isometric resistance training on pain and functioning among adults with osteoarthritis of the knee. Arch. Phys. Med. Rehabil. 83, 1187–1195.

Yang, H.C., Cheng, T.Y., Ling, S.H., Fang, C.L., Ting, T.H., 2011. Utilization of outpatient rehabilitation for disabled elderly stroke patients in different care settings and its implications for long-term care policy. J. Health Manage. 9, 1–16.
