# Supplementary material for: Effects of elastic band resistance training on the physical and mental health of elderly individuals: A mixed methods systematic review
Source: PLoS One. 2024 May 13;19(5):e0303372. doi: 10.1371/journal.pone.0303372 (PMC11090353; doi:10.1371/journal.pone.0303372)
Supplement: S1 File — (ZIP) [file pone.0303372.s001.zip › Supporting Information/Included study 43.pdf]

Experimental Gerontology 65 (2015) 16–22

Contents lists available at ScienceDirect

Experimental Gerontology

journal homepage: www.elsevier.com/locate/expgero

The effect of six months of elastic band resistance training, nutritional supplementation or cognitive training on chromosomal damage in institutionalized elderly

Bernhard Franzke [a], Barbara Halper [a], Marlene Hofmann [b], Stefan Oesen [a], Béatrice Pierson [c], Ariane Cremer [c], Evelyn Bacher [c], Birgit Fuchs [c], Andreas Baierl [d], Anela Tosevska [a], Eva-Maria Strasser [e], Barbara Wessner [a,b], Karl-Heinz Wagner [a,c,*], Vienna Active Ageing Study Group (VAAS)

[a] University of Vienna, Research Platform Active Ageing, Althanstraße 14, 1090 Vienna, Austria
[b] University of Vienna, Centre for Sport Science and University Sports, Department of Sport and Exercise Physiology, Auf der Schmelz 6, 1150 Vienna, Austria
[c] University of Vienna, Faculty of Life Sciences, Department of Nutritional Sciences, Althanstraße 14, 1090 Vienna, Austria
[d] University of Vienna, Institute for Statistics and Operations Research, Grillparzerstraße 7, 1010 Vienna, Austria
[e] Karl Landsteiner Institute for Remobilization and Functional Health/Institute for Physical Medicine and Rehabilitation, Kaiser Franz Joseph Spital, SMZ-Süd, Kundratstraße 3, 1100 Vienna, Austria

ARTICLE INFO

Article history:
Received 7 November 2014
Received in revised form 16 January 2015
Accepted 3 March 2015
Available online 5 March 2015

Section Editor: Holly M Brown-Borg

Keywords:
Chromosomal damage
DNA damage
Exercise
Training
Aging
VAAS

ABSTRACT

Increased DNA and chromosomal damage are linked to aging and age-related diseases like cardiovascular diseases, diabetes or cancer. Physical activity and an optimal status of micro- and macronutrients are known to reduce the incidence of MN, a marker for chromosomal instability and mutagenicity. Once older people reach a certain age they change from a home-living situation to an institutionalized situation, which is often accompanied by malnutrition, depression and inactivity. We conducted the current study to investigate the effect of a six month progressive resistance training (RT), with or without protein and vitamin supplementation (RTS) or cognitive training (CT) only, on chromosomal damage measured by the cytokinesis block micronucleus cytome assay in 97 Austrian institutionalized women and men (65–98 years). All three intervention groups demonstrated a tendency of a reduced frequency of cells with MN (−15%) as well as for the total number of MN (−20%), however no significant time-effect was observed. Besides a significant increase in plasma B12 and red blood cell folate status, the six month change of B12 was negatively correlated with the six month change of the MN frequency in the RTS group ($r = -0.584$, $p = 0.009$). Our results suggest that in this age group either physical or cognitive training may result in similar biochemical changes and therefore enhance resistance against genomic instability. Supplementation with the vitamins B12 and folic acid could contribute to reduced chromosomal damage in institutionalized elderly.

© 2015 Elsevier Inc. All rights reserved.

1. Introduction

The loss of muscle mass, strength, function and the associated reduction of life quality is linked to the process of aging (Johnston et al., 2008; Seene et al., 2012). Physical activity, regular exercise, and especially resistance training are important in preventing severe muscle atrophy, enhancing function and maintaining physical independence in the elderly (Demontis et al., 2013; Arnold and Bautmans, 2014). The process of aging and the loss of muscle mass are both associated with higher levels of DNA damage and deteriorated antioxidant defense (Fulle et al., 2004; Gianni et al., 2004). It is well known that there are increased levels of chromosomal and DNA damage in people with chronic diseases such as cardiovascular diseases, type 2 diabetes and cancer (Battershill et al., 2008; Müllner et al., 2014). Resistance training, together with protein supplementation, seems to be most effective for increasing muscle mass and strength in the elderly (Shahar et al., 2013; Lustgarten et al., 2014). To lower the frequency of micronuclei (MN), which is a well-established marker for chromosomal damage and highly correlated with cellular mutation, an adequate status of the vitamins B12 and folate is recommended (Fenech and Bonassi, 2011; Ni et al., 2012).

Studies concerning the effect of exercise and training on MN frequency are rare. However, examinations on that topic show varying results. Regular physical activity seems to lower chromosomal damage (Goon et al., 2008; Huang et al., 2009), whereas acute exhausting exercise tends to increase the MN frequency (Schiffl et al., 1997). Training status also influences the body's ability to deal with exercise-induced oxidative stress. After an ironman triathlon, trained individuals showed no increase in chromosomal damage or even a reduced MN frequency

* Corresponding author at: Department of Nutritional Sciences, University of Vienna, Althanstraße 14, A-1090 Vienna, Austria.
E-mail address: karl-heinz.wagner@univie.ac.at (K.-H. Wagner).

http://dx.doi.org/10.1016/j.exger.2015.03.001
0531-5565/© 2015 Elsevier Inc. All rights reserved.

(Reichhold et al., 2008), whereas only untrained subjects demonstrated an increased mutation rate even after 30 min of intense running (Umegaki et al., 1998).

As life expectancy is still growing, it is of great need to investigate health- and fitness-related markers in the oldest of the population (8th, 9th and 10th decade). Currently there is a general lack of data for this age group. This aspect is of particular interest since the living situation of many elderly individuals is changing in this period towards a transfer to new surroundings such as elderly homes, which is often accompanied by conditions of malnutrition, multiple medications or increased depression and loss of life quality (Smoliner et al., 2009).

We conducted the current study to investigate the effect of a six month progressive resistance training (using elastic bands), with or without protein and vitamin supplementation, on chromosomal damage in Austrian institutionalized elderly. The cytokinesis block micronucleus cytome assay (CBMN-assay) was performed to measure genome integrity. In the present study we hypothesized that increased physical fitness improves resistance against chromosomal instability in institutionalized elderly. Protein and vitamin supplementation should enhance this protective effect. With our study cohort subjects, being at or above life expectancy in Austria (study/Austria: women 82.86/83.25 years; men 84.85/77.95 years) (StatisticsAustria, 2014) and 75% of our study population being 80 years old or older, we were able to generate novel data on chromosomal damage and physical performance after a six month training period in this particular age group of institutionalized elderly.

2. Methods

2.1. Subjects

Ninety-seven institutionalized elderly women and men (aged 65–98 years), recruited from five different senior residencies in the area of Vienna (Curatorship of Viennese Retirement Homes), volunteered for the study. The subjects were mentally (Mini Mental State Examination ≥23) and physically (Short Physical Performance Battery >4) able to participate in the study. They were sedentary (less than 1 h of physical activity or exercise per week) and free of severe diseases that would contra-indicate medical training therapy or measurement of physical performance, including cardiovascular diseases, diabetic retinopathy and regular use of cortisone-containing drugs. The health condition of all study subjects was assessed by specialists in internal medicine and gerontology. Furthermore, regular strength training (more than once per week) in the last six months before inclusion was an exclusion criterion. Written informed consent was obtained from all participants before entry into the study in accordance with the Declaration of Helsinki. This study was approved by the ethics committee of the City of Vienna (EK-11-151-0811) and registered at ClinicalTrials.gov, NCT01775111.

2.2. Study design

The present study was conducted in a randomized, controlled, observer-blind design. The participants were randomly divided into three parallel groups – cognitive training (CT), resistance training (RT), RT + supplement (RTS) – and matched for gender. Blood samples were taken and physical performance tests were executed before (T1), after three months (T2) and after six months (T3) of intervention. The goal was to assess and compare the effects of either a resistance training intervention, a resistance training and nutritional supplementation intervention or a cognitive training intervention on institutionalized elderly.

2.3. Resistance training

The resistance training groups (RT and RTS) received two weekly sessions of resistance training, conducted on two non-consecutive days and supervised by a sport scientist. Training attendance was recorded every session. The only equipment used were elastic bands and a chair. The progressive resistance training protocol was designed based on the guidelines of the American College of Sports Medicine for resistance training with older subjects (Nelson et al., 2007). The main part consisted of ten exercises for the main muscle groups (legs, back, abdomen, chest, shoulder and arms). One training session started with 10 min of warm-up, continued with 30–40 min of strength training and ended with a 10 minute cool-down. To keep the training stimulus high enough, the exercise program was adjusted to the participants' individual needs, by either adapting the resistance of the elastic band (shorter or stronger band) or by modifying the exercise, by means of performing a more difficult version. In the initial phase (4 weeks) one set of 15 repetitions was performed in order to learn the correct form of each exercise. From the fifth week on the intensity and volume has progressively been increased from two sets of light exercises to two sets of heavy resistance. If the participants could easily perform two sets of 15 repetitions they were told to either take more resistance or to perform a more difficult version of the exercise.

2.4. Resistance training and supplementation

The RTS group followed the same training protocol as the RT group. Additionally they received a liquid supplement every morning, as well as directly after each training session. It consisted of 20.7 g protein (56 energy (En)%, 19.7 g whey protein, 3 g leucine, >10 g essential amino acids), 9.3 g carbohydrates (25 En%, 0.8 BE), 3.0 g fat (18 En%), 1.2 g roughage (2 En%), 800 IU (20 μg) of vitamin D, 250 mg calcium, vitamins C, E, B6 and B12, folic acid and magnesium (1 portion FortiFit, Nutricia). Total energy per drink was 150 kcal. The intake of the nutritional supplement was controlled at breakfast as well as after the training sessions.

2.5. Cognitive training

The CT groups performed coordinative or cognitive tasks two times per week, equally to the RT and RTS groups. In contrast to a classic control group, which would not get any treatment, the training frequency of this group was the same as in the other groups. Therefore the “bias” of regularly being part in group activities was minimized (socialization factor). The participants of the CT group mainly performed memory training and finger dexterity exercises in sitting position (Gatterer and Croy, 2004).

2.6. Cytokinesis block micronucleus cytome assay

Blood samples were taken early morning after an overnight fast using heparin, serum and EDTA tubes (Greiner Bio-One, Kremsmunster, Austria). Peripheral blood lymphocytes were isolated using Ficoll separation tubes (Greiner Bio- One). The cytokinesis block micronucleus cytome (CBMN) assay was conducted according to the protocol of Fenech (Fenech, 2007) and Wallner et al. (Wallner et al., 2012). Cells were stimulated to perform mitotic division with phytohemagglutinin (PAA, Pasching, Austria) using a concentration of 1×10^6 cells/ml in culture medium. Samples were incubated at 37 °C and 5% CO_2, and after 44 h, cytochalasin B (Sigma Aldrich, Vienna, Austria) was added to stop further cell division. Cells were spotted onto microscope slides, stained (Diff-Quick; Medion Diagnostics, Dudingen, Switzerland) and counted using a bright field microscope (1000-fold magnification; Olympus, Wien, Austria). For each sample, duplicates were performed and two slides of each duplicate were produced. From the four resulting slides, 500 cells per slide (2000 per subject) were counted equally to minimize experimental variation and eliminate scorer bias.

To assess chromosomal damage of blood lymphocytes, the frequency of MN, nucleoplasmic bridges and nuclear buds in 2000 binucleate (BN) cells was counted, as well as the number of apoptotic and necrotic cells. Furthermore, the nuclear division index (NDI) was calculated to

measure cytostatic effects and the mitogenic response of lymphocytes (Fenech, 2007).

2.7. Chair rise test

To perform well in the chair rise test, the participants had to stand up from a chair (46 cm seat height) as often as possible within 30 s. To ensure a safe test-setting, the chair was placed against the wall. For one successful repetition, participants had to fully stand up (hip and knee fully extended) and sit back, with their arms crossed over their chest. A last-second-attempt was considered valid, if the person had covered more than 50% of the range of motion (Jones et al., 1999).

2.8. Handgrip strength test

To assess handgrip strength, participants performed an isometric handgrip strength test (kg) using a dynamometer. The test was conducted in a sitting position and maximal isometric contraction within 4–5 s was measured (JAMAR compatible handgrip dynamometer adapted to handle different sizes). The better result of two trials (one minute break in between) for each hand was noted (Mijnarends et al., 2013).

2.9. Six minute walking test

The participants had to walk for 6 min as fast and as far as possible. The 6 minute walking test is a valid tool to evaluate aerobic endurance in the elderly. Participants were allowed to slow down and even take a short rest. Every subject performed the test separately without being disturbed by others. They had to walk back and forth on a 30 meter shuttle track and the distance covered within 6 min was registered (Steffen et al., 2002).

2.10. Vitamin B12 and folate

Plasma concentrations of vitamin B12 and folate in erythrocytes were measured according to Müllner et al. (Müllner et al., 2013) by using radioimmunoassay. Standard curves were drawn and sample values calculated according to the protocol published by the kit producer (MP Biomedicals, Germany).

2.11. Statistics

Statistical analyses were performed using IBM SPSS Statistics 21. Baseline group differences were measured, using the Kruskal–Wallis-H and chi-squared tests. MN frequency was adjusted for sex as described in Bonassi et al. (Bonassi et al., 2001). To assess the overall differences between the time points, the Friedman test was conducted and if significant, the Wilcoxon test was used to calculate the differences between each time point by considering Bonferroni correction. The CBMN-assay was performed according to the protocol of Fenech (Fenech, 2007). To minimize scoring variation, all scorers have been trained on the same slides by the same persons and regularly reassessed their scoring, to keep quality on the highest level. The Spearman test was performed to assess the link between B12 plasma levels and MN frequency. A p-value of less than 0.05 was considered significant.

3. Results

3.1. Baseline characteristics

At baseline, data from 97 women and men were included into the calculations. Twenty of the former 117 participants quit the study before obtaining the first blood samples (Fig. 3). From the 97 study subjects, 13.4% were male and 86.6% female, which constitutes a representative distribution in the houses of the Curatorship of Viennese Retirement Homes. The participants had a mean-age of 83.0 ± 6.1 years (Table 1).

No significant baseline differences between the intervention groups were observed, except for the Nuclear Division Index (NDI) ($p = 0.017$). Baseline data of the parameters of the CBMN-assay, of physical performance, and of vitamin status (B12 and folic acid) are presented in Table 1.

3.2. Intervention effects

After six months of intervention, 70 participants completed all tests. The drop-outs were due to health problems and lack of motivation. The six month drop-out rate of about 28% was lower than expected (35–40% predicted before starting the study) for this high-age group. Data of the CBMN-assay are presented in Table 2. Table 3 shows the results for performance and vitamin status.

For the parameters of the CBMN-assay, no significant difference between the intervention groups occurred, however not only the RT

Table 1
Baseline characteristics for CBMN assay, physical performance and vitamin status of the intervention group resistance training (RT), resistance training and supplementation (RTS) and cognitive training (CT).

Parameter	All	RT	RTS	CT	p-Value
Subjects [number]	97	35	29	33	0.756
Age [years]	83.0 ± 6.1	82.8 ± 5.7	82.5 ± 7.5	83.5 ± 5.4	0.769
CBMN-parameter					
Cells with MN [per 1000 BNCs]	23.2 ± 11.8	24.6 ± 12.8	22.9 ± 13.4	21.9 ± 9.0	0.785
Total number of MN [per 1000 BNCs]	26.3 ± 12.0	27.6 ± 13.9	25.0 ± 10.8	26.0 ± 10.9	0.880
Nucleoplasmic bridges [per 1000 BNCs]	1.09 ± 1.04	1.13 ± 1.14	0.88 ± 0.89	1.23 ± 1.05	0.411
Nuclear buds [per 1000 BNCs]	3.72 ± 3.43	3.56 ± 2.75	3.34 ± 2.62	4.23 ± 4.57	0.972
Apoptotic cells [% per 1000 BNCs]	0.76 ± 0.55	0.83 ± 0.64	0.69 ± 0.37	0.73 ± 0.58	0.769
Necrotic cells [% per 1000 BNCs]	0.66 ± 0.42	0.73 ± 0.47	0.65 ± 0.35	0.59 ± 0.41	0.270
Nuclear division index	1.98 ± 0.07	1.96 ± 0.08	1.99 ± 0.06	1.99 ± 0.07	**0.017**
Performance parameters					
Chair rise [repetitions]	12.1 ± 4.1	11.9 ± 3.3	12.6 ± 4.8	11.9 ± 4.1	0.674
6 minute walking [m]	368 ± 97	373 ± 87	353 ± 102	377 ± 103	0.600
Handgrip [kg]	18.4 ± 6.5	19.8 ± 6.6	18.7 ± 6.6	16.7 ± 5.9	0.095
Vitamin status					
B12 [pmol/l]	393 ± 379	346 ± 268	452 ± 432	388 ± 433	0.721
Folic acid [nmol/l]	186 ± 110	204 ± 116	154 ± 33	192 ± 142	0.441

Data are means ± standard deviation; p-values are calculated using Kruskal–Wallis-H test and chi-square test for group differences; significant differences are highlighted using bold formatting. Data for MN have been sex-adjusted according to Bonassi et al. (Steffen et al., 2002).

Table 2
The effect of 3 (T2) and 6 (T3) months of intervention [resistance training (RT), resistance training and supplementation (RTS) and cognitive training (CT)] on the parameters of the CBMN-assay.

Intervention	RT				RTS				CT			
	T1	T2	T3	p	T1	T2	T3	p	T1	T2	T3	p
Subjects [number]	35	23	26		29	27	22		33	22	22	
CBMN-parameter												
Cells with MN [per 1000 BNCs]	24.6 ± 12.8	18.9 ± 10.6	19.9 ± 11.0	0.561	22.9 ± 13.4	19.4 ± 11.1	18.9 ± 12.0	0.411	21.9 ± 9.0	16.7 ± 6.1	19.2 ± 9.5	0.252
Total number of MN [per 1000 BNCs]	27.6 ± 13.9	22.8 ± 13.2	24.1 ± 14.9	0.943	25.0 ± 10.8	23.9 ± 15.7	22.4 ± 14.4	0.522	26.0 ± 10.9	19.7 ± 74	23.5 ± 12.5	0.278
Nucleoplasmic bridges [per 1000 BNCs]	1.13 ± 1.14	0.53 ± 0.72	0.50 ± 0.49	0.491	0.88 ± 0.89	0.52 ± 0.62	0.59 ± 1.16	0.129	1.23 ± 1.05	0.57 ± 0.63	0.64 ± 0.85	**0.025**
Nuclear buds [per 1000 BNCs]	3.56 ± 2.75	3.24 ± 2.34	3.93 ± 3.96	0.940	3.34 ± 2.62	2.63 ± 2.45	2.98 ± 2.35	0.442	4.23 ± 4.57	3.53 ± 3.83	3.03 ± 3.08	0.759
Apoptotic cells [% per 1000 BNCs]	0.83 ± 0.64	0.58 ± 0.28	0.51 ± 0.32	0.257	0.69 ± 0.37	0.51 ± 0.34	0.48 ± 0.23	**0.024**	0.73 ± 0.58	0.62 ± 0.35	0.43 ± 0.23	**0.021**
Necrotic cells [% per 1000 BNCs]	0.73 ± 0.47	0.55 ± 0.37	0.47 ± 0.25	0.113	0.65 ± 0.35	0.59 ± 0.54	0.41 ± 0.26	0.084	0.59 ± 0.41	0.54 ± 0.38	0.46 ± 0.27	0.838
Nuclear division index	1.96 ± 0.08	2.00 ± 0.06	1.97 ± 0.08	0.186	1.99 ± 0.06	1.97 ± 0.08	1.96 ± 0.08	0.285	1.99 ± 0.07	1.96 ± 0.07	1.97 ± 0.06	0.471

Data are means ± standard deviation; p-values are calculated using Friedman test for differences between time points; significant differences are highlighted using bold formatting.

and RTS but also the CT group showed similar tendencies for reduction of chromosomal damage. Possibly due to the large standard deviation, only nucleoplasmic bridges (CT: $p = 0.025$) and apoptotic cells (RTS: $p = 0.024$; CT: $p = 0.021$) showed significant time effects. After six months of intervention, we observed a non-significant reduction of cells with MN (RT: −19%, RTS: −18%, CT: −12%) as well as the total number of MN (RT: −13%, RTS: −10%, CT: −10%) in our study cohort of institutionalized elderly (Table 2).

The participants of the RT and the RTS groups demonstrated significant improvements in the chair rise and the 6 minute walking tests (chair rise: RT: +23%, $p = 0.002$, RTS: +24%, $p = 0.001$; 6 minute walking: RT: +13%, $p = 0.021$, RTS +11%, $p = 0.015$). The CT group did not improve in functional parameters and none of the three groups improved significantly at the handgrip strength test.

Vitamin status changed significantly only in the RTS group (plasma B12: +130%, $p = 0.006$; red blood cell folate: +43%, $p = 0.018$) after six months. Furthermore, only in the RTS group, a significant negative correlation between the six month change of the B12 plasma level and the MN frequency was observed (RTS: $r = -0.584$, $p = 0.009$; RT: $r = 0.081$, $p = 0.715$; CT: $r = -0.259$, $p = 0.284$), which indicates decreased chromosomal damage with increased B12 plasma status (Fig. 1).

3.3. Drop-out analysis

To investigate possible reasons for drop-outs in this study, we compared baseline data of those who performed the six month testing ($N = 70$) with those who quit ($N = 27$) before the last sampling.

Interestingly, we observed significant differences between finishers and drop-outs (Fig. 2). Finishers showed significant lower MN frequencies (−20%, $p = 0.034$), a better physical performance in the 6 minute walking test (+24%, $p = 0.002$) and also at the chair rise test (+21%, $p = 0.023$) at baseline.

4. Discussion

4.1. Baseline characteristics

The aim of the present study was to investigate the effect of either a strength training, a strength training and nutritional, or a cognitive training intervention on chromosomal damage in PBMCs in a cohort of elderly institutionalized, women and men.

Although more women than men participated in this study (87.6% female), the gender distribution was representative for the group of people living in retirement homes in Vienna (KWP, Kuratorium-Wiener-Pensionisten-Wohnhäuser, 2014a).

The mean age of the study population (women 82.9 ± 6.0 years; men 84.9 ± 6.7 years) reflects the high age of subjects from institutionalized facilities and was almost seven years higher than the present life expectancy for men in Austria (StatisticsAustria, 2014; KWP, Kuratorium-Wiener-Pensionisten-Wohnhäuser, 2014b).

Although only very limited data are available for this age group (8th, 9th and 10th decade), our results seem to be within the given range for the CBMN-assay (Fenech, 2007). Notably, our data on MN formation in the elderly clearly demonstrate that there is a great heterogeneity in this age group, with the mean value (26.3 ± 12.0 MN/1000 BN cells) being at the upper limit of the expected range for the CBMN-assay

Table 3
The impact of 3 (T2) and 6 (T3) months of intervention [resistance training (RT), resistance training and supplementation (RTS) and cognitive training (CT)] on physical performance and vitamin status.

Intervention	RT				RTS				CT			
	T1	T2	T3	p	T1	T2	T3	p	T1	T2	T3	p
Subjects [number]	35	23	26		29	27	22		33	22	22	
Performance parameters												
Chair rise [repetitions]	11.9 ± 3.3	13.6 ± 3.7	14.6 ± 3.7	**0.002**	13.0 ± 4.4	14.3 ± 4.6	16.1 ± 5.4	**0.001**	11.9 ± 4.2	11.8 ± 3.5	11.3 ± 3.3	0.829
6 minute walking [m]	373 ± 87	394 ± 89	422 ± 91	**0.021**	357 ± 101	399 ± 158	396 ± 143	**0.015**	373 ± 102	374 ± 117	383 ± 111	0.650
Handgrip [kg]	19.8 ± 6.6	20.4 ± 62	20.2 ± 6.1	0.610	19.1 ± 6.4	19.4 ± 6.2	19.4 ± 5.9	0.629	16.7 ± 6.0	17.2 ± 7.0	16.3 ± 6.8	0.316
Vitamin status												
B12 [pmol/l]	346 ± 268	388 ± 349	384 ± 352	0.738	452 ± 432	799 ± 802	1047 ± 934	**0.006**	388 ± 433	607 ± 925	439 ± 629	0.956
Folic acid [nmol/l]	204 ± 116	186 ± 111	228 ± 123	0.678	154 ± 33	231 ± 150	216 ± 83	**0.018**	192 ± 142	207 ± 129	206 ± 70	0.204

Data are means ± standard deviation; p-values are calculated using Friedman test for differences between time points; significant differences are highlighted using bold formatting.

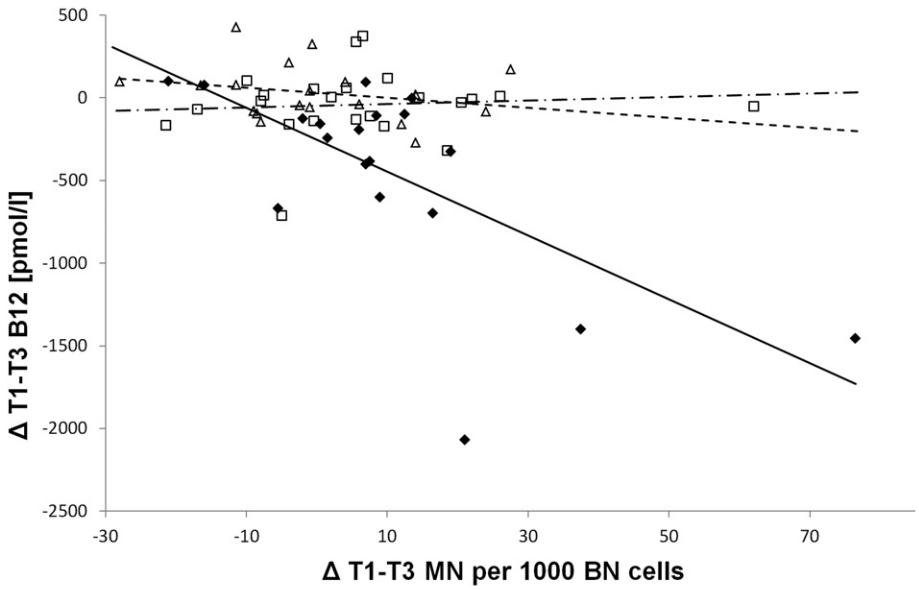

Fig. 1. Correlation between six month change (T1–T3) of plasma B12 status and MN frequency. Filled square — RTS group (full line), empty square — RT group (dotted line), triangle — CT group (broken line); RTS: r = −0.584, p = 0.009; RT: r = 0.081, p = 0.715; CT: r = −0.259, p = 0.284.

(0–30 MN/1000 BN cells) (Fenech, 2007). Considering the individual range, we observed MN frequencies from 5.5 MN/1000 BN cells up to 98 MN/1000 BN cells. Deviating from what has been reported on younger age groups, no significant difference between men and women was observed. However, as described in the literature (Fenech et al., 1994; Norppa and Falck, 2003), the total number of MN and cells with MN was about 10% higher in women than in men (data not shown).

Besides gender, it is well known that aging highly influences the formation of MN. Most studies focusing on the incidence of MN over the human lifespan reported a linear relationship until the age of 60–70 years (Fenech and Bonassi, 2011; Wallner et al., 2012; Bonassi et al., 2001; Peace and Succop, 1999). So far, only very little data are available on study cohorts in their 80s or 90s, demonstrating hints for opposite observations (Bolognesi et al., 1997; Bonassi et al., 1995; Wojda et al., 2007; Fenech, 1998). In our subjects of very old institutionalized elderly, at or above life expectancy, we did not observe any age related effects. Interestingly, even a tendency for lower MN frequency with higher age occurred. Investigating the same subjects, we recently showed that there is a leveling-off of the MN frequency at about 60–70 years of age (Franzke et al., 2014). These findings indicate that there might be a threshold of genome instability that limits the upper rate of MN formation. If the “survivors” are able to reach that high age of 85 years or above, they seem to be either more resistant to MN formation or cells with MN are more likely to undergo apoptosis (Wojda et al., 2006, 2007).

At baseline, the subjects of the present study demonstrated a low physical performance compared to reference values for maintaining physical independence at this age (Werle et al., 2009; Rikli and Jones, 2013). They relatively performed best at the chair rise test where they reached 104 ± 37% of the reference for their age. Nonetheless, our results demonstrated the relatively weak physical condition of our study participants that performed 90 ± 30% in the handgrip strength test and only 68 ± 18% in the 6 minute walking test, compared to normative values. In the elderly, aerobic fitness seems to be of great importance to prevent genomic instability. Mota et al. (Mota et al., 2010) clearly demonstrated that elderly subjects with higher VO_2max showed less DNA damage and higher mitochondrial activity. As we observed a negative correlation between MN frequency and the 6 minute walking test, our results confirm this aspect, demonstrating a link between chromosomal damage and aerobic fitness (Franzke et al., 2014).

According to the definitions of the World Health Organization (WHO), our subjects showed a lower red blood cell folate status but an appropriate plasma vitamin B12 level (de Benoist, 2008).

4.2. Intervention effects

After six months of strength training alone, combined with dietary supplementation, or cognitive training, our subjects showed a tendency for decreased chromosomal damage, independently of the intervention. We observed reductions in MN frequency between 10–15% (Table 2), but due to the high standard deviation the results were not significant. Only very scattered observations about the effect of exercise on MN formation are available and their conclusions are inconsistent, whereas in institutionalized elderly they are missing completely. Goon et al. (Goon et al., 2008) compared people performing Tai Chi (N = 35; age = 58.5 years) for at least one year with sedentary people (N = 35; age = 57.0 years) and observed significantly lower chromosomal damage in the first cohort. Similarly, Huang et al. (Huang et al., 2009) observed lower MN frequencies in Japanese metal workers with higher physical activity. Studies of very intense or very long exercise regimes showed contrary results. Endurance trained athletes did not show higher MN frequency after an ironman triathlon (Reichhold et al., 2008) but acute exhaustive sprint exercise increased the MN frequency significantly (Schiffl et al., 1997). However, there seems to be a dose-dependent effect of exercise on DNA damage since very prolonged exhausting and/or unaccustomed exercise could impair the balance between ROS production and the antioxidant defense system, therefore possibly leading to chronic oxidative stress. As Radak et al. (Radak et al., 2013) pointed out, a moderate level of oxidative stress is essential for the adaptive response after exercise. To our knowledge, we were the first to perform an intervention study to investigate the effect of six month resistance exercise on chromosomal damage in the very oldest of our society.

At the six-month time point, only the RT and the RTS group improved significantly at the chair rise and the 6 minute walking test. There was no change at the handgrip strength test for any group (Table 3).

Although the CT group had neither a physical exercise intervention nor a nutritional supplementation, they surprisingly showed similar tendencies for decreased chromosomal damage compared to the RT

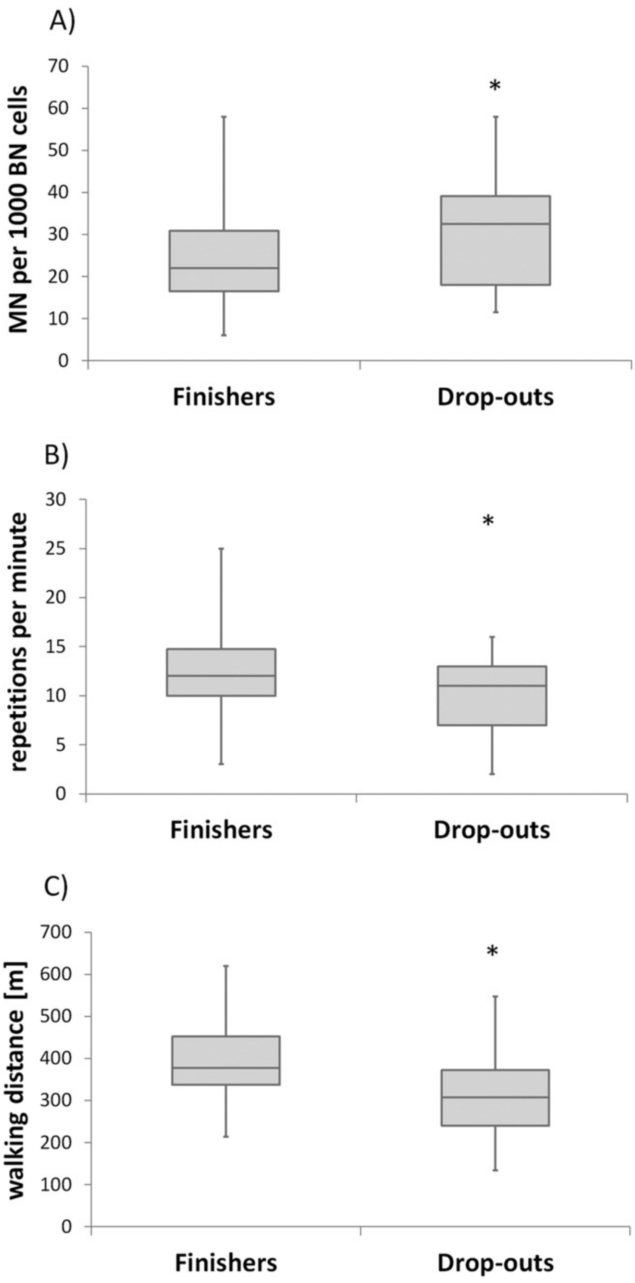

Fig. 2. Comparison of baseline data: study finishers vs. drop-outs. Boxplot A) MN frequency ($p = 0.034$), boxplot B) chair rise test ($p = 0.023$), boxplot C) 6 minute walking test ($p = 0.002$).

and the RTS groups. Similar to physical activity, socialization and psychological stress seem to have an impact on DNA damage (Gidron et al., 2006; Møller et al., 1996; Bouayed et al., 2009). Notably, in elderly institutionalized people, oxidative stress is higher when compared to free-living subjects (Maugeri et al., 2004). Social isolation and the psychosocial environment have severe impacts on the subjects' wellbeing, influencing stress parameters and the endocrinal system and therefore affecting DNA stability (Arnetz et al., 1983; Epel, 2009). As the participants of the current study met twice a week for 60 min and performed either strength training or cognitive exercises, these social events might have improved resistance against chromosomal and DNA damage in the CT group to a similar extent as in the RT and RTS groups. We hypothesize therefore that being a part in this study motivated the subjects of the CT group, which seems to induce similar biochemical changes for their body as in the physical training groups.

Only the RTS group showed significant changes in vitamin B12 and folate status after six months of consuming the provided supplement. It is well known, that these two nutrients are strongly linked to cell differentiation and therefore correlate particularly with the MN frequency (Fenech and Bonassi, 2011; Ni et al., 2012). Although our subjects showed a sufficient B12 plasma level at baseline, we observed a significant negative correlation between the six month change of B12 and the six-month-change of the MN frequency in the RTS group ($r = -0.584$, $p = 0.009$) (Fig. 1). Consequently, an increase in vitamin B12, due to supplementation, is associated with a decrease in chromosomal damage in our study cohort of institutionalized elderly. This observation underlines the importance of a well-balanced diet and a sufficient supply of nutrients in this age-group. Therefore it could be discussed to recommend a supplementation of B12 and folic acid in this very high age group in order to prevent chromosomal damage.

4.3. Drop-out analysis

Compliance with a treatment, either medical, nutritional or physical, is of great importance for the success of a therapy and for human intervention studies (Rich et al., 1996; Hubbard et al., 2012). Therefore, we decided to compare the baseline data of the study finishers to the drop-outs.

Interestingly, the finishers differed significantly from the drop-outs in MN frequency, in the 6 minute walking test and the chair rise test (Fig. 2). These results indicate, that elderly institutionalized study participants with better physical performance and a lower cellular mutation rate are more likely to finish an intervention program than same-aged subjects with lower fitness and higher chromosomal damage from the same study cohort. These parameters can therefore be discussed as predictors for lifestyle interventions.

5. Conclusion

To our knowledge, we were the first to investigate the effect of a six-month lifestyle intervention of cognitive training, resistance training alone or combined with a nutritional supplement on resistance against chromosomal damage in highly aged institutionalized elderly, as measured by the CBMN-assay. In this very heterogenic age group, we observed a tendency for a reduction of the MN frequency and of cells with MN of up to −15% and −20% respectively. Unexpectedly, the participants of the CT group also demonstrated similar tendencies for chromosomal damage, whereas only the RT and RTS groups improved their physical performance. After six months of consuming the provided supplement, the RTS group showed significant higher vitamin B12 and folate levels and their B12 increase correlated significantly with a reduction of the MN frequency. Interestingly, those subjects with higher chromosomal damage and lower physical performance at baseline were more likely to quit the study before the last samplings.

Our results from studying a cohort of very old institutionalized elderly led us to several conclusions: 1) Higher B12 plasma levels, due to an oral supplementation containing vitamin B12, are negatively linked to chromosomal damage. 2) Compliance with a human intervention study seems to be influenced by the subjects' baseline physical condition. 3) In this age group either physical or cognitive training could achieve similar biochemical changes which tend to enhance resistance against genomic instability. 4) Social events and social well-being might be an important factor in institutionalized elderly's health.

With the increasing life expectancy and consequently more people moving to residential care homes, further studies of the elderly, especially of the very oldest of our society, are demanded to investigate the underlying mechanisms of exercise and nutrition on cellular mutation.

Supplementary data to this article can be found online at http://dx.doi.org/10.1016/j.exger.2015.03.001.

Acknowledgment

The authors thank the Curatorship of Viennese Retirement Homes and its residents for taking part in the study. Special thanks go to Dr. Oliver Neubauer for revising and improving the manuscript. This work was supported by the University of Vienna by funding the Research Platform Active Ageing and the Anniversary Fund of the Austrian National Bank (No. 14541).

References

Arnetz, B.B., Theorell, T., Levi, L., Kallner, A., Eneroth, P., 1983. An experimental study of social isolation of elderly people: psychoendocrine and metabolic effects. Psychosom. Med. 45 (5), 395–406.

Arnold, P., Bautmans, I., 2014. The influence of strength training on muscle activation in elderly persons: a systematic review and meta-analysis. Exp. Gerontol. 58C, 58–68. http://dx.doi.org/10.1016/j.exger.2014.07.012.

Battershill, J.M., Burnett, K., Bull, S., 2008. Factors affecting the incidence of genotoxicity biomarkers in peripheral blood lymphocytes: impact on design of biomonitoring studies. Mutagenesis 23 (6), 423–437. http://dx.doi.org/10.1093/mutage/gen040.

Bolognesi, C., Abbondandolo, A., Barale, R., et al., 1997. Age-related increase of baseline frequencies of sister chromatid exchanges, chromosome aberrations, and micronuclei in human lymphocytes. Cancer Epidemiol. Biomarkers Prev. 6 (4), 249–256.

Bonassi, S., Bolognesi, C., Abbondandolo, A., et al., 1995. Influence of sex on cytogenetic end points: evidence from a large human sample and review of the literature. Cancer Epidemiol. Biomarkers Prev. 4 (6), 671–679.

Bonassi, S., Fenech, M., Lando, C., et al., 2001. HUman MicroNucleus project: international database comparison for results with the cytokinesis-block micronucleus assay in human lymphocytes: I. Effect of laboratory protocol, scoring criteria, and host factors on the frequency of micronuclei. Environ. Mol. Mutagen. 37 (1), 31–45.

Bouayed, J., Rammal, H., Soulimani, R., 2009. Oxidative stress and anxiety: relationship and cellular pathways. Oxidative Med. Cell. Longev. 2 (2), 63–67.

de Benoist, B., 2008. Conclusions of a WHO Technical Consultation on folate and vitamin B12 deficiencies. Food Nutr. Bull. 29 (2 Suppl.), S238–S244.

Demontis, F., Piccirillo, R., Goldberg, A.L., Perrimon, N., 2013. The influence of skeletal muscle on systemic aging and lifespan. Aging Cell 12 (6), 943–949. http://dx.doi.org/10.1111/acel.12126.

Epel, E.S., 2009. Psychological and metabolic stress: a recipe for accelerated cellular aging? Hormones (Athens) 8 (1), 7–22.

Fenech, M., 1998. Important variables that influence base-line micronucleus frequency in cytokinesis-blocked lymphocytes—a biomarker for DNA damage in human populations. Mutat. Res. 404 (1–2), 155–165.

Fenech, M., 2007. Cytokinesis-block micronucleus cytome assay. Nat. Protoc. 2 (5), 1084–1104. http://dx.doi.org/10.1038/nprot.2007.77.

Fenech, M., Bonassi, S., 2011. The effect of age, gender, diet and lifestyle on DNA damage measured using micronucleus frequency in human peripheral blood lymphocytes. Mutagenesis 26 (1), 43–49. http://dx.doi.org/10.1093/mutage/geq050.

Fenech, M., Neville, S., Rinaldi, J., 1994. Sex is an important variable affecting spontaneous micronucleus frequency in cytokinesis-blocked lymphocytes. Mutat. Res. 313 (2–3), 203–207.

Franzke, B., Halper, B., Hofmann, M., et al., 2014. The influence of age and aerobic fitness on chromosomal damage in Austrian institutionalised elderly. Mutagenesis http://dx.doi.org/10.1093/mutage/geu042.

Fulle, S., Protasi, F., Di Tano, G., et al., 2004. The contribution of reactive oxygen species to sarcopenia and muscle ageing. Exp. Gerontol. 39 (1), 17–24.

Gatterer, G., Croy, A., 2004. Geistig fit ins Alter. Springer, Vienna.

Gianni, P., Jan, K.J., Douglas, M.J., Stuart, P.M., Tarnopolsky, M.A., 2004. Oxidative stress and the mitochondrial theory of aging in human skeletal muscle. Exp. Gerontol. 39 (9), 1391–1400. http://dx.doi.org/10.1016/j.exger.2004.06.002.

Gidron, Y., Russ, K., Tissarchondou, H., Warner, J., 2006. The relation between psychological factors and DNA-damage: a critical review. Biol. Psychol. 72 (3), 291–304. http://dx.doi.org/10.1016/j.biopsycho.2005.11.011.

Goon, J.A., Noor Aini, A.H., Musalmah, M., Yasmin Anum, M.Y., Wan Ngah, W.Z., 2008. Long term Tai Chi exercise reduced DNA damage and increased lymphocyte apoptosis and proliferation in older adults. Med. J. Malaysia 63 (4), 319–324.

Huang, P., Huang, B., Weng, H., Nakayama, K., Morimoto, K., 2009. Effects of lifestyle on micronuclei frequency in human lymphocytes in Japanese hard-metal workers. Prev. Med. 48 (4), 383–388. http://dx.doi.org/10.1016/j.ypmed.2008.12.023.

Hubbard, G.P., Elia, M., Holdoway, A., Stratton, R.J., 2012. A systematic review of compliance to oral nutritional supplements. Clin. Nutr. 31 (3), 293–312. http://dx.doi.org/10.1016/j.clnu.2011.11.020.

Johnston, A.P., De Lisio, M., Parise, G., 2008. Resistance training, sarcopenia, and the mitochondrial theory of aging. Appl. Physiol. Nutr. Metab. 33 (1), 191–199. http://dx.doi.org/10.1139/H07-141.

Jones, C.J., Rikli, R.E., Beam, W.C., 1999. A 30-s chair-stand test as a measure of lower body strength in community-residing older adults. Res. Q. Exerc. Sport 70 (2), 113–119. http://dx.doi.org/10.1080/02701367.1999.10608028.

KWP, Kuratorium-Wiener-Pensionisten-Wohnhäuser, 2014a. Geschäftsbericht der Häuser zum Leben 2012. http://www.kwp.at/pics/web/gb.pdf (accessed march 17, 2014).

KWP, Kuratorium-Wiener-Pensionisten-Wohnhäuser, 2014b. Die Geschichte des KWP. http://kwp.at/geschichte.aspx (accessed march 17, 2014).

Lustgarten, M.S., Price, L.L., Chale, A., Phillips, E.M., Fielding, R.A., 2014. Branched chain amino acids are associated with muscle mass in functionally limited older adults. J. Gerontol. A Biol. Sci. Med. Sci. 69 (6), 717–724. http://dx.doi.org/10.1093/gerona/glt152.

Maugeri, D., Santangelo, A., Bonanno, M.R., et al., 2004. Oxidative stress and aging: studies on an East-Sicilian, ultraoctagenarian population living in institutes or at home. Arch. Gerontol. Geriatr. Suppl. 9, 271–277. http://dx.doi.org/10.1016/j.archger.2004.04.037.

Mijnarends, D.M., Meijers, J.M., Halfens, R.J., et al., 2013. Validity and reliability of tools to measure muscle mass, strength, and physical performance in community-dwelling older people: a systematic review. J. Am. Med. Dir. Assoc. 14 (3), 170–178. http://dx.doi.org/10.1016/j.jamda.2012.10.009.

Møller, P., Wallin, H., Knudsen, L.E., 1996. Oxidative stress associated with exercise, psychological stress and life-style factors. Chem. Biol. Interact. 102 (1), 17–36.

Mota, M.P., Peixoto, F.M., Soares, J.F., et al., 2010. Influence of aerobic fitness on age-related lymphocyte DNA damage in humans: relationship with mitochondria respiratory chain and hydrogen peroxide production. Age (Dordr.) 32 (3), 337–346. http://dx.doi.org/10.1007/s11357-010-9138-8.

Müllner, E., Brath, H., Toferer, D., et al., 2013. Genome damage in peripheral blood lymphocytes of diabetic and non-diabetic individuals after intervention with vegetables and plant oil. Mutagenesis 28 (2), 205–211. http://dx.doi.org/10.1093/mutage/ges073.

Müllner, E., Brath, H., Nersesyan, A., et al., 2014. Nuclear anomalies in exfoliated buccal cells in healthy and diabetic individuals and the impact of a dietary intervention. Mutagenesis 29 (1), 1–6. http://dx.doi.org/10.1093/mutage/get056.

Nelson, M.E., Rejeski, W.J., Blair, S.N., et al., 2007. Physical activity and public health in older adults: recommendation from the American College of Sports Medicine and the American Heart Association. Circulation 116 (9), 1094–1105. http://dx.doi.org/10.1161/CIRCULATIONAHA.107.185650.

Ni, J., Liang, Z., Zhou, T., Cao, N., Xia, X., Wang, X., 2012. A decreased micronucleus frequency in human lymphocytes after folate and vitamin B12 intervention: a preliminary study in a Yunnan population. Int. J. Vitam. Nutr. Res. 82 (6), 374–382. http://dx.doi.org/10.1024/0300-9831/a000134.

Norppa, H., Falck, G.C., 2003. What do human micronuclei contain? Mutagenesis 18 (3), 221–233.

Peace, B.E., Succop, P., 1999. Spontaneous micronucleus frequency and age: what are normal values? Mutat. Res. 425 (2), 225–230.

Radak, Z., Zhao, Z., Koltai, E., Ohno, H., Atalay, M., 2013. Oxygen consumption and usage during physical exercise: the balance between oxidative stress and ROS-dependent adaptive signaling. Antioxid. Redox Signal. 18 (10), 1208–1246. http://dx.doi.org/10.1089/ars.2011.4498.

Reichhold, S., Neubauer, O., Ehrlich, V., Knasmüller, S., Wagner, K.H., 2008. No acute and persistent DNA damage after an Ironman triathlon. Cancer Epidemiol. Biomarkers Prev. 17 (8), 1913–1919. http://dx.doi.org/10.1158/1055-9965.EPI-08-0293.

Rich, M.W., Gray, D.B., Beckham, V., Wittenberg, C., Luther, P., 1996. Effect of a multidisciplinary intervention on medication compliance in elderly patients with congestive heart failure. Am. J. Med. 101 (3), 270–276.

Rikli, R.E., Jones, C.J., 2013. Development and validation of criterion-referenced clinically relevant fitness standards for maintaining physical independence in later years. Gerontologist 53 (2), 255–267. http://dx.doi.org/10.1093/geront/gns071.

Schiffl, C., Zieres, C., Zankl, H., 1997. Exhaustive physical exercise increases frequency of micronuclei. Mutat. Res. 389 (2–3), 243–246.

Seene, T., Kaasik, P., Riso, E.M., 2012. Review on aging, unloading and reloading: changes in skeletal muscle quantity and quality. Arch. Gerontol. Geriatr. 54 (2), 374–380. http://dx.doi.org/10.1016/j.archger.2011.05.002.

Shahar, S., Kamaruddin, N.S., Badrasawi, M., et al., 2013. Effectiveness of exercise and protein supplementation intervention on body composition, functional fitness, and oxidative stress among elderly Malays with sarcopenia. Clin. Interv. Aging 8, 1365–1375. http://dx.doi.org/10.2147/CIA.S46826.

Smoliner, C., Norman, K., Wagner, K.H., Hartig, W., Lochs, H., Pirlich, M., 2009. Malnutrition and depression in the institutionalised elderly. Br. J. Nutr. 102 (11), 1663–1667. http://dx.doi.org/10.1017/S0007114509990900.

StatisticsAustria, 2014. Life expectancy according to life tables 1970/72–2010/12. www.statistik.at (accessed March 17, 2014).

Steffen, T.M., Hacker, T.A., Mollinger, L., 2002. Age- and gender-related test performance in community-dwelling elderly people: Six-Minute Walk Test, Berg Balance Scale, Timed Up & Go Test, and gait speeds. Phys. Ther. 82 (2), 128–137.

Umegaki, K., Higuchi, M., Inoue, K., Esashi, T., 1998. Influence of one bout of intensive running on lymphocyte micronucleus frequencies in endurance-trained and untrained men. Int. J. Sports Med. 19 (8), 581–585. http://dx.doi.org/10.1055/s-2007-971963.

Wallner, M., Blassnigg, S.M., Marisch, K., et al., 2012. Effects of unconjugated bilirubin on chromosomal damage in individuals with Gilbert's syndrome measured with the micronucleus cytome assay. Mutagenesis 27 (6), 731–735. http://dx.doi.org/10.1093/mutage/ges039.

Werle, S., Goldhahn, J., Drerup, S., Simmen, B.R., Sprott, H., Herren, D.B., 2009. Age- and gender-specific normative data of grip and pinch strength in a healthy adult Swiss population. J. Hand. Surg. Eur. Vol. 34 (1), 76–84. http://dx.doi.org/10.1177/1753193408096763.

Wojda, A., Zietkiewicz, E., Mossakowska, M., Pawłowski, W., Skrzypczak, K., Witt, M., 2006. Correlation between the level of cytogenetic aberrations in cultured human lymphocytes and the age and gender of donors. J. Gerontol. A Biol. Sci. Med. Sci. 61 (8), 763–772.

Wojda, A., Zietkiewicz, E., Witt, M., 2007. Effects of age and gender on micronucleus and chromosome nondisjunction frequencies in centenarians and younger subjects. Mutagenesis 22 (3), 195–200. http://dx.doi.org/10.1093/mutage/gem002.
