# Supplementary material for: Effects of elastic band resistance training on the physical and mental health of elderly individuals: A mixed methods systematic review
Source: PLoS One. 2024 May 13;19(5):e0303372. doi: 10.1371/journal.pone.0303372 (PMC11090353; doi:10.1371/journal.pone.0303372)
Supplement: S1 File — (ZIP) [file pone.0303372.s001.zip › Supporting Information/Included study 42.pdf]

Fitness/Older Adults

Effects of Resistance Training on Functional Ability in Elderly Individuals

Mariane M. Fahlman, PhD; Nancy McNevin, PhD; Debra Boardley, PhD, RD, LD; Amy Morgan, PhD; Robert Topp, PhD, RN

Abstract

Purpose. *Determine the effects of 16 weeks of strength training on measures of functional ability in elderly who are functionally limited.*

Design. *Quasi-experimental trial in which elderly volunteers were assigned to either an exercise group or a control group.*

Participants. *Eighty-seven participants (65–93 years) living independently but with some functional limitations.*

Intervention. *Thirteen different strength training exercises using Thera-Band resistive bands (Hygenic Corporation, Akron, Ohio). The program was 16 weeks in duration, and the frequency was three times per week. Participants exercised in a group setting one time per week and were given a home exercise book to follow for two additional sessions per week.*

Measures. *Functional ability was operationalized to include a variety of measures related to functional ability that impact activities of daily living, morbidity, and mortality in the elderly, including upper- and lower-body strength and gait.*

Analysis. *Intervention effects were analyzed using a 2 (groups: exercise group vs. control group) × 3 (time: baseline vs. mid vs. post) analysis of variance.*

Results. *The exercise group demonstrated significant improvements in upper-body strength as measured by biceps curl ($F[2,140] = 39.870$; $p < .05$) and lower-body strength as measured by chair sit-to-stand ($F[2,124] = 25.887$; $p < .05$). Gait velocity ($F[2,140] = 37.317$; $p < .05$) and step length ($F[2,140] = 4.182$; $p < .05$) both increased for the exercise group at week 9, but this increase disappeared by week 17. Compared with minimal changes in the control group, the exercise group demonstrated significant improvements in upper-body strength as measured by biceps curl and lower-body strength as measured by chair sit-to-stand.*

Conclusion. *Some measures of function ability were improved after a 16-week structured exercise program for functionally limited elderly. Because functional ability has been inversely correlated with short-term morbidity and the need for assisted living among older adults, providing opportunities to exercise is crucial to future functioning and independence of the elderly population. (Am J Health Promot 2011;25[4]:237–243.)*

Key Words: Exercise, Elderly, Functional Ability, Resistance Training, Prevention Research. Manuscript format: research; Research purpose: intervention testing; Study design: quasi-experimental; Outcome measures: functional ability; Setting: local community; Health focus: fitness/physical health; Strategy: exercise intervention; Target population: seniors; Target population circumstances: low functional ability

Mariane M. Fahlman, PhD, and Nancy McNevin, PhD, are with Wayne State University, Detroit, Michigan. Dr. McNevin is now with the University of Windsor, Windsor, Ontario, Canada. Debra Boardley, PhD, RD, LD, is with the University of Toledo, Toledo, Ohio. Amy Morgan, PhD, is with Bowling Green State University, Bowling Green, Ohio. Robert Topp, PhD, RN, is with the University of Louisville, Louisville, Kentucky.

Send reprint requests to Mariane M. Fahlman, PhD, Wayne State University, Matthaei 262, Detroit, MI 48202; m.fahlman@wayne.edu.

This manuscript was submitted November 25, 2008; revisions were requested May 25 and August 9, 2009; the manuscript was accepted for publication August 10, 2009.

Copyright © 2011 by American Journal of Health Promotion, Inc.

0890-1171/11/$5.00 + 0
DOI: 10.4278/ajhp.081125-QUAN-292

INTRODUCTION

In the United States, there are currently 36 million people 65 years and older; 4.5 million of this group are older than 85 years. Future projections indicate that by the year 2050, those numbers will increase to 80 million people 65 years and older, with 20 million of them older than 85 years.[1] If medical science continues to develop technologies that prolong lives, those numbers will be still higher. Currently, individuals over 65 years use more than 30% of health care dollars, and those 85 years and older consume more than double the annual health care dollars of those 65 to 85 years.[2] The longer people live, the greater the likelihood that they will develop chronic diseases, placing them at an increased risk for disability and dependence.[3] As the number of older individuals rises, the number of dependent individuals will rise proportionately.[1] A minimum level of functional ability, defined as the ability to perform normal everyday activities safely, independently, and without undue fatigue,[4] is necessary for independent living; thus, an increase or maintenance of functional ability will decrease the population of disabled, dependent elderly.

Multiple researchers are examining exercise as a method to increase functional ability and enhance the prospects for independent living in the elderly.[5,6] Disability is inversely related to physical activity,[7] whereas exercising at least 4 days per week is strongly correlated with enhanced functional ability and ease of performing activities of daily living.[8] This is due, in part, to changes that even a

modest amount of exercise has on gait. Elderly individuals demonstrate significant declines in both kinematic (i.e., step length) and kinetic (i.e., push-off force) aspects of gait compared with those of younger ambulators.[9] These changes are due, in part, to a decrease in lean muscle mass, and as a consequence, muscle strength. In the frail elderly, exacerbation of these deficits leads to increased risk for falls.[10] However, exercise in the form of resistance training has been found to increase muscle strength and endurance in a sample of frail men who were at risk for falls,[11] and Rubenstein et al.[11] noted no increase in the incidence of falls for this sample despite an increase in the amount of activities in which they engaged. Resistance training has also been found to lead to higher gait velocities, improved stair climbing power, and increased spontaneous physical activity[12] in elderly men and women.

Other researchers have demonstrated similar results. Ramsbottom et al.[13] conducted resistance training exercises with community-dwelling elderly over 70 years twice weekly for 24 weeks. At the conclusion of the study, the exercise group demonstrated significant increases in dynamic balance, functional reach, and functional mobility, whereas the control group remained unchanged. McCartney et al.[14] conducted a 2-year resistance training program for community-dwelling seniors 60 to 80 years. Measures taken at 10 months indicated that the exercise group had significant increases in the following: treadmill walking, measures of strength and power, and muscle size. Participants in the control group did not experience any changes. Puggard[3] conducted an 8-month combined aerobic and resistance training program with community-dwelling seniors 75 and 85 years. The 75-year-old group exercised twice weekly, whereas the 85-year-old group only exercised once per week. In spite of the exercise sessions being below the 5 days per week/ 50 minutes per session recommendations for exercise training by the American College of Sports Medicine,[15] the exercise groups exhibited limited improvements in measures of functional ability during the 8-month period, whereas the control group significantly declined in those same measures.

PURPOSE

Previous work has demonstrated that structured resistance training programs lead to increased measures of functional ability in community-dwelling elderly. However, there is no evidence that elderly who are already identified as functionally limited are able to achieve these same benefits or whether these benefits are attainable with a low-cost resistance training program designed to be completed in the home. Therefore, the purpose of this research was to determine the effects of 16 weeks of resistance training on selected measures of functional ability in elderly individuals who exhibited decreased functional ability. Functional ability was operationalized as the number of biceps curls performed in 30 seconds, the number of chair sit-to-stands performed in 30 seconds, gait characteristics, and isokinetic knee and arm flexion and extension. In addition, based on previous research indicating that the ability to ascend stairs was linked to functional ability, we added the time required to ascend and descend a flight of 27 stairs.[16] We hypothesized that elderly adults with limited functional ability who participated in 16 weeks of resistance training would exhibit increased functional ability compared with a nonexercising control group.

METHODS

Design and Sample

Participant Screening, Inclusion, and Exclusion Criteria. This was a quasi-experimental trial conducted in a large metropolitan area. Newspaper advertisements and flyers placed in areas frequented by senior citizens invited prospective participants to call for screening and eligibility for an exercise program. The participant population was independently living seniors who experienced some limitation in function, who were currently not exercising, who had no medical conditions that prohibited exercise, and who were able to participate three times per week for 16 weeks in an exercise intervention. Potential participants underwent telephone prescreening that consisted of 10 questions from the physical functioning component of the Medical Outcomes Study 36-item Short Form Health Survey.[17] A score of 24 out of 30 (mean for individuals older than 65 years) or below characterized the individual as functionally limited. Limitations in this group included difficulty with mobility and difficulty with activities of daily living, such as the ability to dress, use the toilet, and transfer from bed and chair. Additional screening consisted of a physical exam conducted by a physician and 12-lead electrocardiogram. (Figure 1). Once participants were cleared for participation, a random table of numbers was used to assign them to either the exercise group or control group. Participants in each group had similar demographic characteristics (Table 1). The study was approved by the Human Investigation Committee at Wayne State University, Detroit, Michigan, and all participants provided signed informed consent.

Intervention. The exercise group reported to The University of Toledo, Toledo, Ohio, one time per week for group instruction and was instructed to exercise at home on 2 other days per week. The trainers were graduate students in exercise physiology. Participants were trained to use Thera-Band resistance bands (Hygenic Corporation, Akron, Ohio), instructed in the appropriate technique for every exercise, and taught to complete training logs detailing their exercise at home.

The training period lasted 16 weeks with testing occurring during weeks 0 (pre), 9 (mid), and 17 (post). There were no adverse exercise effects, although four participants left the exercise group and nine left the control group for other reasons. All exercise sessions began with a 5-minute warm-up and ended with a 5-minute cool-down. The sessions began with one set of 10 repetitions for weeks 1 and 2 and progressed to two sets of 12 repetitions for weeks 3 to 16.

Thera-Band Resistance. Thera-Band provides a color-coded system of determining resistance. Force

Figure 1
Decision Tree of Participant Screening Procedures

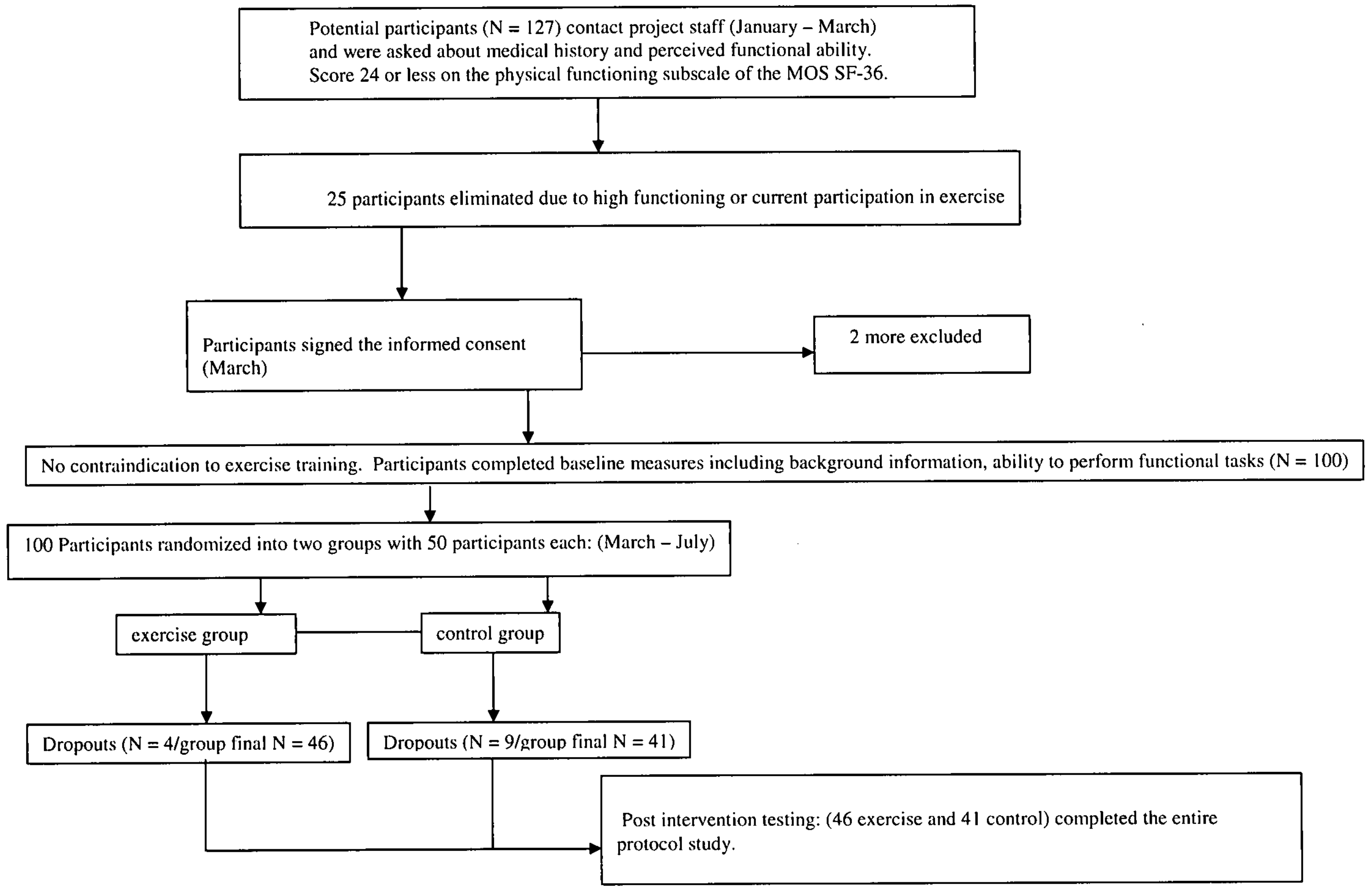

production is dependent on the color and percent elongation. There is a 20% to 30% increase in tension between colors of Thera-Band elastic bands at 100% elongation. The colors (from minimal to maximal strength), yellow, red, green, blue, black, silver, and gold, were coded 1 to 7 for statistical analysis. Participants were instructed to select the color that provided sufficient resistance to produce "mild" fatigue following the last repetition. Exercise leaders encouraged participants to change colors and thus increase the tension as exercises became easier. Different colors based on the strength of the muscle group were used for each exercise (Table 2). The 13 exercises included chair squats, hip flexion, hip extension, standing abduction, standing adduction, chest press, lateral shoulder raise, seated row, abdominal curl-up, biceps curl, triceps extension, calf raise, and toe raise. The control group was instructed to maintain normal activity and not begin any exercise programs during the course of the study. As an incentive to remain in the study, the control group was promised 4 weeks of exercise training at the completion of the investigation. Participants completed training logs that were turned in to the trainers weekly when they reported for the university exercise session. The logs were checked for accuracy; participants reported any concerns, and the trainers made suggestions regarding training, including changing to a higher-resistance band (Table 2).

Functional Ability Variables

Functional ability was measured by the time required to ascend and descend a flight of 27 stairs, the number of biceps curls performed in 30 seconds, the number of chair sit-to-stands performed in 30 seconds, gait characteristics, and isokinetic knee and arm flexion and extension. All of the tests have been shown to be valid and reliable for assessing the performance areas impacting functional ability in older adults.[4,16] Researchers responsible for data collection were blinded to the treatment group and made no effort to determine the treatment group while conducting the testing.

Table 1
Demographic Characteristics*

	Exercise Group (n = 46)	Control Group (n = 41)
Age, y	75 ± 1	76 ± 2
Height, cm	162 ± 2	160 ± 2
Weight, kg	80.2 ± 3.6	78.5 ± 2.5
BMI, kg ÷ m^2	30.5 ± 4.2	29.5 ± 4.2
≤2 chronic conditions (excluding arthritis)	63%	51%
>2 chronic conditions (excluding arthritis)	40%	49%
Arthritis	96%	98%
Completion of high school or greater education	89%	95%
Income ≤$20,000	57%	63%
PF-10	21.4 ± 0.7	21.3 ± 0.8
Compliance with exercise	Mean, 92%; range, 67%–100%	N/A

* Baseline factors between groups were analyzed to determine the effectiveness of randomization. There were no significant differences at baseline between the groups for any demographic or dependent variables. N/A indicates not applicable; PF-10, 10 questions from the physical functioning component of the Medical Outcomes Study 36-item Short Form Health Survey.

Table 2
Sample Data for Thera-Band Color Used for Exercise*

Exercise	Color of Band, mean ± SEM	Approximate Weight, pounds
Squat		
Week 0	2.42 ± 0.15[1,2]	5.46[1,2]
Week 9	4.17 ± 0.33[1]	8.31[1]
Week 17	4.33 ± 0.33[2]	9.23[2]
Bicep curl—right		
Week 0	2.42 ± 0.15[1,2]	5.46[1,2]
Week 9	4.17 ± 0.39[1]	8.31[1]
Week 17	4.33 ± 0.33[2]	9.23[2]
Bicep curl—left		
Week 0	2.42 ± 0.15[1,2]	5.46[1,2]
Week 9	4.17 ± 0.39[1]	8.31[1]
Week 17	4.33 ± 0.33[2]	9.23[2]
Hip extension—right		
Week 0	2.42 ± 0.15[1,2]	5.46[1,2]
Week 9	4.25 ± 0.35[1]	8.88[1]
Week 17	4.33 ± 0.33[2]	9.23[2]
Hip extension—left		
Week 0	2.42 ± 0.15[1,2]	5.46[1,2]
Week 9	4.17 ± 0.39[1]	8.31[1]
Week 17	4.33 ± 0.33[2]	9.23[2]

* The colors (from minimal to maximal strength) were yellow, red, green, blue, black, silver, and gold and were coded 1 to 7 for statistical analysis. Means are based on the color of the Thera-Band: yellow, 2.9 pounds (1); red, 3.9 pounds (2); green, 5 pounds (3); blue, 7.1 pounds (4); black, 9.7 pounds (5); silver, 13.2 pounds (6); and gold, 21.6 pounds (7). Participants changed colors and thus increased tension over time. Significant differences are indicated by superscript. SEM indicates standard error of the mean.

Measurements of Functional Ability Variables

Time Up and Down the Stairs. Each participant was asked to ascend and descend a flight of 27 7-inch steps as quickly as possible. The stair ascent timing began on the participant's first movement and concluded when the participant reached the top of the steps. Following a 30-second rest at the top of the stairs, participants were told to descend the stairs as quickly as possible with timing concluded when the participants reached the start point. The trial was measured to the nearest 10th of a second.[16]

Biceps Curl. Each participant was asked to move a 5-pound (women) or 8-pound (men) weight through the full elbow range of motion, starting from a fully extended arm position, as many times as possible in 30 seconds. The score for this test was the number of full curls completed in 30 seconds, as counted by the test administrator.[4]

Chair Sit-to-Stand. Each participant was asked to rise and be seated in a straight-backed chair (seat = 17 inches) as many times as possible in 30 seconds. The score for this test was the number of stands completed in 30 seconds, as counted by the test administrator.[4]

Knee and Arm Flexion and Extension. Knee and arm flexion and extension were measured using an isokinetic dynamometer (Kin Com 125 AP; Chattanooga Group, Hixon, Tennessee). The concentric force curve demonstrating the greatest average force of each exercise was chosen to represent the participant's maximal strength.

Gait Characteristics. Characteristics of gait were assessed as participants ambulated down an instrumented GAITRite mat (CIR Systems Incorporated, Havertown, Pennsylvania). The GAITRite electronic walkway contains six sensor pads embedded within a roll-up carpet to produce an active area 24 inches (61 cm) wide and 144 inches (366 cm) long. As the participant ambulates down the walkway, the system captures the relative arrangement, geometry, and applied pressure of each footfall as a function of time. The application software, integrated with an IBM-compatible computer, operates the walkway, processes the raw data into footfall patterns, and computes the temporal and spatial parameters. The spatial parameter computed from footfall information was step length, and the temporal parameter computed was gait velocity.

Analysis

The dependent variables of time up and down the stairs, biceps curl, chair sit-to-stand, arm and leg flexion and extension, gait velocity, step time, and step length were analyzed separately using a 2 (groups: exercise group vs. control group) × 3 (time: baseline vs. mid vs. post) analysis of variance with repeated measures on the second factor. *Post hoc* analyses were used to assess significant interactions and main effects. The statistical package used to run all analyses was SPSS (version 16.0; SPSS Inc., Chicago, Ilinois).

RESULTS

The final sample consisted of 41 participants in the control group of 75.6 ± 1.3 years (body mass index [BMI], 29.5 ± 4.2) and 46 participants in the exercise group of 74.8 ± 1.0

Table 3
Measures of Functional Ability*

Test	Week 0 Exercise Group (n = 46)	Week 0 Control Group (n = 41)	Week 9 Exercise Group (n = 46)	Week 9 Control Group (n = 41)	Week 16 Exercise Group (n = 46)	Week 16 Control Group (n = 41)	Significance
Time up stairs, s	18.5 ± 0.9	18.1 ± 1.4	18.7 ± 2.2	16.8 ± 1.2	15.9 ± 1.5	16.0 ± 1.2	Time: $p < 0.05$
Time down stairs, s	20.9 ± 1.9	20.2 ± 2.1	20.9 ± 3.0	18.4 ± 1.7	17.0 ± 2.0	17.8 ± 2.4	NS
Biceps curl, no.	15.2 ± 0.7	15.0 ± 0.8	17.1 ± 0.8	15.8 ± 0.7	19.5 ± 0.9	16.9 ± 0.8	Time: $p < 0.05$; group × time: $p < 0.05$
Chair sit to stand, no.	9.8 ± 0.6	9.5 ± 0.8	12.5 ± 0.7	10.9 ± 0.8	12.8 ± 0.8	10.9 ± 0.8	Time: $p < 0.05$; group × time: $p < 0.05$
Arm flexion, peak torque in pounds	29.1 ± 2.2	26.2 ± 1.7	28.9 ± 2.3	27.2 ± 1.6	39.4 ± 2.4	39.5 ± 2.4	Time: $p < 0.05$
Arm extension, peak torque in pounds	28.5 ± 1.4	27.7 ± 1.7	30.1 ± 1.6	29.4 ± 2.2	30.4 ± 1.6	26.8 ± 1.6	NS
Leg flexion, peak torque in pounds	39.9 ± 2.3	37.2 ± 2.4	40.0 ± 2.6	37.4 ± 2.1	39.4 ± 2.3	39.5 ± 2.4	NS
Leg extension, peak torque in pounds	54.4 ± 3.7	53.0 ± 3.4	54.9 ± 4.0	57.9 ± 3.3	54.0 ± 3.7	54.1 ± 4.2	NS
Gait velocity, m/min	85 ± 5	93 ± 4	93 ± 6	88 ± 3	90 ± 4	92 ± 4	Time: $p < 0.05$
Step time, s	1.1 ± 0.3	1.0 ± 0.2	1.0 ± 0.2	1.0 ± 0.3	1.0 ± 0.3	0.99 ± 0.4	NS
Step length, cm	75 ± 10	99 ± 12	99 ± 13	87 ± 15	84 ± 17	89 ± 15	Time: $p < 0.05$

* NS indicates not significant.

years (BMI, 30.5 ± 4.2) (Table 1). Baseline factors between groups were analyzed to determine the effectiveness of randomization. There were no significant differences at baseline between the groups for any demographic or dependent variables.

Time Up the Stairs

Only the main effect of time ($F[2,132] = 6.934$; $p < .05$) reached statistical significance (Table 3). Subsequent *post hoc* analysis revealed that both groups improved performance at week 17, indicating that the treatment failed to distinguish between groups. Both the main effect of group and the group × time interaction failed to reach significance ($F < 1$).

Time Down the Stairs

Analysis of the data revealed no significant main effects or interactions ($F < 1$).

Biceps Curl

Analysis of the biceps curl data revealed a significant main effect of time ($F[2,140] = 39.870$; $p < .05$), which was qualified by its interaction with group ($F[2,140] = 3.3.873$; $p < .05$). *Post hoc* analysis indicated that although performance between groups was equivalent during baseline and mid training sessions, the exercise group showed improved performance by week 17 compared with the control group. There was no main effect of group ($p = .08$).

Chair Sit-to-Stand

Analysis of the sit-to-stand data revealed a significant main effect of time ($F[2,124] = 25.887$; $p < .05$), which was qualified by its interaction with group ($F[2,124] = 1.509$; $p < .05$). *Post hoc* analysis indicated that although performance between groups was equivalent during baseline testing, the exercise group was significantly better than the control group at weeks 9 and 17. There was no main effect of group ($F < 1$).

Arm Flexion

Analysis of the arm flexion data revealed only a significant main effect of time ($F[2,132] = 83.8$; $p < .05$), indicating that the intervention failed to distinguish between the exercise group and the control group. There were no main effects of group or interaction effects ($F < 1$).

Arm Extension, Knee Extension, and Knee Flexion

Separate analysis of the arm flexion, knee extension, and knee flexion date revealed no significant main effects or interactions ($F < 1$).

Measures of Gait

Velocity. Analysis of the velocity data revealed a significant main effect of time ($F[2,140] = 37.317$; $p < .05$. *Post hoc* analysis indicated that the exercise group improved in week 9 and maintained that improvement through week 17. There was no main effect of group ($p = .07$).

Step Time. Analysis of the data revealed no significant main effects or interactions ($F < 1$).

Step Length. Analysis of the velocity data revealed a significant main effect of time ($F[2,140] = 4.182$; $p < .05$). *Post hoc* analysis indicated that the exercise group improved in week 9 and maintained that improvement through week 17. There was no main effect of group ($p = .08$).

DISCUSSION

Currently researchers are trying to determine which exercise programs have the greatest potential to improve functional ability in elderly individuals. Many studies have combined resistance training with aerobic conditioning, but often only one training program is practical. After testing the efficacy of resistance training, Hunter et al.[18] recommend resistance training as a method of improving functional ability. In the current study, resistance training was effective for increasing upper

strength only as measured by the biceps curl and lower body strength and endurance as measured by the sit-to-stand. There were minimal gains in gait velocity and step length. However, there was no change in other measures of strength, such as arm extension, leg flexion, leg extension, and time up the stairs. The fact that there was no group effect for time up the stairs is most likely due to the fact that aerobic conditioning as well as resistance training is important for increases in that measure. Other authors who have reported increases in measures requiring aerobic conditioning used mixed programs that included both aerobic and resistance training.[3,13,19]

There are parallels between this study and previous work in that the exercise group experienced an 11% increase compared with the control group in both the biceps curl and the chair sit-to-stand. This is consistent with previous studies using elderly participants,[3,13,19] but there were no changes in measures of isokinetic knee and arm flexion and extension or time up and down the stairs. Most likely these results are due to the training principle of specificity. According to this principle, the change in muscle function is specific to the muscle fibers involved in the activity.[20] The specific strength training exercises that the participants engaged in targeted the biceps and chair sit-to-stand. Participants used Thera-Bands to complete these exact exercises three times per week; thus, it would logically follow that they would demonstrate increases in these tasks compared with the control group, who had no such exercise experience. Additionally, participants in the present study trained with Thera Bands but were tested with an isokinetic device. It is possible that a training effect occurred but was specific to isotonic measurement.

Unlike previous studies, the effect of strength training did not lead to a global improvement in the gait parameters of all participants who were exposed to it. However, the results did show that strength training led to statistically significant increases in both temporal (velocity) and spatial (step length) parameters. The finding that only 8 weeks of training made a statistically significant impact on variables such as gait velocity indicates that even in a population whose initial performances were poor, the benefits appear to be fairly immediate. Although we did not observe additional benefits to gait beyond that observed after the 8-week protocol (i.e., after 16 weeks), it is possible that additional training with specific emphasis on gait may encourage improved gains beyond 8 weeks. What was not expected was the failure to detect gait improvements in step time. One possible explanation for these findings is that a longer duration of strength training may be necessary to elicit improvements in older adults.

In conclusion, the specific exercises that were employed resulted in increased strength only when the exact exercises performed were measured. Increases in dynamic strength did not translate into increases in isokinetic strength or increases in measures that require strength plus aerobic endurance training, suggesting that exercise programs targeted to the functionally limited elderly need to include both resistance and aerobic training and should be designed to address specific deficits rather than be generic in nature. However, 16 weeks of strength training was well tolerated by this population of functionally limited elderly, and the intervention resulted in increases in some measures of functional ability. Given the fact that functional ability has been inversely correlated with short-term morbidity and the need for assisted living among older adults, it seems evident that providing opportunities to exercise is crucial to future functioning and independence of the elderly population.

SO WHAT? Implications for Health Promotion Practitioners and Researchers

What is already known on this topic?

We can say with confidence that well designed and executed exercise programs result in increased strength and endurance in the elderly. These increases have been shown to lead to increases in functional capacity and decreased need for institutionalization.

What does this article add?

This research demonstrates that a well designed and executed exercise program also resulted in increased strength and improved gait in elderly who self identify as functionally impaired. These gains were limited to the specific areas exercised and not global in scope. Additionally, this program was designed to be largely home based and self-paced, reducing the need for the elderly to travel to an exercise site and reducing the need for an exercise instructor.

What are the implications for health promotion practice or research?

As the population ages and the number of functionally limited individuals rises, practitioners are searching for ways to enable the elderly to function well without the need for institutionalization. There seems to be moderate support for the assertion that exercise programs for the elderly need to be designed with specific rather than global outcomes in mind. If this assertion holds true, health promotion researchers need to focus on the particular interventions most likely to improve functional capacity in the specific population of elderly they are working with, and health practitioners should encourage the elderly to engage in activities designed specifically with their needs in mind.

Acknowledgment

This study was funded by NIH R01 NR04929 and the Hygenic Corporation.

References

1. Louria DB. Extraordinary longevity: individual and societal issues. *J Am Geriatr Soc.* 2005;53:S317–S319.
2. *Older Americans 2004: Key Indicators of Well-Being.* Washington, DC; 2003.
3. Puggard L. Effects of training on functional performance in 65, 75 and 85 year old women: experiences deriving from community based studies in Odense, Denmark. *Scand J Med Sports.* 2003;13:70–76.
4. Rikli R, Jones C. *Senior Fitness Test Manual.* Champaign, Ill: Human Kinetics; 2001.
5. Latham N, Bennett D, Stretton C, Anderson C. Systematic review of progressive resistance strength training in older adults. *J Gerontol A Biol Sci Med Sci.* 2004;59A:48–61.
6. Nelson M, Layne J, Bernstein M, et al. The effects of multidimensional home-based exercise on functional performance in

elderly people. *J Gerontol A Biol Sci Med Sci.* 2004;59A:154–160.
7. Spirduso W, Cronin D. Exercise dose-response effects on quality of life and independent living in older adults. *Med Sci Sports Exerc.* 2001;33:S598–S608.
8. Stessman J, Hammerman-Rozenberg R, Maaravi Y, Cohen A. Effects of exercise on ease of performing activities of daily living and instrumental activities of daily living from age 70 to 77: the Jerusalem longitudinal study. *J Am Geriatr Soc.* 2002;50:1934–1938.
9. Winter DA, Patla AE, Frank JS, Walt SE. Biomechanical walking pattern changes in the fit and healthy elderly. *Phys Ther.* 1990;70:340–347.
10. DiBrezzo R, Shadden B, Raybon B, Powers M. Exercise intervention designed to improve strength and dynamic balance among community-dwelling older adults. *J Aging Phys Act.* 2005;13:198–209.
11. Rubenstein LZ, Josephson KR, Trueblood PR, et al. Effects of a group exercise program on strength, mobility, and falls among fall-prone elderly men. *J Gerontol A Biol Sci Med Sci.* 2000;55A:M317–M321.
12. Fiatarone MA, O'Neill EF, Ryan ND, et al. Exercise training and nutritional supplementation for physical frailty in very elderly people. *N Engl J Med.* 1994;330:1769–1775.
13. Ramsbottom R, Ambler A, Potter J, et al. The effect of 6 months training on leg power, balance, and functional mobility of independently living adults over 70 years old. *J Aging Phys Act.* 2004;12:497–510.
14. McCartney N, Hicks Al, Martin J, Webber CE. Long-term resistance training in the elderly: effects on dynamic strength, exercise capacity, muscle, and bone. *J Gerontol.* 1995;50A:B97–B104.
15. Mazzeo RS, Tanaka H. Exercise prescription for the elderly. *Sports Med.* 2001;31:809–818.
16. Topp R, Mikesky A, Thompson K, Myere A. Determinants of four functional tasks among older adults: an exploratory regression analysis. *J Orthop Sports Phys Ther.* 1998;27:144–153.
17. McHorney C, Ware J, Lu J, Sherbourne C. The MOS 36-item Short-Form Health Survey (SF36). Tests of data quality, scaling assumptions, and reliability across diverse patient groups. *Medical Care.* 1994;32:40–66.
18. Hunter GR, Treuth MS, Weinsier RL, et al. The effects of strength conditioning on older women's ability to perform daily tasks. *J Am Geriatr Soc.* 1995;43:756–760.
19. Capodaglio P, Capodaglio E, Ferri A, et al. Muscle function and functional ability improves more in community-dwelling older women with a mixed-strength training programme. *Age Ageing.* 2005;34:141–147.
20. Romer L, McConnell A. Specificity and reversibility of inspiratory muscle training. *Med Sci Sport Exerc.* 2003;2003:237–244.

Copyright of American Journal of Health Promotion is the property of American Journal of Health Promotion and its content may not be copied or emailed to multiple sites or posted to a listserv without the copyright holder's express written permission. However, users may print, download, or email articles for individual use.
