# Supplementary material for: Effects of elastic band resistance training on the physical and mental health of elderly individuals: A mixed methods systematic review
Source: PLoS One. 2024 May 13;19(5):e0303372. doi: 10.1371/journal.pone.0303372 (PMC11090353; doi:10.1371/journal.pone.0303372)
Supplement: S1 File — (ZIP) [file pone.0303372.s001.zip › Supporting Information/Included study 56.pdf]

Development and Feasibility of a Senior Elastic Band Exercise Program for Aged Adults: A Descriptive Evaluation Survey

Kuei-Min Chen, PhD,[a] Wei-Shyuan Tseng, BS,[b] Hsin-Ting Huang, BS,[c] and Chun-Huw Li, MS[d]

ABSTRACT

Objective: This study aimed to develop a tailored elastic band exercise program for aged adults (persons 65 years and older), to evaluate the feasibility of a program, and to determine appropriate elastic band exercise frequencies and preferences of aged adults.

Methods: This study had 2 phases. In phase I, 11 professional experts were consulted to develop the Senior Elastic Band (SEB) exercise program. They responded to detailed description and demonstrations of the program contained on either a hard copy or a DVD. In phase II, 20 participants 65 years or older were interviewed for their feedback on the SEB after participating in 1 month of instructor-led SEB group practice. Both quantitative and qualitative strategies were included in the subject evaluation. The quantitative evaluation results were analyzed using descriptive statistics of mean and SD. The qualitative revision suggestions were critically analyzed and summarized using content analysis to revise the program.

Results: Both the experts in phase I and the senior participants in phase II rated the SEB highly and commented that the program was feasible, safe, suitable, and helpful. The participants further suggested practicing SEB 3 times per week for 60 minutes per session in a group of 20 to 29 people.

Conclusions: Based on the feedback from the expert panel, the final SEB included 3 phases with 20 movements. The program took 40 minutes to complete. The SEB program was supported by the 11 experts with 5 professional backgrounds and was well accepted by a small group of community seniors. The participants expressed that the program was feasible, was manageable, and could be helpful to their health promotion. (J Manipulative Physiol Ther 2013;36:505-512)

Key Indexing Terms: *Complementary Therapies; Middle Aged; Aged; Program Description; Strength Training*

Having an appreciation for the health status of aged adults (65 years and older) is the basis for health professionals implementing activities aimed at promoting the health of seniors.[1] Based on a previous study investigating the health status of community seniors, the 6 most common health problems of seniors were as follows: (1) poor cardiopulmonary fitness, (2) decreased body flexibility, (3) weak upper body muscle power, (4) reduced lower body muscle endurance, (5) poor balance, and (6) sleep disturbances.[1] These problems were mostly physically related. Scientific evidence supports that physical activity and exercise provide opportunities to add years of active independent living, reduce disability, and improve seniors' quality of life.[2] Exercise for seniors creates 3 types of health benefits: (1) reduces the risk of developing chronic diseases, (2) aids in the management of chronic diseases, and (3) improves the ability to function and stay independent.[3] Hence, selecting or developing appropriate exercise programs aimed at lessening those health conditions would assist in preventing illness, promoting health, and decreasing the use of health care resources over time.

Among the various exercises, progressive resistance exercises using weights, exercise machines, and elastic bands

[a] Professor, College of Nursing, Kaohsiung Medical University, Kaohsiung, Taiwan.
[b] Instructor, Center for Continuing Education and Social Resources, Kaohsiung Medical University, Kaohsiung, Taiwan.
[c] Research Assistant, College of Nursing, Kaohsiung Medical University, Kaohsiung, Taiwan.
[d] Lecturer, Department of Nursing, Yuhing Junior College of Health Care and Management, Kaohsiung, Taiwan.
Submit reprint requests to: Kuei-Min Chen, PhD, 100 Shih-Chuan 1st Rd, Sanmin District, Kaohsiung 80708, Taiwan (e-mail: *kmc@kmu.edu.tw*).
Paper submitted February 9, 2013; in revised form July 24, 2013; accepted July 25, 2013.
0161-4754/$36.00
Copyright © 2013 by National University of Health Sciences.
http://dx.doi.org/10.1016/j.jmpt.2013.08.002

can increase muscle strength and size in seniors.[4,5] Elastic band exercises are recognized as safe, convenient, inexpensive, and effective strategies to enhance the neuromuscular system, improve muscle strength and power, and increase the ability of seniors to perform functional tasks.[6] They have received widespread attention and increased popularity.[7] The inherent assets of the elastic band accommodate the length-tension characteristics of normal joint and muscle actions.[8] With their feature-rich attributes, exercises can be designed and tailored for various populations with different levels of body functioning.[9,10]

Elastic band exercises have proven to be beneficial in both healthy and frail seniors.[11,12] A resistance training program using elastic tubing served as a practical and effective way of eliciting strength gains in adults older than 65 years.[13] In a study of 89 functionally limited seniors who participated in a 6-month in-home video-facilitated elastic band resistance exercise program, the experimental group had significant improvements in knee extension strength (16.7%) and hip extension (20.5%) strength.[11] Furthermore, Binder et al[14] found that 3 months of supervised progressive resistance training improved thigh muscle strength and whole body fat-free mass for community-dwelling sedentary seniors 78 years and older. Elastic band exercises also increased dynamic muscle strength in seniors with orthostatic hypotension[15] and improved the sit-to-stand performance of community-dwelling seniors.[16]

In addition, a modest intensity stretching exercise program increased the range of joint motions and flexibility of seniors.[17,18] Furthermore, Topp et al[19] found that after a 12-week dynamic resistance strength training program using elastic tubing, the community-dwelling seniors (mean age, 71.1 years) demonstrated slower gait velocity, enhanced balance, and improved ability to walk backward. The research group further examined another 14-week resistance training program (3 times per week using elastic bands) to determine its effects on ankle strength, training intensity, postural control, and gait velocity of 42 seniors (mean age, 72 years). After the training, the resistance group exhibited improved ankle dorsiflexion, training resistances, and gait velocity.[20] Finally, resistance training improved the functional ability of 131 seniors with limited body functioning.[21] It was also found that dynamic or isometric resistance training improved functional ability and reduced knee joint pain of patients with knee osteoarthritis.[12]

As supported by the literature, elastic band exercises are beneficial for seniors. However, among the reviewed studies, the protocol of carrying out the elastic band exercises for aged adults has not been clearly presented. Thus, the purposes of this study were as follows: (1) to develop a senior-tailored elastic band exercise program, (2) to evaluate the feasibility of the elastic band exercise program with a group of aged adults, and (3) to explore appropriate elastic band exercise frequencies and preferences of participants.

Methods

Design

This descriptive evaluation survey study had 2 phases. Phase I began with sending surveys to a panel of professional experts to develop a senior-tailored elastic band exercise program called the *Senior Elastic Band* (SEB) program. The SEB was developed initially by a certified exercise trainer who had been teaching physical fitness activities for 16 years and the principal investigator of the study who is a doctoral-prepared gerontologic nursing who has expertise in complementary/alternative therapy and has been conducting exercise intervention research for seniors for 10 years. A hard copy and a DVD containing detailed descriptions and demonstrations of the program were sent to the experts. The experts evaluated the SEB based on their best appraisal of simplicity, safety, suitability, and helpfulness of the protocol. Phase II inquired the seniors' perspectives on the SEB protocol using a descriptive design with quantitative and semistructured evaluations. Participants were interviewed individually after 1 month of the instructor-led SEB group practice, which was conducted 3 times per week, 40 minutes per session. The feedback from the seniors was further incorporated in the revision of the SEB program.

Setting and Participants

A panel of 11 experts was invited to participate in phase I of the study: 3 senior exercise trainers, 2 physical education scholars, 2 gerontologic nurse practitioners, 2 geriatric physicians, and 2 physical therapists. Owing to the preliminary feasibility testing of the seniors' feedback on the SEB program, phase II of the study was conducted in one senior day care center in southern Taiwan. Using a convenience sampling strategy, all eligible participants in that senior day care center were intended to be recruited, and a total of 20 senior participants participated (participation rate, 98%). Inclusion criteria were as follows: (1) community-dwelling seniors 65 years and older, (2) no previous training with elastic bands, (3) ability to stand alone without assistive devices, (4) cognitively alert (a score of ≥ 8 on the Short Portable Mental Status Questionnaire), and (5) independent or mild dependency for self-care indicated by a Barthel Index score of 91 or higher. Participants with dementia, wheelchair users, and those with severe or acute cardiovascular, musculoskeletal, or pulmonary illnesses were excluded because of the self-report format and the nature of elastic band exercises.

Data Collection

In phase I, the preliminary SEB exercise program, including hard copy descriptions and DVD demonstrations of each exercise, was sent to the advisory panel for their critique and evaluation. The 11 experts were asked to rate

each exercise on the 4 criteria: (1) simplicity, (2) safety, (3) suitability, and (4) helpfulness. Simplicity is concerned with the difficulty level for seniors to perform a particular exercise; safety focuses on whether the exercise is safe and not dangerous for seniors to practice; suitability refers to whether or not the particular exercise is appropriate for and capable of being practiced by seniors; helpfulness asks whether the exercise is helpful in promoting the health of seniors.[22] For each evaluation criterion, experts rated each exercise on a scale from 1 to 4: 1 means the exercise is very difficult, very dangerous, extremely inappropriate, not good for health promotion, and should be deleted; 2 means major revisions should be made to the exercise; 3 means the exercise is fine but warrants minor revision; and 4 means the exercise is simple, safe, suitable, good for health promotion, and very essential and should not be omitted.[22] The experts were asked to provide suggestions for revisions if they rated the exercise with a score of 3 or less. Cronbach α coefficients for the 4 criteria of the evaluation form were .93, .93, .94, and .94, respectively.

In phase II, 20 participants were interviewed individually to evaluate the feasibility of the SEB exercise program. Similarly, participants were asked to rate the level of simplicity, safety, suitability, and helpfulness of the program. Each criterion was rated on a 10-point Cantril ladder scale,[23] ranging from 0 to 10, with 0 meaning it was very difficult to perform, very dangerous, very inappropriate, and useless in contributing to the seniors' health and 10 meaning it was very easy, very safe, very appropriate, and very helpful to the seniors' health. Finally, participants were asked to reflect on their experiences in the SEB program based on the 5 open-ended questions: (1) How do you feel after 1 month of performing the SEB exercises? (2) How many times per week of elastic band exercise is appropriate? (3) What length of time per exercise session is appropriate? (4) How many people per group are appropriate in doing elastic band exercises? (5) What type of instructor do you prefer to lead the group and learn the elastic band exercise from? The researcher delivered the questions verbally to the participants and marked their verbal answers on the evaluation forms. The seniors' reactions, comments, and suggestions were taken into consideration in revising the SEB exercise program. Cronbach α coefficients for the 4 criteria of the evaluation form were .62, .59, .79, and .91, respectively.

Ethical Considerations

This study was approved by the institutional review board of the Fooyin University Hospital (FYH-IRB-098-12-02) and funded by the National Science Council, Taiwan (NSC 97-2314-B-037-052-MY3). After the approval by the administrator of the day care center, a list of names of those seniors willing to participate was obtained from the staff, and their written informed consents were obtained. It was emphasized that participation in this study was voluntary and participants were free to withdraw at anytime.

Data Analysis

The Statistical Package for the Social Sciences (SPSS) Version 17.0 (Sinter Information Corp, Taiwan, China) was used to analyze the data. The quantitative evaluation results of the experts were analyzed using descriptive statistics of mean and SD to determine the consensus of the experts on each elastic band exercise. The qualitative revision suggestions of these experts were critically analyzed and summarized using content analysis to revise the program. Descriptive statistics such as mean, SD, frequency, and percentage were used to describe the characteristics of the senior participants and their ratings on the program. Qualitative responses were summarized into categories and presented using frequency distributions.

Results

Demographics of the Participants

The demographic profiles of the experts in phase I (N = 11) and the seniors in phase II (N = 20) were presented in Table 1. The characteristics of the aged adult participants were similar to the community-dwelling seniors in general.

Results of Aim 1: To develop a SEB Program. The preliminary SEB protocol had 3 phases: (1) warm-up, (2) aerobic motions, and (3) static stretching. The entire program took 40 minutes to complete. Descriptions and diagrams of some key movements are provided in Table 2 and Figure 1.

Warm-up. Seven movements were designed to loosen up the body and elevate the energy of participants for a safe transition to the next phase: (1) turning the wrists, (2) pulling the arms, (3) taking a bath, (4) lifting a weight, (5) drawing a circle, (6) stepping on the floor, and (7) twisting the waist. The mean ratings of the experts on the 4 criteria were mostly above 3, except that the mean (SD) score on safety for "twisting the waist" was 2.73 (1.10) (Table 3). In the movement of twisting the waist, the experts suggested that the continuous twisting motion of the waist be changed to holding the twisting position for 5 seconds and then releasing to prevent possible harm to the waist in the fast twisting motion. Furthermore, the experts suggested that seniors bend their knees and elbows slightly to protect their joints. The experts also suggested a name change for the following 3 movements to better fit the descriptions of the movements: (1) changing "lifting the weight" to "bending the knees," (2) changing "drawing a circle" to "turning the buttocks," and (3) changing "stepping on the floor" to "raising the legs."

Aerobic motions. Seven low-to-medium speed exercises were incorporated in this phase to enhance the cardiovascular-respiratory workout: (1) spreading the wings, (2)

Table 1. *Demographics of the participants*

	Phase I experts (n = 11)			Phase II seniors (n = 20)		
Variables	n	%	Mean ± SD	n	%	Mean ± SD
Age (y)			49.96 ± 11.65			72.00 ± 5.12
≤64	9	81.82		0	0.00	
65-74	2	18.18		16	80.00	
75-84	0	0.00		3	15.00	
≥85	0	0.00		1	5.00	
Sex						
Male	4	36.36		2	10.00	
Female	7	63.64		18	90.00	
Education						
None	0	0.00		6	30.00	
Elementary	0	0.00		7	35.00	
High school	2	18.18		6	30.00	
College	2	18.18		1	5.00	
Graduate school	7	63.64		0	0.00	
Working with seniors (y)			11.84 ± 7.20			–
Marital status						
Married	–	–		6	30.00	
Widowed	–	–		14	70.00	
Living arrangement						
Alone	–	–		7	35.00	
With family	–	–		13	65.00	
Exercise habit (d/wk)			–			4.55 ± 3.19
Yes	–	–		14	70.00	
No	–	–		6	30.00	
Chronic illness			–			0.95 ± 0.76
Yes	–	–		14	70.00	
No	–	–		6	30.00	
Cognitive function [a]			–			9.75 ± 0.55
Self-care ability [b]			–			100.00 ± 0.00

– Indicates that data were not collected.
[a] Cognitive functional was measured by the Short Portable Mental Status Questionnaire.
[b] Self-care ability was measured by the Barthel Index.

boxing, (3) swinging the arms, (4) squeezing the belly, (5) twisting the hips, (6) shaking the body, and (7) jumping and waving. Mean scores of the experts' ratings on the 4 criteria were mostly above 3, except that the mean score on the simplicity and safety of "squeezing the belly" was 2.91, and the mean score on the safety of "twisting the hips" was 2.91. On the other hand, the movement of "swinging the arms" received a score of 4 on the criteria of simplicity, suitability, and helpfulness (Table 3). The experts pointed out that the movements of squeezing the belly and twisting the hip were either too difficult or not completely safe for seniors to perform; therefore, these 2 movements were eliminated. Based on the experts' suggestions, 2 movements were added: (1) raising hands: train the muscle strength and endurance of the arms and (2) whipping out a sword: enhancing trunk rotation and muscle strength of the arms. For safety reasons, the movement of "jumping and waving" was changed to "stepping and pushing." However, because all of the experts thought the movement of swinging the arms was too easy to perform, the movement was changed to "shooting an arrow": bend the elbow and pull the arm after swinging. Finally, the experts again suggested that seniors bend their knees and elbows slightly in most of these fast movements to protect the joints.

Static stretching. Seven low-speed, gentle stretching exercises were incorporated in this phase to build up muscle power/endurance and increase range of motion and flexibility: (1) directing traffic, (2) picking an apple, (3) attacking the front, (4) turning eight, (5) opening arms, (6) touching knees, and (7) rotating shoulders. Mean values of the experts' ratings on the 4 criteria were mostly above 3, except that the mean (SD) score on simplicity for "turning eight" was 2.91 (0.83), and the mean (SD) score on safety for "rotating shoulders" was 2.73 (1.42) (Table 3). The experts suggested that the turning-eight exercise was too complicated to perform and that rotating shoulders was too dangerous. Therefore, these 2 movements were eliminated. In addition, one new movement called "stretching the sides" was suggested by the experts and was added in the protocol to enhance body flexibility.

Results of Aim 2: To Evaluate the Feasibility of the SEB Program With a Group of Seniors. Participants' mean ratings of the simplicity, safety, suitability, and helpfulness of the revised SEB program ranged from 9 ± 1.08 to 9.90 ± 0.45, indicating that

Table 2. *Descriptions of the first movement in each phase of the SEB*

Phase: movement	Descriptions
Warm-up: turning the wrists	1. Stand with legs open as wide as the shoulders, slightly bend the knees, raise hands in the front as high as the shoulders, and slightly bend the elbows.<br>2. Turn the left forearm and wrist from inward to outward for 10 times.<br>3. Same procedure to the right-hand side for 10 times<br>4. Turn both forearms and wrists from inward to outward for 10 times.
Aerobic motions: spreading the wings	1. Stand with legs close, put hands on the sides of the body, bend the elbows as 90°, and hold the elastic band with hands in front of the chest.<br>2. Left foot steps forward, the heel of the left foot touches the floor, raise the arms on the sides of the body as high as the shoulders, and hands pull the elastic band outward at the same time<br>3. Return to the beginning position.<br>4. Same procedure to the right foot<br>5. Alternate the left and right feet for 4 eight-beat time.
Static stretching: directing traffic	1. Stand with legs close, raise arms up-high, open arms wider than the shoulders, and hold the elastic band with hands.<br>2. Step left foot forward to the left side (45°), put the right arm up-high and left arm to the left (90°), turn the body to the left and backward (exhale); hold the position for 6 s; inhale, turn the body back to the front, and put the left foot back.<br>3. Same procedure to the right-hand side

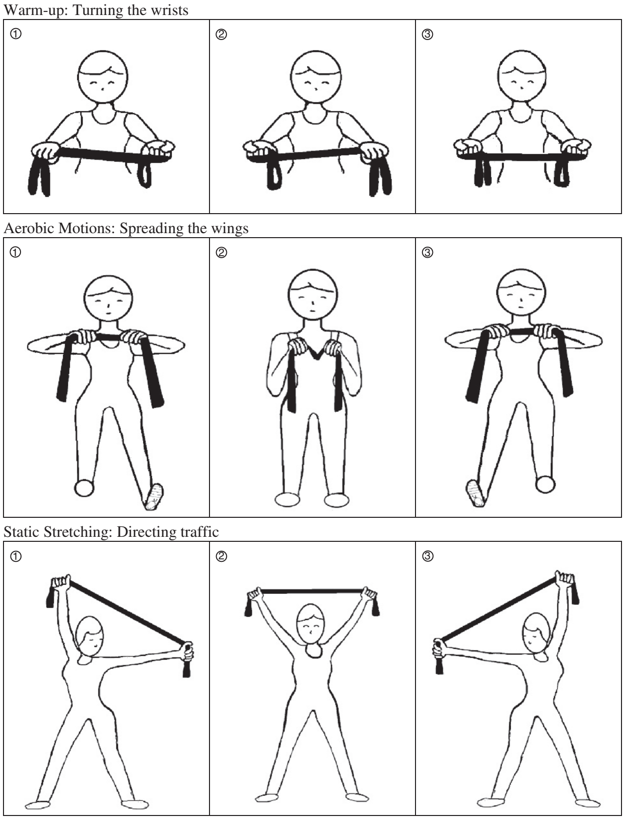

Fig 1. *Diagrams of the first movement in each phase of the SEB.*

the program was feasible, safe, suitable, and helpful to the seniors (Table 4). The adjusted movements and the added movements based on the experts' suggestions were acceptable by the senior participants. The seniors revealed that the postures in the static stretching phase were somewhat challenging, especially when they held the static posture. However, those postures were tolerable and they got better after each practice. Therefore, no changes will be made to the SEB program before it is used in an experimental-control study. In terms of the participants' feedback toward the SEB, the participants reported that their body flexibility and range of joint motions increased (n = 15), that they felt energetic and had more strength in daily activities (n = 12), and that they slept better at night (n = 15). Most of them (n = 17) promised that they would continue doing SEB exercise; moreover, all of them would recommend their friends and families to join a SEB program (n = 20).

Results of Aim 3: To Explore Appropriate SEB Exercise Frequencies and Preferences for Seniors. Most participants preferred to practice elastic band exercises 3 times per week (n = 15) to better promote their health. Some preferred doing them at least 5 times per week (n = 2) or even practicing them every day (n = 3). In terms of exercise program duration, most (n = 18) felt that an hour would be an appropriate length. Most participants preferred to do the elastic band exercises in a large group of 20 to 29 people (n = 10), and no one wanted to exercise alone. Furthermore, more than half of the participants preferred to have a female instructor to lead them exercise (n = 11). Most of them also preferred that the age of the instructor be between 40 and 49 years (n = 12). Finally, they only had 2 major concerns in terms of the characteristics of the instructor: (1) the instructor must have patience with the learners (n = 20), and (2) it would be better if the instructor was someone with whom they were familiar (n = 12) (Table 5).

Discussion

After the evaluations of 11 experts and 20 community senior participants, the final SEB exercise program included 3 phases with 20 movements: (1) 7 warm-up movements (12 minutes), (2) 7 aerobic motions (10 minutes), and (3) 6

Table 3. *Ratings of the experts on each movement of the preliminary SEB exercise program (n = 11)*

Criteria phases/movemenfsts	Simplicity, mean ± SD	Safety, mean ± SD	Suitability, mean ± SD	Helpfulness, mean ± SD
Warm-up				
1. Turning the wrists	3.91 ± 0.30	3.73 ± 0.65	3.82 ± 0.40	3.82 ± 0.40
2. Pulling the arms	3.55 ± 0.69	3.09 ± 1.22	3.27 ± 1.01	3.45 ± 0.93
3. Taking a bath	3.45 ± 0.82	3.36 ± 0.81	3.55 ± 0.82	3.55 ± 0.82
4. Lifting a weight	3.36 ± 0.92	3.36 ± 0.92	3.55 ± 0.93	3.55 ± 0.93
5. Drawing a circle	3.73 ± 0.47	3.73 ± 0.47	3.73 ± 0.47	3.45 ± 1.04
6. Stepping on the floor	3.91 ± 0.30	3.82 ± 0.40	4.00 ± 0.00	3.73 ± 0.90
7. Twisting the waist	3.18 ± 0.98	2.73 ± 1.10	3.09 ± 0.94	3.36 ± 1.03
Aerobic motions				
1. Spreading the wings	3.73 ± 0.47	3.64 ± 0.50	3.82 ± 0.40	3.91 ± 0.30
2. Boxing	3.55 ± 0.69	3.45 ± 0.69	3.73 ± 0.65	3.64 ± 0.67
3. Swinging the arms	4.00 ± 0.00	3.82 ± 0.40	4.00 ± 0.00	4.00 ± 0.00
4. Squeezing the belly	2.91 ± 1.04	2.91 ± 1.22	3.27 ± 1.19	3.09 ± 1.14
5. Twisting the hips	3.27 ± 1.01	2.91 ± 1.04	3.09 ± 1.04	3.18 ± 1.08
6. Shaking the body	3.18 ± 0.98	3.09 ± 1.14	3.09 ± 1.04	3.18 ± 1.08
7. Jumping and waving	3.36 ± 0.67	3.18 ± 1.08	3.55 ± 0.69	3.64 ± 0.50
Static stretching				
1. Directing traffic	3.45 ± 1.04	3.18 ± 1.17	3.36 ± 1.21	3.36 ± 1.21
2. Picking an apple	3.73 ± 0.90	3.45 ± 0.93	3.64 ± 0.92	3.73 ± 0.90
3. Attacking the front	3.45 ± 0.69	3.64 ± 0.50	3.64 ± 0.50	3.73 ± 0.47
4. Turning eight	2.91 ± 0.83	3.18 ± 0.98	3.18 ± 0.98	3.45 ± 1.04
5. Opening arms	3.55 ± 0.69	3.36 ± 1.03	3.64 ± 0.67	3.73 ± 0.65
6. Touching knees	3.55 ± 0.69	3.45 ± 0.82	3.73 ± 0.65	3.73 ± 0.65
7. Rotating shoulders	3.09 ± 1.22	2.73 ± 1.42	3.09 ± 1.22	3.09 ± 1.38

Table 4. *Ratings of the participants on each phase of the SEB (n = 20)*

Criteria phases	Simplicity, mean ± SD	Safety, mean ± SD	Suitability, mean ± SD	Helpfulness, mean ± SD
Warm-up	9.75 ± 0.55	9.70 ± 0.57	9.90 ± 0.45	9.40 ± 0.99
Aerobic motions	9.05 ± 1.15	9.80 ± 0.52	9.75 ± 0.64	9.45 ± 1.10
Static stretching	9.00 ± 1.08	9.50 ± 0.89	9.45 ± 1.15	9.40 ± 1.14

static stretching postures (18 minutes). The program took 40 minutes to complete. In addition to the minor revisions of some movements suggested by the experts, they also recommended the deletion of 4 movements and the addition of 3 movements. Because the experts' evaluations and recommendations were consistent, the revised SEB was not send out to the experts for a second round of evaluations. In phase II, the community seniors had positive evaluations toward the revised SEB, as indicated by at least a score of 9 of 10 on every criterion of each phase of the program. As suggested by Chen et al,[22] it is important that exercise programs for seniors be manageable but also contain a degree of challenge so that seniors have an opportunity for improvement, a sense of overcoming a challenge, and a chance to promote self-efficacy. In the preliminary SEB, the movement of "swing the arms" in the aerobic motions got a full score of 4 on the criterion of simplicity. The research team decided to add a little challenge to the movement and changed it to shooting an arrow. On the other hand, squeezing the belly in aerobic motions and turning eight in static stretching were either too complicated to learn or not safe enough to perform; thus, these movements were eliminated from the program. According to Chen et al,[24] if the exercise programs are too complicated to learn or perform, they are likely to discourage senior learners, lower self-esteem, diminish interest, and decrease participation. Therefore, it is essential to find a balance between building seniors' self-confidence and providing an opportunity for self-improvement.

After 1 month of the SEB exercises, participants gave positive feedback about the program, such as self-expressions of enhanced body flexibility and range of joint motions; became energetic; had more strength in performing daily activities; and slept better at night. The enhanced body flexibility and range of joint motions were congruent with previous studies[17,18]; the improvement of daily functional abilities was consistent with the other reports.[6,21] However, the beneficial effects of muscle strength, endurance, gait, and balance reported in the literature were not supported by this pilot study. Possibly a short-term exercise program length of 1 month is not long enough to manifest these improvements. Besides, these beneficial effects were only self-expressions from the participants and not formally tested. A formal evaluation of the long-term SEB program effects would be essential in future studies.

Table 5. *Participants preferences for the elastic band exercises (n = 20)*

Variables	*f*
Exercise frequency (times/wk)	
3	15
5	2
7	3
Exercise duration (min/session)	
30	2
60	18
Group size (no. of people)	
10-19	5
20-29	10
30-39	5
Sex of the instructor	
Female	11
No preference	9
Age of the instructor (y)	
40-49	12
50-55	8
Characteristics of the instructor	
Patience	20
Someone familiar	12

In general, the SEB program was well accepted by this group of community seniors because most of them expressed a willingness to continue practicing SEB and all of them would recommend their families and friends to join the program. They suggested practicing SEB 3 times per week for 60 minutes per sessions in large groups of 20 to 29 persons. Results were somewhat similar to a newly published study of comparing the yoga exercise preferences of community and institutional seniors. Results indicated that community seniors preferred to practice yoga 61 to 90 minutes every day in a group of 11 to 20 persons, whereas the institutional seniors preferred to practice yoga 31 to 60 minutes 3 times per week in a group of less than 10.[25] However, in the study of Chen et al,[25] age and sex did not appear to be the key issues for community and institutional seniors. The community seniors cared the most about whether the instructor was professionally trained, and the institutional seniors were concerned the most about whether the instructor was considerate and cared about their feelings. On the contrary, this sample of community seniors preferred to have a female instructor in the ages of 40 to 49 years. Professional training of the instructor was not an issue, but they would like to have someone with whom they were familiar and who had patience to lead the exercise group. The possible reason might be that they had very positive experiences with the current elastic band instructor; therefore, they projected the characteristics of the current instructor.

Finally, the SEB program was different from traditional elastic band exercises in various ways. The movements in the SEB were less strenuous to accommodate reduced body flexibility and strength experienced by many seniors. Most of the movements in the program emphasized bending the knees and elbows slightly to protect joins. Furthermore, the thickness of the elastic band was chosen as medium so that the level of resistance training could be easily increased or decreased to accommodate the muscle strength of seniors.

Limitations and Future Studies

Although an evaluation survey was used to gather the opinions of experts on the SEB program, only 11 experts with 5 different professional backgrounds were consulted and the revisions made to the program were not validated by the experts. It is possible that the opinions of the experts in this study were not fully expressed, or valuable suggestions and essential comments from experts with other professional backgrounds were not considered in developing the SEB. Furthermore, a small convenience sample, rather than a large probability sample, was used in phase II, which may have resulted in selection bias and limited generalizability of results. In addition, most of the participants were regular exercisers, which might have confounded the results and weakened their interpretations. Finally, the beneficial effects after 1 month of SEB practicing revealed by the participants were only self-expressions and not formally tested. These results should be interpreted with caution.

The beneficial effects of the SEB should be further tested in a large random sample of community seniors. The valuable information provided by the senior participants on the SEB implementation protocol may be helpful in arranging and promoting this exercise program.

Conclusion

The SEB program was supported by the 11 experts with 5 professional backgrounds and was well accepted by a small group of community seniors. The senior participants expressed that the program was feasible, was manageable, and could be helpful to their health promotion.

Practical Applications

- The SEB exercise program included 3 phases (warm-up, aerobic motions, and static stretching) with 20 movements.
- The community participants rated the SEB highly and commented that the program was feasible, safe, suitable, and helpful.
- The SEB could be applied to seniors 3 times per week and 60 minutes per session for their health promotion, maintenance, or rehabilitation.

Funding Sources and Potential Conflicts of Interest

This study was funded by the National Science Council, Taiwan (NSC 97-2314-B-037-052-MY3). The sponsor

approved the study design and supported the study financially. The sponsor did not interfere with the study procedure. No conflicts of interest were reported for this study.

Contributorship

Concept development (provided idea for the research): KMC, WST, HTH

Design (planned the methods to generate the results): KMC, WST

Supervision (provided oversight and was responsible for organization and implementation and writing of the manuscript): KMC

Data collection/processing (were responsible for experiments, patient management, organization, or reporting data): WST, HTH, CHL

Analysis/Interpretation (were responsible for statistical analysis, evaluation, and presentation of the results): KMC, CHL

Literature search (performed the literature search): KMC, HTH

Writing (was responsible for writing a substantive part of the manuscript): KMC

Critical review (revised the manuscript for intellectual content, this does not relate to spelling and grammar checking): KMC

Other (list other specific novel contributions)

References

1. Chen KM, Huang HT, Lin MH, Wang YC, Li CH, Hsu PC. Physical health of community-dwelling older adults: live longer and stay healthier. In: *Proceedings of the 41st Biennial Convention of Sigma Theta Tau International: 29 October–2 November 2011; Grapevine, TX*. Edited by Sigma Theta Tau International; 2011:52.
2. Chodzko-Zajko W. National blueprint to increase physical activity among adults age 50 and over. Illinois: University of Illinois at Urbana-Champaign; 2003.
3. U.S. Department of Health and Human Services. Health, United States. With chart book on trends in the health of Americans. Hyattsville, MD: Department of Health and Human Services; 2003.
4. Brown AB, McCartney N, Sale DS. Positive adaptations to weight-lifting training in the elderly. J Appl Physiol 1990;69: 1725-33.
5. Hess JA, Woollacott M. Effect of high-intensity strength-training on functional measures of balance ability in balance-impaired older adults. J Manipulative Physiol Ther 2005;28: 582-90.
6. Galvao DA, Taaffe DR. Resistance exercise dosage in older adults: single versus multisite effects on physical performance and body composition. J Am Geriatr Soc 2005;53:2090-7.
7. Ghigiarelli JJ, Nagle EF, Gross FL, Robertson RJ, Irrgang JJ, Myslinski T. The effects of a 7-week heavy elastic band and weight chain program on upper-body strength and upper-body power in a sample of division 1-AA football players. J Strength Cond Res 2009;23:756-64.
8. Patterson RM, Jansen CWS, Hogan HA, Nassif MD. Material properties of thera-band tubing. Phys Ther 2001; 81:1437-45.
9. Newton RU, Hakkinen K, Hakkinen A, McCormick M, Volek J, Kraemer WJ. Mixed methods resistance training increases power and strength of young and older men. Med Sci Sports Exerc 2002;34:1367-75.
10. Damush TM, Damush JG. The effects of strength training on strength and health-related quality of life in older adult women. Gerontologist 1999;39:705-10.
11. Dancewicz TM, Krebs DE, McGibbon CA. Lower-limb extensor power and lifting characteristics in disabled elders. J Rehabil Res Dev 2003;40:337-48.
12. Topp R, Woolley S, Hornyak J, Khuder S, Kahaleh B. The effect of dynamic versus isometric resistance training on pain and functioning among adults with osteoarthritis of the knee. Arch Phys Med Rehabil 2002;83:1187-95.
13. Manor B, Topp R, Page P. Validity and reliability of measurements of elbow flexion strength obtained from older adults using elastic bands. J Geriatr Phys Ther 2006;29:16-9.
14. Binder EF, Yarasheski KE, Steger-May K, et al. Effects of progressive resistance training on body composition in frail older adults: results of a randomized, controlled trial. J Gerontol: Med Sci 2005;60A:1425-31.
15. Zion AS, DeMeersman R, Diamond BE, Bloomfield DM. A home-based resistance-training program using elastic for elderly patients with orthostatic hypotension. Clin Auton Res 2003;13:286-92.
16. Chen TA, Wu YT, Lee MB, Liang KC, Lin KN, Tsai MW. Effects of exercise on depression symptoms, physical function, and quality of life in community-dwelling elderly. Formos J Phys Ther 2009;34:209-18.
17. Swank AM, Funk DC, Durham MP, Roberts S. Adding weights to stretching exercise increases passive range of motion for healthy elderly. J Strength Cond Res 2003;17:374-8.
18. Sugimoto D, Blanpied P. Flexible foil exercise and shoulder internal and external rotation strength. J Athl Training 2006; 41:280-5.
19. Topp R, Mikesky A, Wigglesworth J, Holt W, Edwards JE. The effect of a 12-week dynamic resistance strength training program on gait velocity and balance of older adults. Gerontologist 1993;33:501-6.
20. Topp R, Mikesky A, Dayhoff NE, Holt W. Effect of resistance training on strength, postural control, and gait velocity among older adults. Clin Nurs Res 1996;5:407-27.
21. Topp R, Boardley D, Morgan AL, Fahlman M, McNevin N. Exercise and functional tasks among adults who are functionally limited. West J Nurs Res 2005;27:252-70.
22. Chen KM, Tseng WS, Ting LF, Huang GF. Development and evaluation of a yoga exercise programme for older adults. J Adv Nurs 2007;57:432-41.
23. Cantril H, Kilpatrick FP. Self-anchoring scaling: a measure of individuals' unique reality worlds. J Indiv Psychol 1960;16: 158-73.
24. Chen KM, Chen WT, Huang MF. Development of the Simplified Tai-Chi Exercise Program (STEP) for frail older adults. Complement Ther Med 2006;14:200-6.
25. Chen KM, Wang HH, Li CH. Community vs. institutional elders' evaluations of and preferences for yoga exercises. J Clin Nurs 2011;20:1000-7.
