# Supplementary material for: Effects of elastic band resistance training on the physical and mental health of elderly individuals: A mixed methods systematic review
Source: PLoS One. 2024 May 13;19(5):e0303372. doi: 10.1371/journal.pone.0303372 (PMC11090353; doi:10.1371/journal.pone.0303372)
Supplement: S1 File — (ZIP) [file pone.0303372.s001.zip › Supporting Information/Included study 40.pdf]

International Journal of Nursing Studies 55 (2016) 4–14

Contents lists available at ScienceDirect

International Journal of Nursing Studies

journal homepage: www.elsevier.com/ijns

Feasible modalities and long-term effects of elastic band exercises in nursing home older adults in wheelchairs: A cluster randomized controlled trial

Kuei-Min Chen [a,*], Chun-Huw Li [b,1], Hsin-Ting Huang [a,2], Yin-Yin Cheng [a,2]

[a] College of Nursing, Kaohsiung Medical University, 100 Shih-Chuan 1st Rd., Sanmin District, Kaohsiung 80708, Taiwan
[b] Department of Nursing, Yuhing Junior College of Health Care and Management, No. 15, Lane 420, Dachang 2nd Rd., Sanmin District, Kaohsiung 80776, Taiwan

ARTICLE INFO

Article history:
Received 27 April 2015
Received in revised form 19 November 2015
Accepted 22 November 2015

Keywords:
Exercise
Frail elderly
Nursing homes
Randomized controlled trial
Resistance training
Wheelchairs

ABSTRACT

Background: Physical activity holds promise for mobility-impaired older adults to prevent further disabilities and improve their health. However, staffing constraints have made it challenging to promote physical activity in long-term care facilities.
Objectives: To test the feasibility and effects of 12 months Wheelchair-bound Senior Elastic Band (WSEB) group-exercises that were led by volunteers for the first six months followed by the DVD-guided for another six months on functional fitness, activities of daily living (ADL), and sleep quality of nursing home older adults in wheelchairs.
Design: Cluster randomized controlled trial with two groups, pre-test and post-tests.
Settings: Ten nursing homes, Taiwan.
Participants: 127 participants participated voluntarily; 107 of them completed the study. Inclusion criteria: (1) aged 65 years and over, (2) using wheelchairs for mobility, (3) living in facility for at least three months, (4) cognitively intact, and (5) heavy or moderate dependency in ADL. Majority of participants were middle-old older adults (75–84 years old, 53.2%), female (51.4%), and had chronic illnesses (98.1%).
Methods: Participants were randomly assigned by facility to either the experimental (five nursing homes, $n = 56$) or control group (five nursing homes, $n = 51$). The WSEB program was conducted three times per week and 40 min per session in two stages: volunteer-led for the first six months (stage I) followed by the DVD-guided modality for another six months (stage II). The primary outcomes (functional fitness: lung capacity, body flexibility, range of joint motion, and muscle strength and endurance) and the secondary outcomes (ADL measured by the Barthel Index; sleep quality measured by the Pittsburgh Sleep Quality Index) of the participants were measured at three time points: pre-test, at the six-month interval, and at the end of 12 months of the study. No blinding was applied.
Results: All of the functional fitness indicators of the experimental group participants improved significantly ($p < .05$), and were all better than the control group at six-month and 12-month of the study ($p < .05$). No symptoms of discomfort occurred during interventions.

* Corresponding author. Tel.: +886 7 3136900.
E-mail addresses: kmc@kmu.edu.tw (K.-M. Chen), numa@ms.yuhing.edu.tw (C.-H. Li), qwety789@livemail.tw (H.-T. Huang), kuyin3641@yahoo.com.tw (Y.-Y. Cheng).
[1] Tel.: +886 917665157.
[2] Tel.: +886 7 3136900.

http://dx.doi.org/10.1016/j.ijnurstu.2015.11.004
0020-7489/© 2015 Elsevier Ltd. All rights reserved.

Conclusions: Nursing home older adults in wheelchairs who received WSEB exercise training had better functional fitness, ADL, and sleep quality than those who did not. It was a feasible way of carrying out this exercise program by using the volunteer-led followed by the DVD-guided modalities. The program can be applied in institutional settings routinely.

© 2015 Elsevier Ltd. All rights reserved.

What is already known about the topic?

- Physical activity holds promise for mobility-impaired older adults to prevent further disabilities and improve their overall health. However, staffing constraints have made it challenging to promote physical activity in long-term care facilities.
- Trained volunteers could help nursing home older adults to engage in more physical activity and DVD-delivered exercise programs can produce clinically meaningful gains in physical functions that are maintained beyond intervention termination.
- Resistance training, such as elastic band exercises, can prevent the decline of muscular strength, which is a commonly seen problem in older adults, and has been proven to be beneficial in both healthy and frail older adults.

What this paper adds

- Nursing home older adults in wheelchairs who received Wheelchair-bound Senior Elastic Band exercise training had better functional fitness, activities of daily living, and sleep quality than those who did not.
- The positive effects of the Wheelchair-bound Senior Elastic Band exercise program occurred after six months of volunteer-led exercises and were maintained or even enhanced through the following six months of DVD-guided practices.
- The modality of being volunteer-led followed by the modality of being DVD-guided was a feasible way of carrying out the Wheelchair-bound Senior Elastic Band program in institutional settings, routinely and longitudinally.

1. Introduction

Older adults who live in long-term care facilities are increasing in number drastically with age (U.S. Department of Health and Human Services, 2013), and the ratio of their activities of daily living (ADL) dependency was increased from 3.9% in 2004 to 4.2% in 2014 (American Health Care Association, 2014). Over 50% of Canadian nursing home older adults sit in a wheelchair every day (Clarke et al., 2009), and the factors associated with the use of wheelchairs by ambulatory older adults included fear of falling during walking, muscle weakness, and impaired balance (Chen et al., 2013). Shore (2008) reported that 12.3% of older adults in wheelchairs experienced worsening health, and wheelchair-use has been associated with many other adverse outcomes, such as deconditioning, falls, discomfort, loss of independence, social isolation, and sleep disturbances (Chen et al., 2015a; Gavin-Dreschnack et al., 2010). Being sedentary may result in a decline in physical fitness, and lead to a spiral of inactivity and further decline (Ikezoe et al., 2013).

Physical activity holds promise for mobility-impaired older adults to prevent further disabilities and improve their overall health (World Health Organization, 2014). However, staffing constraints have made it challenging to promote physical activity in long-term care facilities since older adults are becoming older, sicker, and require more assistance with their ADL after being admitted to the facilities (Benjamin et al., 2011; Wang and Tsay, 2012). Volunteers would be an alternative resource to help nursing home older adults to engage in more physical activity. However, volunteers should receive proper training, and feasible modalities should be applied to carry out the activities (Van der Ploeg et al., 2014).

In terms of the feasible modalities used in physical activities for older adults, Froehlich-Grobe et al. (2014) found that the staff-supported group exercise approach holds promise for encouraging exercise among inactive wheelchair users. Compared to individual exercises, group exercises resulted in higher levels of interest and exercise participation, brought more positive emotional effects (e.g., increased social interaction and sense of personal accomplishment), and less financial and time burdens (Shin et al., 2007). Socialization and self-efficacy are key motivators for older adults to initiate and maintain exercise programs (Phillips et al., 2004). Group-based exercise contributes to a higher adherence rate (Martin et al., 2013). In addition, a DVD-delivered exercise program can produce clinically meaningful gains in physical functioning that are maintained beyond intervention termination (Wójcicki et al., 2014). The DVD technology had a positive effect on exercise compliance (Kingston et al., 2010), eased the workload of busy providers (Khan et al., 2011), and can be applied on a broad scale (McAuley et al., 2003). It is especially beneficial when applied in multicenter-based exercise training programs to reduce cost and preserve resources (Gothe et al., 2015).

Among various types of physical activities, resistance training can prevent the decline of muscular strength, which is a commonly seen problem in older adults (Burton and Sumukadas, 2010). Elastic band exercise is considered as an effective and safe resistance type of exercise (So et al., 2013). With its feature-rich characteristic, exercises can be designed for different purposes (Newton et al., 2002), even

for older adults with mobility limitations (Webber and Porter, 2010). Studies indicated that elastic band exercises improved muscle strength (Chen et al., 2015b; Latham and Liu, 2010), promoted muscle hypertrophy (Yasuda et al., 2014), enhanced lower-extremity function (Chang et al., 2012; Chen et al., 2009), and shortened movement time in older adults (Webber and Porter, 2010). Further, elastic band exercises improved functional ability (Topp et al., 2005), increased flexibility and range of joint motion (Sugimoto and Blanpied, 2006; Swank et al., 2003), and improved the overall physical fitness of older adults (So et al., 2013).

In a large government-funded, three-year research project, we developed an elastic band exercise program for older adults in wheelchairs in the first phase of the project, called the Wheelchair-bound Senior Elastic Band (WSEB) exercise program (Chen et al., 2013). The WSEB program was tested in the second phase of the project with positive effects of improving functional fitness, enhancing sleep quality, and decreasing depression of nursing home older adults in wheelchairs after six months of group exercises (Chen et al., 2015a,b). However, the WSEB program was not able to go beyond the six months of practice and become a daily activity in long-term care facilities due to the lack of sufficient and stable personnel to lead the program, which was a commonly obstacle in many long-term care facilities (Benjamin et al., 2014; Chen et al., 2015b). As suggested by Chen et al. (2013), the WSEB exercise program has to be guided by a trained and certified instructor initially; once a person has a familiarity with the program, different modalities of practice could be possible for long-term practice. Therefore, all of the participants in the second phase of the project entered into the third phase and continued their intervention with the modality of DVD-guided group practice of the WSEB exercise program.

The purpose of this study was to test the modality feasibility and long-term effects of WSEB group-exercises for 12 months on the effects of functional fitness, ADL, and sleep quality of nursing home older adults in wheelchairs; this modality was led by volunteer instructors for the first six months followed by the DVD-guided modality for another six months. We hypothesized that the positive effects of the WSEB program would occur after six months of volunteer-led exercises and would be maintained throughout the following six months of the DVD-guided exercises.

2. Materials and methods

2.1. Design and justifications

A cluster randomized controlled trial using two groups along with pre-test and post-tests was conducted (ClinicalTrials.gov identifier: NCT02165150). Ten nursing homes participated voluntarily and were randomly assigned to either the experimental or control group using a black box drawing. A cluster-randomization, instead of an individual-randomization, was adopted to reduce possible contamination among participants if one nursing home had both experimental and control groups participants. The intervention (WSEB program) was conducted with two modalities in two stages: volunteer-led for the first six months (stage I) followed by the DVD-guided modality for another six months (stage II). For the safety of participants and as suggested by Chen et al. (2013), the sequence of these two stages was essential, and it was not reasonable for these two modalities to be designed as two experimental groups. Data were collected at three time-points to detect the trend of changes: pre-test, at six months, and at 12 months of the study. Although the intervention lasted for 15 months (six months volunteer-led and nine months DVD-guided) in this large longitudinal research project, to keep the comparison interval consistent, the same interval of six months data of DVD-guided phase were used for the comparisons.

2.2. Setting and participants

This study was conducted in 10 community-based, privately funded, but government-accredited nursing homes. There are 64 nursing homes in Kaohsiung, southern Taiwan. The study sites resemble the majority of nursing homes in southern Taiwan that are small in scale (less than 49 beds) and are scattered throughout the communities to allow the frequent family visits to the elderly residents. Based on the statistical software Sample Power 2.0 (SPSS Inc., Chicago, IL) with the statistical ANCOVA, the required sample size was 56 participants per group (power = .80; alpha = .05; R-square of covariate in medium level .13; effect size in medium level .25). The parameter of medium level was used for both R-square of covariate and effect size because the WSEB exercise program was a newly developed exercise program and no previous research and data could be used as the reference for the parameter estimation. Based on our previous experience in conducting intervention studies with older adults (Chen et al., 2010), a 15% possible withdraw rate was added and 64 participants per group was expected to be recruited. Inclusion criteria for the participants were: (1) aged 65 years and over, (2) using wheelchairs for mobility, (3) living in the facility for at least three months, (4) cognitively intact (Short Portable Mental Status Questionnaire, SPMSQ, score $\geq$ 8) (Pfeiffer, 1975), and (5) heavy or moderate dependency in their ADL (Barthel Index, BI, score of 21–90) (Liu, 2007). Exclusion criteria were: (1) having severe or acute cardiovascular, musculoskeletal, or pulmonary illnesses, or (2) suffering from a spinal cord injury with no rehabilitation potential.

Since the participants were in wheelchairs and heavily or moderately dependent on others for their ADL, more attention or assistance might be needed in the group exercise process. To consider the manageable capacity of exercise instructors, the appropriate group size was set as approximately 15 people per cluster. Therefore, five nursing homes (clusters) for each group were invited to participate in the study. The principal investigator (PI) approached the directors of nursing homes by phone, met with them individually, and explained the purpose and design of the study. All of them agreed to participate voluntarily and be randomized by the PI through a black box drawing to either the experimental or control group. In

the black box drawing process, 10 balls with numbers from 1 to 10 represented the 10 nursing homes and were put into a black box. The sequence of assigning the numbers to the experimental or control group was pre-determined randomly by the PI. The first ball drawn represented the experimental group and the second ball represented the control group, alternating until all balls were drawn. Afterwards, the participants of a nursing home were recruited to participate in the group drawn.

2.3. Ethical considerations

After approval by the Institutional Review Board of the university hospital (IRB#: FYH-IRB-098-12-02) and the nursing home administrators, potential participants were contacted by the staff of nursing homes initially. Those older adults who were willing to participate in the study were referred to the research team by the staff of the nursing homes.

2.4. Intervention

The WSEB exercise program developed by Chen et al. (2013) was applied as the intervention for the experimental group participants in addition to their regular daily activities; the control group continued their regular daily activities, and they were invited to participate in the WSEB exercises after the completion of the study. The program includes two levels: the basic and the advanced. The basic WSEB program has three phases with four exercises in each phase: (1) warm-up, (2) aerobic motion, and (3) harmonic stretching. Two relatively challenging exercises were added in each phase of the basic WSEB program to form the advanced WSEB program (Chen et al., 2013) (Fig. 1). As

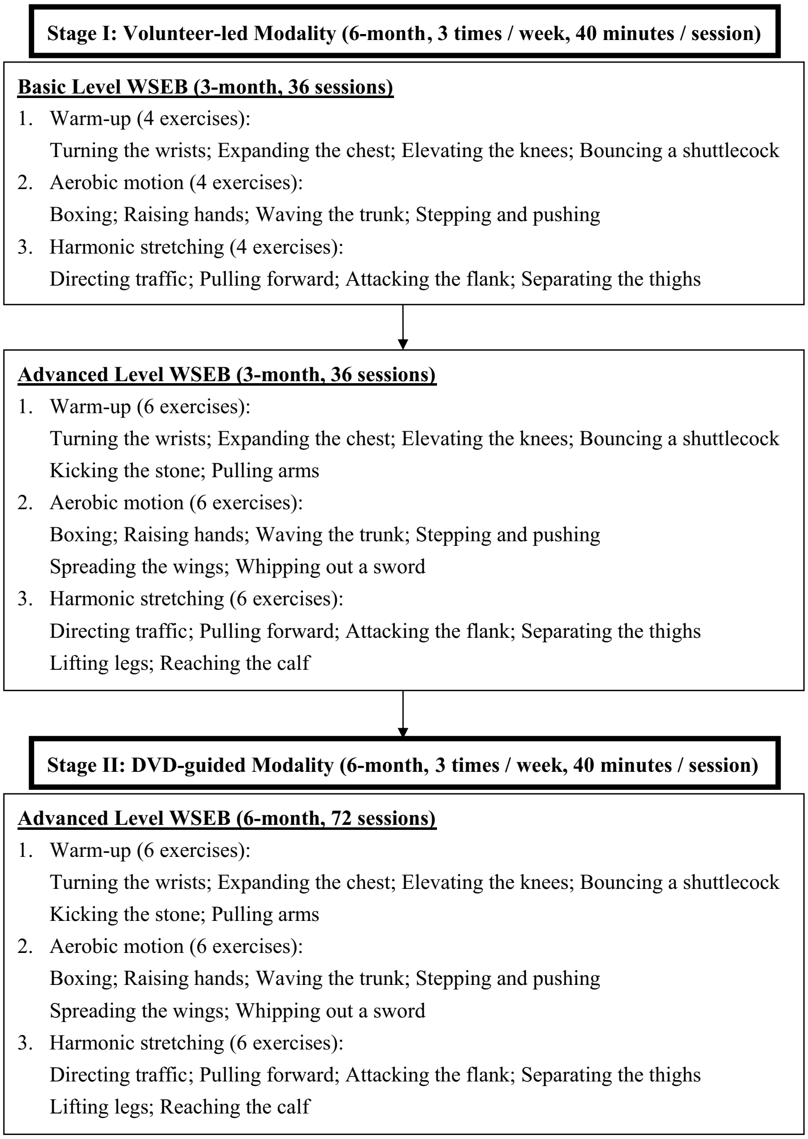

Fig. 1. Wheelchair-bound Senior Elastic Band (WSEB) Exercise Program Protocol.

suggested by Chen et al. (2013), the basic level needs to be taught and practiced for three months, and then the program proceeds to the advanced level. The WSEB program was conducted three times per week, 40 min per session, with a total of 144 sessions.

As for the modalities of carrying out the intervention, in stage I (volunteer-led modality) the basic WSEB program was taught and practiced for the first three months (36 sessions) followed by the advanced WSEB program for another three months (36 sessions) (Fig. 1). The instructors who led the program were volunteers recruited from the facilities. Most of these volunteers were middle-age women. No special requirements were needed to be an instructor in the WSEB program as long as they had patience with older adults and were willing to be trained and certified by the PI in a nine-hour training program. The nine-hour training program was separated into three sessions (3 h per session), which included: (1) introduction to the research project (15 min), (2) introduction to the basic concept and beneficial effects of elastic band exercises (35 min), (3) teach and practice the 6 exercises in the warm-up phase (100 min), (4) teach and practice the 6 exercises in the aerobic motion phase (80 min), (5) teach and practice the 6 exercises in the harmonic stretching phase (130 min), (6) practice the techniques of leading the group exercises to the elderly population (120 min), and (7) be tested and certified as WSEB instructors (60 min). A WSEB program manual that includes the above information was provided to the volunteers who received the training. To ensure intervention consistency across the intervention clusters, a pre-recorded CD made by the PI was used in conjunction with an instructor demonstrating each exercise. The other two instructors were responsible for the safety of the participants and took the attendance. However, a total of five volunteers from each nursing home were required to be trained for possible substitutes among the volunteers when they were not be able to lead some of the sessions.

In stage II (DVD-guided modality), the participants follow the DVD to practice the advanced WSEB program for another six months (72 sessions) (Fig. 1). The instructor was filmed while she led the group practice in the first six months, and then that DVD which contained the instructor with whom the participants were familiar was used to continue the group practice in stage II. Only one of the three instructors was required to be present in order to facilitate the group process and to safeguard the participants' well-being while performing the exercises. In this stage, group leaders from the participants were identified and rotated to lead the group practices. All of the interventions were carried out in the lobbies of the nursing homes. The volunteers or the staff of the nursing homes would assist the elderly residents in wheelchairs to the lobby for the exercise sessions and escort them back to their rooms once the exercise sessions were completed. During the 12-month exercises, any signs or symptoms of discomfort that occurred while exercises were required to be recorded by the instructors.

2.5. Data collection

The study was conducted from March 2012 to April 2013. Data were collected by three research assistants who were not blinded as to which group the participants were allocated but were trained by the PI for consistency in delivering the questions in the questionnaire and in performing the procedures of physical measurements to ensure inter-rater reliability. The interviews were individually conducted face-to-face and took place in a small lounge or private room in the nursing homes. The primary outcome variable was functional fitness and the secondary outcome variables were ADL and sleep quality.

2.5.1. Functional fitness

Functional fitness included lung capacity, body flexibility, range of joint motion, and muscle strength and endurance. Lung capacity was measured by the TruZone^{TM} Peak Flow Meter (Trudell Medical International, Ontario, Canada: range 60–800 l). Body flexibility included upper body flexibility (measured by the back scratch test) and lower body flexibility (measured by the chair sit-and-reach test) (Rikli and Jones, 2001). Range of joint motion included flexion and abduction of the shoulder on the dominant side and was measured by the goniometer. Finally, muscle strength and endurance included hand-grip strength, upper limb muscle endurance, and lower limb muscle endurance. Hand-grip strength was measured using a digital handgrip dynamometer (Model TKK-5401 GRIP-D: Takei Scientific Instruments Co., LTD, Japan: range 5–100 kg); upper limb muscle endurance was measured by the arm curl test (Rikli and Jones, 2001); and lower limb muscle endurance was measured by the chair-stand test (Rikli and Jones, 2001). The details of these physical measurement procedures were reported elsewhere (Chen et al., 2015b). A higher score represents a better functional fitness performance. These measurement devices were selected because they were widely used in research studies with good psychometric properties (Yang et al., 2015). Two trial tests were measured in a row for each measurement device, and the intra-class correlations were all above .98. Further, these physical measurement devices were maintained by the project manager and calibrated for their functions before each data collection to minimize the systematic errors.

2.5.2. ADL

The BI score was used to measure the participants' ADL performance. It comprises seven items (feeding, grooming, toileting, bathing, dressing, bowel control, and bladder control) to assess self-care ability, and three items (transfers, mobility, and climbing stairs) to detect activity capacity (Mahoney and Barthel, 1965). The score ranges from 0 to 100: 0 to 20 = totally dependent; 21 to 60 = heavily dependent; 61 to 90 = moderately dependent; 91 to 99 = mildly dependent; 100 = total independence in ADL functioning (Liu, 2007). A Cronbach's alpha of .90 was attained from this sample.

2.5.3. Sleep quality

The Pittsburgh Sleep Quality Index (PSQI) developed by Buysse et al. (1989) was used to measure the sleep quality of the participants. The PSQI is an 18-item, self-reported questionnaire that includes seven components: subjective sleep quality, sleep latency, sleep duration, habitual sleep

efficiency, sleep disturbances, the use of sleep medications, and daytime dysfunction. The seven component scores are summed to yield a global score; the higher the score, the worse the sleep quality. A PSQI global score > 5 indicates clinically significant sleep disturbances (90% sensitivity and 87% specificity) (Buysse et al., 1989). The Chinese PSQI, which has been shown to generate data with good reliability and validity (Chen et al., 2010; Lu et al., 2013), was used in this study. A Cronbach's alpha of .68 was attained from this sample.

2.6. Data analysis

SPSS Version 19.0 was used to analyze the data. The Pearson χ^2 test or the independent *t*-test was used to test group differences in the demographic profiles and the pre-test data. Since all outcome variables were interval or ratio scales, the parametric tests were used. The mixed-design, two-way ANOVA was used to detect the interaction effects between time and group. For the group differences at six months and at 12 months in the study, ANCOVA was computed on those variables with significant interaction effects using the pre-test data as the covariate to offset the possible group differences at the beginning of the study. One-way ANOVA repeated measures were executed to analyze the differences among three time points in each group, and the Bonferroni-adjusted test was used for the post hoc analysis. The level of significance was set as $p < .05$.

3. Results

3.1. Demographic profiles and baseline differences of the participants

Using convenience sampling, a total of 127 participants were recruited from 10 nursing homes (experimental group: five nursing homes, $n = 64$; control group: five nursing homes, $n = 63$) and 107 of them completed the 12-month study (experimental group $n = 56$, control group $n = 51$) (retention rate: 84.3%). Thirteen participants withdrew at stage I (volunteer-led) (experimental group $n = 5$; control group $n = 8$) and seven more participants dropped out at stage II (DVD-guided) (experimental group $n = 3$; control group $n = 4$). Reasons for withdrawal were deceased ($n = 12$) or discharged from nursing homes ($n = 8$) (Fig. 2). There were no significant differences in the

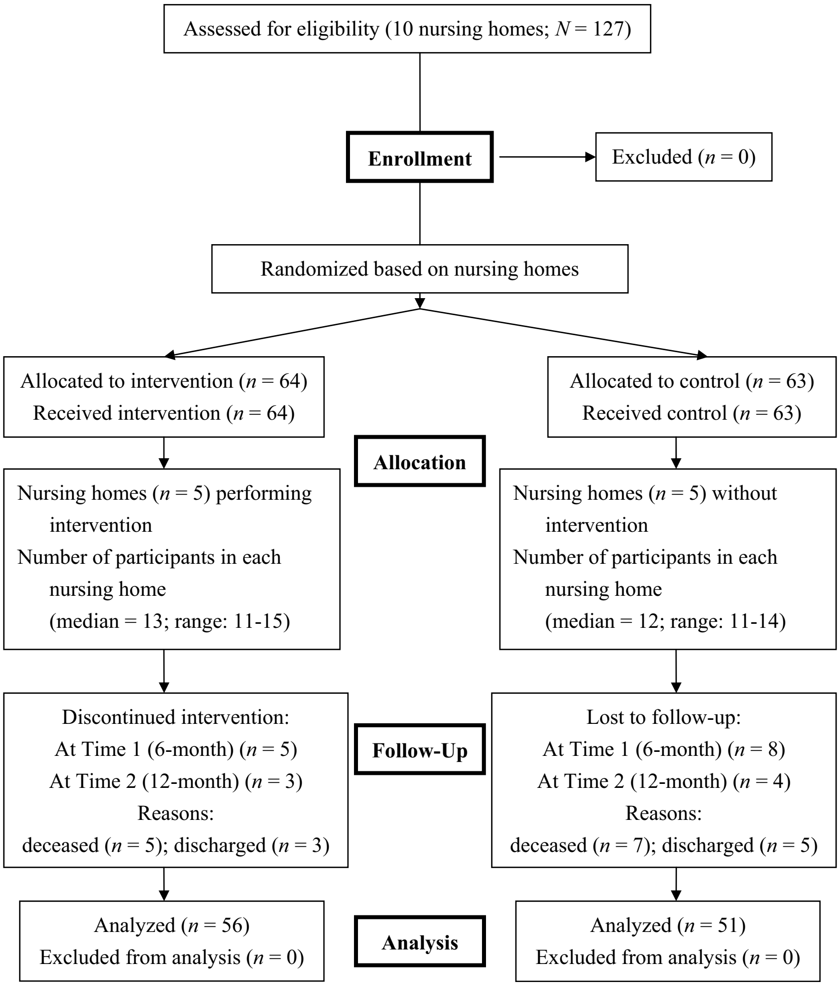

Fig. 2. Flow of the participants.

demographic profiles of the participants who remained in the study and those who dropped out (all $p > .05$).

The mean age of the 127 participants was 79.46 ± 7.06 years (range: 65–99 years old) with the majority of them being middle-old older adults (75–84 years old, 53.2%). The majority of participants was male (50.4%), widowed (59.1%), and had a six-year elementary school education (39.4%) or illiterate (33.1%). The cognitive function of the participants was intact (SPMSQ score = 9.05 ± 1.19). Almost all of the participants (98.1%) had chronic illnesses with an average number of 2.10 ± 1.01. The top three chronic illnesses reported were: (1) hypertension ($n = 71$, 66.4%), (2) stroke ($n = 46$, 43%), and (3) diabetes ($n = 33$, 30.8%) (Table 1). The size of each cluster ranged from 11 to 15 with a median of 13, and the demographic profiles of the participants across study sites and in the two groups showed no significant differences (all $p > .05$) (Table 1).

For baseline comparisons, no significant differences were found between the two groups, except that the experimental group had better upper body flexibility ($t = 2.78$, $p = .006$) than the control group (Table 1).

3.2. Intervention adherence

All of the five nursing homes in the experimental group completed the 144 intervention sessions (three times per week and 40 min per session). There were 72 sessions in each of the volunteer-led and DVD-guided stages. Nineteen participants in the volunteer-led stage and 25 participants in the DVD-guided stage completed all of the 72 sessions; the average numbers of sessions per participants were 68 ($SD = 6.12$, range: 38–72) for the volunteer-led stage and 67 ($SD = 8.89$, range: 31–72) for the DVD-guided stage. The average numbers of participants per training session were 55 ($SD = 1.86$, range: 51–59) for the volunteer-led stage and 52 ($SD = 1.53$, range: 47–55) for the DVD-guided stage. Based on the calculations of absence reason frequency divided by the sum of all absence reasons frequencies, the top three frequently reported absence reasons were: (1) hospitalization due to pneumonia, urinary tract infection, etc. (163/638 = 25.6%), (2) physical discomfort such as diarrhea, fever, flu, etc. (103/638 = 16.1%), and (3) quarantine isolation due to scarps, herpes, etc. (84/638 = 13.2%). These absence reasons were similar in both of the volunteer-led and DVD-guided stages, and no significant differences were found among the five nursing homes. During the 12-month exercises, no signs or symptoms of discomfort occurred while exercising.

3.3. Differences between two groups at different time points

Since a face-to-face individual structured-interview was applied to collect the data, participants responded to the entire interview questions and no missing data occurred. Significant interaction effects were found between three time points and two different groups in

Table 1
Demographic profiles and baseline differences of the participants ($N = 127$).

Variables	Total ($N = 127$)		Experimental ($n = 64$)		Control ($n = 63$)		Statistics (p)
	M (n)	SD (%)	M (n)	SD (%)	M (n)	SD (%)	
Age	79.46	7.06	78.83	7.69	80.10	6.35	$t = -1.01$ (.314)
Gender							$\chi^2 = 0.01$ (.929)
Male	(64)	(50.4)	(32)	(50.0)	(32)	(50.8)	
Female	(63)	(49.6)	(32)	(50.0)	(31)	(49.2)	
Marital status							$\chi^2 = 0.40$ (.820)
Single	(14)	(11.0)	(6)	(9.4)	(8)	(12.7)	
Married	(38)	(29.9)	(20)	(31.3)	(18)	(28.6)	
Widowed	(75)	(59.1)	(38)	(59.4)	(37)	(58.7)	
Education							$\chi^2 = 3.41$ (.182)
Illiterate	(42)	(33.1)	(26)	(40.6)	(16)	(25.4)	
Elementary	(50)	(39.4)	(23)	(35.9)	(27)	(42.9)	
High school	(35)	(27.6)	(15)	(23.4)	(20)	(31.8)	
Number of chronic illness	2.10	1.01	2.14	0.89	2.06	1.12	$t = 0.43$ (.668)
Cognitive function[a,d]	9.05	1.19	9.06	1.15	9.03	1.23	$t = 0.15$ (.885)
Lung capacity (l)[d]	153.35	86.66	149.69	85.40	157.06	88.45	$t = -0.48$ (.633)
Upper body flexibility (cm)[d]	−27.41	15.78	−23.65	15.72	−31.24	15.02	$t = 2.78$ (.006)
Lower body flexibility (cm)[d]	−15.74	13.24	−17.41	12.84	−14.05	13.52	$t = -1.44$ (.153)
Shoulder flexion (°)[d]	140.64	21.34	142.19	20.88	139.06	21.86	$t = 0.82$ (.412)
Shoulder abduction (°)[d]	140.43	23.49	142.36	22.85	138.48	24.15	$t = 0.93$ (.354)
Hand grip strength (kg)[d]	12.76	6.04	13.44	6.16	12.06	5.89	$t = 1.29$ (.200)
Upper limb muscle endurance (times/30 s)[d]	6.34	5.05	7.16	5.03	5.51	4.97	$t = 1.86$ (.065)
Lower limb muscle endurance (times/30 s)[d]	1.25	2.85	1.41	2.95	1.10	2.76	$t = 0.61$ (.540)
ADL[b,d]	55.16	23.90	58.28	23.88	51.98	23.68	$t = 1.49$ (.138)
Sleep quality[c,e]	7.57	4.37	8.31	3.92	6.83	4.69	$t = 1.94$ (.055)

Note: M = mean; SD = standard deviation; n = sample size; % = percentage.
[a] Cognitive function was measured by the Short Portable Mental Status Questionnaire (SPMSQ).
[b] ADL = Activities of Daily Living (measured by the Barthel Index).
[c] Sleep quality was measured by the Pittsburgh Sleep Quality Index (PSQI).
[d] Higher score indicates better outcome.
[e] Higher score indicates worse outcome.

all of the variables (all $p < .05$). To reflect the issue of intention-to-treat, all of the data from 127 participants were included in the data analysis. The experimental group had better outcomes in all of the variables than the control group at six months in the study (all $p < .05$) (Table 2), and these significant differences were maintained throughout the 12-month study (Table 3). Since possible cluster-induced effects on the variance of outcome data may exist, the 95% confidence intervals were reported in group comparisons (Tables 2 and 3).

3.4. Changes among three time points in each group

For those who completed the 12-month study ($N = 107$), the trend of change was further analyzed. Results indicated that all of the functional fitness indicators of the experimental group participants ($n = 56$) improved significantly (all $p < .05$). However, no significant differences were found in the variables of ADL ($F = 0.41$, $p = .667$) and sleep quality ($F = 2.45$, $p = .091$) in the experimental group (See online Appendix A). On the other hand, the conditions in the control group participants ($n = 51$) deteriorated significantly: lung capacity ($F = 5.85$, $p = .010$), lower body flexibility ($F = 31.48$, $p < .001$), shoulder flexion ($F = 5.54$, $p = .012$), hand grip strength ($F = 3.63$, $p = .042$), ADL ($F = 9.59$, $p < .001$), and sleep quality ($F = 6.97$, $p = .001$) (See online Appendix A).

4. Discussion

This study found that participants who received WSEB exercise training had better functional fitness, ADL, and

Table 2
Group differences at six-month of the study ($N = 114$).

Variables	Unadjusted *M*	Adjusted *M*[c]	95% CI	*SS*	*df*	*MS*	*F* (*p*)
Lung capacity (l)[d]							
E	178.81	182.27	171.31 ~ 193.24	42,356.83	1	42,356.83	23.48 (<.001)
C	147.36	143.65	132.30 ~ 155.01				
E-C	31.45	38.62	22.83 ~ 54.42				
Upper body flexibility (cm)[d]							
E	−21.08	−23.98	−25.36 ~ −22.60	436.57	1	436.57	15.67 (<.001)
C	−31.13	−28.01	−29.45 ~ −26.58				
E-C	10.05	4.03	2.02 ~ 6.05				
Lower body flexibility (cm)[d]							
E	−13.32	−11.60	−13.22 ~ −9.97	1349.46	1	1349.46	34.37 (<.001)
C	−16.71	−18.56	−20.25 ~ −16.88				
E-C	3.39	6.97	4.61 ~ 9.32				
Shoulder flexion (°)[d]							
E	146.46	145.98	143.52 ~ 148.45	2505.08	1	2505.08	27.45 (<.001)
C	136.09	136.60	134.05 ~ 139.15				
E-C	10.37	9.39	5.84 ~ 12.94				
Shoulder abduction (°)[d]							
E	148.39	147.95	145.14 ~ 150.76	3118.62	1	3118.62	26.27 (<.001)
C	137.00	137.47	134.56 ~ 140.29				
E-C	11.39	10.47	6.42 ~ 14.52				
Hand grip strength (kg)[d]							
E	14.64	13.91	13.23 ~ 14.58	67.26	1	67.26	9.92 (.002)
C	11.57	12.36	11.66 ~ 13.06				
E-C	3.07	1.55	0.58 ~ 2.53				
Upper limb muscle endurance (times/30 s)[d]							
E	9.73	8.94	8.22 ~ 9.67	293.17	1	293.17	37.76 (<.001)
C	4.82	5.66	4.91 ~ 6.41				
E-C	4.55	3.28	2.22 ~ 4.34				
Lower limb muscle endurance (times/30 s)[d]							
E	2.58	2.27	1.76 ~ 2.77	31.72	1	31.72	8.27 (.005)
C	0.87	1.20	0.68 ~ 1.73				
E-C	1.71	1.06	0.33 ~ 1.80				
ADL[a,d]							
E	56.95	54.19	51.12 ~ 57.26	665.17	1	665.17	4.74 (.032)
C	46.36	49.32	46.14 ~ 52.50				
E-C	10.59	4.87	0.44 ~ 9.31				
Sleep quality[b,e]							
E	7.68	7.04	6.24 ~ 7.84	45.27	1	45.27	4.83 (.030)
C	7.64	8.32	7.50 ~ 9.15				
E-C	0.04	−1.29	−2.44 ~ −0.13				

Note: E = Experimental group; C = Control group; E-C = Between-group difference; *M* = mean; 95% CI = 95% confidence interval.
SS = sum of square; *df* = degree of freedom; *MS* = mean of square.
[a] ADL = Activities of Daily Living (measured by the Barthel Index).
[b] Sleep quality was measured by the Pittsburgh Sleep Quality Index (PSQI).
[c] The pre-test data were used as the covariate to adjust the possible group differences at the beginning of the study.
[d] Higher score indicates better outcome.
[e] Higher score indicates worse outcome.

Table 3
Group Differences at 12-Month of the Study ($N = 107$).

Variables	Unadjusted M	Adjusted M[c]	95% CI	SS	df	MS	F (p)
Lung capacity (l)[d]							
E	182.86	187.43	173.31 ~ 201.54	81,818.49	1	81,818.49	28.91 (<.001)
C	136.96	131.94	117.16 ~ 146.73				
E-C	45.90	55.48	35.02 ~ 75.94				
Upper body flexibility (cm)[d]							
E	−18.46	−21.06	−22.62 ~ −19.50	1910.03	1	1910.03	56.68 (<.001)
C	−32.61	−29.76	−31.39 ~ −28.12				
E-C	14.15	8.69	6.40 ~ 10.98				
Lower body flexibility (cm)[d]							
E	−10.93	−9.15	−11.02 ~ −7.28	3490.94	1	3490.94	71.10 (<.001)
C	−18.78	−20.74	−22.70 ~ −18.78				
E-C	7.85	11.59	8.87 ~ 14.32				
Shoulder flexion (°)[d]							
E	149.20	148.49	145.50 ~ 151.48	5179.65	1	5179.65	40.67 (<.001)
C	133.76	134.54	131.41 ~ 137.68				
E-C	15.44	13.95	9.61 ~ 18.29				
Shoulder abduction (°)[d]							
E	151.61	151.14	147.83 ~ 154.44	7074.24	1	7074.24	45.52 (<.001)
C	134.33	134.85	131.39 ~ 138.31				
E-C	17.28	16.29	11.50 ~ 21.07				
Hand grip strength (kg)[d]							
E	15.23	14.65	13.78 ~ 15.53	258.01	1	258.01	23.76 (<.001)
C	10.89	11.52	10.60 ~ 12.44				
E-C	4.34	3.13	1.86 ~ 4.41				
Upper limb muscle endurance (times/30 s)[d]							
E	10.75	10.00	9.08 ~ 10.93	519.45	1	519.45	43.68 (<.001)
C	4.67	5.49	4.52 ~ 6.46				
E-C	6.08	4.52	3.16 ~ 5.88				
Lower limb muscle endurance (times/30 s)[d]							
E	2.54	2.33	1.84 ~ 2.83	32.66	1	32.66	9.42 (.003)
C	1.00	1.22	0.71 ~ 1.74				
E-C	1.54	1.11	0.39 ~ 1.83				
ADL[a,d]							
E	57.86	55.61	52.11 ~ 59.10	2241.92	1	2241.92	12.93 (<.001)
C	43.92	46.39	42.73 ~ 50.06				
E-C	13.94	9.21	4.13 ~ 14.29				
Sleep quality[b,e]							
E	7.39	6.74	5.95 ~ 7.54	97.25	1	97.25	11.08 (.001)
C	7.98	8.70	7.86 ~ 9.53				
E-C	−0.59	−1.96	−3.13 ~ −0.79				

Note: E = Experimental group; C = Control group; E-C = Between-group difference; M = mean; 95% CI = 95% confidence interval.
SS = sum of square; df = degree of freedom; MS = mean of square.
[a] ADL = Activities of Daily Living (measured by the Barthel Index).
[b] Sleep quality was measured by the Pittsburgh Sleep Quality Index (PSQI).
[c] The pre-test data were used as the covariate to adjust the possible group differences at the beginning of the study.
[d] Higher score indicates better outcome.
[e] Higher score indicates worse outcome.

sleep quality than those who did not, and confirmed our hypothesis that the positive effects of WSEB program occurred after six months of volunteer-led exercises and were maintained or even enhanced through the following six months of DVD-guided practices. However, these interpretations need to bear in mind that although a low attrition rate occurred in this study, lacking data of those lost to follow-up might have an impact on the results. We specifically focused on finding the feasible modalities to carry out the WSEB exercise program longitudinally in this study. Only one person was needed in leading the exercises while using the DVD modality and fewer withdrawals of the participants occurred in the DVD-guided stage than the instructor-led stage. Therefore, the sequence of instructor-led followed by DVD-guided group practice of WSEB exercises was feasible and acceptable by this group of nursing home older adults in wheelchairs for routine and long-term applications.

The functional fitness improvements echoed the majority of the research conducted in the area of elastic band exercises for older adults (Chang et al., 2012; Chen et al., 2009; Latham and Liu, 2010; Sugimoto and Blanpied, 2006; Swank et al., 2003). Functional fitness is essential in completing the daily functional tasks and the exercises in the WSEB program are specifically designed to train these functions. For example, the exercises of pulling the arms, raising hands, and an action similar to whipping out a sword were aimed at improving upper body flexibility in order to perform daily activities, such as combing hair, changing clothes, and scrubbing the back while showering or bathing (Chen et al., 2013). Even with this particular group of participants who were rather old in age, less

educated, and had multiple morbidities, the potential to promote their functional fitness was present.

Participants in the two groups were heavily dependent on others for their ADL at baseline, and significant differences between the two groups occurred at both six and 12 months of the study. This result might be attributed to the significant ADL deterioration in the control group since the experimental group participants did not show significant changes in their ADL after WSEB exercises. The possible explanation was that the ADL deterioration that occurred in the control group participants might possibly have happened to the experimental group participants and compromised the significant ADL improvement in the experimental group. The experimental group did not show significant change in their ADL as did the control group was an indication that the WSEB exercises prevented a decline in the ADL of the experimental group.

Sleep quality was less discussed in the effects of elastic band exercises in older adults. Results showed that both experimental and control group participants had PSQI total scores > 5 at baseline, indicating that participants had clinically significant sleep disturbances (Buysse et al., 1989). According to Foley et al. (2004), sleep disturbances might be related to inactive life styles, such as repetitive daily routines and lack of physical exercise. Especially for this particular group of older adults in wheelchairs, poor sleep practices, such as excessive time spent in bed and sleeping during the day, exacerbated the problem of sleep disturbances. However, the experimental group participants practiced stretching and relaxing in the WSEB program and their bodies were moved to burn energy, thereby enhancing their sleep quality.

4.1. Study limitations

First, although the method of random assignment by study sites into experimental or control group was applied, the process was done by the PI, which might threaten the concealment of allocation. Second, the participants from each study site were recruited conveniently after the study site randomization was completed. The participants were aware of the study group they were assigned to, which might influence their willingness to participate, lead to a high risk of selection bias, result in the baseline differences between the two groups, undermine the validity of the results, and limit the generalizability of the findings. In future studies, it is suggested that the participants will be recruited and underwent baseline assessment before the study sites will be assigned to the study groups. Third, the three research assistants who collected the data were not blinded as to which group the participants were allocated, which might create a risk of bias in data collection. Possible blinding should be incorporated in future study designs. Fourth, this study only measured the intervention outcomes quantitatively, and the participants' experiences toward the WSEB exercises were not explored. Adding the qualitative inquiry might enrich the data. Fifth, the experiences of the staff in the experimental clusters toward the implementation of the WSEB program were not examined. The work of the nursing staff might be affected by the program implementation, and their perspectives toward the program should be explored in the future studies. Finally, the interventions were implemented at the lobbies of the nursing homes, and some of the lobbies were located right next to the entrance. Participants may have been distracted during the interventions. A wide and ventilated setting is recommended for the WSEB group exercises.

5. Conclusions

This study concluded that the WSEB exercise program improved functional fitness, ADL, and sleep quality of nursing home older adults in wheelchairs with multiple morbidities, and the volunteer-led modality followed by the DVD-guided modality was a feasible way of carrying out the WSEB program in an institutional setting, routinely and longitudinally. When applying the WSEB exercise program in the clinical settings, we suggest that in the volunteer-led stage, the volunteers must be pre-trained, and the nine-hour training protocol indicated in this study should be followed. It must be emphasized that the volunteers should not only learn how to practice WSEB exercises themselves, but also be equipped with skills for leading the group exercises for older adults. In the DVD-guided stage, at least two large TV screens would be more appropriate to broadcast the DVD. In this stage, at least one instructor was still needed to facilitate the group exercise and to safeguard the participants' well-being while performing the exercises. Group leaders from the participants should be identified and rotated to lead the group practices. The volunteer-led followed by the DVD-guided modality holds promise in incorporating the WSEB exercises as a part of daily activities for nursing home older adults in wheelchairs.

Acknowledgements

Sincere appreciation is directed by our group to the National Science Council, Taiwan for funding this study (NSC 99-2628-B-037-066-MY3), to Professor Frank Belcastro for his superlative manuscript editing, to the directors and staff of 10 nursing homes for their support and assistance, and to the 127 wonderful older adults for their generous participation.

Conflict of interest: No conflict of interest has been declared by the authors.

Funding: This study was funded by the National Science Council, Taiwan (NSC 99-2628-B-037-066-MY3). The funding source supported the study financially and had no involvement in the study design, data collection, analysis and interpretation of data, writing of the report, and decision to submit the paper for publication.

Ethical approval: Approval was received from the Institutional Review Board of Fooyin University Hospital (FYH-IRB-098-12-02).

Appendix A. Supplementary data

Supplementary data associated with this article can be found, in the online version, at http://dx.doi.org/10.1016/j.ijnurstu.2015.11.004.

References

American Health Care Association, 2014. Trends in Nursing Facility Characteristics. American Health Care Association, Washington, DC.

Benjamin, K., Edwards, N., Guitard, P., Murray, M.A., Caswell, W., Perrier, M.J., 2011. Factors that influence physical activity in long-term care: perspectives of residents, staff, and significant others. Can. J. Aging 30, 1–12.

Benjamin, K., Edwards, N., Ploeg, J., Legault, F., 2014. Barriers to physical activity and restorative care for residents in long-term care: a review of the literature. J. Aging Phys. Act. 22, 154–165.

Burton, L.A., Sumukadas, D., 2010. Optimal management of sarcopenia. J. Clin. Interv. Aging 5, 217–228.

Buysse, D.J., Reynolds III, C.F., Monk, T.H., Berman, S.R., Kupfer, D.J., 1989. The Pittsburgh Sleep Quality Index: a new instrument for psychiatric practice and research. Psychiatry Res. 28, 193–213.

Chang, T.F., Liou, T.H., Chen, C.H., Huang, Y.C., Chang, K.H., 2012. Effects of elastic-band exercise on lower-extremity function among female patients with osteoarthritis of the knee. Disabil. Rehabil. 34, 1727–1735.

Chen, T.A., Wu, Y.T., Lee, M.B., Liang, K.C., Lin, K.N., Tsai, M.W., 2009. Effects of exercise on depression symptoms, physical function, and quality of life in community-dwelling elderly. Formos. J. Phys. Therapy 34, 209–218.

Chen, K.M., Chen, M.H., Lin, M.H., Fan, J.T., Lin, H.S., Li, C.H., 2010. Effects of yoga on sleep quality and depression in elders in assisted living facilities. J. Nurs. Res. 18, 53–61.

Chen, K.M., Tseng, W.S., Chang, Y.H., Huang, H.T., Li, C.H., 2013. Feasibility appraisal of an elastic band exercise program for older adults in wheelchairs. Geriatr. Nur. (Lond.) 34, 373–376.

Chen, K.M., Huang, H.T., Cheng, Y.Y., Li, C.H., Chang, Y.H., 2015a. Sleep quality and depression of nursing home older adults in wheelchairs after exercises. Nurs. Outlook 63, 357–365.

Chen, K.M., Li, C.H., Chang, Y.H., Huang, H.T., Cheng, Y.Y., 2015b. An elastic band exercise program for older adults using wheelchairs in Taiwan nursing homes: a cluster randomized trial. Int. J. Nurs. Stud. 52, 30–38.

Clarke, P., Chan, P., Santaguida, P.L., Colantonio, A., 2009. The use of mobility devices among institutionalized older adults. J. Aging Health 21, 611–626.

Foley, D., Ancoli-Israel, S., Britz, P., Walsh, J., 2004. Sleep disturbances and chronic disease in older adults: results of the 2003 National Sleep Foundation sleep in America survey. J. Psychosom. Res. 56, 497–502.

Froehlich-Grobe, K., Lee, J., Aaronson, L., Nary, D.E., Washburn, R.A., Little, T.D., 2014. Exercise for everyone: a randomized controlled trial of project workout on wheels in promoting exercise among wheelchair users. Arch. Phys. Med. Rehabil. 95, 20–28.

Gavin-Dreschnack, D., Volicer, L., Morris, C., 2010. Prevention of overuse of wheelchairs in nursing homes. Ann. Long-Term Care: Clin. Care Aging 18, 34–38.

Gothe, N.P., Wójcicki, T.R., Olson, E.A., Fanning, J., Awick, E., Chung, H.D., Zuniga, K.E., Mackenzie, M.J., Motl, R.W., McAuley, E., 2015. Physical activity levels and patterns in older adults: the influence of a DVD-based exercise program. J. Behav. Med. 38, 91–97.

Ikezoe, T., Asakawa, Y., Shima, H., Kishibuchi, K., Ichihashi, N., 2013. Daytime physical activity patterns and physical fitness in institutionalized elderly women: an exploratory study. Arch. Gerontol. Geriatr. 57, 221–225.

Khan, M.A., Shah, S., Grudzien, A., Onyejekwe, N., Banskota, P., Karim, S., Jin, J., Kim, Y., Gerber, B.S., 2011. A diabetes education multimedia program in the waiting room setting. Diabetes Therapy: Res. Treat. Educ. Diabetes Relat. Disord. 2, 178–188.

Kingston, G., Gray, M.A., Williams, G., 2010. A critical review of the evidence on the use of videotapes or DVD to promote patient compliance with home programs. Disabil. Rehabil. Assist. Technol. 5, 153–163.

Latham, N., Liu, C.J., 2010. Strength training in older adults: the benefits for osteoarthritis. Clin. Geriatr. Med. 26, 445–459.

Liu, W.M., 2007. Assessment for older adults. In: Kao, S.F. (Ed.), Gerontological Nursing. 2nd ed. Yeong Dah, Taipei, Taiwan (pp. 8-1–8-21).

Lu, M.J., Lin, S.T., Chen, K.M., Tsang, H.Y., Su, S.F., 2013. Acupressure improves sleep quality of psychogeriatric inpatients. Nurs. Res. 62, 130–137.

Mahoney, F.I., Barthel, D.W., 1965. Functional evaluation: the Barthel Index. Md. State Med. J. 14, 61–65.

Martin, J.T., Wolf, A., Moore, J.L., Rolenz, E., DiNinno, A., Reneker, J.C., 2013. The effectiveness of physical therapist-administered group-based exercise on fall prevention: a systematic review of randomized controlled trials. J. Geriatr. Phys. Therapy 36, 182–193.

McAuley, E., Jerome, G.J., Marquez, D.X., Elavsky, S., Blissmer, B., 2003. Exercise self-efficacy in older adults: social, affective, and behavioral influences. Ann. Behav. Med. 25, 1–7, http://dx.doi.org/10.1207/S15324796ABM2501_01.

Newton, R.U., Hakkinen, K., Hakkinen, A., McCormick, M., Volek, J., Kraemer, W.J., 2002. Mixed methods resistance training increases power and strength of young and older men. Med. Sci. Sports Exerc. 34, 1367–1375.

Pfeiffer, E., 1975. A short portable mental status questionnaire for the assessment of organic brain deficit in elderly patients. J. Am. Geriatr. Soc. 23, 433–441.

Phillips, E.M., Schneider, J.C., Mercer, G.R., 2004. Motivating elders to initiate and maintain exercise. Arch. Phys. Med. Rehabil. 85, S52–S57.

Rikli, R.E., Jones, C.J., 2001. Senior Fitness Test Manual. Human Kinetics, Champaign, IL.

Shin, Y.I., Lee, H.S., Jang, Y.S., 2007. The effect of group exercise program on the activities of daily living and muscle strength in the spinal cord injury person. J. Rehabil. Res. 11, 77–95.

Shore, S.L., 2008. Use of an economical wheelchair in India and Peru: impact on health and function. Med. Sci. Monit. 14 (12), PH71–PH79.

So, W.Y., Song, M., Park, Y.H., Cho, B.L., Lim, J.Y., Kim, S.H., Song, W., 2013. Body composition, fitness level, anabolic hormones, and inflammatory cytokines in the elderly: a randomized controlled trial. Aging Clin. Exp. Res. 25 (2), 167–174.

Sugimoto, D., Blanpied, P., 2006. Flexible foil exercise and shoulder internal and external rotation strength. J. Athlet. Train. 41, 280–285.

Swank, A.M., Funk, D.C., Durham, M.P., Roberts, S., 2003. Adding weights to stretching exercise increases passive range of motion for healthy elderly. J. Strength Cond. Res. 17, 374–378.

Topp, R., Boardley, D., Morgan, A.L., Fahlman, M., McNevin, N., 2005. Exercise and functional tasks among adults who are functionally limited. West. J. Nurs. Res. 27, 252–270.

U.S. Department of Health and Human Services, 2013. A Profile of Older Americans: 2013. U.S. Department of Health and Human Services, Washington, DC.

Van der Ploeg, E.S., Walker, H., O'Connor, D.W., 2014. The feasibility of volunteers facilitating personalized activities for nursing home residents with dementia and agitation. Geriatr. Nur. (Lond.) 35, 142–146.

Wang, H.H., Tsay, S.F., 2012. Elderly and long-term care trends and policy in Taiwan: challenges and opportunities for health care professionals. Kaohsiung J. Med. Sci. 28, 465–469.

Webber, S.C., Porter, M.M., 2010. Effects of ankle power training on movement time in mobility-impaired older women. Med. Sci. Sports Exerc. 42, 1233–1240.

Wójcicki, T.R., Fanning, J., Awick, E., Olson, E.A., Motl, R.W., McAuley, E., 2014. Maintenance effects of a DVD-delivered exercise intervention on physical function in older adults. J. Gerontol. A, http://dx.doi.org/10.1093/gerona/glu188, Advance online publication.

World Health Organization, 2014. Physical Activity and Older Adults. Retrieved from http://www.who.int/dietphysicalactivty/factsheet_olderadults/en/

Yang, H.J., Chen, K.M., Chen, M.D., Wu, H.C., Chang, W.J., Wang, Y.C., Huang, H.T., 2015. Applying the transtheoretical model to promote functional fitness of community older adults participating in elastic band exercises. J. Adv. Nurs. 71, 2338–2349.

Yasuda, T., Fukumura, K., Fukuda, T., Iida, H., Imuta, H., Sato, Y., Yamasoba, T., Nakajima, T., 2014. Effects of low-intensity, elastic band resistance exercise combined with blood flow restriction on muscle activation. Scand. J. Med. Sci. Sports 24, 55–61, http://dx.doi.org/10.1111/j.1600-0838.2012.01489.x.
