# Supplementary material for: Effects of elastic band resistance training on the physical and mental health of elderly individuals: A mixed methods systematic review
Source: PLoS One. 2024 May 13;19(5):e0303372. doi: 10.1371/journal.pone.0303372 (PMC11090353; doi:10.1371/journal.pone.0303372)
Supplement: S1 File — (ZIP) [file pone.0303372.s001.zip › Supporting Information/Included study 54.pdf]

JAN
Informing Practice and Policy Worldwide through Research and Scholarship

ORIGINAL RESEARCH: EMPIRICAL RESEARCH – QUANTITATIVE

Applying the transtheoretical model to promote functional fitness of community older adults participating in elastic band exercises

Hui-Ju Yang, Kuei-Min Chen, Ming-De Chen, Hui-Chuan Wu, Wen-Jane Chang, Yueh-Chin Wang & Hsin-Ting Huang

Accepted for publication 14 May 2015

Correspondence to K. M. Chen:
e-mail: kmc@kmu.edu.tw

Hui-Ju Yang MS RN
Lecturer
School of Nursing, Tzu Hui Institute of Technology, Pingtung, Taiwan and
Doctoral Student
College of Nursing, Kaohsiung Medical University, Kaohsiung, Taiwan

Kuei-Min Chen PhD RN
Professor
College of Nursing, Kaohsiung Medical University, Taiwan

Ming-De Chen PhD OT
Assistant Professor
Department of Occupational Therapy, Kaohsiung Medical University, Taiwan

Hui-Chuan Wu MS RN
Lecturer
Department of Midwifery, Fooyin University, Kaohsiung, Taiwan

Wen-Jane Chang MS RN
Lecturer
Department of Nursing, Fooyin University, Kaohsiung, Taiwan

Yueh-Chin Wang MS RN
Lecturer
Department of Nursing, Fooyin University, Kaohsiung, Taiwan

Hsin-Ting Huang BS RN
Research Assistant
College of Nursing, Kaohsiung Medical University, Taiwan

YANG H. J., CHEN K. M., CHEN M. D., WU H. C., CHANG W. J., WANG Y. C. & HUANG H. T. (2015) Applying the transtheoretical model to promote functional fitness of community older adults participating in elastic band exercises. *Journal of Advanced Nursing* **71**(10), 2338–2349. **doi: 10.1111/jan.12705**

Abstract

Aims. The transtheoretical model was applied to promote behavioural change and test the effects of a group senior elastic band exercise programme on the functional fitness of community older adults in the contemplation and preparation stages of behavioural change.

Background. Forming regular exercise habits is challenging for older adults. The transtheoretical model emphasizes using different strategies in various stages to facilitate behavioural changes.

Design. Quasi-experimental design with pre-test and post-tests on two groups.

Methods. Six senior activity centres were randomly assigned to either the experimental or control group. The data were collected during 2011. A total of 199 participants were recruited and 169 participants completed the study (experimental group $n = 84$, control group $n = 85$). The elastic band exercises were performed for 40 minutes, three times per week for 6 months. The functional fitness of the participants was evaluated at baseline and at the third and sixth month of the intervention. Statistical analyses included a two-way mixed design analysis of variance, one-way repeated measures analysis of variance and an analysis of covariance.

Results. All of the functional fitness indicators had significant changes at post-tests from pre-test in the experimental group. The experimental group had better performances than the control group in all of the functional fitness indicators after three months and 6 months of the senior elastic band exercises.

Conclusion. The exercise programme provided older adults with appropriate strategies for maintaining functional fitness, which improved significantly after the participants exercising regularly for 6 months.

Keywords: community, elastic band, functional fitness, nursing, older adults, physical activity, transtheoretical model

© 2015 John Wiley & Sons Ltd

Why is this research needed?

- The health conditions of older adults deteriorate with age and establishing regular physical exercise habit is a challenge for people in this particular age group.
- Interventions that correspond to the dynamic processes and principles of behavioural change benefit the strengthening of exercise behaviours.
- The economic benefits obtained by applying the contemplation and preparation stages of the transtheoretical model as an exercise behaviorial change intervention can be increased.

What are the key findings?

- After 3 months of regular elastic band exercises, the lung capacity, cardiopulmonary fitness, body flexibility, muscle power and muscle endurance of the community older adults significantly improved.
- The functional fitness performance of the experimental group participants after 6 months of the senior elastic band exercises was significantly superior to that of the control group.
- Most participants who were in the contemplation or preparation stages of the transtheoretical model switched to the action stage after 6 months of elastic band interventions.

How should the findings be used to influence policy/ practice/research/education?

- The elastic band exercise programme is inexpensive, easy to use, safe and suitable for older adults to practice at home.
- Providing appropriate exercise prescriptions for people in the contemplation and preparation stages of behaviorial change can effectively facilitate regular exercise behaviours and enhance the functional fitness of older adults.
- A long-term follow-up study of outcomes should be conducted to enhance the effects and benefits of the elastic band exercise programme as a physical exercise option for older adults.

Introduction

According to the World Health Organization (WHO 2013), the four primary risk factors of non-communicable diseases are tobacco use, harmful use of alcohol, unhealthy diets and physical inactivity. Physical inactivity, or having a sedentary lifestyle, is one of the 10 main reasons for global mortality and disability (WHO 2013). Approximately 60-85% of adults worldwide have sedentary lifestyles and 6% of all annual mortalities are related to insufficient physical exercise (WHO 2013). More than two-million deaths occur because of sedentary lifestyles (WHO 2013). Since 1993, Taiwan has become an ageing society and the proportion of citizens older than 65 years has increased; it reached 11·50% at the end of 2013. The ageing index is 80·51%, which is higher than the global average (30·77%) and that of developing countries (20·69%) (Department of Statistics, Ministry of the Interior, Taiwan 2014). Ageing affects cardiorespiratory health, muscle and joint functions, quality of life and independent living and increases the mortality rate (Nelson *et al.* 2007, Sui *et al.* 2007, Gremeaux *et al.* 2012). Compared with those who exercise regularly, inactive older adults have higher mortality and disability rates and functional dependency and require the additional use of healthcare resources (Nylen *et al.* 2010, Martins *et al.* 2011). Therefore, promoting regular physical activity habits for older adults has attracted substantial attention.

Background

Transtheoretical model (TTM)

The physical and psychological conditions of older adults deteriorate with age, such as muscle weakness, physical frailty and memory loss (Milanović *et al.* 2013); thus, forming regular physical exercise habits is challenging for people in this particular age group. The TTM, based on various theories of psychotherapy and behaviorial change (hence the name 'transtheoretical'), was developed by Prochaska and DiClemente in 1983. This model suggests that behaviorial change is a dynamic process that can be divided into various stages instead of an all-or-none phenomenon (Prochaska & DiClemente 1983). The core constructs of this model include stages of change, self-efficacy and decisional balance and several cognitive and behaviorial processes of change (Prochaska & Marcus 1994). The stages of change include the following: (1) Pre-contemplation: participants were not exercising and had no intention to exercise in the following 6 months; (2) Contemplation: participants were not exercising, but had the intention to exercise within the next 6 months; (3) Preparation: participants planned to begin exercising within 1 month or were exercising irregularly; (4) Action: participants had been regularly exercising for the past 6 months; (5) Maintenance: participants had been regularly exercising for over 6 months.

Various strategies can be employed in different stages to achieve behaviorial changes. Interventions that correspond to the dynamic processes and principles of behaviorial change are beneficial for strengthening exercise behaviours (Prochaska & Velicer 1997). Consciousness rising (actively seeking information on exercise behaviour) involves increased awareness about the causes and consequences of

© 2015 John Wiley & Sons Ltd

and solutions for a particular problem behaviour and is highly appropriate for stages ranging from pre-contemplation to contemplation. Social liberation (awareness and acceptance of social changes on encouraging active lifestyles) provides appropriate knowledge and information for reducing the internal conflict and resistance of individuals with problem behaviours, thereby raising awareness of behaviorial change, which is appropriate for stages ranging from pre-contemplation to action. Furthermore, dramatic relief (caring about consequences of inactivity or no exercise), environmental reevaluation (understanding how inactivity affects the physical and social environments) and self-reevaluation (seeing oneself as an active person) can reinforce the motivation to change and are appropriate for stages ranging from contemplation to preparation. Self-liberation (choosing and making the commitment to change and believing in the ability to change a behaviour) involves demonstrating the determination to change and is appropriate for stages ranging from preparation to maintenance. Counterconditioning (substituting alternatives for inactivity or no exercise), reinforcing management (using rewards to encourage or maintain behaviorial changes), stimulus control (avoiding or controlling stimuli and other causes that support inactivity or no exercise) and beneficial relationships (seeking social support for adopting and maintaining exercise behaviours) can support changes and reduce the risk of relapses and are appropriate for stages ranging from action to maintenance (Prochaska *et al.* 1992, Prochaska & Marcus 1994, Kim *et al.* 2004).

Kao *et al.* (2002) stated that the effectiveness of exercise behaviour change can be improved by selecting an appropriate exercise programme and applying the TTM at the contemplation and preparation stages. The group-based intervention enhances self-efficacy and the adherence to and persistence in the exercise programme (McAuley *et al.* 2003, McPhate *et al.* 2013). Interventions can include teaching, providing or leading diverse exercise methods, such as group exercise courses. In addition, informing participants of the benefits of exercises, assisting participants to overcome exercise obstacles and establishing goals for enhancing participant motivation and confidence to change behaviours are essential actions that should be incorporated into the exercise programme design (Tseng *et al.* 2003).

Exercise and functional fitness in older adults

Designing physical exercise programs suitable for older adults is essential for fulfilling their exercise needs and preventing possible harm caused by inappropriate physical activity (Warren *et al.* 2010). According to Chodzko-Zajko *et al.* (2009), the muscle strength of people older than 50 years decreases 15-20% every 10 years. Haddock and Wilkin (2010) found that the lower limb muscle power of older adults is the key to maintaining physical independence. People who have lost their lower limb muscle power encounter difficulties in performing activities necessary for daily life (such as walking, climbing stairs and using the toilet); thus, they become physically disabled (Puthoff & Nielsen 2007, Haddock & Wilkin 2010). Rikli and Jones (2001) found that lower limb muscle power is related to aerobic endurance, muscular endurance, flexibility and dynamic equilibrium and is an essential factor for functional fitness. They asserted that functional fitness refers to possessing the physiological capacity to perform regular daily activities safely and independently without undue fatigue. Functional fitness is a predictor of disability, dependency and hospitalization (Guralnik *et al.* 1995). Physical exercise programs that aim to enhance cardiac, respiratory and muscular joint functions (e.g. elasticity and balance) must include aerobic and resistance exercises (Weening-Dijksterhuis *et al.* 2011, Gremeaux *et al.* 2012).

The Senior Elastic Band (SEB) exercise programme is specifically designed to address the six common health problems (e.g. reduced cardiovascular capacity, flexibility, upper limb muscle power and lower limb muscle endurance; poor balance; and sleep disorder) encountered by community older adults (Chen *et al.* 2013). The programme has been verified by 11 experts with combined expertise in exercise for older adults, gerontological nursing, physical therapy, exercise physiology, sports medicine and sports injuries using Delphi techniques (Chen *et al.* 2013). A pilot study further demonstrated that after 4 weeks of SEB exercises, a small sample of 20 participants improved in six out of nine functional fitness indicators compared with their baseline; the indicators included improved lung capacity, cardiopulmonary fitness, upper and lower body flexibility, upper limb muscle power and lower limb muscle endurance (Chan *et al.* 2015). Although promising results were found in this pilot study, a convenience sampling method involving one group pre-test and post-test was used. No control group was involved and many confounding factors may have affected the results, implying weaker inferences. Further verifications of the effects of the SEB exercises are required.

The study

Aims

This study applied the TTM to test the effects of the group SEB exercises on the functional fitness of community older

© 2015 John Wiley & Sons Ltd

adults in the contemplation and preparation stages of behaviorial change.

Design

A quasi-experimental design with pre-test and post-tests on two groups was used in this study. Six senior activity centres in Southern Taiwan that volunteered to participate in the study were randomly assigned to either the experimental group (three centres) or the control group (three centres) using a lottery system. The functional fitness of the participants from the senior activity centres was assessed at baseline and after 3 and 6 months into the intervention.

Setting and participants

The study was conducted at six senior activity centres in Southern Taiwan. Approximately 30-35 older adults from each senior activity centre participated, which yielded a total sample size of 199 (experimental group $n = 97$, control group $n = 102$). The inclusion criteria stipulated that participants: (1) were older than 65 years; (2) had no prior elastic band exercise experience; (3) could stand independently without an assistive device; (4) had intact cognitive function as indicated by a Short Portable Mental Status Questionnaire score of 8-10 (Pfeiffer 1975); (5) were mildly dependent or independent in activities of daily living as indicated by a Barthel Index score of 91-100 (Mahoney 1965); and (6) were in the contemplation stage (not exercising, but had the intention to exercise within the next 6 months) or preparation stage (intending to begin exercising within 1 month or were exercising irregularly) according to the TTM. Regular activity indicated exercising for 30 minutes at least three times per week (Administration of Sports, Ministry of Education, Taiwan 2013). Participants with severe or acute cardiovascular, muscular-skeletal or pulmonary diseases were excluded from the study.

A total of 172 participants completed the 6-month study, thus yielded a retention rate of 86·43% (experimental group, $n = 87$; control group, $n = 85$). Participants with an attendance rate of lower than 50% (experimental group $n = 3$) were excluded from the data analysis to maintain a consistent intervention intensity. Hence, a total of 169 participants (experimental group $n = 84$, control group $n = 85$) were included in the data analysis. Based on the chi-square and *t*-test results, the characteristics of the participants who dropped out or were excluded from the final data analysis had no significant differences from those of the remaining participants (Figure 1).

Intervention

The experimental group participants engaged in the SEB exercise programme for 40 minutes three times per week for 6 months. The programme had three phases: (1) warm-up: seven movements in 12 minutes for loosening up the joints of the entire body; (2) aerobic motion: seven movements in 10 minutes for increasing cardiovascular capacity and improving balancing skill; and (3) static stretching: six movements in 18 minutes for enhancing muscle power and endurance and improving flexibility (Chen *et al.* 2013). Each exercise session was led by two to three certified instructors who had completed 9 hours of SEB training, which ensured the consistency of the interventions among the three senior activity centres in the experimental group (environmental reevaluation). According to the TTM, in the process of leading the group exercises, the certified instructors explained the purposes and benefits of each exercise design (consciousness raising), assisted the older adults to establish exercise goals (self-liberation), ensured the accuracy of each movement, determined the improvements of the participants and assisted participants to overcome exercise-related obstacles. The daily exercise duration and the goals of each participant were documented in written form. The participants who attended each session every month were awarded a certificate of excellence. The awarded participants were invited to share their success stories about exercising and the benefits of exercising to enhance their confidence and motivation to change (social liberation, dramatic relief and self-reevaluation). Participants in the control group did not receive any intervention and maintained their daily activities.

Data collection

The data were collected during 2011. The stages of participant behaviorial changes were examined using the exercise status questionnaire constructed by Reed *et al.* (1997). Participants indicated whether they exercised regularly. Regular exercise was defined as exercising for at least 30 minutes three times per week. The five response options were as follows: (1) 'No, I do not and I do not plan to regularly exercise within the next 6 months' (pre-contemplation); (2) 'No, I do not, but I plan to start exercising regularly within the next 6 months' (contemplation); (3) 'No, I do not, but I plan to start exercising regularly within the next month' (preparation); (4) 'Yes, I do, but the habit of exercising has not persistent for more than 6 months' (action); and (5) 'Yes, I do and I have exercised regularly for more than 6 months' (maintenance). Participants

© 2015 John Wiley & Sons Ltd

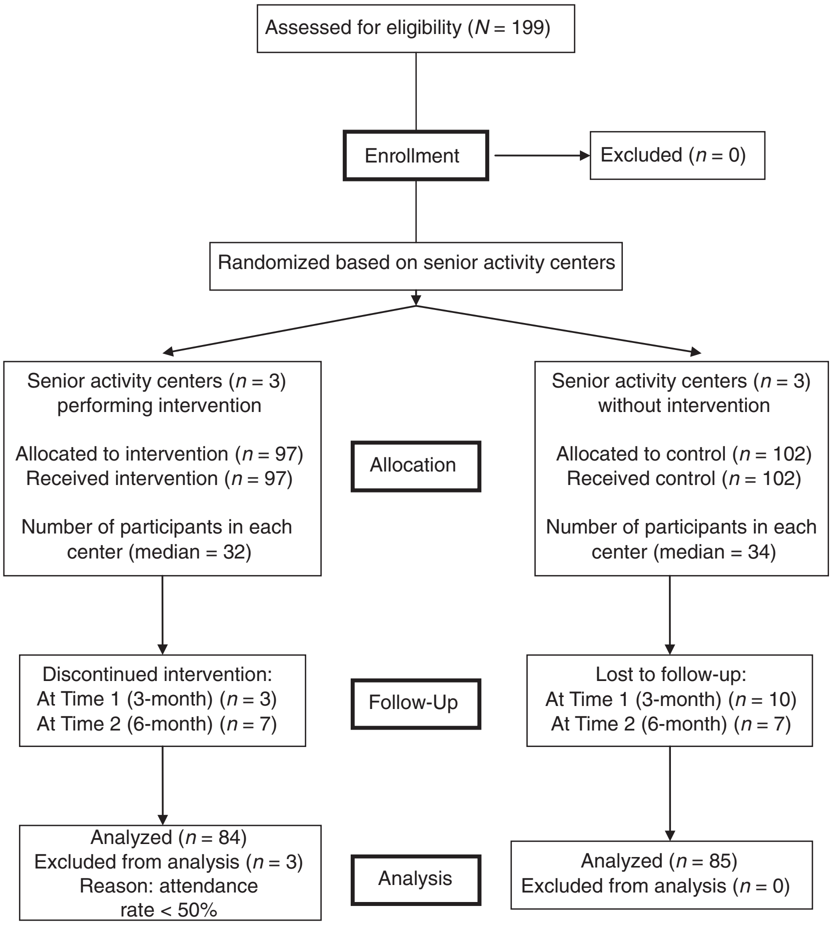

Figure 1 Flow of the participants.

selected the option that most appropriately described their current exercise status.

Functional fitness included lung capacity, cardiopulmonary fitness, upper and lower body flexibility, upper limb muscle power and lower limb muscle endurance. Lung capacity was measured using the TruZone™ Peak Flow Meter (Trudell Medical International, London). Participants drew a deep breath, placed the mouthpiece in their mouths and forcefully blew into the meter through the mouthpiece. This process was repeated twice and the optimal performance (in litters) was recorded (Chen *et al.* 2010). Cardiopulmonary fitness was measured using the 2-minute step test (Rikli & Jones 2001). Participants were instructed to stand straight and the middle distance between the patella and pelvic bones was marked on a wall with coloured tape. Participants stepped in a manner where their knees were raise above the marked spot and the number of steps within 2 minutes was recorded (Rikli & Jones 2001).

Upper body flexibility was measured by the back scratch test (Rikli & Jones 2001), where the participants reached the dominant hand back over the shoulder and the other hand behind the back, attempting to touch the middle fingers together; the distance (in centimetre) between the middle fingers was measured. Participants whose middle fingers did not touch received a negative score (−) and those whose fingers overlapped received a positive score (+) (Rikli & Jones 2001). Lower body flexibility was measured using the chair sit-and-reach test (Rikli & Jones 2001). Participants sat on the front one-third of a chair, bent one leg, placing the foot flat on the floor and extended the dominant leg as straight as possible in front of the hip, placing the heel on the floor and flexing the foot at approximately 90 degrees. Participants stretched out the arms, hands overlapping and slowly bent forward at the hip joint, reaching as far forward as possible towards or past the toes. The distance (in centimetre) between the middle fingers and the hallux of the dominant foot was recorded. Those whose middle fingers did not touch the hallux of the dominant foot received a negative score (−), whereas those whose middle fingers reached

 © 2015 John Wiley & Sons Ltd

beyond the toe received a positive score (+) (Rikli & Jones 2001).

Upper limb muscle power was measured using a digital handgrip dynamometer (TKK 5101; Takei Scientific Instruments, Tokyo, Japan) to test upper limb isometric contraction strength. Participants held the handle at the second joint on the index finger of the dominant hand, kept the forearms straight on both sides without bending or swinging back and forth and strongly gripped the handle. Two measurements were performed and the optimal result (in kilogram) was recorded (Rikli & Jones 2001). Lower limb muscle endurance was determined by recording the number of chair sit-to-stand repetitions performed in 60 seconds (Rikli & Jones 2001). Before the test, a chair without arm rests and wheels was placed against a wall. Participants sat on the chair, placed their feet on the ground, shoulder-width apart and crossed their hands against their chest parallel to their thighs. The number of completed standing and sitting motions in 60 seconds was recorded (Rikli & Jones 2001).

Ethical considerations

This study was approved by the Institutional Review Board of a university and the directors of the senior activity centres. All participants were informed of the study purpose by the researchers and consented to participation by signing a written consent form. During the study period, participants maintained the right to withdraw from the study at any time. All data were collected anonymously and were coded to maintain confidentiality.

Data analysis

After the data were collected, coded and archived, the Statistical Products and Services Solutions (SPSS) 19·0 software package for Windows was used for statistical analysis. Homogeneity tests were conducted on the demographic data of the participants. The categorical variables were analysed using the chi-square tests and the continuous variables were examined using the independent *t*-tests. The analysis results of the differences between the pre-test and the post-tests were highly dependent on the baseline. However, regardless of a statistically significant difference at baseline, analysis of variance (ANOVA) can be used for appropriate adjustment to reduce the risk of bias in outcomes (Overall & Magee 1992, Egbewale *et al.* 2014). Therefore, a two-way mixed design ANOVA was used to determine whether interactions existed between time and group. One-way repeated measures ANOVAs were conducted on variables with significant interaction effects to analyse the simple main effects of each group at three time points. An analysis of covariance (ANCOVA) was performed to examine the between-group differences after 3 and 6 months into the intervention. The pre-test data were used as covariates to eliminate the influences of possible group differences at baseline. Results were considered statistically significant when $P < 0{\cdot}05$.

Validity and reliability

The sample size was calculated by performing power analysis based on the data of our previous pilot study (Chan *et al.* 2015), where the value of alpha was set as 0·05, power = 0·90 and effect size = 0·2; thus, the required sample size was 166. Considering the attrition rate, the sample size was increased by 20%. Thus, 199 participants were needed. Participants were recruited conveniently from six senior activity centres and the centres were randomly assigned to either the experimental or control group using a lottery system. Data were collected by four research assistants using physical measurement tools and a structured questionnaire. To enhance the inter-rater consistency, the research assistants were trained together before the study began. Audits and quality-control meetings were regularly performed during data collection to ensure inter-rater consistency. The test-retest reliability of the exercise status questionnaire was $k \geq 0{\cdot}78$. The physical measurement tools were calibrated regularly by specialists. Two sequential trial tests were employed for each measurement device and the intraclass correlations were all above 0·90. In terms of data analysis, ANCOVA was used to adjust and reduce the risk of bias in outcomes due to baseline differences.

Results

Participants' Demographic Profiles

The average age of the participants was 71·28 (SD 5·54) years old; 115 participants (68%) were female, 166 participants were married (98·22%) and 126 participants (74·56%) had at least an elementary school education. More than half (57·40%) of the participants had an average of 0·83 chronic diseases (SD 0·87). Fifty-six participants (33·14%; experimental group = 27 participants, control group = 29 participants) were in the contemplation stage (not exercising, but had the intention to exercise within the next 6 months), whereas 113 participants (66·86%; experimental group = 57 participants, control group = 56 participants) were in the preparation stage (planning to begin

© 2015 John Wiley & Sons Ltd

exercising within a month or had been exercising irregularly). Further examination of the demographic differences between the two groups revealed that participants in the experimental group were younger than those in the control group ($t = -2.49$, $P = 0.014$) and the experimental group had more female participants than did the control group ($\chi^2 = 12.79$, $P < 0.001$). No significant differences in the remaining demographic characteristics and stages of change were found between the two groups.

Baseline comparisons between the two groups

In the pre-test, the upper and lower body flexibility of the experimental group participants was superior to that of the control group participants ($t = 3.08$, $P = 0.002$; $t = 2.25$, $P = 0.026$). However, the lung capacity and upper limb muscle power of the experimental group participants was inferior to that of the control group participants ($t = -2.18$, $P = 0.031$; $t = -2.69$, $P = 0.008$). No significant differences were found in the variables of cardiopulmonary fitness and lower limb muscle endurance between the two groups (all $P > 0.05$).

Interaction effects between different time points and different groups

A two-way mixed design ANOVA indicated that the two groups exhibited significant interactions at the three time points in all of the variables ($P < 0.001$).

Changes among three time points in each group

All six variables had significant changes among the pre-test and post-tests in the experimental group ($P < 0.001$). The post hoc comparisons indicated that the results of post-test II (6 months into the intervention) were superior to those of post-test I (3 months into the intervention) and the pretest (before intervention). Furthermore, the results of post-test I were superior to those of the pre-test (Table 1). In the beginning of the study, 97 experimental group participants were in the contemplation or preparation stages of the TTM and 84 participants (86·60%) switched to the action stage after 6 months of intervention.

As for the control group, only the lung capacity ($F = 3.61$, $P = 0.034$) and cardiopulmonary fitness ($F = 14.91$, $P < 0.001$) of the control group participants changed significantly at the three time points. However, the post hoc comparisons indicated that the lung capacity of the control group participants determined by post-test I was inferior to that determined by the pre-test; the cardiopulmonary fitness of the participants determined by post-test II was superior to that determined by post-test I and the pre-test (Table 2). By the end of the 6-month study, 85 control group participants (83·33%) remained in the contemplation or preparation stages of the TTM.

Differences of the two groups in each time point

After the pre-test data were controlled for as the covariate, the results indicated that the experimental group participants outperformed the control group participants in all of the variables 3 months ($P < 0.001$) (Table 3) and 6 months into the intervention ($P < 0.001$) (Table 4).

Discussion

The lung capacity, cardiopulmonary fitness, upper and lower body flexibility, upper limb muscle power and lower limb muscle endurance of the participants in the experimental group were significantly improved 3 months into the SEB intervention; this improvement continued throughout the 6 months of the study. The experimental group participants significantly outperformed the control group participants. These results were similar to those reported in

Table 1 Changes among three time points on variables with significant interaction effects in the experimental group ($n = 84$).

	Pre-test		Post-test I		Post-test II			
Variables	M	SD	M	SD	M	SD	F (P)	Post hoc†
Lung capacity (L)	305·02	96·96	319·76	106·54	342·14	116·02	25·08 (<0·001)***	Pre < PostI < PostII
Cardiopulmonary fitness (times)	78·43	21·77	95·19	19·93	107·74	17·57	103·47 (<0·001)***	Pre < PostI < PostII
Upper body flexibility (cm)	−3·13	9·90	−1·15	9·26	0·82	8·61	41·55 (<0·001)***	Pre < PostI < PostII
Lower body flexibility (cm)	7·32	10·48	9·07	10·09	12·43	9·70	48·96 (<0·001)***	Pre < PostI < PostII
Upper limb muscle power (kg)	22·13	6·95	24·10	6·77	24·62	7·29	44·47 (<0·001)***	Pre < PostI < PostII
Lower limb muscle endurance (times)	27·61	8·89	30·99	8·88	34·63	10·88	49·98 (<0·001)***	Pre < PostI < PostII

***$P < 0.001$.
†Bonferroni post hoc test (adjusting α levels of multiple tests).

 © 2015 John Wiley & Sons Ltd

Table 2 Changes among three time points on variables with significant interaction effects in the control group (n = 85).

	Pre-test		Post-test I		Post-test II			
Variables	M	SD	M	SD	M	SD	F (P)	Post hoc†
Lung capacity (L)	339·76	110·03	321·65	100·13	330·88	111·52	3·61 (0·034)*	Pre > Post I
Cardiopulmonary fitness (times)	74·28	26·32	77·81	26·61	86·71	27·40	14·91 (<0·001)***	Pre < Post II<br>Post I < Post II
Upper body flexibility (cm)	−8·38	12·17	−8·99	13·19	−8·37	12·82	0·58 (0·521)	–
Lower body flexibility (cm)	3·58	11·13	2·73	11·87	2·54	13·16	1·54 (0·218)	–
Upper limb muscle power (kg)	25·17	7·72	25·32	8·47	25·06	8·48	0·32 (0·712)	–
Lower limb muscle endurance (times)	27·72	8·23	27·14	8·56	28·00	9·64	1·06 (0·344)	–

*P < 0·05; ***P < 0·001.
†Bonferroni post hoc test (adjusting α levels of multiple tests).
– Post hoc analysis was not performed due to non-significant F value.

Table 3 Group differences on variables with significant interaction effects at third month of the study (N = 169).

Variables		Adjusted M	SS	d.f.	MS	F (P)
Lung capacity (L)	E	334·34	26881·23	1	26881·23	12·21 (0·001)**
	C	307·24				
Cardiopulmonary fitness (times)	E	94·04	8327·91	1	8327·91	36·57 (<0·001)***
	C	78·95				
Upper body flexibility (cm)	E	−3·67	292·73	1	292·73	12·51 (0·001)**
	C	−6·50				
Lower body flexibility (cm)	E	7·50	379·37	1	379·37	16·73 (<0·001)***
	C	4·28				
Upper limb muscle power (kg)	E	25·46	80·10	1	80·10	9·12 (0·003)**
	C	23·98				
Lower limb muscle endurance (times)	E	31·13	624·78	1	624·78	24·76 (<0·001)***
	C	27·00				

**P < 0·01; ***P < 0·001.
E, experimental group; C, control group.

Table 4 Group differences on variables with significant interaction effects at sixth month of the study (N = 169).

Variable		Adjusted M	SS	d.f.	MS	F (P)
Lung capacity (L)	E	358·23	68433·35	1	68433·35	17·60 (<0·001)***
	C	314·98				
Cardiopulmonary fitness (times)	E	107·33	14969·56	1	14969·56	39·78 (<0·001)***
	C	87·11				
Upper body flexibility (cm)	E	−1·60	700·77	1	700·77	22·25 (<0·001)***
	C	−5·97				
Lower body flexibility (cm)	E	10·87	1688·47	1	1688·47	48·53 (<0·001)***
	C	4·08				
Upper limb muscle power (kg)	E	26·09	225·37	1	225·37	29·16 (<0·001)***
	C	23·61				
Lower limb muscle endurance (times)	E	34·84	1820·23	1	1820·23	41·72 (<0·001)***
	C	27·79				

***P < 0·001.
E, experimental group; C, control group.

© 2015 John Wiley & Sons Ltd

relevant literature. For example, performing aerobic exercises improved the cardiopulmonary functions of older adults (Forster *et al.* 2009); resistance training increased muscle strength (Bird *et al.* 2009, Chang *et al.* 2012); and practicing aerobic and muscle exercises improved functional fitness (Martins *et al.* 2011). Based on the positive results found in this study, the SEB exercise programme was suggested to be further disseminated and applied to enhance the functional fitness of community older adults.

In addition, the drop-out rate in the experimental group was 10·30%, which was significantly lower than that in the control group (16·60%). Furthermore, the average attendance rate of participants in the experimental group was 84·58%. Overall, 97 experimental group participants were in the contemplation or preparation stages of the TTM and 84 participants (86·60%) switched to the action stage after 6 months of intervention. Therefore, providing group exercise courses and applying related enhancement strategies for older adults in the contemplation and preparation stages of behaviorial change according to the TTM were appropriate and beneficial. Interventions that correspond to the dynamic processes and principles of behaviorial change benefit strengthening exercise behaviours (Prochaska & Velicer 1997). The TTM is a useful model to be applied to form the regular physical exercise habits of community older adults.

Factors that hinder older adults from participating in physical exercise include poor memory, fatigue caused by exercise, insufficient funds for accessing exercise equipment and inadequate exercise facilities (Reichert *et al.* 2007, Ibrahim *et al.* 2013). The SEB exercise programme is easy to learn and convenient because only one elastic band is required for performing the exercises and the programme can be performed at home; therefore, older adults can exercise when transportation is unavailable, on rainy days or when they are incapable of going outside. The primary reasons for dropping out of the programme for the experimental group participants included unsatisfactory physical conditions (e.g. car accidents, previous wrist injuries) or the need to care for grandchildren. Such reasons were unrelated to willingness and differed from the factors that hindered older adults from performing physical exercises in previous studies (Reichert *et al.* 2007, Ibrahim *et al.* 2013). Therefore, the SEB exercise programme reduces the obstacles to exercising and promotes the maintenance of regular physical activity. The programme could be extended to involve participants from various ethnic groups and in different regions.

Although various physical activities are readily available and can be performed, not all of them are suitable for older adults. Older adults should select activities that are beneficial for improving their physical condition and that correspond to their physical and psychological fitness capacity. In addition, the obstacles that hinder older adults from exercising must be removed to maximize exercise outcomes. The proposed SEB exercise programme fulfils these requirements. Most of the functional fitness indicators of the participants improved significantly by the third month of the SEB programme intervention and these positive effects increased over the duration of the intervention, indicating that the SEB exercise programme can significantly improve the functional fitness of older adults, thus enhancing independence and reducing disability.

Unexpectedly, the cardiopulmonary fitness of the control group participants also improved in the third and sixth month of the study. For ethical consideration, 56 participants (65·88%) in the control group maintained their original irregular exercising habits. The main exercise of participants in the control group was calisthenics (n = 9; 18%) and health maintenance sports (n = 18; 36%). These exercises might have caused the enhanced cardiopulmonary fitness of the control group participants.

Study limitations

To prevent confounding effects on the experimental and control groups during interventions, the community centres, instead of the individual participants, were used as the unit for randomly assigning the participants into the two groups. Therefore, significant differences existed between the experimental and control group participants in the pretest about upper and lower body flexibility, lung capacity and upper limb muscle power. Subsequent statistical corrections were conducted on these significant differences by using ANCOVA. However, other uncontrollable factors, including available physical exercise options, environmental convenience and safety at the community centre, that causes the differences might have influenced the research results. Hence, these data must be interpreted with caution. Additionally, because of the limited human resources and time, a quasi-experimental research method and convenience sampling were employed in this study. The research results cannot be extended to older adults in other regions. Therefore, future studies could include older adults from various ethnic groups and regions as research participants and employ the TTM to enhance the sustainability of regular exercise habits. In addition, a long-term follow-up study of outcomes should be conducted to enhance the effects and benefits of the SEB exercise programme as a physical exercise option for older adults.

© 2015 John Wiley & Sons Ltd

Conclusion

The SEB exercise programme improved the functional fitness of community older adults. The programme is inexpensive, easy to use, safe and suitable to be applied in the community centres or practiced at home. Moreover, this study adopted the TTM to select participants who were in the contemplation and preparation stages of behaviorial change. Providing appropriate exercise prescriptions, such as the SEB exercise program, for people in the contemplation and preparation stages of behaviorial change can effectively facilitate regular exercise behaviours and enhance the functional fitness of community older adults. A long-term follow-up study of outcomes should be conducted to enhance the effects and benefits of the elastic band exercise programme as a physical exercise option for older adults. Future studies should develop various strategies that can be employed in different stages to facilitate exercise behaviour changes among older adults.

Acknowledgements

The authors sincerely appreciate the financial support by the National Science Council, Taiwan (NSC97-2314-B-037-052-MY3), and are grateful to Professor Frank Belcastro for editing the manuscript; to the administrators, staff and volunteers at the senior activity centres for their support and assistance; and to the 199 older adults for their generous participation.

Funding

This study was funded by the National Science Council, Taiwan (NSC97-2314-B-037-052-MY3).

Conflict of interest

No conflict of interest has been declared by the authors.

Author contributions

All authors have agreed on the final version and meet at least one of the following criteria [recommended by the ICMJE (http://www.icmje.org/recommendations/)]:

- substantial contributions to conception and design, acquisition of data, or analysis and interpretation of data;
- drafting the article or revising it critically for important intellectual content.

References

Administration of Sports, Ministry of Education, Taiwan (2013) Enhancement of the national fitness. Retrieved from http://www.sa.gov.tw/wSite/ct?xItem=12091&ctNode=320&mp=11 on 18 December 2013.

Bird M.L., Hill K., Ball M. & Williams A.D. (2009) Effects of resistance and flexibility: exercise interventions on balance and related measures in older adults. *Journal of Aging and Physical Activity* **17**(4), 444–454.

Chan S.Y., Kuo C.C., Chen K.M., Tseng W.S., Huang H.T. & Li C.H. (2015) Health promotion outcomes of a newly-developed elastic band exercise program for older adults in community: a pilot-testing. The Journal of Nursing Research. (in press).

Chang T.F., Liou T.H., Chen C.H., Huang Y.C. & Chang K.H. (2012) Effects of elastic-band exercise on lower-extremity function among female patients with osteoarthritis of the knee. *Disability and Rehabilitation* **34**(20), 1727–1735. doi:10.3109/09638288.2012.660598.

Chen K.M., Fan J.T., Wang H.H., Wu S.J., Li C.H. & Lin H.S. (2010) Silver yoga exercises improved physical fitness of transitional frail elders. *Nursing Research* **59**(5), 364–370. doi:10.1097/NNR.0b013e3181ef37d5.

Chen K.M., Tseng W.S., Huang H.T. & Li C.H. (2013) Development and feasibility of a senior elastic band exercise program for aged adults: a descriptive evaluation survey. *Journal of Manipulative and Physiological Therapeutics* **36**(8), 505–512.

Chodzko-Zajko W.J., Proctor D.N., Fiatarone-Singh M.A., Minson C.T., Nigg C.R., Salem G.J. & Skinner J.S. (2009) American College of Sports Medicine position stand: exercise and physical activity for older adults. *Medicine and Science in Sports and Exercise* **41**(7), 1510–1530. doi:10.1249/MSS.0b013e3181a0c95c.

Department of Statistics, Ministry of the Interior, Taiwan (2014) 103 years first three weeks in the internal affairs of statistics bulletin (end of 102 population structure analysis). Retrieved from http://www.moi.gov.tw/stat/news_content.aspx?sn=8057 on 01 March 2014.

Egbewale B.E., Lewis M. & Sim J. (2014) Bias, precision and statistical power of analysis of covariance in the analysis of randomized trials with baseline imbalance: a simulation study. *BioMed Central Medical Research Methodology* **14**(1), 49.

Forster A., Lambley R., Hardy J., Young J., Smith J., Green J. & Burns E. (2009) Rehabilitation for older people in long-term care. *Cochrane Database of Systematic Reviews (Online)* **1**, CD004294. doi: 10.1002/14651858.CD004294.pub2.

Gremeaux V., Gayda M., Lepers R., Sosner P., Juneau M. & Nigam A. (2012) Exercise and longevity. *Maturitas* **73**(4), 312–317. doi:10.1016/j.maturitas.2012.09.012.

Guralnik J.M., Ferrucci L., Simonsick E.M., Salive M.E. & Wallace R.B. (1995) Lower-extremity function in persons over the age of 70 years as a predictor of subsequent disability. *The New England Journal of Medicine* **332**(9), 556–561. doi:10.1056/nejm199503023320902.

Haddock B.L. & Wilkin L.D. (2010) Health-related variables and functional fitness among older adults. *The International Journal of Aging and Human Development* **70**(2), 107–118. doi:10.2190/AG.70.2.a.

© 2015 John Wiley & Sons Ltd

Ibrahim S., Karim N.A., Oon N.L. & Ngah W.Z. (2013) Perceived physical activity barriers related to body weight status and sociodemographic factors among Malaysian men in Klang Valley. *BioMed Central Public Health* **13**(1), 275. doi:10.1186/1471-2458-13-275.

Kao Y.H., Lu C.M. & Huang Y.C. (2002) Impact of a transtheoretical model on the psychosocial factors affecting exercise among workers. *The Journal of Nursing Research* **10**(4), 303–310.

Kim C.J., Hwang A.R. & Yoo J.S. (2004) The impact of a stage-matched intervention to promote exercise behavior in participants with type 2 diabetes. *International Journal of Nursing Studies* **41**(8), 833–841.

Mahoney F.I. (1965) Functional evaluation: the Barthel index. *Maryland State Medical Journal* **14**, 61–65.

Martins R., Coelho E., Silva M., Pindus D., Cumming S., Teixeira A. & Verissimo M. (2011) Effects of strength and aerobic-based training on functional fitness, mood and the relationship between fatness and mood in older adults. *The Journal of Sports Medicine and Physical Fitness* **51**(3), 489–496.

McAuley E., Jerome G.J., Marquez D.X., Elavsky S. & Blissmer B. (2003) Exercise self-efficacy in older adults: social, affective and behavioral influences. *Annals of Behavioral Medicine* **25**(1), 1–7.

McPhate L., Simek E.M. & Haines T.P. (2013) Program-related factors are associated with adherence to group exercise interventions for the prevention of falls: a systematic review. *Journal of Physiotherapy* **59**(2), 81–92.

Milanović Z., Pantelić S., Trajković N., Sporiš G., Kostić R. & James N. (2013) Age-related decrease in physical activity and functional fitness among elderly men and women. *Clinical Interventions in Aging* **8**, 549–556.

Nelson M.E., Rejeski W.J., Blair S.N., Duncan P.W., Judge J.O., King A.C., Macera C.A. & Castaneda-Sceppa C. (2007) Physical activity and public health in older adults: recommendation from the American College of Sports Medicine and the American Heart Association. *Circulation* **116**(9), 1094–1105. doi:10.1161/circulationaha.107.185650.

Nylen E.S., Kokkinos P., Myers J. & Faselis C. (2010) Prognostic effect of exercise capacity on mortality in older adults with diabetes mellitus. *Journal of the American Geriatrics Society* **58**(10), 1850–1854. doi:10.1111/j.1532-5415.2010.03068.x.

Overall J.E. & Magee K.N. (1992) Directional baseline differences and type I error probabilities in randomized clinical trials. *Journal of Biopharmaceutical Statistics* **2**(2), 189–203.

Pfeiffer E. (1975) A short portable mental status questionnaire for the assessment of organic brain deficit in elderly patients. *Journal of the American Geriatrics Society* **23**(10), 433–441.

Prochaska J.O. & DiClemente C.C. (1983) Stages and processes of self-change of smoking: toward an integrative model of change. *Journal of Consulting and Clinical Psychology* **51**(3), 390–395.

Prochaska J.O. & Marcus B.H. (1994) The transtheoretical model: applications to exercise. In *Advances in Exercise Adherence* (Dishman R.K., ed.), Human Kinetics, Champaign, IL, pp. 161–180.

Prochaska J.O. & Velicer W.F. (1997) The transtheoretical model of health behavior change. *American Journal of Health Promotion* **12**(1), 38–48.

Prochaska J.O., DiClemente C.C. & Norcross J.C. (1992) In search of how people change: applications to the addictive behaviors. *American Psychologist* **47**(9), 1102–1114.

Puthoff M.L. & Nielsen D.H. (2007) Relationships among impairments in lower-extremity strength and power, functional limitations and disability in older adults. *Physical Therapy* **87** (10), 1334–1347. doi:10.2522/ptj.20060176.

Reed G.R., Velicer W.F., Prochaska J.O., Rossi J.S. & Marcus B.H. (1997) What makes a good staging algorithm: examples from regular exercise. *American Journal of Health Promotion* **12** (1), 57–66. doi:10.4278/0890-1171-12.1.57.

Reichert F.F., Barros A.J., Domingues M.R. & Hallal P.C. (2007) The role of perceived personal barriers to engagement in leisure-time physical activity. *American Journal of Public Health* **97**(1), 515–519. doi:10.2105/ajph.2005.070144.

Rikli R.E. & Jones J.J. (2001) *Senior Fitness Test Manual*. Human Kinetics, Champaign, IL.

Sui X., LaMonte M.J., Laditka J.N., Hardin J.W., Chase N., Hooker S.P. & Blair S.N. (2007) Cardiorespiratory fitness and adiposity as mortality predictors in older adults. *The Journal of the American Medical Association* **298**(21), 2507–2516. doi:10.1001/jama.298.21.2507.

Tseng Y.H., Lin T.H. & Chen H.P. (2003) Application of the transtheoretical model to changing the exercise behavior of older adults. *The Journal of Nursing* **50**(4), 76–80. doi:10.6224/JN.50.4.76.

Warren J.M., Ekelund U., Besson H., Mezzani A., Geladas N. & Vanhees L. (2010) Assessment of physical activity: a review of methodologies with reference to epidemiological research. *European Journal of Cardiovascular Prevention & Rehabilitation* **17**(2), 127–139.

Weening-Dijksterhuis E., deGreef M.H., Scherder E.J., Slaets J.P. & van der Schans C.P. (2011) Frail institutionalized older persons: acomprehensive review on physical exercise, physical fitness, activities of daily living and quality-of-life. *American Journal of Physical Medicine & Rehabilitation* **90**(2), 156–168. doi:10.1097/PHM.0b013e3181f703ef.

World Health Organization (2013) The World Health Report 2002: Reducing Risks, Promoting Healthy Life. Retrieved from http://www.who.int/whr/2002/en/index.html on 03 November 2013.

 © 2015 John Wiley & Sons Ltd

The *Journal of Advanced Nursing (JAN)* is an international, peer-reviewed, scientific journal. *JAN* contributes to the advancement of evidence-based nursing, midwifery and health care by disseminating high quality research and scholarship of contemporary relevance and with potential to advance knowledge for practice, education, management or policy. *JAN* publishes research reviews, original research reports and methodological and theoretical papers.

For further information, please visit *JAN* on the Wiley Online Library website: www.wileyonlinelibrary.com/journal/jan

Reasons to publish your work in *JAN*:

- **High-impact forum:** the world's most cited nursing journal, with an Impact Factor of 1·527 – ranked 14/101 in the 2012 ISI Journal Citation Reports © (Nursing (Social Science).
- **Most read nursing journal in the world:** over 3 million articles downloaded online per year and accessible in over 10,000 libraries worldwide (including over 3,500 in developing countries with free or low cost access).
- **Fast and easy online submission:** online submission at http://mc.manuscriptcentral.com/jan.
- **Positive publishing experience:** rapid double-blind peer review with constructive feedback.
- **Rapid online publication in five weeks:** average time from final manuscript arriving in production to online publication.
- **Online Open:** the option to pay to make your article freely and openly accessible to non-subscribers upon publication on Wiley Online Library, as well as the option to deposit the article in your own or your funding agency's preferred archive (e.g. PubMed).

© 2015 John Wiley & Sons Ltd
