# Supplementary material for: Effects of elastic band resistance training on the physical and mental health of elderly individuals: A mixed methods systematic review
Source: PLoS One. 2024 May 13;19(5):e0303372. doi: 10.1371/journal.pone.0303372 (PMC11090353; doi:10.1371/journal.pone.0303372)
Supplement: S1 File — (ZIP) [file pone.0303372.s001.zip › Supporting Information/Included study 55.pdf]

Geriatric Nursing 34 (2013) 373–376

Contents lists available at ScienceDirect

Geriatric Nursing

journal homepage: www.gnjournal.com

Feature Article

# Feasibility appraisal of an elastic band exercise program for older adults in wheelchairs

Kuei-Min Chen, PhD, RN [a,*], Wei-Shyuan Tseng, BS [b], Ya-Hui Chang, BS [a], Hsin-Ting Huang, BS [a], Chun-Huw Li, MS, RN [c]

[a] *College of Nursing, Kaohsiung Medical University, 100 Shih-Chuan 1st Road, Sanmin District, Kaohsiung 80708, Taiwan*
[b] *Center for Continuing Education and Social Resources, Kaohsiung Medical University, Taiwan*
[c] *Department of Nursing, Yuhing Junior College of Health Care and Management, Taiwan*

ARTICLE INFO

*Article history:*
Received 6 February 2013
Received in revised form
13 May 2013
Accepted 18 May 2013
Available online 6 June 2013

*Keywords:*
Complementary therapy
Elastic band
Exercise program
Program review
Older adults in wheelchairs

ABSTRACT

This study appraised the feasibility of an elastic band exercise program for older adults in wheelchairs. A descriptive program review survey was used. A wheelchair-bound senior elastic band (WSEB) exercise program tailored to older adults in wheelchairs was initially developed by a group of 12 experts. A feasibility appraisal survey was administered to 10 older adults in wheelchairs through individual interviews after 4 weeks of the WSEB program. Study participants revealed that the WSEB program was feasible, safe, appropriate, and helpful to them. Participants further suggested practicing the WSEB program 3 times/week for 40 min/session in a group of 15–20 people. The finalized WSEB program has 2 levels: the basic and the advanced WSEB program. It is suggested that the basic level to be taught first with practice until participants are familiar with those exercises before proceeding to the advanced level.
© 2013 Mosby, Inc. All rights reserved.

## Introduction

Age-related decline in functional ability is associated with decline in skeletal muscle mass and voluntary strength,[1] which are essential in completing the activities of daily living and in maintaining an independent lifestyle.[2] In nursing homes, the number of older adults using wheelchairs is reported to be over 50% of the total population.[3] One of the major contributors to the need for wheelchair use is muscle weakness in the lower extremities due to previous falls, strokes, etc.[4] However, many of these individuals with muscle weakness may not require wheelchair use and have a high potential for rehabilitation.[4] Inappropriate and excessive reliance on wheelchairs restricts physical activities,[5] which in turns leads to an increased level of disability and mortality in older adults.[6]

Several wheelchair exercise programs have been developed and their beneficial effects have been reported. For example, a 16-week low-intensity aerobic wheelchair exercise on a treadmill (2 times/week, 30 min/session) improved fitness, upper-body health, and an active lifestyle for inactive persons with chronic spinal cord injury (SCI).[7] Also, an 8-week heavy-resistance exercise program (twice/week with 10–12 repetitions in 5 sets) was used with 8 male athletes with SCI (with another 8 healthy physical education students as the control group). Results indicated that athletes using wheelchairs showed a significantly higher improvement in the force development rate.[8] Another study found that athletes with quadriplegia who used wheelchairs playing rugby had better respiratory muscle strength and thoracic mobility than those sedentary subjects with quadriplegia.[9] Wheelchair rugby training has also been proven to be effective in increasing forced vital capacity, forced expired volume after 1 s, and maximal voluntary ventilation values for people with tetraplegia.[10] Furthermore, a group of researchers[11] applied a 12-week upper body physical training program on 30 frail older women sitting in wheelchairs. Significant improvements were found in arms strength and shoulder flexibility.[11]

Although these exercise programs were specifically designed for people in wheelchairs, most of these studies focused on people with SCI. Few studies focused on older adults only trained and evaluated their upper body strength. Brown, McCartney, and Sale[12] asserted that the muscles of older adults, both upper and lower extremities, are as trainable as those of their youthful counterparts. Since the reason for older adults in wheelchairs is due to muscle

This study was funded by the National Science Council, Taiwan (NSC99-2628-B-037-066-MY3).
* Corresponding author. Tel.: +886 7 3136900.
*E-mail addresses:* kmc@kmu.edu.tw, kueimin@yahoo.com (K.-M. Chen).

0197-4572/$ – see front matter © 2013 Mosby, Inc. All rights reserved.
http://dx.doi.org/10.1016/j.gerinurse.2013.05.005

weakness in the lower extremities not caused by the SCI, wheelchair exercise programs aimed at enhancing both upper and lower body strength are essential in preventing further deterioration and disuse syndromes.

Elastic band exercises are recognized as a safe and effective strategy to improve muscle strength[13,14] and increase the older adults' ability to perform functional tasks.[14–16] By changing the thickness and length of the elastic band, the level of resistance training can be flexibly increased or decreased.[17] Our research group developed a senior-tailored elastic band (SEB) exercise program to remedy the common physical weaknesses of community-dwelling older adults, such as poor cardiopulmonary fitness, decreased body flexibility, weak upper body muscle power, reduced lower body muscle endurance, poor balance, and sleep disturbances.[18] The SEB program included 3 phases with 20 exercises: 1) warm-up (7 exercises), 2) aerobic motion (7 exercises), and 3) static stretching (6 exercises).[19] Results from a study of 172 community-dwelling older adults showed significant positive health promotion outcomes, such as enhanced lung capacity, body flexibility, and muscle endurance of the lower extremities.[20]

However, whether the SEB program is applicable to those institutional older adults in wheelchairs with multiple morbidities is unknown since the health status of the institutional older adults in wheelchairs might be very different from community-dwelling older adults. Further, the literature contained few studies that focused on exercise programs that included both upper and lower body strength training. Thus, based on the experience of developing the SEB program and the consultations of 12 experts, our research group developed a new wheelchair exercise program specifically tailored for those older adults in wheelchairs due to muscle weakness, call the wheelchair-bound senior elastic band (WSEB). The purpose of this study was to appraise the feasibility of the WSEB exercise program for those older adults in wheelchairs.

## Materials and methods

### *Design*

A descriptive program review research was used. A feasibility appraisal survey was administered to 10 older adults in wheelchairs through face-to-face individual interviews after 4 weeks of the WSEB exercises. The study was approved by the Institutional Review Board of the University.

### *Setting and participants*

The study was conducted in a 49-bed assistive living facility located in southern Taiwan. Inclusion criteria were: 1) aged 65 and over, 2) older adults in wheelchairs, 3) living in the facility for at least 3 months, and 4) cognitively intact (a score of 8 or higher on the short portable mental status questionnaire; SPMSQ).[21] Exclusion criteria included: 1) having severe or acute cardiovascular, musculoskeletal, or pulmonary illness, or 2) suffering from an SCI with no rehabilitation potential. A convenience sample of 11 qualified participants was recruited and 10 of them completed the study (retention rate: 91%). Sample power was not analyzed due to the preliminary feasibility appraisal of the WSEB program.

### *Intervention*

The preliminary WSEB program was developed based on the consultations of 12 experts: 2 geriatricians, 2 gerontological nurse practitioners, 2 physical educators, 2 physical fitness trainers, 2 occupational therapists, and 2 social workers. The program included 3 phases: 1) warm-up: 6 exercises to loosen up the body and cultivate energy for a safe transition to the next phase (turning the wrists, pulling the arms, expanding the chest, elevating the knees, kicking the stone, and bouncing a shuttlecock); 2) aerobic motion: 6 low-to-medium speed exercises to stimulate cardiovascular-respiratory system (spreading the wings, boxing, raising hand, whipping out a sword, waving the trunk, and stepping and pushing); and 3) harmonic stretching: 6 low-speed, gentle stretching exercises to build up muscle strength/endurance and increase range of motion and flexibility (directing traffic, reaching the calf, separating the thigh, lifting the legs, pulling forward, and attacking the flank). The 18 elastic band exercises took 40 min to complete. The descriptions of some exercises in the WSEB program are provided in Table 1.

### *Data collection*

Ten participants were interviewed individually after 4 weeks of the WSEB group practice (3 times/week, 40 min/session) to appraise the feasibility of the WSEB. Participants were asked to rate the level of simplicity, safety, appropriateness, and helpfulness of the program on a 10-point Cantril ladder scale,[22] ranging from 0 to 10 with 0 meaning the exercise is very difficult to perform, very dangerous, very inappropriate, and useless in health promotion for older adults in wheelchairs; 10 indicating the exercise is very easy to perform, very safe, very appropriate, and very helpful in health promotion for older adults in wheelchairs. In addition, 4 open-ended questions asked participants to reflect on their elastic band exercise experiences and to suggest program protocol: 1) How do you feel after 4 weeks of the WSEB exercises? 2) How many times per week of elastic band exercise is appropriate? 3) How long per exercise session is appropriate? 4) How many people per group are appropriate in doing elastic band exercises? The researcher delivered the questions verbally to the participants and marked their verbal answers on the evaluation forms. The seniors' feedback was taken into consideration in revising the WSEB program.

### *Data analysis*

The statistical package for the social sciences (SPSS) version 17.0 was used to analyze the data. Descriptive statistics were used to

**Table 1**
Descriptions of one exercise in each phase of the wheelchair-bound senior elastic band exercise program.

| Phase: exercise | Descriptions |
|---|---|
| Warm-up: turning the wrists | 1. Sit up-straight, legs open as wide as the shoulders, raise hands in the front as high as the shoulders, and slightly bend the elbows.<br>2. Turn the left forearm and wrist from inward to outward for 6 times.<br>3. Same procedure to the right-hand side for 6 times.<br>4. Turn both forearms and wrists from inward to outward for 6 times. |
| Aerobic motion: stepping and pushing | 1. Sit up-straight, legs open as wide as the shoulders, and hold the elastic band in hands in front of the chest.<br>2. Stepping on the floor on the same point while both hands are pushing forward and backward as high as the shoulders for 6 8-beat times. |
| Harmonic stretching: separating the thigh | 1. Sit up-straight with legs closed, put the elastic band around the thigh, and hold 2 ends of the elastic band in hands.<br>2. Move the legs outward while holding the breath for 5 s.<br>3. Exhale and move the legs inward and touch the knees.<br>4. Repeat outward and inward for 6 times. |

describe the characteristics of the participants and their feasibility appraisal of the WSEB program. The qualitative comments and suggestions of the participants were critically analyzed and summarized into categories and presented in frequency distributions.

## Results

### *Profiles of the participants*

The mean age of the participants was 82.10 ± 5.86 years: young-old senior (65–74 years old; $n = 1$), middle-old senior (75–84 years old; $n = 5$), and old–old senior (85 years old and above; $n = 4$). More than half of the participants were female ($n = 6$), widowed ($n = 7$), and had no education ($n = 6$). The cognitive functioning of the participants was intact with a mean SPMSQ score of 9.70 ± 0.58. All of the participants had at least one chronic illness, with a mean of 1.50 ± 0.71. The top 3 chronic illnesses reported by the participants were hypertension ($n = 7$), diabetes ($n = 4$), and heart disease ($n = 2$). An average Barthel index (BI) score of 60.50 ± 32.36 indicated that participants were severely (BI = 21–60; $n = 5$) to moderately (BI = 61–90; $n = 5$) dependent in self-care, such as bathing, eating, and toileting.[23]

### *WSEB program feasibility appraisal*

The average ratings of the participants on the 4 evaluation criteria of the WSEB program ranged from 9.22 ± 0.83 to 9.78 ± 0.67 (Table 2). In responding to the first open-ended question as to how the participants felt after 4 weeks of the WSEB exercises, the participants noted that they had more muscle strength in both hands and legs ($n = 6$), had increased body flexibility and range of joint motion ($n = 5$), and felt more energetic ($n = 5$). All of them ($N = 10$) expressed that they would like to continue the WSEB exercises. As for the program protocol suggestions (open-ended questions #2–#4), most participants preferred to take part in the WSEB program 3 times/week ($n = 9$), and most of them ($n = 6$) felt the original 40 min program was doable. Finally, most participants preferred to do the elastic band exercises in a group of 15–20 people ($n = 6$) (Table 3).

## Discussion

The participants gave high scores on the 4 evaluation criteria of the WSEB program, indicating that the program was feasible, safe, appropriate, and helpful to older adults in wheelchairs. However, in responding to the program protocol suggestions in the open-ended questions, the participants suggested that it would be better if the program was divided into basic and advanced levels. Therefore, the finalized WSEB program is designed to have 2 stages, and the sequence of the exercises has been changed accordingly.

The basic WSEB program has 4 exercises in each phase: 1) warm-up: turning the wrists, expanding the chest, elevating the knees, and bouncing a shuttlecock; 2) aerobic motion: boxing, raising hand, waving the trunk, and stepping and pushing; and 3) harmonic stretching: directing traffic, pulling forward, attacking the flank, and separating the thigh. As for the advanced WSEB program, 2 relatively challenging elastic band exercises in each phase as identified by the experts were added: 1) warm-up: kicking the stone and pulling the arms; 2) aerobic motion: spreading the wings and whipping out a sword; and 3) harmonic stretching: lifting the legs and reaching the calf. Therefore, the advanced WSEB program has 6 exercises in each of the 3 phases.

While instructing the older adults to practice the WSEB exercises, it is suggested that the basic level be taught first with practice until participants are familiar with those exercises. The instructors can then teach the new exercises in the advanced level. Most importantly, daily practice of the advanced WSEB program is essential to the success of the program. A 6-month teaching and practicing protocol of the WSEB is provided in Table 4.

The WSEB program is different from those traditional elastic band exercises in that the exercises in the WSEB program are less strenuous to accommodate reduced muscle strength and body flexibility experienced by many older adults, and the thickness of the elastic band is medium so that the level of resistance training can be easily increased or decreased to accommodate the muscle strength of older adults. The WSEB program is also different from the SEB program for the following reasons: 1) the WSEB program has 2 levels (basic and advanced) to adjust for the learner's needs; however, the SEB program has only one level; 2) although both the SEB and WSEB programs have 3 phases and the structures of the programs are similar, the SEB program has a total of 20 elastic band exercises which differ from the 18 exercises in the WSEB program; and 3) all of the SEB exercises are performed in a standing position; however, the elastic band exercises in the WSEB program are executed while sitting in a wheelchair. The exercises in the WSEB program have to accommodate the special characteristics of the wheelchair, and some exercises are carried out with the use of the wheelchair's handrail.

**Table 2**
Feasibility appraisal of the wheelchair-bound senior elastic band exercise program ($N = 10$).

| Criteria phases | Simplicity ($M$ ± SD) | Safety ($M$ ± SD) | Appropriateness ($M$ ± SD) | Helpfulness ($M$ ± SD) |
|---|---|---|---|---|
| Warm-up | 9.44 ± 0.73 | 9.78 ± 0.67 | 9.33 ± 0.87 | 9.44 ± 0.88 |
| Aerobic motion | 9.33 ± 0.71 | 9.56 ± 0.73 | 9.33 ± 0.71 | 9.44 ± 0.73 |
| Harmonic stretching | 9.22 ± 0.83 | 9.67 ± 0.71 | 9.33 ± 0.71 | 9.22 ± 0.83 |

**Table 3**
Suggestions for the wheelchair-bound senior elastic band program protocol ($N = 10$).

| Variables | $f$ |
|---|---|
| Exercise frequency (times/week) | |
| 3 | 9 |
| 7 | 1 |
| Exercise duration (min/session) | |
| 30 | 3 |
| 40 | 6 |
| 60 | 1 |
| Group size (number of people) | |
| <10 | 4 |
| 15–20 | 6 |

**Table 4**
A 6-month teaching and practicing protocol of the wheelchair-bound senior elastic band exercise program.

| Sessions[a] | Exercises |
|---|---|
| 1–4 | Teach 4 warm-up exercises in the basic level WSEB |
| 5–8 | Teach 4 aerobic motion exercises in the basic level WSEB |
| 9–12 | Teach 4 harmonic stretching exercises in the basic level WSEB |
| 13–36 | Practice the basic level WSEB 3 times/week |
| 37–40 | Teach 2 relatively challenging warm-up exercises in the advanced level WSEB |
| 41–44 | Teach 2 relatively challenging aerobic motion exercises in the advanced level WSEB |
| 45–48 | Teach 2 relatively challenging harmonic stretching exercises in the advanced level WSEB |
| 49–72 | Practice the advanced level WSEB 3 times/week |

[a] Each session lasts 35 min and has a 5 min break in the middle of the session.

The profiles of this group of older adults in wheelchairs indicated that they were mostly middle–old or old–old older adults with severe or moderate dependency levels for the activities of daily living, and all of them had at least one chronic illness. Although the sample size was small and the effects of the WSEB program were not formally tested, these relatively frail older adults had a positive feedback after 1 month in the WSEB program. Since no negative reactions were reported and the program appraisal from the participants resulted in high scores, the WSEB program shows initial promise for older adults in wheelchairs.

Finally, the study participants suggested practicing WSEB program 3 times/week, 40 min/practice, and with a group of 15–20 people. These suggestions are different from the SEB protocol that were suggested by the community-dwelling seniors: 3 times/week, 60 min/session and in a large group of 20–29 people.[19] These differences might be due to differences in learning abilities and functional abilities between the institutional older adults in wheelchairs and community-dwelling older adults. These findings further confirmed that not all older adults should receive the same intervention to improve muscle strength. Attention must be given to the needs of various sub-groups of the senior populations. When the WSEB program is used with older adults in long-term care facilities, it would be important to start with simple, basic, and not too strenuous elastic band exercises, teach them step-by-step allowing them enough time to become acquainted with those exercises, and then move on to the advanced level. With this gradual training pattern, the older adults would build up their confidence, keep their interest in the program, and grow in a sense of achievement, which in turn may increase their adherence to the program.

### *Study limitations*

A small convenience sample of 10 people in this study could possibly limit a variety of feedbacks that might have been revealed from a larger probability sample. Further, the positive feedbacks expressed by the participants, such as having more muscle strength in hands and legs, increased body flexibility and range of joint motion, and feelings of being more energetic, were subjective and no measurements were made to verify these findings. Since a formal outcome evaluation of the WSEB program was not conducted, a pilot study using objective measurements is needed to test the effects of the WSEB program.

## Acknowledgments

Sincere appreciation is directed by our group to Professor Frank Belcastro for his superlative manuscript editing, to the 12 experts in the advisory panel for their constructive suggestions, to the 10 wonderful seniors for their generous participation, and to the administrator and staff of the assistive living facility for their support and assistance.

## References

1. Doherty TJ. Aging and sarcopenia. *J Appl Physiol*. 2003;95:1717–1727.
2. Topp R, Mikesky A, Bawel K. Developing a strength training program for older adults: planning, programming, and potential outcomes. *Rehabil Nurs*. 1994;19:266–297.
3. Brechtelsbauer DA, Louie A. Wheelchair use among long-term care residents. *Ann Longterm Care*. 1999;7:213–220.
4. Karmarkar AM, Dicianno BE, Cooper R, et al. Demographic profile of older adults using wheeled mobility devices. *J Aging Res*. 2011;2011(560358):1–11. http://dx.doi.org/10.4061/2011/560358.
5. Rimmer JH. Exercise and physical activity in persons aging with a physical disability. *Phys Med Rehabil Clin N Am*. 2005;16:41–56.
6. Hirvensalo M, Rantanen T, Heikkinen E. Mobility difficulties and physical activity as predictors of mortality and loss of independence in the community-living older population. *J Am Geriatr Soc*. 2000;48:493–498.
7. van der Scheer JW, de Groot S, Postema K, et al. Design of a randomized-controlled trial on low-intensity aerobic wheelchair exercise for inactive persons with chronic spinal cord injury. *Disabil Rehabil*; 2012:1–8. http://dx.doi.org/10.3109/09638288.2012.709301.
8. Turbanski S, Schmidtbleicher D. Effects of heavy resistance training on strength and power in upper extremities in wheelchair athletes. *J Strength Cond Res*. 2010;24:8–16.
9. Moreno MA, Zamunér AR, Paris JV, et al. Effects of wheelchair sports on respiratory muscle strength and thoracic mobility of individuals with spinal cord injury. *Am J Phys Med Rehabil*. 2012;91:470–477.
10. Moreno MA, Paris JV, Sarro KJ, et al. Wheelchair rugby improves pulmonary function in people with tetraplegia after 1 year of training. *J Strength Cond Res*. 2013;27:50–56.
11. Venturelli M, Lanza M, Muti E, et al. Positive effects of physical training in activity of daily living-dependent older adults. *Exp Aging Res*. 2010;36:190–205.
12. Brown AB, McCartney N, Sale DS. Positive adaptations to weight-lifting training in the elderly. *J Appl Physiol*. 1990;69:1725–1733.
13. Chen YJ, Huang TH, Lin LC. Elastic band resistance training improved muscular fitness in older adults. *Sports Res Rev*. 2011;113:77–86.
14. Fahlman MM, McNevin N, Boardley D, et al. Effects of resistance training on functional ability in elderly individuals. *Am J Health Promot*. 2011;25:237–243.
15. Chang TF, Liou TH, Chen CH, et al. Effects of elastic-band exercise on lower-extremity function among female patients with osteoarthritis of the knee. *Disabil Rehabil*. 2012;34:1727–1735.
16. Patil P, Rao SA. Effects of Thera-Band® elastic resistance-assisted gait training in stroke patients: a pilot study. *Eur J Phys Rehabil Med*. 2011;47:427–433.
17. Damush TM, Damush JG. The effects of strength training on strength and health-related quality of life in older adult women. *Gerontologist*. 1999;39:705–710.
18. Chen KM, Huang HT, Lin MH, et al. *Physical Health of Community-dwelling Older Adults: Live Longer and Stay Healthier*. Presented at the 41st Biennial Convention of Sigma Theta Tau International, Grapevine, Texas, USA; 2011.
19. Chen KM, Tseng WS, Huang HT, et al. *Development and Evaluation of a Senior-tailored Elastic Band Exercise Program*. Presented at the 23rd International Nursing Research Congress, Brisbane, Australia; 2012.
20. Chen KM. *Developing, Establishing, and Testing the Health Indicators, Health Assessment Toolkit, and Health Promotion Program for Older Adults: A Final Report*. Taipei, Taiwan: National Science Council; 2011.
21. Pfeiffer E. A short portable mental status questionnaire for the assessment of organic brain deficit in elderly patients. *J Am Geriatr Soc*. 1975;23:433–441.
22. Cantril H, Kilpatrick FP. Self-anchoring scaling: a measure of individuals' unique reality worlds. *J Individ Psychol*. 1960;16:158–173.
23. Shah S, Vanclay F, Cooper B. Improving the sensitivity of the Barthel Index for stroke rehabilitation. *J Clin Epidemiol*. 1989;42:703–709.
