# Supplementary material for: Effects of elastic band resistance training on the physical and mental health of elderly individuals: A mixed methods systematic review
Source: PLoS One. 2024 May 13;19(5):e0303372. doi: 10.1371/journal.pone.0303372 (PMC11090353; doi:10.1371/journal.pone.0303372)
Supplement: S1 File — (ZIP) [file pone.0303372.s001.zip › Supporting Information/Included study 41.pdf]

Experimental Gerontology 147 (2021) 111272

Contents lists available at ScienceDirect

Experimental Gerontology

journal homepage: www.elsevier.com/locate/expgero

# Effects of progressive elastic band resistance exercise for aged osteosarcopenic adiposity women☆

Yu-Hao Lee[a,1], Pi-Hsia Lee[b,1], Li-Fong Lin[a,c], Chun-De Liao[a,d], Tsan-Hon Liou[a,e,g,2], Shih-Wei Huang[a,e,f,*,2]

[a] Department of Physical Medicine and Rehabilitation, Shuang Ho Hospital, Taipei Medical University, Taipei, Taiwan
[b] School of Nursing, College of Nursing, Taipei Medical University, Taipei 10675, Taiwan
[c] Institute of Gerontology and Health Management, Taipei Medical University, Taipei, Taiwan
[d] School and Graduate Institute of Physical Therapy, College of Medicine, National Taiwan University, Taipei, Taiwan
[e] Department of Physical Medicine and Rehabilitation, School of Medicine, College of Medicine, Taipei Medical University, Taipei, Taiwan
[f] Graduate Institute of Sports Science, National Taiwan Sport University, Taoyuan, Taiwan
[g] Department of Physical Medicine and Rehabilitation, Wan Fang Hospital, Taipei Medical University, Taiwan

ARTICLE INFO

Section Editor: Anna-Karin Welmer

*Keywords:*
Osteopenia
Sarcopenia
Obesity
Osteosarcopenic obesity
Resistance exercise
Elderly women, osteosarcopenic adiposity

ABSTRACT

*Purpose:* Osteosarcopenic adiposity (OSA), which is described as the concurrent occurrence of osteopenia, sarcopenia, and adiposity, can lead to frailty and increase the risk of physical disability in elderly women. Progressive elastic band resistance exercise training (peRET) is considered a safe and feasible exercise intervention for elderly women with sarcopenic obesity. This study investigated the effects of elastic band resistance exercise on the physical capacity and body composition of elderly women with osteosarcopenic adiposity.
*Method:* A total of 15 and 12 women were randomly assigned to the experimental (12 weeks of resistance exercise) and control groups (no exercise intervention), respectively. Lean mass (measured using a dual-energy X-ray absorptiometer) and physical capacity assessments (such as timed up and go test and single leg stance tests) were conducted at baseline, 12 weeks (end of intervention), and 6 months after the intervention. Outcome differences within the study and control groups were analyzed using repeated-measures analysis of variance with a post-hoc test. The Mann–Whitney *U* test was used to examine differences between groups at different time points.
*Results:* After the intervention, no body composition changes in muscle mass and fat were observed between the study and control groups. Moreover, muscle mass and fat body composition did not significantly differ at different time points. The bone density was higher in the study group, with a higher T-score than their baseline values, but did not significantly differ compared with the control group. The study group exhibited more improved physical function than the control group, but the effect did not last after 6 months of follow-up.
*Conclusions:* A 12-week progressive elastic band resistance training program effectively increased the physical capacity and improved the bone density; however, without persistent training, the positive effect diminished at 6-month follow-up.

## 1. Introduction

Aging causes unfavorable changes in body composition, such as a decrease in muscle mass and an increase in fat mass (Ding et al., 2007). Although a universally accepted definition for sarcopenia is lacking, it usually refers to the loss of muscle mass and physical function (Dawson-Hughes and Bischoff-Ferrari, 2016). Sarcopenia is associated with increased risks of falls, fractures, and mortality (Hong et al., 2015;

☆ Clinical trial registration number: ChiCTR-IPR-15006069.

* Corresponding author at: Department of Physical Medicine and Rehabilitation, Shuang Ho Hospital, Taipei Medical University, 291 Jhongjheng Rd, Jhonghe, New Taipei City 235, Taiwan.
*E-mail address:* 13001@s.tmu.edu.tw (S.-W. Huang).

[1] Yu-Hao Lee and Pi-Hsia Lee equally contributed to this study.
[2] Tsan-Hon Liou and Shih-Wei Huang equally contributed to this study.

https://doi.org/10.1016/j.exger.2021.111272
Received 16 November 2020; Received in revised form 29 January 2021; Accepted 2 February 2021
Available online 4 February 2021
0531-5565/© 2021 Elsevier Inc. All rights reserved.

Wijnhoven et al., 2012; Newman et al., 2006). Sarcopenic obesity, a condition in which sarcopenia coexists with obesity, has been reported to exert an additive negative effect on all-cause mortality (Van Aller et al., 2019; Atkins et al., 2014), increase the risks of cognitive impairment (Tolea et al., 2018) and cardiovascular disease (Stephen and Janssen, 2009), and lead to frailty and the inability to partake in daily living activities (Hirani et al., 2017). Thus, researchers have been paying increased attention to this condition. Most recent studies on managing sarcopenic obesity have involved exercise (resistance training and/or aerobic training) with nutritional interventions; however, an optimal regimen has not yet been established (Trouwborst et al., 2018). Bone mineral loss is another crucial problem among older adults, particularly postmenstrual women. According to World Health Organization (WHO) criteria, patients with bone mineral density (BMD) T-scores of less than −1 and −2.5 are diagnosed as having osteopenia and osteoporosis, respectively (WHO, 2007). At low BMD, the bones become fragile and the risk of fracture increases, eventually leading to increased morbidity and mortality and decreased quality of life (Romagnoli et al., 2004). Siris et al. (Kanis et al., 2013) reported that a 50-year-old white woman with osteopenia had a 16% risk of fracturing a hip, and the risk increased to 33% with the coexistence of osteoporosis. General management to prevent falls and treat osteopenia or osteoporosis includes having a balanced diet, consuming adequate amounts of calcium and vitamin D, performing appropriate weight-bearing or resistance exercise, and avoiding smoking and excessive alcohol consumption. For patients with advanced bone loss or high fracture risk, pharmacological intervention is recommended (Kanis et al., 2013; Menopause, 2010).

Sarcopenia and osteopenia (or osteoporosis) have similar risk factors and biological pathways (Curtis et al., 2015). Binkley and Buehring (2009) first described the coexistence of these two conditions by using the term “sarco-osteopenia” or “sarco-osteoporosis”; patients with both these conditions have increased risks of falls and fractures, increased morbidity and mortality, and lower quality of life. Moreover, in some individuals, BMD does not increase with body weight when muscle mass is inadequate (Sowers et al., 1992; Stenholm et al., 2008). Thus, a new term, “osteosarcopenic obesity (OSO)” was proposed by specialists (Ilich et al., 2014) to encompass the conditions of bone and muscle loss occurring concurrently with obesity. Subsequent cohort studies have reported that OSO is prevalent among middle-aged and older adults and is independently related to frailty and poor physical performance in middle-aged and older women (Chung et al., 2016; Szlejf et al., 2017). The possible causes of OSO include nutritional deficiency and low-grade chronic inflammation related to obesity or other chronic medical conditions, such as cancer, diabetes, and endocrine imbalance (Ilich et al., 2016). Because of the heterogeneity of adipose tissue had been recognized recently (Alalwan, 2020), in 2020, OSO had been change to osteosarcopenic adiposity (OSA) by Ilich et al. based on the fact that adiposity includes all kinds of the body fat while obesity typically refers more to overweight (Ilich et al., 2020).

Unlike interventions for sarcopenic obesity or osteoporosis alone, those for OSA have not been well investigated. Resistance training has been recognized as an effective strategy for stimulating an osteogenic response, preserving BMD, and preventing aging-related muscle mass attenuation and loss of leg strength in older adults (Ryan et al., 2004; Liao et al., 2017; Calle and Fernandez, 2010). Among the various resistance training exercises, progressive elastic band resistance exercise training (peRET) is a safe, simple, relatively low-cost, and effective training method characterized by a dynamic form with different stretching ranges and speeds, wherein resistance strength is determined by the stretching tension during exercise. peRET has demonstrated positive effects on muscle strength and physical function in older people with disabilities (Chen et al., 2013; Hofmann et al., 2016). In patients with sarcopenic obesity, peRET improved body composition (including BMD), muscle quality, and physical function (Huang et al., 2017; Liao et al., 2018). To the best of our knowledge, the effect of peRET on elderly women with OSA has not yet been investigated. Therefore, the present randomized controlled study investigated the effect of peRET on the body compositions and physical function of elderly women with OSA. We hypothesized that peRET can improve body compositions and physical function of elderly OSA women.

## 2. Methods

### 2.1. Participant recruitment

Participants were recruited from a local community proximal to a university hospital by using posters or flyers between April 2015 and April 2016.

### 2.2. Screening evaluation

In this study, we included postmenopausal women aged between 60 and 90 years who had been diagnosed as having OSA. In the screening stage, sarcopenia was defined in our study using the method reported by Janssen et al., (2002). Total skeletal muscle mass (TSM, kg) was measured using an 8-polar bioelectrical impedance analysis (BIA) device with multifrequency current (Inbody™ 220, Biospace, Seoul, Republic of Korea). The validity of the device in estimating TSM has been verified (Anderson et al., 2012). According to Janssen et al., TSM can be estimated using the following equation: $TSM = [(Ht^2/BIA\text{-}R) \times 0.401 + (sex \times 3.825) + (age \times -0.071) + 5.102]$, where Ht is the individual's height in cm, BIA-R is BIA resistance in ohms, the value of sex is assigned as 1 for men and 0 for women, and age is calculated in years (Janssen et al., 2000). Percentage skeletal muscle mass index (SMI%) is then calculated from TSM by using the following formula: $SMI = TSM/\text{total body mass} \times 100\%$.

### 2.3. Inclusion and exclusion criteria

After the patients were screened, muscle mass and BMD were determined using a Hologic QDR-1000/W whole body dual-energy X-ray absorptiometer (Hologic, Waltham, MA, USA). Hologic enhanced whole-body analysis software (version 5.71) was used to provide estimates of the following components: whole body lean mass (kg), TSM (kg), and ALM (kg). AMI ($kg/m^2$) and LMI ($kg/m^2$), are calculated as the ALM and whole body lean mass divided by squared height in meters, respectively. SMI (%) was also calculated mentioned above. Participants who met the criteria of the European Working Group on Sarcopenia in Older People (EWGSOP) (Cruz-Jentoft et al., 2010) of low muscle mass (less than 5.67 $kg/m^2$ based on DXA) and a grip strength of <20 kg or gait speed (GS) of <0.8 m/s were diagnosed with sarcopenia.

Osteopenia was diagnosed according to the aforementioned WHO criteria (T-score < −1.0).We measured BMD by using the standard protocol. Lumbar spine BMD was determined through the imaging of lumbar vertebra one through four (L1-L4), which included the body of the vertebra, the pedicles, lamina, spinous process, and transverse processes. The coefficient of variation in BMD determined using a spine phantom (Foley et al., 2010), total body lean mass measurement, and fat mass measurement were 0.39%, 0.5%, and 1.5%, respectively (Sakai et al., 2006).

The participants' body fat percentage (BF%) were calculated from the data obtained from DXA; participants with BF% > 35% were categorized as obese (Li et al., 2012).

Participants were excluded if they had any of the following conditions: (1) neurological impairment or cognitive impairment caused by previous stroke or traumatic brain injury; (2) severe musculoskeletal disorders, such as a joint swelling or contracture, that could prevent them from performing exercise; (3) history of receiving arthroplasty or artificial implants, which could influence the accuracy of the body composition analysis; and (4) unstable cardiopulmonary disease, such as uncontrolled hypertension and heart failure, which may restrict patients from tolerating resistance training. Initially, 54 patients were

determined to be eligible for this study; of them, 24 were excluded because they did not meet the inclusion criteria and 3 declined to participate. Finally, 27 participants were enrolled for randomization: 15 were allocated to the study group and 12 to the control group (Fig. 1).

### 2.4. Ethics

After explaining the study protocol to all participants, we obtained their written informed consent. This study was approved by the Joint Institutional Review Board of Taipei Medical University (trial number: 201306019) and registered at the Chinese Clinical Trial Registry (trial number: ChiCTR-IPR-15006069) on April 3, 2015. The study protocol conforms to all CONSORT guidelines and reports the required information accordingly.

### 2.5. Study design and randomization

This study is a prospective, single-blinded, randomized, controlled trial. After the assessment, eligible participants were randomized into the experimental group (peRET group) and control group. For randomization, an independent administrative assistant provided participants with concealed envelopes containing allocation instructions; allocations were based on a list of random numbers that had been computer-generated by an independent randomization center. Assessors (a reporting radiologist and a statistician) were blinded to the participant allocation status.

### 2.6. Intervention and control groups

The peRET group underwent a 12-week training program. Participants in this group performed individual resistance exercises using elastic bands (Thera-Band®, The Hygenic Corporation, Akron, OH, USA) under the supervision of senior licensed physical therapists and in accordance with a treatment manual designed in this study. Our training program followed American College of Sports Medicine guidelines (Nelson et al., 2007) for older adults. The exercise program was performed 3 times per week, with a total of 36 sessions in 12 weeks (Liao et al., 2018). Participants were divided into small groups of less than six people during training sessions. Each session consisted of 10 min of warm-up exercises followed by 40 min of elastic band resistance exercises and 5 min of cooling-down exercises at the end. The resistance exercises targeted all major muscle groups in the shoulders, arms, lower limbs, chest, and abdomen, with 1–2 exercises included for each muscle group. A total of 3 sets of 10 repetitions of gentle concentric and eccentric contractions through the full range of motion were performed for each exercise. The resistance levels of the elastic bands were indicated by different band colors (yellow, red, green, blue, black, or silver), and the resistance intensity was increased by 25% from 3 to 15.3 pounds during full elongation. The resistance was set depending on the perception of participants. Participants began by performing exercises with the yellow band, which had the lowest resistance intensity, and their exertion was assessed using the Borg scale. When participants rated their perceived effort at 13 (somewhat difficult) on the Borg scale, the intensity was increased to the next level. If participants were unable to tolerate the new resistance level, then they continued with the previous

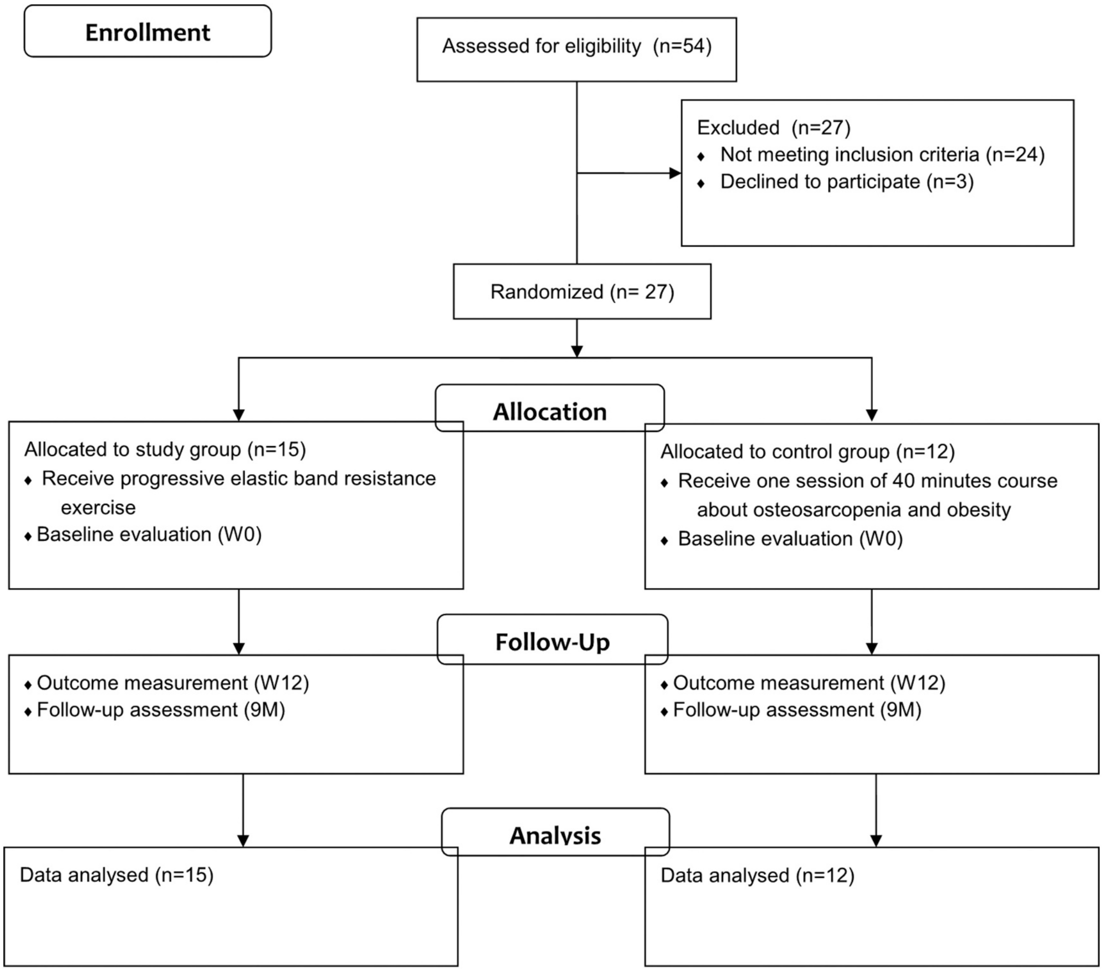


**Fig. 1.** Flow diagram of participant selection.

resistance level for several sessions until their strength had sufficiently increased. To ensure that all participants received treatment, they performed the exercises at the rehabilitation department of our hospital and were required to complete 36 intervention sessions. To ensure intervention quality, a standard intervention program was conducted under the supervision of a senior physical therapist in accordance with a treatment manual.

Participants in the control group attended a 40-min group lecture and received a booklet with educational content regarding OSA and home exercise instructions with pictorial demonstrations. The exercise instruction content was the same as that for the intervention group. After the lecture, participants were given a yellow elastic band and allowed to exercise at home.

### 2.7. Outcome measurements

Anthropometric and other outcome measurements were obtained at baseline and 12 weeks and 6 months after the intervention. Body height was measured using a Leicester stadiometer (Invicta, Leicester, UK), and weight was measured using an electronic scale (Heine, Dover, USA). Total body fat, total BF%, appendicular lean mass (ALM), lean muscle mass index (LMI, whole-body skeletal muscle mass/height (Dawson-Hughes and Bischoff-Ferrari, 2016)), and SMI (ALM/height (Dawson-Hughes and Bischoff-Ferrari, 2016)) were calculated using data obtained from the DXA. Physical capacity assessments performed in this study were as follows: 1. *Functional forward reach (FFR):* FFR was measured to assess balance performance and is defined as the maximal distance that the participant could reach forward beyond an arm's length while standing still. 2. *Single leg stance (SLS):* SLS was used to assess balance control ability. SLS is measured as the time for which one could stand on the dominant leg with one eye open and one eye closed. 3. *GS*: GS was defined as the time required for a patient to walk 10 m at a self-selected pace. 4. *Timed up and go (TUG) test*: This test was used to measure the time required for a patient to rise from a chair (height, 42 cm; depth, 26 cm), walk 3 m away, and walk back and be seated in the chair at a self-selected speed. A walking aid was used, if necessary. 5. *Timed chair rise (TCR) test*: In this test, participants were asked to stand up from a seated position in a chair (height, 43 cm) with arms folded and to sit down as many times as they could in 30 s. 6. *Grip strength*: The grip strength of the participant's dominant hand was measured using a standard hydraulic hand dynamometer (Baseline® Digital, Fabrication Enterprises Inc., New York City, NY, USA). Participants were asked to remain in a seated position with arms adducted, elbows flexed at 90°, and the unsupported forearm and wrist in a neutral position. The mean force of three measured maximal contractions caused by gripping the dynamometer for 3–5 s was recorded as the grip strength. FFR, SLS, GS, TUG, and TCR tests were performed once as a practice trial, and the mean of two subsequent tests was formally recorded. All the aforementioned tests have been validated for application to older adults in other studies (Jones et al., 1999; Shumway-Cook et al., 2000; Gill and McBurney, 2008; Choi et al., 2014; Takacs et al., 2014). All outcome measures were assessed by a blinded examiner.

### 2.8. Statistical analysis

Demographic and baseline data were analyzed for each group. For continuous variables, we used the Mann–Whitney *U* test to evaluate the differences between the groups. Categorical variables are presented as numbers and proportions, and the chi-squared test was performed to compare differences between the study and control groups. Outcome differences within the study and control groups at different evaluation times were analyzed using repeated-measures analysis of variance with a post hoc test. An independent statistician performed all analyses by using SPSS (version 20.0). The significance level was defined as $p < 0.05$, and analyses were based on the intention-to-treat principle. Missing data were substituted using modern imputation methods and analyzed using a set of repeated imputations performed through predictive models. A change in the TUG score of 4.09 s was used as the minimal clinically significant difference in a study on elderly patients with dementia.(Ries et al., 2009) The power was calculated based on the minimal clinically important difference in the change in TUG. By using GPower 3.1, we defined the statistical power as 0.80 and the significance level as 0.05. The sample size was 12 in each group ($n = 24$).

## 3. Results

No adverse event was observed after the intervention in any of the study participants. No dropouts were recorded during the study period. Moreover, a comparison of demographic data indicated no significant difference between the groups (Table 1). Furthermore, no significant differences in BF%, absolute muscle mass (TSM and appendicular limb muscle mass), relative muscle mass (LMI and SMI), and BMD of spine were observed between the groups at baseline, 3 months, and 9 months (Table 2). After the 12-week exercise intervention, BF%, TSM, and LMI exhibited a trend of improvement; however, within- or between-group comparisons indicated no significant differences. By contrast, BMD and the T-score of spine were significantly increased in the study group ($p = 0.022$ and 0.026, respectively) compared with those at baseline, but the positive effect was not maintained at 6-month follow-up. Regarding the physical capacity assessment, GS, TUG test scores, and TCR scores revealed significant improvements in the study group compared with the control group after the intervention ($p = 0.012$, 0.030, and 0.044, respectively), but the improvement was not maintained at 9 months. No significant differences in FFR, GS, and SLS were observed between the groups. FFR scores were significantly improved compared with those at baseline in the study group, and this improvement was maintained at 6-month follow-up (at 9 months) after intervention. Moreover, GS and the TUG score were significantly improved in the study group compared with those at baseline at 3 months after the intervention, but no significant difference was noted at 9 months after the intervention. However, grip strength and SLS scores exhibited no changes after exercise training (Table 3).

## 4. Discussion

The main findings of this study are the significant improvements in BMD/T-score of spine; and physical capacity parameters, namely FFR, GS, TUG test scores, and TCR test scores. Among them, the improvements in BMD/T-score of spine, GS, TUG test scores, and TCR test scores were significantly greater in the study group than in the control group; however, the improvement in the TUG test score was not significantly different between the groups. Moreover, no significant differences were observed between the groups after the intervention in terms of BF%, absolute muscle mass, relative muscle mass, or BMD of spine. Except for FFR, the significant changes in no other indicator were maintained at the 6-month evaluation.

Ilich et al. (Ilich et al., 2015) reported an association of OSA with decreased handgrip strength, walking ability, and balance, which led to increased risks of falls and fractures. The positive effect of conventional or nonconventional exercise was proposed in another study (Kelly and

**Table 1**
Patient demographics.

| Variables | Study group ($n = 15$) | Control group ($n = 12$) | $p$ value |
|---|---|---|---|
| Age (years) | 70.13 (4.41) | 71.82 (5.23) | 0.382 |
| Height (cm) | 150.40 (5.29) | 150.23 (5.61) | 0.937 |
| Weight (kg) | 60.94 (7.93) | 65.21 (7.54) | 0.179 |
| [a]BMI (kg/m$^2$) | 26.95 (3.31) | 28.93 (3.55) | 0.156 |

All numbers are presented as means (standard deviation).
$p$ value was calculated using the Mann–Whitney $U$ test.
[a] BMI = body mass index.

**Table 2**
Body composition and muscle mass of the participants in the study and control groups.

| Measurements | Study group, $n = 15$, Mean (SE) | p value[a] | Control group, n = 12, Mean (SE) | p value[a] | p value[b] |
|---|---|---|---|---|---|
| Body fat percentage | | | | | |
| Baseline | 41.27 (6.07) | | 44.20 (7.71) | | 0.574 |
| Month 3 | 38.58 (8.34) | 0.251 | 43.24 (7.43) | 0.645 | 0.148 |
| Month 9 | 39.91 (5.06) | 0.404 | 44.03 (6.80) | 0.930 | 0.097 |
| Absolute muscle mass | | | | | |
| TSM (kg) | | | | | |
| Baseline | 33.46 (4.39) | | 34.46 (3.97) | | 0.646 |
| Month 3 | 31.46 (8.79) | 0.391 | 34.04 (3.88) | 0.287 | 0.474 |
| Month 9 | 33.14 (4.22) | 0.320 | 33.84 (3.58) | 0.080 | 0.646 |
| ALM (kg) | | | | | |
| Baseline | 13.59 (1.90) | | 13.76 (2.05) | | 0.919 |
| Month 3 | 13.58 (2.14) | 0.980 | 13.56 (1.91) | 0.397 | 1.000 |
| Month 9 | 13.64 (2.27) | 0.832 | 13.27 (2.05) | 0.139 | 0.474 |
| Relative muscle mass | | | | | |
| LMI (kg/m$^2$) | | | | | |
| Baseline | 14.80 (1.95) | | 15.32 (2.05) | | 0.413 |
| Month 3 | 13.84 (3.75) | 0.383 | 15.11 (1.79) | 0.237 | 0.330 |
| Month 9 | 14.66 (1.84) | 0.328 | 15.03 (1.74) | 0.079 | 0.474 |
| SMI (kg/m$^2$) | | | | | |
| Baseline | 5.01 (0.83) | | 5.12 (1.00) | | 0.799 |
| Month 3 | 5.01 (0.94) | 0.980 | 5.01 (0.82) | 0.342 | 0.838 |
| Month 9 | 5.03 (0.98) | 0.847 | 5.13 (0.90) | 0.152 | 0.259 |
| Bone density parameters | | | | | |
| BMD of spine | | | | | |
| Baseline | 0.948 (0.055) | | 0.922 (0.061) | | 0.305 |
| Month 3 | 0.987 (0.081) | 0.022* | 0.964 (0.104) | 0.052 | 0.721 |
| Month 9 | 0.959 (0.088) | 0.492 | 0.922 (0.067) | 0.910 | 0.574 |
| T-score of spine | | | | | |
| Baseline | −2.070 (0.773) | | −2.430 (0.880) | | 0.305 |
| Month 3 | −1.573 (1.070) | 0.026* | −1.891 (1.443) | 0.058 | 0.760 |
| Month 9 | −1.940 (1.195) | 0.573 | −2.445 (0.974) | 0.852 | 0.574 |

[a] Difference between baseline and 3, 6 months. *p* value was calculated using repeated-measures analysis of variance with a post hoc test.
[b] Difference between the groups, *p* value was calculated using the Mann–Whitney U test.
* $p < 0.05$, TSM = total skeletal muscle mass; ALM = appendicular lean mass; LMI = lean muscle mass index (whole-body skeletal muscle mass/height (Dawson-Hughes and Bischoff-Ferrari, 2016)); SMI = skeletal muscle mass index (appendicular lean mass/height (Dawson-Hughes and Bischoff-Ferrari, 2016)); and BMD = bone mineral density.

**Table 3**
Physical capacity and function outcome measures of the study group and control group at various time points.

| Evaluation | Study group, n = 15, Mean (SE) | p value[a] | Control group, n = 12, Mean (SE) | p value[a] | p value[b] |
|---|---|---|---|---|---|
| Physical capacity | | | | | |
| FFR (cm) | | | | | |
| Baseline | 98.27 (11.32) | | 100.77 (10.83) | | 0.610 |
| Month 3 | 108.87 (10.43) | <0.001** | 106.64 (11.33) | 0.053 | 0.507 |
| Month 9 | 108.80 (8.92) | 0.021* | 104.76 (9.80) | 0.320 | 0.330 |
| Grip strength (kg) | | | | | |
| Baseline | 20.40 (4.00) | | 19.34 (6.36) | | 0.799 |
| Month 3 | 20.35 (3.53) | 0.957 | 18.11 (5.91) | 0.394 | 0.413 |
| Month 9 | 19.40 (3.85) | 0.136 | 17.19 (5.42) | 0.086 | 0.281 |
| SLS (s) | | | | | |
| Baseline | 3.21 (2.45) | | 3.17 (2.26) | | 0.305 |
| Month 3 | 3.36 (2.02) | 0.803 | 2.89 (1.60) | 0.667 | 0.198 |
| Month 9 | 3.25 (2.07) | 0.831 | 2.10 (0.87) | 0.123 | 0.077 |
| GS (m/s) | | | | | |
| Baseline | 0.82(0.21) | | 0.90(0.21) | | 0.061 |
| Month 3 | 0.71(0.21)* | <0.001** | 0.88(0.19) | 0.590 | 0.128* |
| Month 9 | 0.81(0.20) | 0.239 | 0.87(0.21) | 0.383 | 0.154 |
| TUG test (s) | | | | | |
| Baseline | 9.58 (2.39) | | 9.67 (3.12) | | 0.077 |
| Month 3 | 7.63 (0.96) | <0.001** | 9.94 (2.99) | 0.374 | 0.030* |
| Month 9 | 8.98 (1.20) | 0.452 | 9.26 (3.21) | 0.374 | 0.068 |
| TCR test (repetitions) | | | | | |
| Baseline | 15.47 (4.03) | | 14.52 (4.76) | | 0.109 |
| Month 3 | 17.13 (2.23) | 0.117 | 13.27 (5.39) | 0.153 | 0.044* |
| Month 9 | 15.20 (2.51) | 0.434 | 14.73 (5.82) | 0.068 | 0.086 |

FFR = functional forward reach; SLS = single leg stance; GS = gait speed; TUG = timed up and go; and TCR = timed chair rise.
[a] Difference between baseline and 3, 6 months.*p* value was calculated using the repeated-measures analysis of variance with a post hoc test.
[b] Difference between the groups.*p* value was compared with the control group and measured using the Mann–Whitney *U* test.
* $p < 0.05$.
** $p < 0.05$.

Gilman, 2017). Our exercise program, which targeted all major muscle groups, efficiently improved mobility in postmenopausal women with OSA after 12 weeks of intervention, potentially reducing the risks of falls and fractures. This result is compatible with that of a previous study that reported that elastic resistance exercise improved the mobility and functional performance of older women (Urzi et al., 2019). However, in our study, the detraining effect was evident, and positive effects diminished after regular resistant training was stopped; these findings are in accordance with those reported in other studies (Mujika and Padilla, 2001; Rossi et al., 2017). To preserve the positive effects, continuing to perform exercises regularly is likely necessary.

Theoretically, resistance training can increase BMD and muscle mass. Our study results revealed a significant increase in BMD of spine in the study group but no significant difference was noted between the groups. Muscle mass and body fat exhibited a nonsignificant trend of improvement. These results indicate that changing the body composition of patients with OSA is challenging and complicated, which can be attributed to several factors. First, the response of elderly women with OSA to exercise may be blunted. A study reported that ribosome biogenesis was decreased in elderly participants who underwent resistance training (Stec et al., 2015), indicating that resistance training–induced muscle hypertrophy is attenuated by aging. Poor insulin sensitivity may be another cause of the blunted response of patients with OSA to exercise because it has been demonstrated to reduce muscle protein breakdown and stimulate muscle protein synthesis (Moller-Loswick et al., 1994; Nygren and Nair, 2003). This finding is compatible with that reported by Geirsdottir et al. (Geirsdottir et al., 2019), who discovered that participants with obesity exhibited less favorable changes in body composition and physical function after a resistance exercise program compared with participants with healthy body weights. Second, because age and obesity can interfere with metabolism in patients with OSA, the training intensity may be insufficient for stimulating adequate osteogenic or muscle synthesis processes. We selected elastic bands for resistance training mainly because of their safety for use and tolerability among older adults. This could be one of the reasons for our observation of the preservation of handgrip strength rather than its increase after peRET; the intensity provided by elastic bands could have been insufficient, making our intervention more likely to be a muscle-endurance training program rather than a strength training program. Cunha et al. (Cunha et al., 2018) reported that a high training volume exerted a positive effect on patients with OSA. In a recent trial, Watson et al. (Watson et al., 2018) reported that BMD and muscle strength were significantly improved after high-intensity resistance and impact training for 8 months in postmenopausal women with osteoporosis and osteopenia. These results indicate the potential benefits of applying higher intensity resistance training combined with

impact activity in patients with OSA for improving outcomes; however, the risk of injury caused by exercise as a result of patients' weight and sarcopenia should be carefully assessed before implementing high-intensity training. Third, nutritional supplementation and dietary interventions may be crucial during the exercise intervention in this group of patients. In their recently published meta-analysis, Liao et al.(Liao et al., 2017) indicated that protein supplementation in addition to resistance training was more effective in preventing muscle mass and strength loss in older adults with obesity. Two recent studies (Kelly et al., 2016; Inglis and Ilich, 2015) have also suggested the importance of micronutrients and microbiomes in OSA prevention and treatment. An optimal dose response has not yet been established, and further exploratory studies should be conducted in the future. Finally, the small sample size of the study and inadequate statistical power of the control group may have caused selection bias. Furthermore, the study group was divided into small exercise training groups, which might lead to study group bias in terms of functional outcome measurement.

Because of the characteristics of the OSA population, changing the body composition and physical capacity of these individuals is challenging. This is the first randomized controlled study with a short-term follow-up to investigate the effect of a 12-week progressive resistance training program on elderly women with OSA. The results revealed positive effects of the progressive elastic resistance training program.

### *4.1. Strengths and limitations*

There were no dropouts or adverse events noted during the study, indicating the satisfactory acceptability and safety of elastic band exercise in the population. However, our study has some limitations. First, this trial was a study with a small sample size. Additional well-powered randomized controlled trials based on the results of the present study are warranted to examine the effects of progressive elastic resistance training as an intervention in elderly women with OSA. Second, there were no men included in this study, so our results cannot be generalized to elderly men with OSA. Third, in our study, the effect of exercise on BMD/T-score of hip was not assessed, thus the results of this study should be interpreted carefully. Further study on what exercise program can benefit BMD of hip in OSA population is warranted.

## 5. Conclusions

A 12-week peRET program effectively increased the BMD of spine and physical capacity of patients; however, when training was stopped, these positive effects could not be maintained at the 6-month follow-up. Because of the complicated nature of OSA, no significant differences were observed between the study and control groups in terms of changes in body composition. More tailored exercise intervention programs for this population should be investigated in the future.

## Declaration of competing interest

None.

## Acknowledgment

This work was supported by the National Science Council of Taiwan (grant no. NSC 102-2314-B-038-053-MY3) and Taipei Medical University-Wan Fang Hospital, Taiwan (grant no. 98TMU-WFH-05-3). The funding source played no role in the design, implementation, data analysis, interpretation, or reporting of this study. The contents of this publication are solely the responsibility of authors and do not necessarily represent the official views of the funding sources.

## References

Alalwan, T.A., 2020. Phenotypes of sarcopenic obesity: exploring the effects on peri-muscular fat, the obesity paradox, hormone-related responses and the clinical implications. Geriatrics (Basel, Switzerland) 5 (1).

Anderson, L.J., Erceg, D.N., Schroeder, E.T., 2012. Utility of multifrequency bioelectrical impedance compared with dual-energy x-ray absorptiometry for assessment of total and regional body composition varies between men and women. Nutrition research (New York, NY) 32 (7), 479–485.

Atkins, J.L., Whincup, P.H., Morris, R.W., Lennon, L.T., Papacosta, O., Wannamethee, S. G., 2014. Sarcopenic obesity and risk of cardiovascular disease and mortality: a population-based cohort study of older men. J. Am. Geriatr. Soc. 62 (2), 253–260.

Binkley, N., Buehring, B., 2009. Beyond FRAX: it's time to consider "sarco-osteopenia". J. Clin. Densitom. 12 (4), 413–416.

Calle, M.C., Fernandez, M.L., 2010. Effects of resistance training on the inflammatory response. Nutr. Res. Pract. 4 (4), 259–269.

Chen, K.M., Tseng, W.S., Huang, H.T., Li, C.H., 2013. Development and feasibility of a senior elastic band exercise program for aged adults: a descriptive evaluation survey. J. Manipulative Physiol. Ther. 36 (8), 505–512.

Choi, Y.M., Dobson, F., Martin, J., Bennell, K.L., Hinman, R.S., 2014. Interrater and intrarater reliability of common clinical standing balance tests for people with hip osteoarthritis. Phys. Ther. 94 (5), 696–704.

Chung, J.H., Hwang, H.J., Shin, H.Y., Han, C.H., 2016. Association between sarcopenic obesity and bone mineral density in middle-aged and elderly Korean. Ann Nutr Metab 68 (2), 77–84.

Cruz-Jentoft, A.J., Baeyens, J.P., Bauer, J.M., Boirie, Y., Cederholm, T., Landi, F., et al., 2010. Sarcopenia: European consensus on definition and diagnosis: report of the European Working Group on sarcopenia in older people. Age Ageing 39 (4), 412–423.

Cunha, P.M., Ribeiro, A.S., Tomeleri, C.M., Schoenfeld, B.J., Silva, A.M., Souza, M.F., et al., 2018. The effects of resistance training volume on osteosarcopenic obesity in older women. J. Sports Sci. 36 (14), 1564–1571.

Curtis, E., Litwic, A., Cooper, C., Dennison, E., 2015. Determinants of muscle and bone aging. J. Cell. Physiol. 230 (11), 2618–2625.

Dawson-Hughes, B., Bischoff-Ferrari, H., 2016. Considerations concerning the definition of sarcopenia. Osteoporosis international : a journal established as result of cooperation between the European Foundation for Osteoporosis and the National Osteoporosis Foundation of the USA 27 (11), 3139–3144.

Ding J, Kritchevsky SB, Newman AB, Taaffe DR, Nicklas BJ, Visser M et al. Effects of birth cohort and age on body composition in a sample of community-based elderly. Am. J. Clin. Nutr. 2007;85(2):405–10.

Foley, S., Quinn, S., Jones, G., 2010. Pedometer determined ambulatory activity and bone mass: a population-based longitudinal study in older adults. Osteoporosis international : a journal established as result of cooperation between the European Foundation for Osteoporosis and the National Osteoporosis Foundation of the USA 21 (11), 1809–1816.

Geirsdottir, O.G., Chang, M., Jonsson, P.V., Thorsdottir, I., Ramel, A., 2019. Obesity, physical function, and training success in community-dwelling nonsarcopenic old adults. J Aging Res 2019, 5340328.

Gill, S., McBurney, H., 2008. Reliability of performance-based measures in people awaiting joint replacement surgery of the hip or knee. Physiotherapy research international : the journal for researchers and clinicians in physical therapy 13 (3), 141–152.

Hirani, V., Naganathan, V., Blyth, F., Le Couteur, D.G., Seibel, M.J., Waite, L.M., et al., 2017. Longitudinal associations between body composition, sarcopenic obesity and outcomes of frailty, disability, institutionalisation and mortality in community-dwelling older men: the Concord health and ageing in men project. Age Ageing 46 (3), 413–420.

Hofmann, M., Schober-Halper, B., Oesen, S., Franzke, B., Tschan, H., Bachl, N., et al., 2016. Effects of elastic band resistance training and nutritional supplementation on muscle quality and circulating muscle growth and degradation factors of institutionalized elderly women: the Vienna active ageing study (VAAS). Eur. J. Appl. Physiol. 116 (5), 885–897.

Hong, W., Cheng, Q., Zhu, X., Zhu, H., Li, H., Zhang, X., et al., 2015. Prevalence of sarcopenia and its relationship with sites of fragility fractures in elderly Chinese men and women. PLoS One 10 (9), e0138102.

Huang, S.W., Ku, J.W., Lin, L.F., Liao, C.D., Chou, L.C., Liou, T.H., 2017. Body composition influenced by progressive elastic band resistance exercise of sarcopenic obesity elderly women: a pilot randomized controlled trial. European journal of physical and rehabilitation medicine 53 (4), 556–563.

Ilich, J.Z., Kelly, O.J., Inglis, J.E., Panton, L.B., Duque, G., Ormsbee, M.J., 2014. Interrelationship among muscle, fat, and bone: connecting the dots on cellular, hormonal, and whole body levels. Ageing Res. Rev. 15, 51–60.

Ilich, J.Z., Inglis, J.E., Kelly, O.J., McGee, D.L., 2015. Osteosarcopenic obesity is associated with reduced handgrip strength, walking abilities, and balance in postmenopausal women. Osteoporosis international : a journal established as result of cooperation between the European Foundation for Osteoporosis and the National Osteoporosis Foundation of the USA 26 (11), 2587–2595.

Ilich, J.Z., Kelly, O.J., Inglis, J.E., 2016. Osteosarcopenic obesity syndrome: what is it and how can it be identified and diagnosed? Curr Gerontol Geriatr Res 2016, 7325973.

Ilich, J.Z., Gilman, J.C., Cvijetic, S., Boschiero, D., 2020. Chronic stress contributes to osteosarcopenic adiposity via inflammation and immune modulation: the case for more precise nutritional investigation. Nutrients 12 (4).

Inglis, J.E., Ilich, J.Z., 2015. The microbiome and osteosarcopenic obesity in older individuals in long-term care facilities. Curr Osteoporos Rep 13 (5), 358–362.

Janssen, I., Heymsfield, S.B., Baumgartner, R.N., Ross, R., 2000. Estimation of skeletal muscle mass by bioelectrical impedance analysis. J Appl Physiol (1985) 89 (2), 465–471.
Janssen, I., Heymsfield, S.B., Ross, R., 2002. Low relative skeletal muscle mass (sarcopenia) in older persons is associated with functional impairment and physical disability. J. Am. Geriatr. Soc. 50 (5), 889–896.
Jones, C.J., Rikli, R.E., Beam, W.C., 1999. A 30-s chair-stand test as a measure of lower body strength in community-residing older adults. Res. Q. Exerc. Sport 70 (2), 113–119.
Kanis, J.A., McCloskey, E.V., Johansson, H., Cooper, C., Rizzoli, R., Reginster, J.-Y., 2013. European guidance for the diagnosis and management of osteoporosis in postmenopausal women. Osteoporos. Int. 24 (1), 23–57.
Kelly, O.J., Gilman, J.C., 2017. Can unconventional exercise be helpful in the treatment, management and prevention of osteosarcopenic obesity? Curr Aging Sci 10 (2), 106–121.
Kelly, O.J., Gilman, J.C., Kim, Y., Ilich, J.Z., 2016. Micronutrient intake in the etiology, prevention and treatment of osteosarcopenic obesity. Curr Aging Sci 9 (4), 260–278.
Li, L., Wang, C., Bao, Y., Peng, L., Gu, H., Jia, W., 2012. Optimal body fat percentage cut-offs for obesity in Chinese adults. Clin. Exp. Pharmacol. Physiol. 39 (4), 393–398.
Liao, C.D., Tsauo, J.Y., Wu, Y.T., Cheng, C.P., Chen, H.C., Huang, Y.C., et al., 2017. Effects of protein supplementation combined with resistance exercise on body composition and physical function in older adults: a systematic review and meta-analysis. Am. J. Clin. Nutr. 106 (4), 1078–1091.
Liao, C.D., Tsauo, J.Y., Huang, S.W., Ku, J.W., Hsiao, D.J., Liou, T.H., 2018. Effects of elastic band exercise on lean mass and physical capacity in older women with sarcopenic obesity: a randomized controlled trial. Sci. Rep. 8 (1), 2317.
Management of osteoporosis in postmenopausal women, 2010. 2010 position statement of the North American Menopause Society. Menopause 17 (1), 25–54.
Moller-Loswick, A.C., Zachrisson, H., Hyltander, A., Korner, U., Matthews, D.E., Lundholm, K., 1994. Insulin selectively attenuates breakdown of nonmyofibrillar proteins in peripheral tissues of normal men. Am. J. Phys. 266 (4 Pt 1), E645–E652.
Mujika, I., Padilla, S., 2001. Muscular characteristics of detraining in humans. Med. Sci. Sports Exerc. 33 (8), 1297–1303.
Nelson, M.E., Rejeski, W.J., Blair, S.N., Duncan, P.W., Judge, J.O., King, A.C., et al., 2007. Physical activity and public health in older adults: recommendation from the American College of Sports Medicine and the American Heart Association. Circulation 116 (9), 1094–1105.
Newman, A.B., Kupelian, V., Visser, M., Simonsick, E.M., Goodpaster, B.H., Kritchevsky, S.B., et al., 2006. Strength, but not muscle mass, is associated with mortality in the health, aging and body composition study cohort. J. Gerontol. A Biol. Sci. Med. Sci. 61 (1), 72–77.
Nygren, J., Nair, K.S., 2003. Differential regulation of protein dynamics in splanchnic and skeletal muscle beds by insulin and amino acids in healthy human subjects. Diabetes 52 (6), 1377–1385.
Ries, J.D., Echternach, J.L., Nof, L., Gagnon Blodgett, M., 2009. Test-retest reliability and minimal detectable change scores for the timed "up & go" test, the six-minute walk test, and gait speed in people with Alzheimer disease. Phys. Ther. 89 (6), 569–579.
Romagnoli, E., Carnevale, V., Nofroni, I., D'Erasmo, E., Paglia, F., De Geronimo, S., et al., 2004. Quality of life in ambulatory postmenopausal women: the impact of reduced bone mineral density and subclinical vertebral fractures. Osteoporosis international : a journal established as result of cooperation between the European Foundation for Osteoporosis and the National Osteoporosis Foundation of the USA 15 (12), 975–980.
Rossi, F.E., Diniz, T.A., Neves, L.M., Fortaleza, A.C.S., Gerosa-Neto, J., Inoue, D.S., et al., 2017. The beneficial effects of aerobic and concurrent training on metabolic profile and body composition after detraining: a 1-year follow-up in postmenopausal women. Eur. J. Clin. Nutr. 71 (5), 638–645.
Ryan, A.S., Ivey, F.M., Hurlbut, D.E., Martel, G.F., Lemmer, J.T., Sorkin, J.D., et al., 2004. Regional bone mineral density after resistive training in young and older men and women. Scand. J. Med. Sci. Sports 14 (1), 16–23.
Sakai, Y., Ito, H., Meno, T., Numata, M., Jingu, S., 2006. Comparison of body composition measurements obtained by two fan-beam DXA instruments. J. Clin. Densitom. 9 (2), 191–197.
Shumway-Cook, A., Brauer, S., Woollacott, M., 2000. Predicting the probability for falls in community-dwelling older adults using the timed up & go test. Phys. Ther. 80 (9), 896–903.
Sowers MF, Kshirsagar A, Crutchfield MM, Updike S. Joint influence of fat and lean body composition compartments on femoral bone mineral density in premenopausal women. Am. J. Epidemiol. 1992;136(3):257–65.
Stec, M.J., Mayhew, D.L., Bamman, M.M., 2015. The effects of age and resistance loading on skeletal muscle ribosome biogenesis. J Appl Physiol (1985) 119 (8), 851–857.
Stenholm, S., Harris, T.B., Rantanen, T., Visser, M., Kritchevsky, S.B., Ferrucci, L., 2008. Sarcopenic obesity: definition, cause and consequences. Curr Opin Clin Nutr Metab Care 11 (6), 693–700.
Stephen, W.C., Janssen, I., 2009. Sarcopenic-obesity and cardiovascular disease risk in the elderly. J. Nutr. Health Aging 13 (5), 460–466.
Szlejf, C., Parra-Rodriguez, L., Rosas-Carrasco, O., 2017. Osteosarcopenic obesity: prevalence and relation with frailty and physical performance in middle-aged and older women. J. Am. Med. Dir. Assoc. 18 (8), 733 e1–e5.
Takacs, J., Garland, S.J., Carpenter, M.G., Hunt, M.A., 2014. Validity and reliability of the community balance and mobility scale in individuals with knee osteoarthritis. Phys. Ther. 94 (6), 866–874.
Tolea, M.I., Chrisphonte, S., Galvin, J.E., 2018. Sarcopenic obesity and cognitive performance. Clin. Interv. Aging 13, 1111–1119.
Trouwborst, I., Verreijen, A., Memelink, R., Massanet, P., Boirie, Y., Weijs, P., et al., 2018. Exercise and nutrition strategies to counteract Sarcopenic obesity. Nutrients 10 (5).
Urzi, F., Marusic, U., Licen, S., Buzan, E., 2019. Effects of elastic resistance training on functional performance and myokines in older women-a randomized controlled trial. J. Am. Med. Dir. Assoc. 20 (7), 830–834.
Van Aller, C., Lara, J., Stephan, B.C.M., Donini, L.M., Heymsfield, S., Katzmarzyk, P.T., et al., 2019. Sarcopenic obesity and overall mortality: results from the application of novel models of body composition phenotypes to the National Health and Nutrition Examination Survey 1999–2004. Clin. Nutr. 38 (1), 264–270.
Watson, S.L., Weeks, B.K., Weis, L.J., Harding, A.T., Horan, S.A., Beck, B.R., 2018. High-intensity resistance and impact training improves bone mineral density and physical function in postmenopausal women with osteopenia and osteoporosis: the LIFTMOR randomized controlled trial. Journal of bone and mineral research : the official journal of the American Society for Bone and Mineral Research 33 (2), 211–220.
WHO, 2007. Scientific group on the assessment of osteoporosis at primary health care level. Available from: URL: http://www.who.int/chp/topics/Osteoporosis.pdf.
Wijnhoven, H.A., Snijder, M.B., van Bokhorst-de van der Schueren, M.A., Deeg, D.J., Visser, M., 2012. Region-specific fat mass and muscle mass and mortality in community-dwelling older men and women. Gerontology 58 (1), 32–40.
